# New Lipophilic Hydroxamates as Promising Trypanocidal Agents: Design, Synthesis, SAR, and Conformational Behavior Studies

**DOI:** 10.1021/acsmedchemlett.4c00111

**Published:** 2024-06-06

**Authors:** George Fytas, Grigoris Zoidis, Antonios Drakopoulos, Martin C. Taylor, John M. Kelly, Alexandra Tsatsaroni, Andrew Tsotinis

**Affiliations:** † Faculty of Pharmacy, Department of Pharmaceutical Chemistry, University of Athens, Panepistimiopolis-Zografou, GR-15771 Athens, Greece; ‡ Department of Chemistry and Molecular Biology, University of Gothenburg, Göteborg SE-412 96, Sweden; § Department of Infection Biology, London School of Hygiene and Tropical Medicine, Keppel Street, London WC1E 7HT, U.K.

**Keywords:** Trypanosoma brucei, acetamidoacetohydroxamic acid derivatives, 2,6-diketopiperazine derivatives, antitrypanosomal activity, toxicity on mammalian cells, conformational behavior studies, NMR

## Abstract

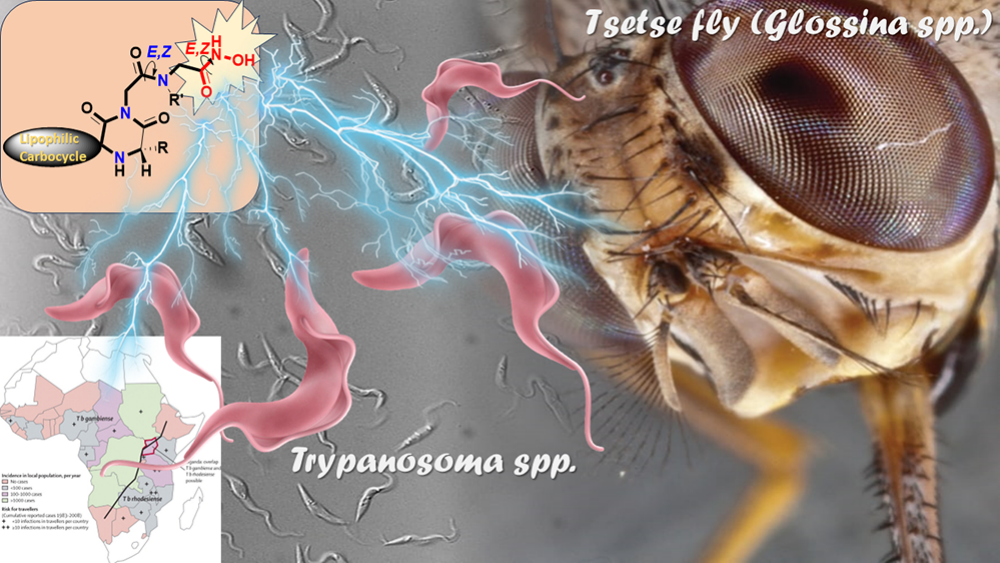

A series of novel hydroxamic acid derivatives was designed and synthesized, and their growth inhibitory activity against bloodstream form *Trypanosoma brucei* was evaluated. These compounds are based on conformationally constrained, lipophilic, spiro carbocyclic 2,6-diketopiperazine (2,6-DKP) scaffolds and bear a side pharmacophoric functionality that contains an acetohydroxamic acid moiety (CH_2_CONHOH) linked with the imidic nitrogen atom of the 2,6-DKP ring via an acetamido portion [CH_2_CON(R), R = H, CH_3_]. Most of these analogues were active in the midnanomolar to low micromolar range against *T*. *brucei*. (*S*)-Isobutyl- or (*S*)-benzyl-substitution on the methylene carbon located between the amine nitrogen atom and carbonyl of the 2,6-DKP ring was studied. The effect of the methyl-substitution on the nitrogen atom of the acetamido portion in the side pharmacophoric functionality was also examined. Compounds **22** and **23**, bearing an isobutyl- or benzyl-substituent, respectively, and concurrently a methyl-substituent, were found to be the most potent hydroxamates of this series (IC_50_ = 34 and 53 nM, respectively). Both had promising selectivity over the parasite compared to mammalian cells (SI = 940 and 470, respectively). Moreover, an *E/Z* conformational behavior study on hydroxamic acid **18** and its methyl-substituted counterpart **21** was undertaken using NMR spectroscopy and theoretical calculations.

Human African trypanosomiasis (HAT or sleeping sickness) results in significant morbidity and suffering. It is caused by protozoan parasites of the *Trypanosoma brucei* species complex, that are transmitted by the tsetse fly and can infect both humans and animals, causing HAT in man and nagana in cattle. Over recent years, a combination of public health measures have considerably reduced the incidence of HAT, although the potential for epidemic outbreaks remains a concern.^1^ In addition, nagana has been an obstruction to the economic development of rural sub-Saharan Africa and a stumbling block to increased production of livestock and agricultural output. The Food and Agriculture Organisation (FAO) estimates that Africa loses US$ 1.5 billion annually in income from agriculture as a result of trypanosomiasis.^2^

In humans, the disease develops in two stages. Patients with early stage disease present with nonspecific symptoms such as fever and weakness. They transition to stage 2 HAT when the parasite crosses the blood brain barrier (BBB). This occurs weeks to years after the initial infection, with the patient developing neurological and psychiatric symptoms such as confusion, lethargy, and convulsions. If left untreated, stage 2 disease is generally fatal.

Due to antigenic variation by the parasite, a vaccine against HAT is unlikely. Drugs that have been used for the treatment of the disease are characterized by high toxicity, low efficacy, high cost and increasing parasitic resistance.^3,4^ Recent drug discovery efforts for HAT have led to the identification of pafuramidine, fexinidazole, and acoziborole.^5^ Among these, fexinidazole is the only *per os* bioavailable drug recently approved for use against both stages of *T*. *b*. *gambiense* HAT.^6^ Despite being a distinct improvement, the treatment regimen involves 10 daily doses under medical supervision, and relapses and toxicity have been reported.^7^ Concurrently, acoziborole has been under clinical evaluation, while the development of pafuramidine has been halted due to adverse renal effects.^8,9^ Thus, additional safe and effective therapeutic agents are needed for the treatment of this devastating parasitic disease, as well as improved drugs for the veterinary sector.

In our previous work, we have reported on a series of spiro carbocyclic 2,6-diketopiperazine-1-acetohydroxamic acid derivatives (Figure 1, compounds **1a**-**g**, **2**, **3**, **4a**-**d**, **4f**, **5a**, **5b**, **5d**) with *T*. *brucei* growth inhibitory potencies in the low nanomolar to submicromolar range without significant toxicity to mammalian cells.^10,11^ These compounds were based on conformationally constrained lipophilic spiro carbocyclic 2,6-DKP scaffold molecules which were modified by introducing an acetohydroxamic acid moiety (CH_2_CONHOH) into their imidic nitrogen. In this way, the combination of a lipophilic spiro carbocyclic 2,6-DKP scaffold and acetohydroxamic acid moiety in one unified chemical entity resulted in potent trypanocidal agents. Structure–activity relationship (SAR) studies showed that the hydroxamic acid group (CONHOH) in these compounds is of vital importance to their trypanocidal activity, since replacement of this group by an amide (CONH_2_), a hydrazide (CONHNH_2_), an *O*-methyl hydroxamate (CONHOCH_3_) or a carboxylic acid (COOH) group resulted in inactive compounds.^10^ On this basis, we hypothesized that these hydroxamates could act by inhibiting essential parasite metalloenzymes, such as members of the iron superoxide dismutase repertoire^12^ or the Trypanosome Alternative Oxidase (TAO),^13^ through the metal-ion binding action of the hydroxamic acid group (via its *Z*(*cis*)-conformation) in the catalytic site. As also shown by the SAR study of this hydroxamate series, molecules containing a benzyl substituent at the position adjacent to the amine nitrogen of the 2,6-DKP ring proved to be the most potent antitrypanosomal agents within this series, with single nanomolar or low midnanomolar IC_50_ values.

**Figure 1:**
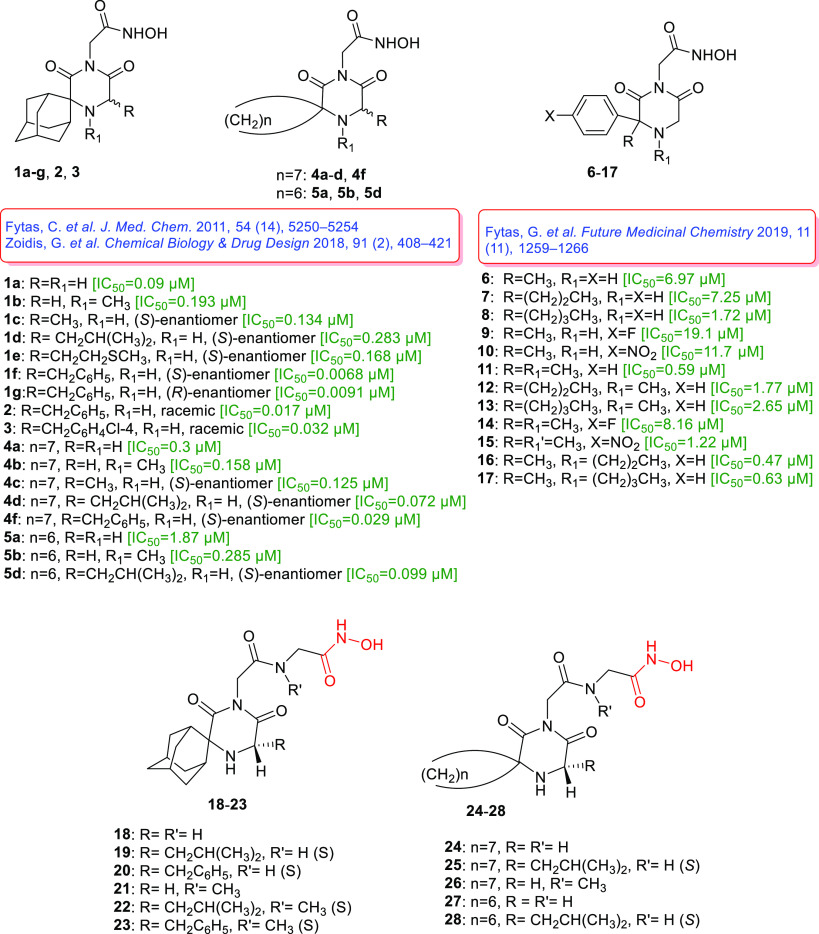
Structures of spiro carbocyclic 2,6-diketopiperazine-1-acetohydroxamic acid derivatives **1**–**5**^10,11^ and 3-alkyl-3-aryl 2,6-diketopiperazine-1-acetohydroxamic acids **6**–**17**^14^ reported previously, along with structures of the new hydroxamic acid derivatives **18**–**28** based on spiro carbocyclic 2,6-diketopiperazine scaffolds.

With the aim of developing hydroxamate compounds of improved potency and selectivity against *T*. *brucei*, we subsequently modified the spiro carbocyclic 2,6-DKP core structure by replacing the spiro-linked carbocyclic component with an alkyl and an aryl substituent. This modification led to a novel series of acetohydroxamic acid derivatives (Figure 1, compounds **6**-**17**) based on conformationally non-constrained 3-alkyl-3-aryl-2,6-DKP scaffolds. The generated hydroxamates **6**-**17** were potent *T*. *brucei* growth inhibitors with submicromolar to low micromolar activities. Additionally, the most active of these compounds had promising selectivity over the parasite with respect to mammalian cells.^14^ SAR studies demonstrated that compounds bearing a methyl, *n*-propyl or *n*-butyl substituent at the *N*(4)-position of the 2,6-DKP ring portion exhibited the highest activity against bloodstream form *T*. *brucei*.

In a search for more potent hydroxamic acid–based trypanocidal agents, and to identify the SAR features that may prove useful in future drug discovery efforts, we designed and synthesized a novel series of hydroxamic acid derivatives (Figure 1, compounds **18**-**28**) as *T*. *brucei* growth inhibitors. These compounds are based on spiro carbocyclic 2,6-DKP scaffolds and characterized by the presence of an acetamido portion (−CH_2_CONR-) between the imidic nitrogen of the spiro carbocyclic 2,6-DKP scaffold and the acetohydroxamic acid moiety (−CH_2_CONHOH). This modification was hoped to lead to an enhanced enzymic metal ion-hydroxamic acid complex stabilization, due to additional interactions between the acetamido portion (−CH_2_CONR-) and sites within the catalytic region of the biological target(s). More specifically, elongating the acetohydroxamic arm with an additional acetamido group could provide more donor atoms, thus improving metal chelation. Furthermore, substituting the *sec*-amide for a *tert*-amide should enhance the lipophilicity of the ligand while also forcing a *Z*-conformation (which was proven beneficial in our previous study) due to steric hindrance. Based on this assumption, compounds **18**, **24** and **27** (Figure 1, R=R′=H) constituted the molecular templates for further SAR studies. We first examined the effect of the (*S*)-isobutyl- or (*S*)-benzyl-substitution on the methylene carbon, as the (*S*)- enantiomer was shown in our previous studies to be more potent than the (*R*)-,^10,11^ located between the amine nitrogen atom and the carbonyl of the 2,6-DKP ring (Figure 1, compounds **19**, **20**, **25** and **28**). We subsequently studied the methyl substitution on the nitrogen atom of the acetamido portion (−CH_2_CONH−) in the side pharmacophoric 2-acetamidoacetohydroxamic acid functionality (−CH_2_CONHCH_2_CONHOH) (Figure 1, compounds **21**-**23** and **26**). The trypanocidal activity of the newly synthesized compounds was assessed against bloodstream form *T*. *brucei in vitro*. In addition, the most active compounds were examined for their toxicity to mammalian cells, using the rat skeletal myoblast L6 cell line.

## Results and Discussion

### Chemistry

**Scheme 1:**
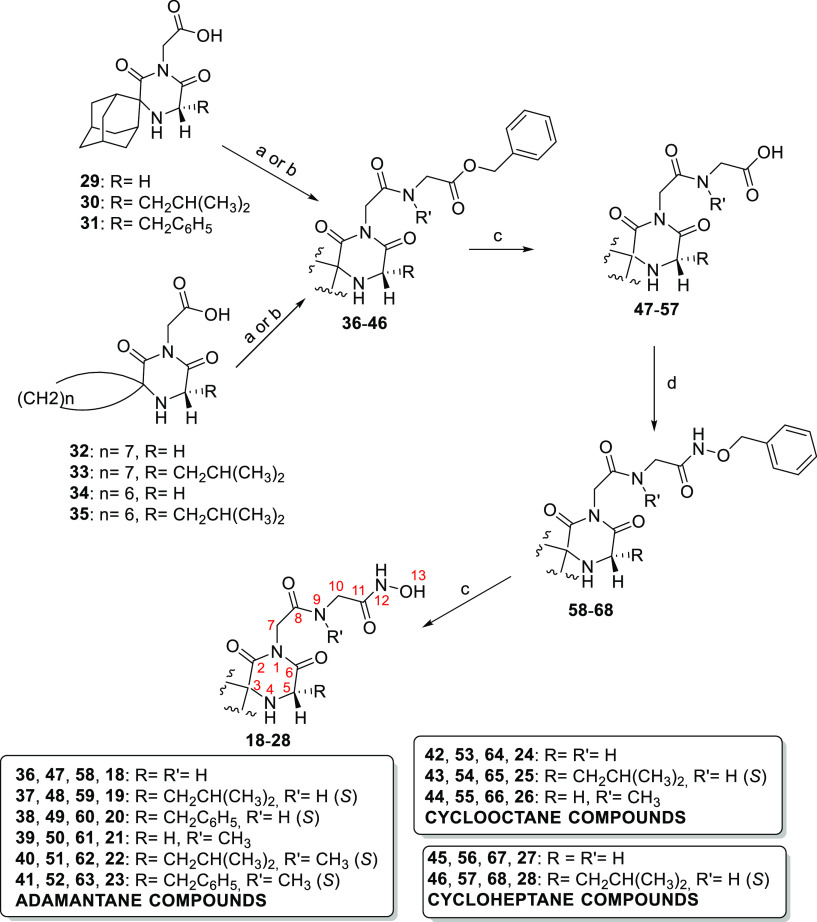
Reagents and conditions: (a) EDCI·HCl, HOBt, ^+^H_3_NCH_2_CO_2_CH_2_C_6_H_5_, TsO^–^, DIEA, CH_2_Cl_2_-DMF 3:5 (v/v), 28 °C, 24h, Ar, for **36-38**, **42**, **43**, **45**, **46**, 61-96%; (b) CDI, THF or THF-DMF 1:1 (v/v) for **44**, 28 °C, 1h, Ar, then ^+^H_2_N(CH_3_)CH_2_CO_2_CH_2_C_6_H_5_, Cl^–^, Et_3_N, 28 °C, 48h, Ar, for **39-41**, **44**, 61-76%; (c) H_2_/Pd–C 10%, abs EtOH or abs EtOH–MeOH 1:1 (v/v) for **47** or abs EtOH-AcOEt 1:1 (v/v) for **50** or MeOH for **18**, **24**, **27** or abs EtOH-AcOEt 3:2 (v/v) for **26**, 50 psi, rt, 3h, ≥ 99% for **47-57**, 88-97% for **18-28**;. (d) EDCI·HCl, HOBt, C_6_H_5_CH_2_ONH_3_^+^ Cl^–^, DIEA, CH_2_Cl_2_-DMF 3:5 (v/v), 28 °C, 24h, Ar, for **58**-**60**, **64**, **65**, **67**, **68**, 57-78%, or CDI, THF, 28 °C, 1h, Ar, then C_6_H_5_CH_2_ONH_3_^+^ Cl^–^, Et_3_N, 28 °C, 48h, Ar, for **61**-**63**, **66**, 64-75%.

Compounds **18**-**28** were synthesized by the procedure depicted in Scheme 1. Chemistry for the preparation of the carboxylic acid building blocks **29**-**35** has been described in our previous publications.^10,11,15^ These carboxylic acids were coupled with glycine or *N*-methylglycine (sarcosine) benzyl ester using either 1-[3-(dimethylamino)propyl]-3-ethylcarbodiimide (EDCI) and 1-hydroxybenzotriazol (HOBt) or 1,1′-carbonyldiimidazol (CDI) as coupling agents. Thus, coupling of acids **29**-**35** with glycine benzyl ester in the presence of EDCI-HOBt gave the respective *N*-substituted glycine benzyl esters **36**-**38**, **42**, **43**, **45** and **46** in good yields (61–96%). However, the EDCI-HOBt coupling reaction between acids **29**-**32** and *N*-methylglycine benzyl ester gives the *N*-methyl counterparts **39**-**41** and **44** in low yields (data not shown). This may be due to the steric hindrance of the *N*-methyl group. Therefore, we decided to employ CDI as coupling agent, and prolong the reaction time from 24 to 48 h. This CDI-mediated coupling reaction allowed us to prepare compounds **39**-**41** and **44** in satisfactory yields (61–76%). Subsequent deprotection of benzyl esters **36**-**46** by catalytic hydrogenolysis (H_2_/10% Pd–C) provided the corresponding *N*-substituted glycine derivatives **47**-**57** in nearly quantitative yields (≥99%). It is noted that the *N*-methylated glycine benzyl esters **39**-**41** and **44**, and their respective glycine amino acid analogues **50**-**52** and **55** appear in the ^1^H and ^13^C NMR spectra as *E* and *Z* conformers (not assigned) due to the hindered rotation around the C(O)-N(CH_3_) amide bond. The nonmethylated glycine analogues **47**-**49**, **53**, **54**, **56** and **57** were subjected to efficient EDCI-HOBt coupling reactions with *O*-benzylhydroxylamine to afford the corresponding *O*-benzyl hydroxamates **58**-**60**, **64**, **65**, **67** and **68** in 57–78% yields. As the EDCI-HOBt coupling reactions of the *N*-methylated glycine analogues **50**-**52** and **55** with *O*-benzylhydroxylamine give products in low yields (<30%), we favored CDI activation coupling in a 48 h reaction time. Thus, we obtained the corresponding methylated *O*-benzyl hydroxamates **61**-**63** and **66** in considerably improved yields (64–75%). Removal of the benzyl-protective group in compounds **58**-**68** by catalytic hydrogenolysis (H_2_/10% Pd–C) afforded the respective hydroxamic acid analogues **18**-**28** in high yields (88–97%).

The ^1^H and ^13^C NMR spectra for the hydroxamic acid analogues **18**-**28** are consistent with *E*/*Z* conformational behavior of these molecules in DMSO solution.^16^ In particular, the hydroxamates **18**-**20**, **24**, **25**, **27** and **28** appear as *E* and *Z* conformers, whereas the methyl-substituted congeners **21**-**23** and **26** appear as four conformers (*EE*, *EZ*, *ZE* and *ZZ*). To determine the *E/Z* conformation, 2D NOESY experiments were conducted for compound **18** and its methyl-substituted counterpart **21**, representatively (SI, NOESY spectra at pages 246 and 247, respectively).

### Computational Studies: Conformational Search and Dihedral Coordinate Scan

To study the conformational profile of the acetamidoacetohydroxamic acid arm, as well as to better understand the impact of the amidic *N*-methyl substitution on its rotation, we conducted a systematic conformational search exemplarily for **18**, **21**, and dihedral coordination scans for the angles C7–C8–N9-H (1_1), C10–C11–N12-H (2_1) in **18** and C7–C8–N9-C9 (1_1), C10–C11–N12-H (2_1) in **21**, using Molecular Mechanics.

**Figure 2:**
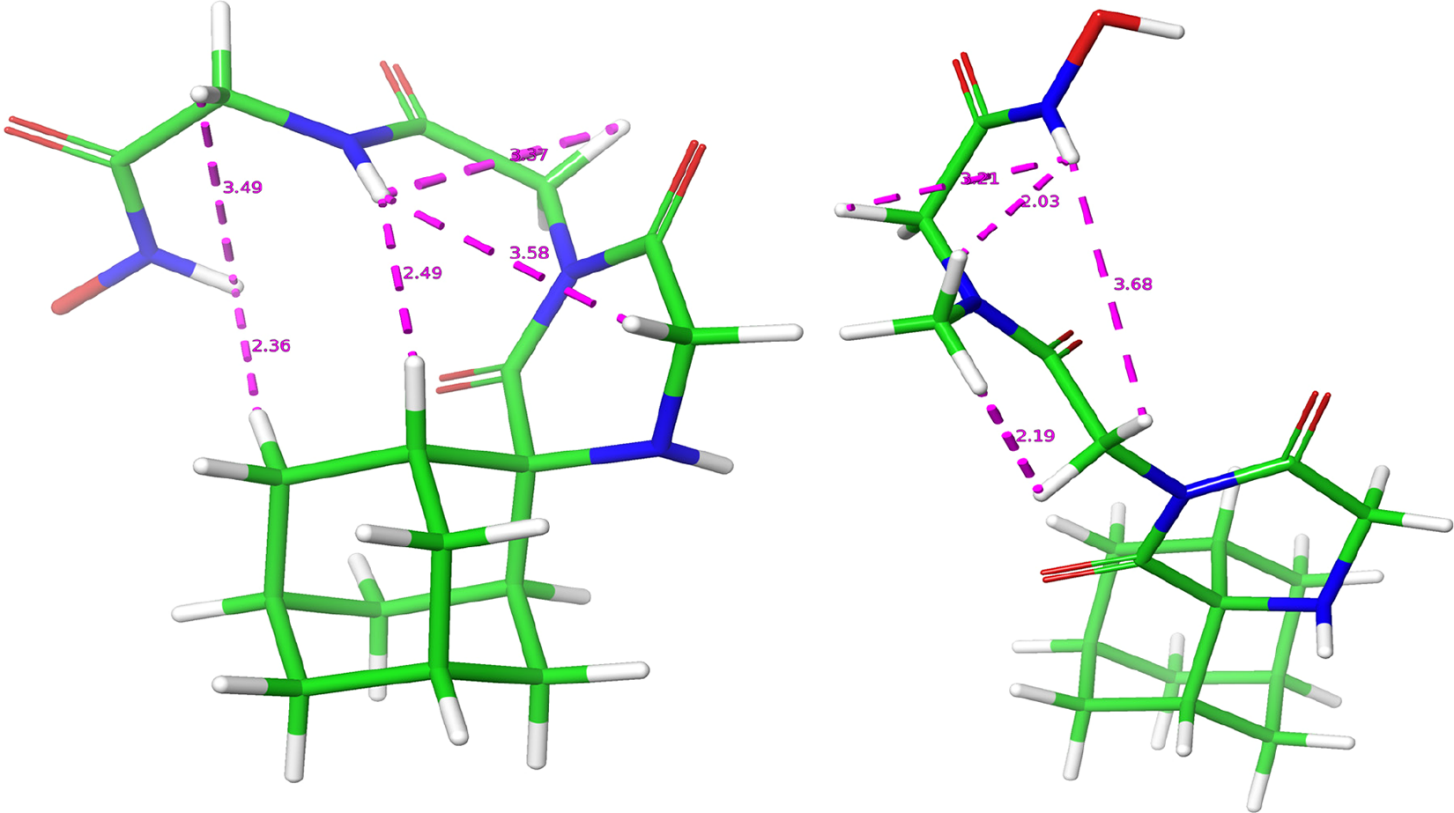
Conformation of minimum energy of **18** (left) and **21** (right). Distances in Å between hydrogens supporting NOE signals are marked in violet. The hydrogen of the hydroxamic OH in **18** is not visible due to the angle of the snapshot; both compounds share the same protonation state (cf. Figures S3–S13).

According to the systematic conformational search for compound **18**, the conformation of minimum energy is the *ZZ* (Figure 2 – the nomenclature convention followed is first to address the conformation of the amide and second of the hydroxamic acid group). The conformer is in agreement with characteristic observations in the NOESY spectra which assign the peaks of the major conformer in the ^1^H NMR spectrum to the *ZZ* conformation, thus validating our molecular modeling results. Most importantly, the distances between 9-NH – 6-CH_2_, 9-NH – (1-Ad)CH as well as 9-NH – 7-CH_2_, 12-NH – 10-CH_2_ and 12-NH – (4,9-Ad)CH_2_ (Figure 2) support the structure elucidation of the NOE signals in the major peaks (SI, NOESY spectrum at page 246). The *ZE* conformer of minimum energy (29/167, relative ΔU = 1.97 kcal/mol) as well as the respective *EZ* conformer (90/167, relative ΔU = 3.49 kcal/mol) are also in accordance with the relevant NOE signals; 9-NH – 6-CH_2_, 9-NH – (1-Ad)CH, 9-NH – 7-CH_2_, 12-OH – 10-CH_2_, and 9-NH – 12-NH, 7-CH_2_ – 10-CH_2_, respectively (Figures S5, S6, SI) (SI, NOESY spectrum at page 246). It is noteworthy to mention that throughout the systematic conformational search of **18**, no *EE* conformer was found. Although an *EE* conformation was expected not to be experimentally assigned for **18** through NMR spectroscopy, due to the fast interconversion rate of the *sec*-amide, its absence in a systematic conformational search suggests that there are additional aspects in the matter. It is possible that in a potential *EE* conformation of **18**, the acetamidoacetohydroxamic acid residue might clash on the adamantane core due to proximity, as the absence of the methyl group could result in a truncated conformer where the linear residue would need to approach too close to the rest of the molecule.

As in **18**, the systematic conformational search of **21** shows that the conformer of minimum energy is *ZZ*. Again, the proton distances measured in the conformer adhere to the NOE signals of the dominant peaks, i.e. 12-NH – 9-CH_3_, 12-NH – 10-CH_2_, 12-NH – 7-CH_2_ and 9-CH_3_ – 7-CH_2_ (Figure 2) (SI, NOESY spectrum at page 247). Following up, the *ZE* conformer of minimum energy (24/315, relative ΔU = 0.48 kcal/mol) adheres to the NOE signals corresponding to the distances of protons 13-OH – 10-CH2, 9-CH_3_ – 7-CH_2_ (Figure S7, SI) (SI, NOESY spectrum at page 247), while the respective *EZ* conformer (26/315, relative ΔU = 0.49 kcal/mol) to 7-CH_2_ – 10-CH_2_, 12-NH – 9-CH_3_ (Figure S8, SI) (SI, NOESY spectrum at page 247). In the case of the bulkier **21**, an *EE* conformation was experimentally found in the NOESY spectrum, and it was assigned to the conformer (55/315, relative ΔU = 0.97 kcal/mol) according to the NOE signals of 7-CH_2_ – 10-CH_2_, 13-OH – 10-CH_2_, 12-NH – 6-CH_2_ (Figure S9, SI) (SI, NOESY spectrum at page 247). In **21** the presence of the methyl group causes a steric hindrance which renders the interconversion rate of the *tert*-amide slower, thus the different chemical exchange of the methyl protons in NMR for *Z* and *E* conformers can be detected under standard conditions. In addition, the presence of the methyl group could prohibit the acetamidoacetohydroxamic acid residue to approach too close to the adamantane ring, thus further enabling an *EE* conformation.

**Figure 3:**
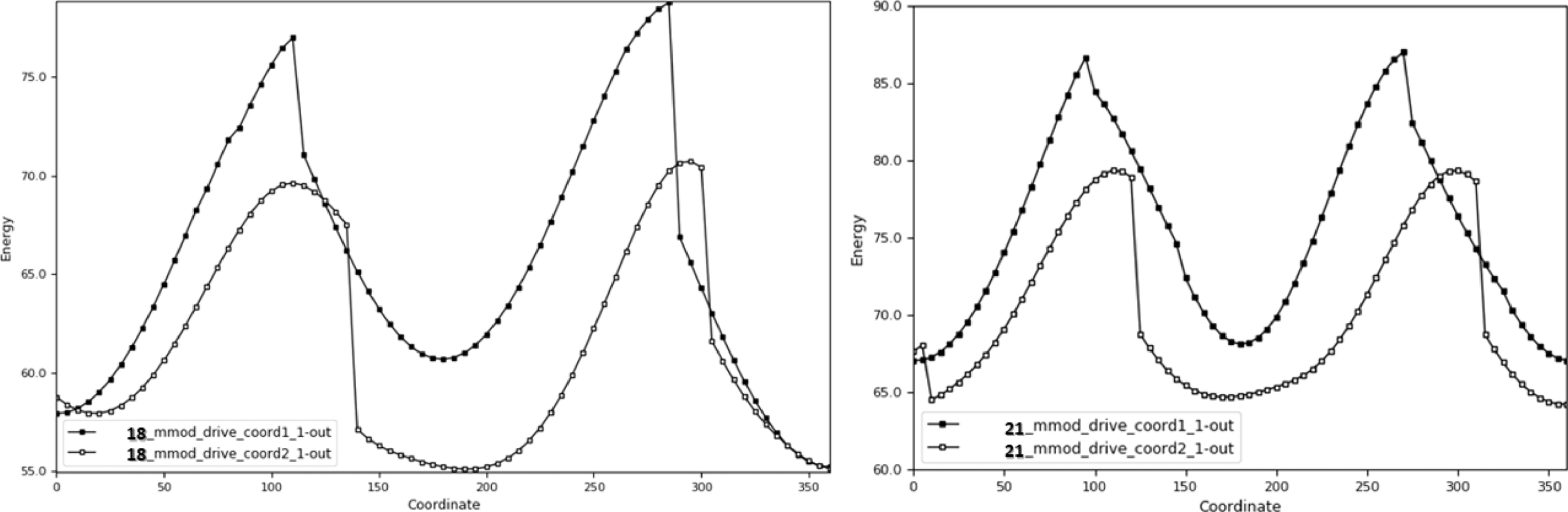
Coordination scan of dihedral angles C7–C8–N9-H (1_1), C10–C11–N12-H (2_1) in **18** (left). Coordination scan of dihedral angles C7–C8–N9-C9 (1_1), C10–C11–N12-H (2_1) in **21** (right). Energy: kcal/mol. Coordinate: degrees.

The coordination scans of the two dihedrals were conducted on a minimized energy structure of the respective compounds (0° - Figures S4, S3, SI) initially one angle at a time while the other remained frozen (Figure 3; Table S2, SI), and in combination for both simultaneously (Figures S1, S2, SI; Table S2, SI). In **18** 1_1 the relative ΔU_**18**_1_ = 23.55 kcal/mol between the global maximum and global minimum, while for 2_1 it is ΔU_**18**_2_ = 15.62 kcal/mol. The combination scan indicates that global minimum energy is reached when 1_1 is 360° and 2_1 is 5°, while the relative ΔU_**18**_C_ = 35.6 kcal/mol compared to the global maximum (Figure S1, SI; Table S2, SI). The respective values for **21** are ΔU_**21**_1_ = 19.94 kcal/mol, ΔU_**21**_2_ = 15.12 kcal/mol and ΔU_**21**_C_ = 31.6 kcal/mol, with global minimum energy reached in the combination scan when 1_1 is 0° and 2_1 is 360° (Figure S2, SI; Table S2, SI). Comparing the two compounds, we observe that **18** has larger energy differences with ΔΔU_1_ = 3.62 kcal/mol, ΔΔU_2_ = 0.50 kcal/mol and ΔΔU_C_ = 4.0 kcal/mol.

The structural motif that we can extrapolate from the systematic conformational search of the two compounds to resolve the NOE signals can be summarized as follows: (i) when the hydroxamic acid group adheres to *E* conformation, the −OH should approach the α-CH_2_ of the hydroxamic carbonyl; (ii) when the amide adheres to *E* conformation, the α-CH_2_ of the hydroxamic carbonyl should approach the α-CH_2_ of the amide carbonyl in both compounds: **18 (**R = H) and **21 (**R = CH_3_). In addition, from the results of the conformational search for the two compounds, we can see that the relative energy differences (ΔU) of the different rotamers in **18** are significantly higher than for **21**. Nonetheless, since **21** has an additional methyl group on the amide nitrogen which adds steric hindrance to the system, its minimum energy conformation is in a higher absolute energy level (an approach of this is expressed by the force field energy U). Thus, **21** has lower energy barriers between its rotamers, although its minimum energy conformation is situated in a higher energy level, while in a reverse manner, **18** has higher energy barriers between its rotamers, and its minimum energy conformation is situated in a lower energy level. This profiling suggests that it should be energetically easier for **21** to populate the rotamers of higher energy than for **18**.

**Scheme 2:**
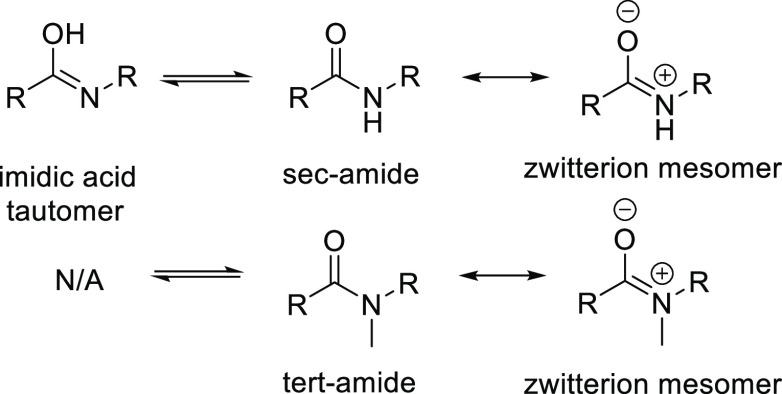
Tautomerization and Resonance Structures of *sec*-Amides and *tert*-Amides

This is further verified by the results of the dihedral coordination scans, where again the relative energy differences are significantly higher in **18**. An examination of the output structures of the coordination scans illustrates that, although more sterically hindered, the dihedral 1_1 of **21** is more free to rotate than that of its counterpart **18** (Figures S10–S13, SI). The secondary amide group in **18** can tautomerize toward the respective imidic acid, whereas the tertiary amide in **21** is not eligible for tautomerization, while both have access to the respective mesomeric zwitterion structure (Scheme 2). As a result, the amide bond in **18** is more rigid than that in **21**, as in the former its character is more strongly shifted toward a double bond relative to the latter. Therefore, despite the steric hindrance, the amide bond in **21** is more prone to rotate than that in **18**, providing an explanation for the energy differences of the coordination scans, as well as for the energy barriers between the rotamers derived from the conformational search.

### Biological Activity

The new hydroxamic acid derivatives **18**–**28** were tested against bloodstream-form *T*. *brucei in vitro*. The results are presented in Table 1, and are expressed as IC_50_ and IC_90_ values. Compounds **19**-**23**, **25** and **28** displayed potent trypanocidal activities with midnanomolar to low micromolar IC_50_ values (0.034–1.65 μM), while having low toxicity against mammalian cells (rat skeletal myoblast L6 cells) (Table 1). In this new series of hydroxamates **18**-**28**, the SAR study revealed the following.

**Table 1:** Activity of Hydroxamic Acid Analogues **18-28** Tested against Cultured Bloodstream-form *Trypanosoma brucei* (pH = 7.4) and Cytotoxicity of the Most Active Compounds against Cultured Rat Skeletal Myoblast L6 Cells (Experimental Details in the SI)^17^

	Activity		Cytotoxicity L6 cells	
Compound	IC_50_ (μM)^a^^b^	IC_90_ (μM)^a^^b^	IC_50_ (μM)^c^	SIs^d^
**18**	25 ± 3	36 ± 2	ND	-
**19**	0.40 ± 0.09	0.98 ± 0.05	35 ± 4	90
**20**^e^	1.23 ± 0.20 (1.53 ± 0.25)	4.88 ± 0.74 (4.26 ± 0.91)	29 ± 2 (25 ± 1)	24 (16)
**21**	1.33 ± 0.11	3.42 ± 0.85	376 ± 7	285
**22**	0.034 ± 0.002	0.057 ± 0.005	32 ± 3	940
**23**	0.053 ± 0.011	0.081 ± 0.003	25 ± 2	470
**24**	>300	>300	ND	-
**25**^e^	1.19 ± 0.28 (0.85 ± 0.09)	1.90 ± 0.10 (1.71 ± 0.21)	223 ± 28 (249 ± 18)	185 (295)
**26**	38 ± 8	ND	ND	-
**27**	>30	ND	ND	-
**28**	1.65 ± 0.13	3.33 ± 0.13	390 ± 6	235

a Concentrations required to inhibit growth of *T*. *brucei* by 50% and 90%, respectively.

b IC_50_ and IC_90_ data are the mean of triplicate experiments ± standard error of the mean (SEM).

c Cytotoxicity was determined by establishing the concentration required to inhibit growth of cultured L6 cells by 50% (IC_50_). Data are the mean of triplicate experiments ± standard error of the mean (SEM).

d Selectivity indices (SIs) were calculated as the ratio of the IC_50_ for L6 cells and *T*. *brucei*.

e Data in brackets refer to the respective hydrochloride. ND: Not determined.

Insertion of an acetamido portion (−CH_2_CONH−) between the spiro adamantane 2,6-DKP scaffold and acetohydroxamate pharmacophoric group (−CH_2_CONHOH) in compound **1a** (IC_50_ = 0.09 μM), leading to compound **18** (IC_50_ = 25 μM), caused a 278-fold loss in potency. The same modification on the cyclooctane- and cycloheptane-based acetohydroxamic acid analogues **4a (**IC_50_ = 0.30 μM) and **5a** (IC_50_ = 1.87 μM), giving the respective compounds **24** and **27**, also led to substantial loss of activity (compare compounds **4a** vs **24** and **5a** vs **27**). However, attachment of the hydrophobic isobutyl substituent to the methylene carbon of the 2,6-DKP ring in the parent compounds **18**, **24** and **27**, resulting in the (*S*)-enantiomers of the corresponding isobutyl-substituted analogues **19**, **25** and **28**, greatly enhanced activity against *T*. *brucei*. Indeed, compounds **19**, **25** and **28** were 62, > 252 and >18 times more potent than the unsubstituted parents **18**, **24** and **27**, respectively, with IC_50_ values in the submicromolar (**19**) or low micromolar (**25**, **28**) range (Table 1). A similar trend was observed when the bulky hydrophobic benzyl substituent was incorporated into the same carbon of the 2,6-DKP ring in the adamantane-based parent **18**. The resulting benzyl substituted analogue **20** [(*S*)-enantiomer] showed a 20-fold increase in potency relative to the unsubstituted parent **18**, with a low micromolar IC_50_ value (Table 1). The large increase in potency for **19**, **20**, **25** and **28** probably reflects the favorable lipophilic and/or stereoelectronic effects exerted by the bulky isobutyl and benzyl substituents in the target binding site. To further address the impact of the different substitution patterns and residues in lipophilicity, we conducted a modeling calculation of drug-like properties and descriptors using the QikProp module of the Schrödinger platform (Table S3, cmp_qikprop.out). The range of the predicted logP for all compounds spans from −1.9 to 0.4, with the lowest values adhering to the *sec*-amides **18**, **24**, **27** and the ring unsubstituted **21**, **26** (Table S3). Additionally, the introduction of a methyl substituent to the nitrogen atom of the acetamido portion (−CH_2_CONH−) in the pharmacophoric 2-acetamidoacetohydroxamic acid functionality (−CH_2_CONHCH_2_CONHOH) of compounds **18**-**20** and **24** appeared to have a favorable effect on the trypanocidal activity and resulted in a significant potency increase for the corresponding *N*-methylated analogues **21**-**23** and **26**; they were recorded as being 19, 12, 23 and >8 times more potent than the nonmethylated counterparts **18**-**20** and **24**, respectively. Compounds **21**-**23** inhibited *T*. *brucei* growth at midnanomolar (**22**, **23**) or low micromolar (**21**) levels. Interestingly, **22** and **23** exhibit the highest predicted values for apparent Caco-2 cell permeability (31 nm/sec and 19 nm/sec, respectively – Table S3), as well as oral absorption percentage (53% and 51%, respectively – Table S3). Thus, even though the models suggest a low level of lipophilicity for **22** and **23** (logP = −0.1 and 0.2, respectively – Table S3), they are predicted to exhibit significantly better permeability and absorption than the other compounds. In addition, these results might be attributed to the favorable spatial arrangement of the methylated side pharmacophoric functionality [−CH_2_CON(CH_3_)CH_2_CONHOH] for interactions with the active site, probably due to the conformational profile described above.

Importantly, the activity results show that the combination of isobutyl- or benzyl-substitution on the 2,6-DKP ring and the concomitant methylation of the side pharmacophoric moiety in the adamantane-based unsubstituted parent compound **18** drastically increased the antitrypanosomal potency and gave the more active compounds **22** and **23** (IC_50_ values: 0.034 and 0.053 μM, respectively). Compounds **22** and **23** appeared to be 735 and 472 times more potent than **18**, respectively. While each of the two modifications separately led to less active compounds (**19**, **20** and **21**), both modifications in concert provided the best antitrypanosomal effect.

It is worth noting that the most active compounds (**19**-**23**, **25** and **28**) were found to have significant selectivity for *T*. *brucei* compared to mammalian cells used (SI = 90–940, Table 1), except for **20** (SI = 24). Among them, the adamantane-based hydroxamates **22** and **23**, which displayed the most potent trypanocidal activity (IC_50_ = 0.034 and 0.053 μM, respectively) proved to be the most parasite-selective compounds (SI = 940 and 470, respectively). Last but not least, QikProp models suggest that our compounds have a property similarity ranging 70–81% compared to marketed drug APIs from a database of 1712 APIs (cmp_qikprop.out), indicating their significant therapeutic potential.

## Conclusion

We have generated a new series of hydroxamic acid derivatives that are based on spiro carbocyclic 2,6-DKP scaffolds. Most of them inhibit the bloodstream-form *T*. *brucei* parasite growth with low micromolar to midnanomolar IC_50_ values. The modification of the previously reported acetohydroxamic acid derivatives **1a**, **4a** and **5a** (Figure 1) by inserting an acetamido portion (−CH_2_CONH−) between the 2,6-DKP ring and the acetohydroxamate group (−CH_2_CONHOH) resulted in compound **18** with marginal activity, and the inactive compounds **24** and **27**. However, compounds **18**, **24** and **27** were successfully transformed into the (*S*)-isobutyl- or (*S*)-benzyl-substituted analogues **19**, **20**, **25** and **28** that displayed submicromolar (**19**) or low micromolar (**20**, **25** and **28**) trypanocidal activities by attaching an isobutyl or benzyl substituent at the vicinal position of the basic nitrogen atom in the 2,6-DKP ring. Conversely, methyl-substitution on the nitrogen atom of the acetamido portion (CH_2_CONH−) in the side pharmacophoric hydroxamate functionality (−CH_2_CONHCH_2_CONHOH) of compounds **18**-**20** and **24** significantly improved trypanocidal potency, as represented by the respective methylated analogues **21**-**23** and **26**. In particular, compounds **22** and **23** [(*S*)-enantiomers] displayed midnanomolar trypanocidal activities, demonstrating that the isobutyl- or benzyl-substitution on the 2,6-DKP ring in conjunction with methylation of the side pharmacophoric moiety in the adamantane-based unsubstituted parent compound **18** was crucial in terms of activity against bloodstream form *T*. *brucei*. Indeed, compounds **22** and **23** exhibited the highest growth inhibitory activity (within the series), with significant selectivity, compared to the mammalian cells. This SAR, taken together with the computational studies conducted on **18** and **21**, demonstrate that the freedom of the acetamidoacetohydroxamic arm to rotate is of paramount importance for achieving high ligand potency.

On the basis of these encouraging findings, compounds **22** and **23** were selected as chemical leads for the development of new hydroxamic acid derivatives with optimized antitrypanosomal potency and outstanding selectivity against the parasite, through appropriate substitutions in both the 2,6-DKP portion and side pharmacophoric moiety.

## Supporting Information Available

Full detailed report of all QikProp models and predicted values (TXT)
Experimental procedures for the synthesis of the target compounds; experimental procedures for the HPLC quantitative determination; experimental procedures for the computational studies; Experimental procedures for the biological evaluation of the compounds; drug-like properties predictions for the tested compounds; copies of NMR spectra of all synthesized compounds (PDF)

## Notes

Safety Statement: In this study, we carried out a series of experiments to validate the hypothesis. Throughout the experiment, we properly followed the laboratory’s safety guidelines and operational protocols. All experimental procedures were carried out in the endorsed laboratory space, with staff wearing adequate personal protective equipment. There were no significant risky behaviors or safety problems during the experiment. After the experiment, we appropriately disposed of all waste and chemical residues.

## Abbreviations

DKP: Diketopiperazine
NMR: Nuclear Magnetic Resonance
HAT: Human African Trypanosomiasis
FAO: Food and Agriculture Organisation
BBB: blood–brain barrier
T.b.: *Trypanosoma brucei*
SAR: Structure–Activity Relationship
TAO: Trypanosome Alternative Oxidase
EDCI: 1-[3-(Dimethylamino)propyl]-3-ethylcarbodiimide
HOBt: 1-Hydroxybenzotriazol
CDI: 1,1′-Carbonyldiimidazol
DIEA: Diethylisopropylamine
Ar: Argon
THF: Tetrahydrofuran
DMF: Dimethylformamide
Et_3_N: Triethylamine
EtOH: Ethanol
NOESY: Nuclear Overhauser Effect Spectroscopy
NOE: Nuclear Overhauser Effect
