# Supplementary material for: New Lipophilic Hydroxamates as Promising Trypanocidal Agents: Design, Synthesis, SAR, and Conformational Behavior Studies
Source: ACS Med Chem Lett. 2024 Jun 6;15(7):1041–8. doi: 10.1021/acsmedchemlett.4c00111 (PMC11247629; doi:10.1021/acsmedchemlett.4c00111)

Supplementary Information

New lipophilic hydroxamates as promising trypanocidal agents: Design, Synthesis, SAR and Conformational Behavior Studies

George Fytas,[*,1] Grigoris Zoidis,[*,1] Antonios Drakopoulos,[2] Martin C. Taylor,[3] John M. Kelly,[3] Alexandra Tsatsaroni,[1] Andrew Tsotinis[1]

[1]*Faculty of Pharmacy, Department of Pharmaceutical Chemistry, University of Athens, Panepistimioupoli-Zografou, GR-15771, Athens, Greece*

[2]*Department of Chemistry and Molecular Biology, University of Gothenburg, Göteborg, SE-412 96, Sweden*

[3]*Department of Pathogen Molecular Biology, London School of Hygiene and Tropical Medicine Keppel Street, London WC1E 7HT, UK*

*To whom correspondence should be addressed: Tel.: +30 210 727 4810; gfytas@pharm.uoa.gr, Tel.: +30 210 727 4809; zoidis@pharm.uoa.gr

List of Contents

I. Experimental procedures for the synthesis of the target compounds **3**
II. Experimental procedures for the HPLC quantitative determination **26**
III. Experimental procedures for the computational studies **27**
IV. Experimental procedures for the biological evaluation of the compounds **33**
V. References **33**
VI. Table S3: Drug-like properties predictions for the tested compounds **34**
VII. Copies of NMR spectra **38**
NMR spectra of **18** (^{1}H, ^{13}C, COSY, HSQC, DEPT) **38**
NMR spectra of **19** (^{1}H, ^{13}C, COSY, HSQC, DEPT) **43**
NMR spectra of **20** (^{1}H, ^{13}C, COSY, HSQC, DEPT) **48**
NMR spectra of **21** (^{1}H, ^{13}C, COSY, HSQC, DEPT) **53**
NMR spectra of **22** (^{1}H, ^{13}C, COSY, HSQC, DEPT) **58**
NMR spectra of **23** (^{1}H, ^{13}C, COSY, HSQC-DEPT) **63**
NMR spectra of **24** (^{1}H, ^{13}C, COSY, HSQC-DEPT) **67**
NMR spectra of **25** (^{1}H, ^{13}C, COSY, HSQC, DEPT) **71**
NMR spectra of **26** (^{1}H, ^{13}C, COSY, HSQC, DEPT) **76**
NMR spectra of **27** (^{1}H, ^{13}C, COSY, DEPT) **81**
NMR spectra of **28** (^{1}H, ^{13}C, COSY, HSQC-DEPT, HMBC) **85**
NMR spectra of **36** (^{1}H, ^{13}C, COSY, HSQC-DEPT, DEPT) **90**
NMR spectra of **37** (^{1}H, ^{13}C, COSY, HSQC, DEPT) **95**
NMR spectra of **38** (^{1}H, ^{13}C, COSY, HSQC, DEPT) **100**
NMR spectra of **39** (^{1}H, ^{13}C, COSY, HSQC, DEPT) **105**
NMR spectra of **40** (^{1}H, ^{13}C, COSY, HSQC-DEPT, DEPT) **110**
NMR spectra of **41** (^{1}H, ^{13}C, COSY, HSQC-DEPT) **115**
NMR spectra of **42** (^{1}H, ^{13}C, COSY, HSQC, DEPT) **119**
NMR spectra of **43** (^{1}H, ^{13}C, COSY, HSQC, DEPT) **124**
NMR spectra of **44** (^{1}H, ^{13}C, COSY, HSQC, DEPT) **129**
NMR spectra of **45** (^{1}H, ^{13}C, COSY, HSQC, DEPT) **134**
NMR spectra of **46** (^{1}H, ^{13}C, COSY, HSQC-DEPT, DEPT) **139**
NMR spectra of **47** (^{1}H, ^{13}C, COSY, HSQC, DEPT) **144**
NMR spectra of **48** (^{1}H, ^{13}C, COSY, HSQC-DEPT, DEPT) **149**
NMR spectra of **49** (^{1}H, ^{13}C, COSY, HSQC-DEPT, DEPT) **154**
NMR spectra of **50** (^{1}H, ^{13}C, COSY, HSQC-DEPT, DEPT) **159**
NMR spectra of **51** (^{1}H, ^{13}C, COSY, HSQC-DEPT, DEPT) **164**
NMR spectra of **52** (^{1}H, ^{13}C, COSY, HSQC-DEPT) **169**
NMR spectra of **53** (^{1}H, ^{13}C, COSY, HSQC, HMBC, DEPT) **173**
NMR spectra of **54** (^{1}H, ^{13}C, COSY, HSQC-DEPT, DEPT) **177**
NMR spectra of **55** (^{1}H, ^{13}C, COSY, HSQC, DEPT) **183**
NMR spectra of **56** (^{1}H, ^{13}C, COSY, HSQC, DEPT) **188**
NMR spectra of **57** (^{1}H, ^{13}C, COSY, HSQC-DEPT, DEPT) **193**
NMR spectra of **58** (^{1}H, ^{13}C, COSY, HSQC-DEPT, DEPT) **198**
NMR spectra of **59** (^{1}H, ^{13}C, COSY, HSQC, DEPT) **203**
NMR spectra of **60** (^{1}H, ^{13}C, COSY, HSQC-DEPT, DEPT) **208**
NMR spectra of **61** (^{1}H, ^{13}C, COSY, HSQC, DEPT) **213**
NMR spectra of **62** (^{1}H, ^{13}C, COSY, HSQC, DEPT) **218**
NMR spectra of **63** (^{1}H, ^{13}C, COSY, HSQC-DEPT) **223**
NMR spectra of **64** (^{1}H, ^{13}C, COSY, HSQC-DEPT, DEPT) **227**
NMR spectra of **65** (^{1}H, ^{13}C, COSY, HSQC, DEPT) **232**
NMR spectra of **66** (^{1}H, ^{13}C, COSY, HSQC, DEPT) **237**
NMR spectra of **67** (^{1}H, ^{13}C, COSY, DEPT) **242**
NMR spectra of **68** (^{1}H, ^{13}C, COSY, HSQC, DEPT) **246**
NOESY NMR of **18** (600.11 MHz, DMSO-d6) **251**
NOESY NMR of **21** (600.11 MHz, DMSO-d6) **252**

Experimental

Chemistry

General. Melting points were determined using a Büchi capillary apparatus and are uncorrected. ^{1}H and ^{13}C NMR spectra were obtained on Bruker MSL 400 (400 MHz ^{1}H), Bruker AVANCE III 600 (600 MHz ^{1}H, 150 MHz ^{13}C) and Bruker AVANCE 200 (50 MHz ^{13}C) spectrometers, using $CDCl_3$ or DMSO-d_6 as solvent. Chemical shifts are reported in δ(ppm) with tetramethylsilane or solvent (DMSO-d_6) as internal standard. Splitting patterns are designated as s, singlet; d, doublet; dd, doublet of doublets; t, triplet; td, triplet of doublets; dt, double of triplets; q, quartet; qd, quartet of doublets; m, multiplet; sept, septuplet; br, broad; v br, very broad; sym, symmetrical. The spectra were recorded at 293 K (20 °C). Carbon multiplicities were established by DEPT experiments. 2D NMR experiments (HMQC and COSY) were performed for the elucidation of the structures of the newly synthesized compounds. Low-resolution mass spectra were recorded on either an API 2000 LC-MS/MS or Thermo-Finnigan AQA model LC-MS system, using positive electrospray ionization mode or Thermo Electron Corporation DSQ mass spectrometer in electron impact (EI). High-resolution mass spectra (HRMS) were determined on a hybrid LTQ-Orbitrap Discovery mass spectrometer under electrospray ionization (ESI) in positive ion mode. Optical rotations were measured on a Perkin Elmer 341 polarimeter at the sodium D line (589). Analytical thin-layer chromatography (TLC) was conducted on precoated Merck silica gel 60 F_{254} plates (layer thickness 0.2 mm) with the spots visualized by iodine vapors and/or UV light. Column chromatography purification was carried out on silica gel 60 (70-230 and 230-400 mesh ASTM). Elemental analyses (C, H, N) were performed by the Department of Microanalysis of NCSR "Democritos" (Greece), and were within ±0.4% of the calculated values. The purity of all tested compounds was determined by elemental analysis. Additional indicative HPLC analysis was implemented for compound **20**. The obtained results correspond to >95% purity (Table S1). Analytical HPLC was performed on a Thermo Finnigan HPLC system (Thermo Finnigan, San Jose, USA) consisting of a SpectraSystem P4000 pump, a SpectraSystem 100 degasser, a SpectraSystem AS3000 autosampler, and a SpectraSystem UV2000 PDA detector, controlled by a SpectraSystem controller. ChromQuest 4.1 software was used for the management of the data. For the HPLC-DAD, a Supelco Analytical Discovery HS C18 (250 mm x 4.6 mm, 5.0 μm) column was used and the injection volume was 10 μL. The mobile phase consisted of H_2O and 1% acetic acid (solvent A) and acetonitrile (solvent B), and solvent gradient of A/B was 95/5 to 0/100. The analyses were performed at r.t. with a constant flow rate of 1 mL/min using a gradient elution of 0-50 min. The commercial reagents were purchased from Alfa Aesar, Sigma-Aldrich and Merck, and were used without further purification. Organic solvents used were in the highest purity, and when necessary, were dried by the standard methods. Yields refer to chromatographically pure materials.

_N_-[3,5-Dioxospiro[piperazine-2,2'-tricyclo[3.3.1.1^{3,7}]dec-4-yl]acetyl]glycine 47

To a stirred solution of carboxylic acid **29**[1] (425 mg, 1.45 mmol) in a dichloromethane-dimethylformamide 3:5 v/v dry mixture (20 mL), were added EDCI.HCl (334 mg, 1.74 mmol), HOBt (235 mg, 1.74 mmol),

glycine benzyl ester tosylate (585 mg, 1.74 mmol) and diisopropylethylamine (DIEA, 675 mg, 5.22 mmol). The mixture was stirred at 28 ° C for 24h under argon, and then poured into water (40 mL) and extracted with AcOEt (4x30 mL). The combined organic extracts were washed with water (40 mL) and brine (40 mL), dried (Na_2SO_4) and evaporated *in vacuo*. The residual viscous oil was chromatographed on silica gel column with AcOEt-*n*-hexane 1:1 v/v, as eluent, to afford the *N*-substituted glycine benzyl ester **36** as a white crystalline solid (450 mg, 67%): An analytical sample was obtained upon dissolution of the product in Et_2O-AcOEt 10:1 v/v and *n*-pentane-mediated precipitation: mp 122-124 °C; ^{1}H NMR (400 MHz, $CDCl_3$) δ 1.54 (d, 2H, *J* = 12.0 Hz, 4′e, 9′e-H), 1.63-1.97 (m, 7H, 1,5′, 6′, 7′, 8′e, 10′e-H), 2.01 (s, 2H, 1′, 3′-H), 2.29 (d, 4H, *J* = 12.0 Hz, 4′a, 8′a, 9′a, 10′a-H), 3.73 (s, 2H, 6-H), 4.07 (d, 2H, *J* ≅ 5.0 Hz, CONHC$H_2$$CO_2$$CH_2$Ph), 4.44 (s, 2H, C$H_2$CONH), 5.17 (s, 2H, CO_2CH_2Ph), 6.35 (~t, 1H, *J* = 4.2 Hz, CON*H*CH_2), 7.28-7.45 (m, 5H, aromatic H); ^{13}C NMR (50 MHz, $CDCl_3$) δ 27.2, 27.4 (5′,7′-C), 32.5 (4′, 9′-C), 32.8 (1′, 3′-C), 33.3 (8′,10′-C), 38.2 (6′-C), 41.6 (CONH*C*$H_2$$CO_2$$CH_2$Ph), 42.1 (*C*$H_2$CONH), 44.6 (6-C), 60.7 (2,2′-C), 67.4 (CO_2*C*H_2Ph), 128.5, 128.7, 128.8 (2, 3, 4, 5, 6- aromatic C), 135.2 (1- aromatic C), 167.4 (*C*$O_2$$CH_2$$C_6H_5$), 169.7 (*C*ONH$CH_2$), 172.6, 174.7 (3, 5-C). Anal. Calcd for $C_{24}H_{29}N_3O_5$: C, 65.59; H, 6.65; N, 9.56; Found: C, 65.72; H, 6.80; N, 9.39.

The benzyl ester derivative **36** (1.11 g, 2.52 mmol) was dissolved in abs EtOH-MeOH 1:1 v/v (63 mL) and 10% Pd on charcoal (133 mg) was added. The mixture was shaken under an atmosphere of 50 psi hydrogen for 3 h at room temperature. The catalyst was then removed by filtration and washed with hot methanol (3x15 mL). The combined filtrates were concentrated to dryness under reduced pressure to afford the title compound **47** as a white crystalline solid (877 mg, >99%). An analytical sample was obtained upon recrystallization of the product from MeOH-Et_2O (decomposed gradually above 175 ° C without distinct mp). ^{1}H NMR (400 MHz, DMSO-d_6) δ 1.42 (d, 2H, *J* = 10.6 Hz, 4′e, 9′e-H), 1.53-1.84 (m, 6H, 5′, 6′, 7′, 8′e, 10′e-H), 1,94 (s, 2H, 1′, 3′-H), 2.15-2.40 (~br s, 4H, 4′a, 8′a, 9′a, 10′a-H), 3.56 (d, 2H, *J* = 5.7 Hz, 6-H), 3.76 (d, 2H, *J* = 5.4 Hz, CONHC$H_2$$CO_2$H), 4.28 (d, 2H, *J* = 5.7 Hz, CH_2CONH), 2.64-6.38 (v br s, 2H, 1-H, CO_2*H*), 8.29 (~t, 1H, *J* = 5.6 Hz, CON*H*CH_2); ^{13}C NMR (50 MHz, DMSO-d_6) δ 26.7 (5′-C), 27.0 (7′-C), 31.9 (1′, 3′-C), 32.0 (4′, 9′-C), 32.8 (8′,10′-C), 37.8 (6′-C), 40.8 (CONH*C*$H_2$$CO_2$H), 41.1 (*C*$H_2$CONH), 44.1 (6-C), 59.5 (2,2′-C), 167.1 (*C*O_2H), 171.2 (*C*ONHCH_2), 172.2, 174.4 (3, 5-C). Anal. Calcd for $C_{17}H_{23}N_3O_5$: C, 58.44; H, 6.64; N, 12.03; Found: C, 58.36; H, 6.72; N, 11.82.

(*S*)-*N*-[6-(2-Methylpropyl)-3,5-dioxospiro[piperazine-2,2'-tricyclo[3.3.1.1^{3,7}]dec-4-yl]acetyl]glycine 48

The benzyl ester precursor **37** was prepared by coupling the carboxylic acid **30**[2] (1.3 g, 3.73 mmol) with glycine benzyl ester following the procedure described for compound **36** (precursor for **47**). The crude product (yellowish viscous oil) was purified by column chromatography on silica gel eluting with AcOEt-*n*-hexane 1:4 to 1:2 v/v to afford **37** as an off-yellow foamy solid, which strongly binds the elution solvents. Removal of the entrapped solvents upon drying at 62-64 °C under high vacuum (10^{-2} mmHg) in an Abderhalden apparatus gave **37** as a glass solid (1.12 g, 61%): ^{1}H NMR (600 MHz, $CDCl_3$) δ 0.96 (d, 3H, *J* =

3.4 Hz, CH(CH_3)CH$_3$), 0.97 (d, 3H, J = 3.2 Hz, CH(CH$_3$)CH_3), 1.18-1.30 (br s, 1H, 1-H), 1.43-1.53 (complex m, 2H, CHHCH(CH$_3$)$_2$, 4′e-H), 1.56 (~d, 1H, J = 12.2 Hz, 9′e-H), 1.64-1.79 (complex m, 5H, 6′, 8′, 10′e-H), 1.84, 1.87 (s+s, 3H, 3′, 5′, 7′-H,), 1.90-1.99 (complex m, 2H, CHHCH(CH$_3$)$_2$), 2.12 (s, 1H, 1′-H), 2.17 (d, 1H, J = 12.2 Hz, 9′a-H), 2.43 (d, 1H, J = 12.3 Hz, 4′a-H), 2.90 (d, 1H, J = 12.8 Hz, 10′a-H), 3.65 (d, 1H, J = 8.9 Hz, 6-H), 4.06 (d, 2H, J = 5.1 Hz, CONHCH_2CO$_2$CH$_2$Ph), 4.27-4.59 (q, AB, 2H, J_{AB} = 15.5 Hz, CH_2CONH), 5.15 (s, 2H, CO$_2$CH_2Ph), 6.44 (t, 1H, J=4.9 Hz, CONHCH$_2$), 7.29-7.37 (complex m, 5H, aromatic H); ^{13}C NMR (50 MHz, CDCl$_3$) δ 21.3, 23.6 (CH(CH$_3$)CH$_3$), 24.7 (CH(CH$_3$)$_2$), 27.2, 27.5 (5′, 7′-C), 31.2 (1′-C), 31.8, 32.8, 33.3, 34.0 (4′, 8′, 9′, 10′-C), 35.2 (3′-C), 38.1 (6′-C), 41.6 (CONHCH$_2$CO$_2$CH2Ph), 41.9 (CH$_2$CH(CH$_3$)$_2$), 42.5 (CH$_2$CONH), 51.8 (6-C), 60.9 (2,2′-C), 67.4 (CO$_2$$CH_2$Ph), 128.5, 128.7, 129.1 (2, 3, 4, 5, 6-aromatic C), 135.1 (1- aromatic C), 167.4 (CO$_2$CH$_2$Ph), 169.7 (CONHCH$_2$), 174.9, 175.9 (3, 5-C); ESI$^+$ MS: m/z 518.2 [M+Na]$^+$, 496.3 [M+H]$^+$.

Benzyl ester **37** (2.1 g, 4.24 mmol) was subjected to catalytic hydrogenolysis (H$_2$/10% Pd-C, 252 mg) in abs EtOH (107 mL) as described for the preparation of **47** from **36**. The resulting white foamy solid strongly binds the hydrogenation solvent. Removal of the entrapped solvent as above in **37** afforded the title compound **48** as a glass solid (1.70 g, 99%): ^{1}H NMR (600 MHz, CDCl$_3$) δ 0.91-0.99 (q like, 6H, J = 2.8, 3.1 Hz, CH(CH_3)CH_3), 1.41-1.52 (m, 2H, CHHCH(CH$_3$)$_2$, 4′e-H), 1.56 (d, 1H, J=11.5 Hz, 9′e-H), 1.62-1.78 (m, 5H, 6′, 8′, 10′e-H), 1.80-1.96 (m, 5H, 3′, 5′, 7′-H, CHHCH(CH$_3$)$_2$), 2.10 (s, 1H, 1′-H), 2.15 (d, 1H, J = 11.8 Hz, 9′a-H), 2.42 (d, 1H, J = 11.9 Hz, 4′a-H), 2.85 (d, 1H, J = 12.2 Hz, 10′a-H), 3.64 (d, 1H, J = 11.0 Hz, 6-H), 4.03 (t like, 2H, J=20.2 Hz, CONHCH_2CO$_2$H), 4.32-4.57 (q, AB, 2H, J_{AB} = 15.8 Hz, CH_2CONH), 4.80-8.0 (v br s, 2H, 1-H, CO$_2$$H$), 7.0 (br s, 1H, CON$HCH_2$); ^{13}C NMR (50 MHz, CDCl$_3$) δ 21.3, 23.6 (CH(CH$_3$)CH$_3$), 24.6 (CH(CH$_3$)$_2$), 27.2, 27.4 (5′, 7′-C), 31.2 (1′-C), 31.8, 32.8, 33.3, 34.0 (4′, 8′, 9′, 10′-C), 35.1 (3′-C), 38.1 (6′-C), 41.7 (CONHCH$_2$CO$_2$H, CH$_2$CH(CH$_3$)$_2$), 42.4 (CH$_2$CONH), 51.8 (6-C), 60.9 (2,2′-C), 168.6 (CO$_2$H), 172.7 (CONHCH$_2$), 175.0, 176.1 (3,5-C). The hydrochloride salt (**48.HCl**) was prepared by treating a clear diethyl ether solution of **48** with saturated solution of HCl in Et$_2$O under ice cooling. The white solid which precipitated was collected by vacuum filtration, triturated with Et$_2$O and dried in vacuo (decomposed gradually above 206 °C without distinct mp). $[\alpha]^{28}_{589}$ = - 15.5 (c, 0.2, DMSO). Anal. Calcd for C$_{21}$H$_{32}$ClN$_3$O$_5$: C, 57.07; H, 7.30; N, 9.51; Found: C, 56.71; H, 7.09; N, 9.75.

(*S*)-*N*-[3,5-Dioxo-6-(phenylmethyl)spiro[piperazine-2,2′-tricyclo[3.3.1.1^{3,7}]dec-4-yl]acetyl]glycine 49

The benzyl ester precursor **38** was prepared by coupling the carboxylic acid **31**[3] (1.0 g, 2.61 mmol) with glycine benzyl ester following the procedure described for compound **36** (precursor for **47**). The crude product (yellowish thick oil) was purified by flash column chromatography (silica gel) eluting first with AcOEt-*n*-hexane 2: 3 and then 1: 1 v/v to afford **38** as a white foamy solid, which strongly binds the elution solvents. Removal of the entrapped solvents as in **37** (precursor for **48**) gave **38** as a glass solid (884 mg, 64%): ^{1}H NMR (600 MHz, CDCl$_3$) δ 1.43-1.53 (complex m, 3H, 1, 4′e, 9′e-H), 1.61-1.86 (complex m, 9H, 3’, 4′a, 5′, 6′, 7’, 8′, 10′e-H), 2.05 (d, 1H, J = 12.2 Hz, 9′a -H), 2.15 (s, 1H, 1′-H), 2.91 (d, 1H, J = 12.8 Hz, 10′a -H),

3.0-3.07 (dd, 1H, *J* = 8.2, 13.9 Hz, C*H*HC_6H_5), 3.35-3.41 (dd, 1H, *J* = 3.95, ~13.9 Hz, CH*H*C_6H_5), 3.88-3.93 (sym m, 1H, 6-H), 4.03-4.11 (sym m, 2H, CONHC*H₂*CO_2CH_2Ph), 4.29-4.56 (q, AB, 2H, *J* = 15.4 Hz, C*H₂*CONH), 5.18 (s, 2H, CO_2C*H₂*Ph), 6.19 (t, 1H, *J* = 4.4 Hz, CON*H*CH_2), 7.21-7.39 (complex m,10, aromatic H); ^{13}C NMR (50 MHz, $CDCl_3$) δ 27.1, 27.2 (5′,7′-C), 30.7 (1′-C), 31.5 (4′-C), 32.7 (9′-C), 33.3 (8′-C), 34.0 (10′-C), 35.0 (3′-C), 38.1 (*C*H_2Ph, 6′-C), 41.6 (CONH*C*$H_2CO_2CH_2$Ph), 42.5 (*C*H_2CONH), 54.3 (6-C), 61.1 (2,2′-C), 67.4 (CO_2***C***H_2Ph), 127.2, 128.5, 128.8, 129.4, 135.2, 136.9 (aromatic C), 167.3 (*C*O_2CH_2Ph), 169.7 (*C*ONHCH_2), 173.8, 174.4 (3,5-C); HRMS (ESI): $[M+Na]^+$ calcd for $C_{31}H_{35}N_3O_5$ 552.2474, found 552.2469.

Benzyl ester **38** (1.1 g, 2.08 mmol) was subjected to catalytic hydrogenolysis (H_2/10% Pd-C, 132 mg) in abs EtOH (52 mL) as described for the preparation of **47** from **36**. The resulting white foamy solid strongly binds the hydrogenation solvent. Removal of the entrapped solvent as in **37** (precursor for **48**) afforded the title compound **49** as a glass solid (910 mg, >99%): ^{1}H NMR (600 MHz, $CDCl_3$) δ 1.37 (d, 2H, *J* = 12.4 Hz, 4′e, 9′e-H), 1.48-1.67 (m, 7H, 4′a, 5′, 6′, 8′, 10′e-H), 1.76 (s, 2H, 3′, 7′- H), 1.89 (d, 1H, *J* = 11.5 Hz, 9′a -H), 2.10 (s, 1H, 1′-H), 2.81 (d, 1H, *J* = 11.6 Hz, 10′a -H), 2.85-2.93 (dd, 1H, *J* = 9.0, 13.3 Hz, C*H*HPh), 3.34 (~d, 1H, *J* = 12.1 Hz, CH*H*Ph), 3.81 (~d, 1H, *J* = 5.0 Hz, 6-H), 3.90-4.07 (qd, 2H, *J* = 4.0 Hz, 18.2 Hz, CONHC*H₂*CO_2H), 4.28-4.54 (q, AB, 2H, J_{AB} = 15.5 Hz, C*H₂*CONH), 4.50-8.50 (v br s, 2H, 1-H, CO_2*H*), 6.88 (s, 1H, CON*H*CH_2), 7.12-7.27 (m, 5H, aromatic H); ^{13}C NMR (50 MHz, $CDCl_3$) δ 27.0 (5′,7′-C), 30.6 (1′-C), 31.4 (4′-C), 32.6 (9′-C), 33.4 (8′-C), 34.1 (10′-C), 34.9 (3′-C), 37.9 (*C*H_2Ph), 38.1 (6′-C), 41.6 (CONH*C*H_2CO_2H), 42.4 (*C*H_2CONH), 54.3 (6-C), 61.2 (2,2′-C), 127.2, 128.8, 129.3 (2, 3, 4, 5, 6 – aromatic C), 137.0 (1– aromatic C), 168.5 (*C*O_2H), 172.8, 173.9, 174.3 (*C*ONHCH_2, 3,5-C). The hydrochloride salt (**49.HCl**) was prepared by treating a clear solution of **49** in AcOEt-Et_2O 1:1 v/v with saturated solution of HCl in Et_2O under ice cooling. The white solid which precipitated was collected by vacuum filtration, triturated with Et_2O and dried in vacuo (decomposed gradually above 118 ° C without distinct mp). $[\boldsymbol{\alpha}]_{\mathbf{589}}^{\mathbf{22}}$ = - 41 (c, 0.2, DMSO). Anal. Calcd for $C_{24}H_{30}ClN_3O_5$: C, 60.56; H, 6.35; N, 8.83; Found: C, 60.90; H, 6.29; N, 8.99.

N-Methyl-*N*-[3,5-dioxospiro[piperazine-2,2'-tricyclo[3.3.1.1^{3,7}]dec-4-yl]acetyl]glycine 50

Carboxylic acid **29**[1] (1.1 g, 3.76 mmol) and 1,1' carbonyldiimidazol (CDI, 732 mg, 4.5 mmol) were stirred together in dry THF (56 mL) at 28 °C for 1 h under argon. Sarcosine benzyl ester hydrochloride (974 mg, 4.5 mmol) and triethylamine (501 mg, 4.95 mmol) were added subsequently, and the mixture was left stirring at 28 °C for 48 h under argon. The solvent was evaporated under reduced pressure, and the resulting residue was partitioned between ethyl acetate (100 mL) and water (80 mL). The aqueous layer was extracted with ethyl acetate (3x50 mL), and the combined organic solution was washed with brine (2x60 mL), dried (Na_2SO_4) and evaporated in vacuo. The residue was chromatographed on silica gel column eluting with AcOEt-*n*-hexane 1:1 followed by 2:1 v/v to afford the *N*-substituted sarcosine benzyl ester **39** as a glass solid (1.19 g, 70%). This compound appears in the ^{1}H and ^{13}C NMR spectra as *E* and *Z* conformers (not assigned), due to the hindered rotation around the C(O)-N(CH_3) amide bond. ^{1}H NMR (400 MHz, $CDCl_3$) δ[(a)] 1.52 (d, 2H, *J* = 12.1 Hz, 4′e, 9′e-H), 1.62-1.94 (m, 7H, 1, 5′, 6′, 7′, 8′e, 10′e-H), 2.03 (s, 2H, 1′, 3′-H), 2.15-

2.41 (~br s, 4H, 4′a, 8′a, 9′a, 10′a-H), [2.95* (s), 3.10 (s), 3H, CH_3], 3.73 (s, 2H, 6-H), [4.11* (s), 4.14 (s), 2H, CON(CH_3)C$H_2$$CO_2$$CH_2$Ph], [4.47* (s), 4.59 (s), 2H, CH_2CON(CH_3)], [5.13 (s), 5.22* (s), 2H, CO_2CH_2Ph], 7.28-7.39 (complex m, 5H, aromatic H); ^{13}C NMR (50 MHz, $CDCl_3$) δ 27.2, 27.4 (5′, 7′-C), 32.4 (4′, 9′-C), 32.7 (1′, 3′-C), 33.3 (8′,10′-C), 35.4*, 35.6 (*C*H_3), 38.1 (6′-C), 40.3*, 40.5 (*C*H_2CON(CH_3)), 44.6 (6-C), 49.7, 51.1* (CON(CH_3)*C*$H_2$$CO_2$$CH_2$Ph), 60.5 (2,2′-C), 67.0, 67.6* (CO_2*C*H_2Ph), 128.3, 128.4, 128.6, 128.7 (2, 3, 4, 5, 6-aromatic C), 135.0*, 135.4 (1-aromatic C), 167.1*, 167.3 (*C*$O_2$$CH_2$Ph), 168.3*, 168.7 (*C*ON(CH_3)CH_2), 172.5, 174.5, 174.6 (3,5-C); HRMS (ESI): [M+H]$^+$ calcd for $C_{25}H_{31}N_3O_5$ 454.2342, found 454.2333.

Benzyl ester **39** (1.07 g, 2.36 mmol) was subjected to catalytic hydrogenolysis (H_2/10% Pd-C, 128 mg) in abs EtOH-AcOEt 1:1 v/v (60 mL) as described for the preparation of **47** from **36**. The resulting white foamy solid strongly binds the aforementioned solvents. Removal of the entrapped solvents as in **37** (precursor for **48**) afforded the title compound **50** as a white semifoamy solid (850 mg, 99%). Compound **50** appears in the ^{1}H and ^{13}C NMR spectra as *E* and *Z* conformers (not assigned), due to the hindered rotation around the C(O)-N(CH_3) amide bond. ^{1}H NMR (400 MHz, $CDCl_3$) δ[(a)] 1.54 (d, 2H, *J* = 11.7 Hz, 4′e, 9′e-H), 1.64-1.92 (m, 6H, 5′, 6′, 7′, 8′e, 10′e-H), 2.03 (s, 2H, 1′, 3′-H), 2.27 (d, 4H, *J* = 11.1 Hz, 4′a, 8′a, 9′a, 10′a-H), [2.94* (s), 3.12 (s), 3H, CH_3], 3.75 (s, 2H, 6-H), 4.10 (s, 2H, CON(CH_3)C$H_2$$CO_2$H), [4.48* (s), 4.59 (s), 2H, CH_2CON(CH_3)], 5.85-6.45 (br s, 2H, 1-H, CO_2*H*); ^{13}C NMR (50 MHz, $CDCl_3$) δ[(a)] 27.2, 27.4 (5′, 7′-C), 32.5 (4′, 9′-C), 32.7 (1′, 3′-C), 33.4 (8′,10′-C), 35.6*, 35.8 (*C*H_3), 38.2 (6′-C), 40.8 (*C*H_2CON(CH_3)), 44.5 (6-C), 50.0, 51.0* (CON(CH_3)*C*$H_2$$CO_2$H), 60.7, 60.9* (2,2′-C), 167.6*, 167.9 (*C*O_2H), 172.3, 172.6, 172.8, 174.3, 174.5 (*C*ON(CH_3)CH_2, 3,5-C); ESI$^+$ MS: m/z 364.2 [M+H]$^+$.

[(a)]The symbol asterisk (*) denotes the weak signal intensity.

(*S*)-*N*-Methyl-*N*-[6-(2-methylpropyl)-3,5-dioxospiro[piperazine-2,2′-tricyclo[3.3.1.1^{3,7}]dec-4-yl] acetyl]glycine 51

The benzyl ester precursor **40** was prepared by coupling the carboxylic acid **30**[2] (1.4 g, 4.0 mmol) with sarcosine benzyl ester following the procedure described for compound **39** (precursor for **50**). The resulting crude product (yellowish thick oil) was purified by column chromatography on silica gel with AcOEt-*n*-hexane 1:2 v/v, as eluent, to afford **40** as a white foamy solid, which strongly binds the elution solvents. Removal of the entrapped solvents as in **37** (precursor for **48**) gave **40** as a glass solid (1.57 g, 76%). This compound appears in the ^{1}H and ^{13}C NMR spectra as *E* and *Z* conformers (not assigned) due to the hindered rotation around the C(O)-N(CH_3) amide bond. ^{1}H NMR (600 MHz, $CDCl_3$) δ[(a)] 0.97 (~br s, 6H, , CH(CH_3)CH_3), 1.13-1.21 (br s, 1H, 1-H), 1.45-1.53 (m, 2H, 4′e-H, C*H*HCH(CH_3)$_2$), 1.56 (d, 1H, *J* = 11.3 Hz, 9′e-H), 1.64-1.80 (complex m, 5H, 6′, 8′, 10′e-H), 1.81-2.04 (complex m, 5H, 3′, 5′, 7′-H, CH*H*C*H*(CH_3)$_2$), 2.17 (s, 1H, 1′-H), 2.18 (d, 1H, *J* = 10.6 Hz, 9′a-H), 2.45 (d, 1H, *J* = 12.1 Hz, 4′a-H), 2.93 (d, 1H, *J* = 12.6 Hz, 10′a-H), [2.96* (s), 3.10 (s), 3H, CON(CH_3)], 3.62-3.70 (br s, 1H, 6-H), [4.05-4.17 (q, AB, 0.44H, *J* = 18.1 Hz), 4.10-4.22 (q, AB, 1.54H, *J* = 17.4 Hz), CON(CH_3)C$H_2$$CO_2$$CH_2$Ph], [4.38 (d, 0.17H, *J* = 15.4 Hz), 4.50 (d, 0.78H, *J* = 15.7 Hz), 4.54 (d, 0.17H, *J* = 15.8 Hz), 4.65 (d, 0.77H, *J* = 15.6 Hz), CH_2CON(CH_3)], [5.14 (s), 5.23* (s), 2H,

CO_2CH_2Ph], 7.29-7.40 (complex m, 5H, aromatic H); ^{13}C NMR (50 MHz, $CDCl_3$) δ[(a)] 21.3, 23.6 (CH(CH_3)CH_3), 24.7 (*C*H(CH_3)$_2$), 27.2, 27.5 (5′, 7′-C), 31.3 (1′-C), 31.8 (9′-C), 32.8 (4′-C), 33.3 (8′-C), 34.0 (10′-C), 35.2 (3′-C, CON(CH_3)), 35.6 (CON(CH_3)), 38.2 (6′-C), 40.8*, 41.0 (CH_2CON(CH_3)), 41.9 (CH_2CH(CH_3)$_2$), 49.7, 51.1* (CON(CH_3)$CH_2CO_2CH_2$Ph), 51.8 (6-C), 60.8 (2,2′-C), 67.0, 67.6* (CO_2CH_2Ph), 128.4, 128.7 (2, 3, 4, 5, 6-aromatic C), 135.1*, 135.4 (1-aromatic C), 167.2*, 167.4 (CO_2CH_2Ph), 168.4*, 168.8 (*C*ON(CH_3)CH_2), 174.8, 175.7 (3,5-C); HRMS (ESI): $[M+H]^+$ calcd for $C_{29}H_{39}N_3O_5$ 510.2968, found 510.2955.

Benzyl ester **40** (3.0 g, 6.08 mmol) was subjected to catalytic hydrogenolysis (H_2/10% Pd-C, 360 mg) in abs EtOH (152 mL) as described for the preparation of **47** from **36**. The resulting white foamy solid strongly binds the hydrogenation solvent. Removal of the entrapped solvent as in **37** (precursor for **48**) gave a white semifoamy solid (2.46 g, >99%). A quantity of this material was dissolved in Et_2O, and the clear solution was concentrated to low volume. The resulting precipitate was isolated by vacuum filtration, washed once with a small volume of Et_2O and dried in vacuo affording the title compound **51** as a white crystalline solid; mp 212-215 ^{o}C (dec). Compound **51** appears in the ^{1}H and ^{13}C NMR spectra as *E* and *Z* conformers (not assigned) due to the hindered rotation around the C(O)-N(CH_3) amide bond. ^{1}H NMR (600 MHz, DMSO-d_6) δ[(a)] 0.92 (d, 6H, *J* = 6.4 Hz, CH(CH_3)CH_3), 1.38 (d, 1H, *J* = 11.5 Hz, 4′e-H), 1.43-1.53 (m, 2H, 9′e-H, C*H*HCH(CH_3)$_2$), 1.58 (d, 1H, *J* = 12.2 Hz, 10′e-H), 1.61-1.82 (complex m, 7H, 5′, 6′, 7′, 8′-H, CH*H*CH(CH_3)$_2$), 1.88 (s, 1H, 3′-H), 1.90-1.98 (m, 1H, C*H*(CH_3)$_2$), 2.08 (s, 1H, 1′-H), 2.15 (d, 1H, *J* = 11.8 Hz, 9′a-H), 2.46 (d, 1H, *J* = 11.7 Hz, 4′a-H), 2.61-2.75 (~br s, 1H, 1-H), [2.79* (s), 3.06 (s), 3H, CON(CH_3)], 2.82 (d, 1H, *J* = 12.4 Hz, 10′a-H), 3.12-3.81 (br s, 1H, CO_2H), 3.47 (br s, 1H, 6-H), [3.92-4.0 (q, AB, 1.26H, J_{AB} = 17.8 Hz), 4.13-4.25 (q, AB, 0.7H, J_{AB} = 18.5 Hz), CON(CH_3)CH_2CO_2H], [4.31-4.43 (q, AB, 0.68H, J_{AB} = 15.9 Hz), 4.41-4.53 (q, AB, 1.32H, J_{AB} = 16.1 Hz), CH_2CON(CH_3)]; ^{13}C NMR (50 MHz, DMSO-d_6) δ[(a)] 21.1, 23.4 (CH(CH_3)CH_3), 24.1 (*C*H(CH_3)$_2$), 26.7, 27.0 (5′, 7′-C), 30.4 (1′-C), 31.3 (9′-C), 32.3 (4′-C), 32.9 (8′-C), 33.4 (10′-C), 33.8 (3′-C), 34.5*, 35.0 (CON(CH_3)), 37.8 (6′-C), 40.6*, 40.7 (CH_2CON(CH_3)), 40.9 (CH_2CH(CH_3)$_2$), 49.1, 50.1* (CON(CH_3)CH_2CO_2H), 51.1 (6-C), 59.7 (2,2′-C), 166.7, 166.8* (CO_2H), 170.6, 170.7* (*C*ON(CH_3)CH_2), 174.5, 175.0 (3,5-C); $[\alpha]^{28}_{589}$ = - 20.5 (c, 0.2, DMSO). Anal. Calcd for $C_{22}H_{33}N_3O_5$: C, 62.98; H, 7.93; N, 10.02; Found: C, 62.63; H, 7.84; N, 9.86.

[(a)]The symbol asterisk (*) denotes the weak signal intensity.

(*S*)-*N*-Methyl-*N*-[3,5-dioxo-6-(phenylmethyl)spiro[piperazine-2,2′-tricyclo[3.3.1.1^{3,7}]dec-4-yl] acetyl]glycine 52

The benzyl ester precursor **41** was prepared by coupling the carboxylic acid **31**[3] (1.47 g, 3.84 mmol) with sarcosine benzyl ester following the procedure described for compound **39** (precursor for **50**). The resulting crude product (yellowish thick oil) was purified by flash column chromatography on silica gel eluting with AcOEt-*n*-hexane 1:2 v/v to afford **41** as a white foamy solid, which strongly binds the elution solvents. Removal of the entrapped solvents as in **37** (precursor for **48**) gave **41** as a glass solid (1.5 g, 72%). This compound appears in the ^{1}H and ^{13}C NMR spectra as *E* and *Z* conformers (not assigned) due to

the hindered rotation around the C(O)-N(CH_3) amide bond. ^{1}H NMR (600 MHz, $CDCl_3$) δ 1.44-1.51 (m, 2H, 4′e, 9′e-H), 1.52-1.58 (br d, 1H, J = 6.7 Hz, 1-H), 1.62-1.81 (complex m, 7H, 5′, 6′, 8′, 9′a, 10′e-H), 1.87 (s, 2H, 3′, 7′-H), 2.08 (d, 1H, J = 12.8 Hz, 4′a -H), 2.25 (s, 1H, 1′-H), 2.96 (d, 1H, J = 12.8 Hz, 10′a -H), 2.99-3.06 (m, 1H, C*H*HPh), [3.01 (s), 3.15 (s), 3H, CH_3], 3.43-3.49 (dd, 1H, J = 3.9, 13.9 Hz, CH*H*Ph), 3.90-3.97 (br s, 1H, 6-H), [4.10-4.21 (q, AB, 0.4H, J_{AB} = 18.3 Hz), 4.13-4.28 (q, AB, 1.58H, J_{AB} = 17.5 Hz), CON(CH_3)C$H_2$$CO_2$$CH_2$Ph], [4.44-4.60 (q, AB, 0.4H, J_{AB} = 15.4 Hz), 4.55-4.69 (q, AB, 1.58H, J_{AB} = 15.7 Hz), CH_2CON(CH_3)], [5.18 (s), 5.24-5.31 (q, AB, J_{AB} = 12.4 Hz), 2H, CO_2CH_2Ph], 7.25-7.44 (complex m, 10H, aromatic H); ^{13}C NMR (150 MHz, $CDCl_3$) δ[(a)] 27.1, 27.2 (5′,7′-C), 30.6 (1′-C), 31.4 (9′-C), 32.6 (4′-C), 33.4 (8′-C), 34.0 (10′-C), 35.1 (3′-C), 35.5*, 35.6 (*C*H_3), 38.1, 38.2 (*C*H_2Ph, 6′-C), 40.6*, 40.8 (*C*H_2CON(CH_3)), 49.7, 51.1* (CON(CH_3)*C*$H_2$$CO_2$$CH_2$Ph), 54.3 (6-C), 61.0 (2,2′-C), 67.0, 67.6* (CO_2*C*H_2Ph), 127.0, 128.4, 128.6, 128.7, 128.8, 129.3, 135.1, 135.5, 137.3 (aromatic C), 167.1*, 167.3 (*C*$O_2$$CH_2$Ph), 168.4*, 168.8 (*C*ON(CH_3)CH_2;), 173.7, 174.4 (3,5-C); HRMS (ESI): $[M+H]^+$ calcd for $C_{32}H_{37}N_3O_5$ 544.2811, found 544.2797.

Benzyl ester **41** (2.75 g, 5.06 mmol) was subjected to catalytic hydrogenolysis (H_2/10% Pd-C, 330 mg) in abs EtOH (127 mL) as described for the preparation of **47** from **36**. The resulting white foamy solid strongly binds the hydrogenation solvent. Removal of the entrapped solvent as in **37** (precursor for **48**) gave a white semifoamy solid (2.28 g, >99%). A quantity of this material was dissolved in a small volume of Et_2O. Addition of *n*-pentane to the clear solution precipitated the product as a white gum, which solidified upon cooling at 0-5 °C for a few hours. The solid material was filtered under vacuum, washed once with a small amount of a cold mixture of Et_2O and *n*-pentane 1:1 v/v, and dried in vacuo affording the title compound **52** as a white crystalline solid; mp 103-106 °C (dec). Compound **52** appears in the ^{1}H and ^{13}C NMR spectra as *E* and *Z* conformers (not assigned) due to the hindered rotation around the C(O)-N(CH_3) amide bond. ^{1}H NMR (600 MHz, $CDCl_3$) δ[(a)] 1.40-1.48 (m, 2H, 4′e, 9′e-H), 1.58-1.77 (complex m, 7H, 5′, 6′, 8′, 9′a, 10′e-H), 1.83 (s, 2H, 3′,7′-H), 2.02 (d, 1H, J = 12.7 Hz, 4′a -H), 2.19 (s, 1H, 1′-H), 2.90 (d, 1H, J = 12.8 Hz, 10′a -H), [2.99* (s), 3.14 (s), 3H, CH_3], 2.93-3.03 (m, 1H, C*H*HPh), 3.38-3.44 (dd, 1H, J = 3.9, 13.9 Hz, CH*H*Ph), 3.60-6.90 (v br s, 2H, 1-H, CO_2*H*), 3.86-3.92 (q, 1H, J = 4.0 Hz, 6-H), [4.03-4.27 (q, AB, J_{AB} = 17.6 Hz), 4.11 (s), 2H, CON(CH_3)C$H_2$$CO_2$H], [4.46 (d, 0.21H, J = 15.5 Hz), 4.54 (d, 1H, J = 15.8 Hz), 4.64 (d, 0.8H, J=15.8) CH_2CON(CH_3)], 7.21-7.34 (complex m, 5H, aromatic H); [13]C NMR (150 MHz, $CDCl_3$) δ[(a)] 27.1, 27.2 (5′,7′-C), 30.6 (1′-C), 31.5 (9′-C), 32.6 (4′-C), 33.4 (8′-C), 34.1 (10′-C), 35.1 (3′-C), 35.6*, 35.8 (*C*H_3), 38.1, 38.2 (*C*H_2Ph, 6′-C), 40.8*, 40.9 (*C*H_2CON(CH_3)), 49.9, 50.9* (CON(CH_3)*C*$H_2$$CO_2$H), 54.4 (6-C), 61.1 (2,2′-C), 127.1, 128.8, 129.4 (2, 3, 4, 5, 6-aromatic C), 137.3 (1-aromatic C), 167.3*, 167.9 (*C*O_2H), 172.4, 174.1* (*C*ON(CH_3)CH_2), 173.9, 174.5 (3,5-C);); $[\boldsymbol{\alpha}]^{\mathbf{24}}_{\mathbf{589}}$ = - 45.5 (c, 0.2, $CHCl_3$); ESI^+ MS: m/z 476.2 $[M+Na]^+$, 454.2 $[M+H]^+$. Anal. Calcd for $C_{25}H_{31}N_3O_5$: C, 66.20; H, 6.89; N, 9.27; Found: C, 66.45; H, 6.63; N, 8.97.

[(a)]The symbol asterisk (*) denotes the weak signal intensity.

***N*-[[3,5-Dioxo-1,4-diazaspiro[5.7]tridec-4-yl]acetyl]glycine 53**

The benzyl ester precursor **42** was prepared by coupling the carboxylic acid **32**[1] (805 mg, 3.0 mmol) with glycine benzyl ester following the procedure described for compound **36** (precursor for **47**). The crude product (yellowish viscous oil) was purified by column chromatography on silica gel eluting first with AcOEt-*n*-hexane 2:1 v/v and then AcOEt to afford **42** as a white crystalline solid (1.20 g, 96%). A specimen was recrystallized from a mixture of AcOEt and Et_2O 1:20 v/v to give an analytical sample which had mp 93-95 ^oC. ^1H NMR (400 MHz, $CDCl_3$) δ 1.38-1.79 (complex m, 12H, 7, 8, 9, 10, 11, 12, 13,-H), 1.88-2.05 (br s, 1H, 1-H), 2.08-2.20 (q, 2H, $J \cong 9.0$ Hz, 7, 13-H), 3.74 (s, 2H, 2-H), 4.06 (d, 2H, $J = 5.2$ Hz, CONHC$H_2$$CO_2CH_2$Ph), 4.45 (s, 2H, C$H_2$CONH), 5.16 (s, 2H, CO_2CH_2Ph), 6.43 (d, 1H, $J = 3.6$ Hz, CON*H*CH_2), 7.29-7.40 (m, 5H, aromatic H); ^{13}C NMR (50 MHz, $CDCl_3$) δ 21.4 (9,11-C), 24.9 (10-C), 28.1 (8,12-C), 31.1 (7,13-C), 41.2 (*C*H_2CONH), 41.6 (CONH*C*$H_2CO_2CH_2$Ph), 45.3 (2-C), 60.1 (6-C), 67.4 (CO_2*C*H_2Ph), 128.5, 128.7, 128.8 (2, 3, 4, 5, 6-aromatic C), 135.2 (1- aromatic C), 167.2 (*C*O_2CH_2Ph), 169.7 (*C*ONHCH_2), 171.6, 176.5 (3,5-C). Anal. Calcd for $C_{22}H_{29}N_3O_5$: C, 63.59; H, 7.04; N, 10.11; Found: C, 63.88; H, 7.15; N, 10.24.

Benzyl ester **42** (1.14 g, 2.74 mmol) was subjected to catalytic hydrogenolysis (H_2/10% Pd-C, 137 mg) in abs EtOH (70 mL) as described for the preparation of **47** from **36**, to give the title compound **53** as a white crystalline solid (888 mg, >99%): mp 169-172 °C (dec) (EtOH-Et_2O); ^1H NMR (400 MHz, DMSO-d_6) δ 1.32-1.82 (m, 12H, 7, 8, 9, 10, 11, 12, 13-H), 1.88-2.05 (m, 2H, 7,13-H), 3.56 (s, 2H, 2-H), 3.76 (d, 2H, $J = 5.6$ Hz, CONHC$H_2$$CO_2$H), 4.25 (s, 2H, C$H_2$CONH), 3.40-5.60 (v br s, 2H, CO_2*H*, 1-H), 8.30 (t, 1H, $J = 5.8$ Hz, CON*H*CH_2); ^{13}C NMR (50 MHz, DMSO-d_6) δ 21.0 (9,11-C), 24.5 (10-C), 27.7 (8,12-C), 30.3 (7,13-C), 40.4 (*C*H_2CONH), 40.6 (CONH*C*H_2CO_2H), 44.8 (2-C), 59.0 (6-C), 166.9 (*C*O_2H), 171.1 (*C*ONHCH_2), 171.5, 176.2 (3,5-C). Anal. Calcd for $C_{15}H_{23}N_3O_5$: C, 55.37; H, 7.13; N, 12.92; Found: C, 55.16; H, 7.22; N, 13.14.

(*S*)-*N*-[[2-(2-Methylpropyl)-3,5-dioxo-1,4-diazaspiro[5.7]tridec-4-yl]acetyl]glycine 54

The benzyl ester precursor **43** was prepared by coupling the carboxylic acid **33**[2] (760 mg, 2.34 mmol) with glycine benzyl ester following the procedure described for compound **36** (precursor for **47**). The crude product (yellowish viscous oil) was purified by column chromatography on silica gel with AcOEt-*n*-hexane 1:1 v/v, as eluent, to afford **43** as a slightly off-yellow glass solid. This material gave a white cotton like solid upon dissolving in Et_2O and subsequent n-pentane-mediated precipitation (720 mg, 65%): mp 110-112 ^oC; ^1H NMR (400 MHz, $CDCl_3$) δ 0.93 (d, 3H, $J = 6.0$ Hz, CH(CH_3)CH_3), 0.96 (d, 3H, $J = 6.4$ Hz, CH(CH_3)CH_3), 1.07-1.36 (v br s, 1H, 1-H), 1.38-2.07 (complex m, 16H, 7, 8, 9, 10, 11, 12, 13-H, CH_2C*H*(CH_3)$_2$), 2.18-2.38 (qd, 1H, $J = 1.6$, 9.1 Hz, 13-H), 3.59-3.75 (dd, 1H, $J = 2.0$, 3.2 Hz, 2-H), 4.07 (d, 2H, $J = 5.2$ Hz, CONHC$H_2$$CO_2CH_2$Ph), 4.32-4.55 (q, AB, 2H, $J_{AB} \cong 15.4$ Hz, CH_2CONH), 5.17 (s, 2H, CO_2CH_2Ph), 6.28 (d, 1H, $J = 4.0$ Hz, CON*H*CH_2), 7.29-7.48 (m, 5H, aromatic H); ^{13}C NMR (50 MHz, $CDCl_3$) δ 20.9 (9-C), 21.3 (CH(*C*H_3)CH_3), 21.5 (11-C), 23.5 (*C*H(CH_3)*C*H_3), 24.6 (10-C), 27.4 (8-C), 28.5, 28.7 (7,12-C), 34.6 (13-C), 40.6 (*C*H_2CH(CH_3)$_2$), 41.6, 41.8 (*C*H_2CONH*C*$H_2CO_2CH_2$Ph), 51.9 (2-C), 60.5 (6-C), 67.4 (CO_2*C*H_2Ph), 128.5, 128.7, 128.8 (2, 3, 4, 5, 6 – aromatic C), 135.2 (1- aromatic C), 167.2 (*C*O_2CH_2Ph), 169.7 (*C*ONHCH_2), 174.3, 177.1 (3,5-C); $[\boldsymbol{\alpha}]^{\mathbf{28}}_{\mathbf{589}}$ = - 19 (c, 0.2, $CHCl_3$). Anal. Calcd for $C_{26}H_{37}N_3O_5$: C, 66.22; H, 7.91; N, 8.91; Found: C, 65.91; H, 8.10; N, 8.63.

Benzyl ester **43** (610 mg, 1.29 mmol) was subjected to catalytic hydrogenolysis (H_2/10% Pd-C, 73 mg) in abs EtOH (32 mL) as described for the preparation of **47** from **36**. The resulting white foamy solid strongly binds the hydrogenation solvent. Removal of the entrapped solvent as in **37** (precursor for **48**) gave the title compound **54** as a glass solid (490 mg, >99%): ^{1}H NMR (600 MHz, $CDCl_3$) δ 0.91 (d, 3H, *J* = 5.7 Hz, CH(C*H*$_3$)CH$_3$), 0.94 (d, 3H, *J* = 5.8 Hz, CH(CH$_3$)C*H*$_3$), 1.42 (t, 1H, *J* = 9.5 Hz, 1-H), 1.45-1.82 (complex m, 12H, 7, 9, 10, 11, 12, 13-H, C*H*HCH(CH$_3$)$_2$), 1.85-1.96 (m, 4H, 8-H, CH*H*C*H*(CH$_3$)$_2$), 2.17-2.26 (q, 1H, *J* = 9.7 Hz, 13-H), 3.58-3.67 (dd, 1H, *J* = 2.3, 9.7 Hz, 2-H), 4.0 (d, 2H, *J* = 2.9 Hz, CONHC*H*$_2$CO$_2$H), 4.38-4.50 (q, AB, 2H, *J* = 16.1 Hz, C*H*$_2$CONH), 6.0-6.40 (br s, 1H, CO$_2$*H*), 7.04 (s, 1H, CON*H*CH$_2$); ^{13}C NMR (50 MHz, $CDCl_3$) δ 20.9 (9-C), 21.2 (CH(*C*H$_3$)CH$_3$), 21.5 (11-C), 23.5 (CH(CH$_3$)*C*H$_3$), 24.5 (C*H*(CH$_3$)$_2$, 10-C), 27.4 (8-C), 28.4 (12-C), 28.7 (7-C), 34.3 (13-C), 40.3 (*C*H$_2$CH(CH$_3$)$_2$), 41.7 (*C*H$_2$CONH*C*H$_2$CO$_2$H), 51.8 (2-C), 60.6 (6-C), 168.4 (*C*O$_2$H), 172.8 (*C*ONHCH$_2$), 174.4, 177.1 (3,5-C). The hydrochloride salt (**54.HCl**) was prepared as described for **48.HCl**, and obtained as a white crystalline solid (decomposed gradually above 200 °C without distinct mp); $[\alpha]^{28}_{589}$ = - 13.5 (c, 0.2, DMSO). Anal. Calcd for $C_{19}H_{32}ClN_3O_5$: C, 54.60; H, 7.72; N, 10.05; Found: C, 54.92; H, 7.58; N, 9.82.

***N*-Methyl-*N*-[[3,5-dioxo-1,4-diazaspiro[5.7]tridec-4-yl]acetyl]glycine 55**

The benzyl ester precursor **44** was prepared by coupling the carboxylic acid **32**[1] (1.1 g, 4.1 mmol) with sarcosine benzyl ester in a tetrahydrofuran – dimethylformamide 1:1 v/v dry mixture (60 mL) following the procedure described for compound **39** (precursor for **50**). The crude product (yellowish viscous oil) was purified by column chromatography on silica gel eluting with AcOEt-*n*-hexane 2:1 v/v followed by 3:1 v/v to afford **44** as an off-white solid (1.07 g, 61%). An analytical sample was obtained as white crystals upon *n*-pentane-mediated precipitation of the product dissolved in Et_2O-AcOEt 100:1 v/v; mp: 89-91 °C. This compound appears in the ^{1}H and ^{13}C NMR spectra as *E* and *Z* conformers (not assigned), due to the hindered rotation around the C(O)-N(CH$_3$) amide bond. ^{1}H NMR (400 MHz, $CDCl_3$) δ[(a)] 1.40-1.85 (complex m, 13H, 1, 7, 8, 9, 10, 11, 12, 13-H), 2.07-2.23 (q, 2H, *J* = 9.2 Hz, 7, 13-H), [2.96*(s), 3.11 (s), 3H, C*H*$_3$], 3.75 (s, 2H, 2-H), [4.11*(s), 4.15 (s), 2H, CON(CH$_3$)C*H*$_2$CO$_2$CH$_2$Ph], [4.48*(s), 4.60 (s), 2H, C*H*$_2$CON(CH$_3$)], [5.14 (s), 5.23* (s), 2H, CO$_2$C*H*$_2$Ph], 7.28-7.40 (m, 5H, aromatic H); ^{13}C NMR (50 MHz, $CDCl_3$) δ[(a)] 21.4 (9,11-C), 24.8 (10-C), 28.1 (8,12-C), 31.1 (7,13-C), 35.5*, 35.6 (*C*H$_3$), 39.6*, 39.8 (*C*H$_2$CON(CH$_3$)), 45.3 (2-C), 49.7, 51.1* (CON(CH$_3$)*C*H$_2$CO$_2$CH$_2$Ph), 60.0 (6-C), 67.0, 67.6* (CO$_2$*C*H$_2$Ph), 128.4, 128.5, 128.6, 128.7, 128.8 (2, 3, 4, 5, 6-aromatic C), 135.4 (1-aromatic C), 166.9*, 167.1 (*C*O$_2$CH$_2$Ph), 168.3*, 168.7 (*C*ON(CH$_3$)CH$_2$), 171.5, 176.5 (3,5-C). Anal. Calcd for $C_{23}H_{31}N_3O_5$: C, 64.31; H, 7.28; N, 9.78; Found: C, 64.22; H, 7.33; N, 9.61.

Benzyl ester **44** (1.01 g, 2.35 mmol) was subjected to catalytic hydrogenolysis (H_2/10% Pd-C, 121 mg) in abs EtOH (60 mL) as described for the preparation of **47** from **36** to give a white foamy solid which strongly binds the aforementioned solvent. Removal of the entrapped solvent as in **37** (precursor for **48**) gave the title compound **55** as a white semifoamy solid (793 mg, >99%). Compound **55** appears in the ^{1}H and ^{13}C NMR spectra as *E* and *Z* conformers (not assigned), due to the hindered rotation around the C(O)-N(CH$_3$)

amide bond. ^{1}H NMR (400 MHz, $CDCl_3$) δ[a] 1.35-1.90 (complex m, 12H, 7, 8, 9, 10, 11, 12, 13-H), 2.04-2.28 (~br s, 2H, 7,13-H), [2.91*(s), 3.10 (s), 3H, C*H₃*], 3.79 (s, 2H, 2-H), 4.04 (s, 2H, CON(CH_3)$CH_2$$CO_2$H), [4.46*(s), 4.57 (s), 2H, CH_2CON(CH_3)], 6.67-7.18 (br s, 2H, CO_2H, 1-H); ^{13}C NMR (50 MHz, $CDCl_3$) δ[a] 21.5 (9,11-C), 24.9 (10-C), 28.1 (8,12-C), 30.6, 30.7 (7,13-C), 35.5*, 35.8 (*C*H_3), 40.1 (*C*H_2CON(CH_3)), 44.5*, 44.7 (2-C), 50.2, 51.4* (CON(CH_3)*C*$H_2$$CO_2$H), 60.6, 60.8* (6-C), 167.4*, 167.5 (*C*O_2H), 170.3, 170.7, 172.3, 175.0, 175.5 (*C*ON(CH_3)CH_2, 3,5-C); ESI$^+$ MS: m/z 340.2 $[M+H]^+$.

[a]The symbol asterisk (*) denotes the weak signal intensity.

_N_-[[3,5-dioxo-1,4-diazaspiro[5.6]dodec-4-yl]acetyl]glycine 56

The benzyl ester precursor **45** was prepared by coupling the carboxylic acid **34**[3] (1.17 g, 4.6 mmol) with glycine benzyl ester following the procedure described for compound **36** (precursor for **47**). The crude product (viscous oil) was purified by column chromatography on silica gel eluting first with AcOEt-*n*-hexane 2:1 v/v and then AcOEt to afford **45** as an off-white solid (1.44 g, 78%). A sample for analysis was obtained as white crystals upon recrystallisation of the product from Et_2O-*n*-pentane; mp 75-77 ^{o}C. ^{1}H NMR (400 MHz, $CDCl_3$) δ 1.49-1.76 (complex m, 10H, 7, 8, 9, 10, 11, 12-H), 1.93 (s, 1H, 1-H), 2.0-2.13 (m, 2H, 7,12-H), 3.72 (s, 2H, 2-H), 4.05 (d, 2H, *J* = 5.1 Hz, CONHC*H₂*$CO_2$$CH_2$Ph), 4.45 (s, 2H, C*H₂*CONH), 5.15 (s, 2H, CO_2C*H₂*Ph), 6.49 (t, 1H, *J* = 4.9 Hz, CON*H*CH_2), 7.27-7.39 (m, 5H, aromatic H); ^{13}C NMR (50 MHz, $CDCl_3$) δ 22.2 (8,11-C), 29.8 (9,10-C), 35.8 (7,12-C), 41.3 (*C*H_2CONH), 41.6 (CONH*C*$H_2$$CO_2$$CH_2$Ph), 45.2 (2-C), 60.7 (6-C), 67.4 (CO_2*C*H_2Ph), 128.4, 128.6, 128.7 (2, 3, 4, 5, 6 – aromatic C), 135.2 (1-aromatic C), 167.2 (*C*$O_2$$CH_2$Ph), 169.7 (*C*ONH$CH_2$), 171.6, 177.1 (3,5-C). Anal. Calcd for $C_{21}H_{27}N_3O_5$: C, 62.83; H, 6.78; N, 10.47; Found: C, 63.05; H, 6.81; N, 10.23.

Benzyl ester **45** (1.0 g, 2.49 mmol) was subjected to catalytic hydrogenolysis (H_2/10% Pd-C, 120 mg) in abs EtOH (63 mL) as described for the preparation of **47** from **36**. The obtained white foamy solid was triturated with Et_2O to give the title compound **56** as a white crystalline solid (766 mg, >99%). Recrystallisation from diethyl ether containing 1% EtOH (v/v) furnished an analytical specimen; mp 147-149 ^{o}C (dec). ^{1}H NMR (400 MHz, DMSO-*d₆*) δ 1.42-1.72 (complex m, 10H, 7, 8, 9, 10, 11, 12-H), 1.84-1.96 (q, 2H, *J* ≅ 9.0 Hz, 7,12-H), 3.55 (s, 2H, 2-H), 3.76 (d, 2H, *J* = 5.7 Hz, CONHC*H₂*CO_2H), 4.25 (s, 2H, C*H₂*CONH), 8.29 (t, 1H, *J* = 5.6 Hz, CON*H*CH_2), 5.25-14.6 (v br s, 2H, CO_2H, 1-H); ^{13}C NMR (50 MHz, DMSO-*d₆*) δ 21.8 (8,11-C), 29.4 (9,10-C), 35.1 (7,12-C), 40.5 (*C*H_2CONH), 40.6 (CONH*C*$H_2$$CO_2$H), 44.7 (2-C), 59.7 (6-C), 166.8 (*C*O_2H), 171.1 (*C*ONHCH_2), 171.5, 176.8 (3,5-C). Anal. Calcd for $C_{14}H_{21}N_3O_5$: C, 54.01; H, 6.80; N, 13.50; Found: C, 53.79; H, 6.72; N, 13.29.

(_S_)-_N_-[[2-(2-Methylpropyl)-3,5-dioxo-1,4-diazaspiro[5.6]dodec-4-yl]acetyl]glycine 57

The benzyl ester precursor **46** was prepared by coupling the carboxylic acid **35**[2] (1.18 g, 3.8 mmol) with glycine benzyl ester following the procedure described for compound **36** (precursor for **47**). The crude product (yellowish viscous oil) was purified by column chromatography on silica gel with AcOEt-*n*-hexane

1:1 v/v, as eluent, to afford **46** as a slightly off-yellow solid (1.36 mg, 78%). An analytical sample was obtained as a white cotton like solid upon dissolution of the product in Et_2O-AcOEt 10:1 v/v, and subsequent *n*-pentane-mediated precipitation; mp 124-126 ^oC. ^1H NMR (400 MHz, $CDCl_3$) δ 0.93 (d, 3H, *J* = 6.4 Hz, CH(C*H₃*)CH₃), 0.96 (d, 3H, *J* = 6.4 Hz, CH(CH₃)C*H₃*), 1.37-2.07 (complex m, 15H, C*H₂*C*H*(CH₃)₂, 1, 7, 8, 9, 10, 11, 12-H), 2.22-2.36 (m, 1H, 12-H), 3.58-3.68 (dd, 1H, *J* = 1.6, 3.2, 9.6 Hz, 2-H), 4.07 (d, 2H, *J* = 4.8Hz, CONHC*H₂*CO₂CH₂Ph), 4.38-4.51 (q, AB, 2H, J_{AB} = 15.6 Hz, C*H₂*CONH), 5.17 (s, 2H, CO₂C*H₂*Ph), 6.31 (~t, 1H, *J* ≅ 4.2 Hz, CON*H*CH₂), 7.30-7.40 (m, 5H, aromatic H); ^{13}C NMR (50 MHz, $CDCl_3$) δ 21.2 (CH(*C*H₃)CH₃), 21.8 (8-C), 22.8 (11-C), 23.5 (CH(CH₃)*C*H₃), 24.5 (*C*H(CH₃)₂), 29.4, 29.7 (9,10-C), 33.6 (7-C), 38.8 (12-C), 40.3 (*C*H₂CH(CH₃)₂), 41.6, 41.8 (*C*H₂CONH*C*H₂CO₂CH₂Ph), 51.8 (2-C), 61.2 (6-C), 67.4 (CO₂*C*H₂Ph), 128.5, 128.7, 128.8 (2, 3, 4, 5, 6– aromatic C), 135.2 (1-aromatic C), 167.2 (*C*O₂CH₂Ph), 169.7 (*C*ONHCH₂), 174.2, 177.5 (3,5-C); $[\alpha]^{25}_{589}$= - 12.5 (c, 0.2, $CHCl_3$). Anal. Calcd for $C_{25}H_{35}N_3O_5$: C, 65.62; H, 7.71; N, 9.18; Found: C, 66.01; H, 7.92; N, 9.40.

Benzyl ester **46** (1.5 g, 3.28 mmol) was subjected to catalytic hydrogenolysis (H_2/10% Pd-C, 180 mg) in abs EtOH (82 mL) as described for the preparation of **47** from **36**. The obtained white foamy solid strongly binds the aforementioned solvent. Removal of the entrapped solvent as in **37** (precursor for **48**) afforded the title compound **57** as a glass solid (1.19 g, 99%): ^1H NMR (400 MHz, $CDCl_3$) δ 0.92 (d, 6H, *J* = 12.6 Hz, CH(C*H₃*)C*H₃*), 1.30-2.08 (complex m, 14H, C*H₂*C*H*(CH₃)₂, 7, 8, 9, 10, 11, 12-H), 2.18-2.33 (m, 1H, 12-H), 3.63 (d, 1H, *J* = 8.5 Hz, 2-H), 4.0 (s, 2H, CONHC*H₂*CO₂H), 4.45 (s, 2H, C*H₂*CONH), 5.95-6.85 (br s, 2H, CO₂*H*, 1-H), 7.09 (s, 1H, CON*H*CH₂); ^{13}C NMR (50 MHz, $CDCl_3$) δ 21.3 (CH(*C*H₃)CH₃), 21.8 (8-C), 22.7 (11-C), 23.5 (CH(CH₃)*C*H₃), 24.6 (*C*H(CH₃)₂), 29.4, 29.7 (9,10-C), 33.6 (7-C), 38.5 (12-C), 40.1 (*C*H₂CH(CH₃)₂), 41.7, 41.8 (*C*H₂CONH*C*H₂CO₂H), 51.8 (2-C), 61.4 (6-C), 168.4 (*C*O₂H), 172.8 (*C*ONHCH₂), 174.2, 177.5 (3,5-C). The hydrochloride salt (**57.HCl**) was prepared as described for **48.HCl**, and obtained as a white solid which had mp 212-215°C (dec); $[\alpha]^{25}_{589}$ = - 7 (c, 0.2, DMSO). Anal. Calcd for $C_{18}H_{30}ClN_3O_5$: C, 53.53; H, 7.49; N, 10.40; Found: C, 53.20; H, 7.56; N, 10.58.

N-Hydroxy-2-[[3,5-dioxospiro[piperazine-2,2'-tricyclo[3.3.1.1^{3,7}]dec-4-yl]acetyl]amino]aceta-mide 18

EDCI.HCl (441 mg, 2.3 mmol), HOBt (311 mg, 2.3 mmol), *O*-benzylhydroxylamine hydrochloride (367 mg, 2.3 mmol) and DIEA (892 mg, 6.9 mmol) were added successively to a stirred solution of the *N*-substituted glycine derivative **47** (660 mg, 1.89 mmol) in a dry mixture of dichloromethane - dimethylformamide 3:5 v/v (28 mL). After 24h of stirring at 28 °C under argon, the mixture was poured into water (40 mL) and extracted with AcOEt (4x40 mL). The combined ethyl acetate solution was washed once with water (50 mL) and once with brine (50 mL), dried (Na_2SO_4) and concentrated to dryness *in vacuo*. The remaining off-yellow viscous oil was chromatographed on silica gel column eluting first with AcOEt-*n*-hexane 2:1 v/v and then AcOEt to afford the *O*-benzyl hydroxamate intermediate **58** as a white solid (500 mg, 58%): mp 167-169°C (MeOH - Et_2O). This compound appears in the ^1H NMR spectrum as *E* and *Z* conformers (not

assigned). ^{1}H NMR (400 MHz, DMSO-d_6) δ 1.43 (d, 2H, *J* = 11.9 Hz, 4′e, 9′e-H), 1.55-1.68 (m, 4H, 6′, 8′e, 10′e-H), 1.75, 1.79 (s+s, 2H, 5′, 7′-H), 1.94 (s, 2H, 1′, 3′-H), 2.25 (t, 4H, *J* = 10.6 Hz, 4′a, 8′a, 9′a, 10′a-H), 3.12 (t, 1H, *J* = 8.4 Hz, 1-H), 3.56 (d, 2H, *J* = 8.4 Hz, 6-H), [3.63 (d, 1.7H, *J* = 5.1 Hz), 3.87-4.01 (br s, 0.2H), CONHC*H*$_2$CONHO], 4.29 (s, 2H, C*H*$_2$CONHCH$_2$), 4.78 (s, 2H, CONHOC*H*$_2$Ph), 7.26-7.46 (m, 5H, aromatic H), [8.07-8.26 (br s, 0.1 H), 8.34 (~t, 0.8H, *J* = 4.7 Hz), CH$_2$CON*H*CH$_2$], 11.17 (s, 1H, CON*H*OCH$_2$Ph); ^{13}C NMR (50 MHz, DMSO-d_6) δ 26.6, 26.9 (5′, 7′-C), 31.8 (1′, 3′-C), 32.0 (4′, 9′-C), 32.8 (8′,10′-C), 37.7 (6′-C), 39.9 (CONH*C*H$_2$CONHO), 41.1 (*C*H$_2$CONHCH$_2$), 44.0 (6-C), 59.4 (2,2′-C), 77.0 (CONHO*C*H$_2$Ph), 128.3, 128.8 (2, 3, 4, 5, 6-aromatic C), 135.8 (1-aromatic C), 165.9, 167.1 (*C*ONHCH$_2$*C*ONHO), 172.1, 174.4 (3,5-C). Anal. Calcd for $C_{24}H_{30}N_4O_5$: C, 63.42; H, 6.65; N, 12.33; Found: C, 63.58; H, 6.59; N, 12.04.

10% Pd on carbon (48 mg) was added to a solution of *O*-benzyl hydroxamate **58** (400 mg, 0.88 mmol) in MeOH (62 mL), and the mixture was hydrogenated for 3 h at room temperature under 50 psi of hydrogen in a Parr apparatus. The catalyst was then removed by filtration and washed with methanol (4x5 mL). The combined filtrates were concentrated to dryness under reduced pressure to leave an off-white solid. Silica gel column chromatography of this material using AcOEt – MeOH (1:0 to 7:3 v/v), as eluent, afforded the title compound **18** as a white crystalline solid (310 mg, 97%). To obtain a recrystallized specimen, compound **18** was dissolved in AcOEt-MeOH 100:1 v/v and precipitated by adding Et_2O to the clear solution; mp 158-160 ° C. ^{1}H NMR (400 MHz, DMSO-d_6) δ 1.42 (d, 2H, *J* = 11.7 Hz, 4′e, 9′e-H), 1.52-1.68 (m, 4H, 6′, 8′e, 10′e-H), 1.75, 1.79 (s+s, 2H, 5′, 7′-H), 1.94 (s, 2H, 1′, 3′-H), 2.25 (~t, 4H, *J* ≅ 11.5 Hz, 4′a, 8′a, 9′a, 10′a-H), 3.11 (t, 1H, *J* = 8.4 Hz, 1-H), 3.55 (d, 2H, *J* = 8.3 Hz, 6-H), [3.61 (d, 1.7H, *J* = 5.2 Hz, E-isomer), 3.93 (d, 0.25H, *J* = 3.9 Hz, Z-isomer), CONHC*H*$_2$CONHOH], 4.28 (s, 2H, C*H*$_2$CONHCH$_2$), [8.07 (~s, 0.1H, Z-isomer), 8.31 (t, 0.87H, *J* = 5.0 Hz, E-isomer), CH$_2$CON*H*CH$_2$], 8.61-9.45 (v br s, 1H, CONHO*H*), [9.87-10.25 (br s, 0.19H, Z-isomer), 10.28-10.95 (br s, 0.59H, E-isomer), CON*H*OH]; ^{13}C NMR (50 MHz, DMSO-d_6) δ 26.2, 26.5 (5′, 7′-C), 31.4 (1′, 3′-C), 31.6 (4′, 9′-C), 32.4 (8′,10′-C), 37.4 (6′-C), [39.1 (E-isomer), 39.6 (Z-isomer), CONH*C*H$_2$CONHOH], 40.8 (*C*H$_2$CONHCH$_2$), 43.6 (6-C), 59.0 (2,2′-C), 165.1, 166.6 (*C*ONHCH$_2$*C*ONHOH), 171.7, 174.1 (3,5-C). Anal. Calcd for $C_{17}H_{24}N_4O_5$: C, 56.03; H, 6.64; N, 15.38; Found: C, 55.86; H, 6.71; N, 15.19.

(*S*)-*N*-Hydroxy-2-[[6-(2-methylpropyl)-3,5-dioxospiro[piperazine-2,2′-tricyclo[3.3.1.1^{3,7}]dec-4-yl] acetyl]amino]acetamide 19

The *O*-benzyl protected hydroxamate **59** was prepared from compound **48** (780 mg, 1.92 mmol) according to the procedure described for the preparation of **58** (precursor for **18**). The crude product material (yellow viscous oil) was purified by column chromatography on silica gel eluting with AcOEt-*n*-hexane 1:1, 2:1 and 1:0 v/v in succession to give **59** as a slightly yellowish foamy solid, which strongly binds the elution solvents. Removal of the entrapped solvents upon drying at 62-64 °C under high vacuum (10^{-2} mmHg) in an Abderhalden apparatus afforded **59** as a glass solid (575 mg, 59%). This compound appears in the ^{1}H and ^{13}C NMR spectra as *E* and *Z* conformers (not assigned). ^{1}H NMR (600 MHz, $CDCl_3$) δ 0.95 (~s, 6H,

CH(CH_3)CH_3), 1.21-1.33 (m, 1H, 1-H), 1.40-1.51 (m, 2H, C*H*HCH(CH_3)$_2$, 4′e-H), 1.55 (d, 1H, J = 11.9 Hz, 9′e-H), 1.61-1.96 (complex m, 10H, 3′, 5′, 6′, 7′, 8′, 10′e-H, CH*H*C*H*(CH_3)$_2$), 2.08 (s, 1H, 1′-H), 2.15 (d, 1H, J = 11.6 Hz, 9′a-H), 2.41 (d, 1H, J = 11.3 Hz, 4′a-H), 2.87 (d, 1H, J = 11.6 Hz, 10′a-H), 3.56-3.67 (~br s, 1H, 6-H), [3.71-3.87 (~q, 1.4H, J = 13.7 Hz), 4.05-4.15 (br s, 0.34H), CONHCH_2CONHO], 4.20-4.54 (q, AB, 2H, J_{AB}=15.4 Hz, CH_2CONHCH_2), 4.82 (s, 2H, CONHOCH_2Ph), [6.64 (s, 0.1H), 7.14 (s, 0.6H), CH_2CON*H*CH_2], 7.27-7.39 (m, 5H, aromatic H), [8.76 (s, 0.1H), 9.75 (s, 0.6H), CON*H*OCH_2Ph]; ^{13}C NMR (50 MHz, $CDCl_3$) δ 21.3, 23.6 (CH(*C*H_3)*C*H_3), 24.7 (*C*H(CH_3)$_2$), 27.2, 27.5 (5′, 7′-C), 31.3 (1′-C), 31.8, 32.8, 33.3, 34.0 (4′, 8′, 9′, 10′-C), 35.1 (3′-C), 38.1 (6′-C), 41.4 (CONH*C*H_2CONHO), 41.8 (*C*H_2CH(CH_3)$_2$), 42.7 (*C*H_2CONHCH_2), 51.8 (6-C), 60.9 (2,2′-C), 78.5, 79.4 (CONHO*C*H_2Ph), 128.7, 129.3 (2, 3, 4, 5, 6-aromatic C), 134.3, 135.0 (1- aromatic C), 166.9, 167.6, 168.6, 172.3 (*C*ONHCH_2*C*ONHO), 175.1, 175.8 (3,5-C); ESI$^+$ MS: m/z 533.2 $[M+Na]^+$, 511.3 $[M+H]^+$.

Compound **59** (1.05 g, 2.06 mmol) was taken in abs EtOH (93 mL) and hydrogenated over 10% Pd-C (126 mg) as described for the preparation of compound **18** from **58**. The crude hydrogenation product (off-white foamy solid) was chromatographed on silica gel column eluting with AcOEt followed by AcOEt – MeOH 9:1 v/v to give a white foamy solid, which strongly binds the aforementioned solvents. Removal of the entrapped solvents upon drying at 55 °C under high vacuum (10^{-2} mmHg) in an Abderhalden apparatus afforded the title compound **19** as an off-white solid (830 mg, 96%). A recrystallized specimen was obtained upon dissolution of the solid in Et_2O- AcOEt 5:1 v/v and concentration of the clear solution to low volume, followed by addition of *n*-pentane to precipitate an off-white solid (decomposed gradually above 95 °C without distinct mp). ^{1}H NMR (600 MHz, DMSO-d_6) δ 0.91 (d, 3H, J = 2.2 Hz, CH(CH_3)CH_3), 0.92 (d, 3H, J = 2.5 Hz, CH(CH_3)CH_3), 1.38 (d, 1H, J = 11.4 Hz, 4′e-H), 1.43-1.52 (m, 2H, C*H*HCH(CH_3)$_2$, 9′e-H), 1.58 (d, 1H, J = 12.1 Hz, 10′e-H), 1.61-1.82 (complex m, 7H, 5′, 6′, 7′, 8′-H, CH*H*CH(CH_3)$_2$), 1.87 (s, 1H, 3′-H), 1.89-1.96 (m, 1H, C*H*(CH_3)$_2$), 2.10 (s, 1H, 1′-H), 2.14 (d, 1H, J = 11.7 Hz, 9′a-H), 2.46 (d, 1H, J = 11.7 Hz, 4′a-H), 2.65 (d, 1H, J = 11.7 Hz, 1-H), 2.82 (d, 1H, J = 12.1 Hz, 10′a-H), 3.43-3.50 (td, 1H, J = ~3.1, 11.2 Hz, 6-H), [3.56-3.66 (sym sept, 1.65H, *E*-isomer), 3.91-3.96 (~br s, 0.2H, *Z*-isomer), CONHCH_2CONHOH], 4.18-4.35 (q, AB, 2H, J_{AB} = 15.9 Hz, CH_2CONHCH_2), [8.01 (s, 0.1H, *Z*-isomer), 8.25 (t, 0.8H, J ≅ 5.6 Hz, *E*-isomer), CH_2CON*H*CH_2], [8.83 (s, 0.64H, *E*-isomer), 9.14 (s, 0.1H, *Z*-isomer), CONHO*H*], [10.09 (s, 0.1H, *Z*-isomer), 10.51 (s, 0.65H, *E*-isomer, CON*H*OH]; ^{13}C NMR (150 MHz, DMSO-d_6) δ 21.1, 23.4 (CH(*C*H_3)*C*H_3), 24.1 (*C*H(CH_3)$_2$), 26.6, 26.9 (5′, 7′-C), 30.4 (1′-C), 31.3, 32.3, 32.8, 33.4 (4′, 8′, 9′, 10′-C), 33.9 (3′-C), 37.8 (6′-C), 40.0 (CONH*C*H_2CONHOH), 40.9 (*C*H_2CH(CH_3)$_2$), 41.6 (*C*H_2CONHCH_2), 51.1 (6-C), 59.7 (2,2′-C), 165.6, 167.1 (*C*ONHCH_2*C*ONHOH), 174.6, 175.0 (3,5-C); $[\alpha]^{20}_{589}$ = - 29 (c, 0.2, $CHCl_3$); HRMS (ESI): $[M+Na]^+$ calcd for $C_{21}H_{32}N_4O_5$ 443.2270, found 443.2267. Anal. Calcd for $C_{21}H_{32}N_4O_5$: C, 59.98; H, 7.67; N, 13.32; Found: C, 59.73; H, 7.84; N, 13.05.

(*S*)-*N*-Hydroxy-2-[[3,5-dioxospiro-6-(phenylmethyl)spiro[piperazine-2,2′-tricyclo[3.3.1.1^{3,7}]dec-4-yl]acetyl]amino]acetamide 20

The *O*-benzyl protected hydroxamate **60** was prepared from compound **49** (910 mg, 2.07 mmol) according to the procedure described for the preparation of **58** (precursor for **18**). The crude product material

(yellowish viscous oil) was purified over a column of flash silica eluting with AcOEt-*n*-hexane 1:1 to 4:1 v/v to give **60** as a white foamy solid, which strongly binds the elution solvents. Removal of the entrapped solvents as in **59** (precursor for **19**) afforded **60** as a glass solid (627 mg, 57%). This compound appears in the ^{1}H and ^{13}C NMR spectra as *E* and *Z* conformers (not assigned). ^{1}H NMR (400 MHz, $CDCl_3$) δ 1.28-1.42 (m, 2H, 4′e, 9′e-H), 1.43-1.80 (complex m, 10H, 1, 3′, 4′a, 5′, 6′, 7′, 8′, 10′e-H), 1.93 (d, 1H, *J* = 11.4 Hz, 9′a -H), 2.03 (s, 1H, 1′-H), 2.72-2.96 (complex m, 2H, 10′a –H, C*H*HPh), 3.30 (d, 1H, *J* = 13.4 Hz, CH*H*Ph), [3.64-3.85 (~br s, 2.56H), 3.95-4.10 (br s, 0.43H), CONHCH_2CONHO, 6-H), 4.15-4.50 (q, AB, 2H, J_{AB} = 15.7 Hz, CH_2CONHCH_2), [4.72 (s), 4.77 (s), 2H, CONHOCH_2Ph], [6.64 (s, 0.14H), 7.04-7.38 (complex m, 10.86H), CH_2CON*H*CH_2, aromatic H], [8.63-9.30 (v br s, 0.10H), 9.55-10.12 (v br s, 0.4H), CON*H*OCH_2Ph]; ^{13}C NMR (50 MHz, $CDCl_3$) δ 27.0, 27.1 (5′,7′-C), 30.6 (1′-C), 31.4 (4′-C), 32.6 (9′-C), 33.3 (8′-C), 34.0 (10′-C), 34.9 (3′-C), 38.0 (6′-C, *C*H_2Ph), 40.7, 41.3 (CONH*C*H_2CONHO), 42.5 (*C*H_2CONHCH_2), 54.3 (6-C), 61.0 (2,2′-C), 78.5 (CONHO*C*H_2Ph), 127.0, 128.6, 128.7, 129.3, 135.0, 137.1 (aromatic C), 166.8, 168.5 (*C*ONHCH_2*C*ONHOCH_2Ph), 173.9, 174.6 (3,5-C); ESI$^+$ MS: m/z 545.4 $[M+H]^+$.

Compound **60** (660 mg, 1.21 mmol) was taken in abs EtOH (55 mL) and hydrogenated over 10% Pd-C (79 mg) as described for the preparation of compound **18** from **58**. The crude hydrogenation product (glass oil) was chromatographed on silica gel column, eluting with AcOEt followed by AcOEt – MeOH 9:1 v/v to give a slightly off-yellow foamy solid. This product material was dissolved in AcOEt (25 mL), and the clear solution was concentrated to almost dryness under reduced pressure. The residual viscous oil was triturated with Et_2O (30 mL), and the solid that formed was collected by filtration, washed with Et_2O (2x15 mL) and dried in vacuo affording the title compound **20** as an off-white solid (510 mg, 93%): mp 162-164 °C (dec); Purity (HPLC): 96.2%; ^{1}H NMR (600 MHz, DMSO-d_6) δ 1.27-1.33 (dd, 2H, *J* ~ 2.0, 12.2 Hz, 4′e, 9′e-H), 1.51-1.73 (complex m, 7H, 4′a, 6′, 7′, 8′, 10′e-H), 1.76 (s, 1H, 5′-H), 1.82 (s, 1H, 3′-H), 2.04 (s, 1H, 1′–H), 2.17 (d, 1H, *J* = 12.0 Hz, 9′a-H), 2.71-2.78 (complex m, 2H, 1-H, C*H*HPh), 2.81 (d, 1H, *J* = 12.2 Hz, 10′a-H), 3.29-3.34 (dd, 1H, under DMSO water signal, CH*H*Ph), [3.58-3.66 (sym sept, 1.72H, *E*-isomer), 3.92-3.97 (~br s, 0.2H, *Z*-isomer) CONHCH_2CONHOH], 3.68-3.76 (td, 1H, *J* ~ 3.4, 10.7 Hz, 6-H), 4.24-4.36 (q, AB, 2H, J_{AB} = 15.8 Hz, CH_2CONHCH_2), 7.15-7.38 (m, 5H, aromatic H), [8.05 (s, 0.1H, *Z*-isomer), 8.28 (t, 0.8H, *J* = 5.7 Hz, *E*-isomer), CH_2CON*H*CH_2], [8.84 (~br s, 0.5H, *E*-isomer), 9.15 (~br s, 0.02H, *Z*-isomer), CONHO*H*], [10.10 (~br s, 0.03H, *Z*-isomer), 10.53 (~br s, 0.5H, *E*-isomer), CON*H*OH]; ^{13}C NMR (50 MHz, DMSO-d_6) δ 26.5, 26.7 (5′,7′-C), 30.0 (1′-C), 30.9 (4′-C), 32.1 (9′-C), 32.7 (8′-C), 33.4 (10′-C), 34.0 (3′-C), 37.4 (*C*H_2Ph), 37.7 (6′-C), 40.0 (CONH*C*H_2CONHOH), 41.6 (*C*H_2CONHCH_2), 54.3 (6-C), 59.9 (2,2′-C), 126.2, 128.0, 129.1 (2, 3, 4, 5, 6-aromatic C), 138.7, (1- aromatic C), 165.5, 167.0 (*C*ONHCH_2*C*ONHOH), 173.7, 174.5 (3,5-C); $[\alpha]^{22}_{589}$= -39 (c, 0.2, DMSO); HRMS (ESI): $[M+Na]^+$ calcd for $C_{24}H_{30}N_4O_5$ 477.2114, found 477.2113. Anal. Calcd for $C_{24}H_{30}N_4O_5$: C, 63.42; H, 6.65; N, 12.33; Found: C, 63.73; H, 6.89; N, 12.14. The hydrochloride salt (**20.HCl**) was prepared by treating a clear solution of **20** in Et_2O- AcOEt 2:1 v/v with HCl-saturated Et_2O under ice cooling. The white precipitate was collected by vacuum filtration, triturated with Et_2O and dried in vacuo

(decomposed gradually above 122 ^{o}C without distinct mp). Anal. Calcd for $C_{24}H_{31}ClN_4O_5$: C, 58.71; H, 6.36; N, 11.41; Found: C, 58.35; H, 6.28; N, 11.18.

_N_-Hydroxy-2-[[3,5-dioxospiro[piperazine-2,2'-tricyclo[3.3.1.1^{3,7}]dec-4-yl]acetyl]methylamino] acetamide 21

A solution of compound **50** (620 mg, 1.71 mmol) and 1, 1'-carbonyldiimidazol (332 mg, 2.05 mmol) in dry THF (34 mL) was stirred at 28 °C under argon for 1 h. After this time, *O*-benzylhydroxylamine hydrochloride (327 mg, 2.05 mmol) and triethylamine (229 mg, 2.26 mmol) were added successively, and the mixture was allowed to stir at 28 °C under argon for 48h. The solvent was evaporated in vacuo, and the residue was partitioned between ethyl acetate (50 mL) and water (40 mL). The aqueous layer was extracted with ethyl acetate (3x40mL) and the combined organic solution was washed with brine (2x40 mL), dried (Na_2 SO_4) and concentrated to dryness under reduced pressure. The residual viscous oil was purified by column chromatography on silica gel eluting with AcOEt-*n*-hexane 1:1 followed by 2:1 v/v to give the *O*-benzyl protected hydroxamate **61** as a white foamy solid. The product material was then dried as in **59** (precursor for **19**), yet without change of the foamy solid state and color (600 mg, 75%). This compound appears in the ^{1}H and ^{13}C NMR spectra as two distinct conformers (not assigned). ^{1}H NMR (400 MHz, $CDCl_3$) δ 1.51 (d, 2H, *J* = 12.2 Hz, 4′e, 9′e-H), 1.63-1.93 (m, 7H, 1, 5′, 6′, 7′, 8′e, 10′e-H), 1.98 (s, 2H, 1′, 3′-H), 2.26 (d, 4H, *J* = 11.6 Hz, 4′a, 8′a, 9′a, 10′a-H), [2.82 (s), 3.08 (s), 3H, C*H₃*], 3.69 (s, 2H, 6-H), [3.92 (s), 3.97 (s), 2H, $CON(CH_3)CH_2CONHO$], [4.42 (s), 4.49 (s), 2H, $CH_2CON(CH_3)$], [4.86 (s), 4.96 (s), 2H, $CONHOCH_2Ph$], 7.28-7.47 (m, 5H, aromatic H), [9.36 (s), 10.09 (s), 1H, CON*H*OCH_2Ph]; ^{13}C NMR (50 MHz, $CDCl_3$) δ 27.2, 27.4 (5′, 7′-C), 32.4 (4′, 9′-C), 32.7 (1′, 3′-C), 33.3 (8′,10′-C), 35.2, 36.0 (*C*H_3), 38.1 (6′-C), 40.5 (*C*$H_2CON(CH_3)$), 44.5 (6-C), 51.0, 51.9 ($CON(CH_3)$*C*H_2CONHO), 60.6 (2,2′-C), 78.4 (CONHO*C*H_2Ph), 128.6, 128.8, 129.4 (2, 3, 4, 5, 6-aromatic C), 135.2 (1-aromatic C), 165.0, 165.9, 167.1, 168.0 (*C*ON(CH_3)CH_2*C*ONHO), 172.7, 174.9 (3,5-C); ESI$^+$ MS: m/z 491.2 $[M+Na]^+$, 469.2 $[M+H]^+$.

Compound **61** (1.0 g, 2.13 mmol) was taken in abs EtOH (95 mL) and hydrogenated over 10% Pd-C (120 mg) as described for the preparation of compound **18** from **58**. The crude hydrogenation product (off-white foamy solid) was chromatographed on silica gel column with AcOEt- MeOH 100:1 v/v, as eluent, to give an off-white gum. This product material was dissolved in AcOEt (30 mL), and the clear solution was concentrated to nearly dryness under vacuum. Trituration of the residue with Et_2O gave a white crystalline solid which strongly binds this solvent as indicated by its ^{1}H NMR spectrum. Removal of the crystal solvent upon drying at 62-64 °C under high vacuum (10^{-2} mmHg) in an Abderhalden apparatus afforded the title compound **21** as a white semifoamy solid (750 mg, 93%). The hydroxamic acid analogue **21** appears in the ^{1}H NMR spectrum as four distinct conformers due to the hindered rotation around the C(O)-N(CH_3) amide bond (cf. main text). ^{1}H NMR (400 MHz, DMSO-*d₆*) δ 1.43 (d, 2H, *J* = 11.7 Hz, 4′e, 9′e-H), 1.56-1.70 (m, 4H, 6′, 8′e, 10′e-H), 1.75, 1.79 (s+s, 2H, 5′, 7′-H), 1.95 (s, 2H, 1′, 3′-H), 2.18-2.32 (m, 4H, 4′a, 8′a, 9′a, 10′a-H), [2.75 (s), 2.77 (s), 3.02 (s), 3.05 (s), 3H, C*H₃*], 3.15 (t, 1H, *J* ≅ 8.0 Hz, 1-H), 3.56 (d, 2H, *J* = 7.4 Hz, 6-H), [3.81

(s), 3.94 (s), 4.13 (s), 4.28 (s), 2H, CON(CH_3)C*H_2*CONHOH], [4.34 (s), 4.72 (s), 4.94 (s), 2H, C*H_2*CON(CH_3)], [8.86 (s), 9.08 (s), 9.15 (s), 9.25 (s), 1H, CONHO*H*], [10.07 (s) , 10.30 (s), 10.56 (s), 10.73 (s), 1H, CON*H*OH]; ^{13}C NMR (50 MHz, DMSO-d_6) δ 26.7, 26.9 (5′, 7′-C), 31.9 (1′, 3′-C), 32.0 (4′, 9′-C), 32.8 (8′,10′-C), 34.3, 35.4 (*C*H_3), 37.8 (6′-C), 40.3, 40.4 (*C*H_2CON(CH_3)), 44.0 (6-C), 48.3, 49.0 (CON(CH_3)*C*H_2CONHOH), 59.4 (2,2′-C), 164.6, 165.0, 166.7, 167.0 (*C*ON(CH_3)CH_2*C*ONHOH), 172.1, 174.5 (3,5-C); HRMS (ESI): $[M+Na]^+$, $[M+H]^+$ calcd for $C_{18}H_{26}N_4O_5$ 401.1801, 379.1981, found 401.1795, 379.1977. Anal. Calcd for $C_{18}H_{26}N_4O_5$: C, 57.13; H, 6.93; N, 14.81; Found: C, 57.34; H, 7.05; N, 14.59.

(*S*)-*N*-Hydroxy-2-[[6-(2-methylpropyl)-3,5-dioxospiro[piperazine-2,2′-tricyclo[3.3.1.1^{3,7}]dec-4-yl]acetyl]methylamino]acetamide 22

The *O*-benzyl protected hydroxamate **62** was prepared from compound **51** (1.1 g, 2.62 mmol) according to the procedure described for the preparation of **61** (precursor for **21**). The crude product material (yellowish viscous oil) was purified by column chromatography on silica gel eluting with AcOEt-*n*-hexane 1:1 followed by 2:1 v/v to give an off-white foamy solid, which strongly binds the elution solvents. Removal of the entrapped solvents as in **59** (precursor for **19**) afforded **62** as an off-white semifoamy solid (980 mg, 71%). This *O*-benzyl hydroxamate compound appears in the ^{1}H NMR spectrum as four distinct conformers (not assigned) due to the hindered rotation around the C(O)-N(CH_3) amide bond. ^{1}H NMR (600 MHz, $CDCl_3$) δ 0.96 (d, 6H, *J* = 3.8 Hz, CH(C*H_3*)C*H_3*), 1.19 (d, 1H, *J* = 10.9 Hz, 1-H), 1.40-1.52 (m, 2H, 4′e-H, C*H*HCH(CH_3)$_2$), 1.56 (d, 1H, *J* = 11.8 Hz, 9′e-H), 1.63-1.79 (complex m, 5H, 6′, 8′, 10′e-H), 1.84, 1.87 (s+s, 3H, 3′, 5′, 7′-H), 1.89-1.97 (complex m, 2H, CH*H*C*H*(CH_3)$_2$), 2.13 (s, 1H, 1′-H), 2.16 (d, 1H, *J* = 12.3 Hz, 9′a-H), 2.42 (d, 1H, *J* = 12.3 Hz, 4′a-H), [2.82 (s), 3.06 (s), 3H, CON(C*H_3*)], 2.88 (d, 1H, *J* = 12.8 Hz, 10′a-H), 3.63 (~t, 1H, *J* = 8.2, 10.3 Hz, 6-H), [3.84 (d, *J* = 15.1 Hz), 3.91 (d, *J* = 17.7 Hz), 3.99 (~br d, *J* = 14.3 Hz), 2H, CON(CH_3)C*H_2*CONHO], [4.30 (d, *J* = 15.3 Hz), 4.36 (d, *J* = 15.4 Hz), 4.50 (d, *J* = 15.5 Hz), 4.55 (d, *J* = 15.4 Hz), 2H, C*H_2*CON(CH_3)], [4.85 (s), 4.95 (s), 2H, CONHOC*H_2*Ph], 7.28-7.42 (complex m, 5H, aromatic H), [9.25 (s), 10.02 (s), 1H, CON*H*OCH_2Ph]; ^{13}C NMR (50 MHz, $CDCl_3$) δ 21.3, 23.5 (CH(*C*H_3)*C*H_3), 24.6 (*C*H(CH_3)$_2$), 27.2, 27.4 (5′, 7′-C), 31.2 (1′-C), 31.7 (9′-C), 32.8 (4′-C), 33.3 (8′-C), 34.0 (10′-C), 35.1 (3′-C, CON(*C*H_3)), 36.0 (CON(*C*H_3)), 38.1 (6′-C), 40.9 (*C*H_2CON(CH_3)), 41.8 (*C*H_2CH(CH_3)$_2$), 50.9 (CON(CH_3)*C*H_2CONHO), 51.7 (6-C), 60.8 (2,2′-C), 78.3 (CONHO*C*H_2Ph), 128.5, 128.7, 129.3 (2, 3, 4, 5, 6-aromatic C), 135.2 (1-aromatic C), 164.9, 165.8, 167.1, 168.1 (*C*ON(CH_3)CH_2*C*ONHO), 175.0, 175.8 (3,5-C); ESI$^+$ MS: m/z 525.3 $[M+H]^+$.

Compound **62** (1.8 g, 3.43 mmol) was taken in abs EtOH (154 mL) and hydrogenated over 10% Pd-C (216 mg) as described for the preparation of compound **18** from **58**. The crude hydrogenation product (white foamy solid) was chromatographed on silica gel column, eluting first with AcOEt and then AcOEt – MeOH 9:1 v/v to give an off-white foamy solid. Removal of the entrapped solvents from this material upon drying as in **19** afforded the title compound **22** as an off-white semifoamy solid (1.43 g, 96%). A quantity of the dry product material was dissolved in AcOEt-Et_2O 1:2 v/v, and the clear solution was concentrated to low volume. Addition of *n*-pentane precipitated compound **22** as a white crystalline solid, which was collected

by vacuum filtration, and dried (decomposed gradually above 99 °C without distinct mp). The hydroxamic acid analogue **22** appears in the ^{1}H NMR spectrum as four distinct conformers due to the hindered rotation around the C(O)-N(CH$_3$) amide bond (cf. main text). ^{1}H NMR (600 MHz, DMSO-d_6) δ 0.91 (d, 3H, *J* = 1.5 Hz, CH(C$\mathit{H_3}$)CH$_3$), 0.93 (d, 3H, *J* = 1.7 Hz, CH(CH$_3$)C$\mathit{H_3}$), 1.38 (d, 1H, *J* = 11.3 Hz, 4′e-H), 1.44-1.53 (m, 2H, 9′e-H, C*H*HCH(CH$_3$)$_2$), 1.58 (d, 1H, *J* = 11.4 Hz, 10′e-H), 1.61-1.75 (m, 5H, 6′, 8′, CH*H*CH(CH$_3$)$_2$), 1.77, 1.79 (s+s, 2H, 5′, 7′-H), 1.88 (s, 1H, 3′-H), 1.90-1.98 (m, 1H, C*H*(CH$_3$)$_2$), 2.08 (s, 1H, 1'-H), 2.15 (d, 1H, *J* = 11.7 Hz, 9′a-H), 2.47 (d, 1H, *J* = 11.7 Hz, 4′a-H), 2.67 (d, 1H, *J* = 11.6 Hz, 1-H), [2.77 (s), 3.02 (s), 3.04 (s), 3.05 (s), 3H, CON(C$\mathit{H_3}$)], 2.83 (d, 1H, *J* = 12.1 Hz, 10′a-H), 3.43-3.51 (~ td, 1H, *J* ≅ 2.2, 2.7, 10.9 Hz, 6-H), [3.78-3.85 (m), 3.86-4.0 (q, *J* = 16.7 Hz), 4.10-4.38 (m), 4.39-4.55 (complex m), 4H, C$\mathit{H_2}$CON(CH$_3$)C$\mathit{H_2}$CONHOH], [8.62-8.95 (v br s), 8.96-9.33 (v br s+v br s), 1H, CONHO*H*], [10.03 (s), 10.26 (s), 10.36-10.91 (br s), 1H, CON*H*OH]; ^{13}C NMR (150 MHz, DMSO-d_6) δ 21.1 (CH(*C*H$_3$)CH$_3$), 23.4 (CH(CH$_3$)*C*H$_3$), 24.0 (*C*H(CH$_3$)$_2$), 26.6, 26.9 (5′, 7′-C), 30.4 (1′-C), 31.3 (9′-C), 32.3 (4′-C), 32.8 (8′-C), 33.4 (10′-C), 33.8 (3′-C), 34.3, 35.4 (CON(*C*H$_3$)), 37.8 (6′-C), 40.6, 40.7, 40.8, 40.9 (*C*H$_2$CON(CH$_3$), *C*H$_2$CH(CH$_3$)$_2$), 48.3, 49.0 (CON(CH$_3$)*C*H$_2$CONHOH), 51.0, 51.1 (6-C), 59.7 (2,2’-C), 164.6, 165.0, 166.7, 167.0 (*C*ON(CH$_3$)CH$_2$*C*ONHOH), 174.5, 174.9 (3,5-C); $[\alpha]^{23}_{589}$= -25 (c, 0.2, CHCl$_3$); HRMS (ESI): [M+Na]$^+$ calcd for $C_{22}H_{34}N_4O_5$ 457.2427, found 457.2424. Anal. Calcd for $C_{22}H_{34}N_4O_5$: C, 60.81; H, 7.89; N, 12.89; Found: C, 61.02; H, 7.98; N, 12.65.

(*S*)-*N*-Hydroxy-2-[[3,5-dioxo-6-(phenylmethyl)spiro[piperazine-2,2’-tricyclo[3.3.1.1^{3,7}]dec-4-yl]acetyl]methylamino]acetamide 23

The *O*-benzyl protected hydroxamate **63** was prepared from compound **52** (1.0 g, 2.20 mmol) according to the procedure described for the preparation of **61** (precursor for **21**). The crude product material (yellowish viscous oil) was purified by flash column chromatography (silica gel) eluting with AcOEt-*n*-hexane 1:1 followed by 2:1 v/v to give a white foamy solid, which strongly binds the elution solvents. Removal of the entrapped solvents as in **59** (precursor for **19**) afforded **63** as a white semifoamy solid (800 mg, 65%). This *O*-benzyl hydroxamate compound appears in the ^{1}H NMR spectrum as four distinct conformers (not assigned) due to the hindered rotation around the C(O)-N(CH$_3$) amide bond. ^{1}H NMR (600 MHz, CDCl$_3$) δ 1.37-1.47 (m, 2H, 4′e, 9′e-H), 1.52 (d, 1H, *J* = 8.3 Hz, 1-H), 1.57-1.75 (complex m, 7H, 5′, 6′, 8′, 9′a, 10′e-H), 1.80, 1.82 (s+s, 2H, 3′, 7′-H), 2.02 (d, 1H, *J* = 10.7 Hz, 4′a-H), 2.15 (s, 1H, 1′-H), [2.75 (s), 2.83 (s), 3.07 (s), 3H, C$\mathit{H_3}$], 2.89 (d, 1H, *J* = 12.5 Hz, 10′a-H), 2.92-2.99 (q, 1H, *J* = 8.6 Hz, C*H*HPh), 3.32-3.43 (dd, 1H, *J* ≅ 3.8, 13.7 Hz, CH*H*Ph), 3.76-4.02 (complex m, 3H, 6-H, CON(CH$_3$)C$\mathit{H_2}$CONHO), 4.34-4.62 (complex m, 2H, C$\mathit{H_2}$CON(CH$_3$)), [4.77 (s), 4.86 (s), 4.96 (s), 2H, CONHOC$\mathit{H_2}$Ph], 7.16-7.47 (complex m, 10, aromatic H), [8.09 (s), 8.89 (~br s), 9.41 (s), 10.10 (s), 1H, CON*H*OCH$_2$Ph]; ^{13}C NMR (150 MHz, CDCl$_3$) δ 27.0 (5′,7′-C), 30.5 (1′-C), 31.3 (9′-C), 32.5 (4′-C), 33.2 (8′-C), 33.9 (10′-C), 34.9 (3′-C, *C*H$_3$), 36.0 (*C*H$_3$), 37.9, 38.0 (*C*H$_2$C$_6$H$_5$, 6′-C), 40.7 (*C*H$_2$CON(CH$_3$)), 50.7, 51.4 (CON(CH$_3$)*C*H$_2$CONHO), 54.1 (6-C), 60.3, 60.9 (2,2′-C), 78.2, 79.2 (CONHO*C*H$_2$Ph), 127.0, 128.4, 128.6, 129.2, 135.2, 137.0, 137.1 (aromatic C), 165.1 , 165.9, 167.1, 167.9 (*C*ON(CH$_3$)CH$_2$*C*ONHO), 173.7, 173.9, 174.5 (3,5-C); ESI$^+$ MS: m/z 581.3 [M+Na]$^+$, 559.3 [M+H]$^+$.

Compound **63** (1.35 g, 2.42 mmol) was taken in abs EtOH (109 mL) and hydrogenated over 10% Pd-C (162 mg) as described for the preparation of compound **18** from **58**. The crude hydrogenation product (off-white foamy solid) was chromatographed on silica gel column with AcOEt, as eluent, to give a white foamy solid. Removal of the entrapped solvents from this material upon drying as in **19** afforded the title compound **23** as a white semifoamy solid (1.07 g, 95%) A quantity of the dry product material was dissolved in AcOEt-Et_2O 1:2 v/v, and the clear solution was concentrated to low volume. Addition of diethyl ether - *n*-pentane 1:1 v/v mixture precipitated a clear gum, which solidified upon cooling at 0-5 ^{o}C for a few hours. The supernatant solution was decanted off, and the white crystalline solid was triturated with a diethyl ether – *n*-pentane 1:1 v/v mixture, collected by vacuum filtration and dried (decomposed gradually above 93 °C without distinct mp). The hydroxamic acid analogue **23** appears in the ^{1}H NMR spectrum as four distinct conformers due to the hindered rotation around the C(O)-N(CH_3) amide bond (cf. main text). ^{1}H NMR (600 MHz, DMSO-d_6) δ 1.25-1.36 (m, 2H, 4′e, 9′e-H), 1.45-1.79 (complex m, 8H, 5′, 6′, 7′, 8′, 9'a, 10′e-H), 1.83 (s, 1H, 3′-H), 2.07 (s, 1H, 1'-H), 2.17 (d, 1H, *J* = 11.8 Hz, 4'a-H), 2.69-2.87 (complex m, 3H, 1, 10'a-H, C*H*HPh), [2.77 (s), 3.04 (s), 3.07 (s), 3H, C*H₃*], 3.28-3.34 (dt, 1H, *J* = 3.4, 13.9 Hz, CH*H*Ph), 3.66-3.77 (td, 1H, *J* = 3.5, 10.6 Hz, 6-H), [3.83 (s), 3.89-4.01 (q, *J* = 16.7 Hz), 4.16 (s), 4.23-4.33 (q, *J* ~ 19.0 Hz), 2H, CON(CH_3)CH_2CONHOH], [4.37 (d, *J* = 8.4 Hz), 4.44-4.59 (complex m), 2H, CH_2CON(CH_3)], 7.14-7.40 (complex m, 5H, aromatic H), [8.84 (s), 9.06 (s), 9.13 (s), 9.22 (s)] 1H, CONHO*H*], [10.05 (s), 10.28 (s), 10.54 (s), 10.72 (s), 1H, CON*H*OH]; ^{13}C NMR (150 MHz, DMSO-d_6) δ 26.6, 26.8 (5′, 7′-C), 30.0 (1′-C), 30.9 (9′-C), 32.1 (4'-C), 32.7 (8′-C), 33.4 (10'-C), 34.0 (3'-C), 34.3, 35.4 (*C*H₃), 37.4, 37.7 (*C*H₂Ph, 6′-C), 40.5, 40.7 (*C*H₂CON(CH_3)), 48.3, 49.1 (CON(CH_3)*C*H₂CONHOH), 54.4 (6-C), 59.9 (2,2′-C), 126.2, 128.0, 129.1 (2, 3, 4, 5, 6-aromatic C), 138.6 (1-aromatic C), 164.6, 165.0, 166.6, 167.0 (*C*ON(CH_3)CH_2*C*ONHOH), 173.6, 174.4 (3,5-C); $[\alpha]^{24}_{589}$= -43.5 (c, 0.2, $CHCl_3$); HRMS (ESI): $[M+Na]^+$, $[M+H]^+$ calcd for $C_{25}H_{32}N_4O_5$ 491.2270, 469.2451, found 491.2267, 469.2451. Anal. Calcd for $C_{25}H_{32}N_4O_5$: C, 64.08; H, 6.88; N, 11.96; Found: C, 64.25; H, 6.97; N, 11.77.

_N_-Hydroxy-2-[[[3,5-dioxo-1,4-diazaspiro[5.7]tridec-4-yl]acetyl]amino]acetamide 24

The *O*-benzyl protected hydroxamate **64** was prepared from compound **53** (600 mg, 1.84 mmol) according to the procedure described for the preparation of **58** (precursor for **18**). The crude product material (yellow viscous oil) was purified by column chromatography on silica gel eluting with AcOEt-*n*-hexane 1:1 v/v followed by AcOEt-MeOH 7:3 v/v containing 0.5% Et_3N to afford **64** as an off-white solid (620 mg, 78%). To obtain an analytical sample, a small quantity of this material was recrystallized from MeOH-Et_2O giving a white solid which melted at 182-184 ° C. This compound appears in the ^{1}H NMR spectrum as *E* and *Z* conformers (not assigned). ^{1}H NMR (400 MHz, DMSO-d_6) δ 1.38-1.72 (m, 12H, 7, 8, 9, 10, 11, 12, 13-H), 1.90-2.04 (q, 2H, *J* ≅ 9.0 Hz, 7,13-H), 2.97 (t, 1H, *J* =8.0 Hz, 1-H), 3.57 (d, 2H, *J* = 7.8 Hz, 2-H), [3.63 (d, 1.7H, *J* = 5.2 Hz), 3.86-3.99 (br s, 0.2H, CONHC*H₂*CONHO], 4.28 (s, 2H, C*H₂*CONHCH₂), 4.79 (s, 2H, CONHOC*H₂*Ph), 7.30-7.43 (m, 5H, aromatic-H), [8.10-8.22 (br s, 0.12H), 8.34 (~t, 0.85H, *J* = 4.9 Hz), CH_2CON*H*CH₂], 11.19 (s, 1H, CON*H*OCH₂Ph); ^{13}C NMR (50 MHz, DMSO-*d6*) δ 20.6 (9,11-C), 24.0 (10-C), 27.3

(8,12-C), 29.9 (7,13-C), 39.5 (CONH*C*H_2CONHO), 40.1 (*C*H_2CONHCH_2), 44.4 (2-C), 58.6 (6-C), 76.6 (CONHO*C*H_2Ph), 127.9, 128.4 (2,3,4,5,6–aromatic C), 135.5 (1-aromatic C), 165.5, 166.6 (*C*ONHCH_2*C*ONHO), 171.1, 175.9 (3,5-C). Anal. Calcd For $C_{22}H_{30}N_4O_5$: C, 61.38; H, 7.02; N, 13.01; Found: C, 61.03; H, 7.07; N, 12.85.

Compound **64** (490 mg, 1.14 mmol) was taken in MeOH (80 mL) and hydrogenated over 10% Pd-C (59 mg) as described for the preparation of compound **18** from **58**. The crude hydrogenation product (white foamy solid) was chromatographed on silica gel column with AcOEt- MeOH 7:3 v/v, as eluent, to afford the title compound as a white crystalline solid (340 mg, 88%): mp 176-179 °C (dec) (MeOH-Et_2O); ^{1}H NMR (400 MHz, DMSO-*d₆*) δ 1.35-1.73 (complex m, 12H, 7, 8, 9, 10, 11, 12, 13-H), 1.88-2.03 (~q, 2H, *J* ≅ 9.0 Hz, 7,13-H), 2.98 (t, 1H, *J* =8.3 Hz, 1-H), 3.56 (d, 2H, *J* = 8.3 Hz, 2-H), [3.60 (d, 1.79H, *J* = 5.6 Hz, *E*-isomer), 3.93 (d, 0.25H, *J* = 4.5 Hz, *Z*-isomer), CONHCH_2CONHOH], 4.26 (s, 2H, CH_2CONHCH_2), [8.06 (s, 0.11H, *Z*-isomer), 8.29 (~t, 0.85H, *J* = 5.6 Hz, *E*-isomer), CH_2CON*H*CH_2], 8.60-9.50 (v br s+v br s, 1H, CONHO*H*), 9.95-10.90 (v br s+v br s, 1H, CON*H*OH); ^{13}C NMR (50 MHz, DMSO-*d₆*) δ 21.0 (9,11-C), 24.4 (10-C), 27.7 (8,12-C), 30.3 (7,13-C), 39.9 (CONH*C*H_2CONHOH), 40.6 (*C*H_2CONHCH_2), 44.8 (2-C), 59.0 (6-C), 165.5, 166.9 (*C*ONHCH_2*C*ONHOH), 171.5, 176.3 (3,5-C). Anal. Calcd For $C_{15}H_{24}N_4O_5$: C, 52.93; H, 7.11; N, 16.46; Found: C, 52.68; H, 6.97; N, 16.07.

(*S*)-*N*-Hydroxy-2-[[[2-(2-methylpropyl)-3,5-dioxo-1,4-diazaspiro[5.7]tridec-4-yl]acetyl]amino] acetamide 25

The *O*-benzyl protected hydroxamate **65** was prepared from compound **54** (480 mg, 1.26 mmol) according to the procedure described for the preparation of **58** (precursor for **18**). The crude product material (off-yellow viscous oil) was purified by column chromatography on silica gel with AcOEt-*n*-hexane 2:1 v/v, as eluent, to give a white foamy solid, which strongly binds the elution solvents. Removal of the entrapped solvents as in **59** (precursor for **19**) afforded **65** as a glass solid (442 mg, 72%). This compound appears in the ^{1}H NMR spectrum as *E* and *Z* conformers (not assigned). ^{1}H NMR (400 MHz, $CDCl_3$) δ 0.90 (d, 3H, *J* = 5.1 Hz, CH(C*H₃*)CH_3), 0.94 (d, 3H, *J* = 5.1 Hz, CH(CH_3)C*H₃*), 1.18-2.01 (complex m, 17H, 1, 7, 8, 9, 10, 11, 12, 13-H, CH_2C*H*(CH_3)$_2$), 2.10-2.31 (~q, 1H, *J* ~ 9.7 Hz, 13-H), 3.59 (d, 1H, *J* = 8.7 Hz, 2-H), [3.78 (d, 1.4H, *J* = 3.0 Hz), 4.09 (s, 0.4H), CONHCH_2CONHOH], 4.36 (s, 2H, CH_2CONHCH_2), 4.82 (s, 2H, CONHOCH_2Ph), [6.71 (s, 0.14H), 7.18-7.42 (m, 5.7H), CH_2CON*H*CH_2, aromatic H], [8.92 (s, 0.13H), 9.81 (s, 0.66H), CON*H*OCH_2Ph]; ^{13}C NMR (50 MHz, $CDCl_3$) δ 20.8 (9-C), 21.2 (CH(*C*H_3)*C*H_3), 21.4 (11-C), 23.5 (*C*H(CH_3)*C*H_3), 24.5 (*C*H(CH_3)$_2$), 24.6 (10-C), 27.4 (8-C), 28.5, 28.6 (7,12-C), 34.5 (13-C), 40.4 (*C*H_2CH(CH_3)$_2$, CONH*C*H_2CONHO), 41.4 (CONH*C*H_2CONHO), 41.9 (*C*H_2CONHCH_2), 51.8 (2-C), 60.4 (6-C), 78.5 (CONHO*C*H_2Ph), 128.6, 129.3 (2,3,4,5,6–aromatic C), 135.0 (1-aromatic C), 166.9, 168.5 (*C*ONHCH_2*C*ONHO), 174.5, 177.4 (3,5-C); EI MS: m/z 487.2 ($[M+H]^+$,4), 486.2 ($[M]^+$,10), 430.0 ($[M+H-CH_2CH(CH_3)_2]^+$,16), 306.1 (100), 279.1 (31), 251.1 (60).

Compound **65** (400 mg, 0.82 mmol) was taken in abs EtOH (37 mL) and hydrogenated over 10% Pd-C (48 mg) as described for the preparation of compound **18** from **58**. The crude hydrogenation product (off-white foamy solid) was chromatographed on silica gel column eluting with AcOEt- MeOH 1:0 to 7:3 v/v to give a white foamy solid, which strongly binds the elution solvents. Removal of the entrapped solvents as in **59** (precursor for **19**) afforded the title compound **25** as a white semifoamy solid (308 mg, 95%): ^{1}H NMR (400 MHz, DMSO-d_6) δ 0.88 (d, 3H, *J* = 6.5 Hz, CH(C*H*$_3$)CH$_3$), 0.91 (d, 3H, *J* = 6.6 Hz, CH(CH$_3$)C*H*$_3$), 1.34-1.97 (complex m, 16H, 7, 8, 9, 10, 11, 12, 13-H, C*H*$_2$C*H*(CH$_3$)$_2$), 2.0-2.11 (q, 1H, *J* = 9.7 Hz, 13-H), 2.55 (d, partially coincides with DMSO signal, 1-H), 3.48 (~t, 1H, *J* ~ 12.6 Hz, 2-H), [3.59 (d, 1.62H, *J* = 5.6 Hz, *E*-isomer), 3.92 (d, 0.2H, *J* = 6.5 Hz, *Z*-isomer), CONHC*H*$_2$CONHOH], 4.24 (s, 2H, C*H*$_2$CONHCH$_2$), [8.04 (t, 0.1H, *J* ~ 5.6 Hz, *Z*-isomer), 8.26 (t, 0.73H, *J* = 5.6 Hz, *E*-isomer), CH$_2$CON*H*CH$_2$], [8.86 (s, 0.72H, *E*-isomer), 9.18 (s, 0.1H, *Z*-isomer), CONHO*H*], [10.12 (s, 0.1H, *Z*-isomer), 10.53 (s, 0.7H, *E*-isomer, CON*H*OH]; ^{13}C NMR (50 MHz, DMSO-d_6) δ 20.3 (9-C), 21.06 (11-C), 21.1, 23.4 (CH(*C*H$_3$)*C*H$_3$), 24.0 (*C*H(CH$_3$)$_2$), 24.2 (10-C), 27.0, 27.8 (8,12-C), 28.3 (7-C), 33.4 (13-C), 39.6 (*C*H$_2$CH(CH$_3$)$_2$, CONH*C*H$_2$CONHOH), 40.0 (CONH*C*H$_2$CONHOH), 41.1 (*C*H$_2$CONHCH$_2$), 51.1 (2-C), 59.4 (6-C), 165.6, 167.0 (*C*ONHCH$_2$*C*ONHOH), 174.0, 176.9 (3,5-C); HRMS (ESI): m/z [M+Na]$^+$, [M+H]$^+$ calcd for $C_{19}H_{32}N_4O_5$ 419.2270, 397.2451, found 419.2267, 397.2450. The hydrochloride salt (**25.HCl**) was prepared by treating a clear diethyl ether solution of compound **25** with saturated solution of HCl in Et_2O under ice cooling. The white solid that formed was collected by vacuum filtration, triturated with Et_2O and dried in vacuo (decomposed gradually above 147 ^{o}C without distinct mp); $[\alpha]_{589}^{22}$= -13.5 (c, 0.2, DMSO); Anal. Calcd for $C_{19}H_{33}ClN_4O_5$: C, 52.71; H, 7.68; N, 12.94; Found: C, 52.36; H, 7.79; N, 12.68.

N-Hydroxy-2-[[[3,5-dioxo-1,4-diazaspiro[5.7]tridec-4-yl]acetyl]methylamino]acetamide 26

The *O*-benzyl protected hydroxamate **66** was prepared from compound **55** (770 mg, 2.27 mmol) according to the procedure described for the preparation of **61** (precursor for **21**). The crude product material (yellowish viscous oil) was purified by column chromatography on silica gel eluting with AcOEt-*n*-hexane 2:1 v/v and then AcOEt-MeOH 1:0 to 8:2 v/v. Evaporation of the appropriate fractions and removal of the remaining traces of solvents under high vacuum (10^{-2} mmHg) gave **66** as a white semifoamy solid (640 mg, 64%). A small quantity of this product material was dissolved in AcOEt, and the clear solution was concentrated to low volume. Addition of Et_2O precipitated compound **66** as a white crystalline solid, which was collected by vacuum filtration, washed with Et_2O and dried; mp 158-160 °C. This compound appears in the ^{1}H and ^{13}C NMR spectra as two distinct conformers (not assigned). ^{1}H NMR (600 MHz, $CDCl_3$) δ 1.40-1.74 (complex m, 12H, 7, 8, 9, 10, 11, 12, 13-H), 1.73-1.87 (br s, 1H, 1-H), 2.04-2.15 (q, 2H, *J* ≅ 9.6 Hz, 7,13-H), [2.81 (s), 3.06 (s), 3H, C*H*$_3$], 3.68 (s, 2H, 2-H), [3.89 (s), 3.94 (s), 2H, CON(CH$_3$)C*H*$_2$CONHO], [4.41 (s), 4.48 (s), 2H, C*H*$_2$CON(CH$_3$)], [4.84 (s), 4.93 (s), 2H, CONHOC*H*$_2$Ph], 7.27-7.41 (m, 5H, aromatic-H), [9.37 (s), 10.07 (s), 1H, CON*H*OCH$_2$Ph]; ^{13}C NMR (50 MHz, $CDCl_3$) δ 21.4 (9,11-C), 24.8 (10-C), 28.1 (8,12-C), 31.0 (7,13-C), 35.1, 36.0 (*C*H$_3$), 39.7 (*C*H$_2$CON(CH$_3$)), 45.2 (2-C), 50.7, 51.6 (CON(CH$_3$)*C*H$_2$CONHO), 60.0 (6-C), 78.4 (CONHO*C*H$_2$Ph), 128.5, 128.7, 129.4 (2,3,4,5,6–aromatic C), 135.2 (1-aromatic C), 165.8, 167.7

(CON(CH_3)$CH_2$$C$ONHO$CH_2$Ph), 171.7, 176.7 (3,5-C). Anal. Calcd for $C_{23}H_{32}N_4O_5$: C, 62.14; H, 7.26; N, 12.60; Found: C, 62.26; H, 7.09; N, 12.76.

Compound **66** (1.1 g, 2.47 mmol) was taken in abs EtOH-AcOEt 3:2 v/v (112 mL) and hydrogenated over 10% Pd-C (132 mg) as described for the preparation of compound **18** from **58**. The crude hydrogenation product (off-white foamy solid) was chromatographed on silica gel column with AcOEt-MeOH 9:1 v/v, as eluent, to give a white foamy solid. This product material was dissolved in AcOEt-MeOH 100:1 v/v, and the clear solution was concentrated to dryness under reduced pressure. Trituration of the residual white gum with Et_2O (20 mL) afforded the title compound **26** as a white crystalline solid, which was collected by vacuum filtration, washed with Et_2O (10 mL) and dried (835 mg, 95%) ; decomposed gradually above 102 °C without distinct mp. The hydroxamic acid analogue **26** appears in the 1H NMR spectrum as four distinct conformers due to the hindered rotation around the C(O)-N(CH_3) amide bond (cf. main text). ^{1}H NMR (600 MHz, DMSO-d_6) δ 1.40-1.57 (m, 8H, 8, 9, 10, 11, 12-H), 1.58-1.70 (m, 4H, 7, 8, 12, 13-H), 1.93-2.02 (m, 2H, 7, 13-H), [2.76 (s), 2.78 (s), 3.02 (s), 3.05 (s), 3H, CH_3], 2.98 (br s, 1H, 1-H), 3.56 (s, 2H, 2-H), [3.81 (s), 3.93 (s), 4.13 (s), 4.27 (s), 2H, CON(CH_3)CH_2CONHOH], [4.32 (s), 4.45 (s), 4.47 (s), 2H, CH_2CON(CH_3)], [8.84 (s), 9.05 (s), 9.13 (s), 9.24 (s), 1H, CONHO*H*], [10.03 (s), 10.26 (s), 10.53 (s), 10.67 (s), 1H, CON*H*OH]; ^{13}C NMR (50 MHz, DMSO-d_6) δ 21.1 (9,11-C), 24.5 (10-C), 27.8 (8,12-C), 30.4 (7,13-C), 34.3, 35.4 (CH$_3$), 39.5, 39.7 (CH$_2$CON(CH_3)), 44.8 (2-C), 48.4, 49.0 (CONHCH$_2$CONHOH), 59.0 (6-C), 164.6, 165.0, 166.5, 166.9 (CON(CH_3)$CH_2$$C$ONHOH), 171.5, 176.2, 176.3 (3,5-C). HRMS (ESI): $[M+Na]^+$ calcd for $C_{16}H_{26}N_4O_5$ 377.1801, found 377.1800. Anal. Calcd for $C_{16}H_{26}N_4O_5$: C, 54.22; H, 7.40; N, 15.81; Found: C, 54.51; H, 7.48; N, 15.56.

N-Hydroxy-2-[[[3,5-dioxo-1,4-diazaspiro[5.6]dodec-4-yl]acetyl]amino]acetamide 27

The *O*-benzyl protected hydroxamate **67** was prepared from compound **56** (750 mg, 2.4 mmol) according to the procedure described for the preparation of **58** (precursor for **18**). The crude product material (off-yellow solid) was purified by flash column chromatography (silica gel) eluting with AcOEt-*n*-hexane 1:1 v/v and then AcOEt-MeOH 1:0 to 7:3 v/v to afford **67** as a white crystalline solid (626 mg, 63%). A recrystallised sample had mp 156-158 °C (from MeOH-Et_2O). This compound appears in the ^{1}H NMR spectrum as *E* and *Z* conformers (not assigned). ^{1}H NMR (400 MHz, DMSO-d_6) δ 1.43-1.71 (complex m, 10H, 7, 8, 9, 10, 11, 12-H), 1.76-1.97 (q, 2H, $J \cong 9.0$ Hz, 7,12-H), 3.01 (t, 1H, $J = 8.3$ Hz, 1-H), 3.57 (d, 2H, $J = 8.1$ Hz, 2-H), [3.63 (d, 1.7H, $J = 5.1$ Hz), 3.89-3.99 (br s, 0.2H), CONHCH_2CONHO], 4.29 (s, 2H, CH_2CONHCH_2), 4.79 (s, 2H, CONHOCH_2Ph), 7.30-7.43 (m, 5H, aromatic H), [8.10-8.22 (br s, 0.1H), 8.33 (t, 0.8H, $J = 4.9$ Hz), CH_2CON*H*CH_2], 11.05-11.28 (br s, 1H, CON*H*OCH_2Ph); ^{13}C NMR (50 MHz, DMSO-d_6) δ 21.8 (8,11-C), 29.4 (9,10-C), 35.1 (7,12-C), 39.9 (CONHCH$_2$CONHO), 40.6 (CH$_2$CONHCH_2), 44.7 (2-C), 59.7 (6-C), 77.0 (CONHOCH$_2$Ph), 128.3, 128.8 (2, 3, 4, 5, 6-aromatic C), 135.8 (1-aromatic C), 165.9, 167.0 (CONH$CH_2$$C$ONHO), 171.5, 176.9 (3,5-C). Anal. Calcd For $C_{21}H_{28}N_4O_5$: C, 60.56; H, 6.78; N, 13.45; Found: C, 60.67; H, 6.85; N, 13.28.

Compound **67** (1.1 g, 2.64 mmol) was taken in MeOH (185 mL) and hydrogenated over 10% Pd-C (132 mg) as described for the preparation of compound **18** from **58**. The crude hydrogenation product (off-white solid) was chromatographed on silica gel column with AcOEt-MeOH 7:3 v/v, as eluent, to afford the title compound **27** as a white solid (776 mg, 90%). Recrystallization from MeOH-Et_2O furnished an analytical specimen, which had mp 172-174 °C (dec). ^{1}H NMR (400 MHz, DMSO-d_6) δ 1.43-1.71 (m, 10H, 7, 8, 9, 10, 11, 12-H), 1.84-1.96 (q, 2H, *J* ≅ 9.0 Hz, 7,12-H), 3.01 (t, 1H, *J* ≅ 8.3 Hz, 1-H), 3.55 (d, 2H, *J* = 8.2 Hz, 2-H), [3.60 (d, 1.8H, *J* = 5.6 Hz, *E*-isomer), 3.93 (d, 0.2H, *J* = 4.8 Hz, *Z*-isomer), CONHCH_2CONHOH], 4.26 (s, 2H, CH_2CONHCH_2), [8.06 (~s, 0.1H, *Z*-isomer), 8.29 (t, 0.8H, *J* = 5.6 Hz, *E*-isomer), CH_2CON*H*CH_2], 8.67-9.28 (v br s+v br s, 1H, CONHO*H*), [9.92-10.25 (v br s, 0.1H, *Z*-isomer), 10.27-10.83 (v br s, 0.6H, *E*-isomer), CON*H*OH]; ^{13}C NMR (50 MHz, DMSO-d_6) δ 21.7 (8,11-C), 29.4 (9,10-C), 35.1 (7,12-C), 40.0 (CONH*C*H_2CONHOH), 40.6 (*C*H_2CONHCH_2) 44.7 (2-C), 59.6 (6-C), 165.5, 166.9 (*C*ONHCH_2*C*ONHOH), 171.5, 176.9 (3,5-C). Anal. Calcd for $C_{14}H_{22}N_4O_5$: C, 51.52; H, 6.80; N, 17.17; Found: C, 51.24; H, 6.92; N, 17.32.

(*S*)-*N*-Hydroxy-2-[[[2-(2-methylpropyl)-3,5-dioxo-1,4-diazaspiro[5.6]dodec-4-yl]acetyl]amino] acetamide 28

The *O*-benzyl protected hydroxamate **68** was prepared from compound **57** (1.0 g, 2.72 mmol) according to the procedure described for the preparation of **58** (precursor for **18**). The crude product material (yellow viscous oil) was purified by column chromatography on silica gel eluting with AcOEt-*n*-hexane 1:1, 2:1 and 1:0 v/v in succession to give a white foamy solid, which strongly binds the elution solvents. Removal of the entrapped solvents as in **59** (precursor for **19**) afforded **68** as a glass solid (912 mg, 71%). This compound appears in the ^{1}H and ^{13}C NMR spectra as *E* and *Z* conformers (not assigned). ^{1}H NMR (400 MHz, $CDCl_3$) δ 0.90 (d, 3H, *J* = 4.4 Hz, CH(C*H*$_3$)CH_3), 0.94 (d, 3H, *J* = 4.5 Hz, CH(CH_3)C*H*$_3$), 1.17-2.04 (complex m, 15H, 1, 7, 8, 9, 10, 11, 12-H, CH_2C*H*(CH_3)$_2$), 2.16-2.30 (m, 1H, 12-H), 3.49-3.65 (br s, 1H, 2-H), [3.77 (d, 1.47H, *J* = 3.7 Hz), 4.09 (s, 0.43H), CONHCH_2CONHO], [4.36 (s), 4.42 (s), 2H, CH_2CONHCH_2], [4.78 (s), 4.82 (s), 2H, CONHOCH_2Ph], [6.70 (s, 0.2H), 7.16-7.42 (complex m, 5.7H), CH_2CON*H*CH_2, aromatic H], [8.90 (s, 0.2H), 9.81 (s, 0.73H), CON*H*OCH_2Ph]; ^{13}C NMR (50 MHz, $CDCl_3$) δ 21.1 (CH(*C*H_3)*C*H_3), 21.7 (8-C), 22.7 (11-C), 23.5 (*C*H(CH_3)*C*H_3), 24.5 (*C*H(CH_3)$_2$), 29.3, 29.6 (9, 10-C), 33.6 (7-C), 38.7 (12-C), 40.2 (*C*H_2CH(CH_3)$_2$), 41.4 (CONH*C*H_2CONHO), 42.0 (*C*H_2CONHCH_2), 51.7 (2-C), 61.1 (6-C), 78.5, 79.5 (CONHO*C*H_2Ph), 128.6, 128.8, 129.3 (2,3,4,5,6–aromatic C), 135.0 (1-aromatic C), 166.9, 168.5 (*C*ONHCH_2*C*ONHO), 174.5, 177.9 (3,5-C); ESI$^+$ MS: m/z 473.3 $[M+H]^+$.

Compound **68** (800 mg, 1.69 mmol) was taken in abs EtOH (76 mL) and hydrogenated over 10% Pd-C (96 mg) as described for the preparation of compound **18** from **58**. The crude hydrogenation product (off-white foamy solid) was chromatographed on silica gel column eluting with AcOEt and then AcOEt-MeOH 9:1 v/v to give a slightly off-yellow foamy solid. This material was dissolved in Et_2O and the clear solution was concentrated to low volume. The resulting precipitate was collected by vacuum filtration, washed with a small amount of Et_2O, and dried affording the title compound **28** as a white crystalline solid (605 mg,

93%). A recrystallized specimen was obtained upon dissolution of the product in AcOEt-MeOH 100:1 v/v and subsequent precipitation by addition of Et_2O; mp 146-148 °C (dec). ^1H NMR (400 MHz, DMSO-d_6) δ 0.88 (d, 3H, J = 6.4 Hz, CH(CH_3)CH_3), 0.92 (d, 3H, J = 6.6 Hz, CH(CH_3)CH_3), 1.31-1.81 (complex m, 12H, 7, 8, 9, 10, 11, 12-H, CH_2CH(CH_3)$_2$), 1.83-2.0 (complex m, 2H, 7-H, CH(CH_3)$_2$), 2.10-2.17 (m, 1H, 12-H), 2.57 (d, 1H, J = 12.0 Hz, 1-H), 3.47 (~t, 1H, J = 11.1 Hz, 2-H), [3.60 (d, 1.71H, J = 5.5 Hz, *E*-isomer), 3.92 (d, 0.23H, J = 4.1 Hz, *Z*-isomer), CONHCH_2CONHOH], 4.25 (s, 2H, CH_2CONHCH_2), [8.03 (s, 0.13H, *Z*-isomer), 8.26 (t, 0.87H, J = 5.4 Hz, *E*-isomer), CH_2CONHCH_2], 8.43-9.56 (v br s, 1H, CONHOH), [9.84-10.21 (v br s, 0.1H, *Z*-isomer), 10.25-10.89 (v br s, 0.5H, *E*-isomer, CONHOH]; ^{13}C NMR (50 MHz, DMSO-d_6) δ 21.1 (CH(CH_3)CH_3), 21.2 (8-C), 22.3 (11-C), 23.5 (CH(CH_3)CH_3), 23.9 (CH(CH_3)$_2$), 29.0, 29.2 (9, 10-C), 33.1 (7-C), 37.6 (12-C), 39.4 (CONHCH_2CONHOH, CH_2CH(CH_3)$_2$), 39.9 (CONHCH_2CONHOH), 41.2 (CH_2CONHCH_2), 51.1 (2-C), 60.2 (6-C), 165.6, 167.0 (CONHC$H_2$$C$ONHOH), 173.9, 177.3 (3,5-C); $[\alpha]^{22}_{589}$ = - 20.5 (c, 0.2, DMSO); ESI$^+$ MS: m/z 383.3, $[M+H]^+$. Anal Calcd for $C_{18}H_{30}N_4O_5$: C, 56.53; H, 7.91; N, 14.65; Found: C, 56.15; H, 8.02; N, 14.38.

Table S1. Elemental analysis data for the tested compounds synthesized in this study.

Compound	Molecular Formula	Calculated %			Found %		
		C	H	N	C	H	N
**18**	$C_{17}H_{24}N_4O_5$	56.03	6.64	15.38	55.86	6.71	15.19
**19**	$C_{21}H_{32}N_4O_5$	59.98	7.67	13.32	59.73	7.84	13.05
**20**	$C_{24}H_{30}N_4O_5$	63.42	6.65	12.33	63.73	6.89	12.14
**20.HCl**	$C_{24}H_{31}ClN_4O_5$	58.71	6.36	11.41	58.35	6.28	11.18
**21**	$C_{18}H_{26}N_4O_5$	57.13	6.93	14.81	57.34	7.05	14.59
**22**	$C_{22}H_{34}N_4O_5$	60.81	7.89	12.89	61.02	7.98	12.65
**23**	$C_{25}H_{32}N_4O_5$	64.08	6.88	11.96	64.25	6.97	11.77
**24**	$C_{15}H_{24}N_4O_5$	52.93	7.11	16.46	52.68	6.97	16.07
**25.HCl**	$C_{19}H_{33}ClN_4O_5$	52.71	7.68	12.94	52.36	7.79	12.68
**26**	$C_{16}H_{26}N_4O_5$	54.22	7.40	15.81	54.51	7.48	15.56
**27**	$C_{14}H_{22}N_4O_5$	51.52	6.80	17.17	51.24	6.92	17.32
**28**	$C_{18}H_{30}N_4O_5$	56.53	7.91	14.65	56.15	8.02	14.38

II. Experimental procedures for the HPLC quantitative determination of the final products

The quantitative determination of the samples was performed with a Thermo Finnigan HPLC system (ThermoFinnigan, San Jose, USA) consisting of a SpectraSystem P4000 pump, a SpectraSystem 1000 degasser, a SpectraSystem AS3000 autosampler, and a SpectralSystem UV2000 PDA detector controlled by a SpectralSystem controller. ChromQuest 4.1 software was used for the management of the data. For the HPLC-DAD phytochemical profiling a Supelco Analytical Discovery HS C18 (250 mm x 4.6 mm, 5.0 μm) column was used and the injection volume was 10 μl. The mobile phase consisted of 1% aqueous acetic acid (solvent A) and acetonitrile (solvent B). The elution conditions used were:

Time (min)	H_2O + 1% A.A.	ACN	Flow (mL/min)
0	95	5	1
17	0	100	1
37	0	100	1
38	95	5	1
50	95	5	1

The flow rate was set to 1 mL/min and the chromatograms were recorded at 260, 280 and 365 nm by monitoring spectra within a wavelength range of 200–700 nm, in room temperature.

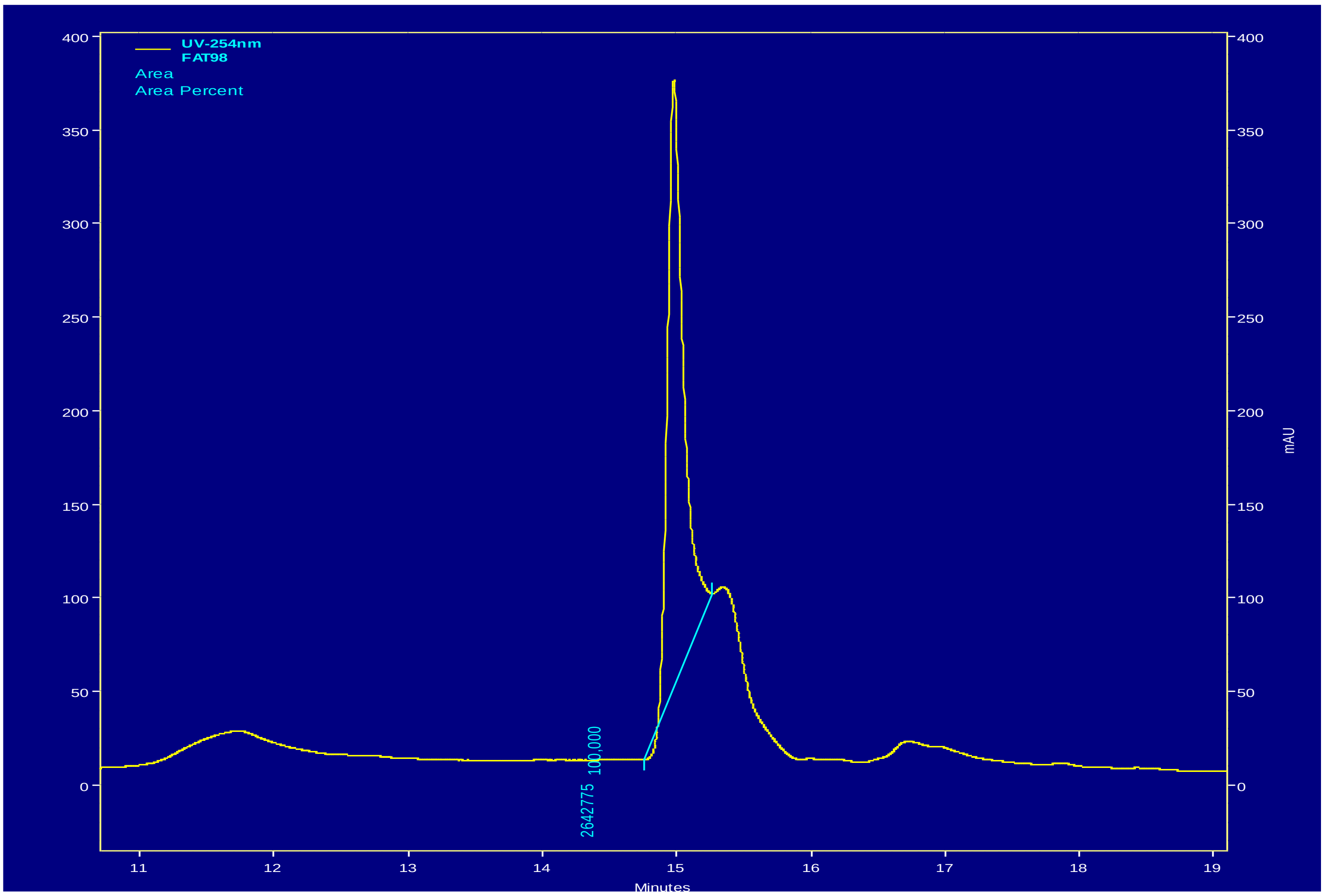

HPLC chromatograph for compound **20**, purity 96.2 %.

III. Experimental procedures for the computational studies

All compounds prior to the conformational search were minimised in Macromodel[4,5] (Schrödinger 2019-1 platform, Schrödinger, LLC, New York, NY, 2019) using the OPLS3e forcefield[6] in gas phase (ε = 1) and an RMS-gradient of 0.0001 kcal/mol Å.

The systematic search was conducted in Macromodel using the Systematic Pseudo Monte Carlo (SPMC) method.[7] The conformational search was conducted in OPLS3e and the potential treatment was set according to the dielectric standard of DMSO (ε = 46.68) to simulate the NMR measurements. The convergence threshold for minimization was set at 0.001 kcal/mol and the maximum iteration limit was set at 2500. Miscellaneous technical characteristics: Maximum number of steps 1000; steps per rotational bond 10000; energy window for saving structures 5.02 kcal/mol. The coordination scans for the dihedrals in Macromodel were setup with analogous settings using an increment of 5°.

Table S2: Values from dihedral coordination scans

**Cmp 18**	U (kcal/mol)	ΔU (kcal/mol)	Degrees
Umin 1_1	55,23		360
Umax 1_1	78,78	23,55	285
Umin 2_1	55,11		190
Umax 2_1	70,73	15,62	295
U Local min 1_1	60,68	18,10	180
U Local min 2_1	55,12	15,61	360
U comb min	51,62		360 (1_1), 5 (2_1)
**Cmp 21**			
	U (kcal/mol)	ΔU (kcal/mol)	Degrees
Umin 1_1	67,06		360
Umax 1_1	87,00	19,94	270
Umin 2_1	64,22		360
Umax 2_1	79,34	15,12	110

U Local min 1_1	68,12	18,88	180
U Local min 2_1	64,69	14,65	170
U comb min	64,24		0 (1_1), 360 (2_1)

Compound **18**_mmod_drive_1-out

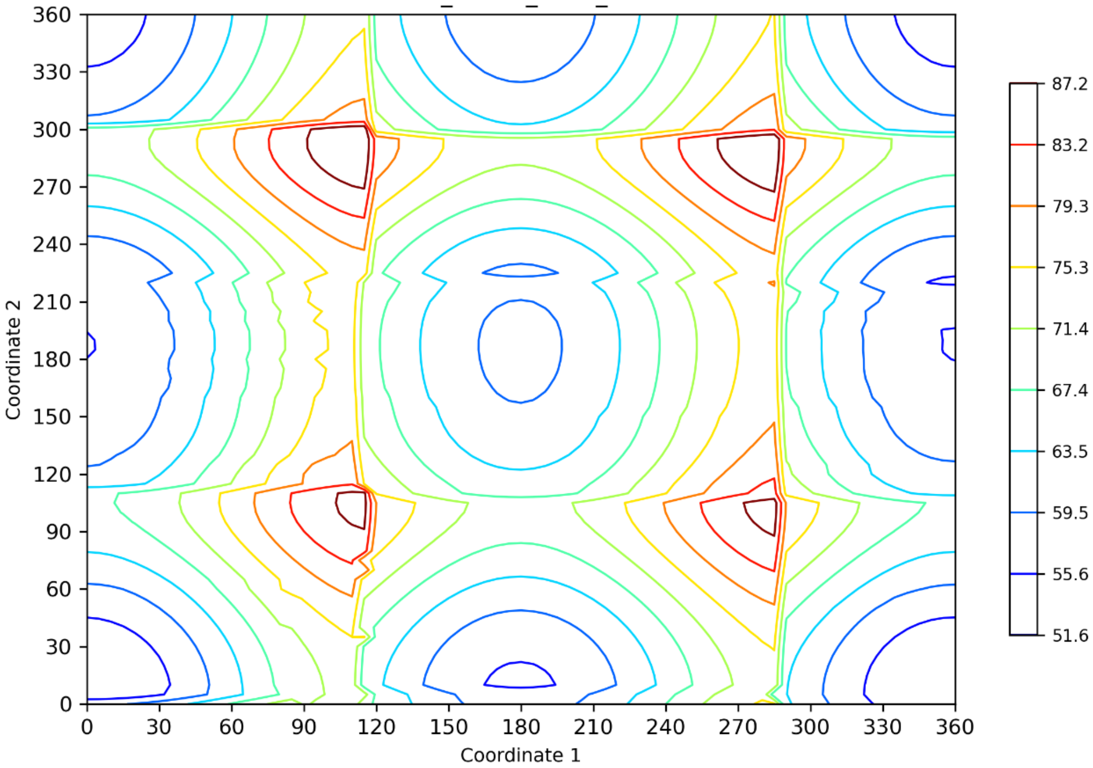

Figure S1: 2D plot of simultaneous coordination scan of dihedral angles C7-C8-N9-H (Coordinate 1) and C10-C11-N12-H (Coordinate 2) in **18**. Output: 5330 structures. Energy: kcal/mol. Coordinate: degrees

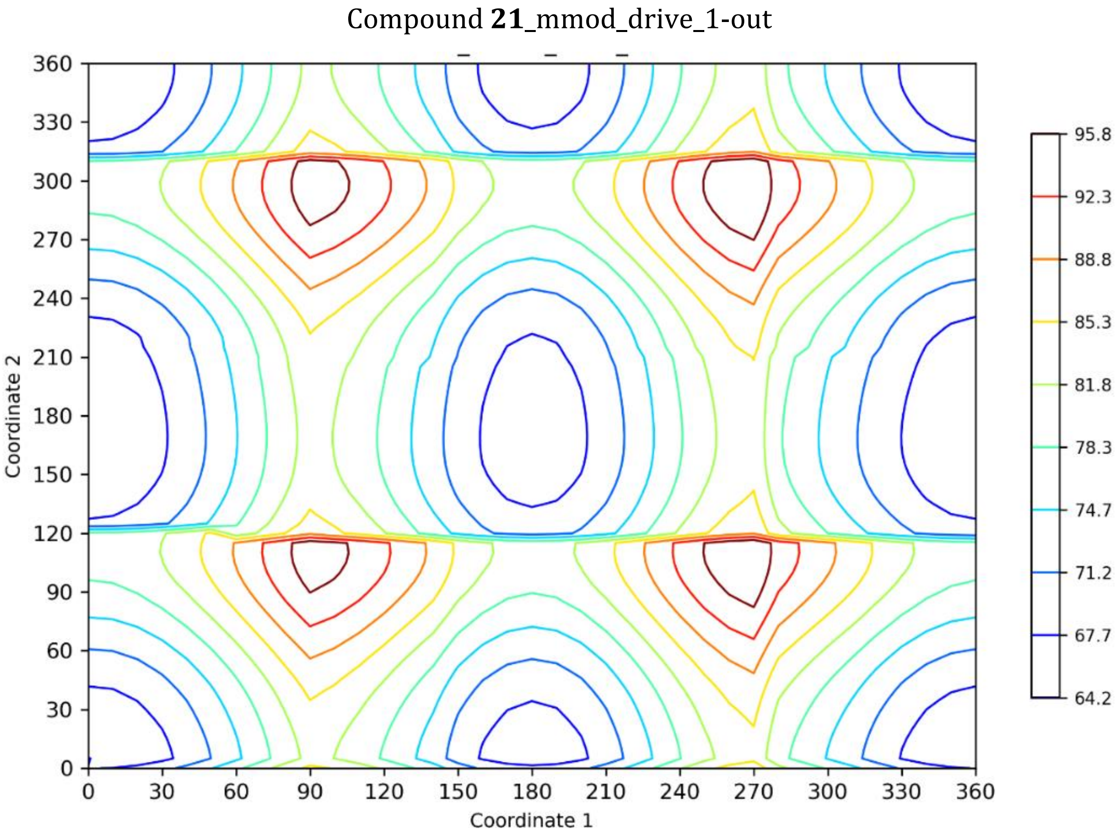

Figure S2: 2D plot of simultaneous coordination scan of dihedral angles C7-C8-N9-C9 (Coordinate 1) and C10-C11-N12-H (Coordinate 2) in **21**. Output: 2702 structures. Energy: kcal/mol. Coordinate: degrees.

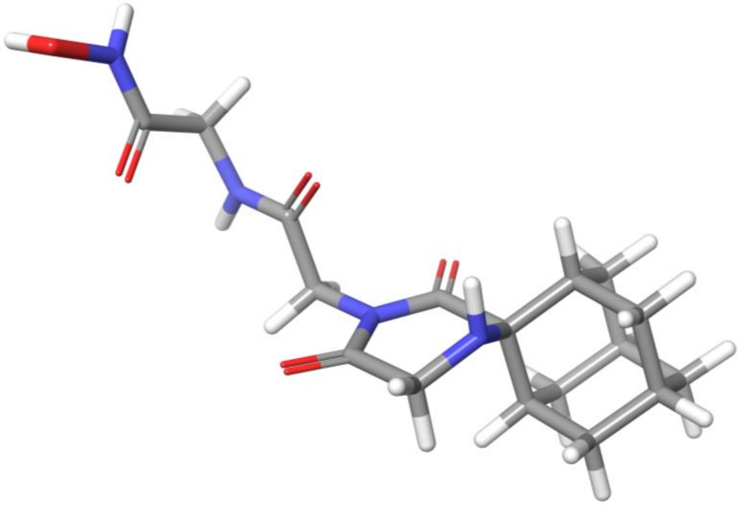

Figure S3: Minimized structure of **18**

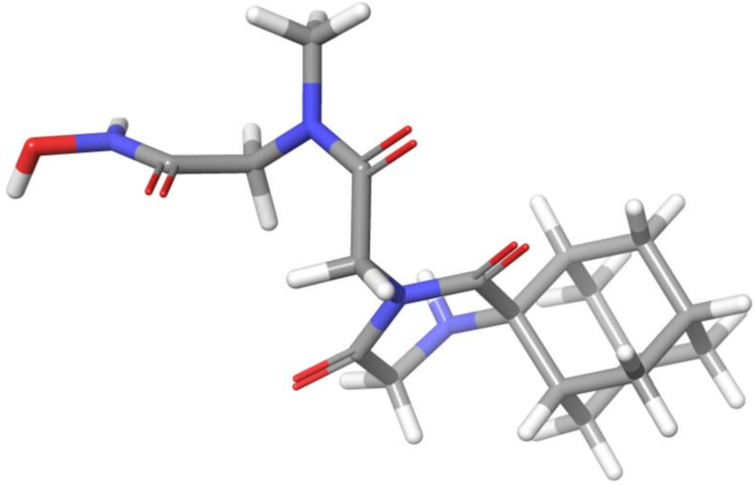

Figure S4: Minimized structure of **21**

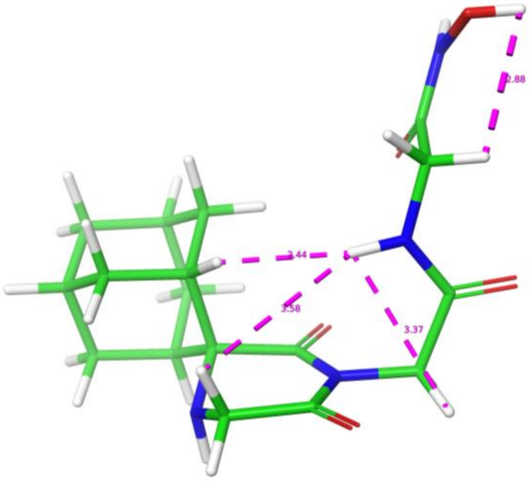

Figure S5: Cmp **18** – *ZE* conformer of minimum energy (29/167).

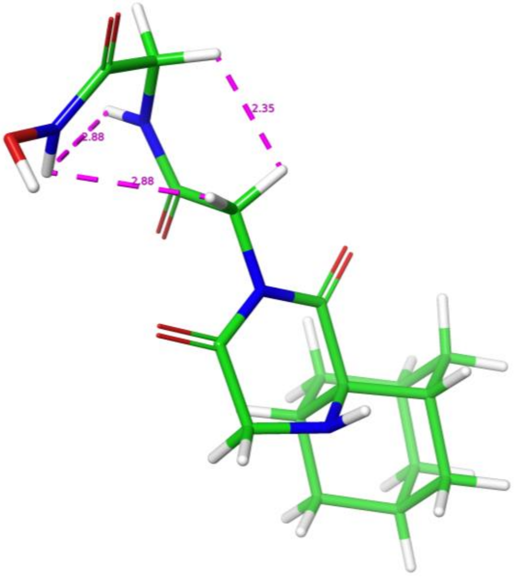

Figure S6: Cmp **18** – *EZ* conformer of minimum energy (90/167).

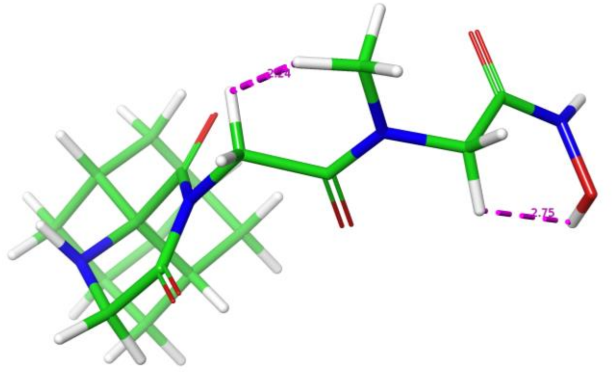

Figure S7: Cmp **21** – *ZE* conformer of minimum energy (24/315).

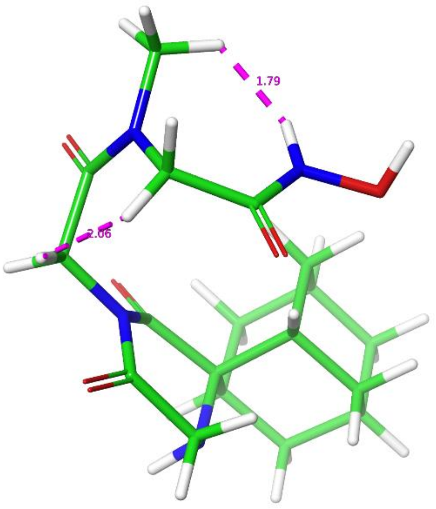

Figure S8: Cmp **21** – *EZ* conformer of minimum energy (26/315).

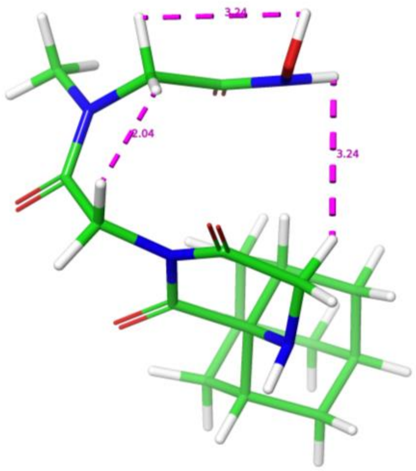

Figure S9: Cmp **21** – *EE* conformer of minimum energy (55/315).

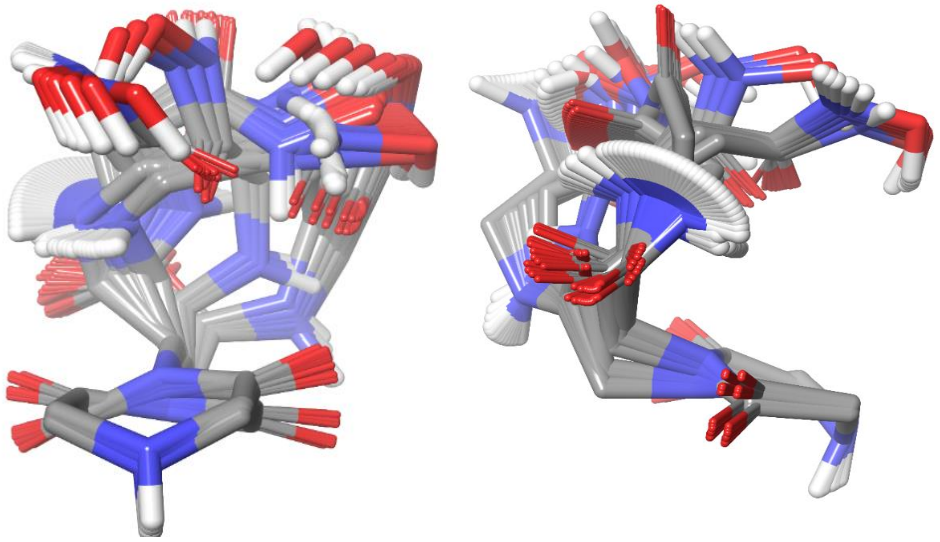

Figure S10: Superimposed structures of coordination scan of dihedral 1_1 in **18** (74 structures – Reference: minimized structure, heterocycle and adamantane cage). Adamantane cage is not shown for clarity.

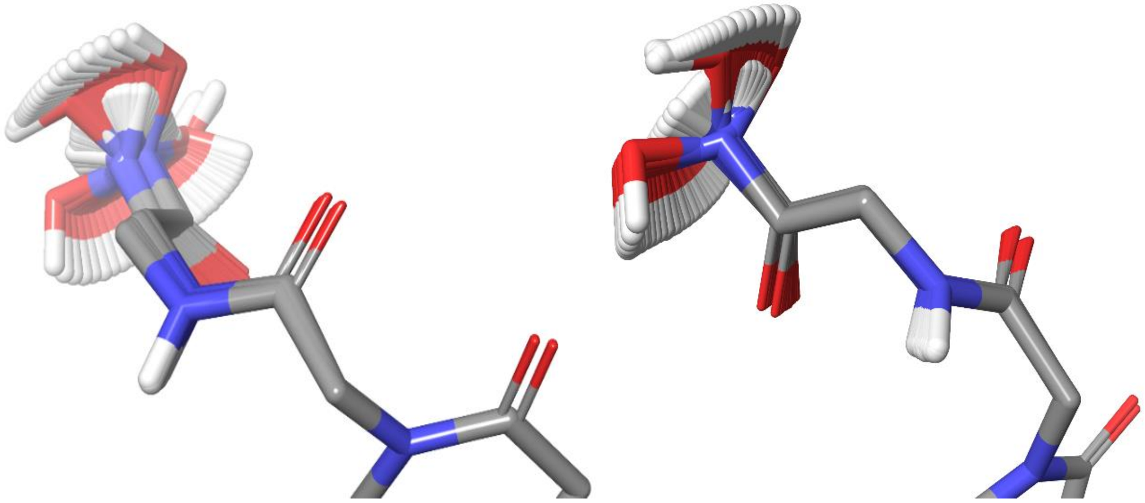

Figure S11: Superimposed structures of coordination scan of dihedral 2_1 in **18** (74 structures – Reference: minimized structure, heterocycle and adamantane cage).

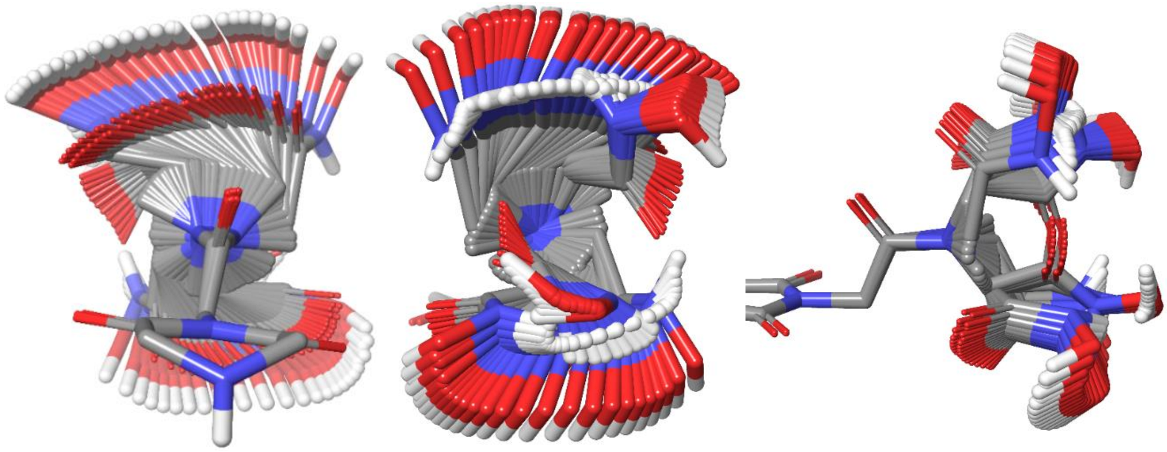

Figure S12: Superimposed structures of coordination scan of dihedral 1_1 in **21** (74 structures – Reference: minimized structure, heterocycle and adamantane cage). Adamantane cage is not shown for clarity.

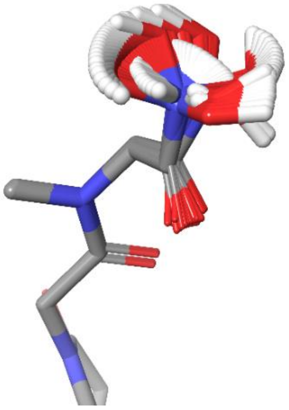

Figure S13: Superimposed structures of coordination scan of dihedral 2_1 in **21** (74 structures – Reference: minimized structure, heterocycle and adamantane cage).

IV. Experimental procedures for the biological evaluation of the compounds

Trypanocidal Activity

Growth inhibition analysis was carried out using bloodstream form *T. b. brucei* (strain 221) cultured at 37°C, as previously described.[8] Briefly, experiments were performed using 96-well microtiter plates (200 μL volumes), with each assay initiated with 2.5 x10^4 parasites mL^{-1}, and test compounds then added at 7 concentrations in a range that had been established in preliminary experiments to encompass both EC50 and EC90 values. Plates were incubated for 48 hours, resazurin (20 μL at 0.125 mg mL^{-1}) was then added, and the plates re-incubated for 16 hours. Fluorescence was determined using a BMG FLUOstar Omega plate reader (excitation 545 nm, emission 590 nm), and the data analysed using GraphPad Prism 9.0 software. The data are expressed as IC_{50}/IC_{90} ± SD, and are the average of 3 independent replicates.

To determine cytotoxicity, L6 cells (a rat myoblast line) were seeded at $1x10^4$ mL^{-1} into 96-well microtiter plates (200 µL of growth medium), at a range of compound concentrations. After 6 days incubation at 37°C, 20 µL resazurin (0.125 mg mL^{-1}) was added to each well. The plates were incubated for 6 hours, and fluorescence determined by plate reader, as above.

V. References

(1) Fytas, C.; Zoidis, G.; Fytas, G. A Facile and Effective Synthesis of Lipophilic 2,6-Diketopiperazine Analogues. *Tetrahedron* **2008**, *64* (28), 6749–6754. https://doi.org/10.1016/j.tet.2008.05.005.
(2) Zoidis, G.; Tsotinis, A.; Tsatsaroni, A.; Taylor, M. C.; Kelly, J. M.; Efstathiou, A.; Smirlis, D.; Fytas, G. Lipophilic Conformationally Constrained Spiro Carbocyclic 2,6-Diketopiperazine-1-Acetohydroxamic Acid Analogues as Trypanocidal and Leishmanicidal Agents: An Extended SAR Study. *Chem Biol Drug Des* **2018**, *91* (2), 408–421. https://doi.org/10.1111/cbdd.13088.
(3) Fytas, C.; Zoidis, G.; Tzoutzas, N.; Taylor, M. C.; Fytas, G.; Kelly, J. M. Novel Lipophilic Acetohydroxamic Acid Derivatives Based on Conformationally Constrained Spiro Carbocyclic 2,6-Diketopiperazine Scaffolds with Potent Trypanocidal Activity. *J. Med. Chem.* **2011**, *54* (14), 5250–5254. https://doi.org/10.1021/jm200217m.
(4) Mohamadi, F.; Richards, N. G. J.; Guida, W. C.; Liskamp, R.; Lipton, M.; Caufield, C.; Chang, G.; Hendrickson, T.; Still, W. C. Macromodel—an Integrated Software System for Modeling Organic and Bioorganic Molecules Using Molecular Mechanics. *Journal of Computational Chemistry* **1990**, *11* (4), 440–467. https://doi.org/10.1002/jcc.540110405.
(5) Watts, K. S.; Dalal, P.; Tebben, A. J.; Cheney, D. L.; Shelley, J. C. Macrocycle Conformational Sampling with MacroModel. *J. Chem. Inf. Model.* **2014**, *54* (10), 2680–2696. https://doi.org/10.1021/ci5001696.
(6) Roos, K.; Wu, C.; Damm, W.; Reboul, M.; Stevenson, J. M.; Lu, C.; Dahlgren, M. K.; Mondal, S.; Chen, W.; Wang, L.; Abel, R.; Friesner, R. A.; Harder, E. D. OPLS3e: Extending Force Field Coverage for Drug-Like Small Molecules. *J. Chem. Theory Comput.* **2019**, *15* (3), 1863–1874. https://doi.org/10.1021/acs.jctc.8b01026.
(7) Goodman, J. M.; Still, W. C. An Unbounded Systematic Search of Conformational Space. *Journal of Computational Chemistry* **1991**, *12* (9), 1110–1117. https://doi.org/10.1002/jcc.540120908.
(8) Giannakopoulou, E.; Pardali, V.; Frakolaki, E.; Siozos, V.; Myrianthopoulos, V.; Mikros, E.; Taylor, M. C.; Kelly, J. M.; Vassilaki, N.; Zoidis, G. Scaffold Hybridization Strategy towards Potent Hydroxamate-Based Inhibitors of *Flaviviridae* Viruses and *Trypanosoma* Species. *Med. Chem. Commun.* **2019**, *10* (6), 991–1006. https://doi.org/10.1039/C9MD00200F.

VI. Table S3: Selection of calculated Drug-like properties for the tested compounds.

Cmpd	dipole	SASA	FOSA	FISA	PISA	volume	QPpolrz	QPlogPo/w	QPlogS	CIQPlogS	QPPCaco	QPlogBB	QPPMDCK	QPlogKp	% Human Oral Absorption	SAamideO	PSA
**18**	4.838	613.419	373.625	239.795	0	1091.254	34.307	-1.547	-0.276	-0.591	4.489	-1.629	5.069	-7.521	29.563	58.625	162.732
**19**	3.255	688.724	498.86	189.864	0	1264.056	39.939	-0.215	-1.334	-1.622	15.566	-1.338	16.47	-6.409	47.022	50.268	146.502
**20**	6.503	724.614	360.075	183.136	181.403	1334.642	44.496	0.447	-1.625	-2.625	15.61	-1.325	19.304	-5.646	50.921	58.13	147.238
**21**	6.493	618.023	411.768	206.255	0	1126.797	35.73	-1.521	-0.71	-0.411	13.655	-1.255	11.186	-6.903	38.358	37.89	150.523
**22**	6.637	710.175	538.135	172.041	0	1335.193	42.786	-0.105	-1.835	-1.44	30.663	-1.142	25.083	-6.081	52.936	34.513	142.767
**23**	6.649	721.184	361.585	189.732	169.867	1371.397	45.857	0.189	-1.867	-2.443	19.339	-1.306	16.521	-5.808	51.075	38.586	145.653
**24**	4.548	602.431	365.196	237.235	0	1065.25	33.267	-1.675	-0.467	0.243	5.75	-1.595	5.384	-7.474	30.736	48.167	164.245
**25**	10.695	692.403	476.814	215.589	0	1265.737	40.006	-0.389	-1.036	-0.765	7.298	-1.612	8.975	-6.883	40.119	60.953	154.932
**26**	6.311	624.125	404.703	219.422	0	1117.351	35.352	-1.687	-0.591	0.424	9.151	-1.441	8.198	-7.146	34.273	44.04	153.685
**27**	4.594	583.878	345.757	238.121	0	1022.102	31.54	-1.92	-0.204	0.421	5.622	-1.587	5.273	-7.491	29.122	48.346	164.844
**28**	12.892	681.879	468.874	213.005	0	1227.01	38.456	-0.606	-0.752	-0.573	7.205	-1.609	9.539	-6.836	38.749	64.728	157.184

The above table is a selection of properties modelled with QikProp – Schrödinger. A full detailed report of all QikProp models and predicted values can be found in the output text file of the program, which is included as a standalone file. A brief description of all QikProp calculated properties and descriptors can be found below.

Properties/descriptors calculated by QikProp

#stars Number of property or descriptor values that fall outside the 95% range of similar values for known drugs.
#amine Number of non-conjugated amine groups
#amidine Number of non-conjugated amine groups

#acid Number of carboxylic acid groups
#amide Number of non-conjugated amide groups.
#rotor Number of non-trivial (not CX3), non-hindered (not alkene, amide, small ring) rotatable bonds
#rtvFG Number of reactive functional groups
CNS Predicted central nervous system activity
mol_MW Molecular weight of the molecule
dipole Computed dipole moment of the molecule (D)
SASA Total solvent accessible surface area (SASA) in square angstroms using a probe with a 1.4 Å radius
FOSA Hydrophobic component of the SASA (saturated carbon and attached hydrogen)
FISA Hydrophilic component of the SASA (SASA on N, O, and H on heteroatoms)
PISA π (carbon and attached hydrogen) component of the SASA
WPSA Weakly polar component of the SASA (halogens, P, and S)
volume Total solvent-accessible volume in cubic angstroms using a probe with a 1.4 Å radius
donorHB Estimated number of hydrogen bonds that would be donated by the solute to water molecules in an aqueous solution
accptHB Estimated number of hydrogen bonds that would be accepted by the solute from water molecules in an aqueous solution
dip^2/V Square of the dipole moment divided by the molecular volume
ACxDN^.5/SA Index of cohesive interaction in solids
glob Globularity descriptor
QPpolrz Predicted polarizability in cubic angstroms
QPlogPC16 Predicted hexadecane/gas partition coefficient
QPlogPoct Predicted octanol/gas partition coefficient
QPlogPw Predicted water/gas partition coefficient
QPlogPo/w Predicted octanol/water partition coefficient
QPlogS Predicted aqueous solubility
CIQPlogS Conformation-independent predicted aqueous solubility
QPlogHERG Predicted IC50 value for blockage of HERG K+ channels
QPPCaco Predicted apparent Caco-2 cell permeability in nm/sec
QPlogBB Predicted brain/blood partition coefficient
QPPMDCK Predicted apparent Madin-Darby canine kidney cell permeability in nm/sec
QPlogKp Predicted skin permeability
IP(eV) PM3 calculated ionization potential (negative of HOMO energy)
EA(eV) PM3 calculated electron affinity (negative of LUMO energy)
#metab Number of likely metabolic reactions
QPlogKhsa Prediction of binding to human serum albumin

HumanOralAbsorption Predicted qualitative human oral absorption
PercentHumanOralAbsorption Predicted human oral absorption on 0 to 100% scale
SAfluorine Solvent-accessible surface area of fluorine atoms
SAamideO Solvent-accessible surface area of amide oxygen atoms
PSA Van der Waals surface area of polar nitrogen and oxygen atoms
#NandO Number of nitrogen and oxygen atoms
RuleOfFive Number of violations of Lipinski's rule of five
RuleOfThree Number of violations of Jorgensen's rule of three
#ringatoms Number of atoms in a ring
#in34 Number of atoms in 3- or 4-membered rings
#in56 Number of atoms in 5- or 6-membered rings
#noncon number of ring atoms not able to form conjugated aromatic systems (e.g. sp3 C)
#nonHatm Number of heavy atoms (nonhydrogen atoms)

Materials & Methods:
The 3D structures were generated in Maestro from the respective SMILES strings, and chiral centers were set accordingly where necessary. The structures were minimized in neutral state using Macromodel with the settings previously described. The minimized structures were used as input in QikProp under default settings.

Schrödinger Release 2024-1: QikProp v7.8, Schrödinger, LLC, New York, NY, 2024.

VII. Copies of NMR spectra

^{1}H NMR of **18** (400.13 MHz, DMSO-d_6)

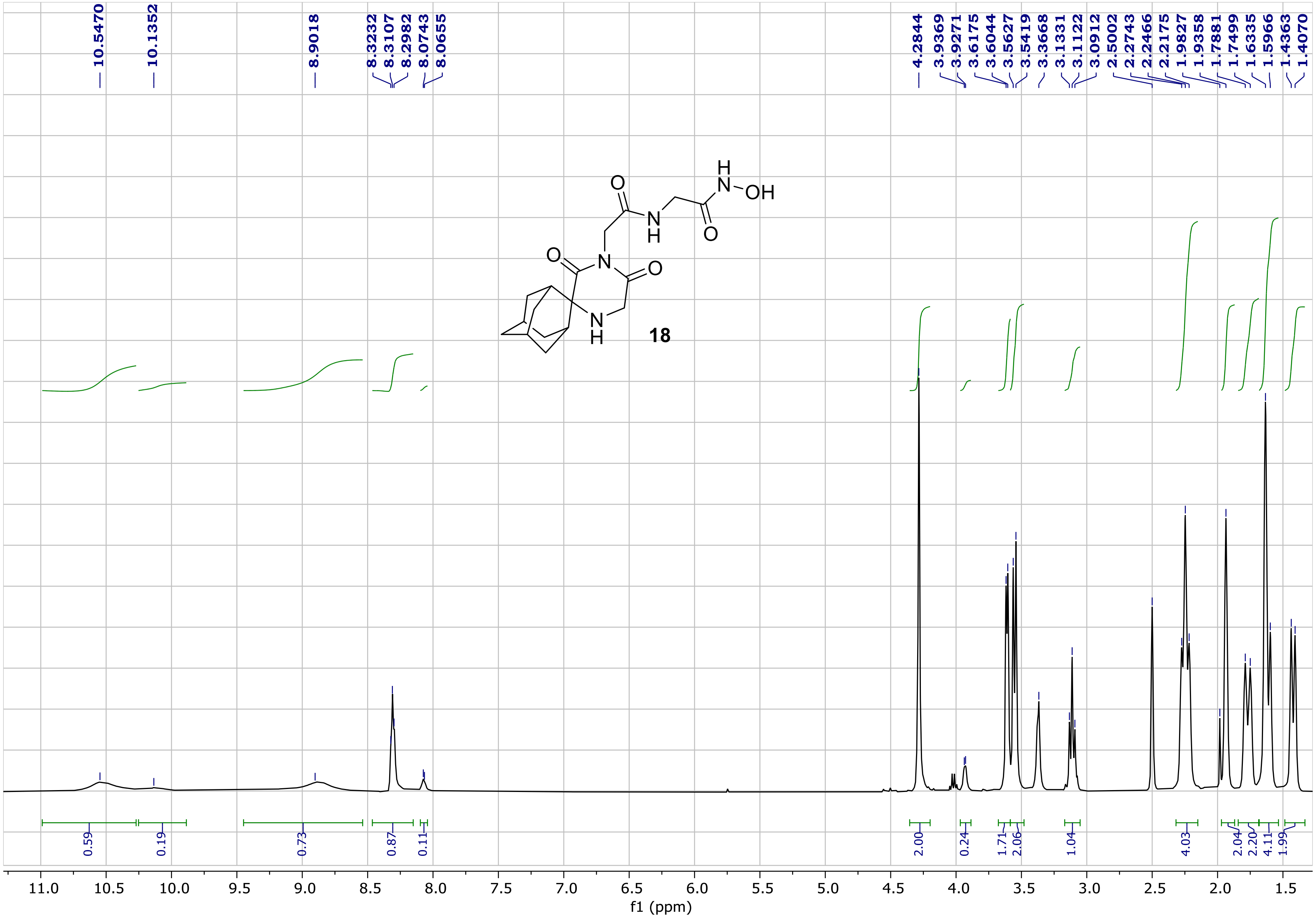

^{13}C NMR of **18** (50.32 MHz, DMSO-d_6)

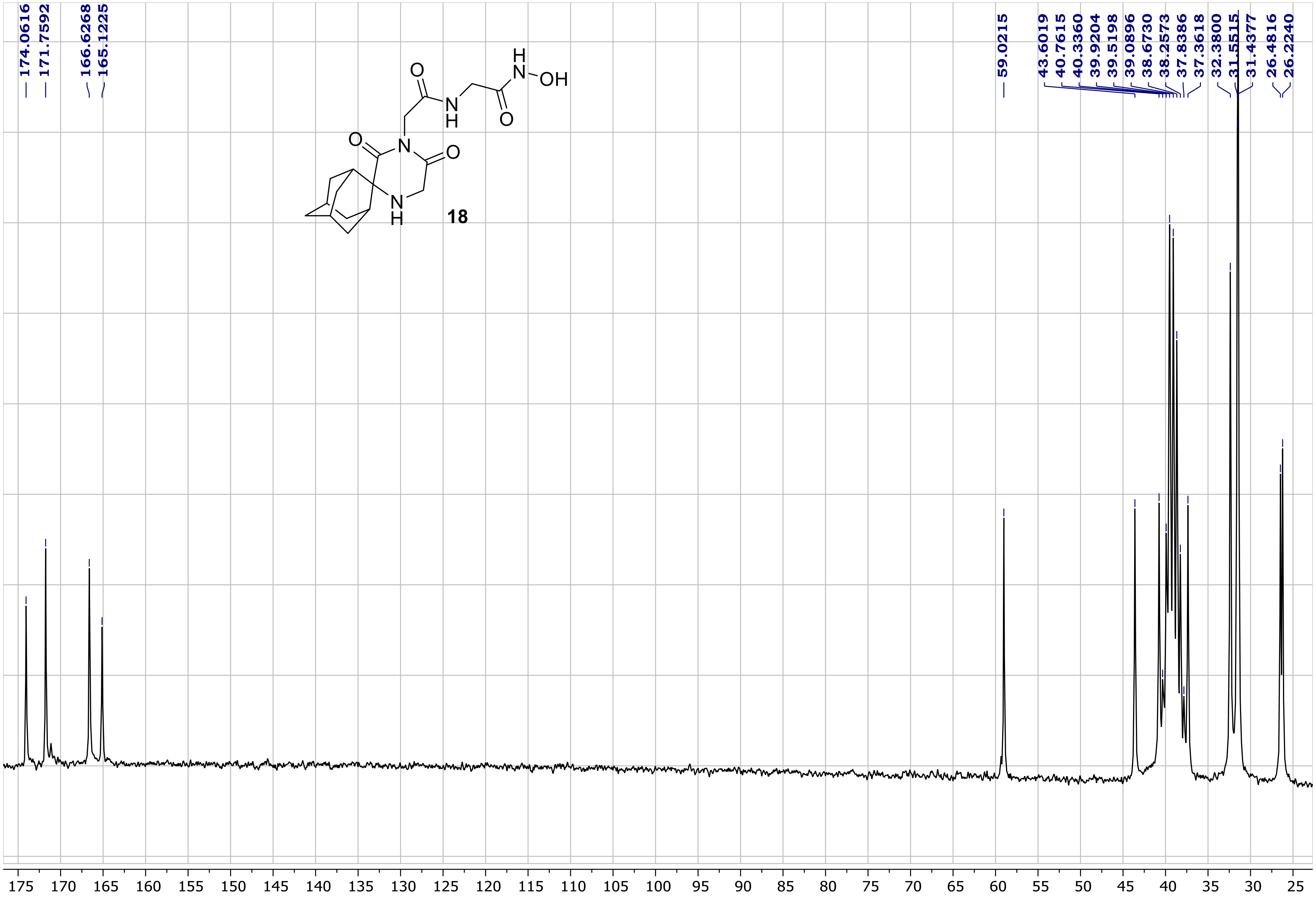

COSY NMR of **18** (400.13 MHz, DMSO-d_6)

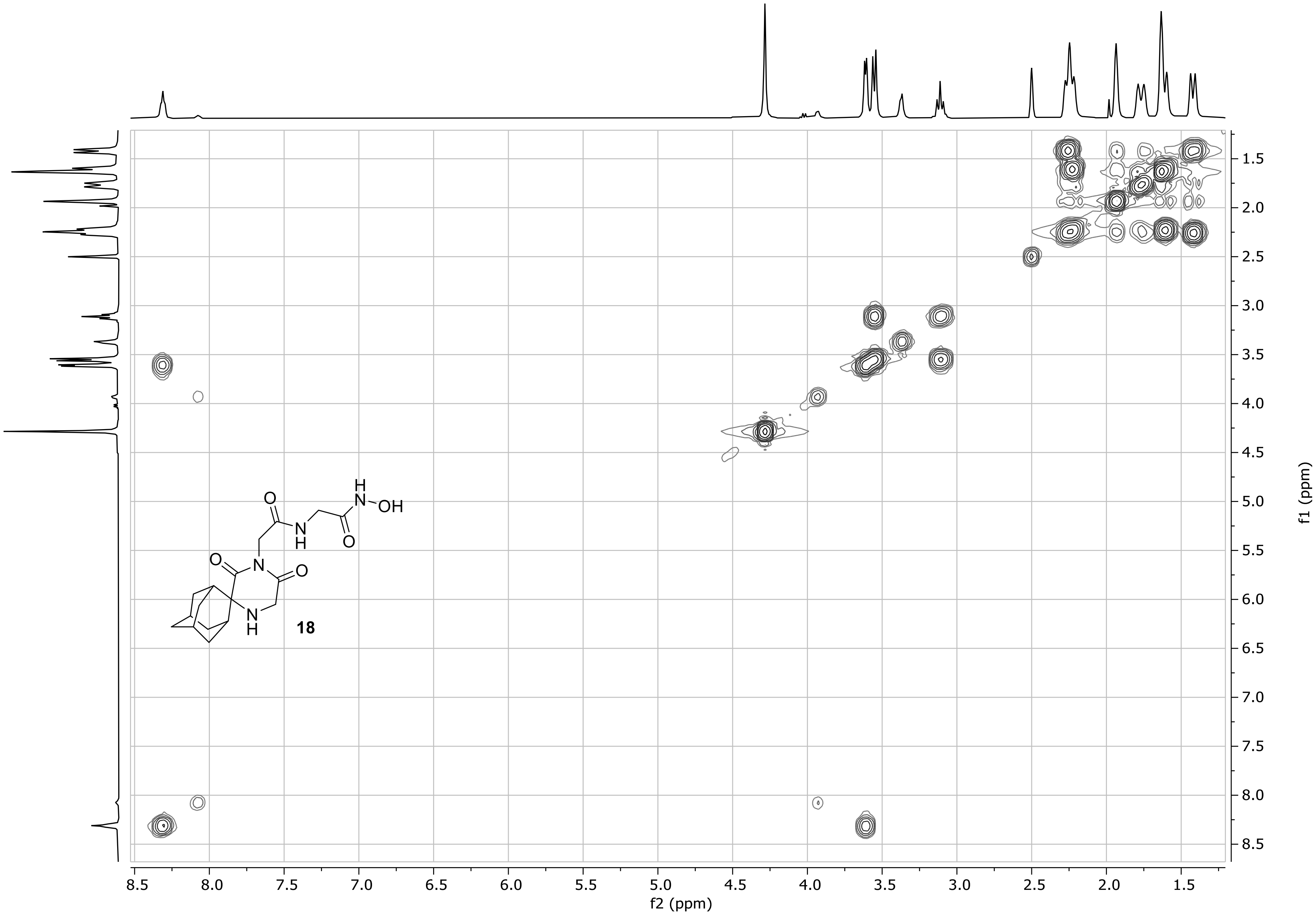

18

HSQC NMR of **18** (400.13 MHz, DMSO-d_6)

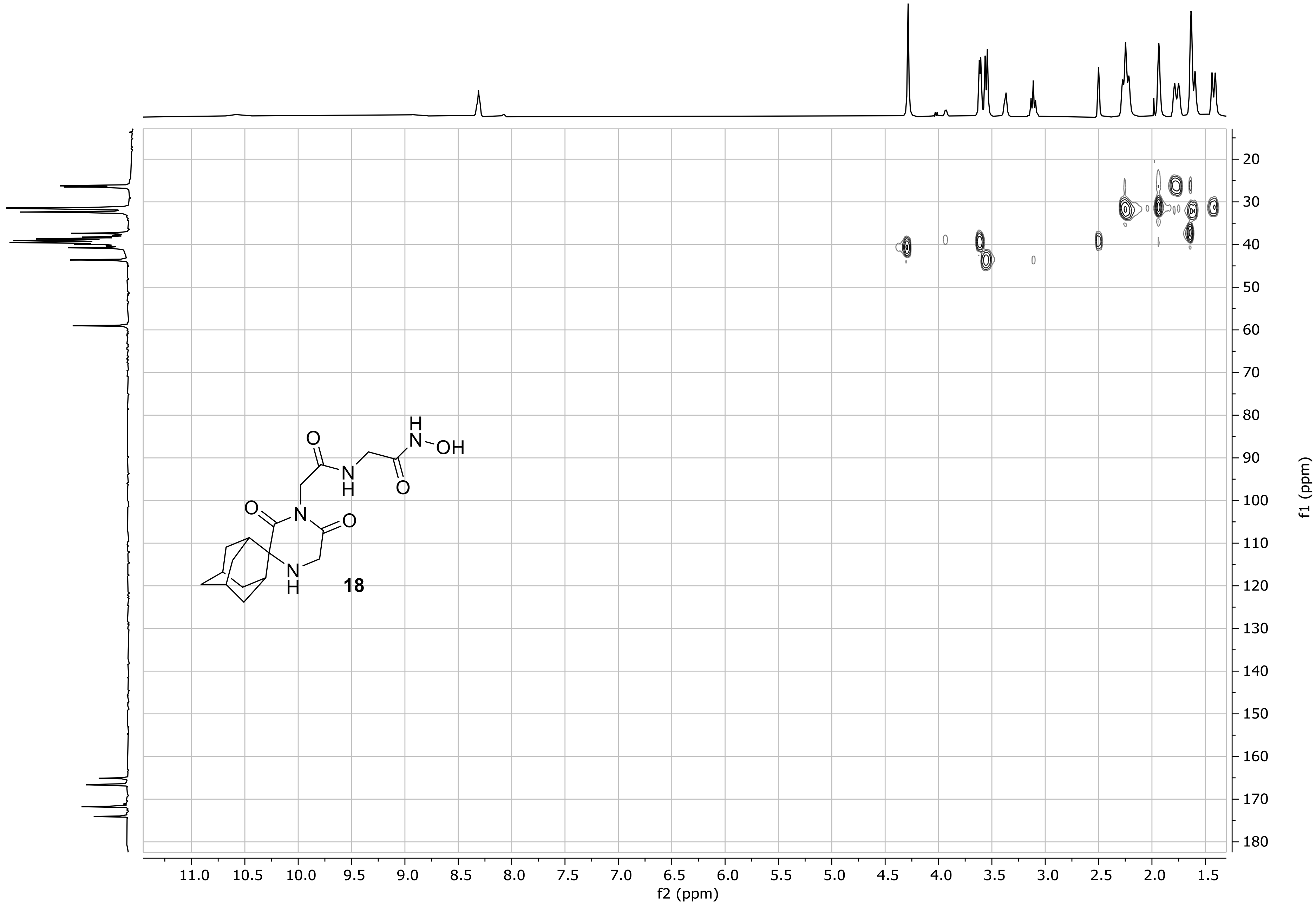

DEPT NMR of **18** (50.32 MHz, DMSO-d_6)

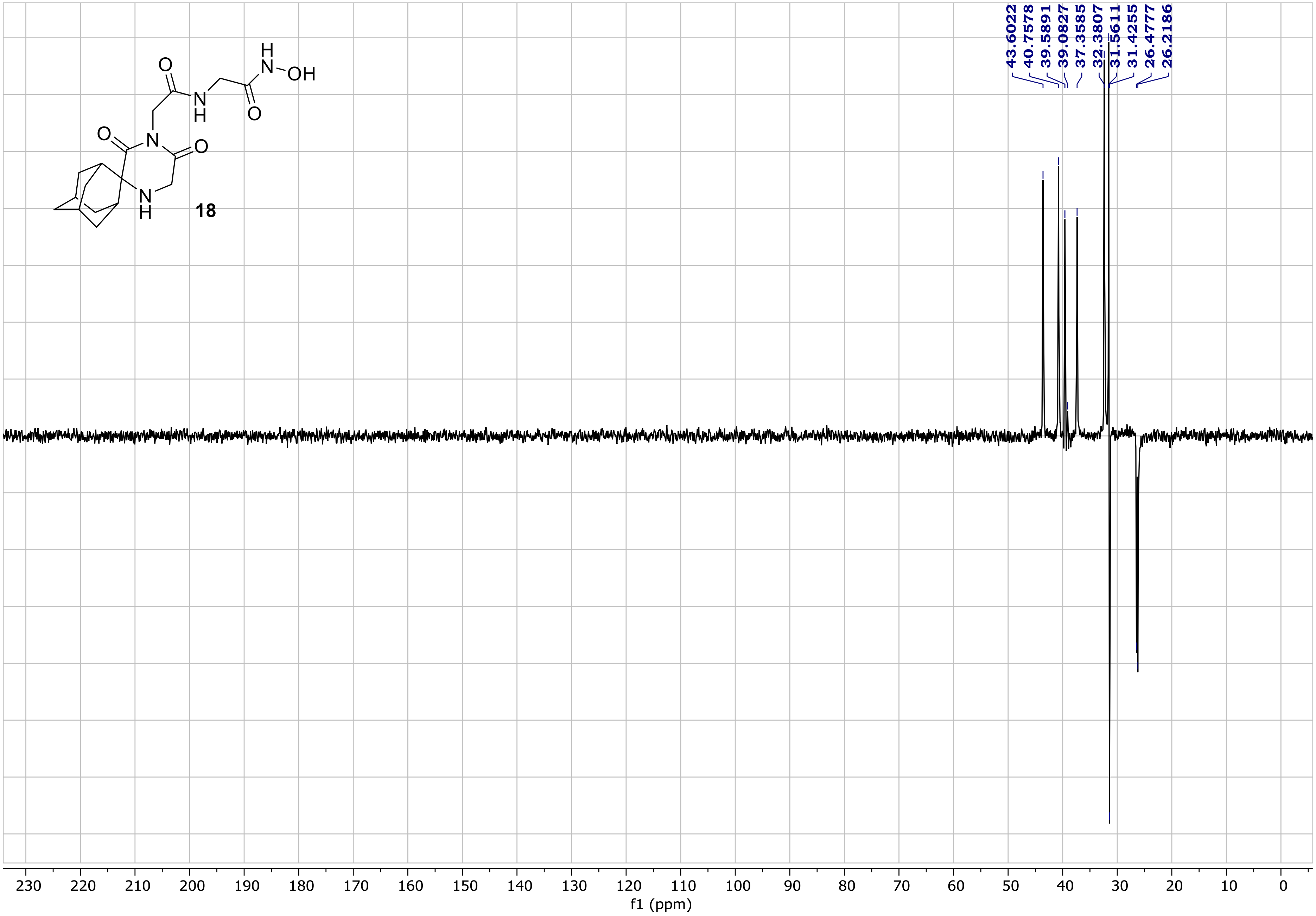

^{1}H NMR of **19** (600.11 MHz, DMSO-d_6)

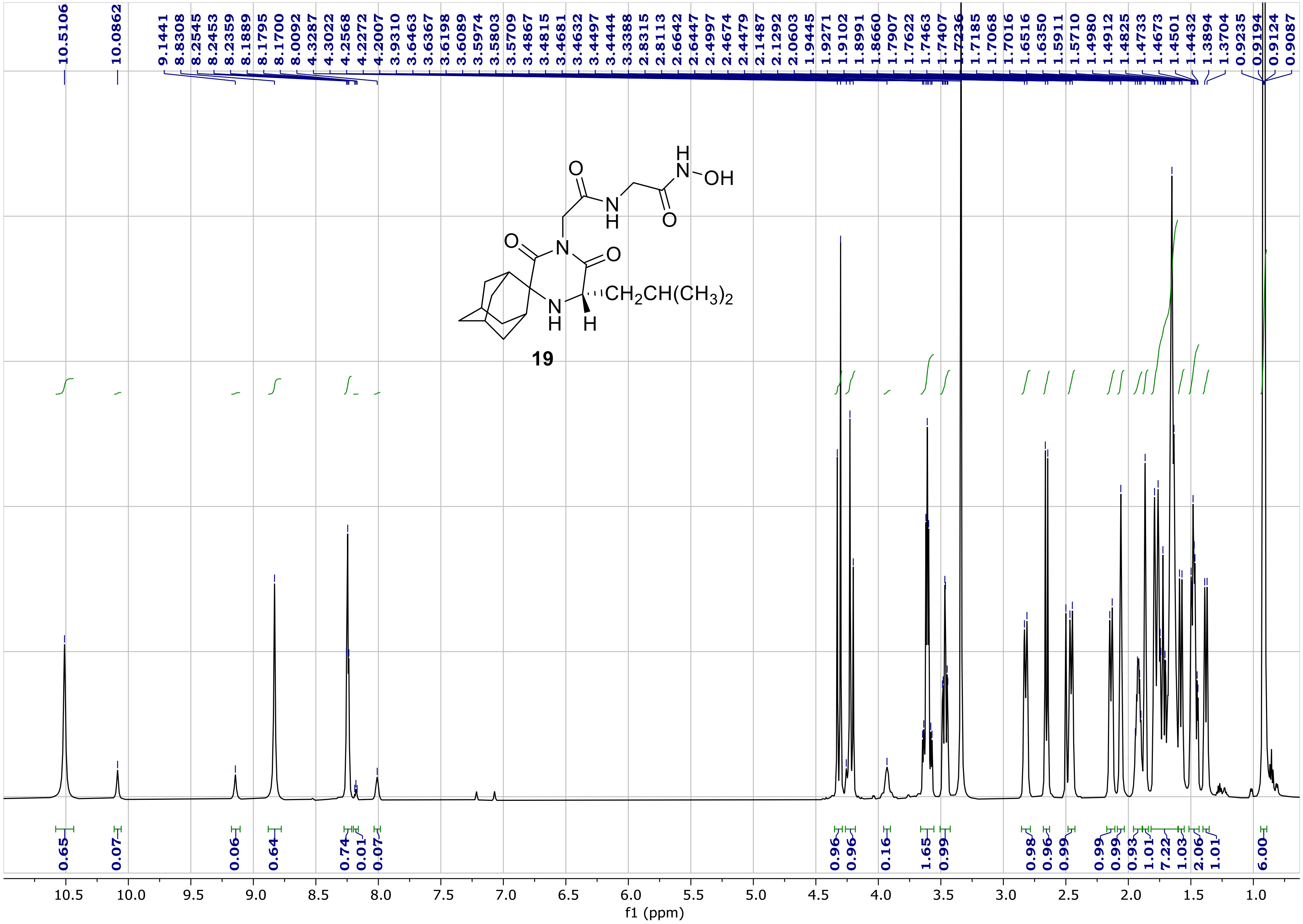

^{13}C NMR of **19** (150.9 MHz, DMSO-d_6)

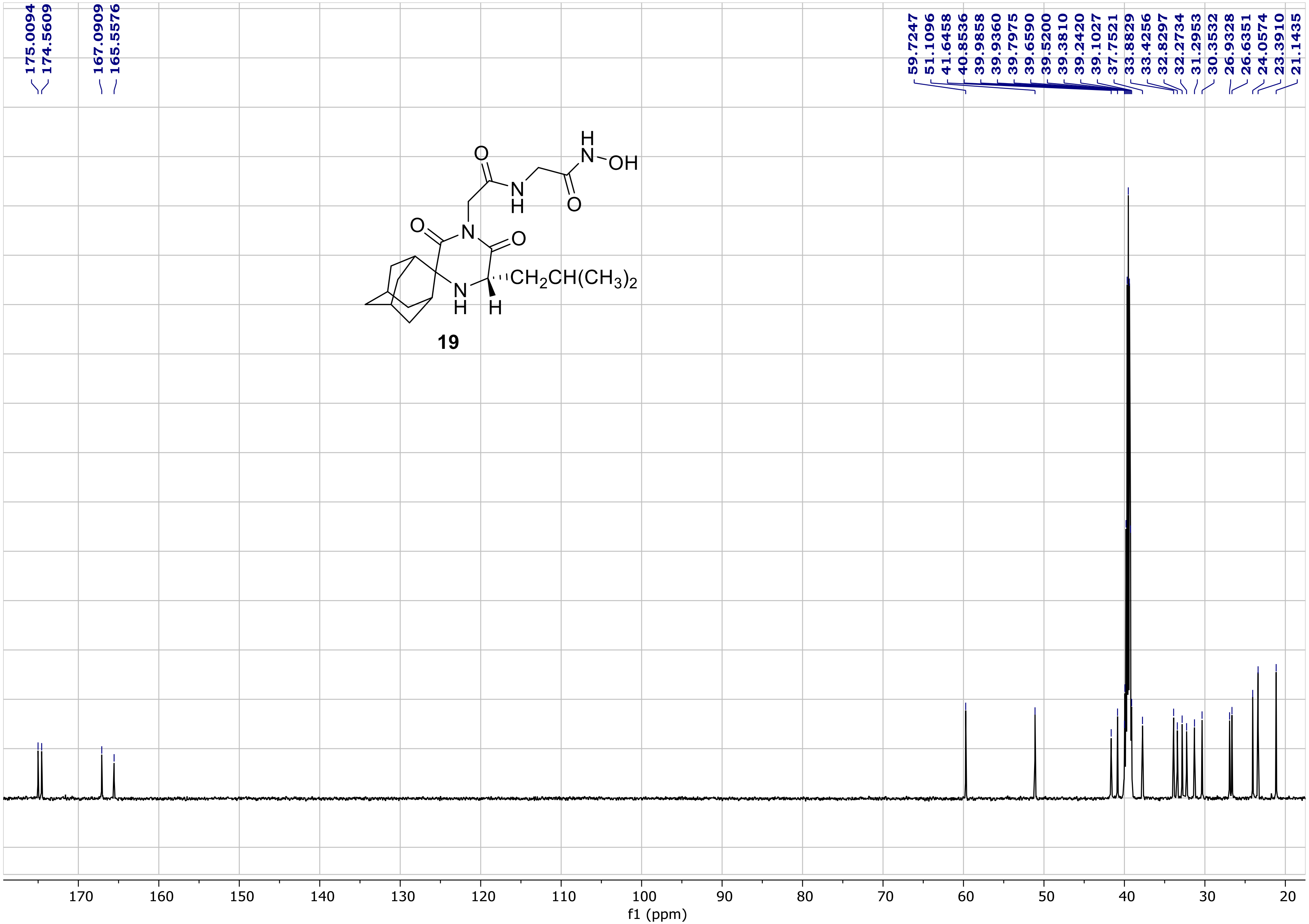

COSY NMR of **19** (600.11 MHz, DMSO-d_6)

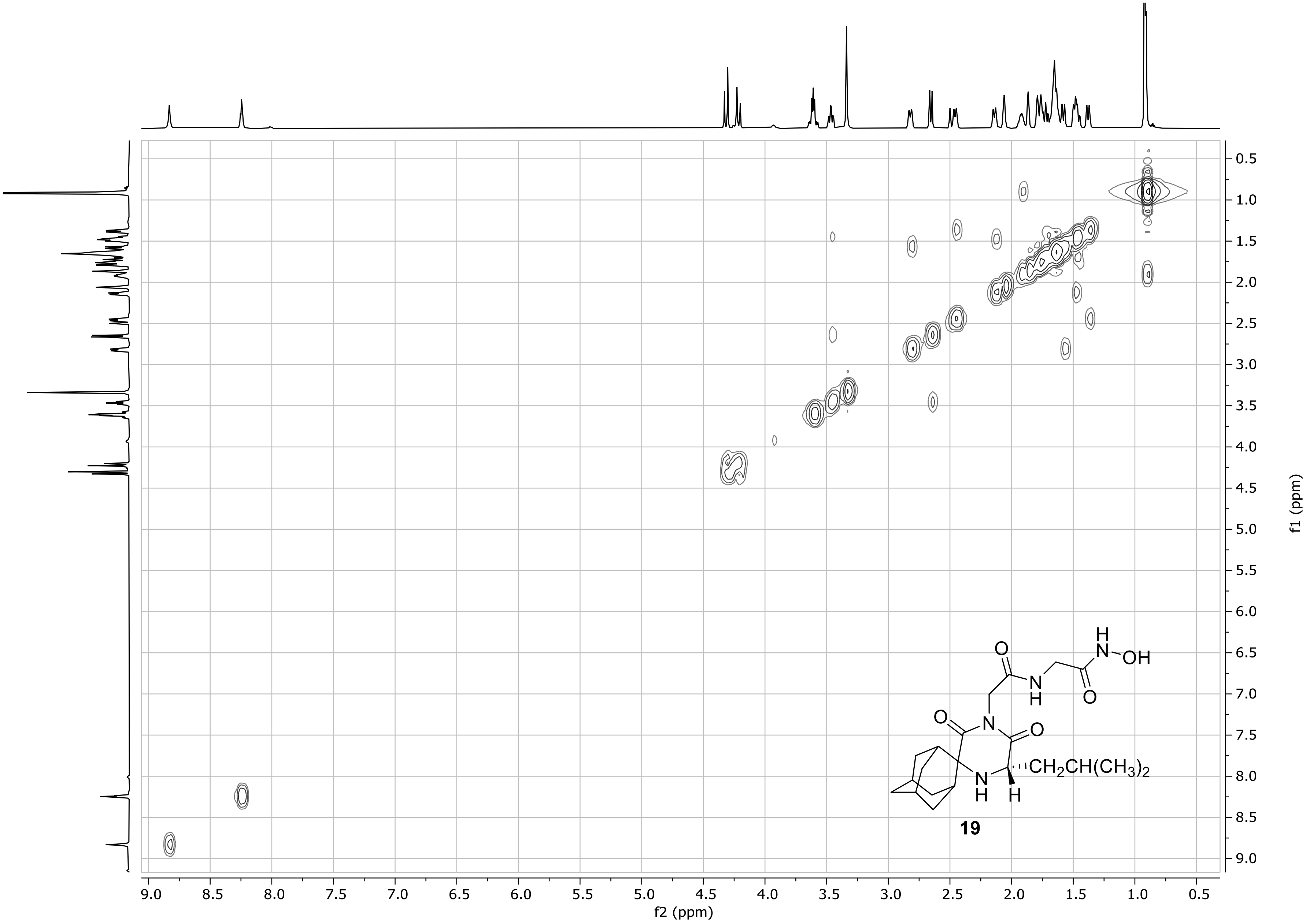

HSQC-DEPT NMR of **19** (600.11 MHz, DMSO-d_6)

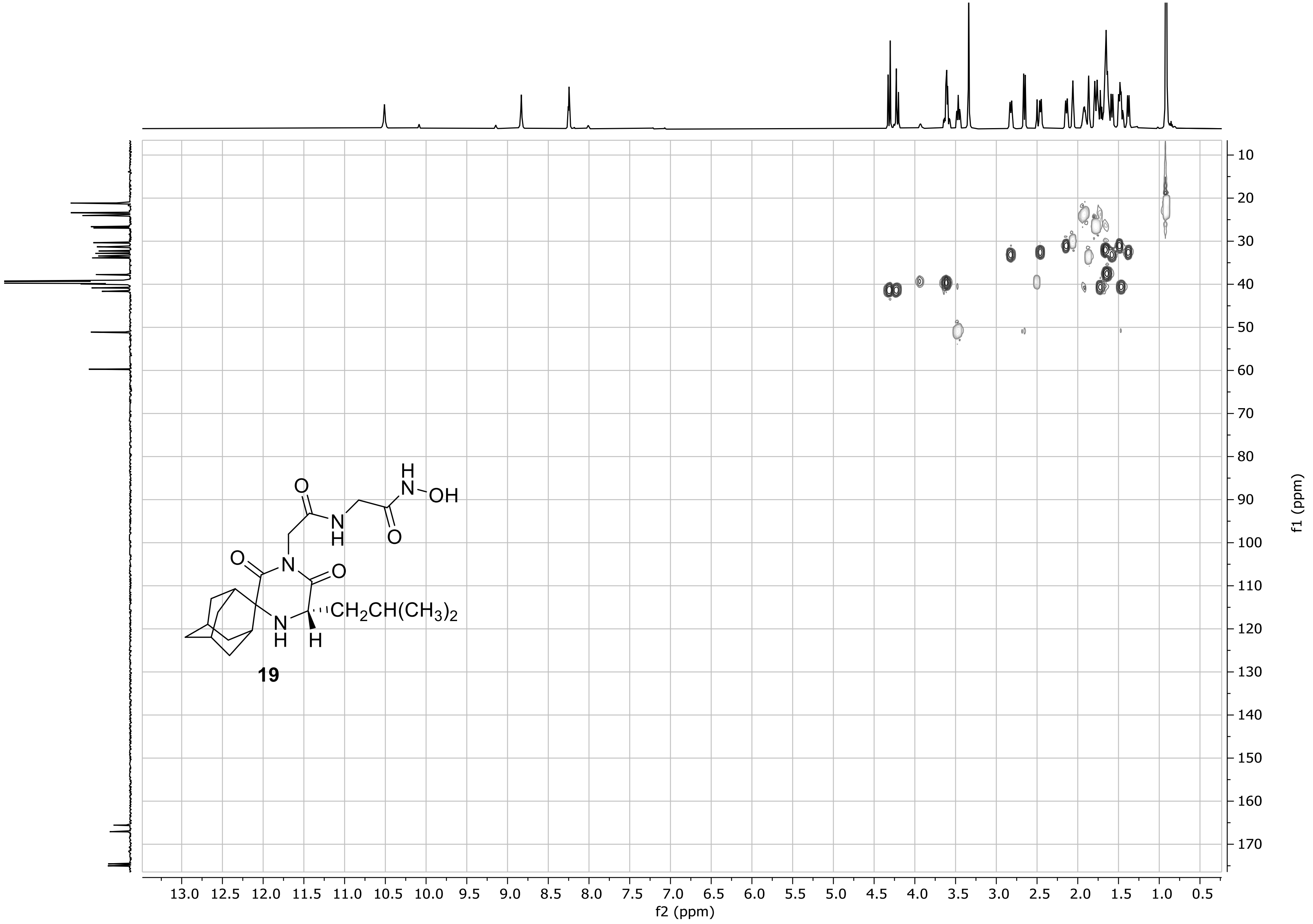

DEPT NMR of **19** (150.9 MHz, DMSO-d_6)

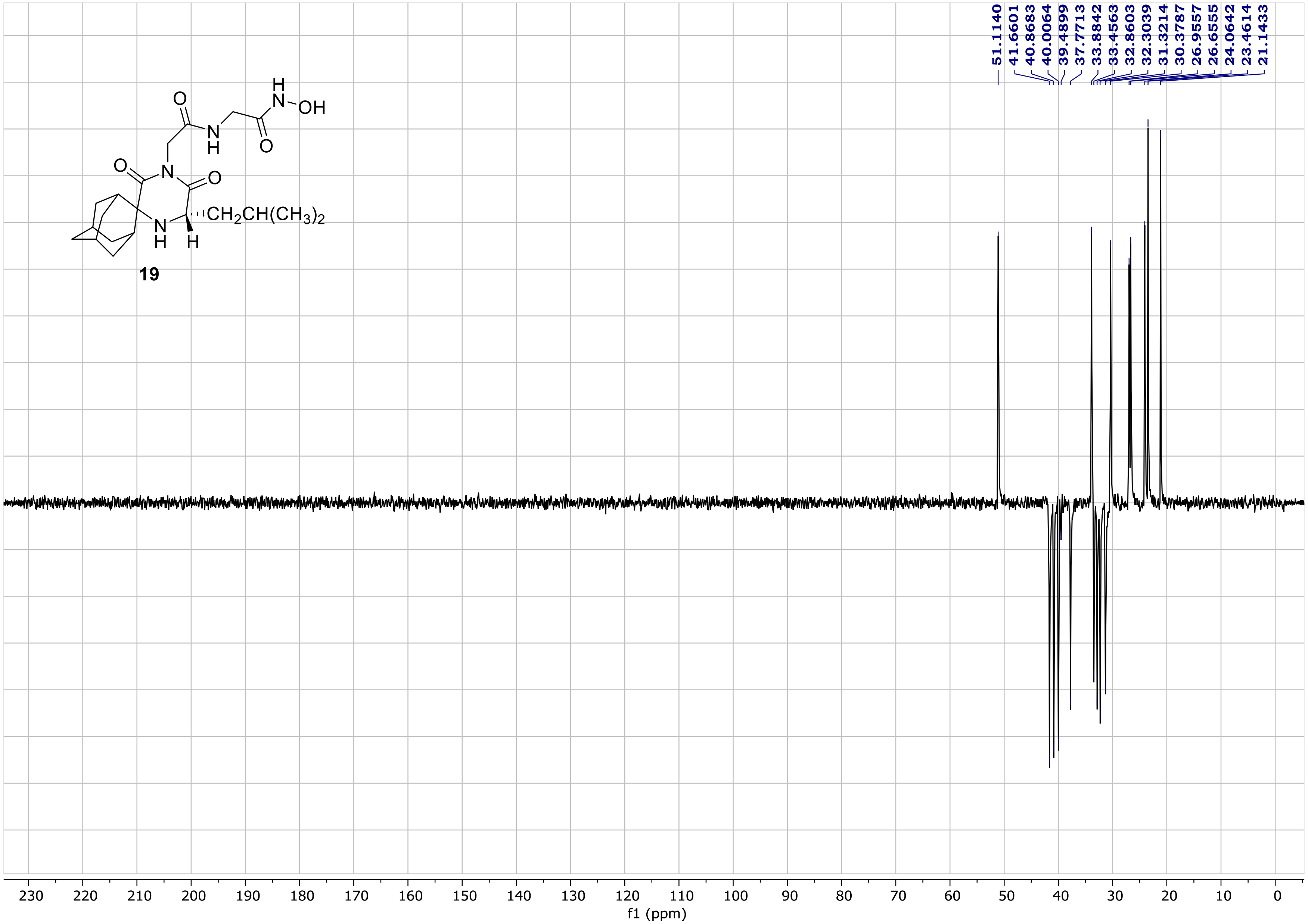

^{1}H NMR of **20** (600.11 MHz, DMSO-d_6)

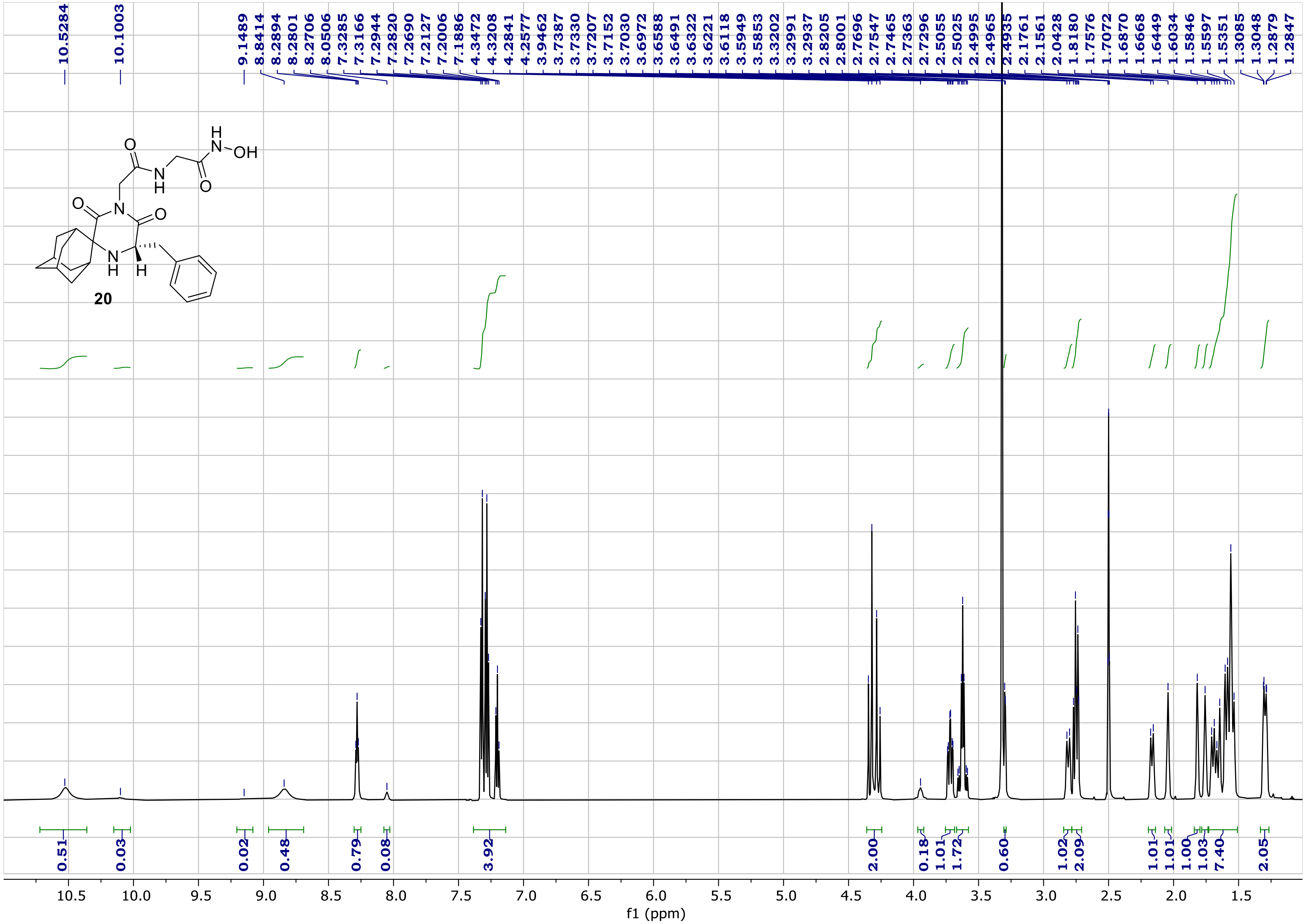

^{13}C NMR of **20** (150.9 MHz, DMSO-d_6)

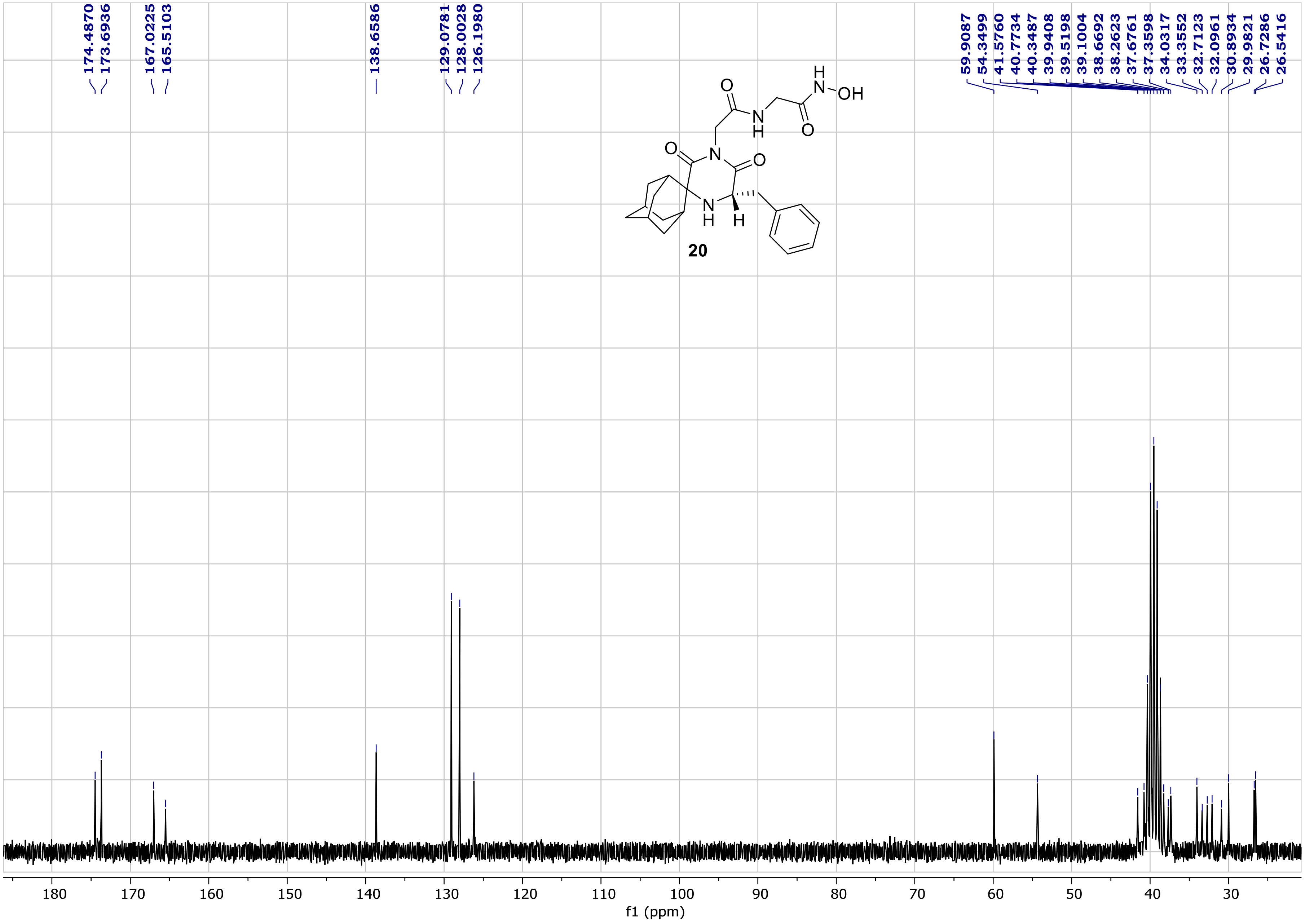

COSY NMR of **20** (600.11 MHz, DMSO-d_6)

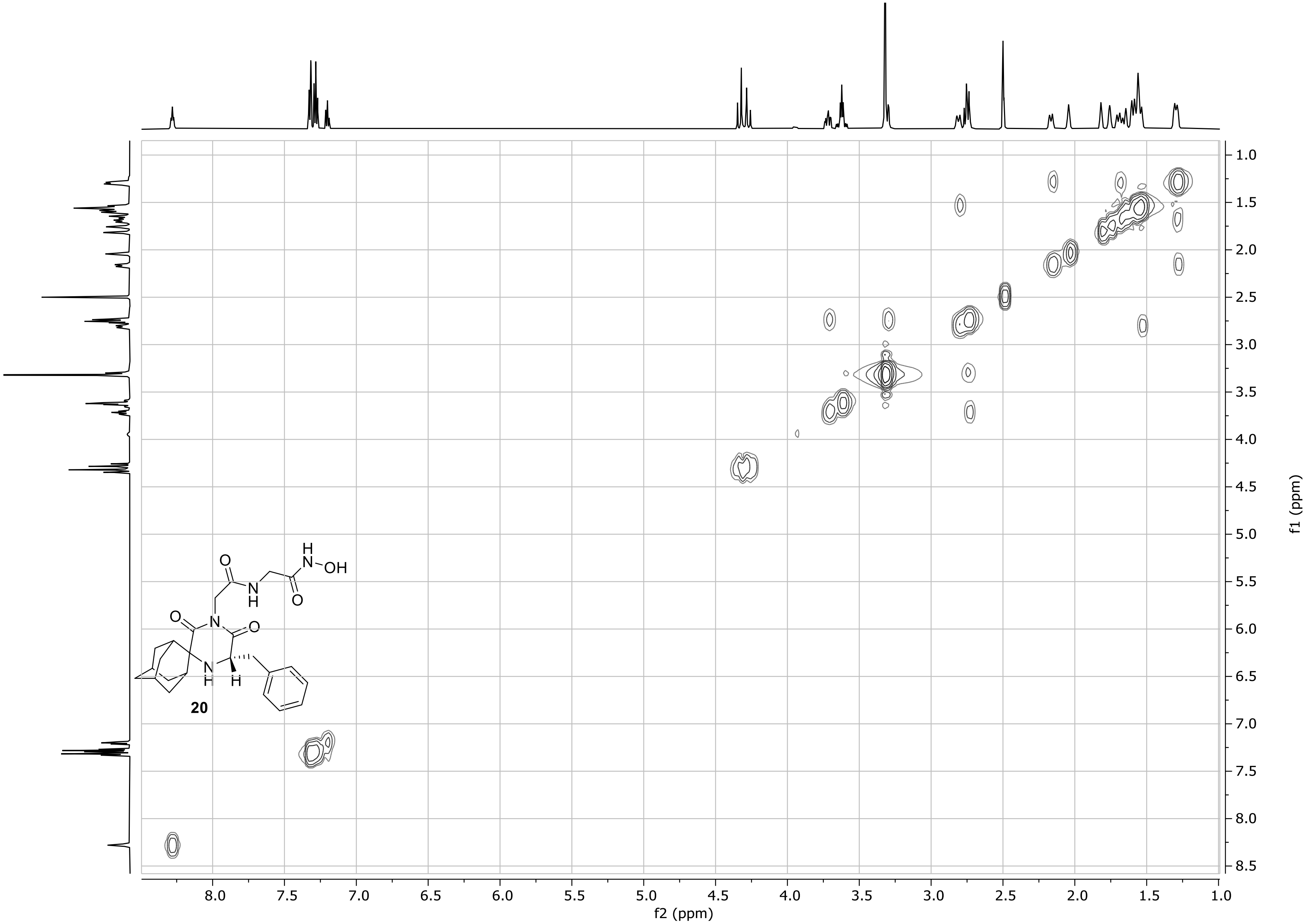

HSQC-DEPT NMR of **20** (600.11 MHz, DMSO-d_6)

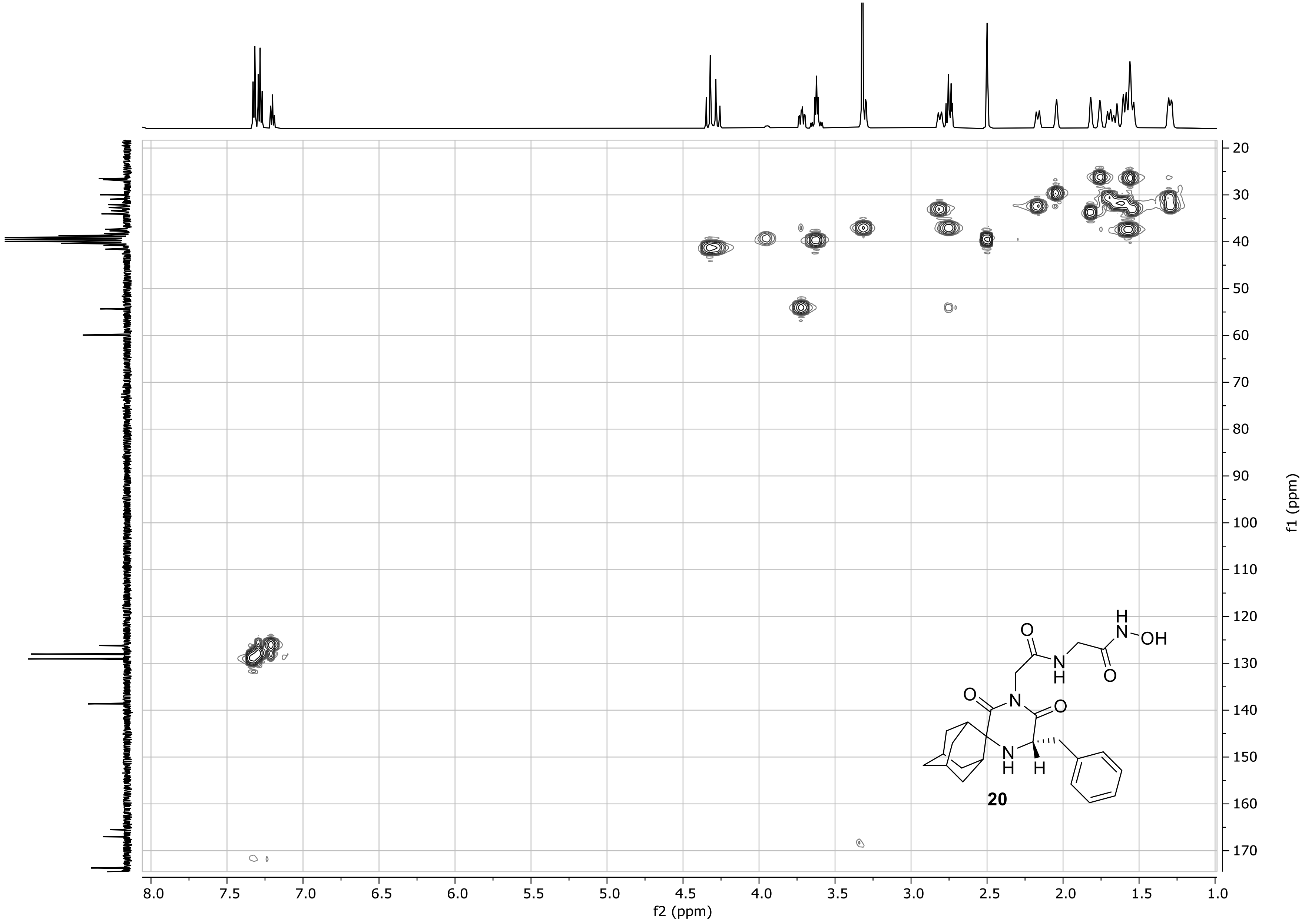

DEPT NMR of **20** (150.9 MHz, DMSO-d_6)

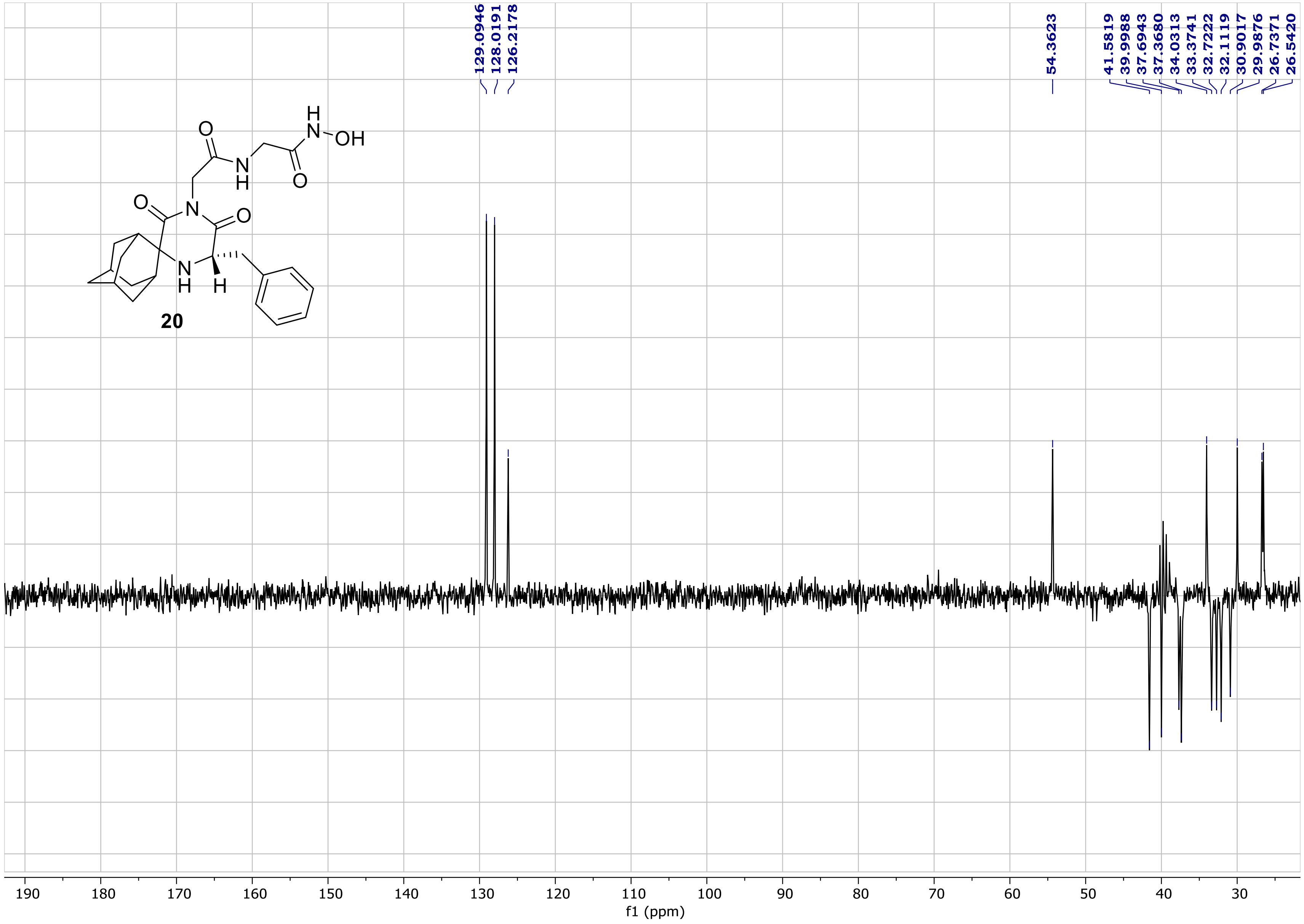

^{1}H NMR of **21** (400.13 MHz, DMSO-d_6)

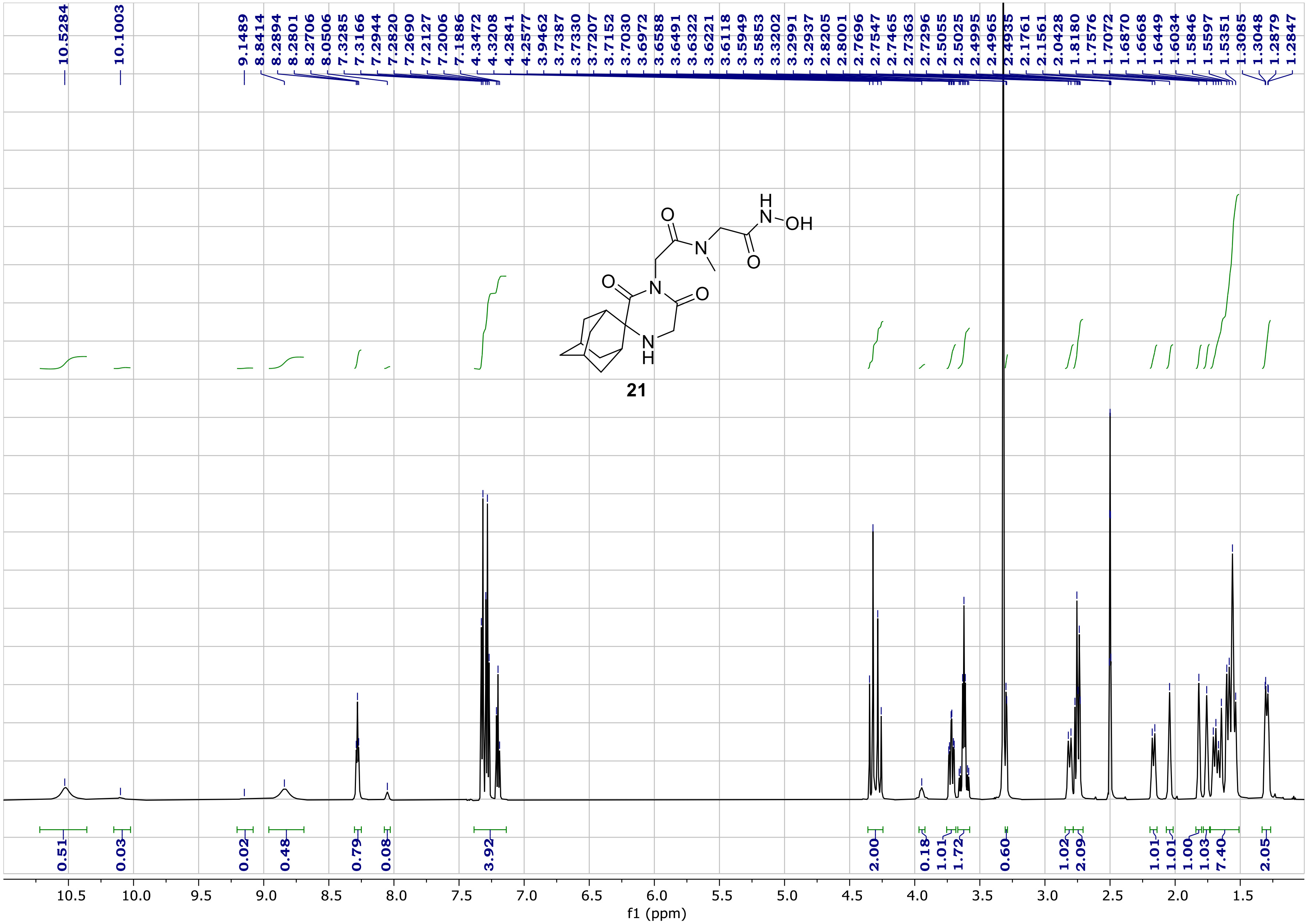

^{13}C NMR of **21** (50.32 MHz, DMSO-d_6)

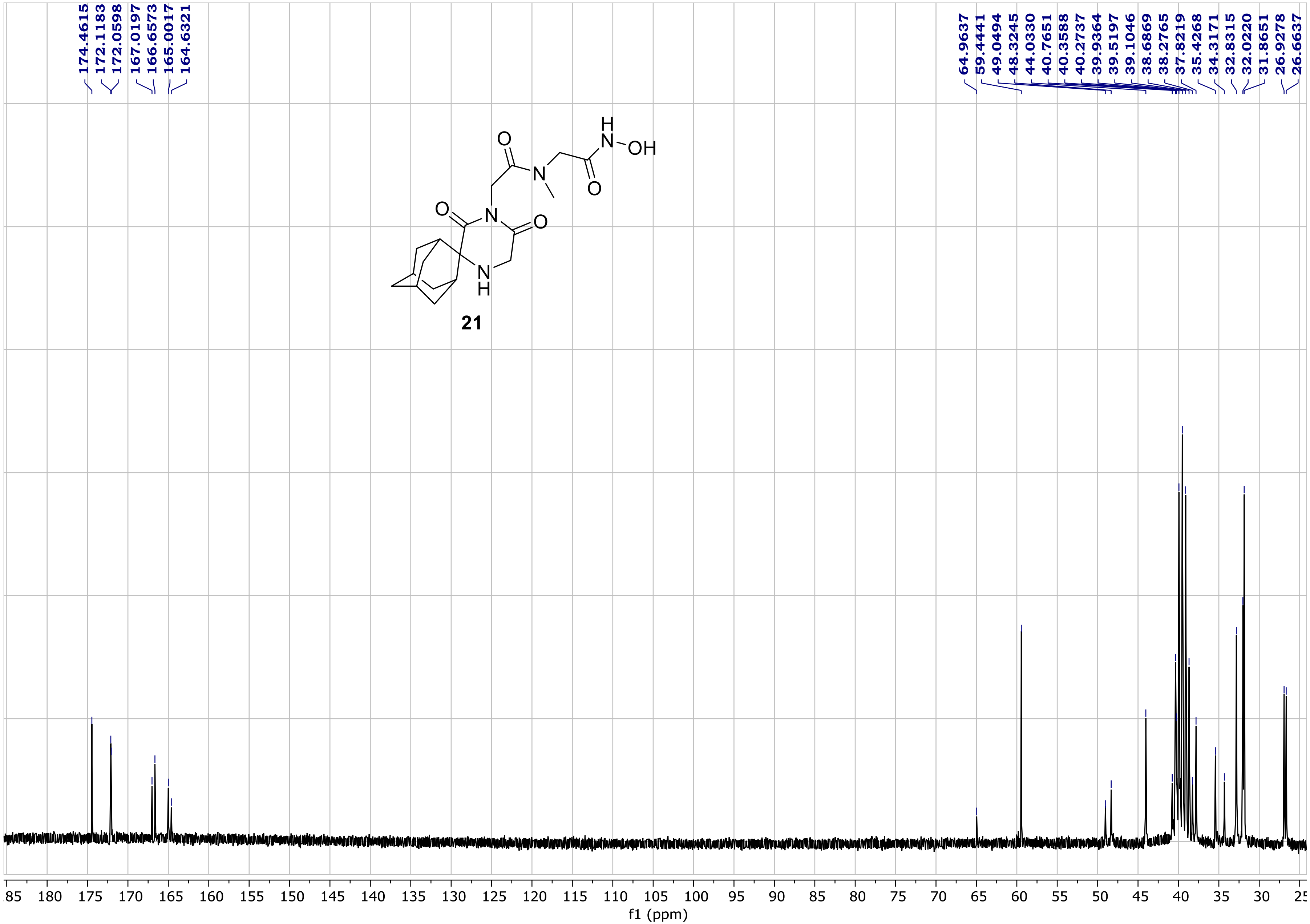

COSY NMR of **21** (400.13 MHz, DMSO-d_6)

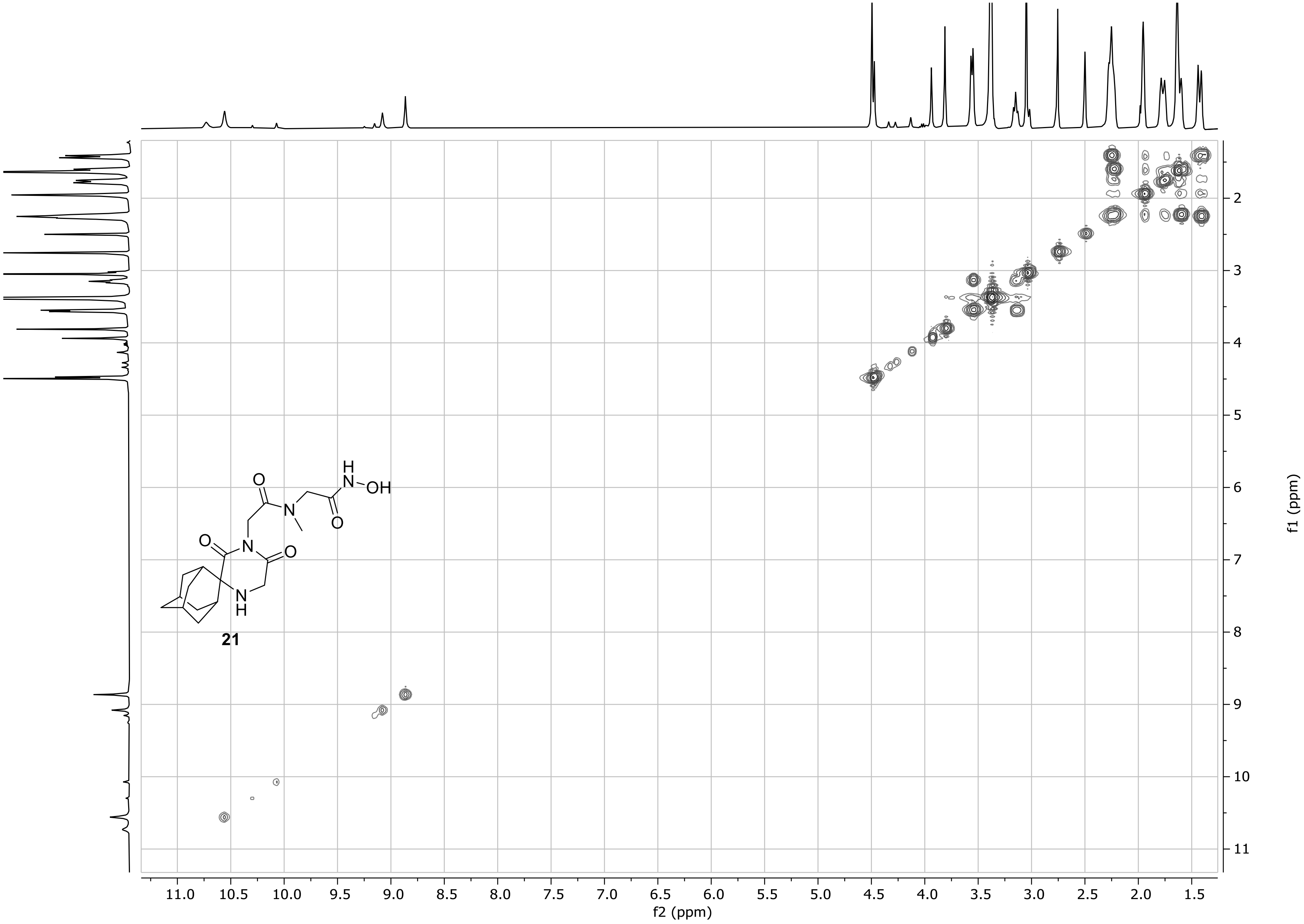

HSQC NMR of **21** (400.13 MHz, DMSO-d_6)

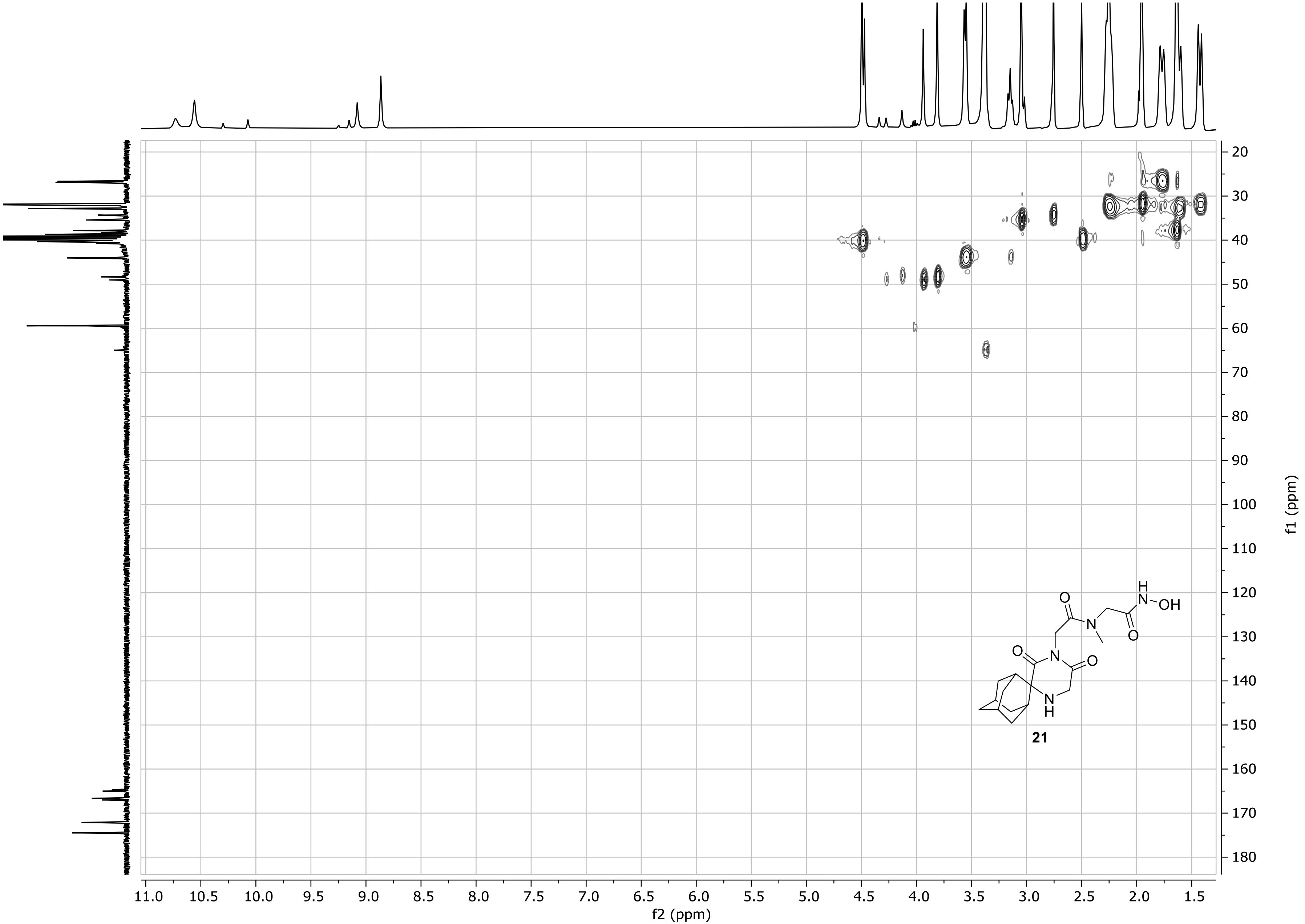

DEPT NMR of **21** (150.9 MHz, DMSO-d_6)

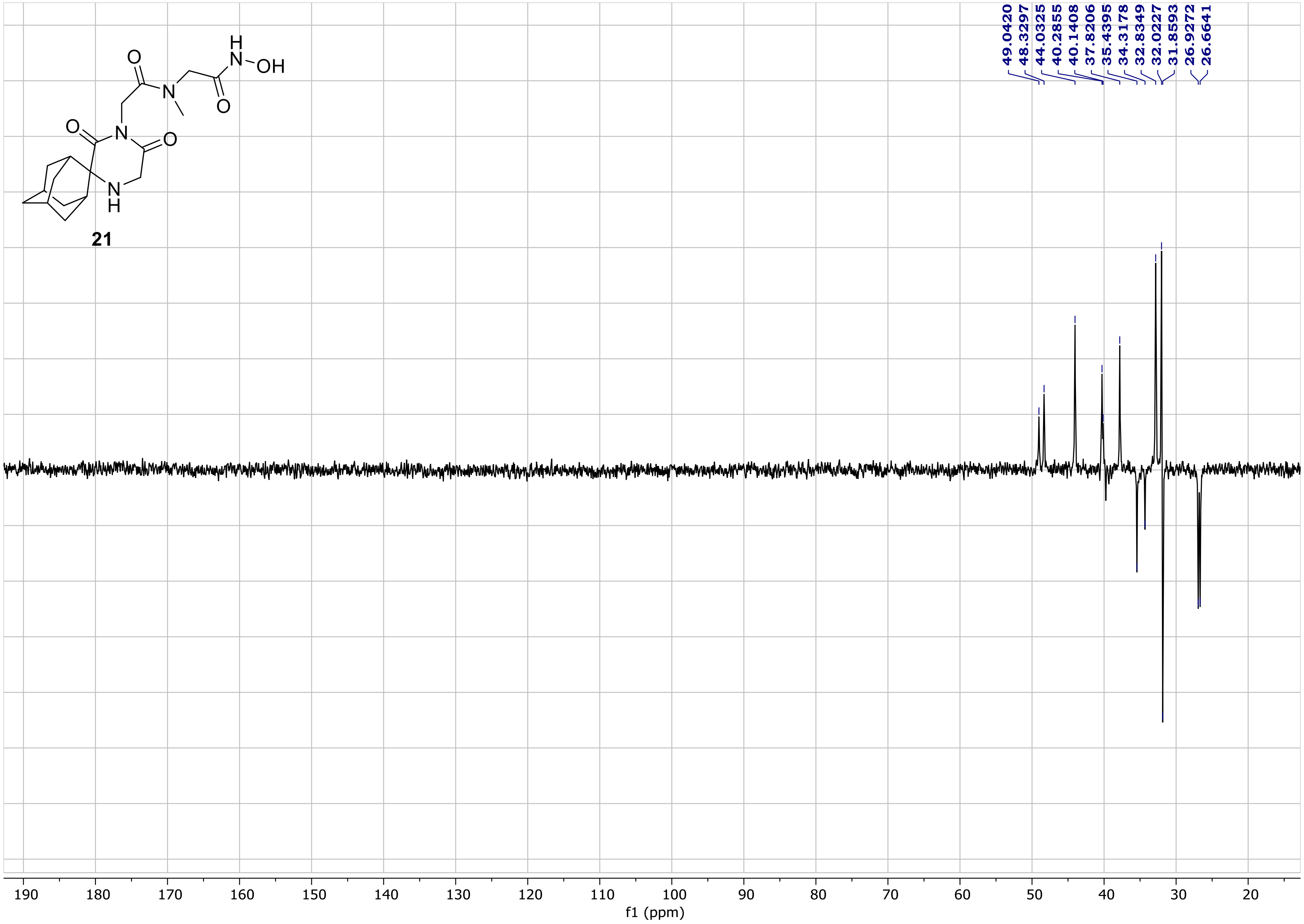

^{1}H NMR of **22** (600.11 MHz, DMSO-d_6)

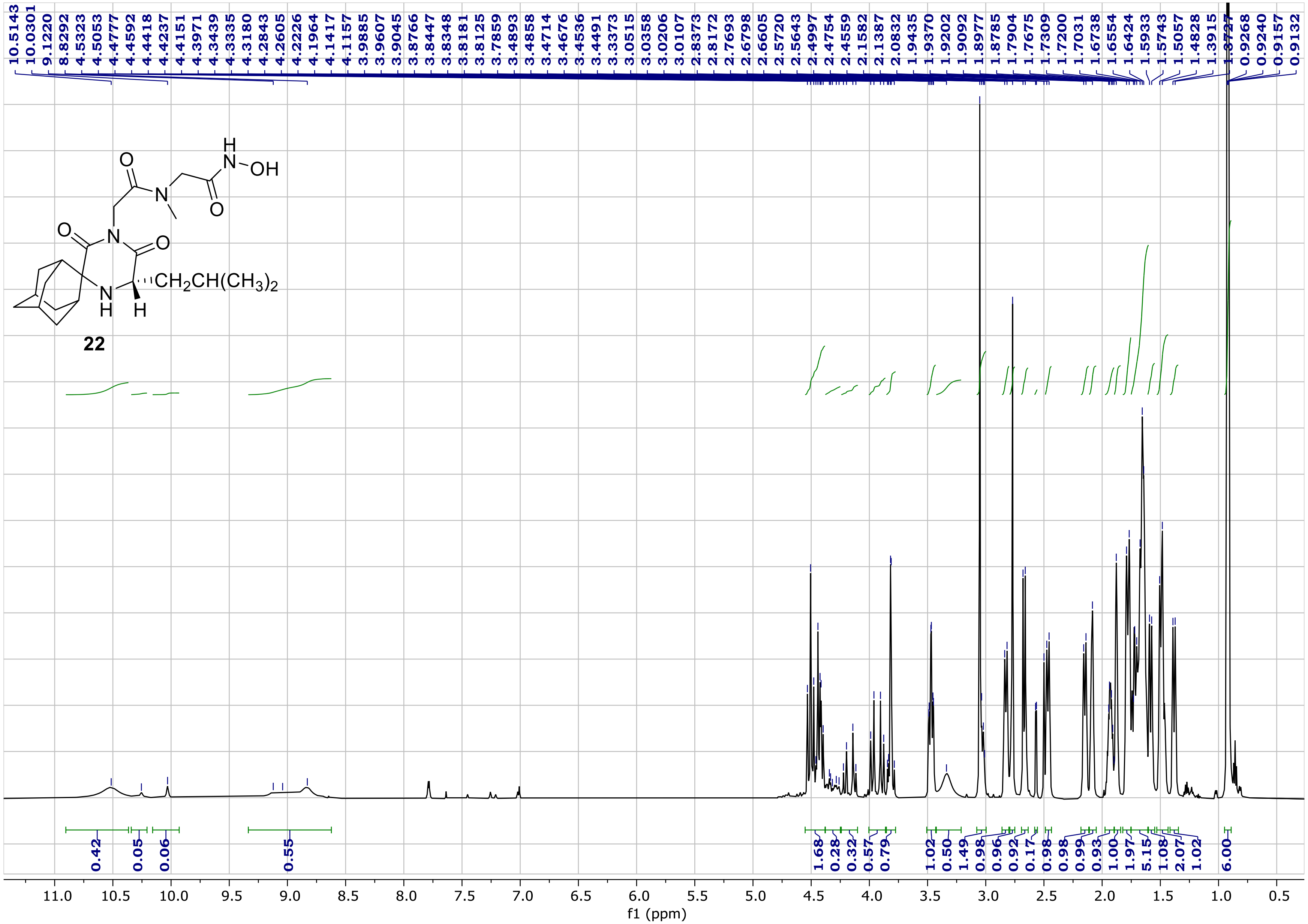

^{13}C NMR of **22** (150.9 MHz, DMSO-d_6)

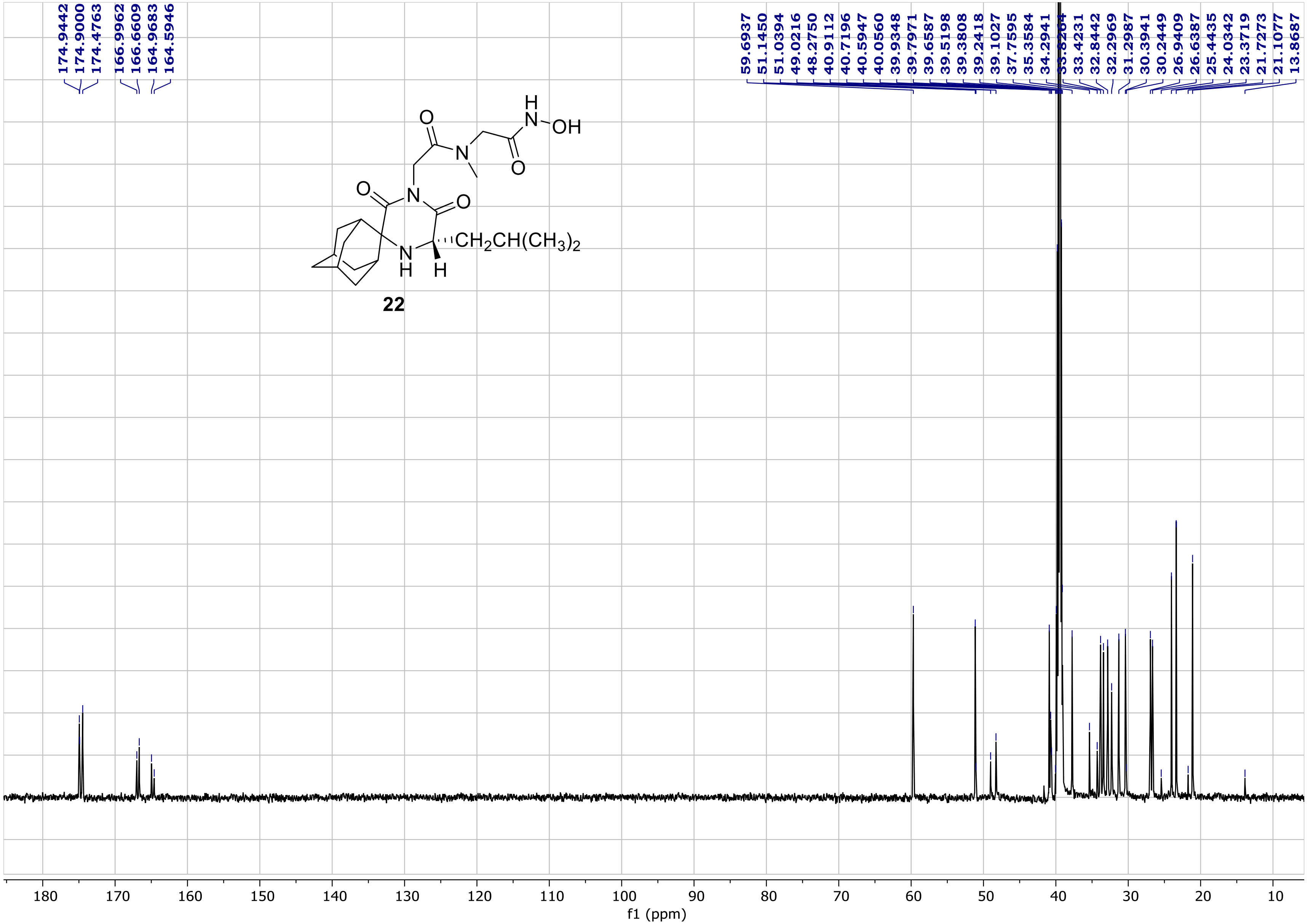

COSY NMR of **22** (600.11 MHz, DMSO-d_6)

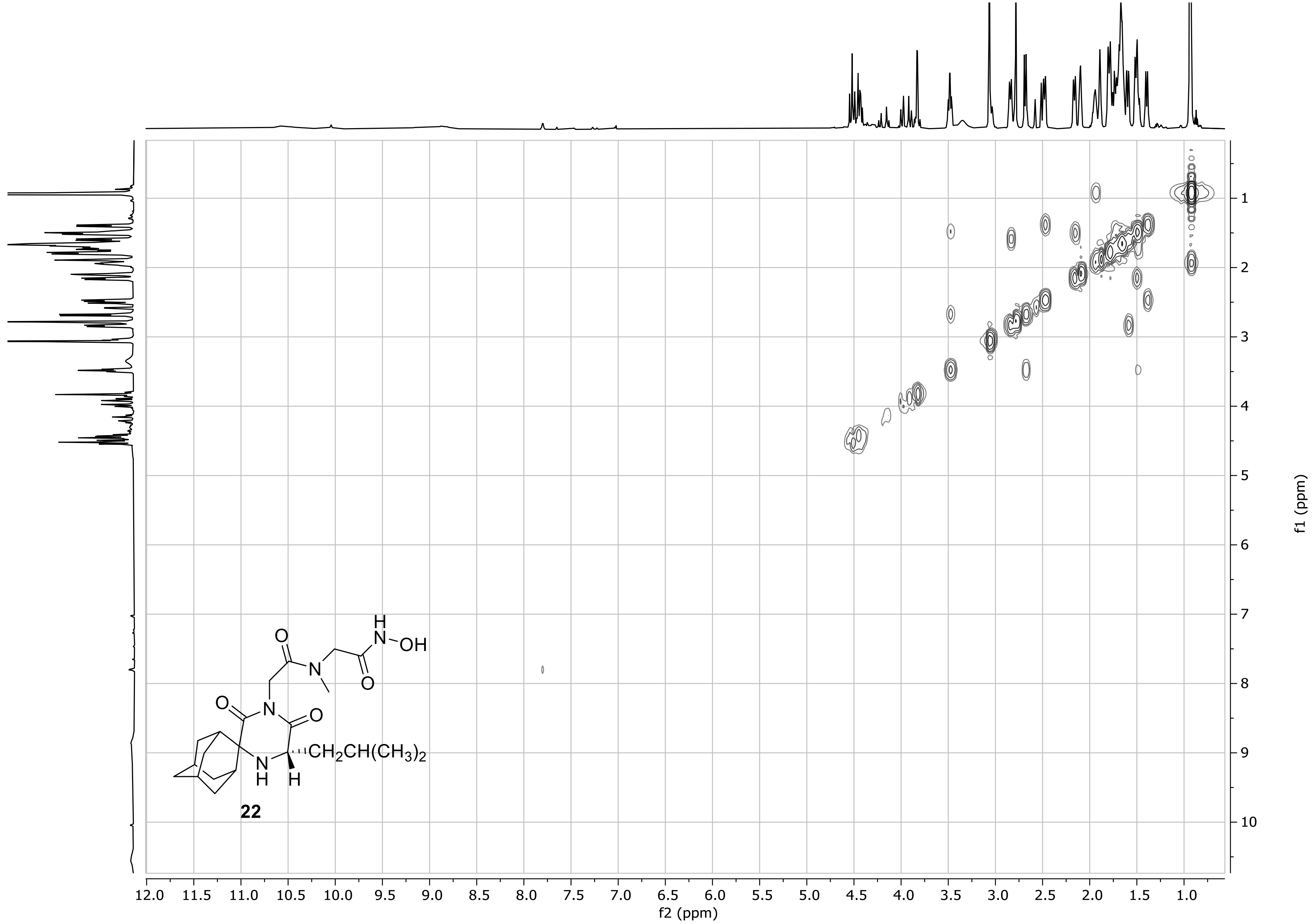

HSQC-DEPT NMR of **22** (600.11 MHz, DMSO-d_6)

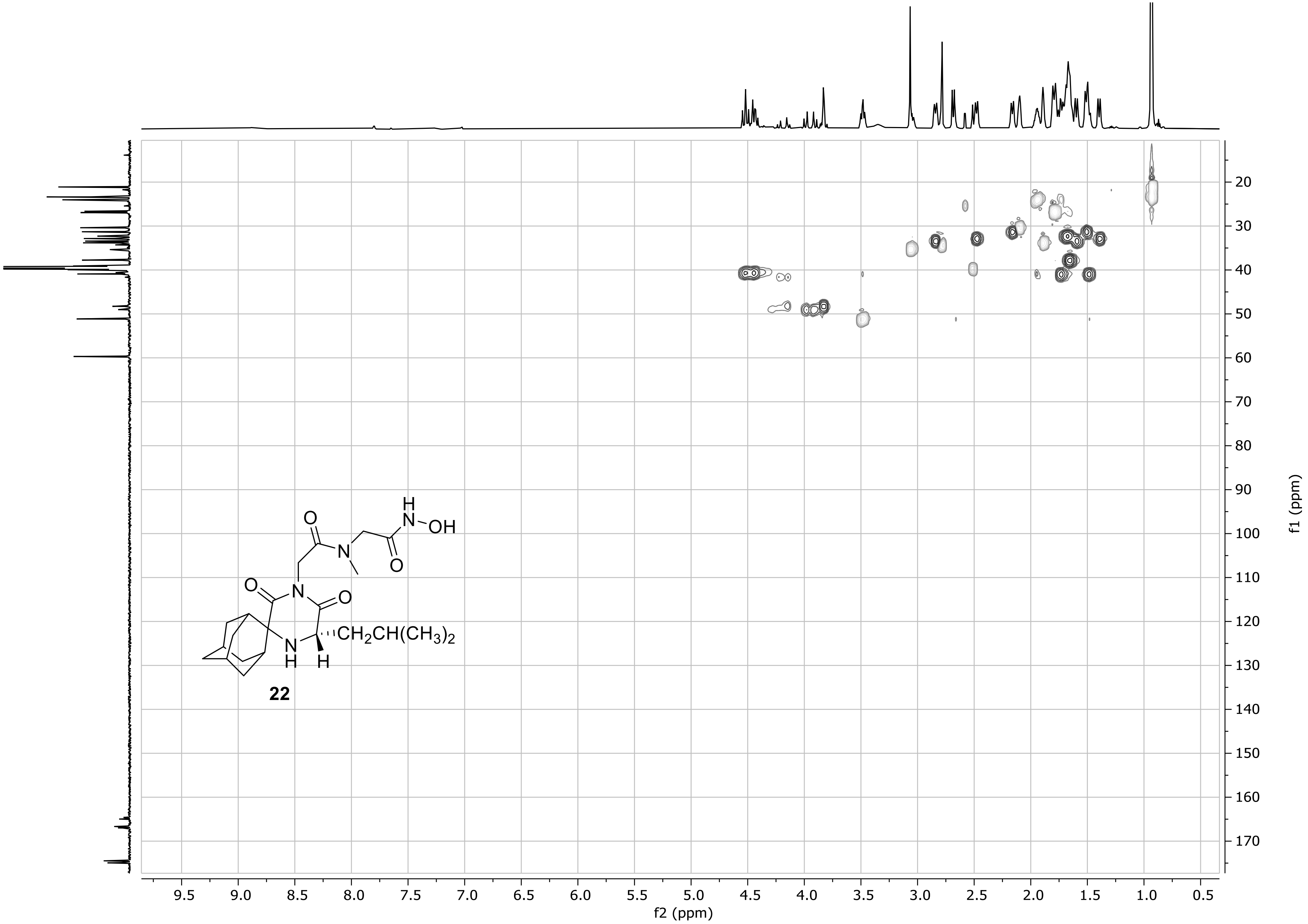

DEPT NMR of **22** (150.9 MHz, DMSO-d_6)

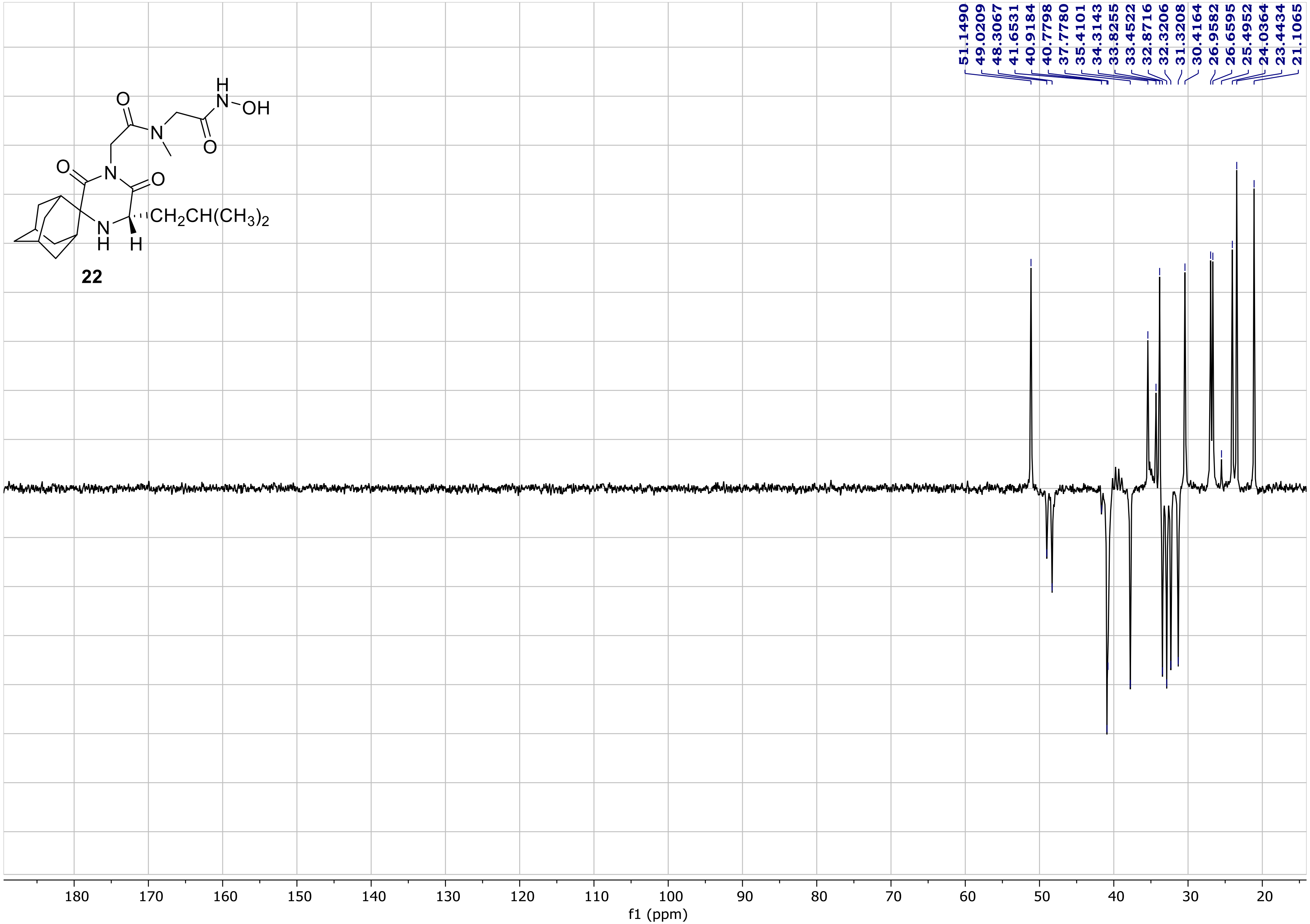

^{1}H NMR of **23** (600.11 MHz, DMSO-d_6)

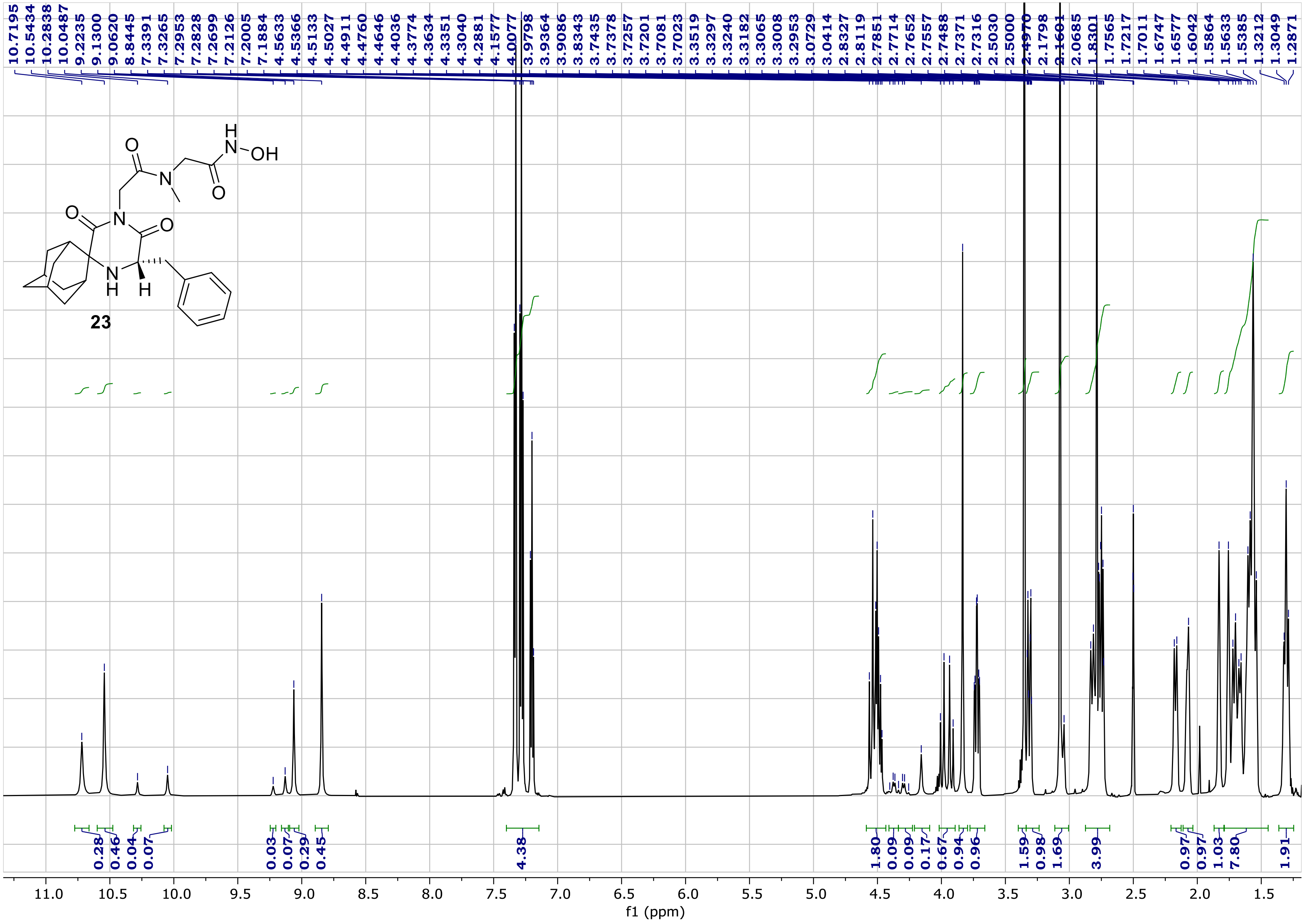

^{13}C NMR of **23** (150.9 MHz, DMSO-d_6)

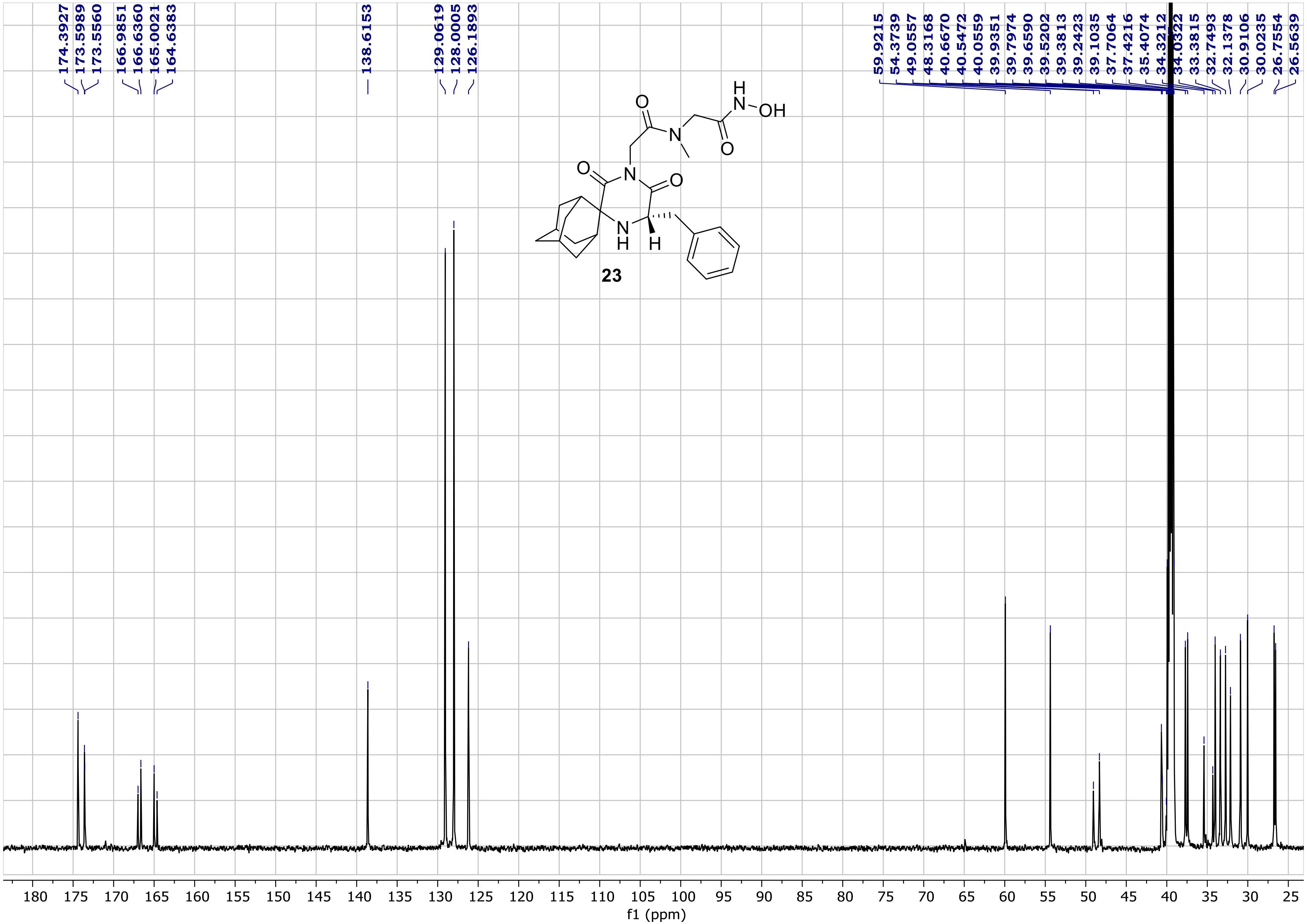

COSY NMR of **23** (600.11 MHz, DMSO-d_6)

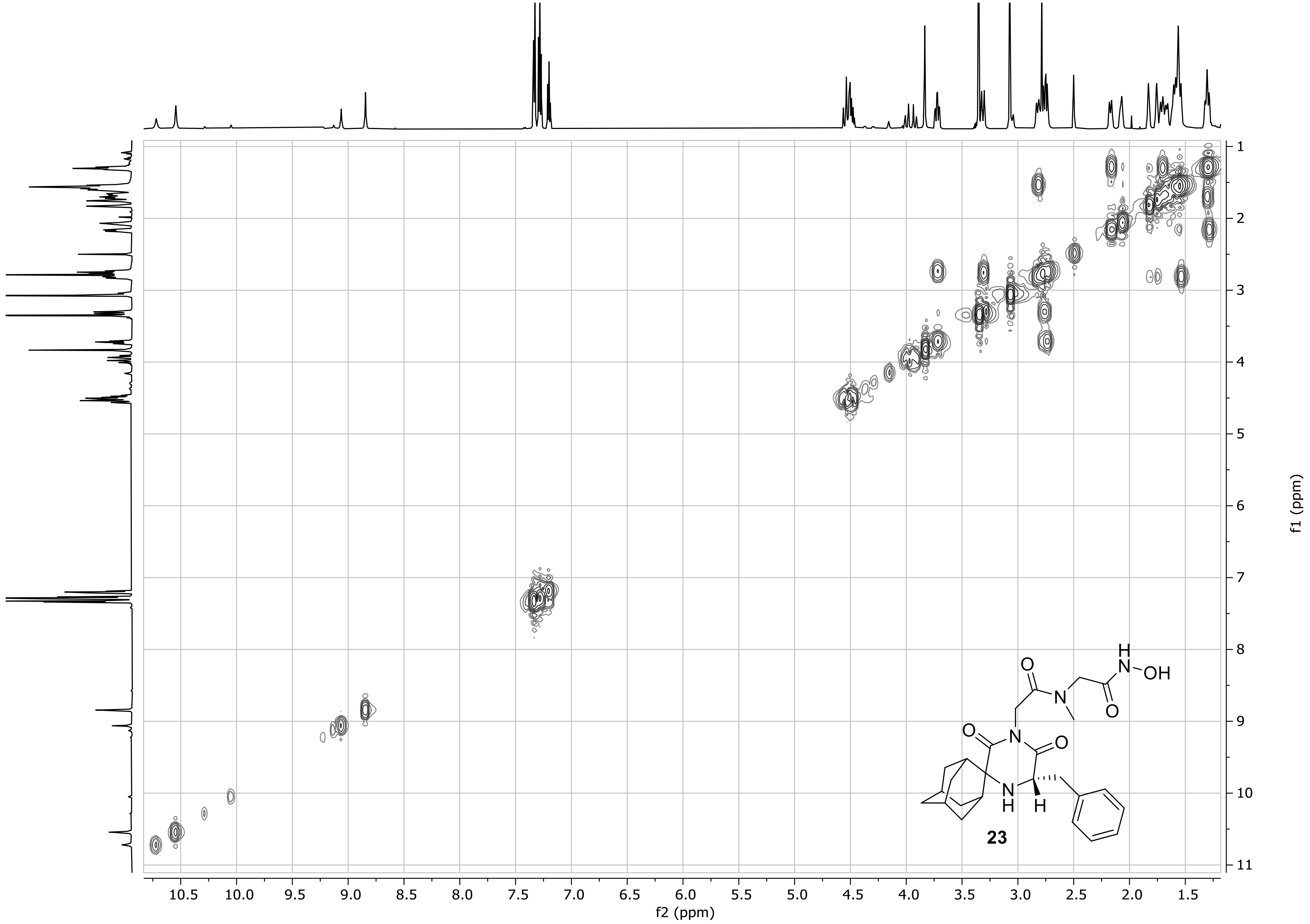

HSQC-DEPT NMR of **23** (600.11 MHz, DMSO-d_6)

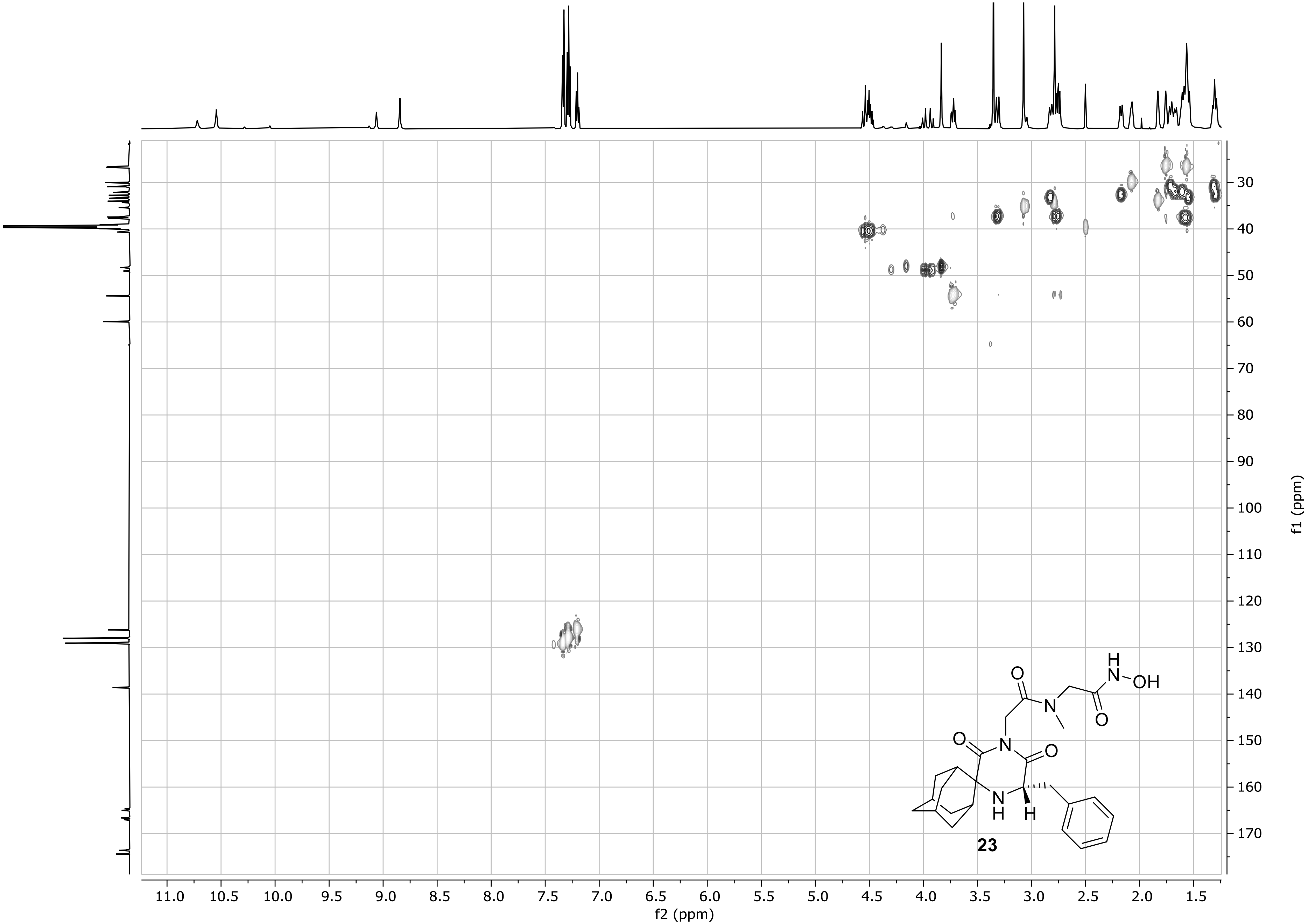

^{1}H NMR of **24** (400.13 MHz, DMSO-d_6)

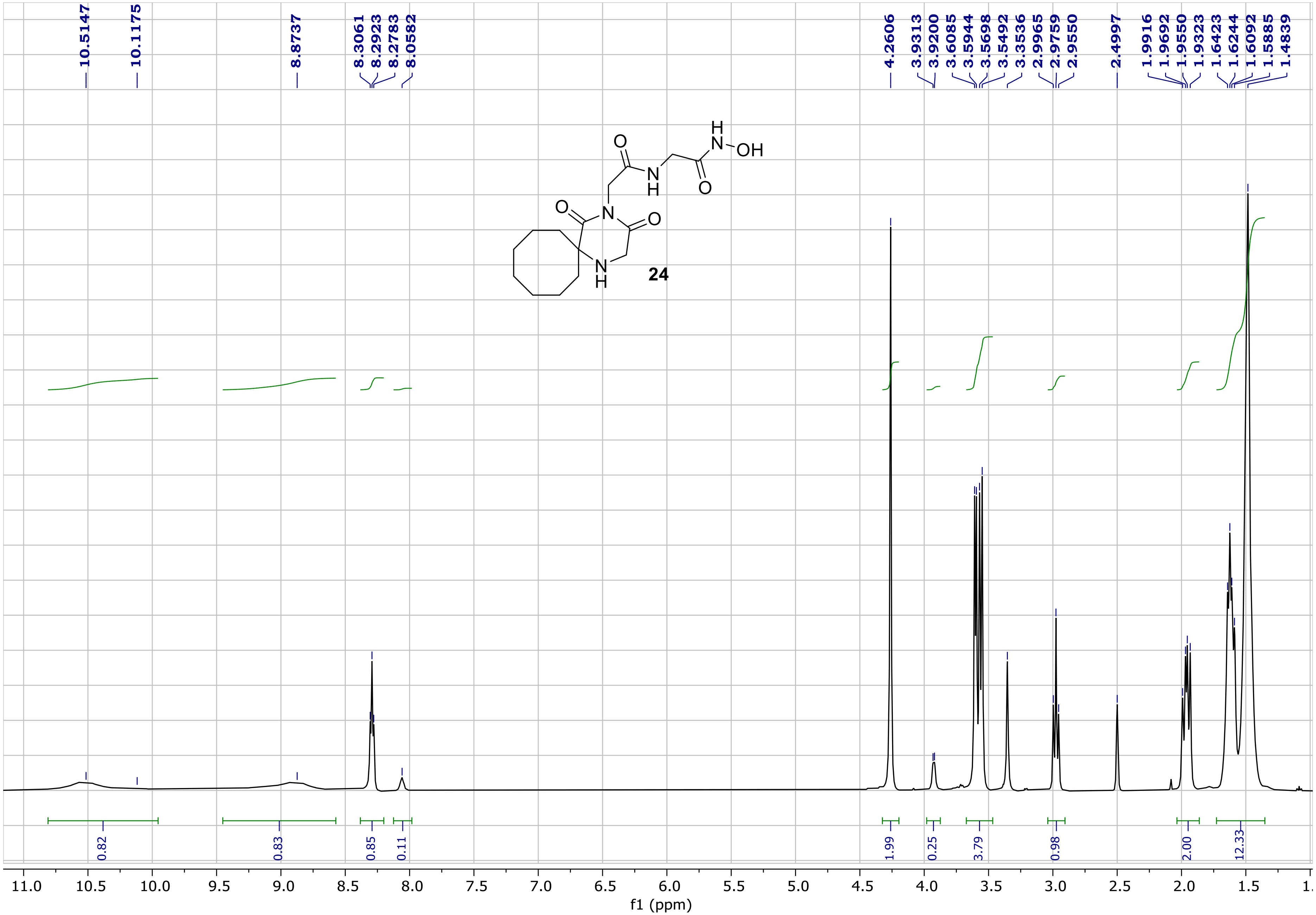

^{13}C NMR of **24** (50.32 MHz, DMSO-d_6)

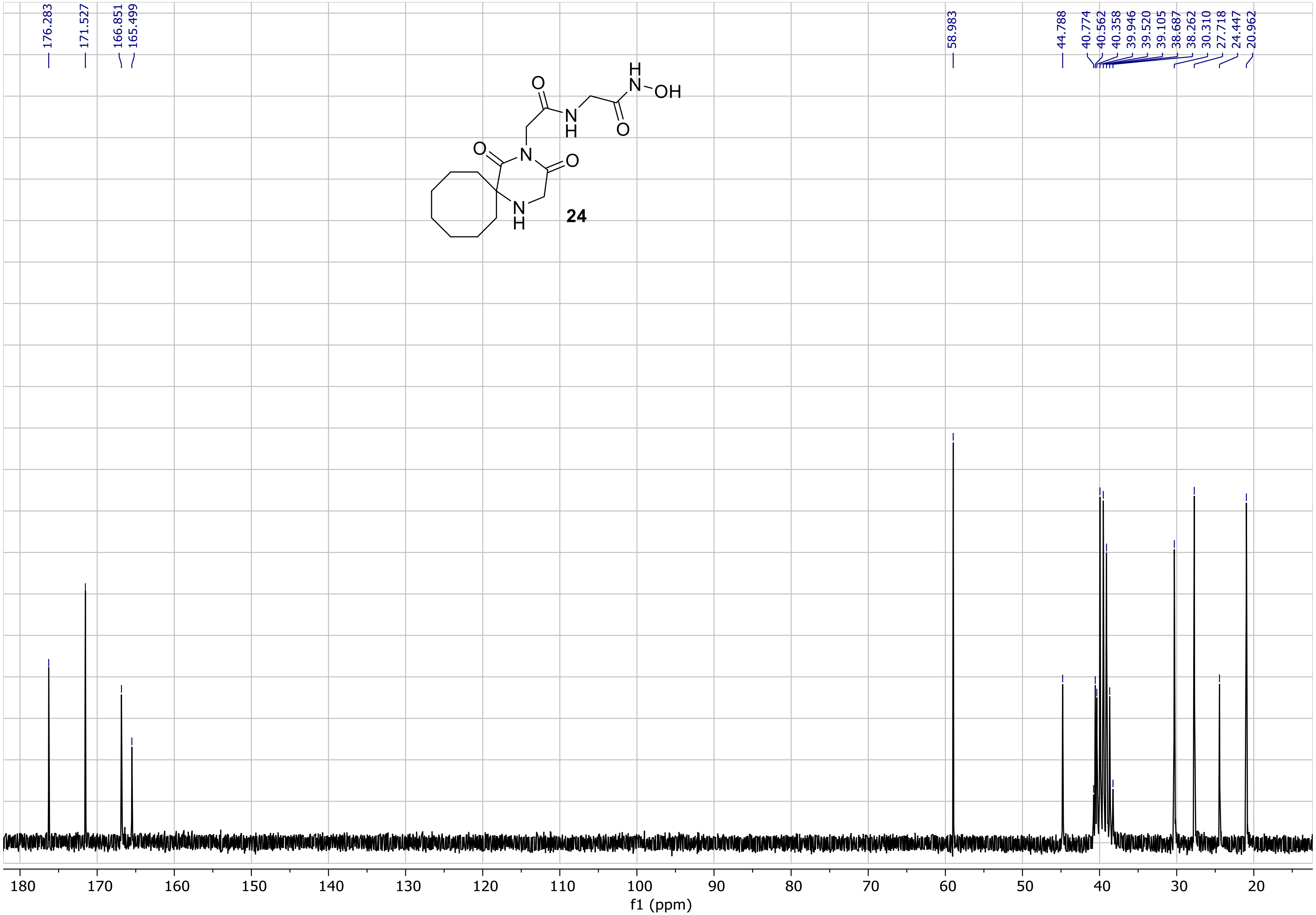

COSY NMR of **24** (400.13 MHz, DMSO-d_6)

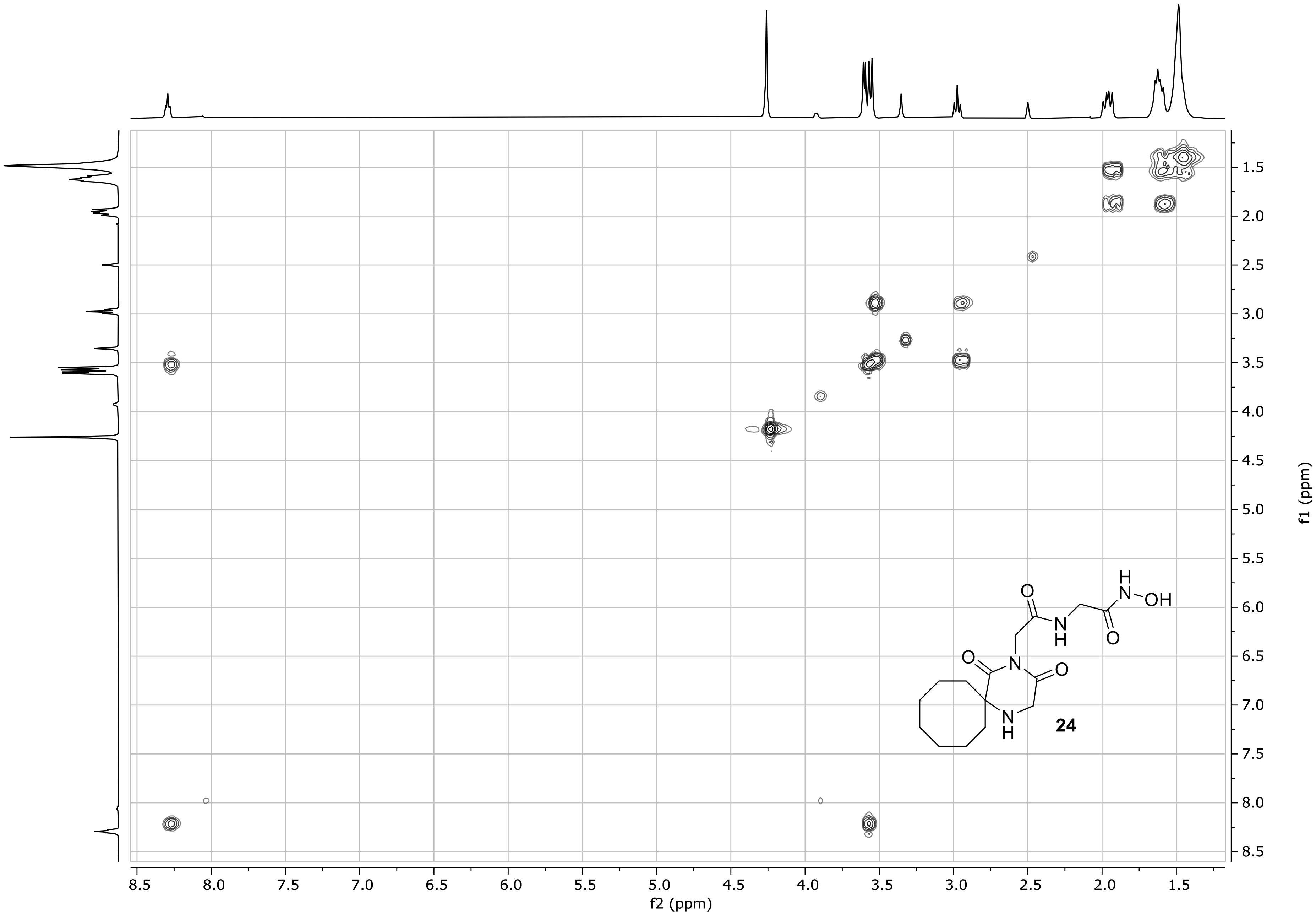

HSQC NMR of **24** (400.13 MHz, DMSO-d_6)

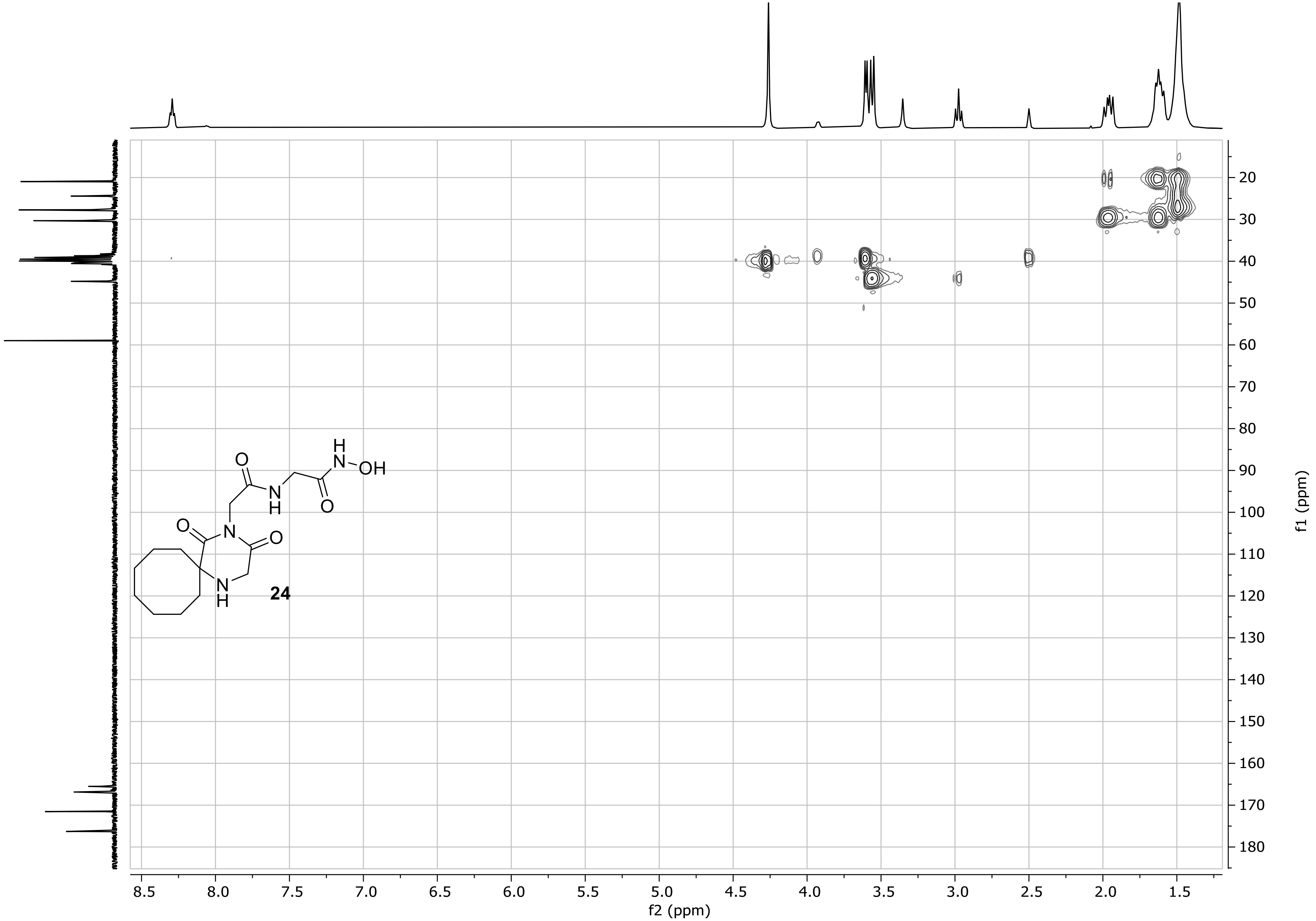

^{1}H NMR of **25** (400.13 MHz, DMSO-d_6)

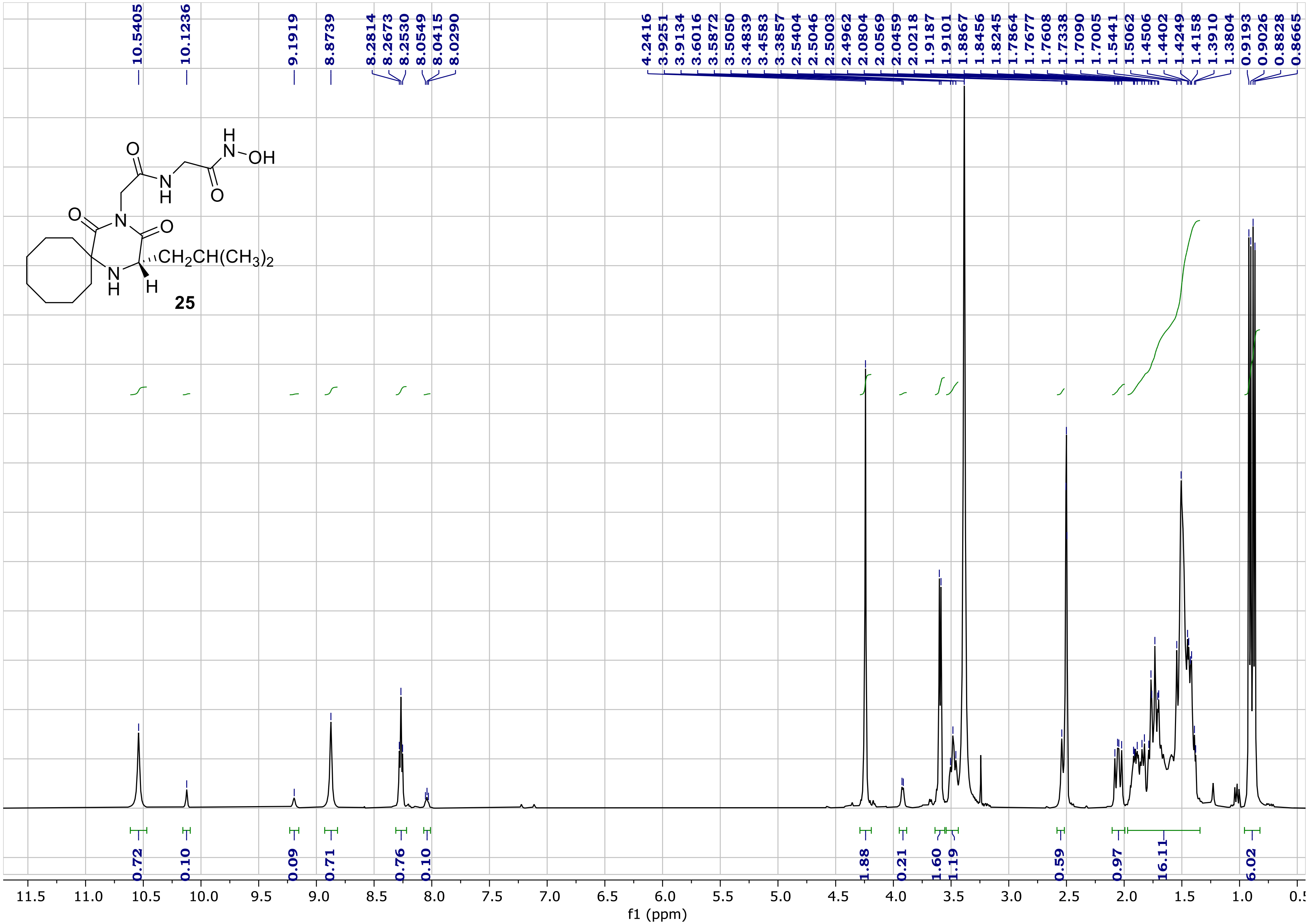

^{13}C NMR of **25** (50.32 MHz, DMSO-d_6)

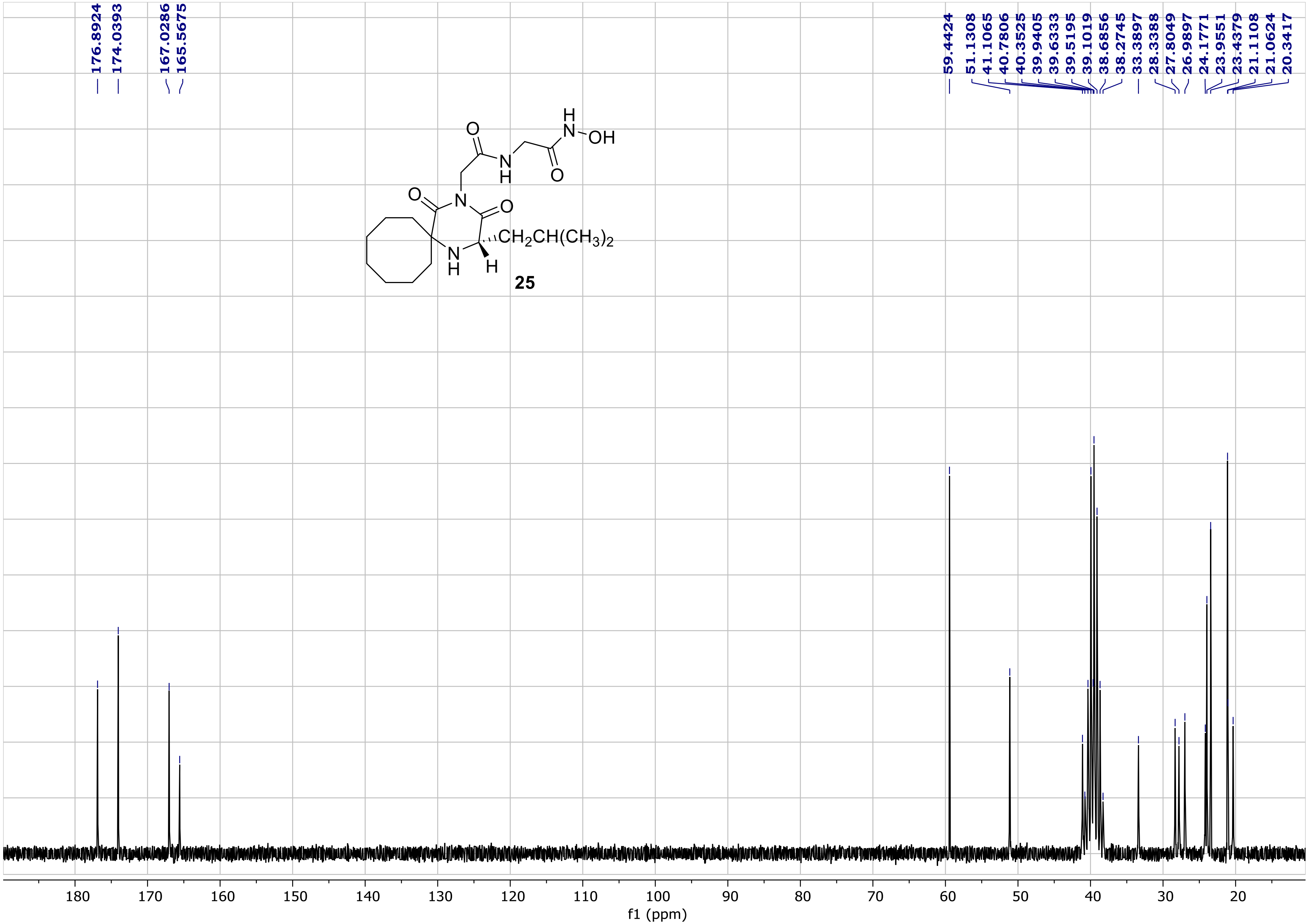

COSY NMR of **25** (400.13 MHz, DMSO-d_6)

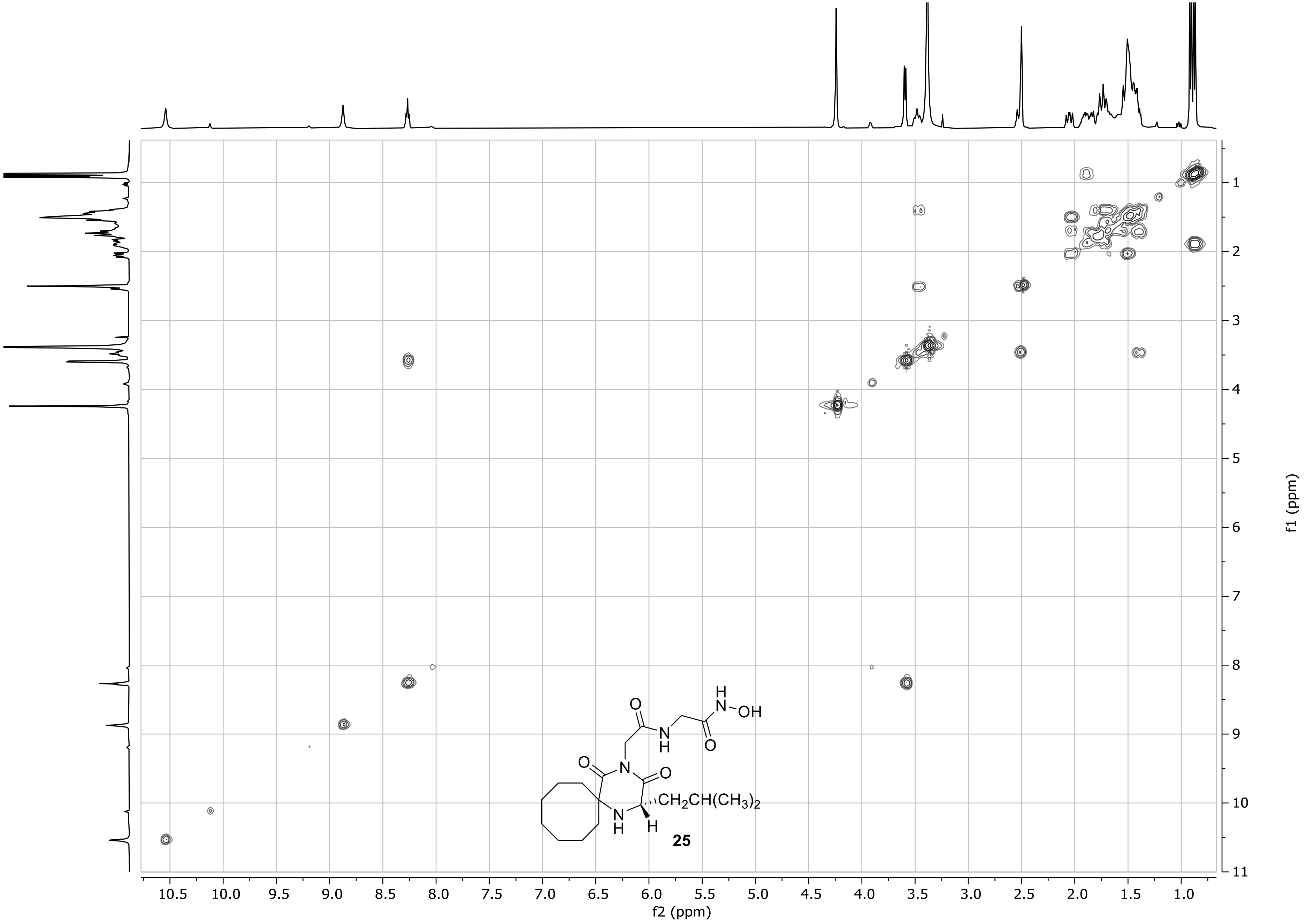

HSQC NMR of **25** (400.13 MHz, DMSO-d_6)

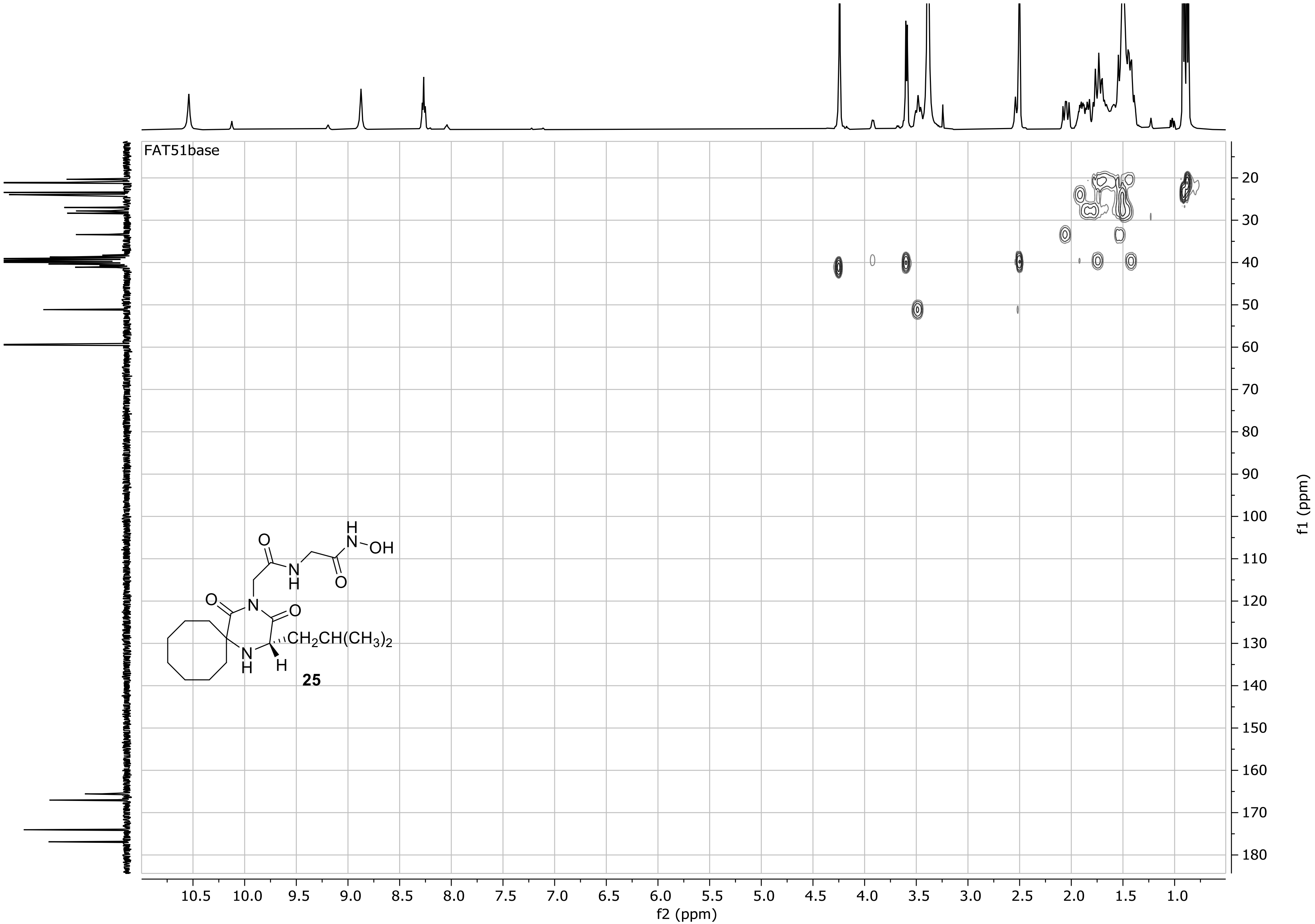

DEPT NMR of **25** (50.32 MHz, DMSO-d_6)

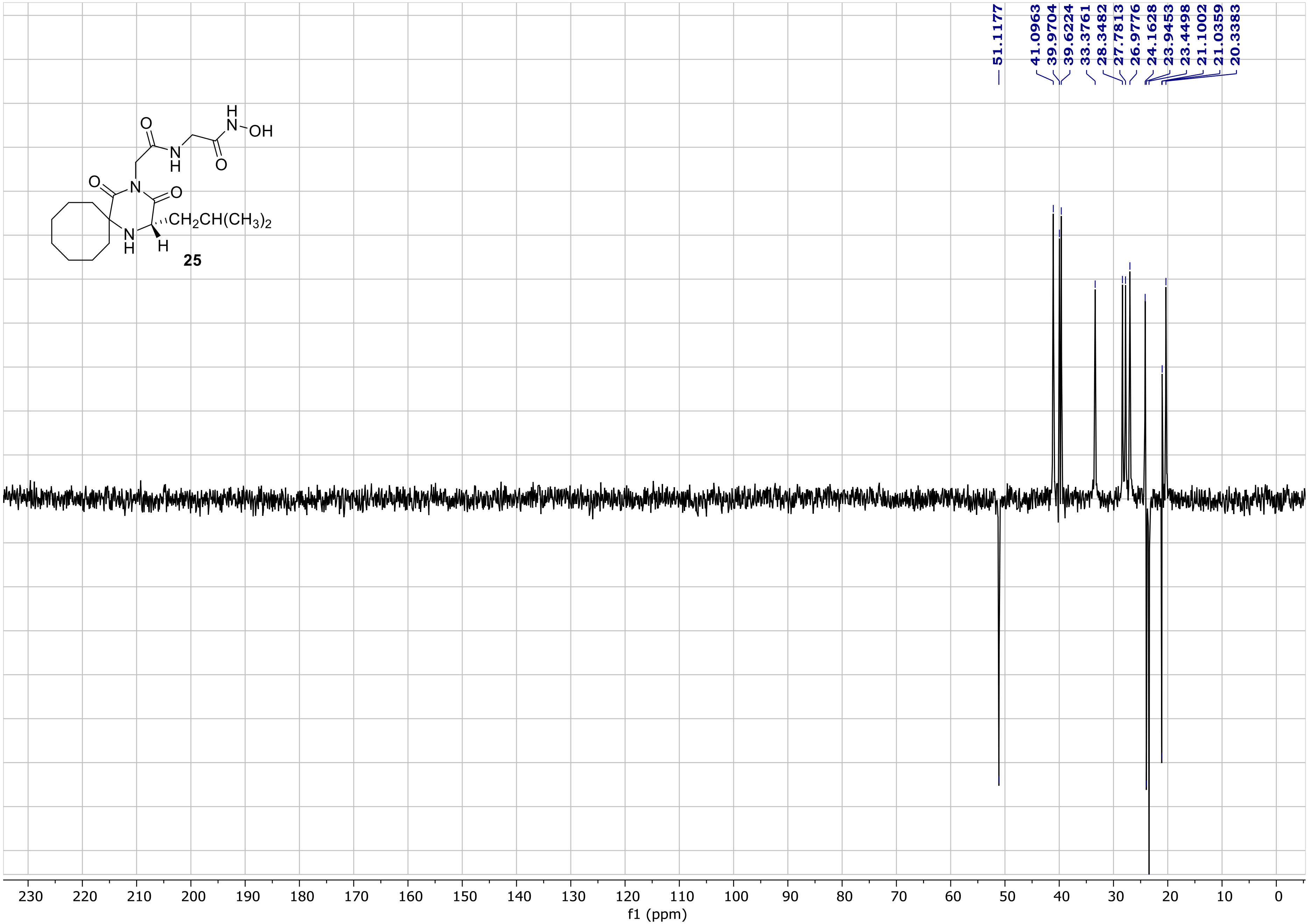

^{1}H NMR of **26** (600.11 MHz, DMSO-d_6)

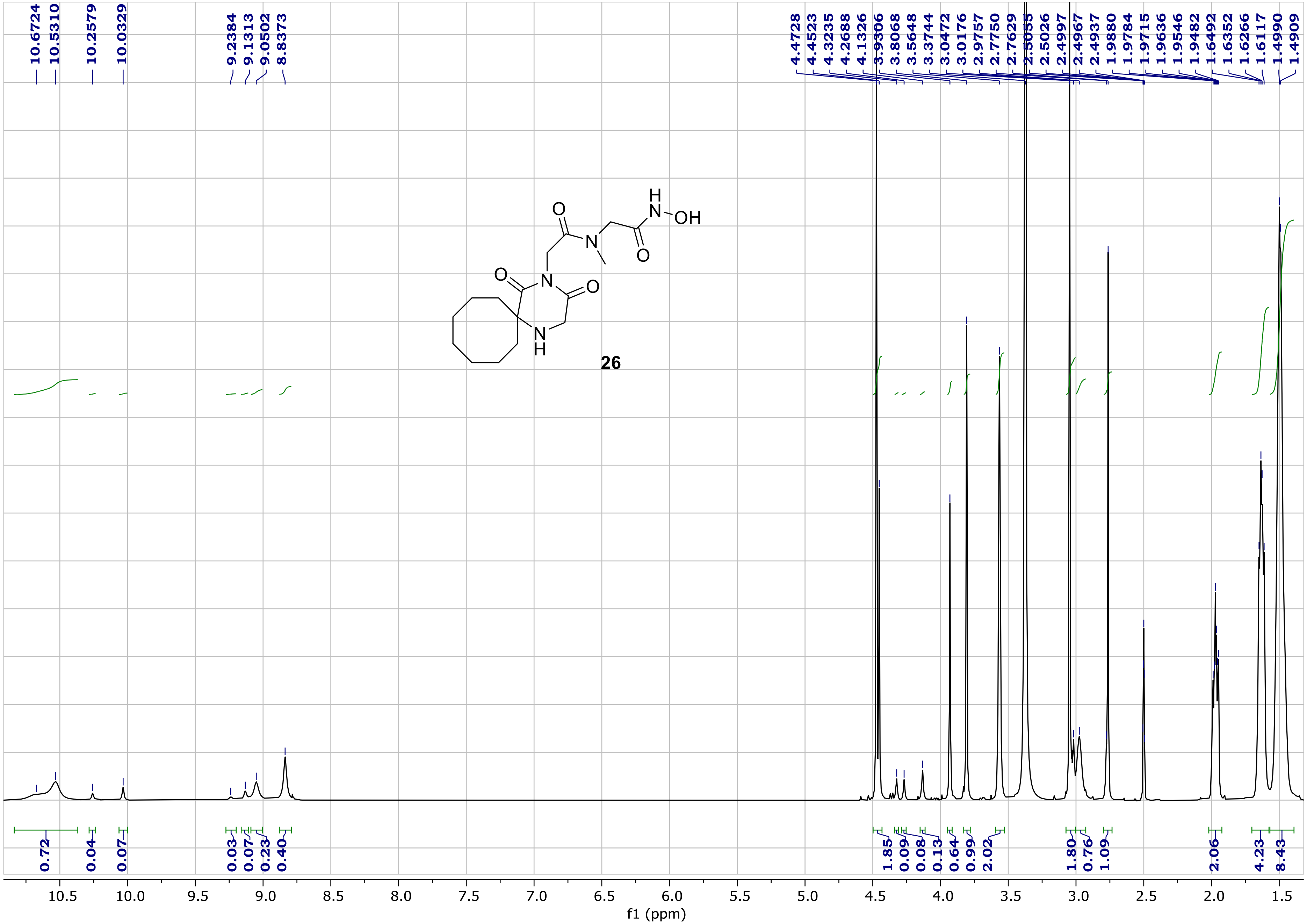

^{13}C NMR of **26** (150.9 MHz, DMSO-d_6)

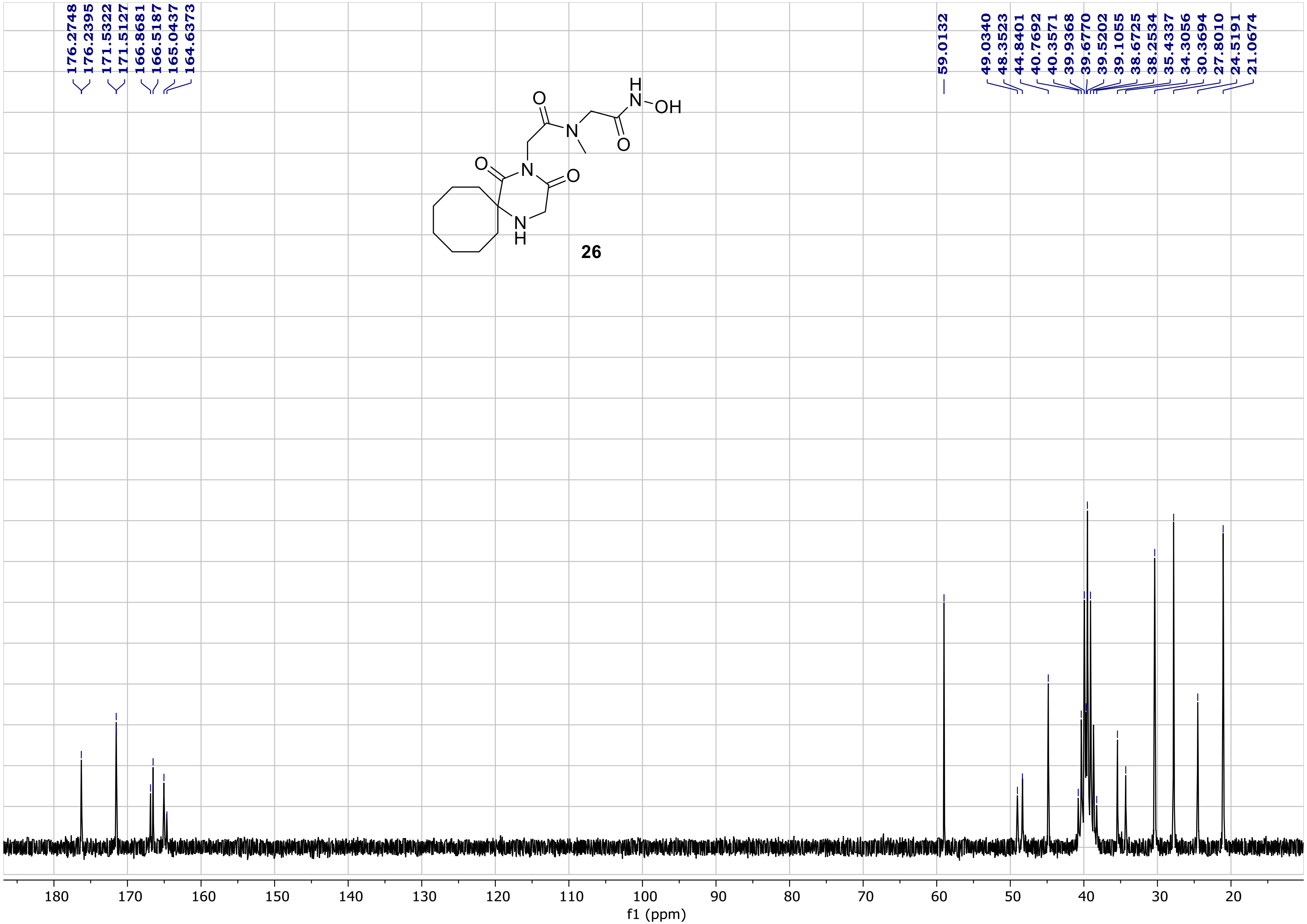

COSY NMR of **26** (600.11 MHz, DMSO-d_6)

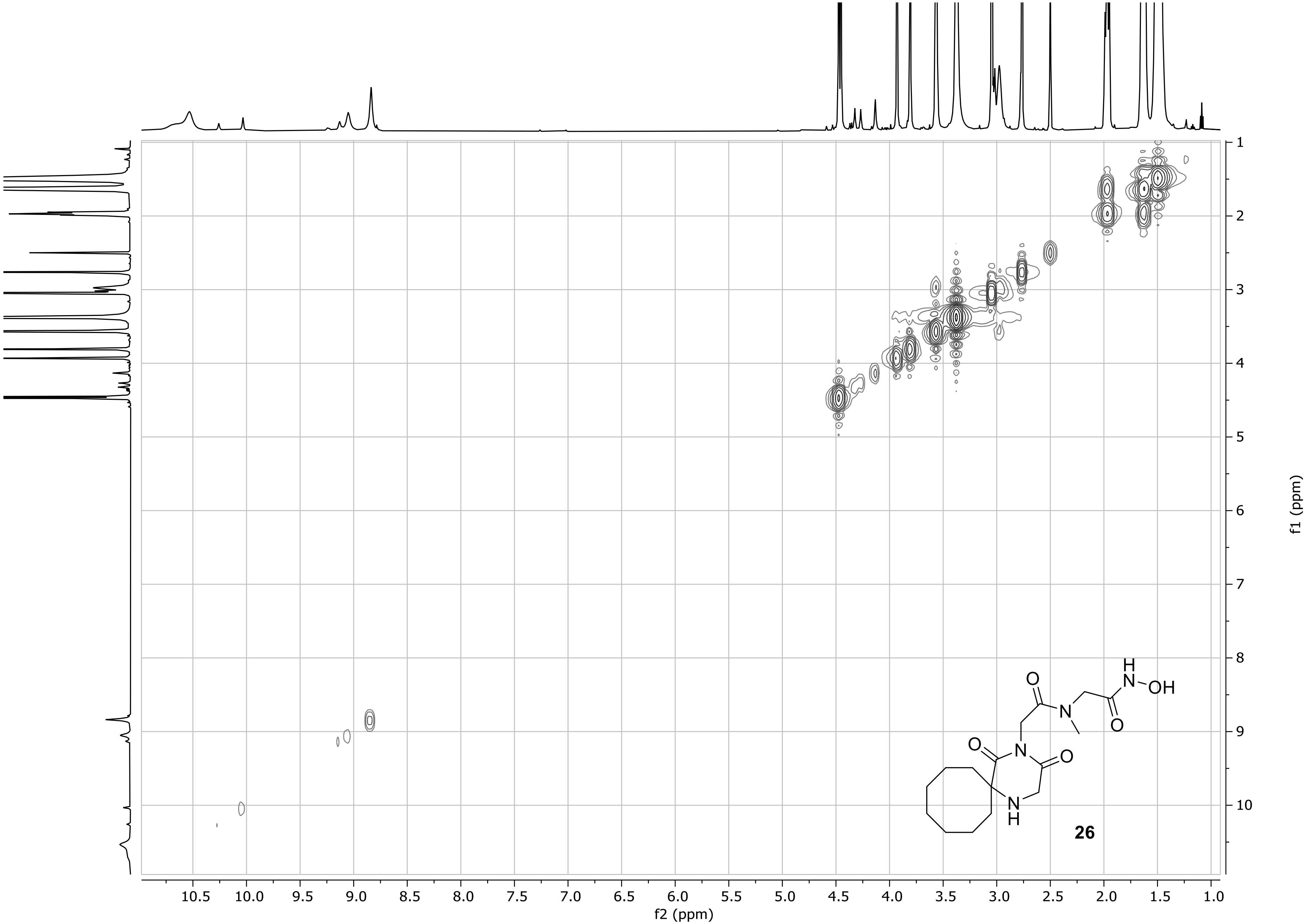

HSQC-DEPT NMR of **26** (600.11 MHz, DMSO-d_6)

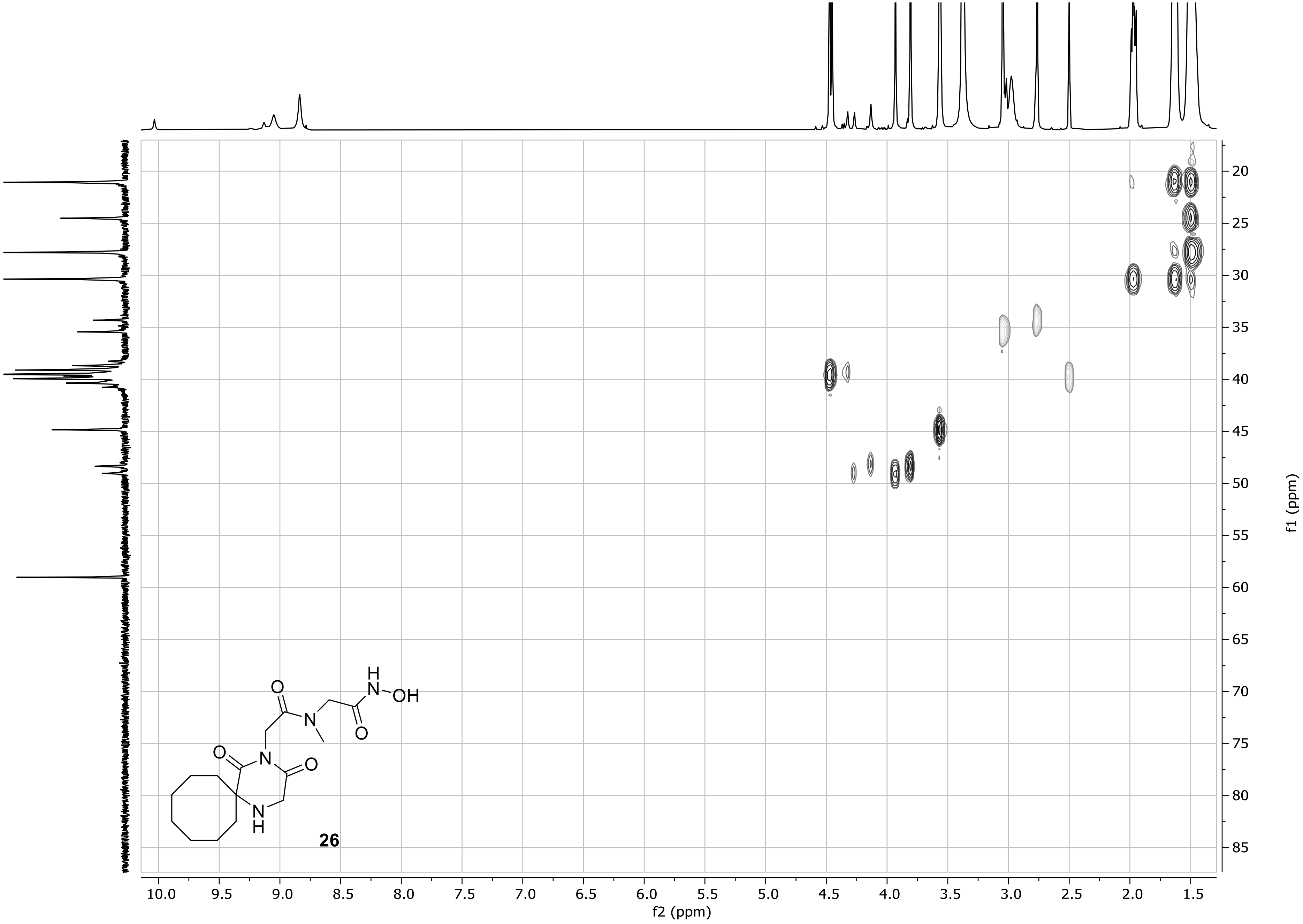

DEPT NMR of **26** (150.9 MHz, DMSO-d_6)

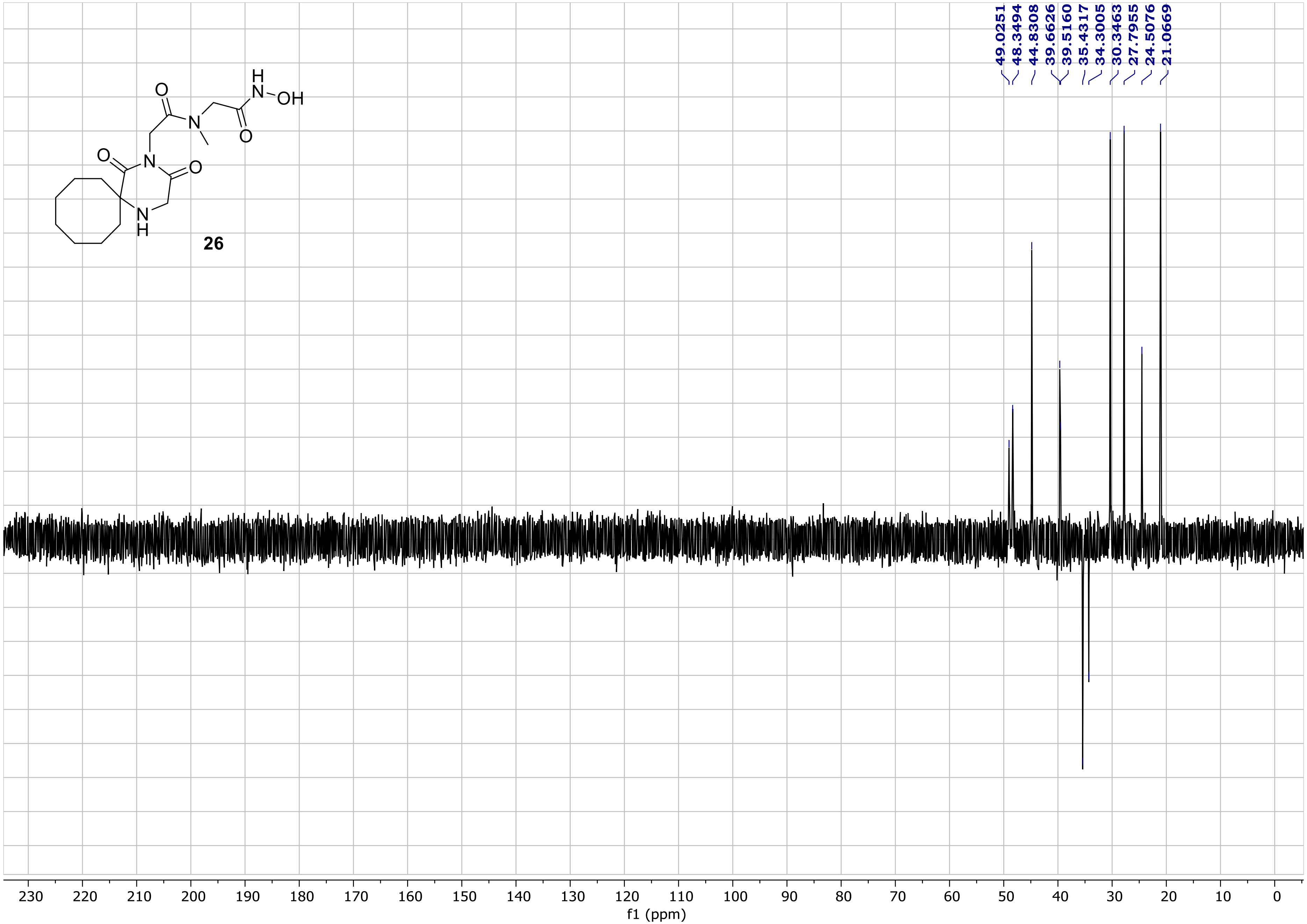

^{1}H NMR of **27** (400.13 MHz, DMSO-d_6)

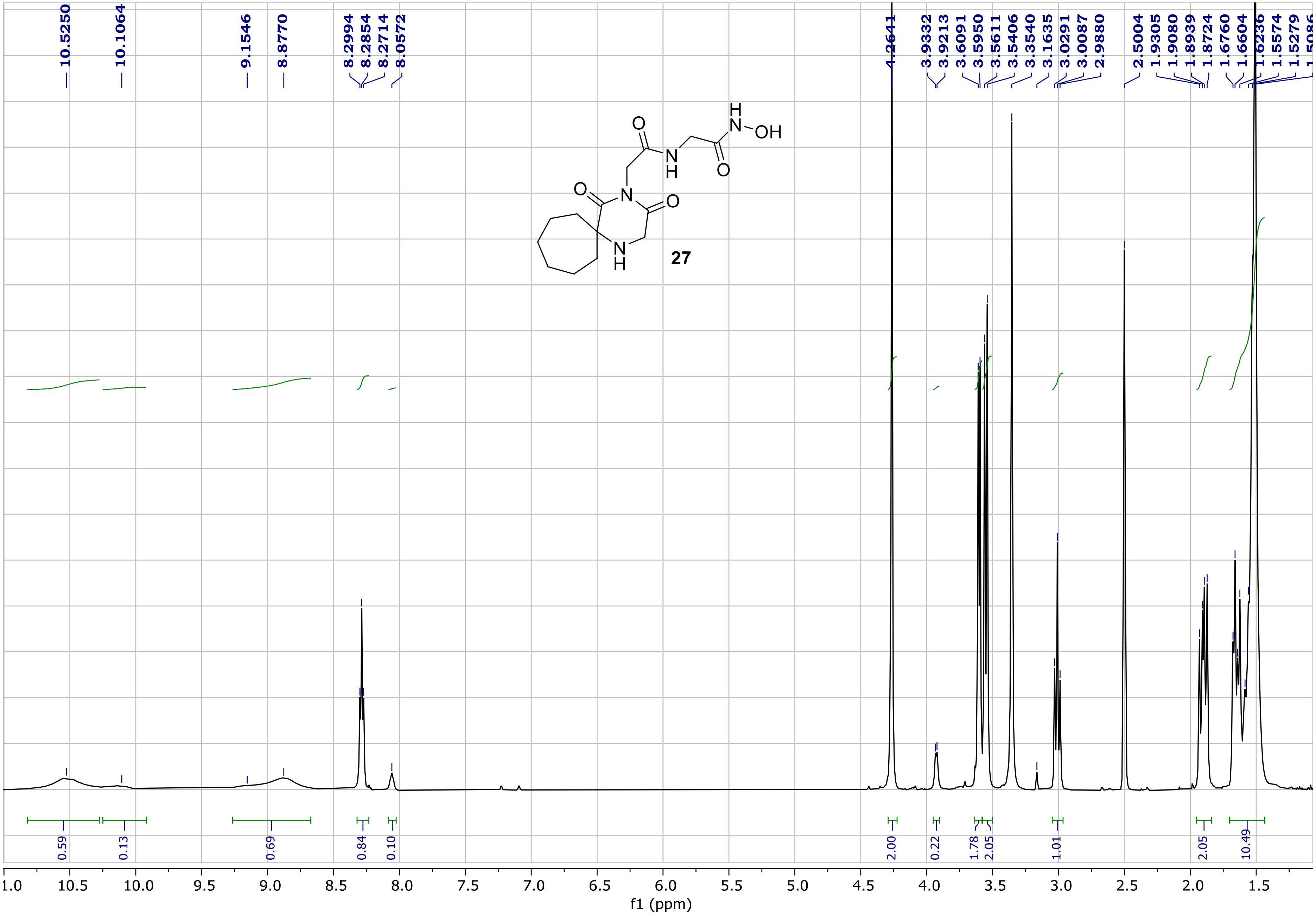

^{13}C NMR of **27** (50.32 MHz, DMSO-d_6)

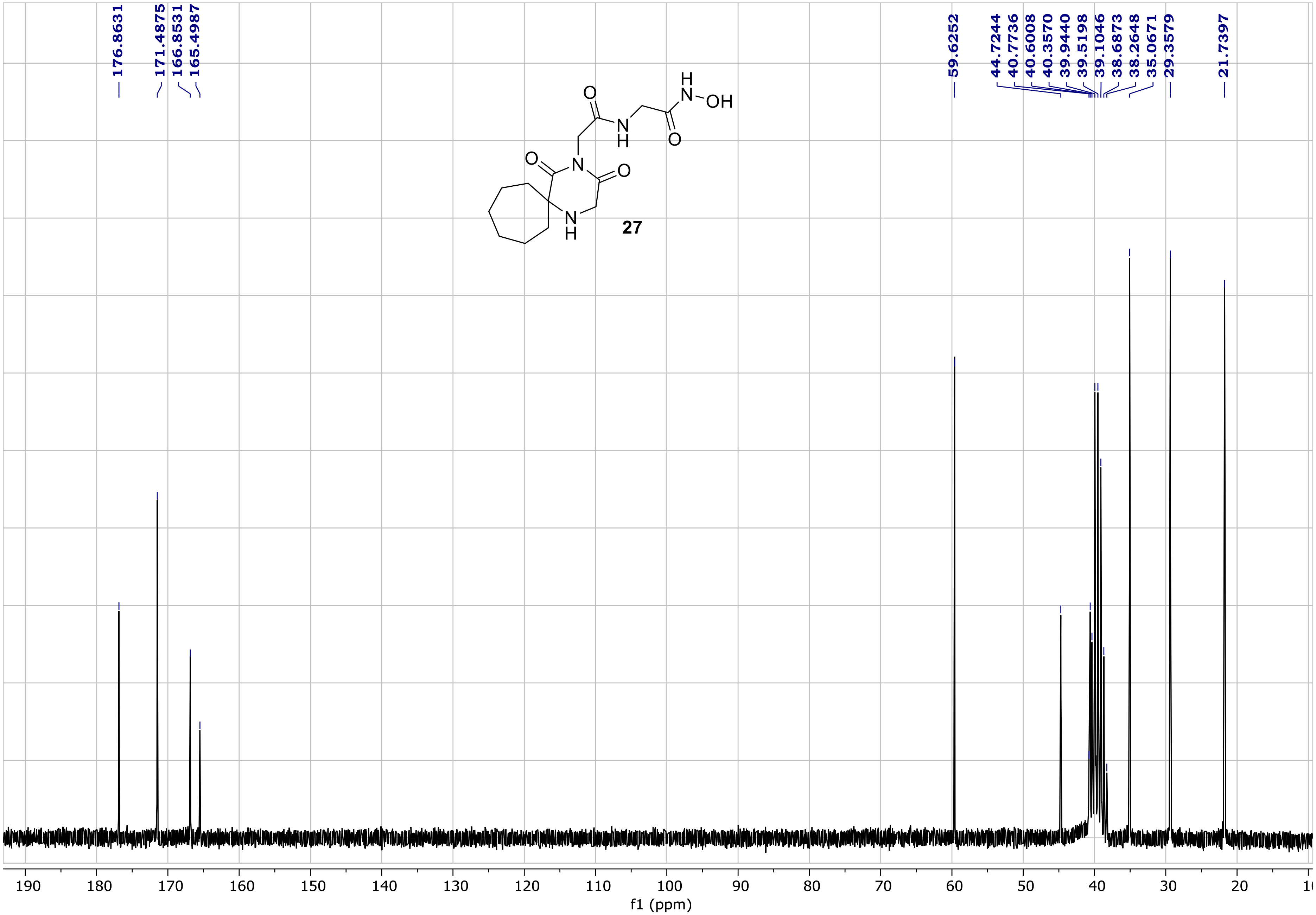

COSY NMR of **27** (400.13 MHz, DMSO-d_6)

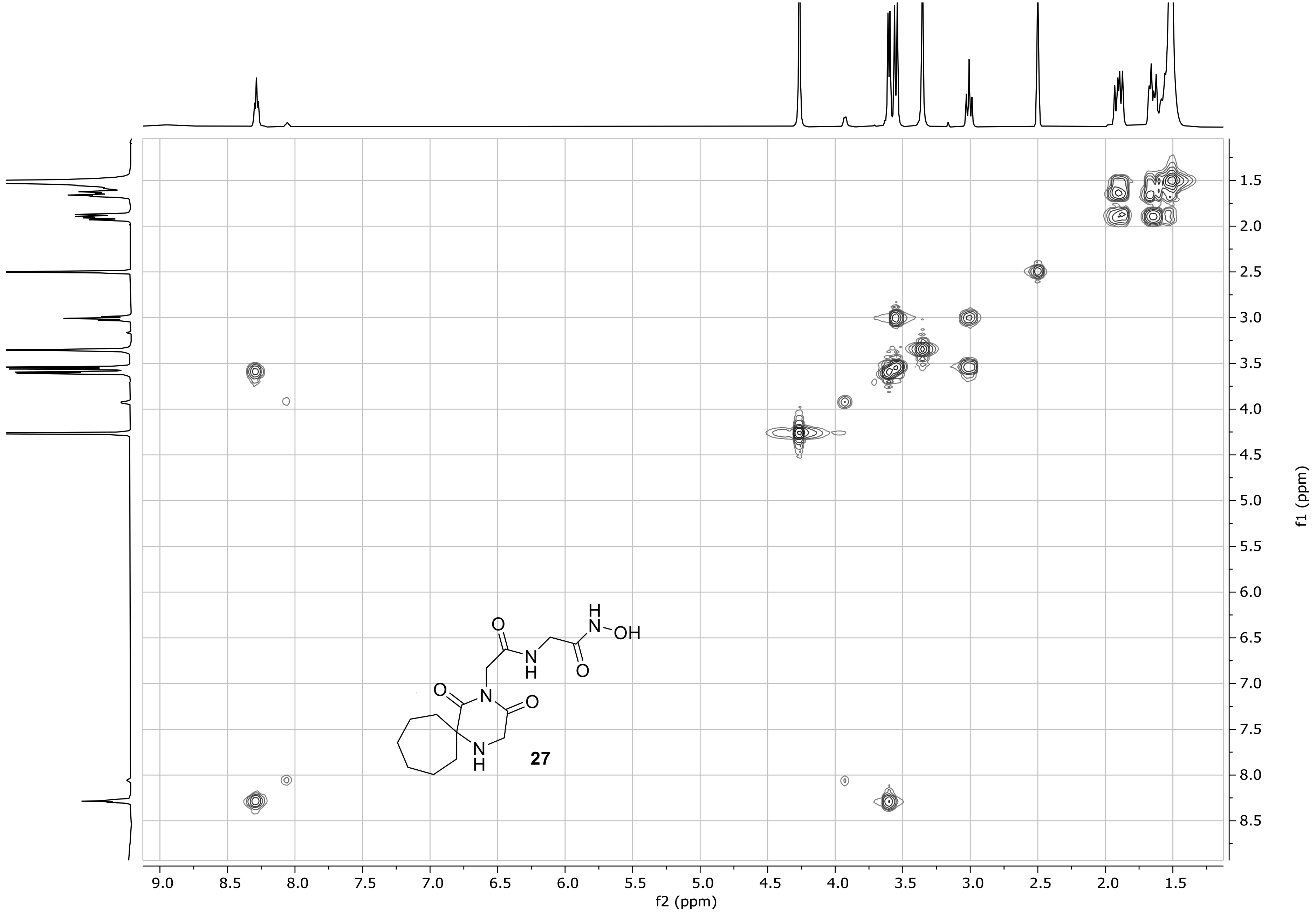

DEPT NMR of **27** (50.32 MHz, DMSO-d_6)

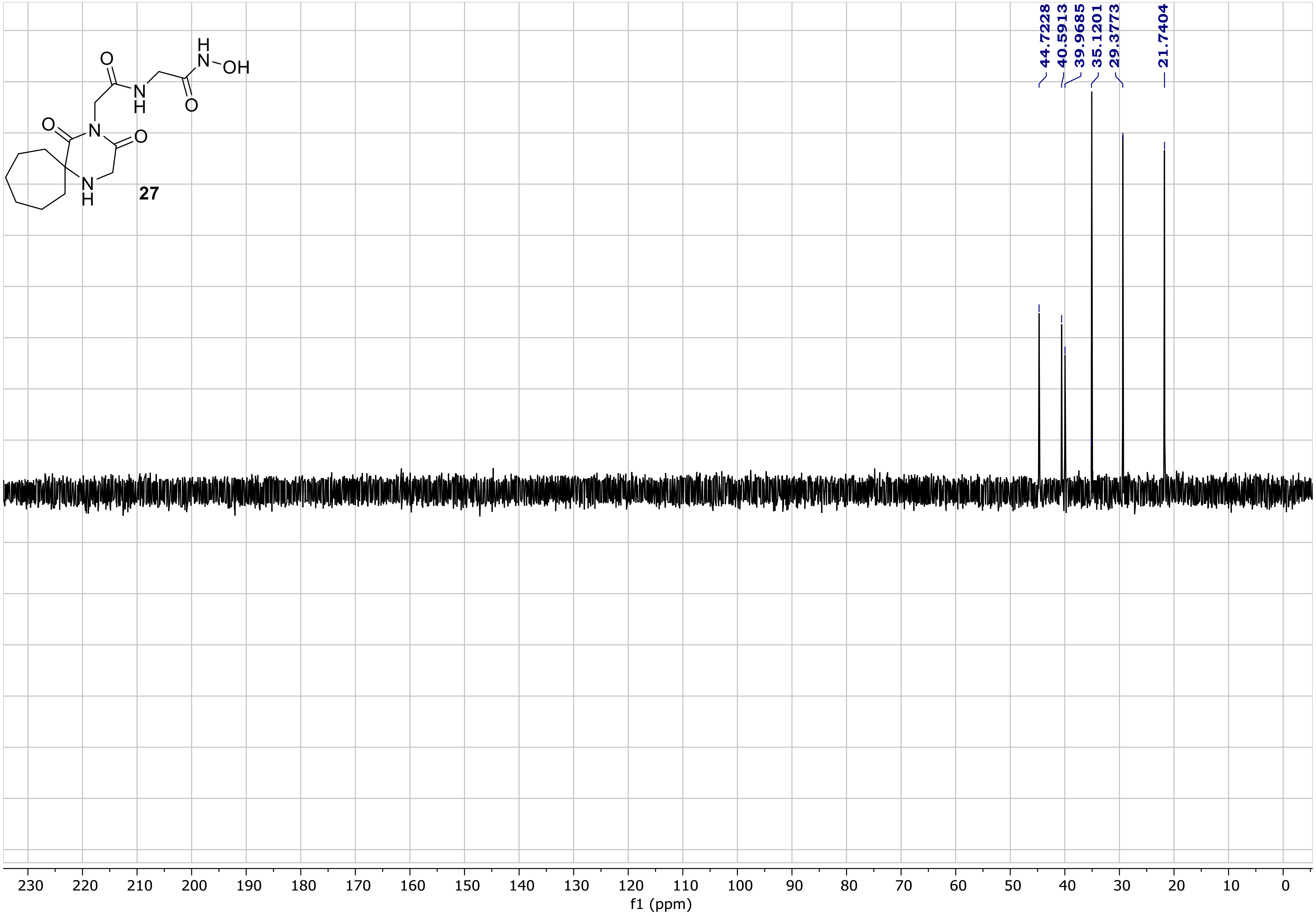

^{1}H NMR of **28** (400.13 MHz, DMSO-d_6)

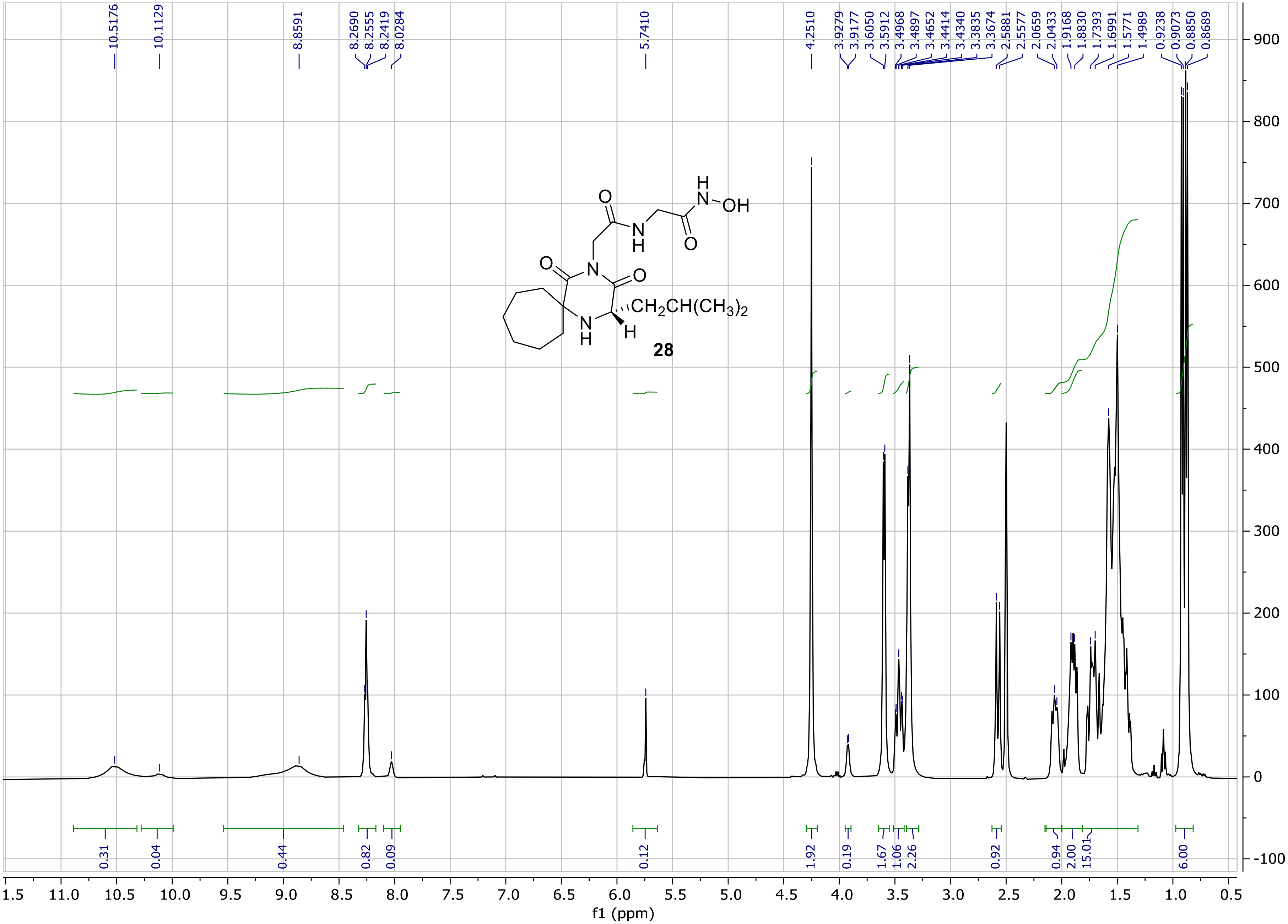

^{13}C NMR of **28** (50.32 MHz, DMSO-d_6)

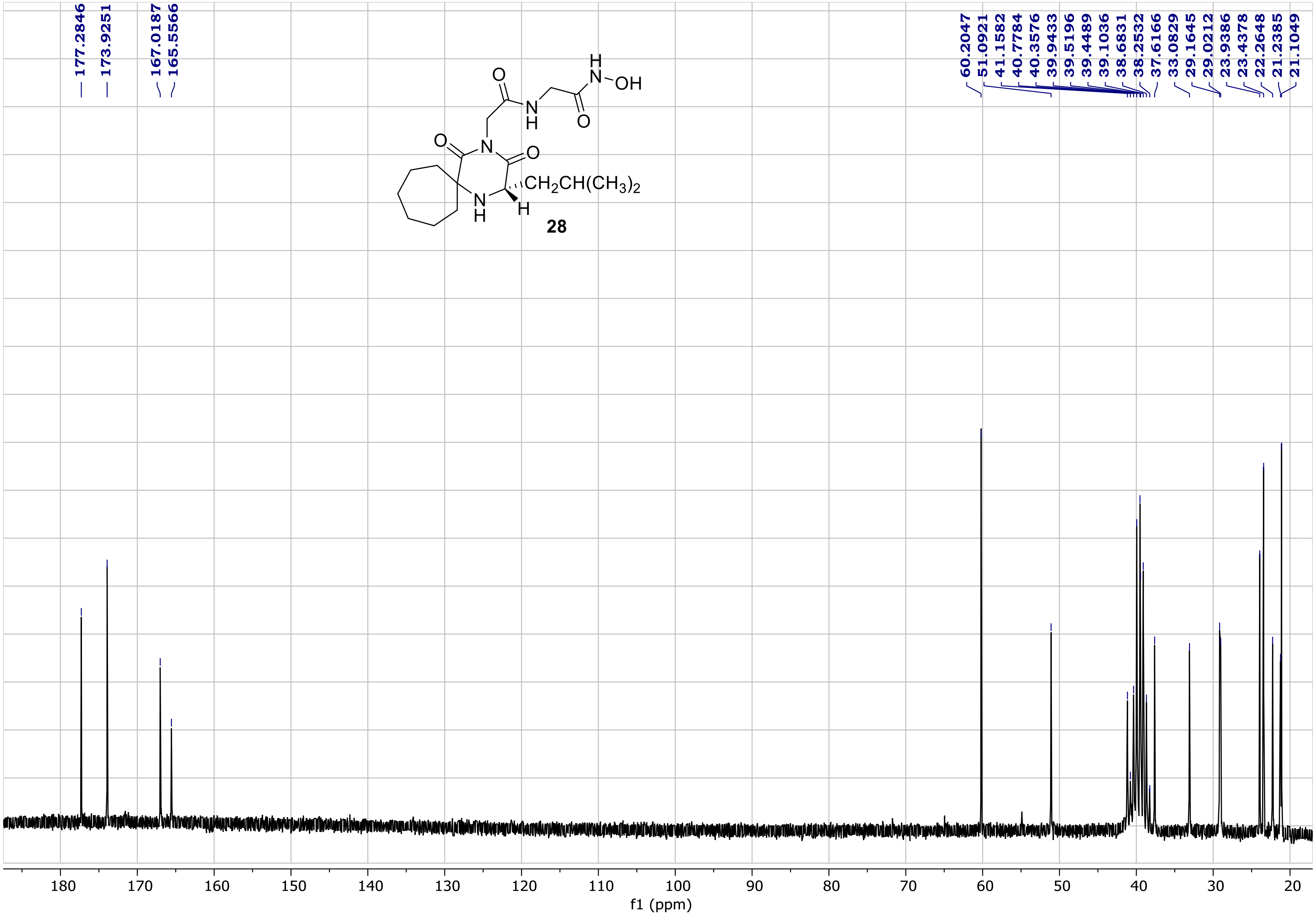

COSY NMR of **28** (400.13 MHz, DMSO-d_6)

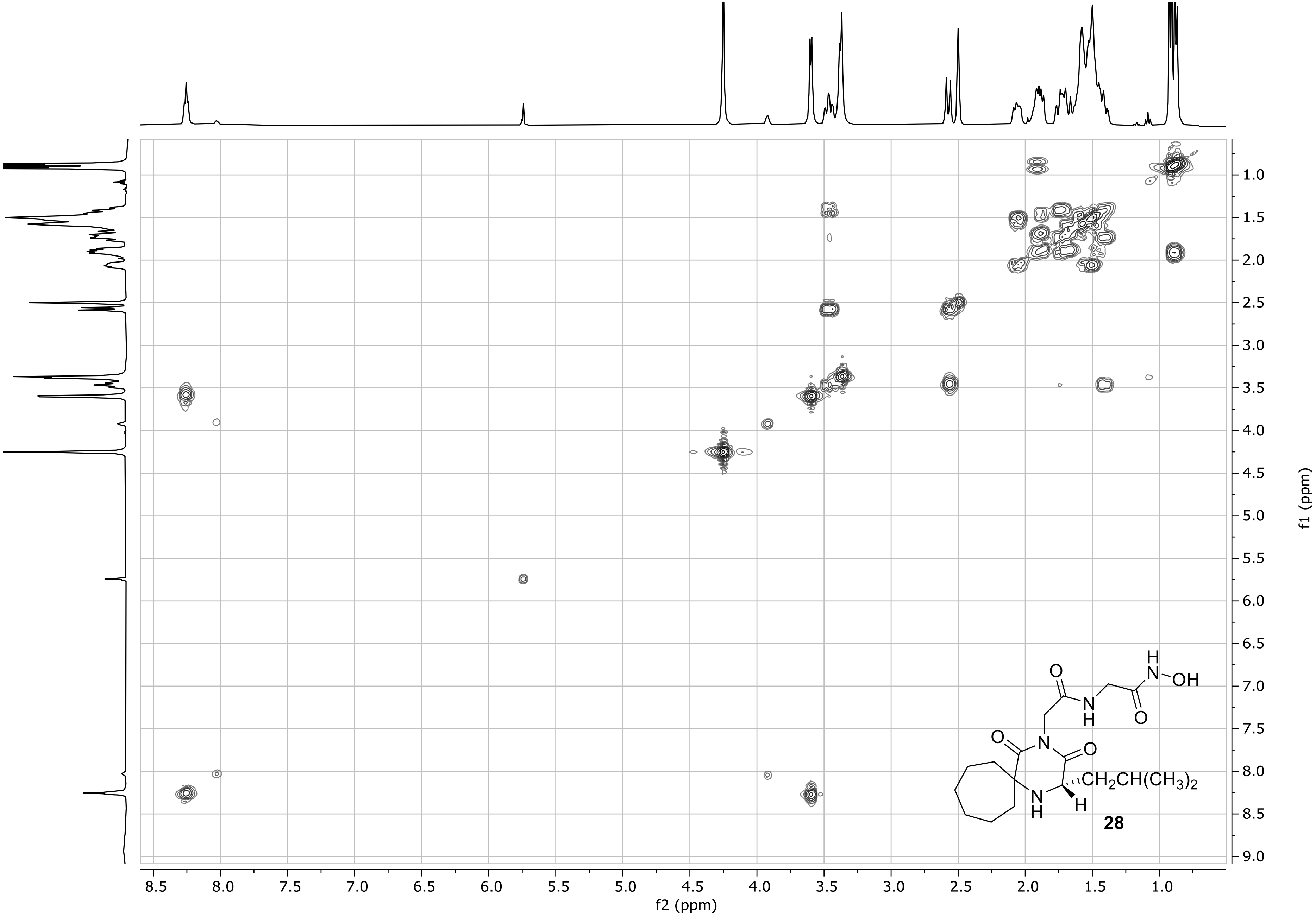

HSQC NMR of **28** (400.13 MHz, DMSO-d_6)

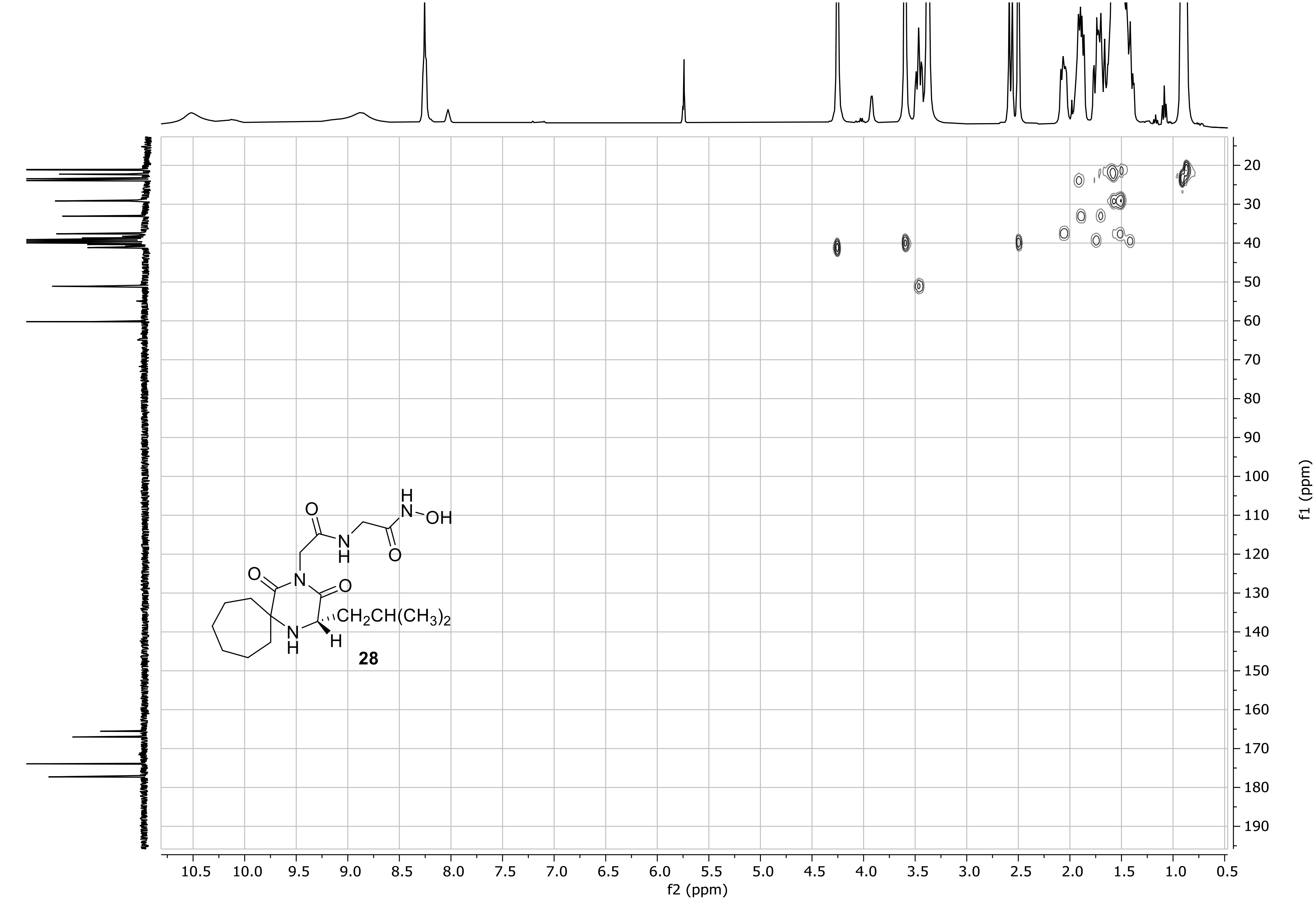

DEPT NMR of **28** (50.32 MHz, DMSO-d_6)

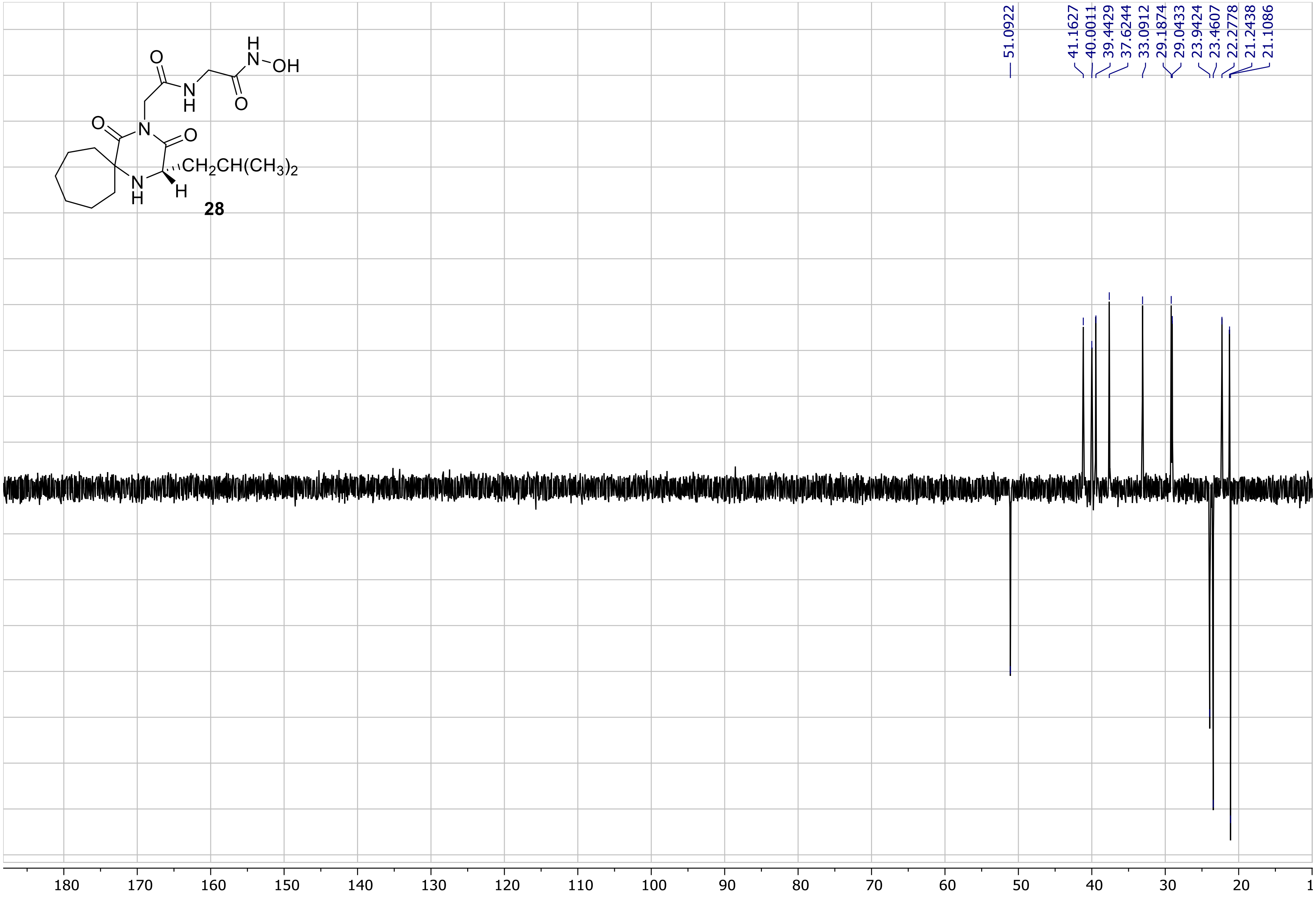

^{1}H NMR of **36** (400.13 MHz, DMSO-d_6)

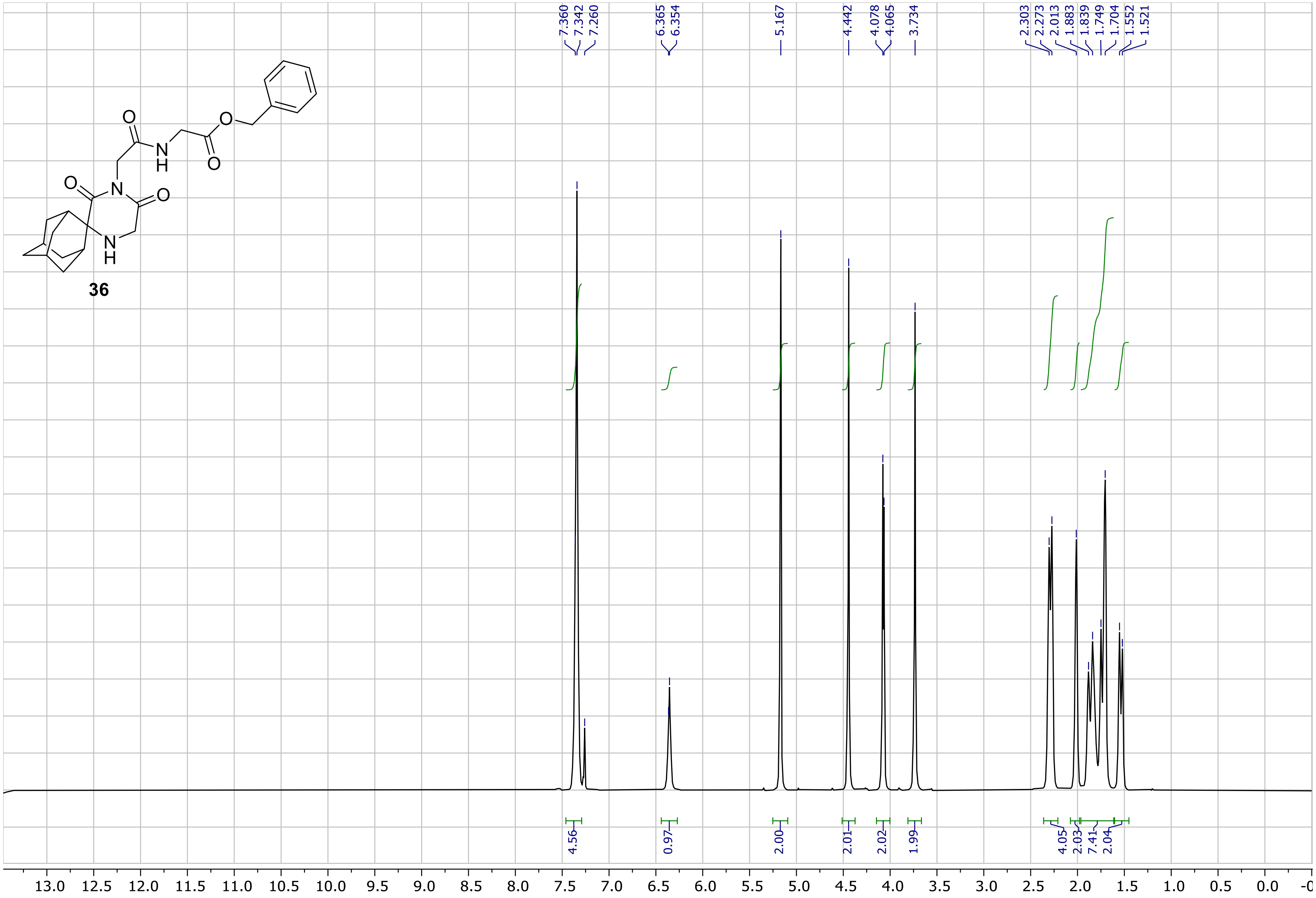

^{13}C NMR of **36** (50.32 MHz, DMSO-d_6)

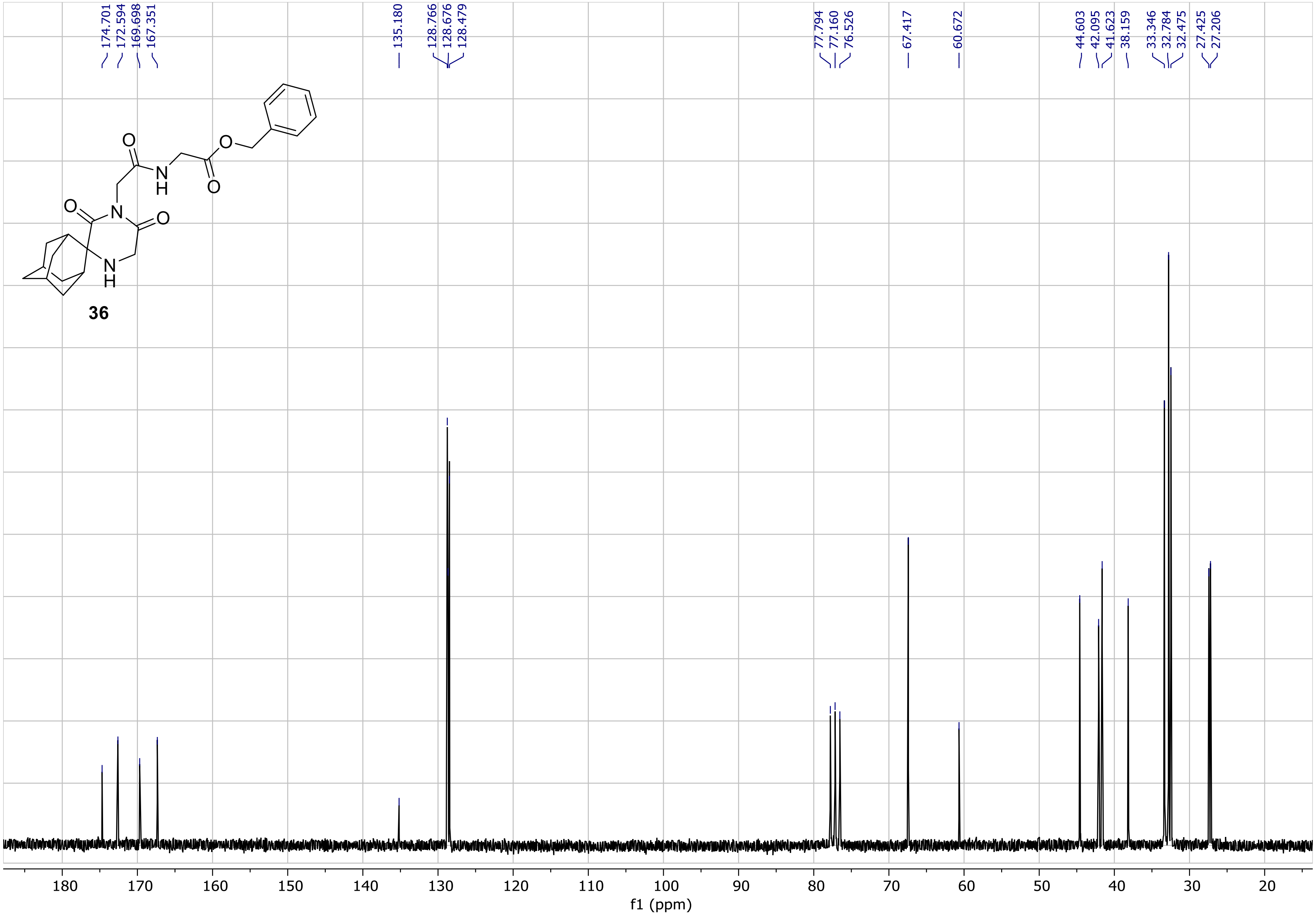

COSY NMR of **36** (400.13 MHz, DMSO-d_6)

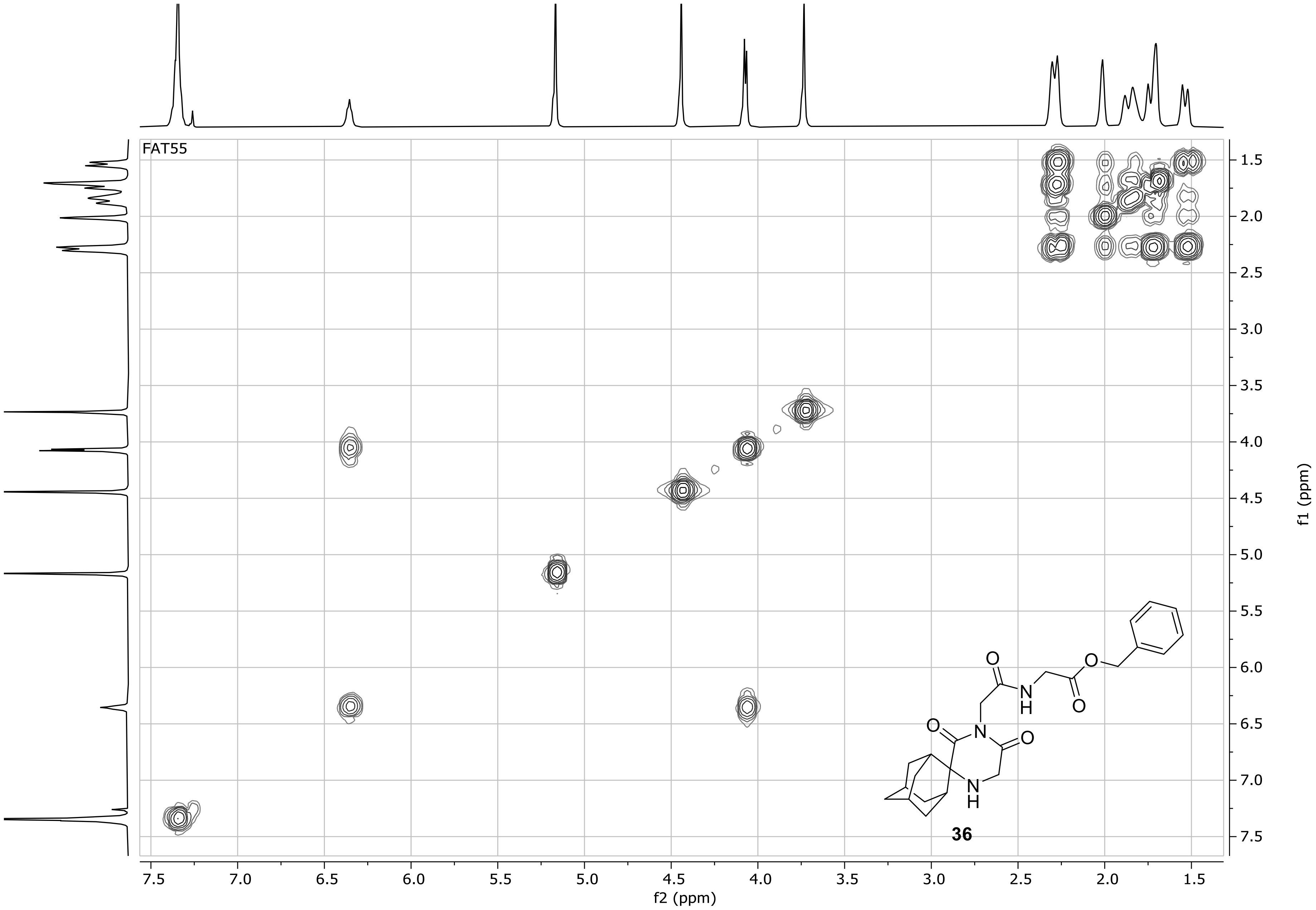

HSQC NMR of **36** (400.13 MHz, DMSO-d_6)

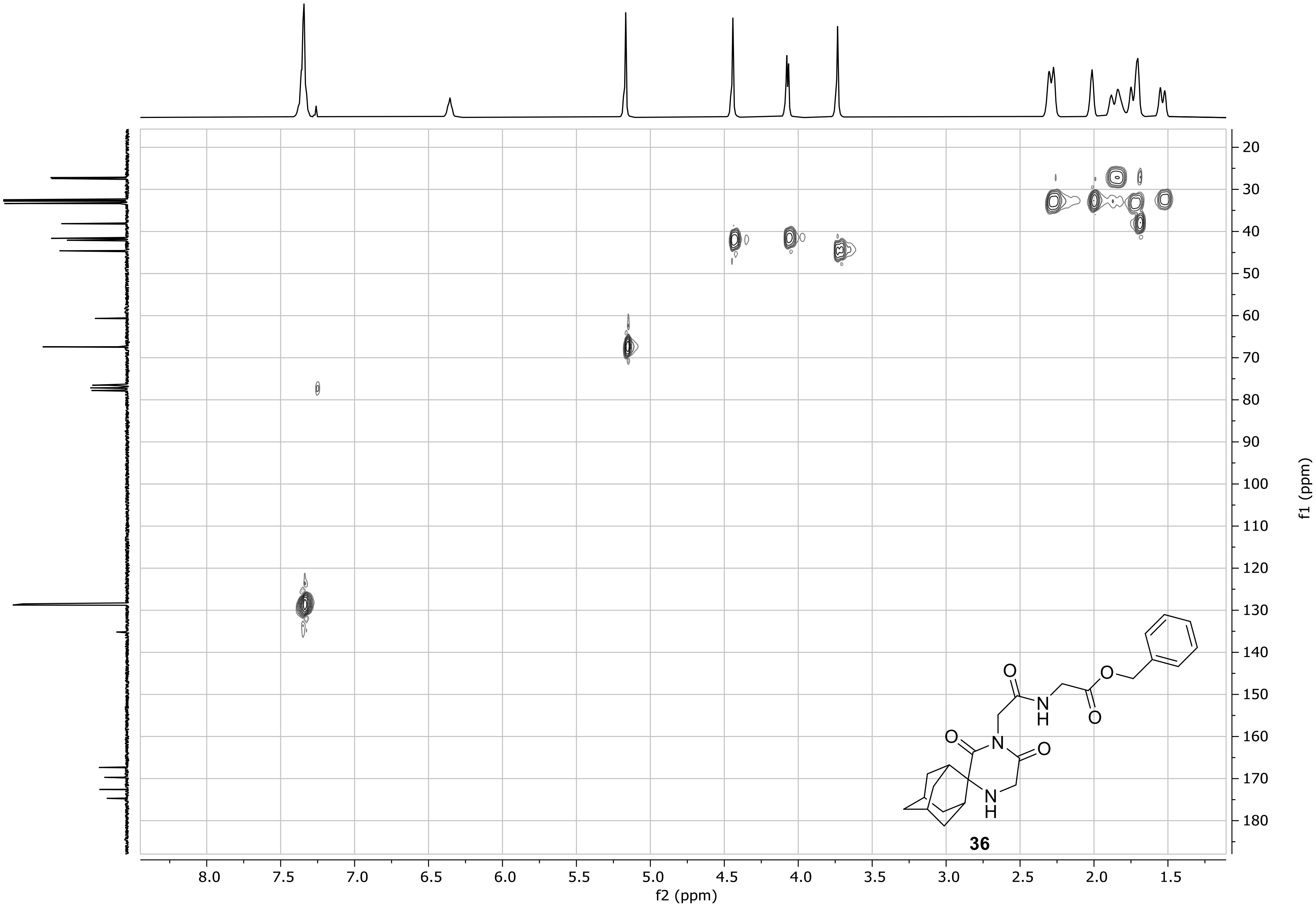

DEPT NMR of **36** (50.32 MHz, DMSO-d_6)

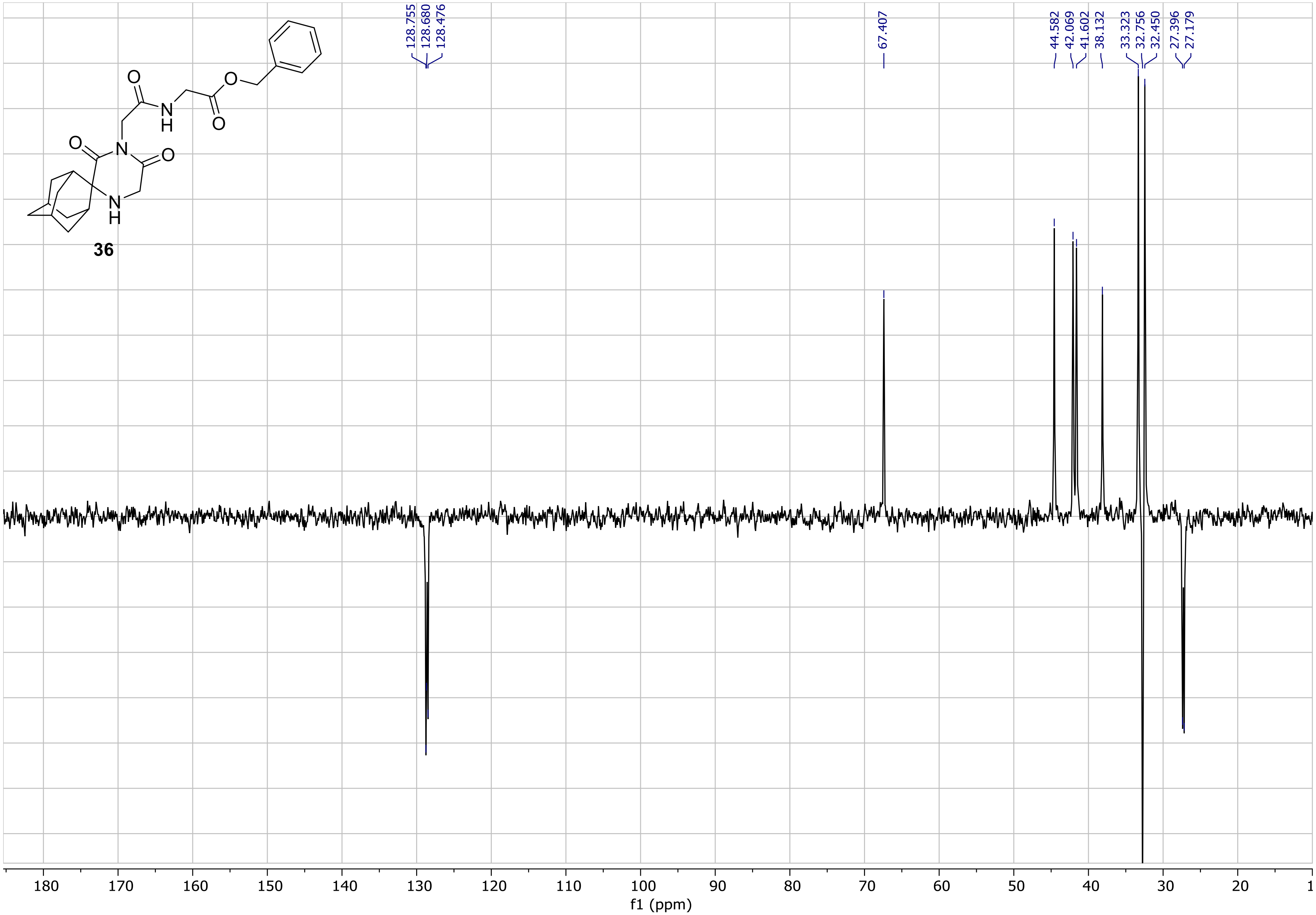

^{1}H NMR of **37** (600.11 MHz, DMSO-d_6)

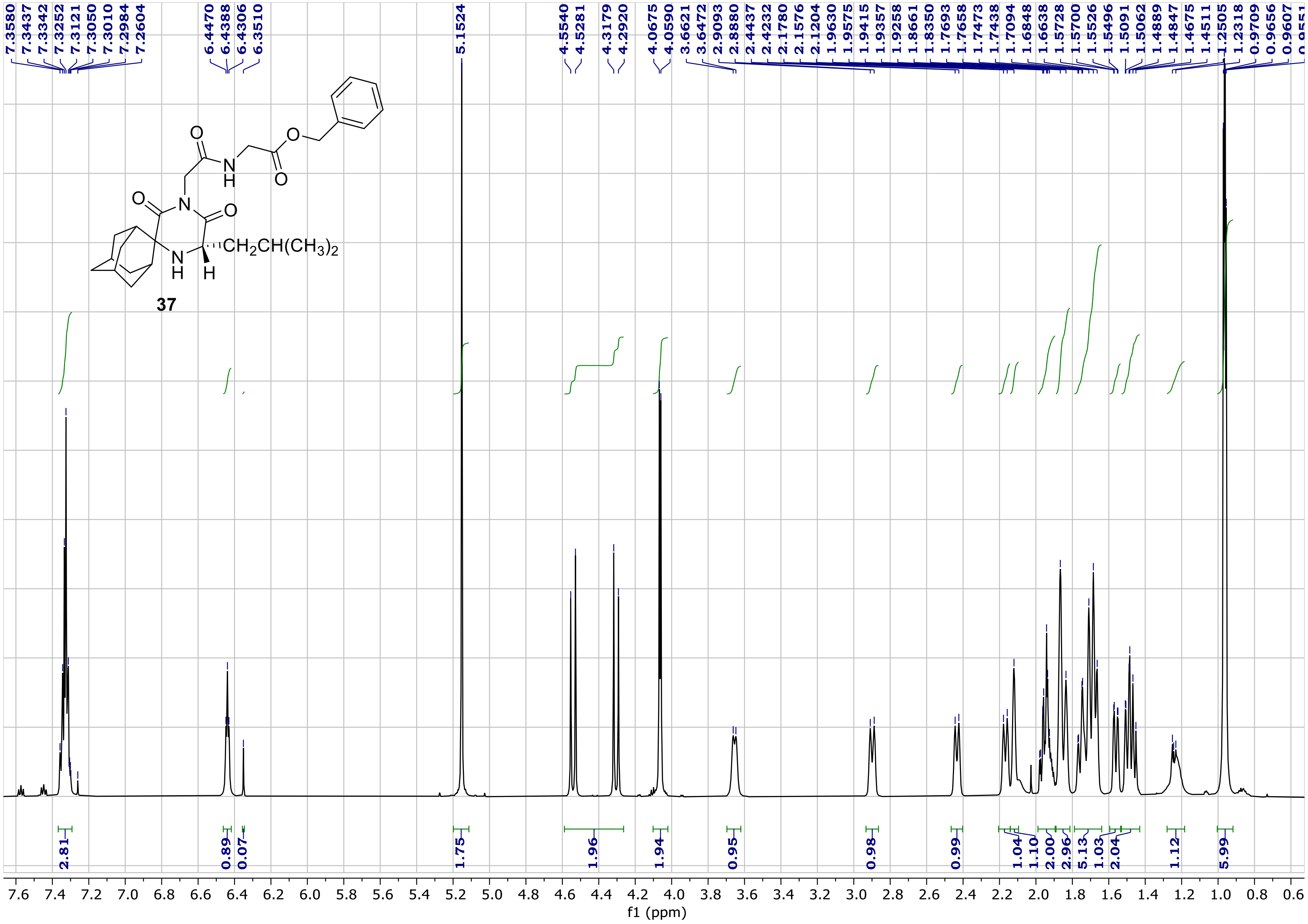

^{13}C NMR of **37** (50.32 MHz, DMSO-d_6)

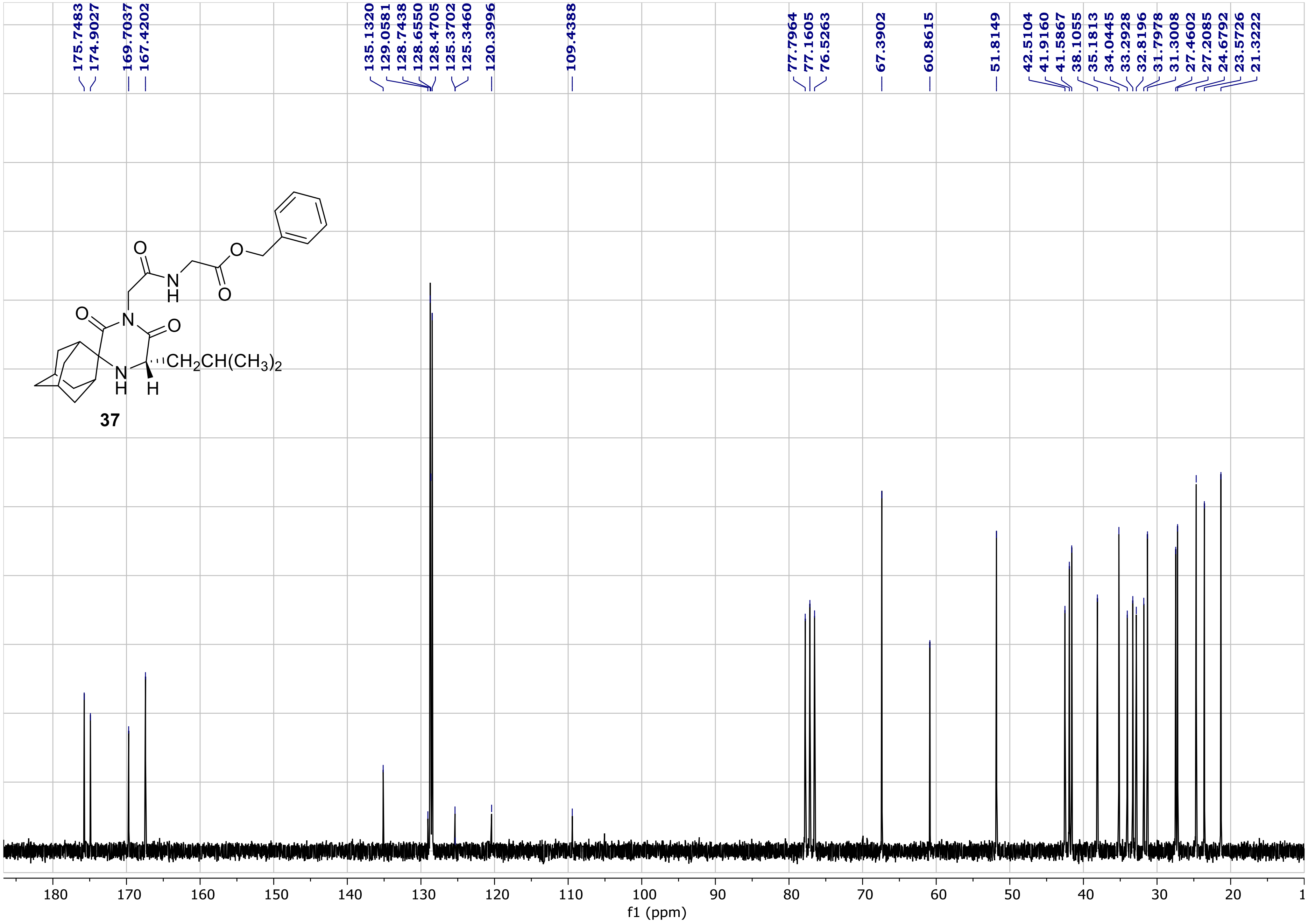

COSY NMR of **37** (400.13 MHz, DMSO-d_6)

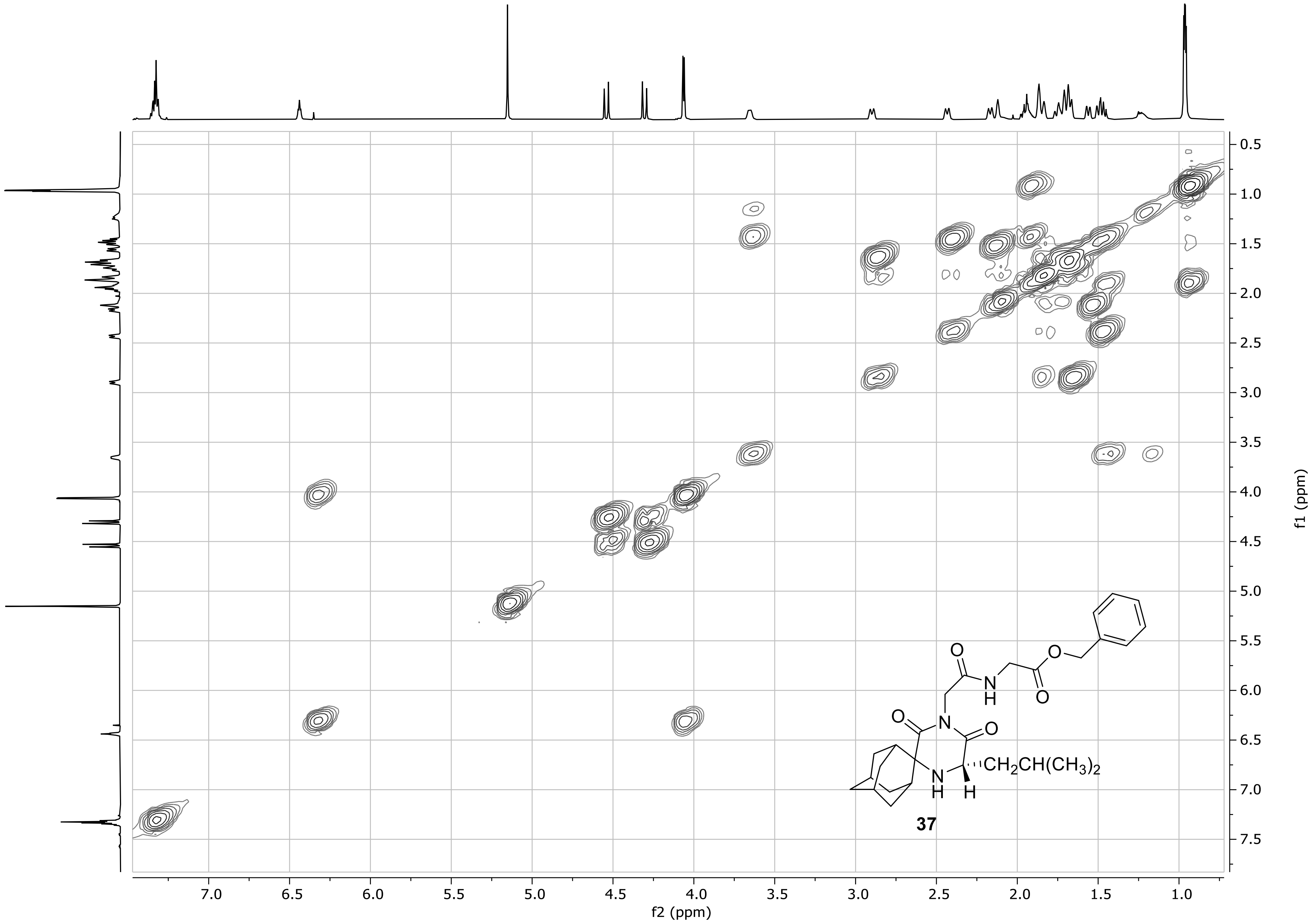

HSQC NMR of **37** (400.13 MHz, DMSO-d_6)

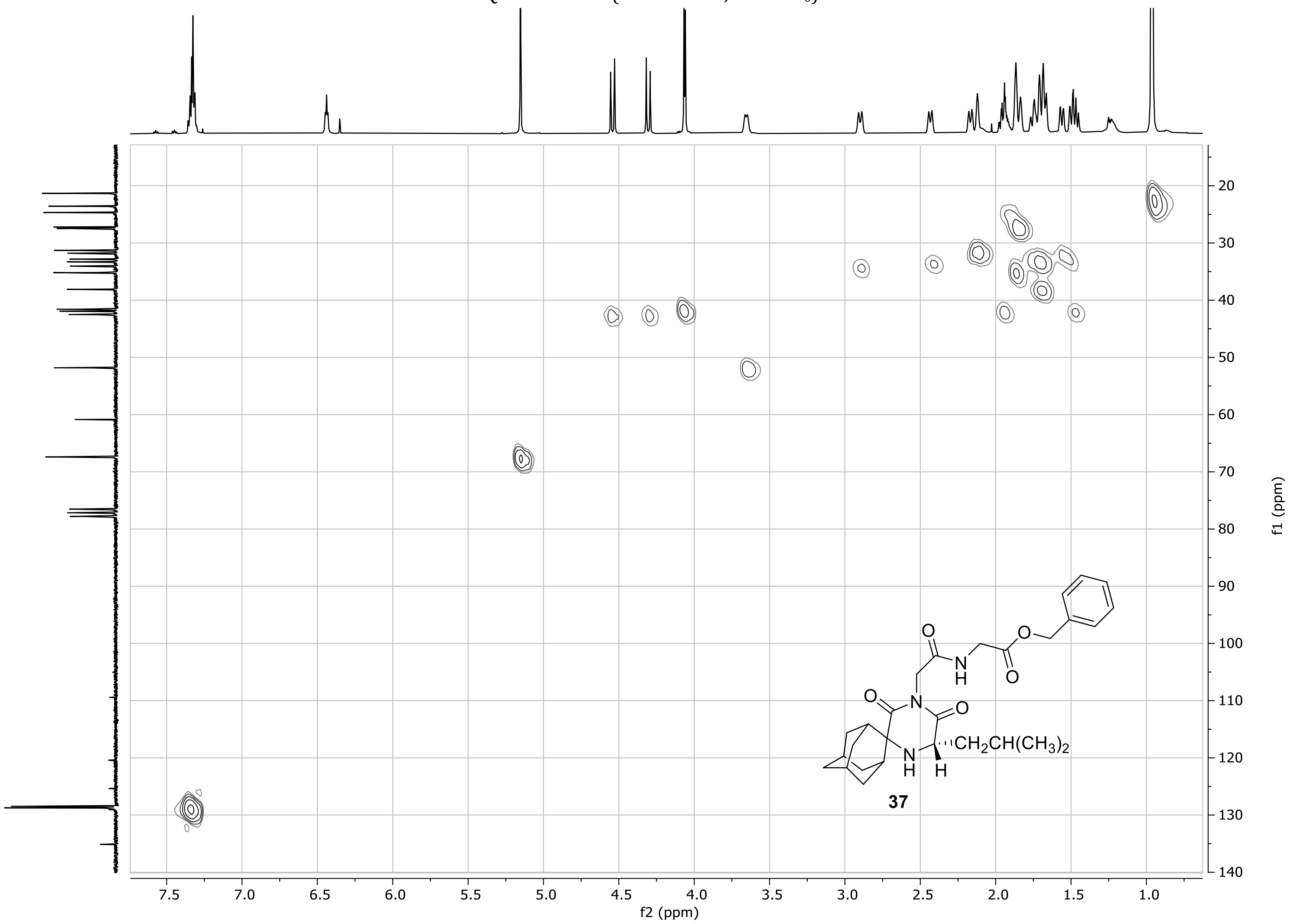

DEPT NMR of **37** (50.32 MHz, DMSO-d_6)

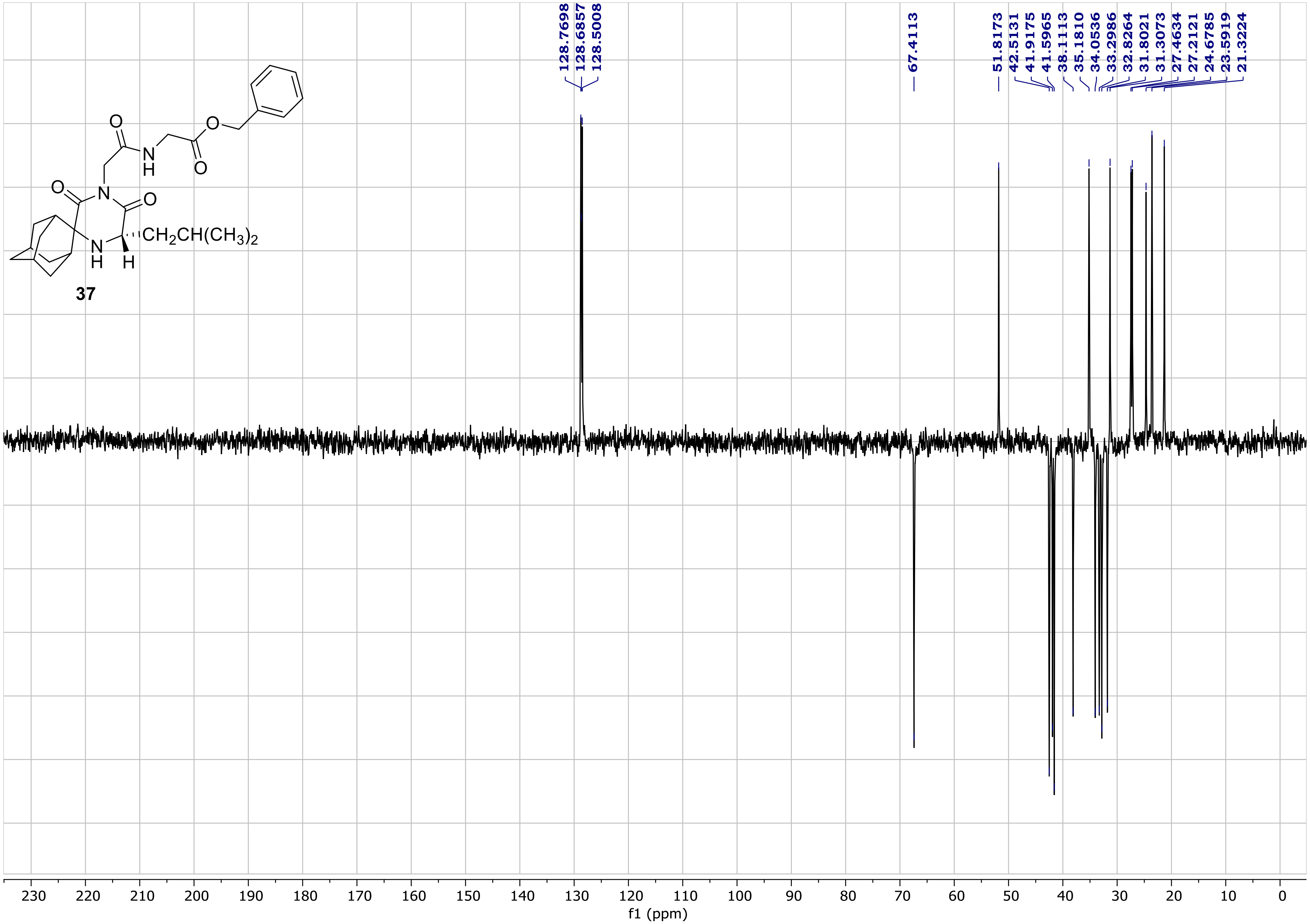

^{1}H NMR of **38** (600.11 MHz, DMSO-d_6)

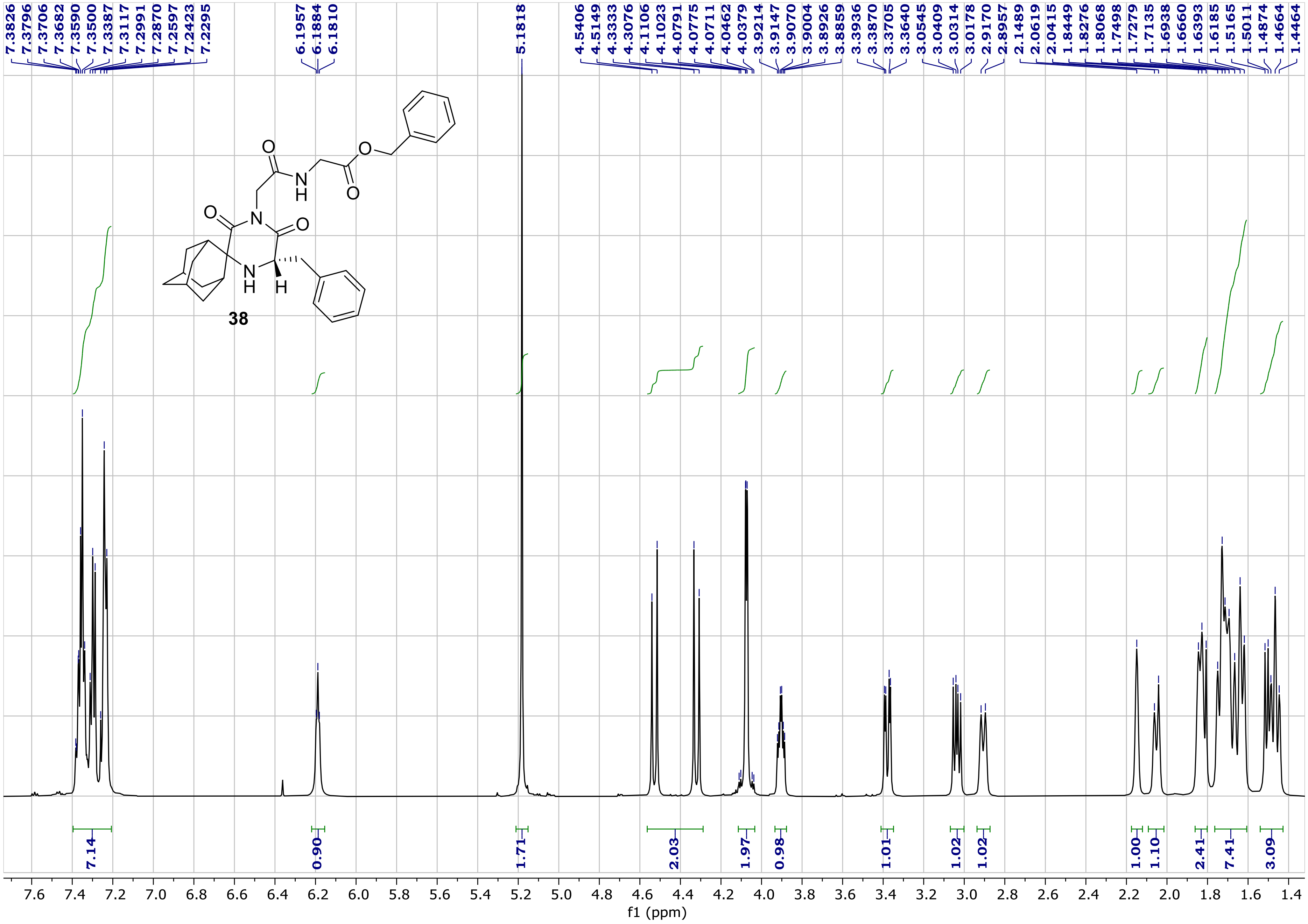

^{13}C NMR of **38** (50.32 MHz, DMSO-d_6)

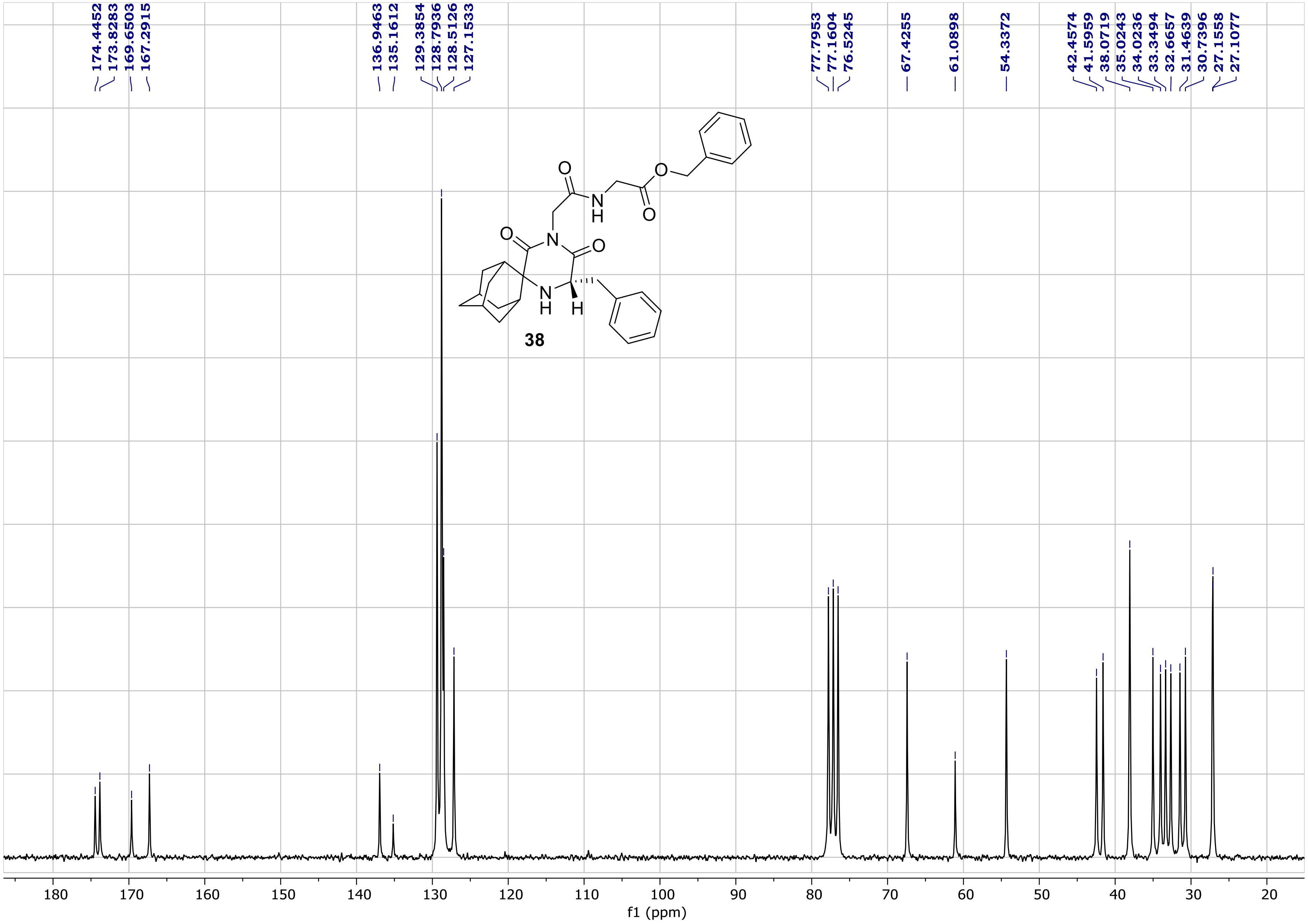

COSY NMR of **38** (400.13 MHz, DMSO-d_6)

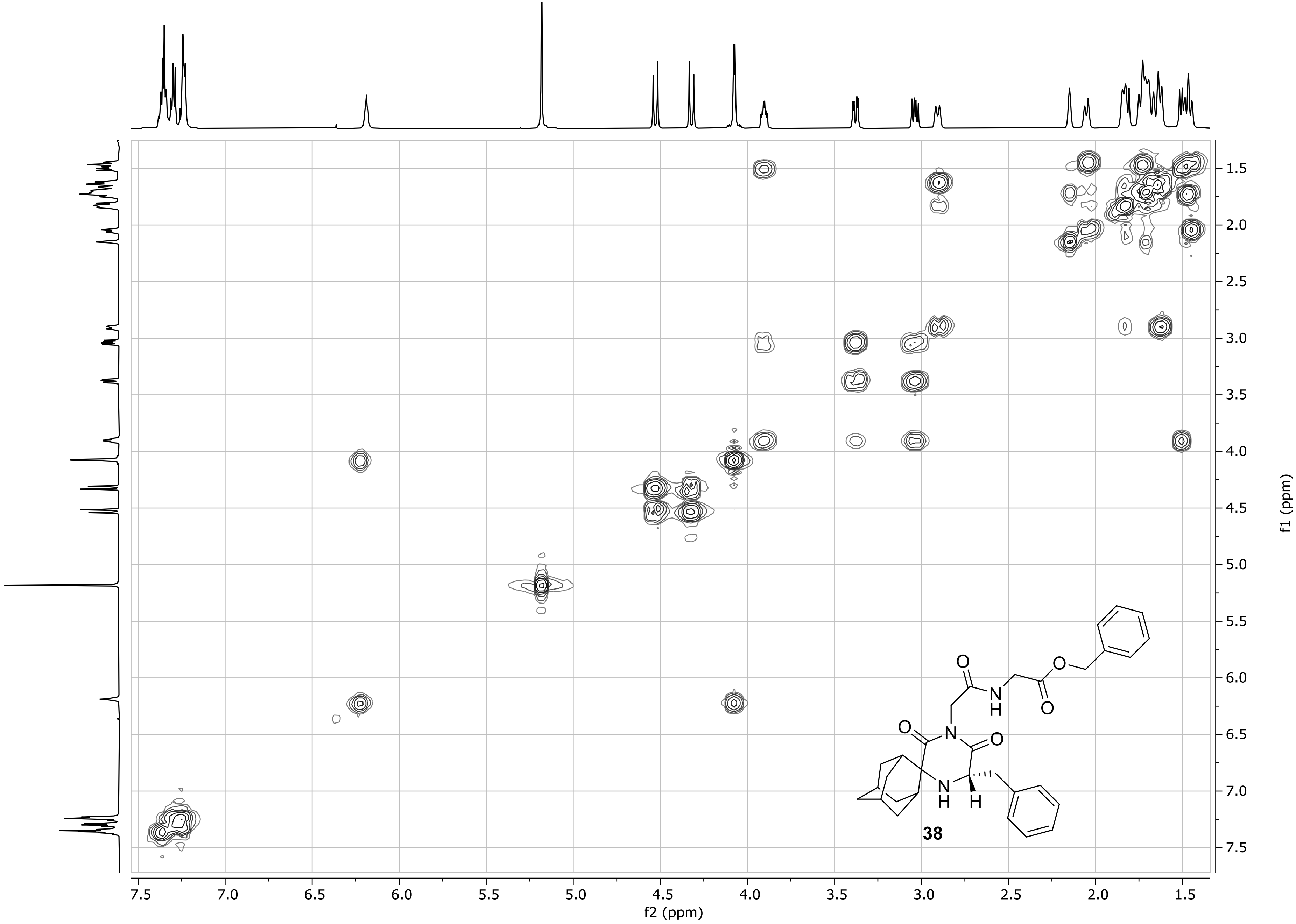

HSQC NMR of **38** (400.13 MHz, DMSO-d_6)

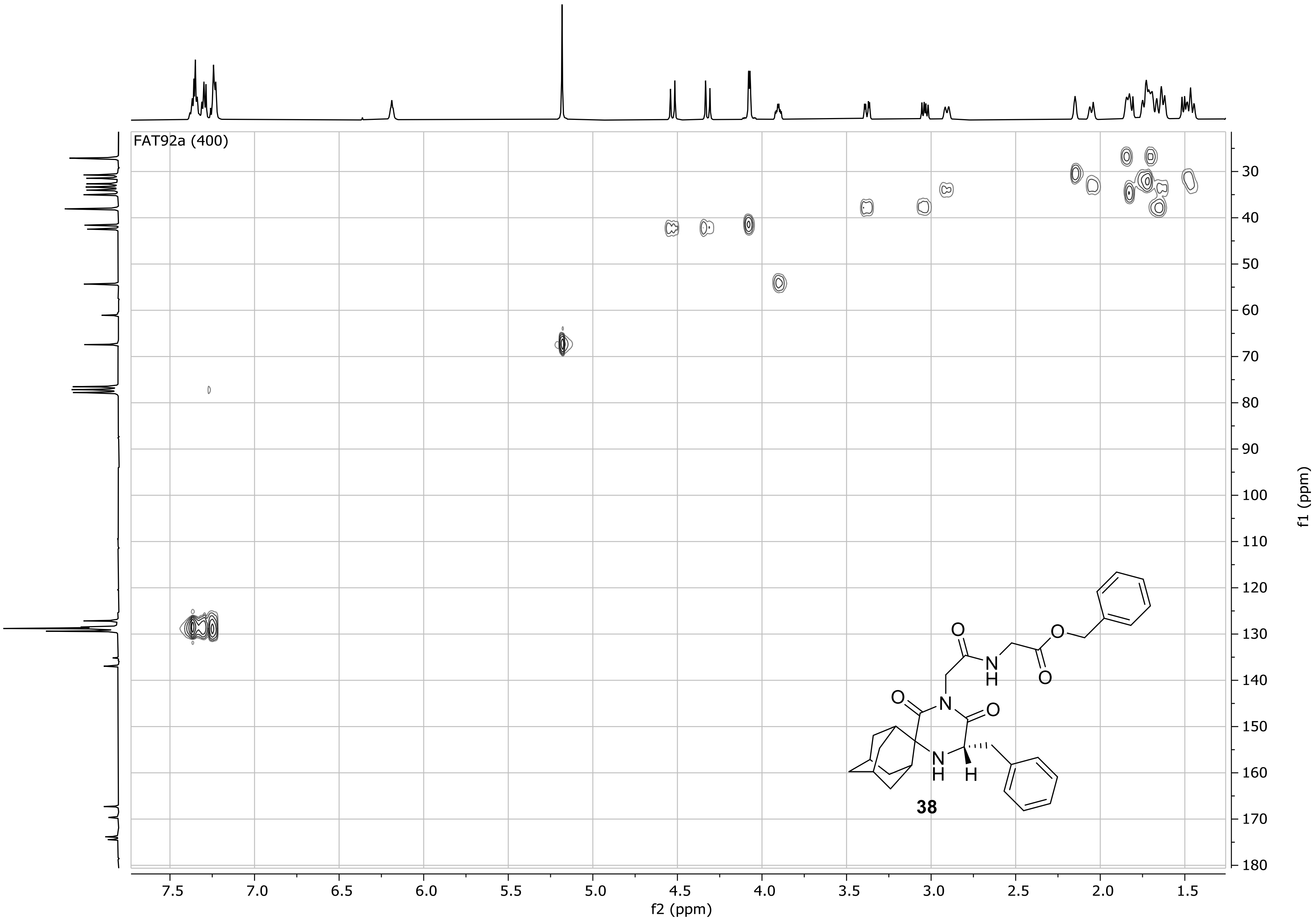

DEPT NMR of **38** (50.32 MHz, DMSO-d_6)

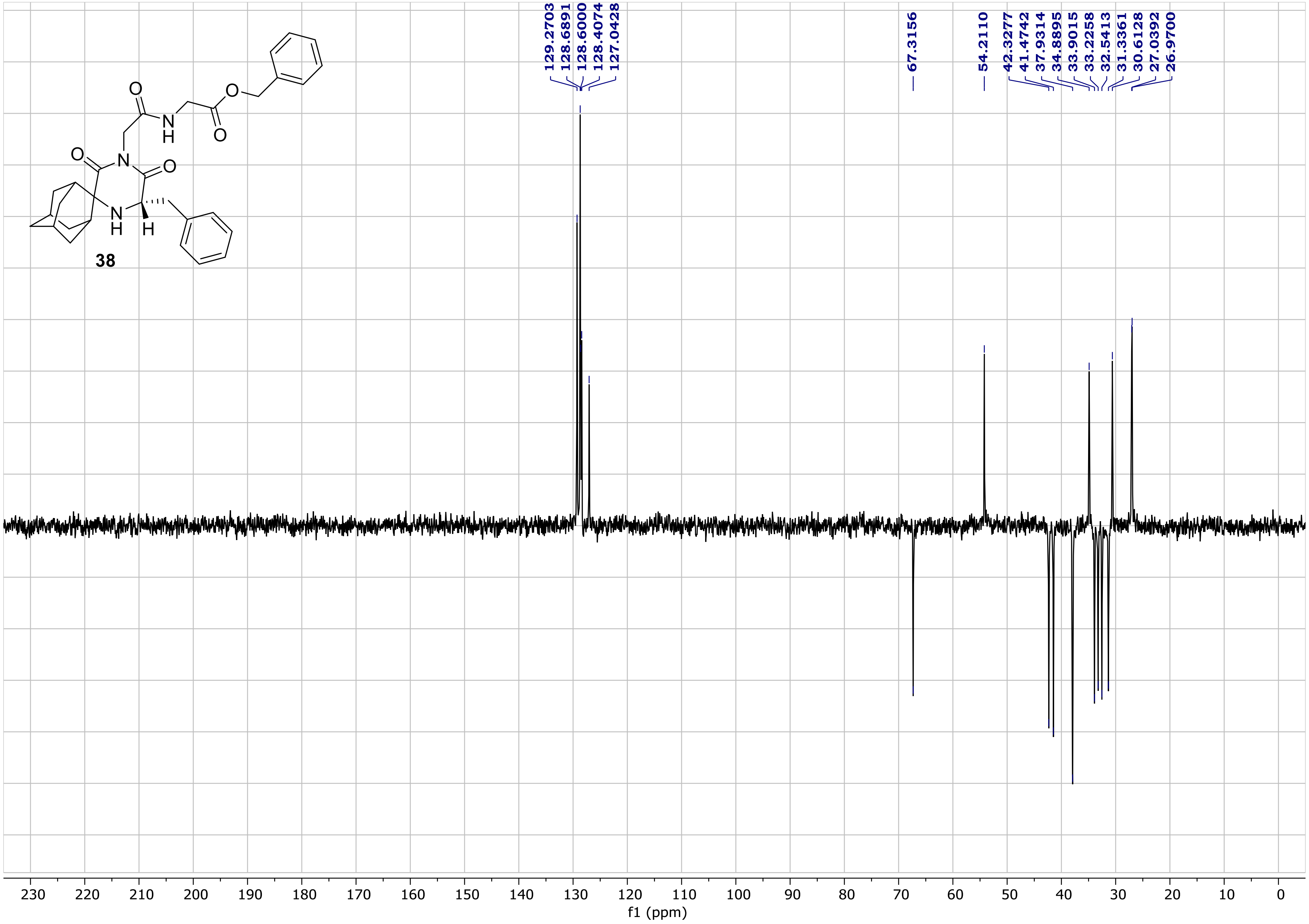

^{1}H NMR of **39** (600.11 MHz, DMSO-d_6)

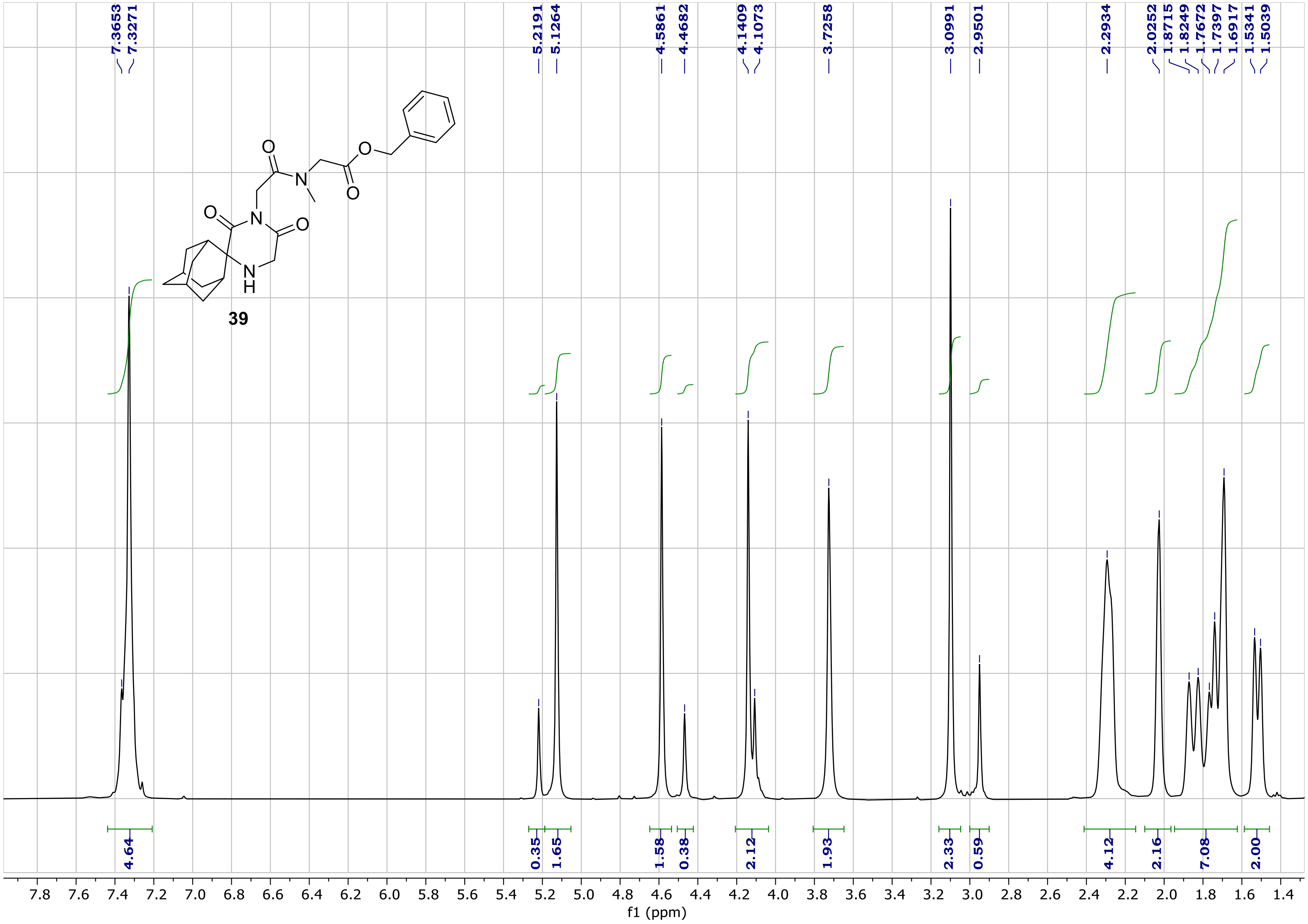

^{13}C NMR of **39** (50.32 MHz, DMSO-d_6)

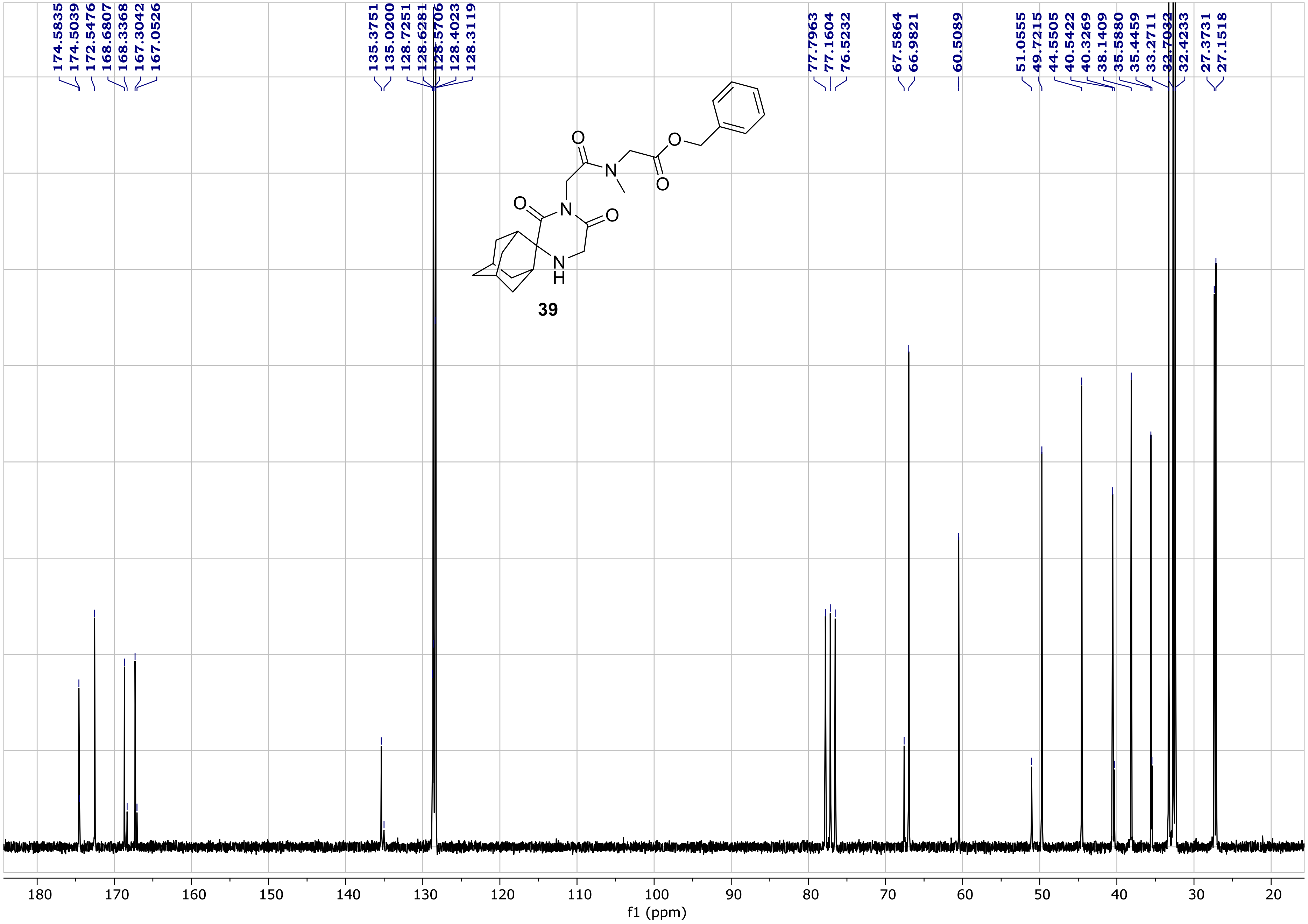

COSY NMR of **39** (400.13 MHz, DMSO-d_6)

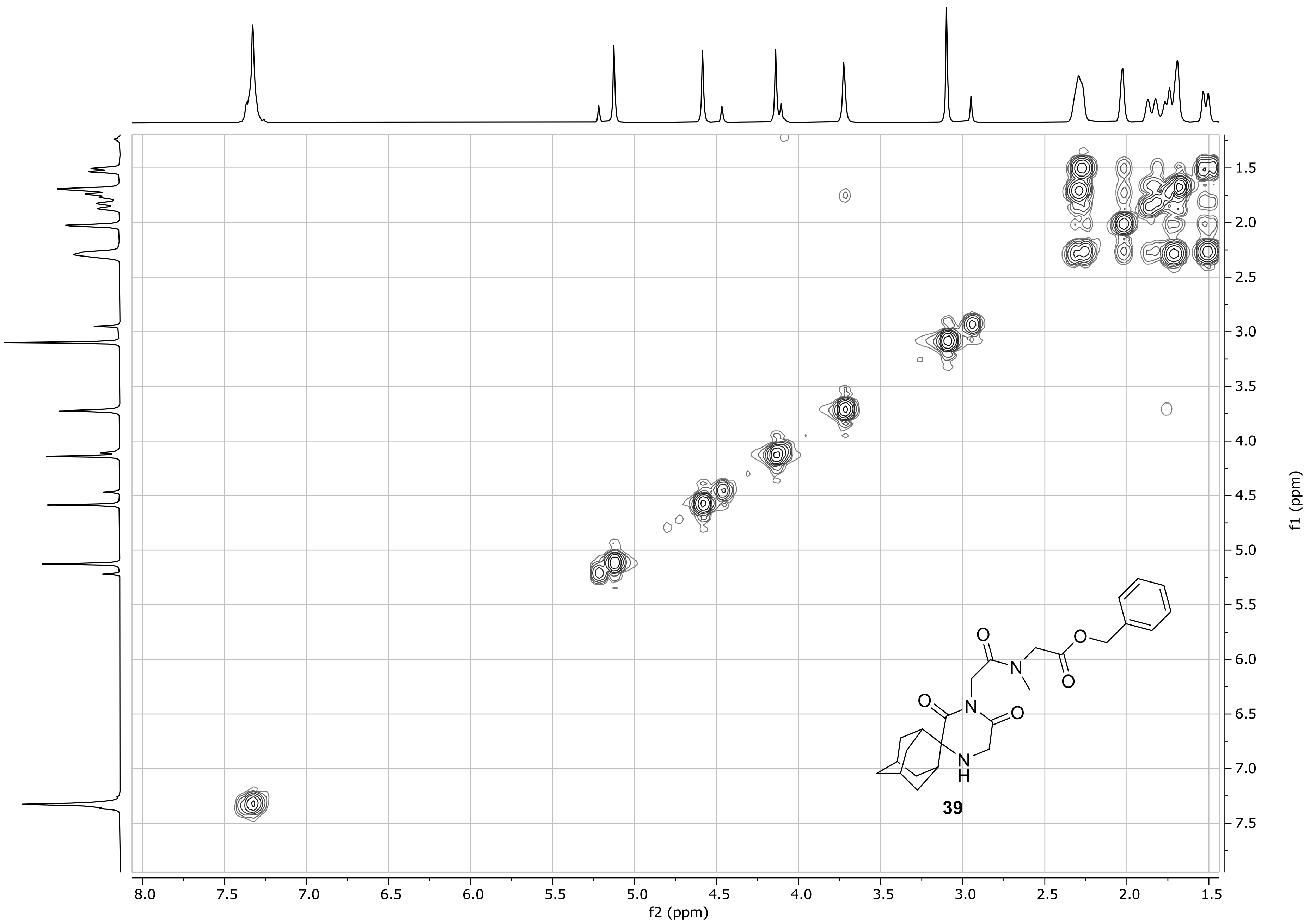

HSQC NMR of **39** (400.13 MHz, DMSO-d_6)

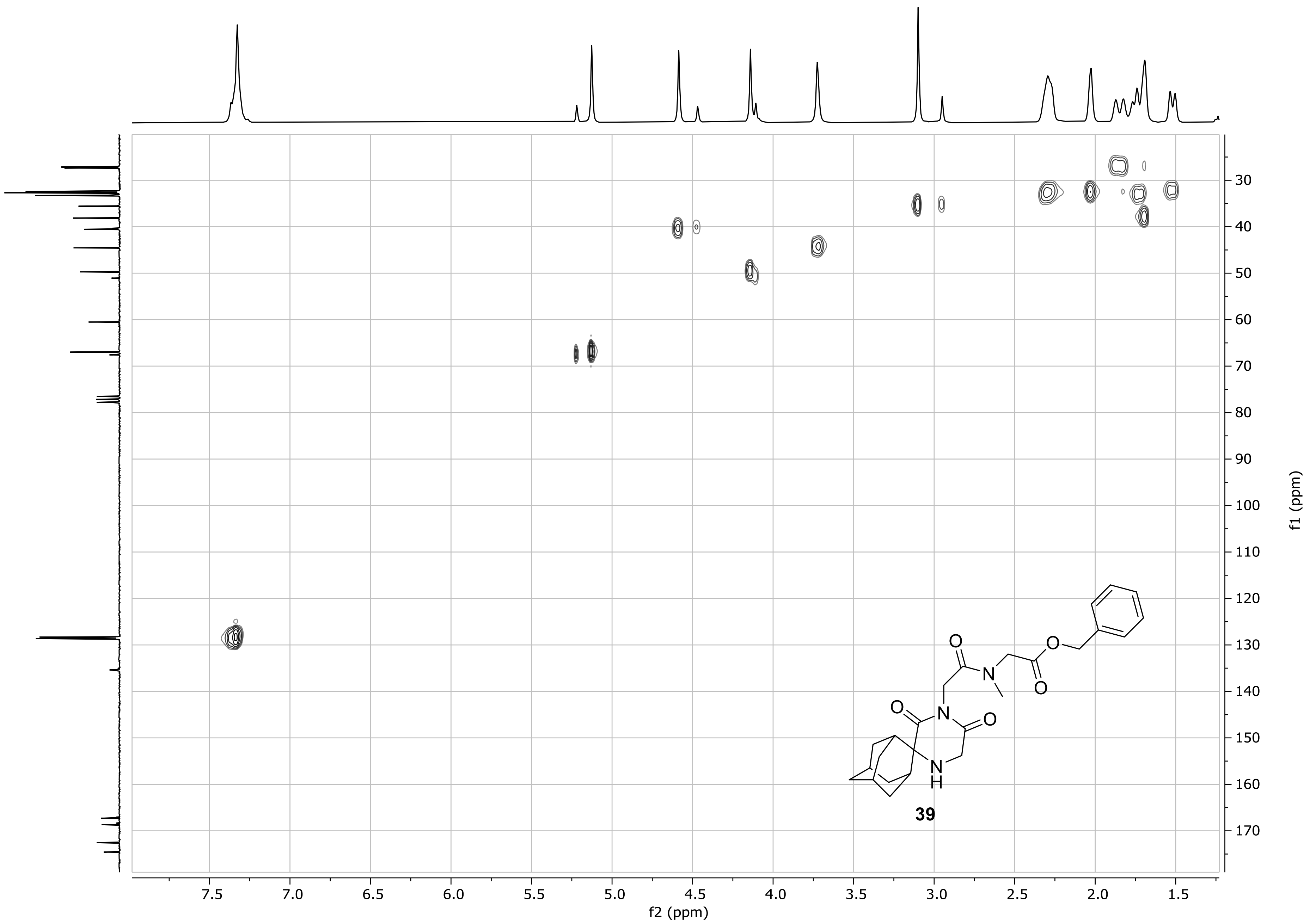

DEPT NMR of **39** (50.32 MHz, DMSO-d_6)

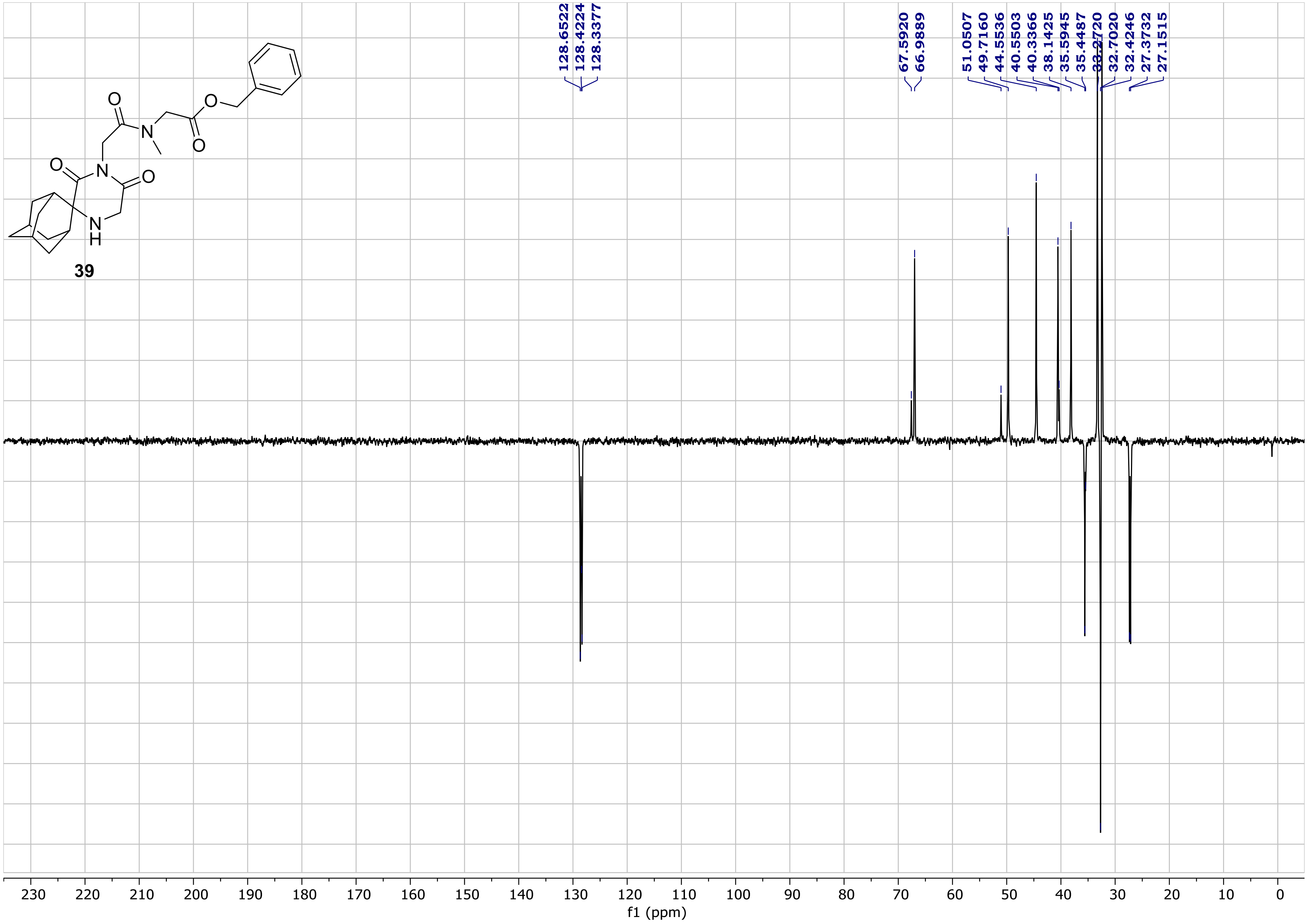

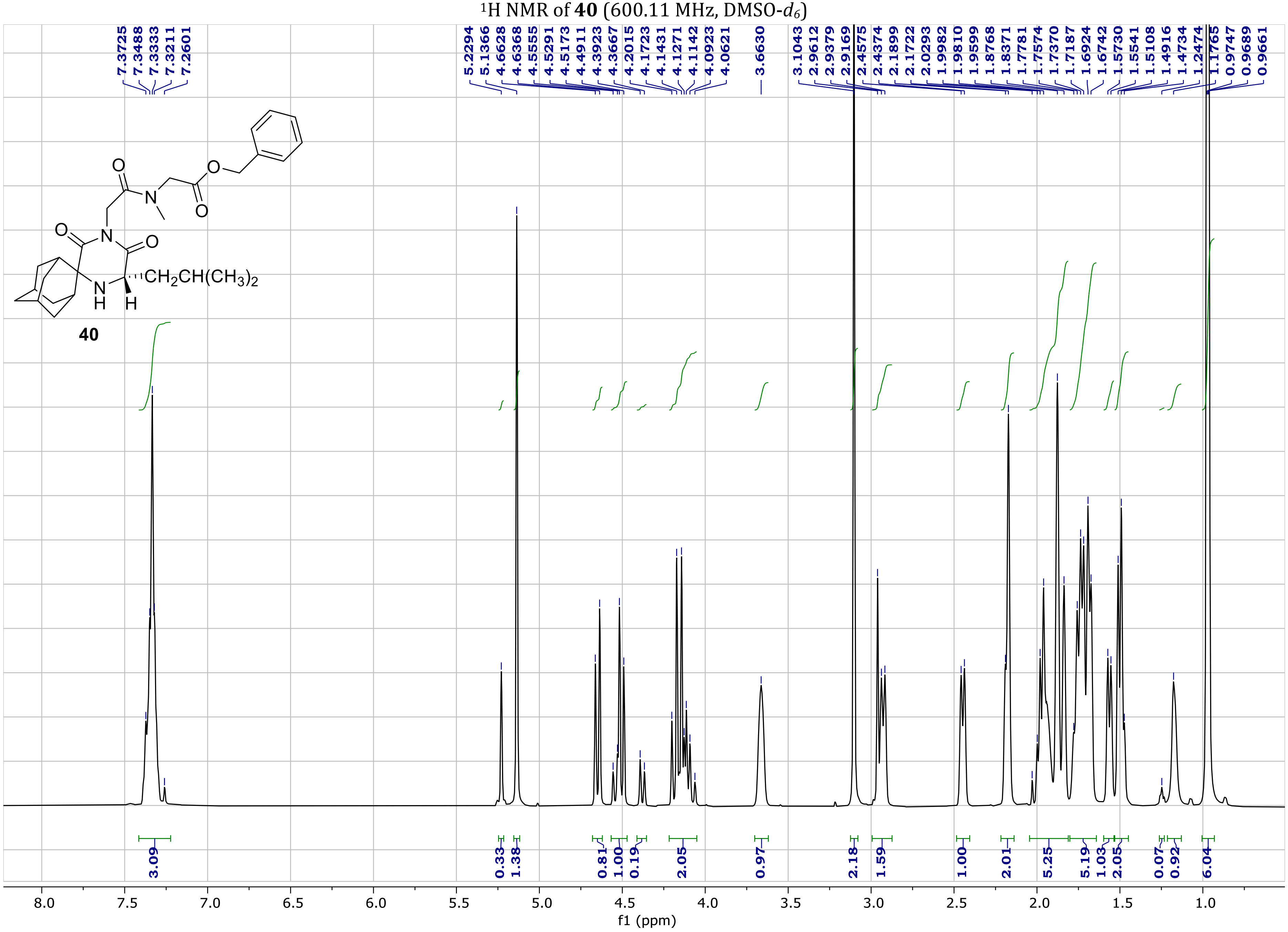

^{1}H NMR of **40** (600.11 MHz, DMSO-d_6)
40
f1 (ppm)

^{13}C NMR of **40** (50.32 MHz, DMSO-d_6)

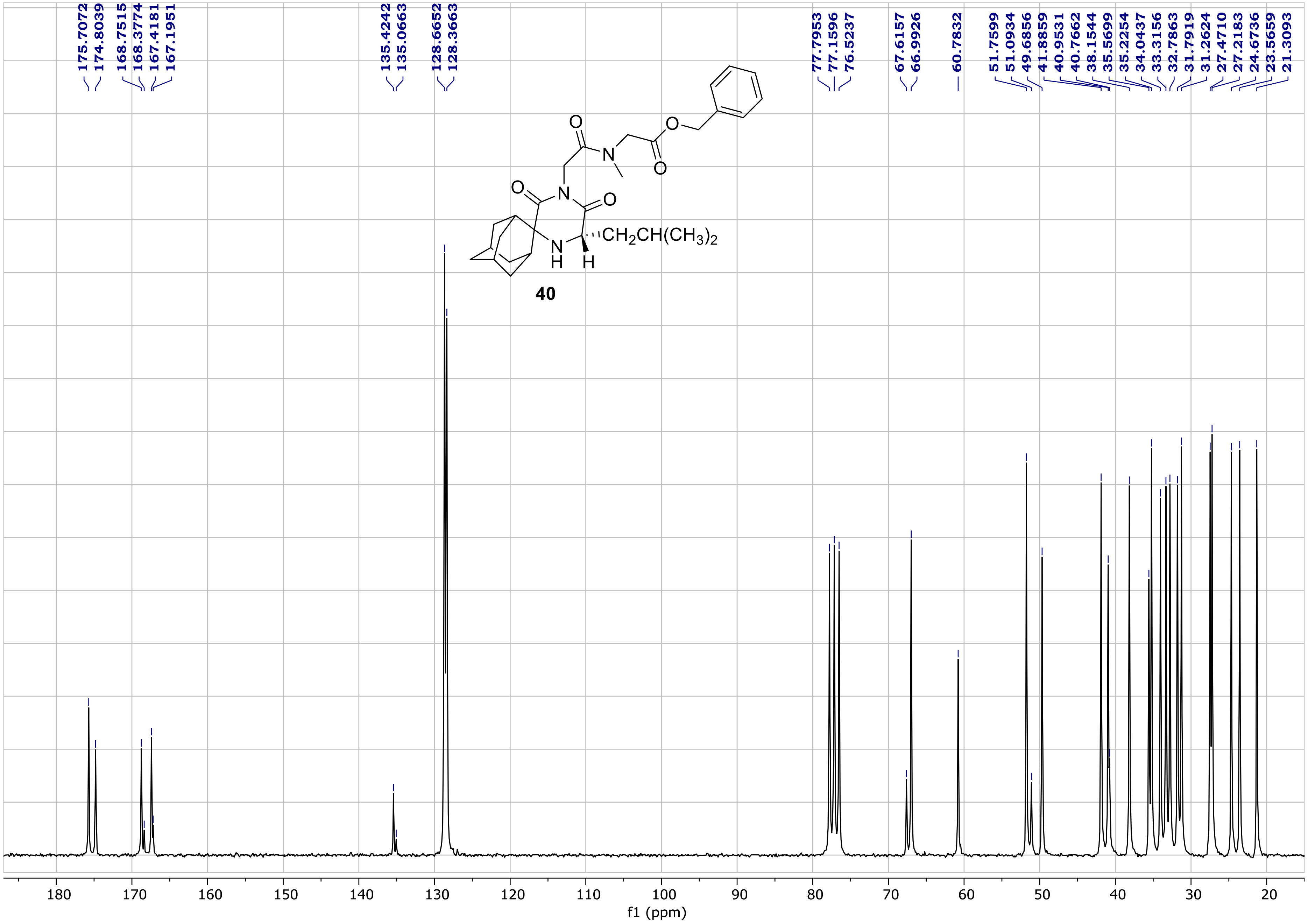

COSY NMR of **40** (600.11 MHz, DMSO-d_6)

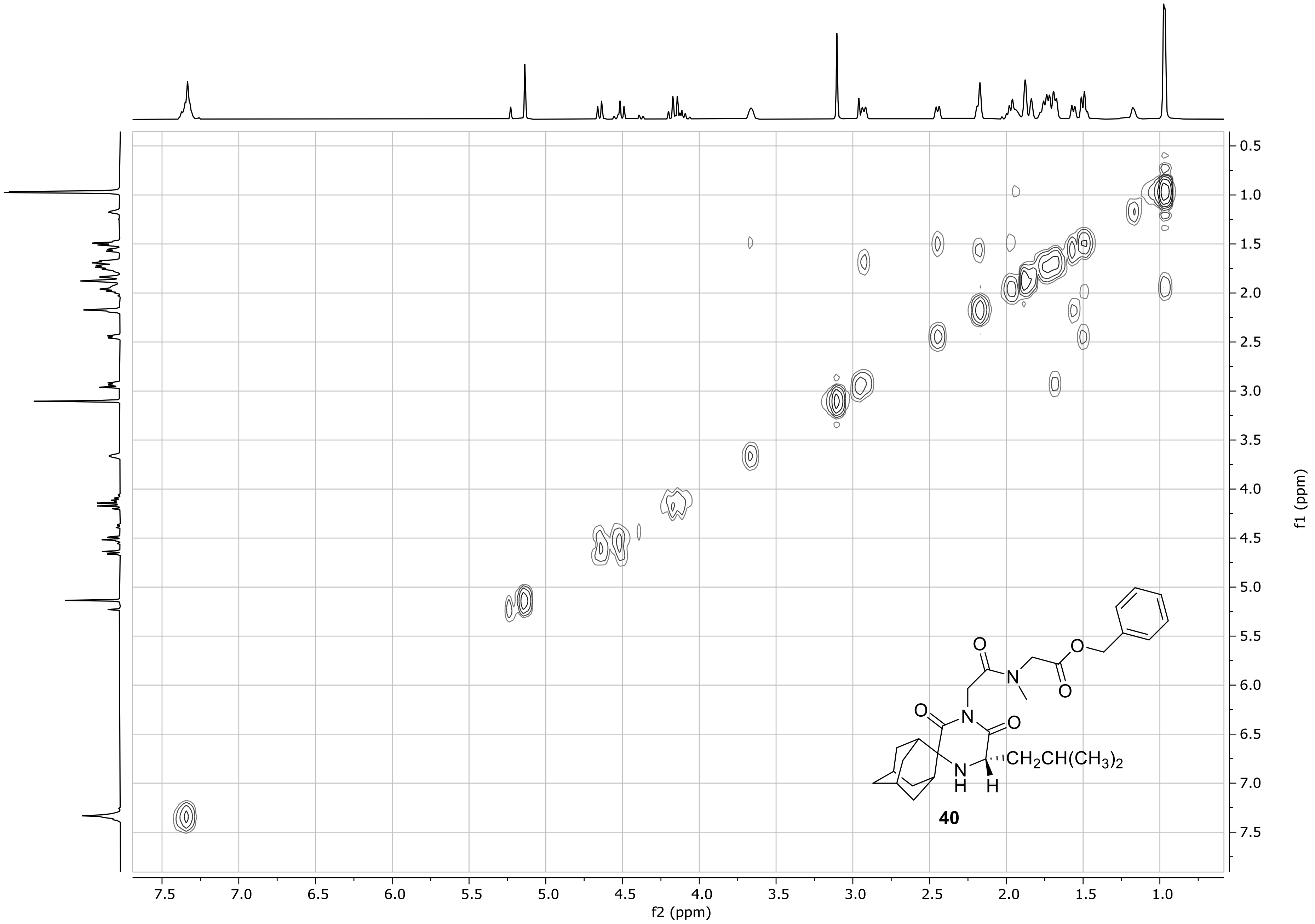

HSQC-DEPT NMR of **40** (600.11 MHz, DMSO-d_6)

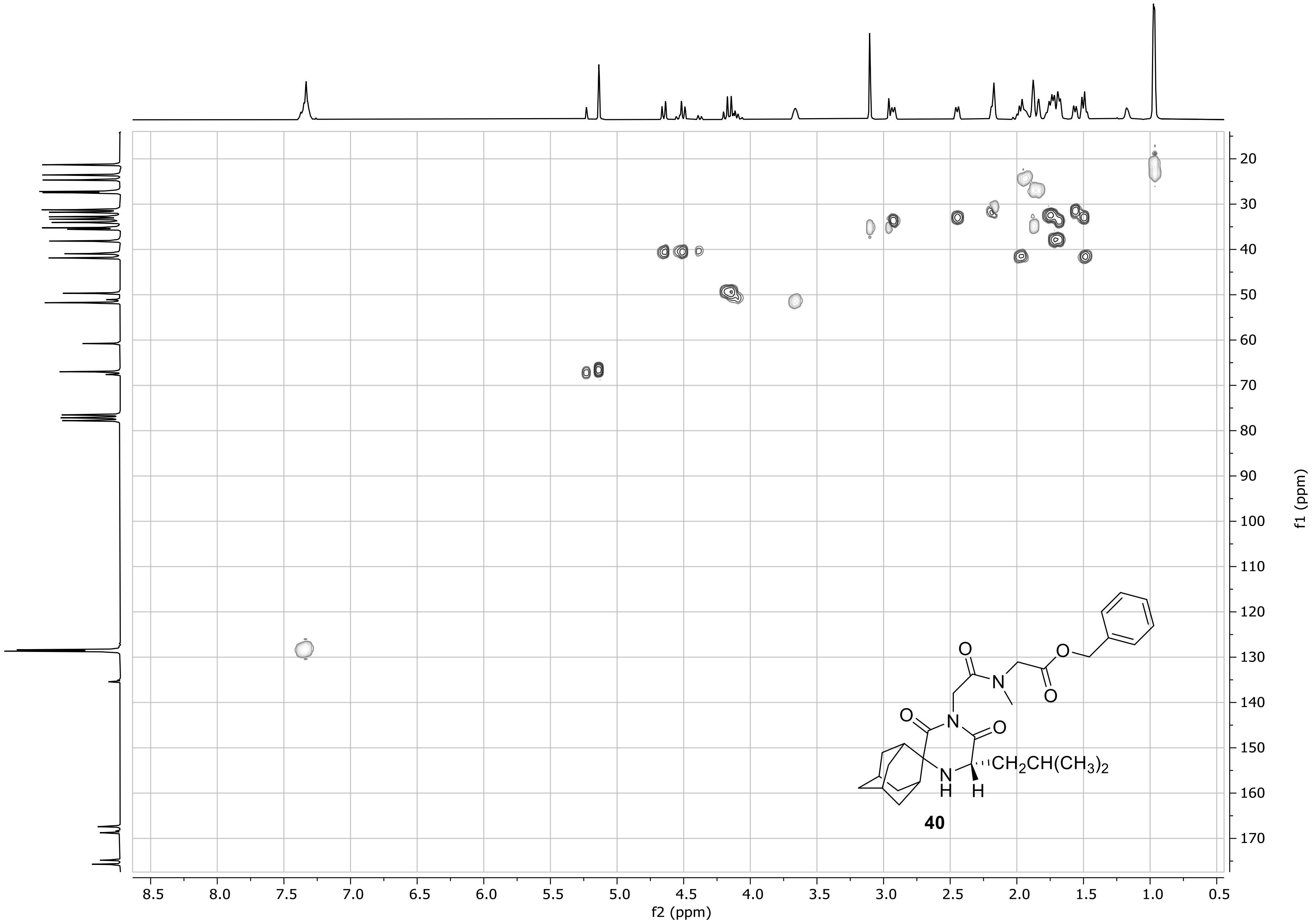

40

DEPT NMR of **40** (50.32 MHz, DMSO-d_6)

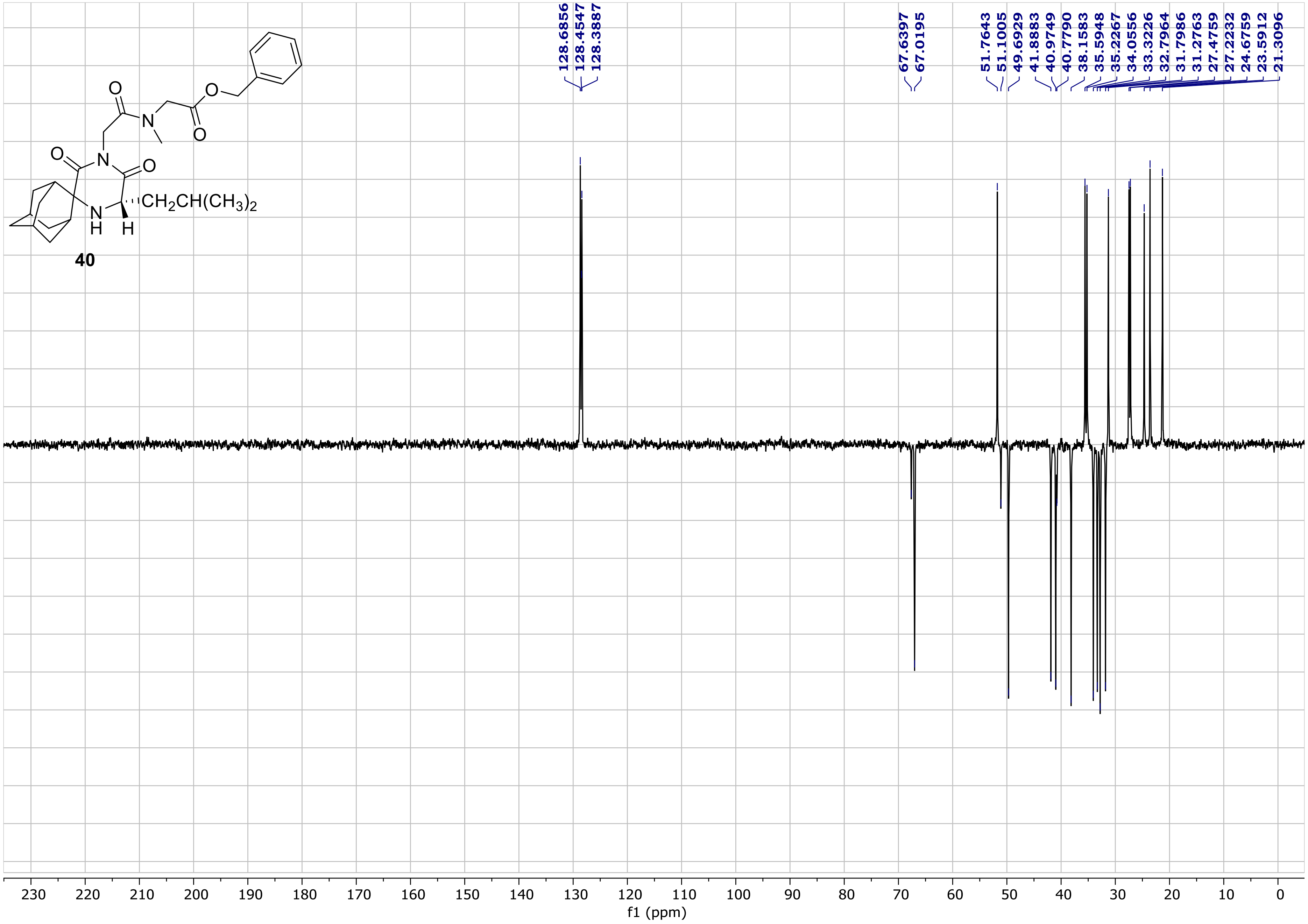

^{1}H NMR of **41** (600.11 MHz, DMSO-d_6)

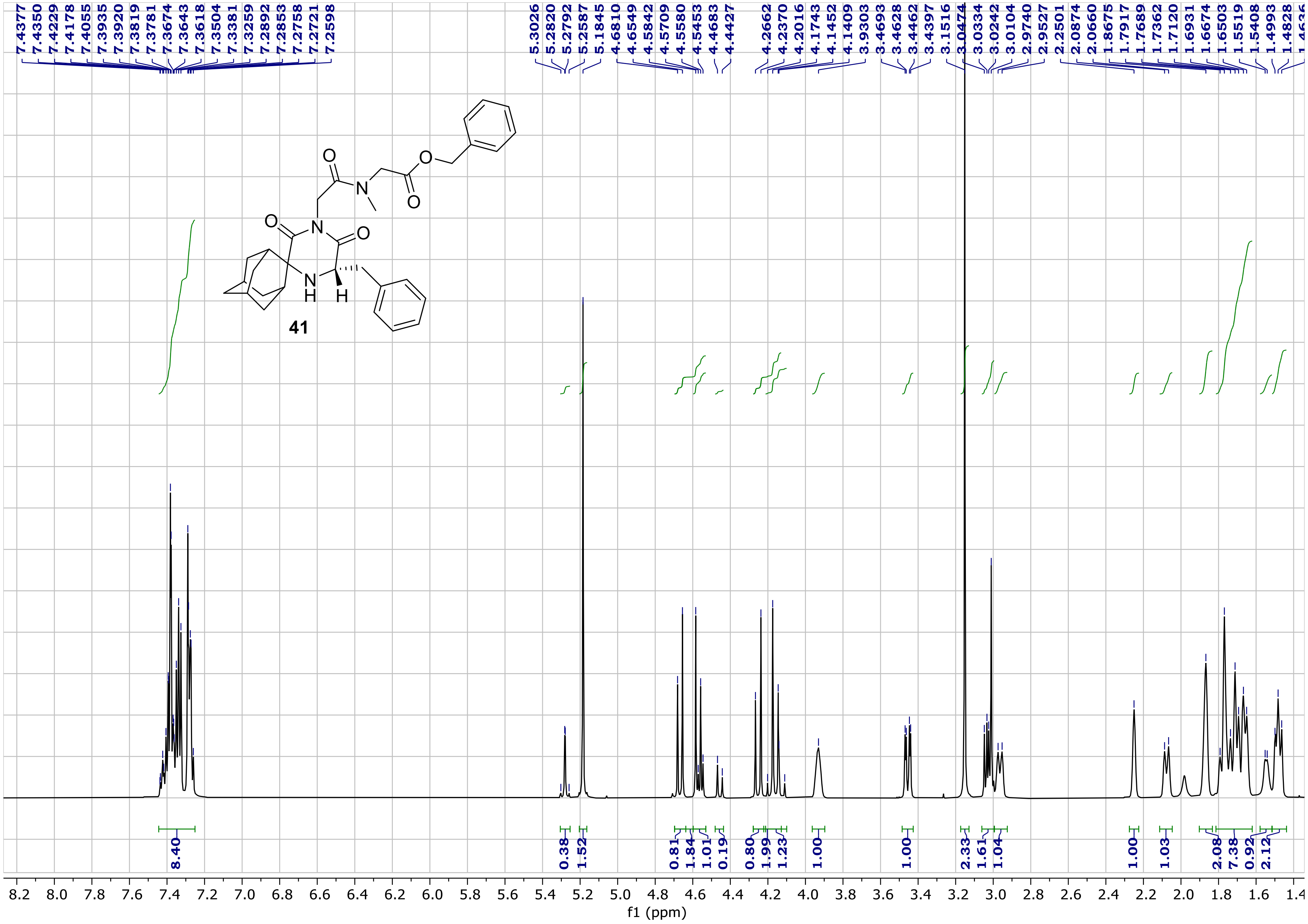

^{13}C NMR of **41** (150.9 MHz, DMSO-d_6)

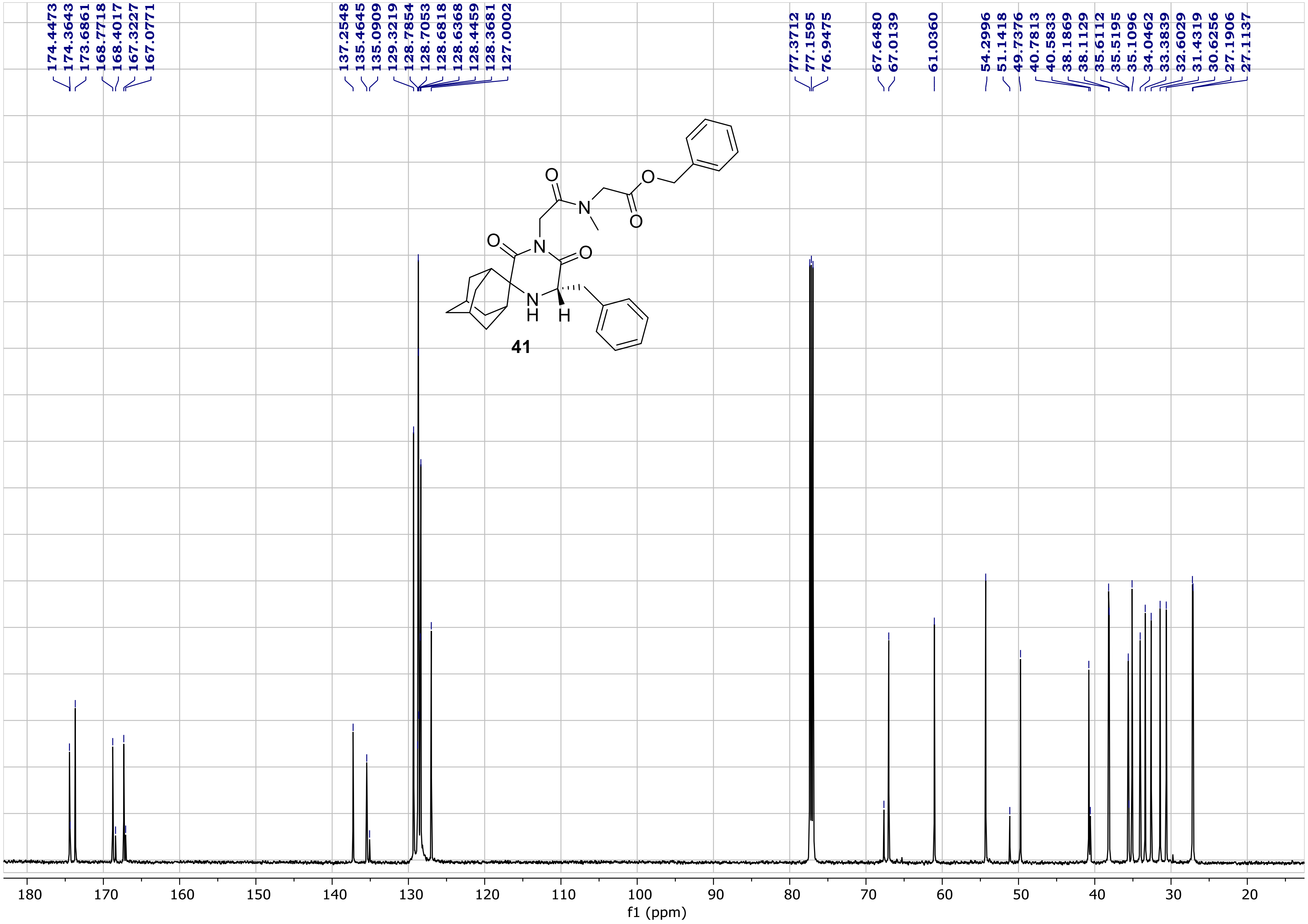

COSY NMR of **41** (600.11 MHz, DMSO-d_6)

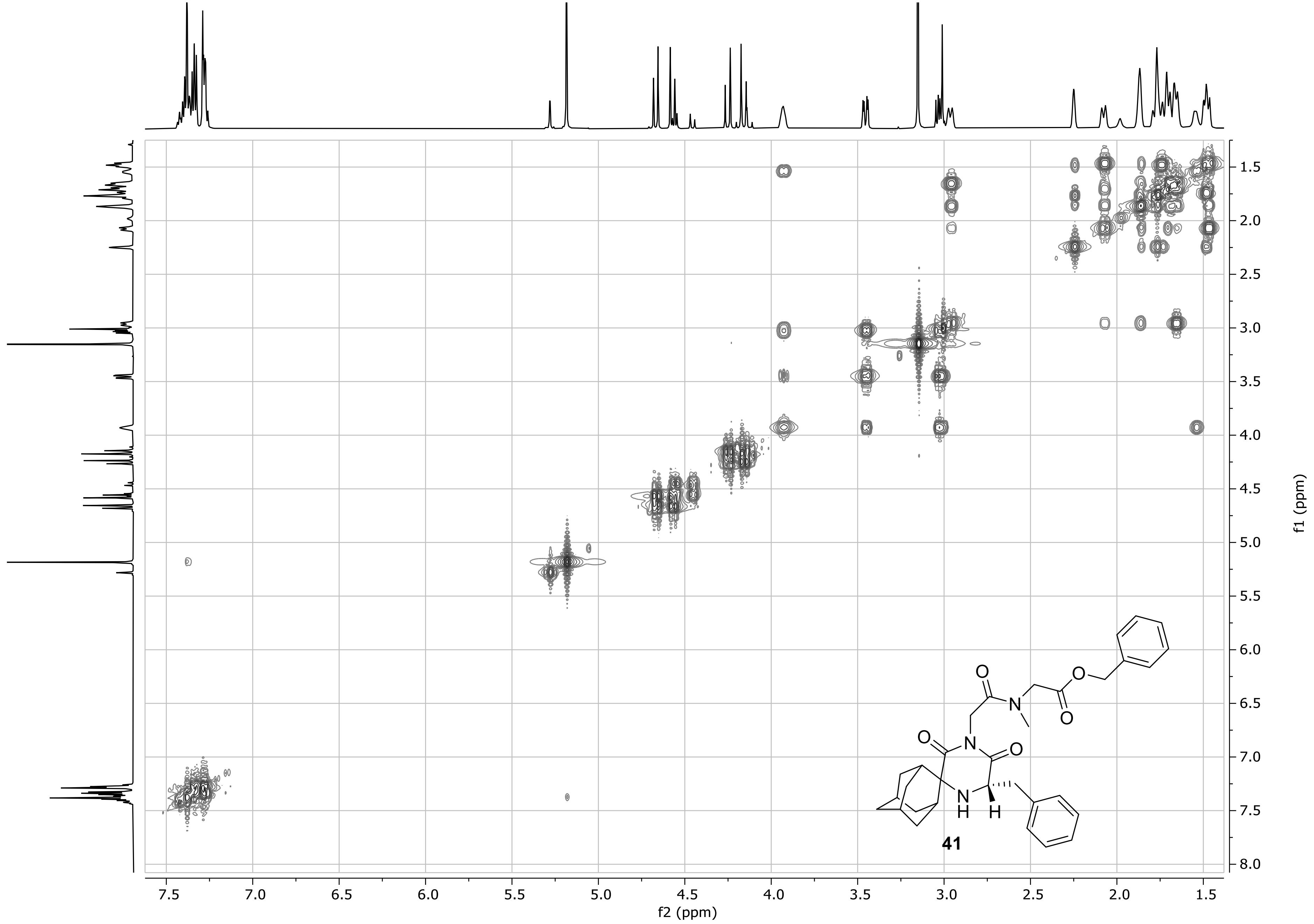

HSQC-DEPT NMR of **41** (600.11 MHz, DMSO-d_6)

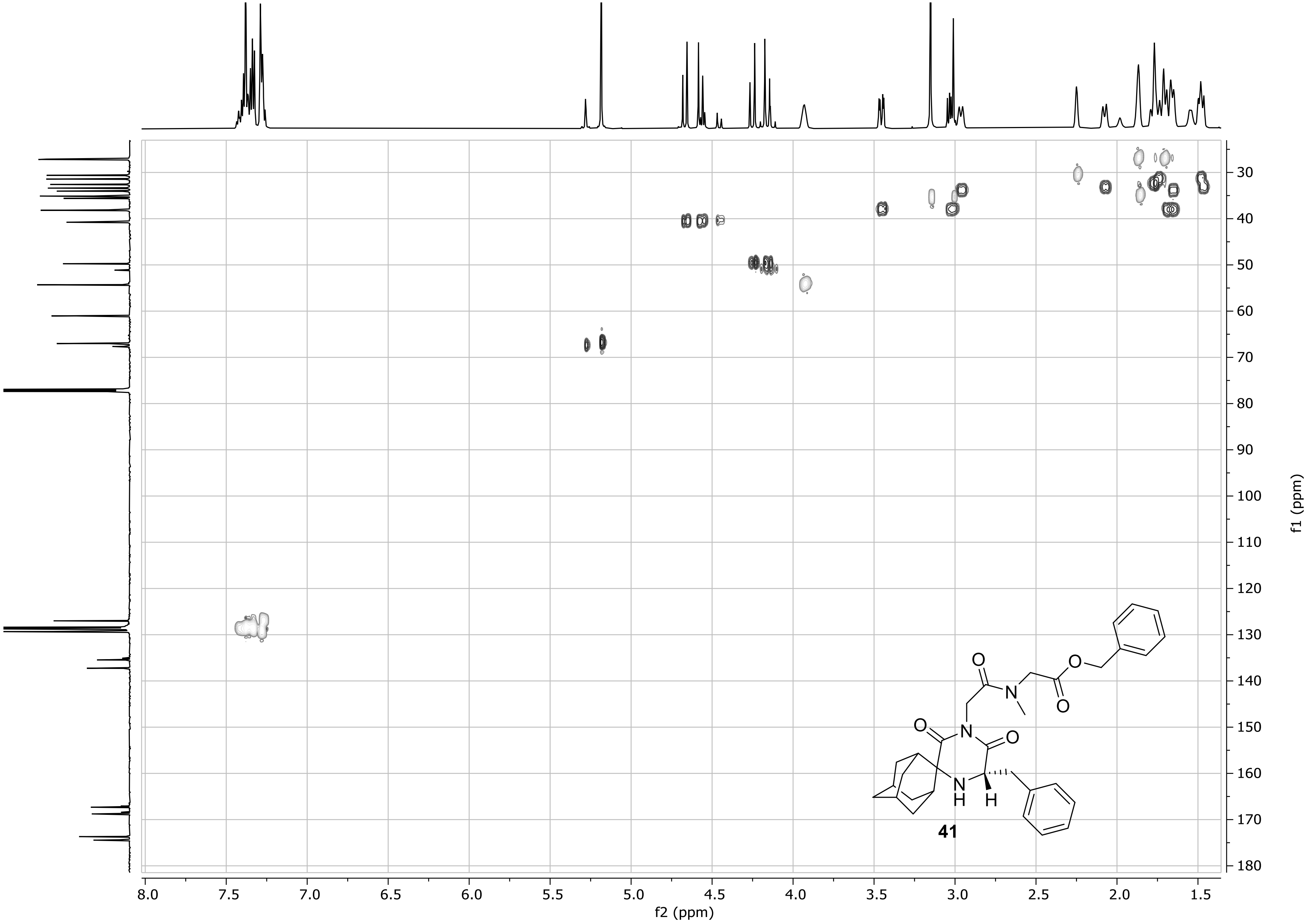

^{1}H NMR of **42** (400.13 MHz, DMSO-d_6)

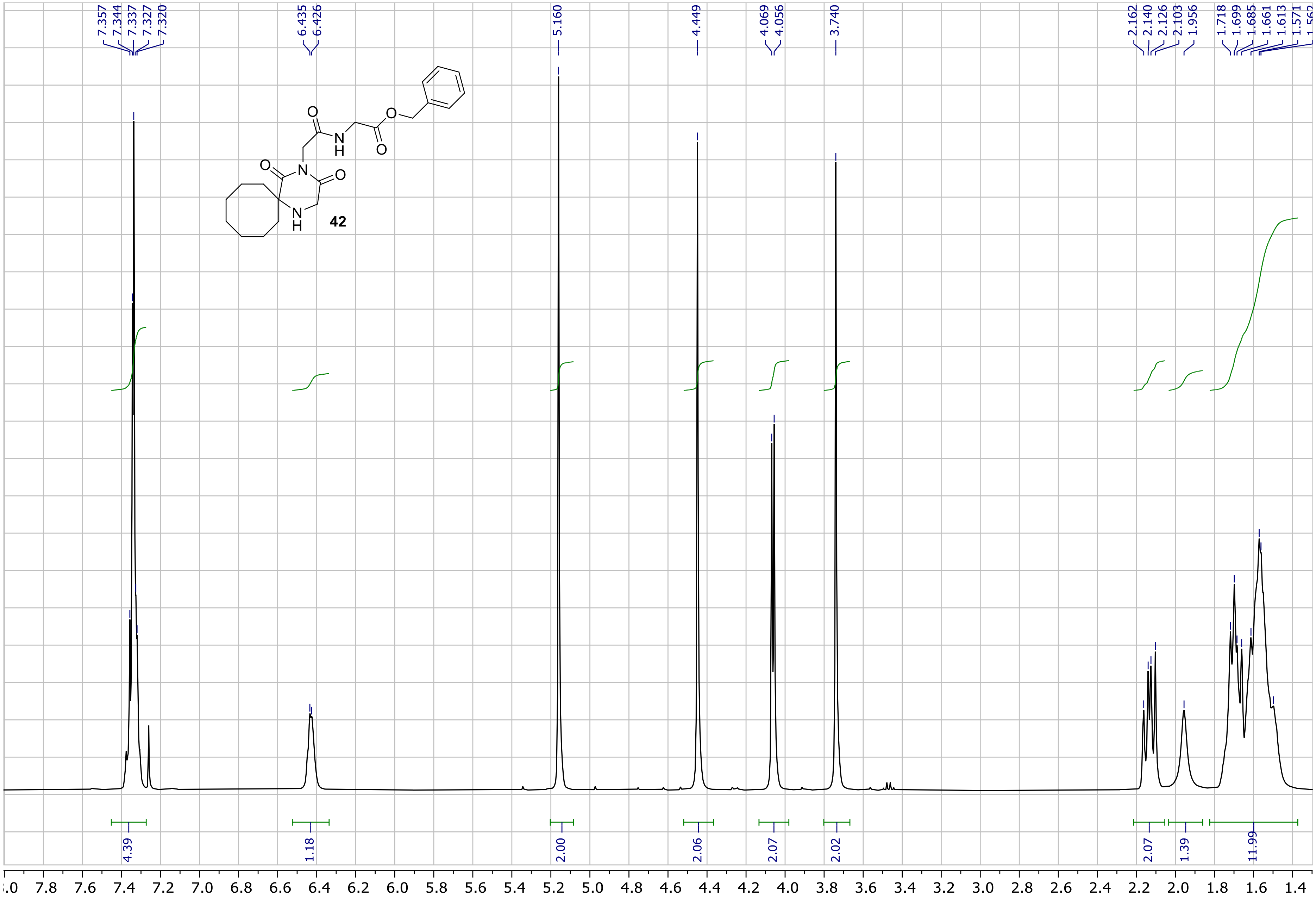

^{13}C NMR of **42** (50.32 MHz, DMSO-d_6)

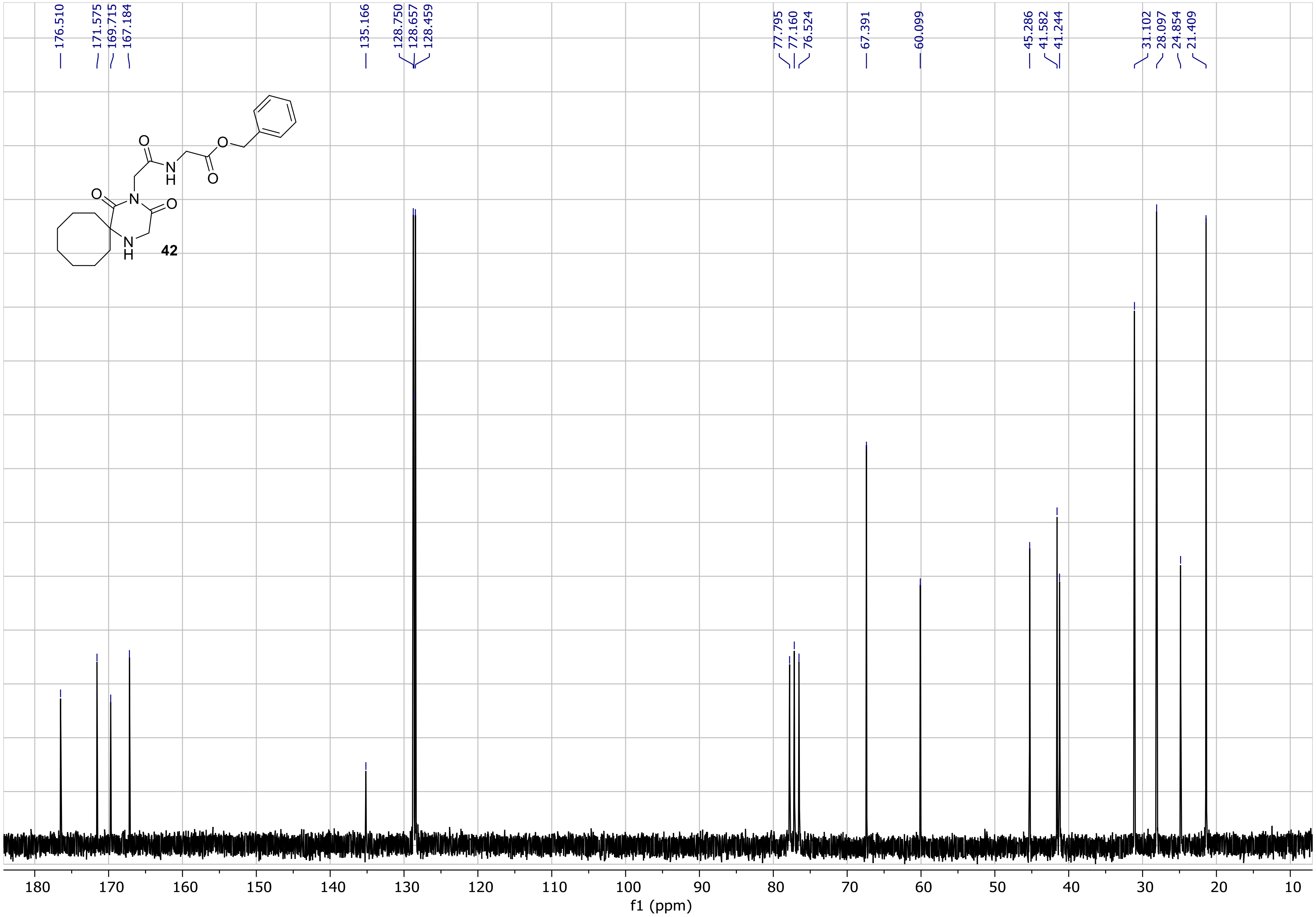

COSY NMR of **42** (400.13 MHz, DMSO-d_6)

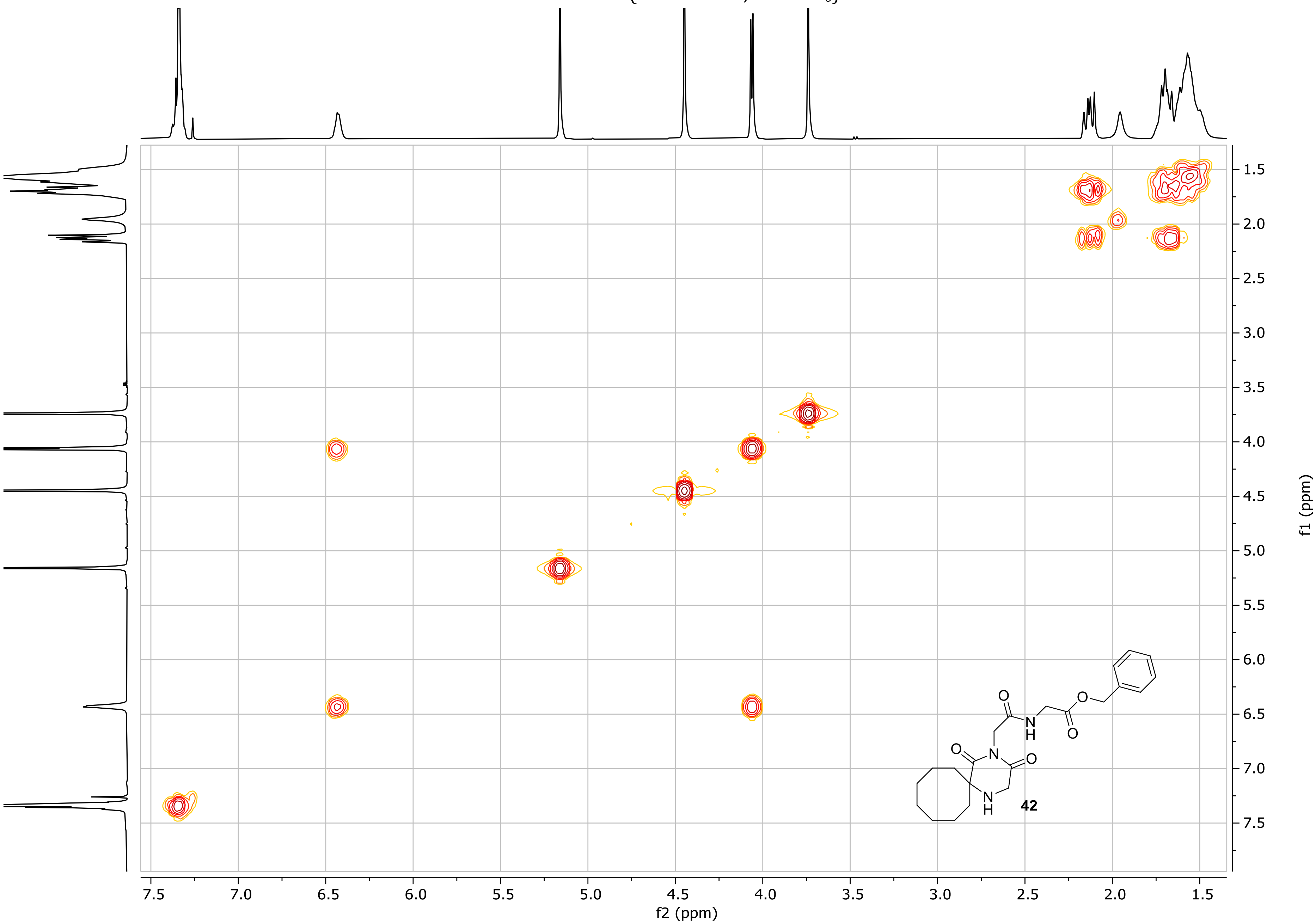

HSQC NMR of **42** (400.13 MHz, DMSO-d_6)

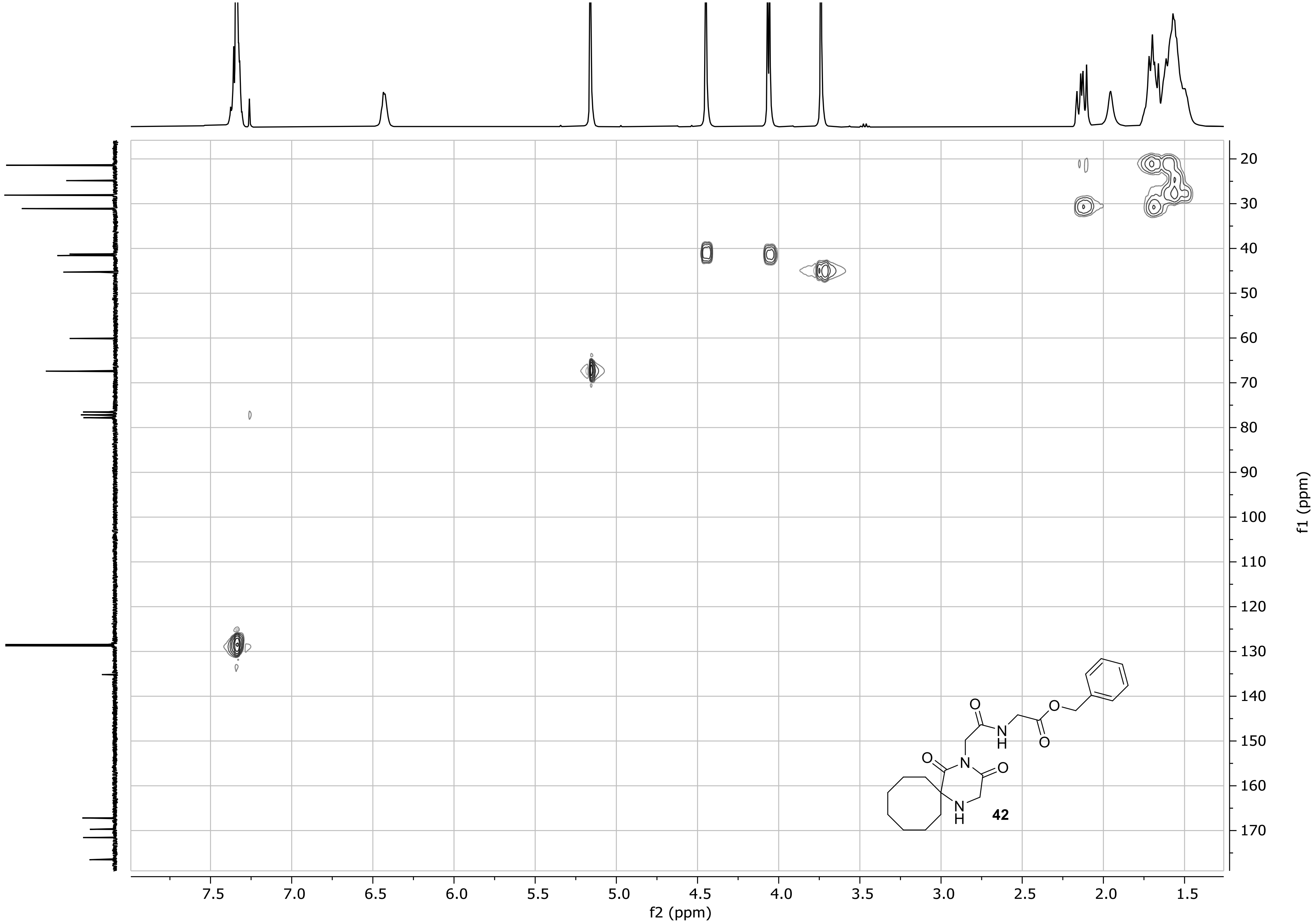

DEPT NMR of **42** (50.32 MHz, DMSO-d_6)

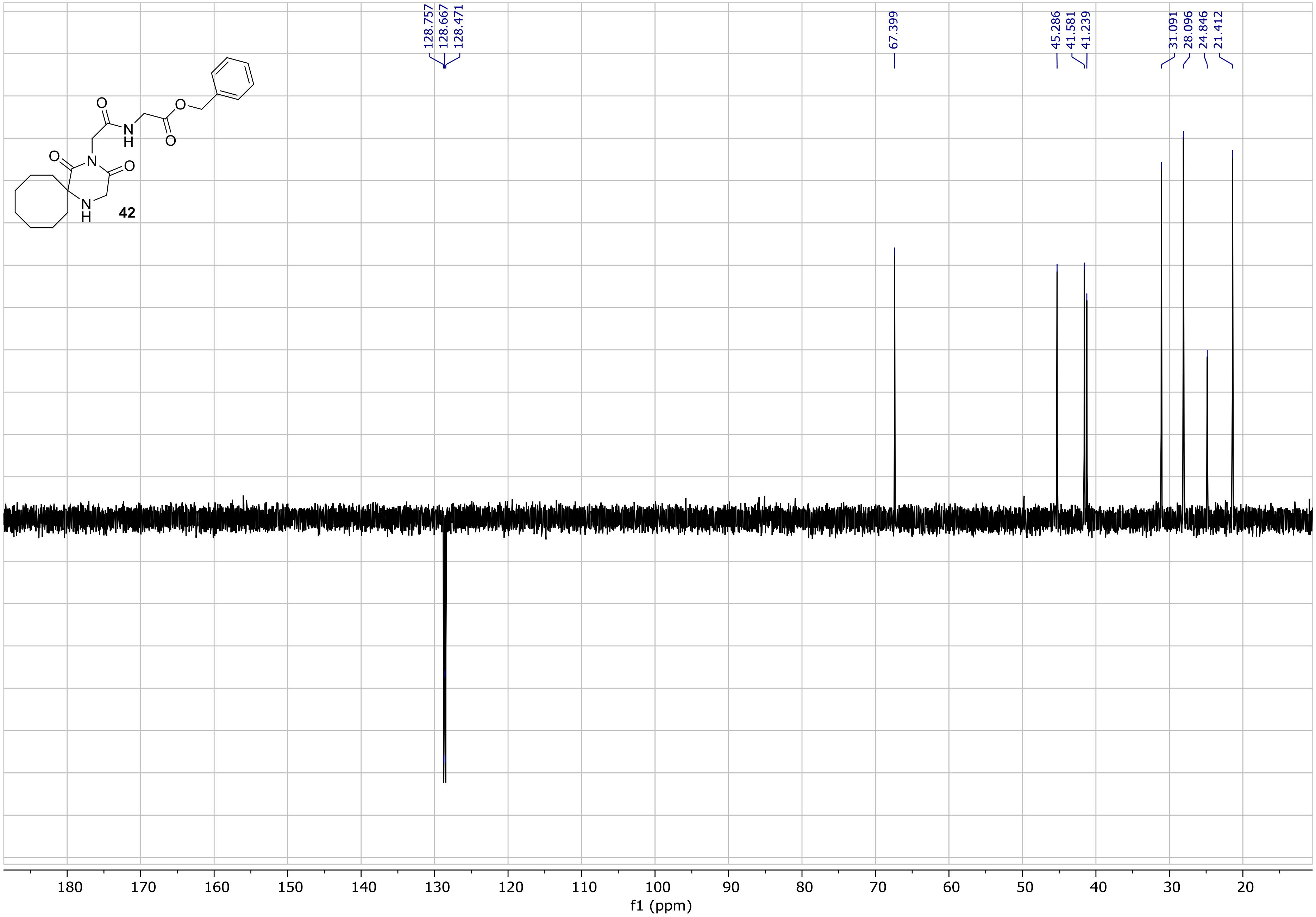

^{1}H NMR of **43** (400.13 MHz, DMSO-d_6)

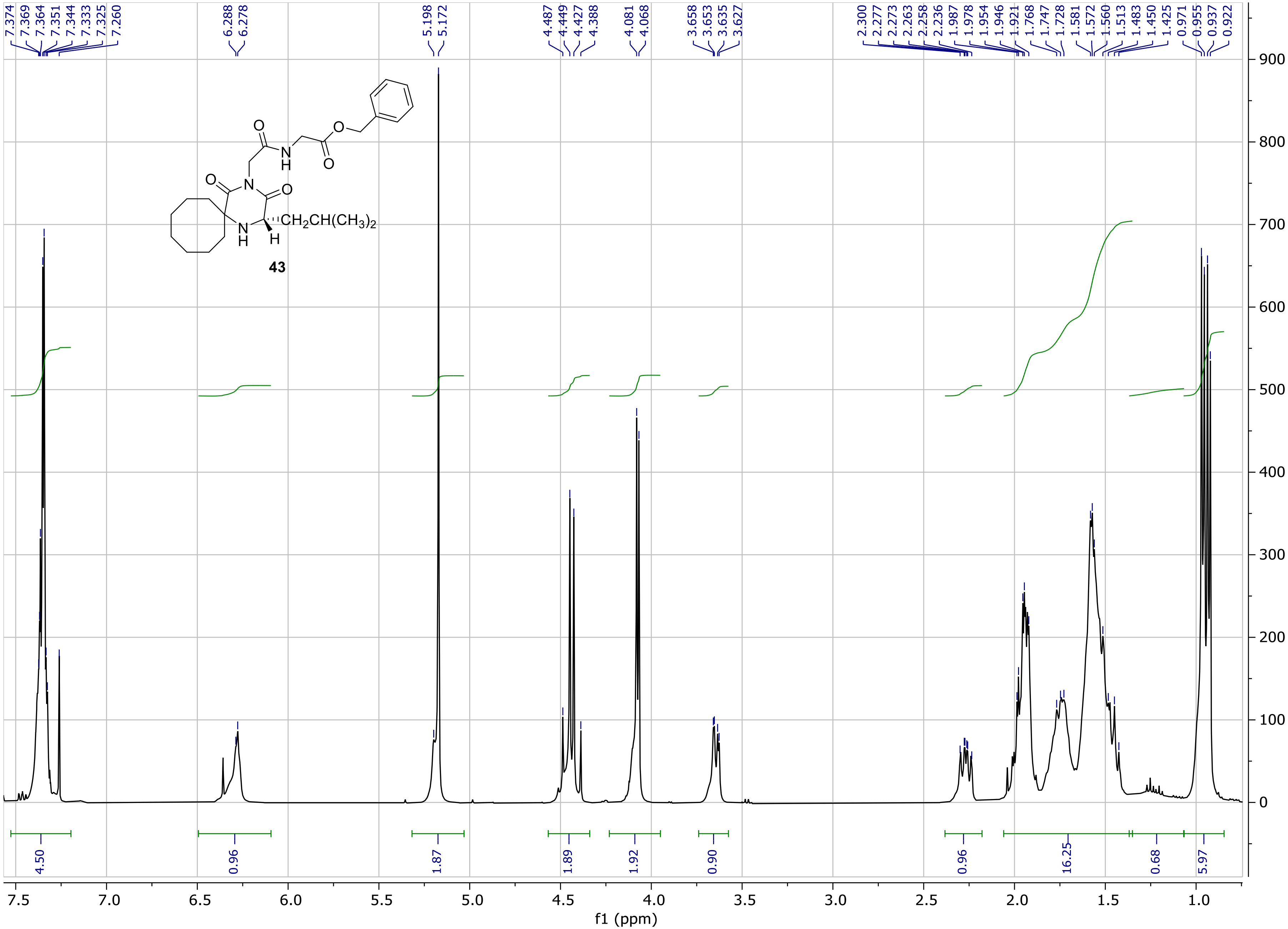

^{13}C NMR of **43** (50.32 MHz, DMSO-d_6)

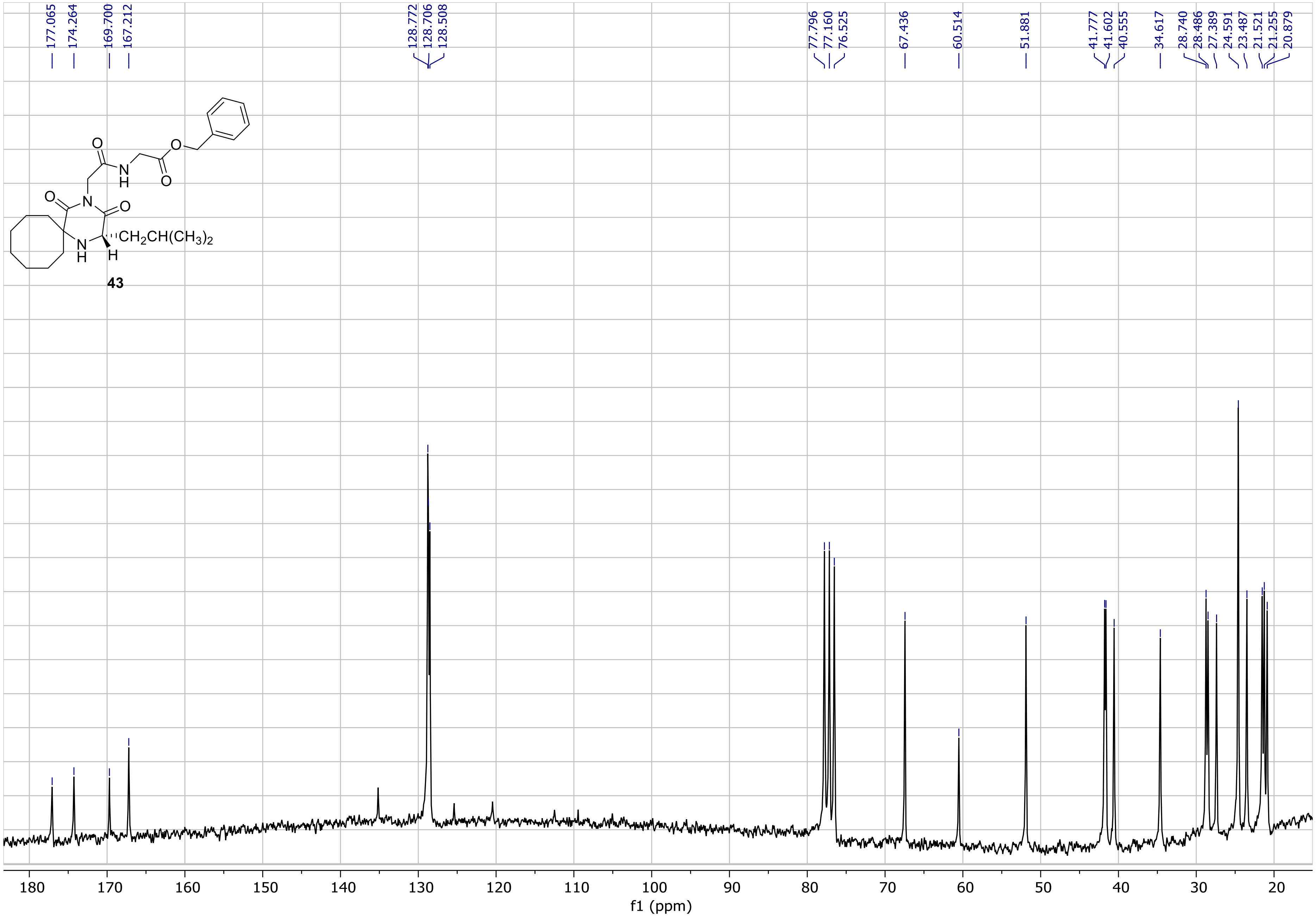

COSY NMR of **43** (400.13 MHz, DMSO-d_6)

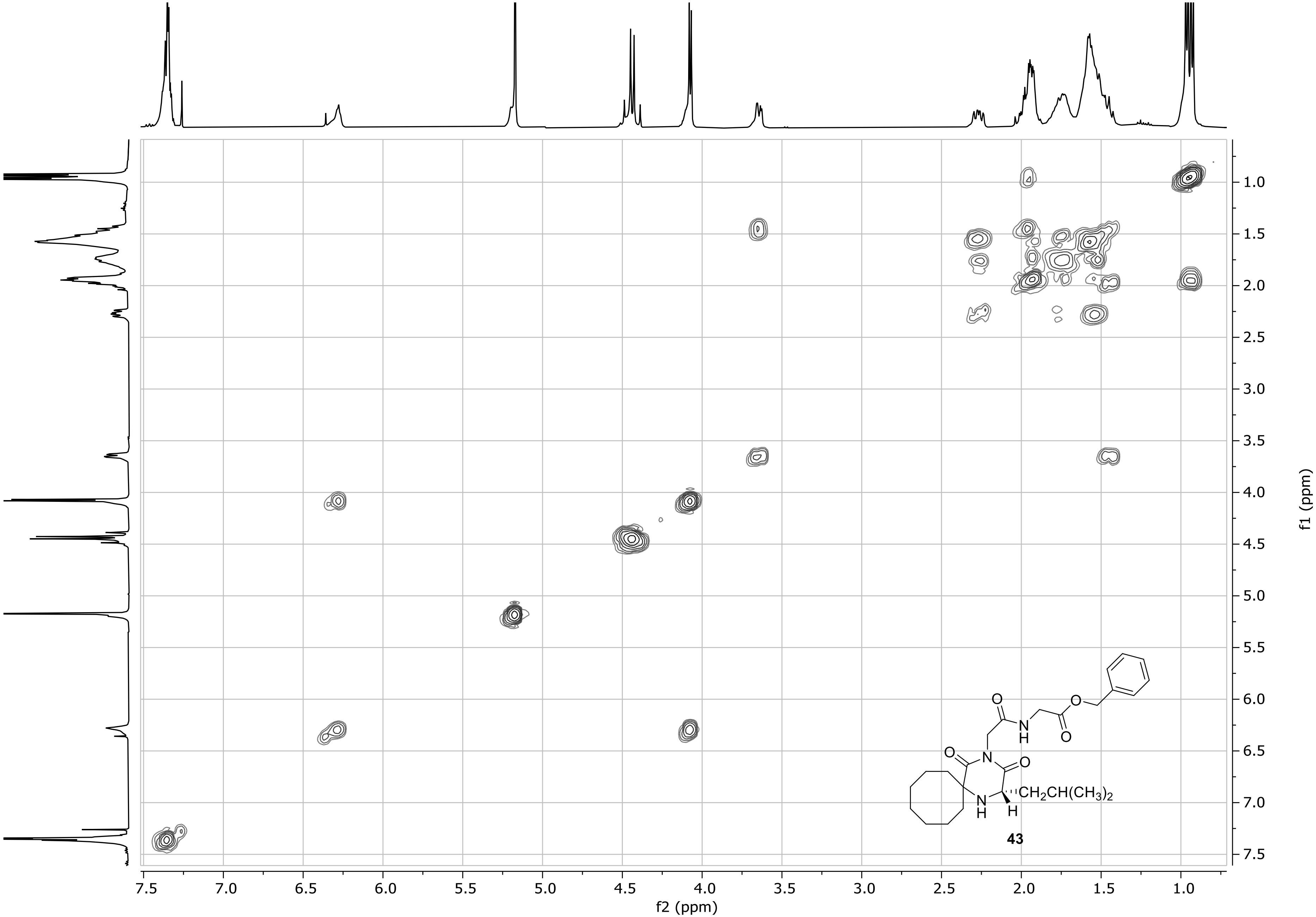

HSQC NMR of **43** (400.13 MHz, DMSO-d_6)

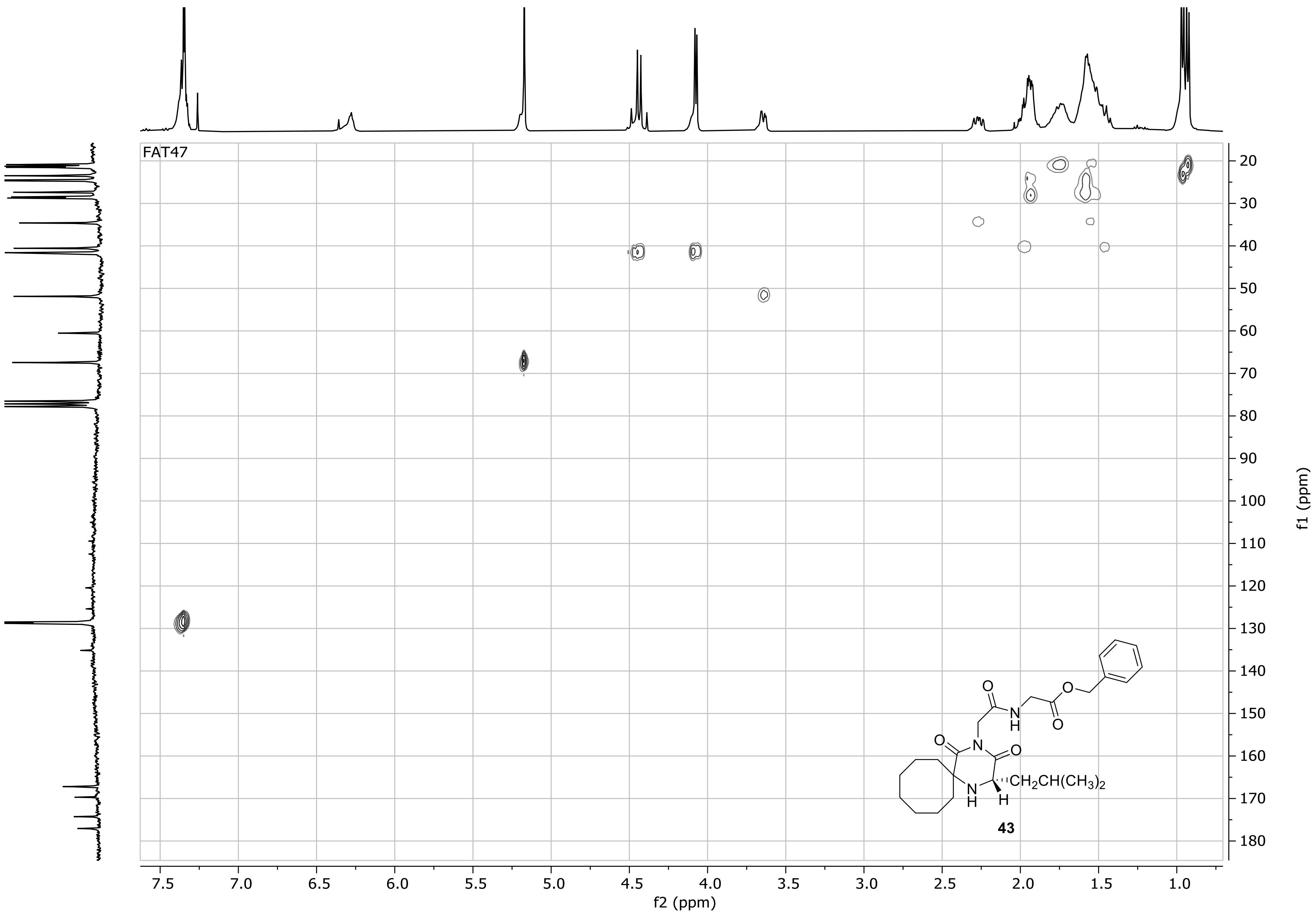

DEPT NMR of **43** (50.32 MHz, DMSO-d_6)

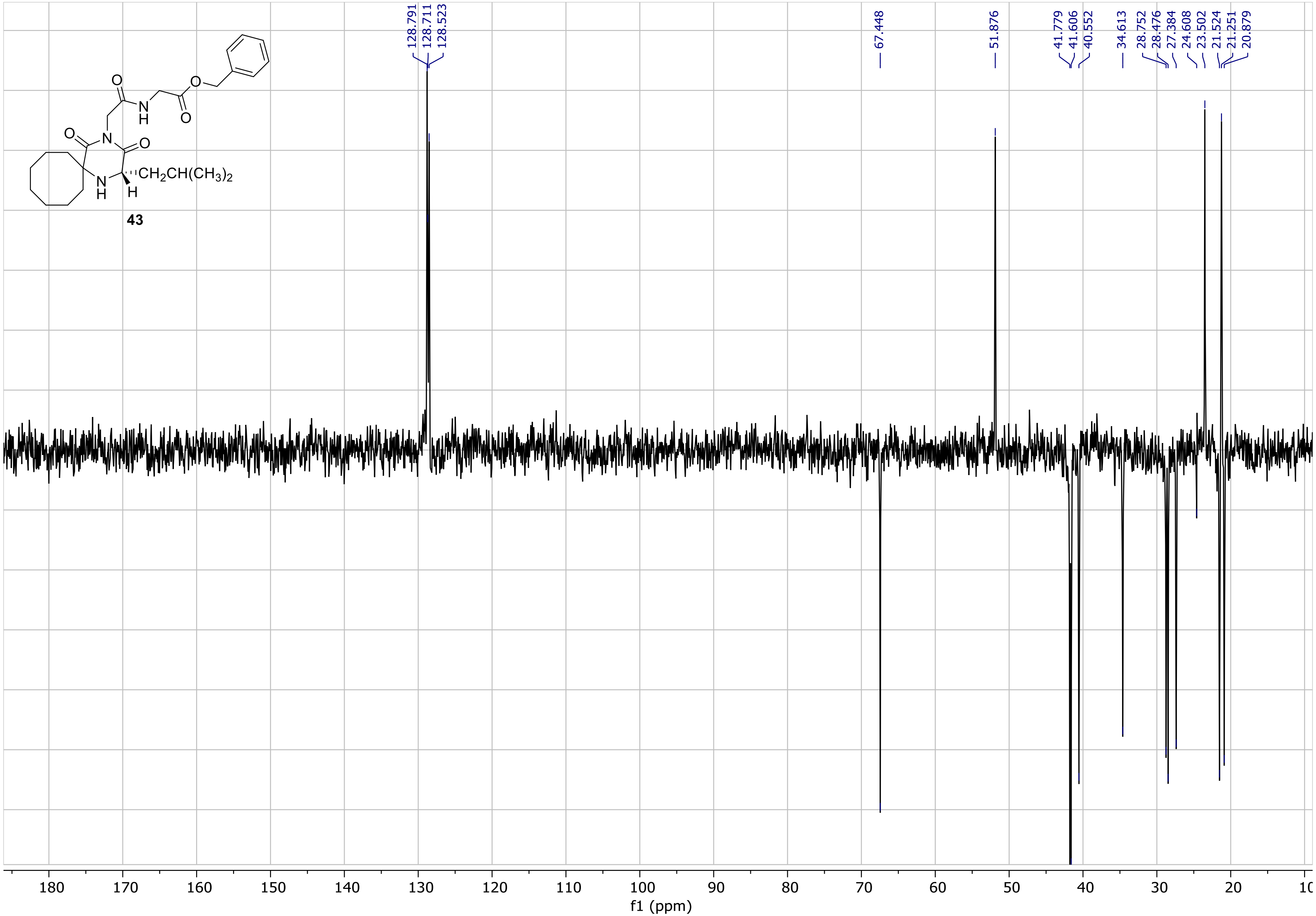

^{1}H NMR of **44** (400.13 MHz, DMSO-d_6)

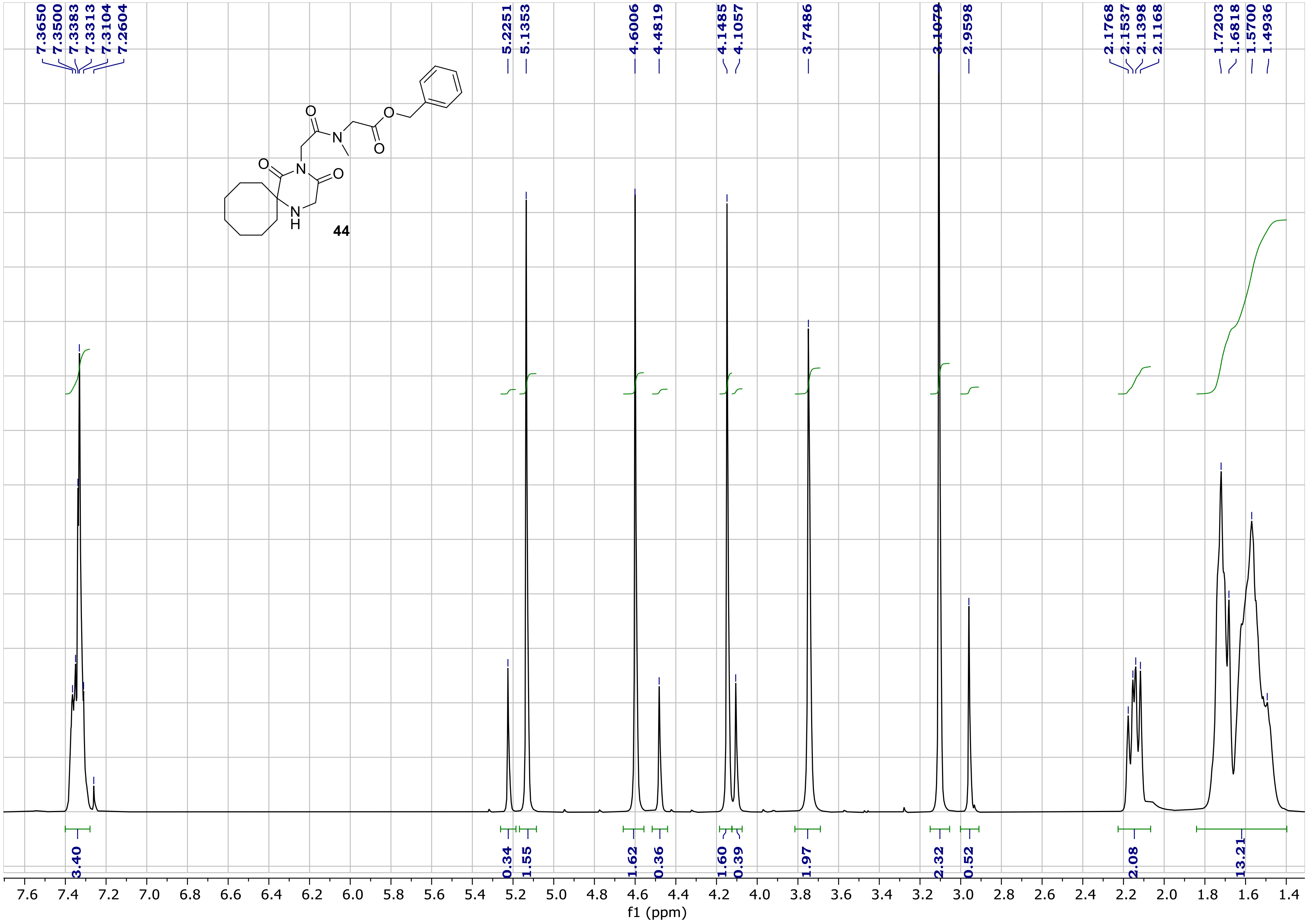

^{13}C NMR of **44** (50.32 MHz, DMSO-d_6)

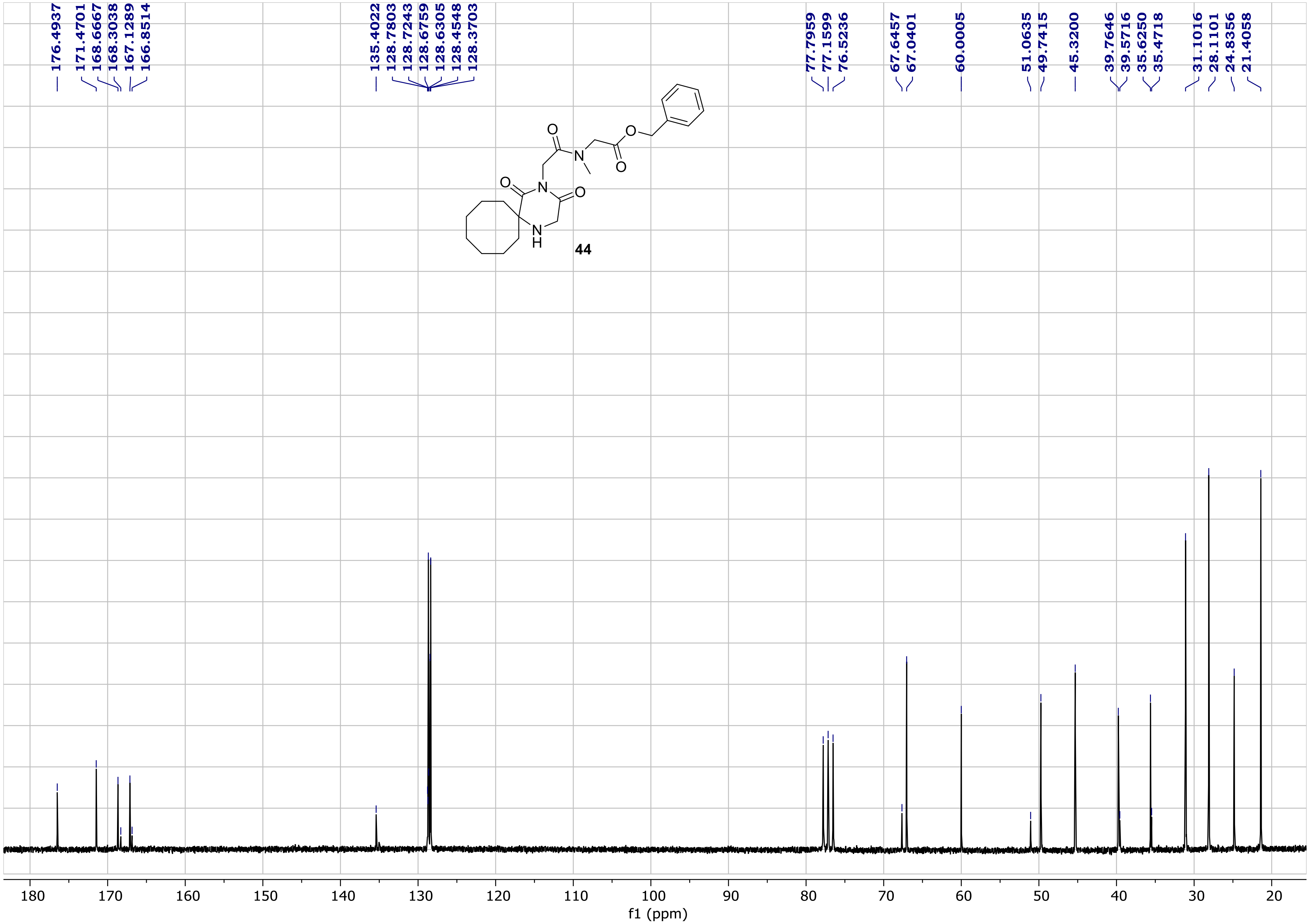

COSY NMR of **44** (400.13 MHz, DMSO-d_6)

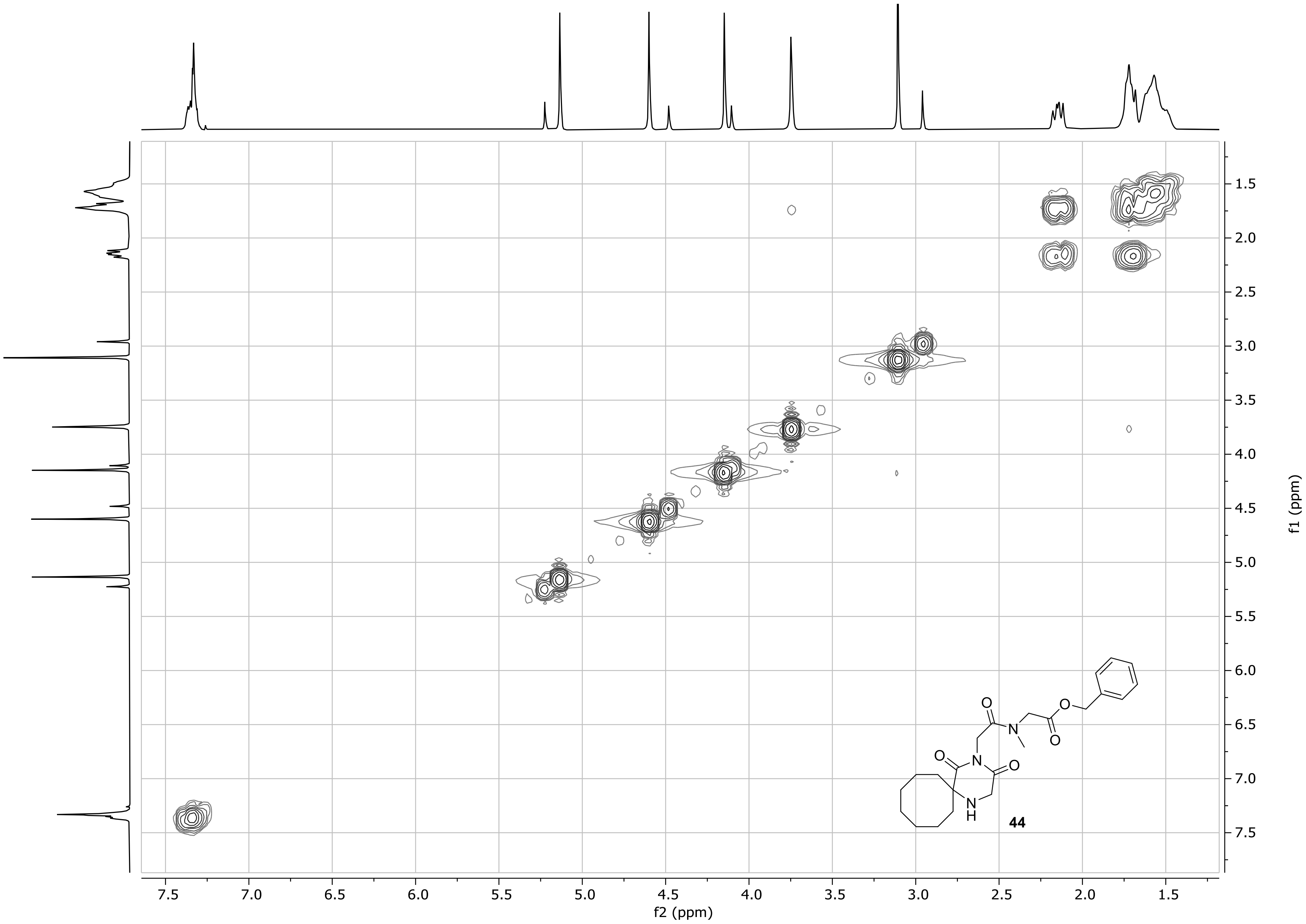

HSQC NMR of **44** (400.13 MHz, DMSO-d_6)

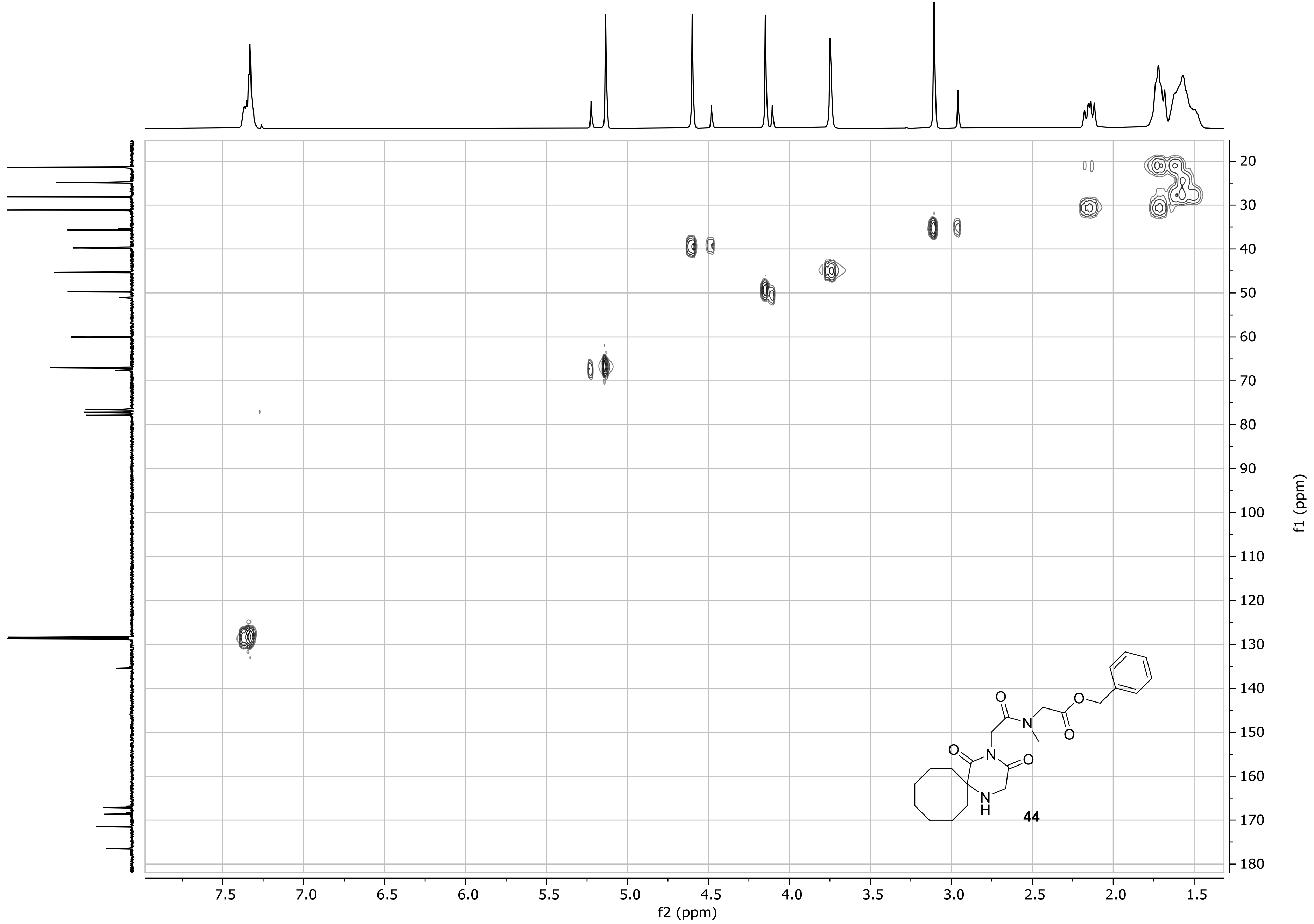

DEPT NMR of **44** (50.32 MHz, DMSO-d_6)

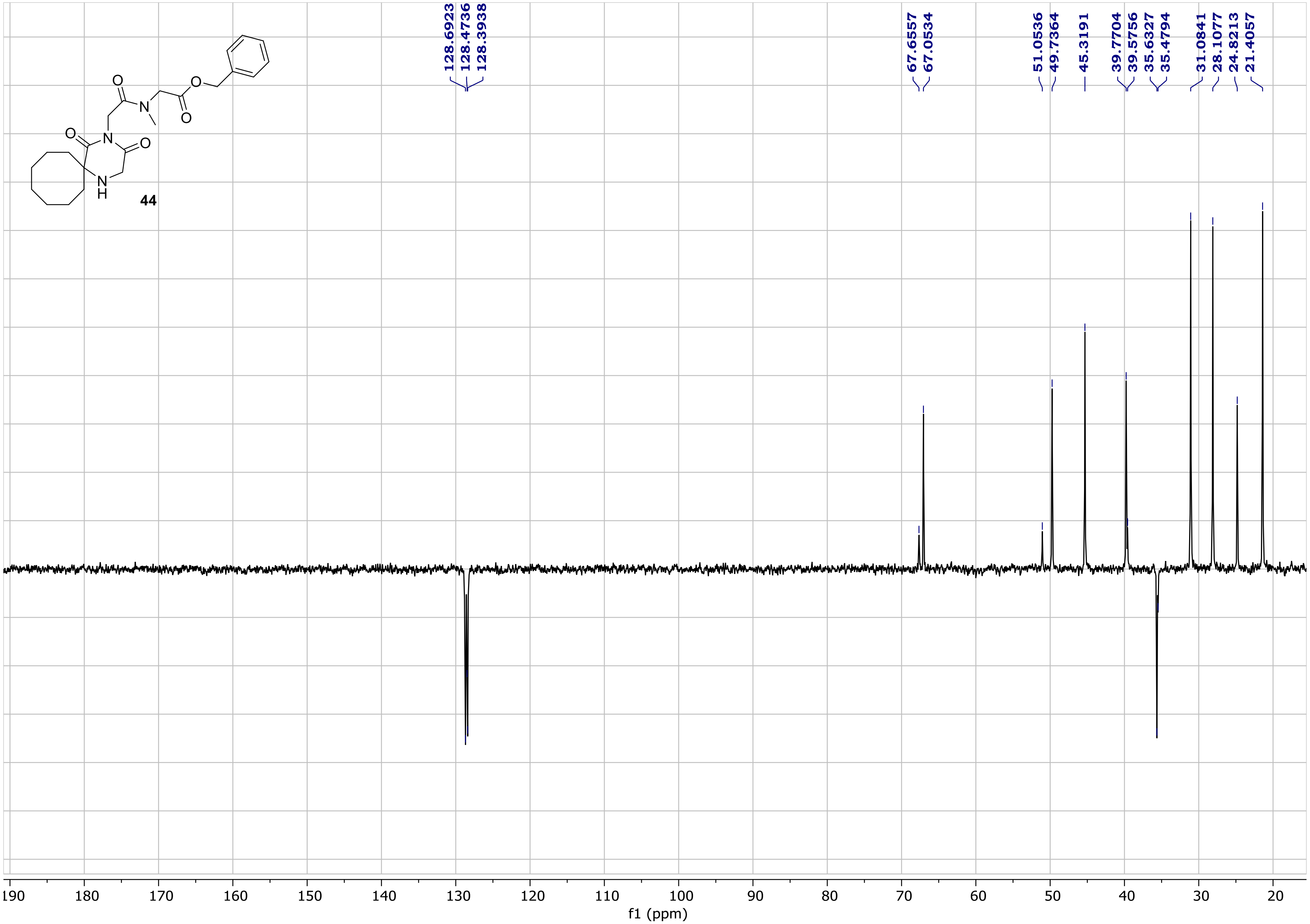

^{1}H NMR of **45** (400.13 MHz, DMSO-d_6)

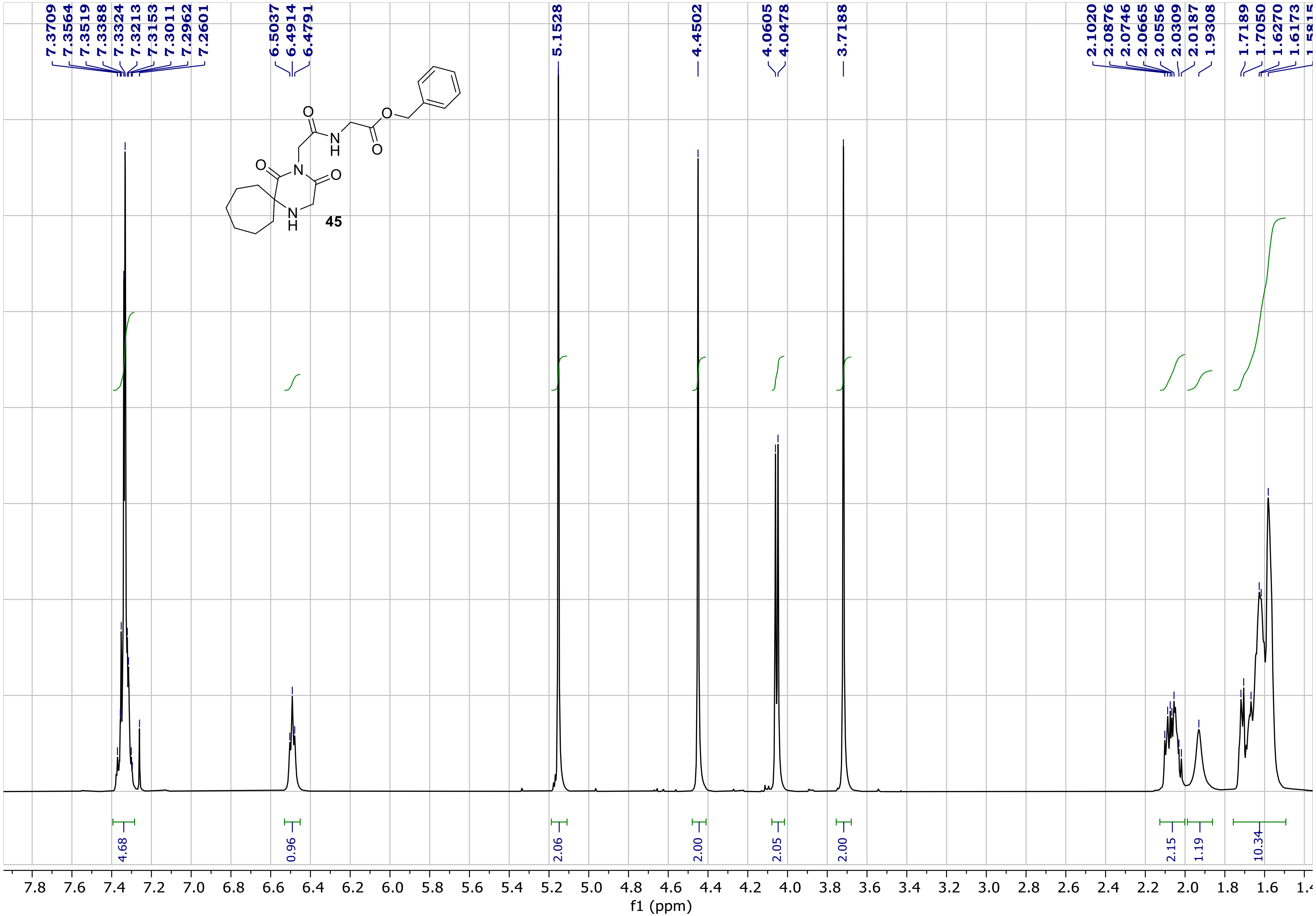

^{13}C NMR of **45** (50.32 MHz, DMSO-d_6)

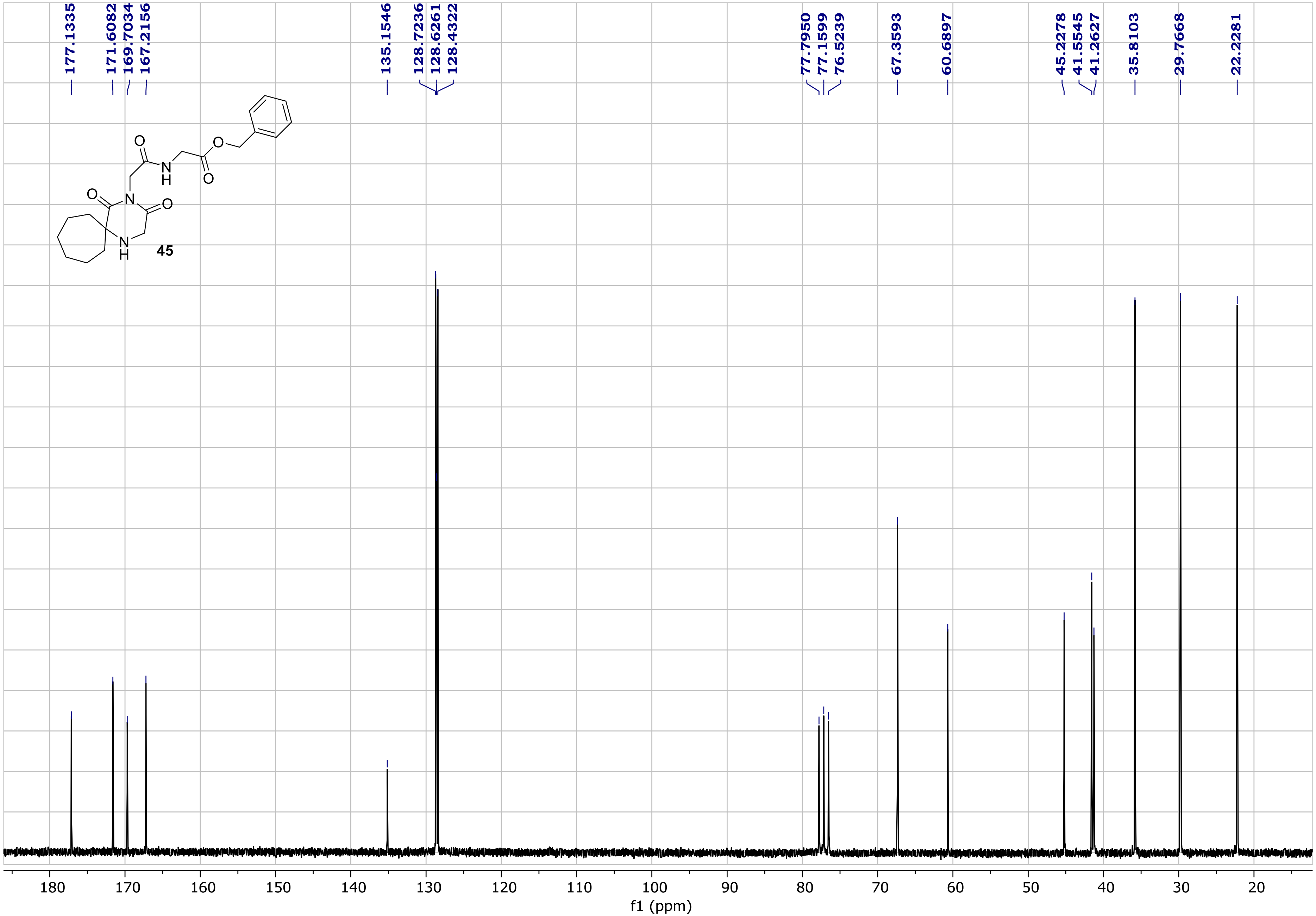

COSY NMR of **45** (400.13 MHz, DMSO-d_6)

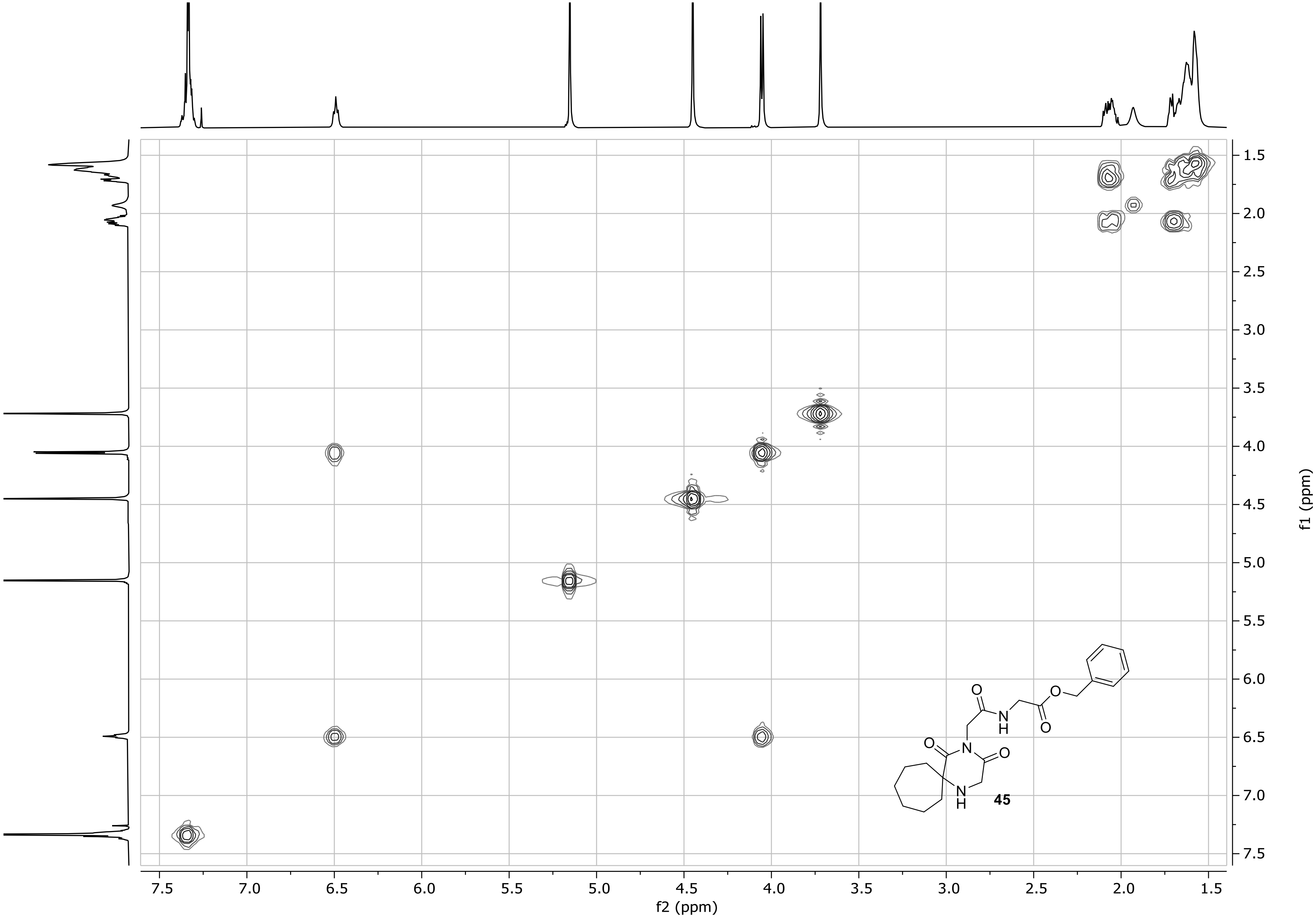

HSQC NMR of **45** (400.13 MHz, DMSO-d_6)

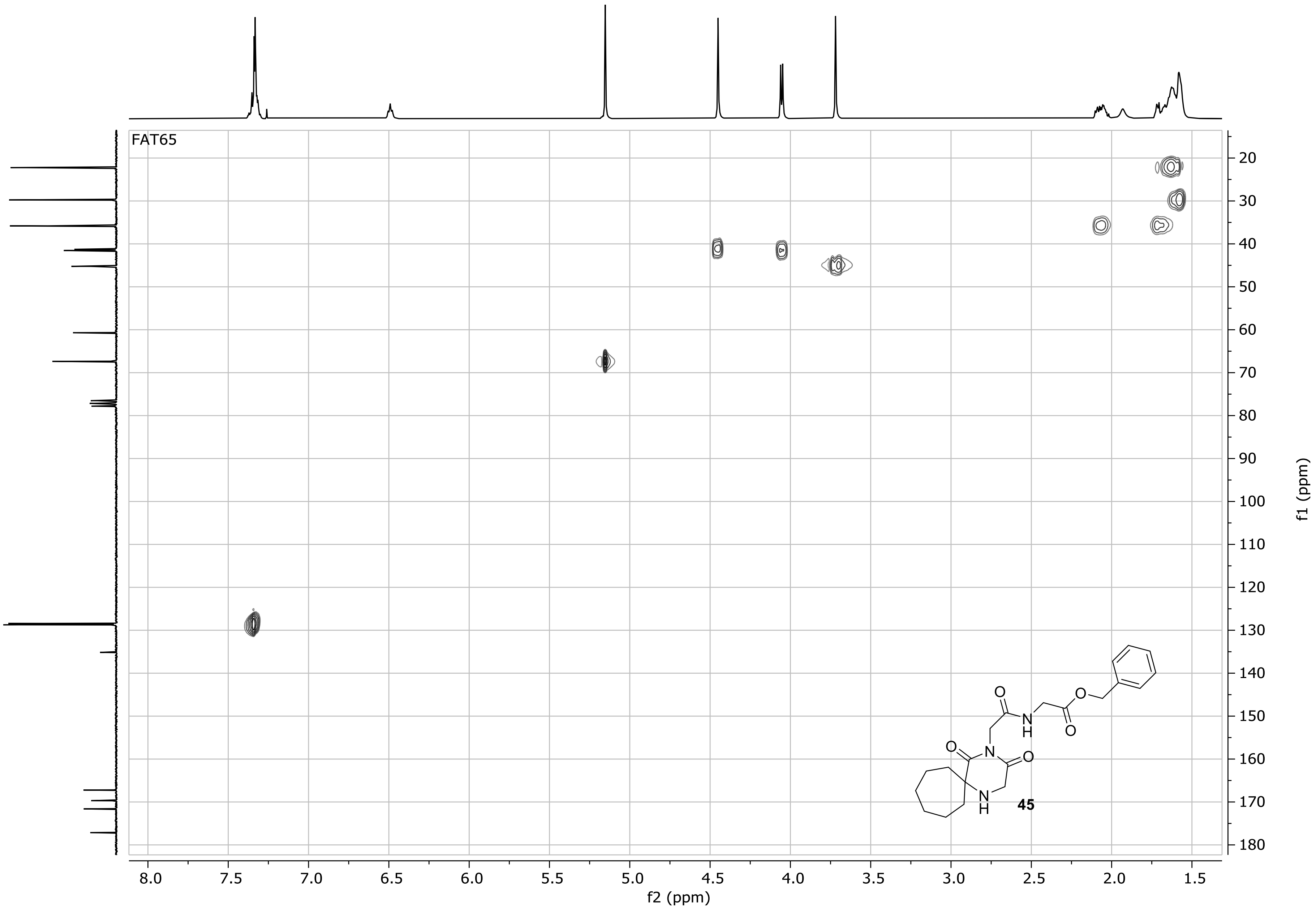

DEPT NMR of **45** (50.32 MHz, DMSO-d_6)

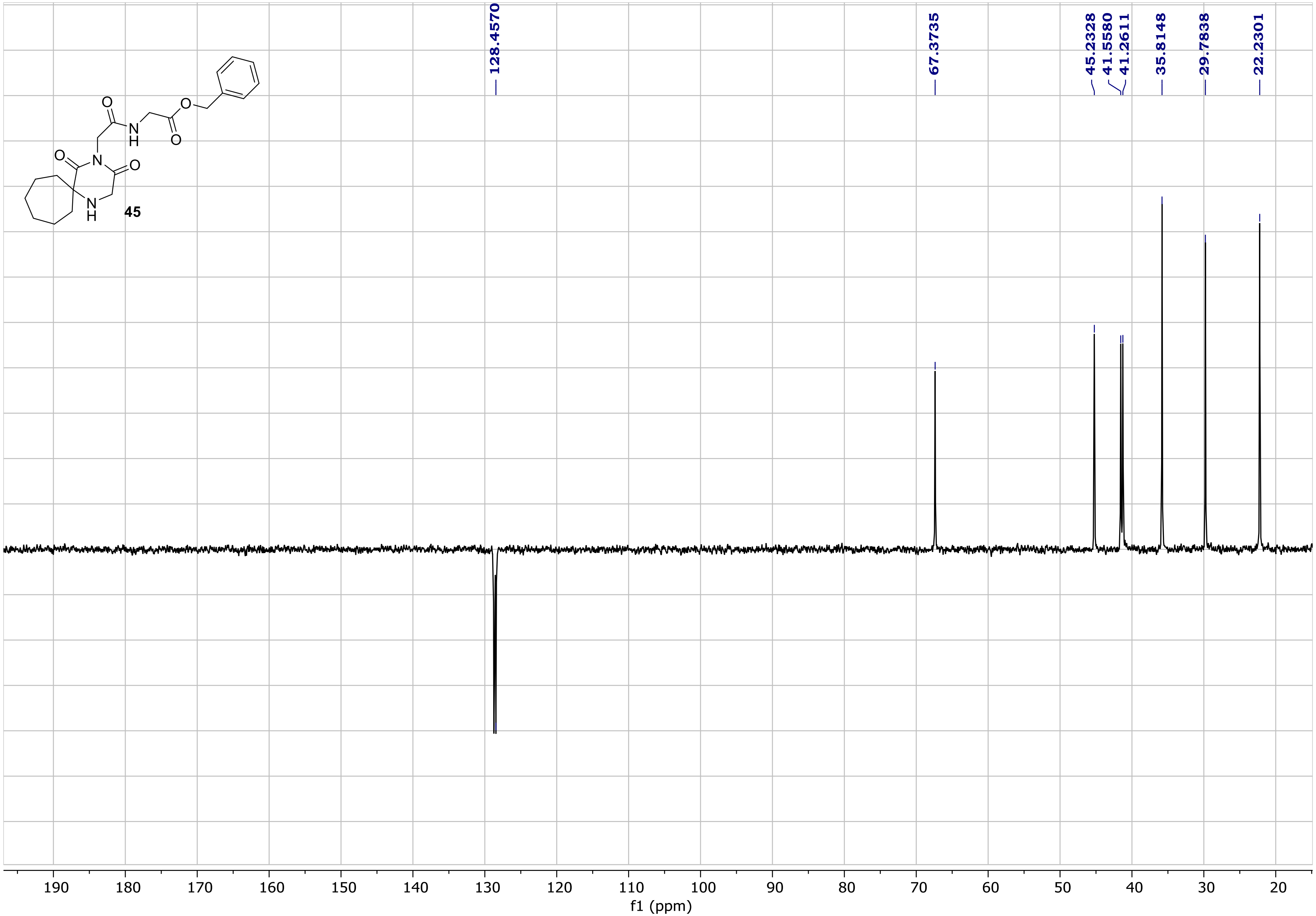

^{1}H NMR of **46** (400.13 MHz, DMSO-d_6)

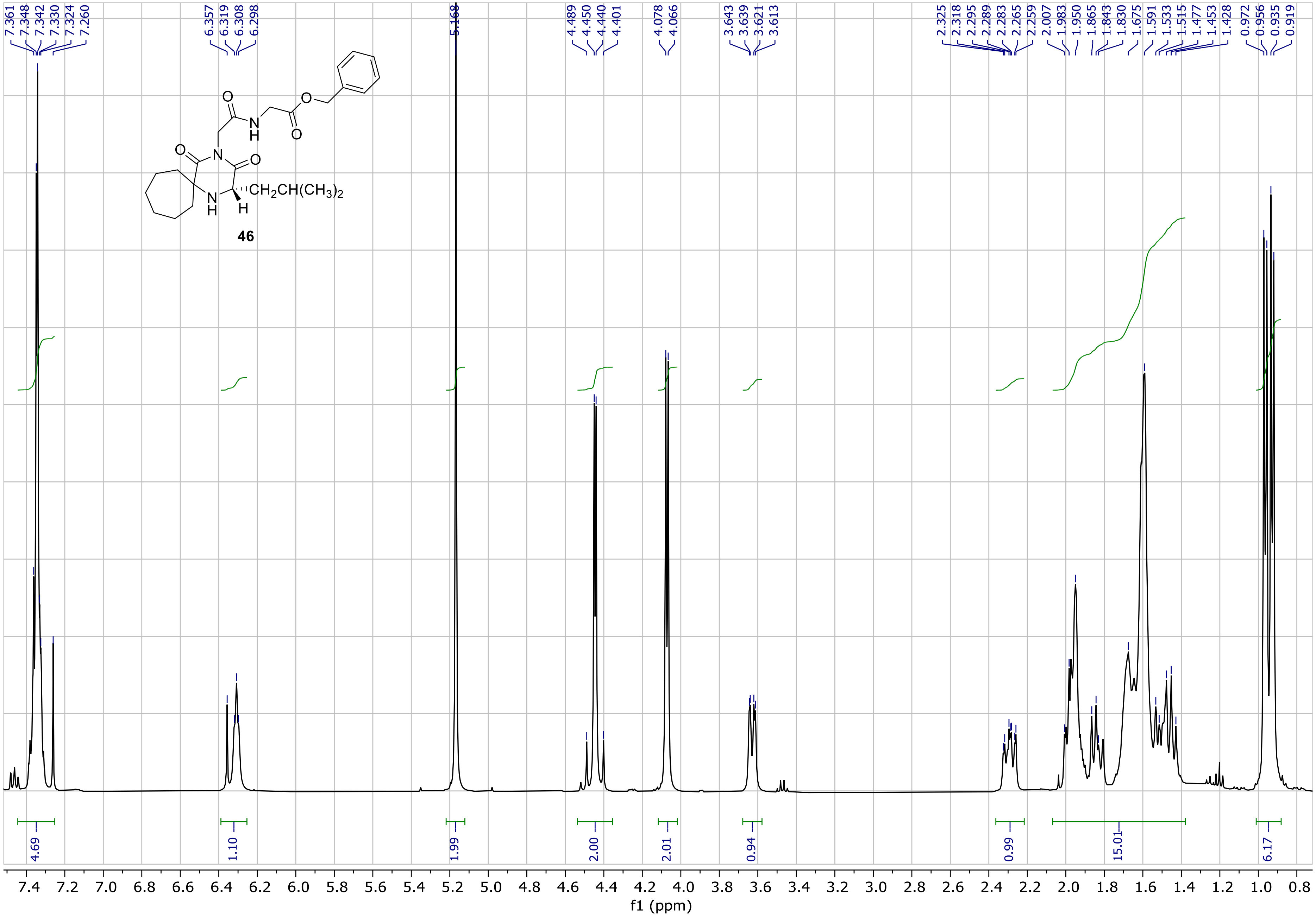

^{13}C NMR of **46** (50.32 MHz, DMSO-d_6)

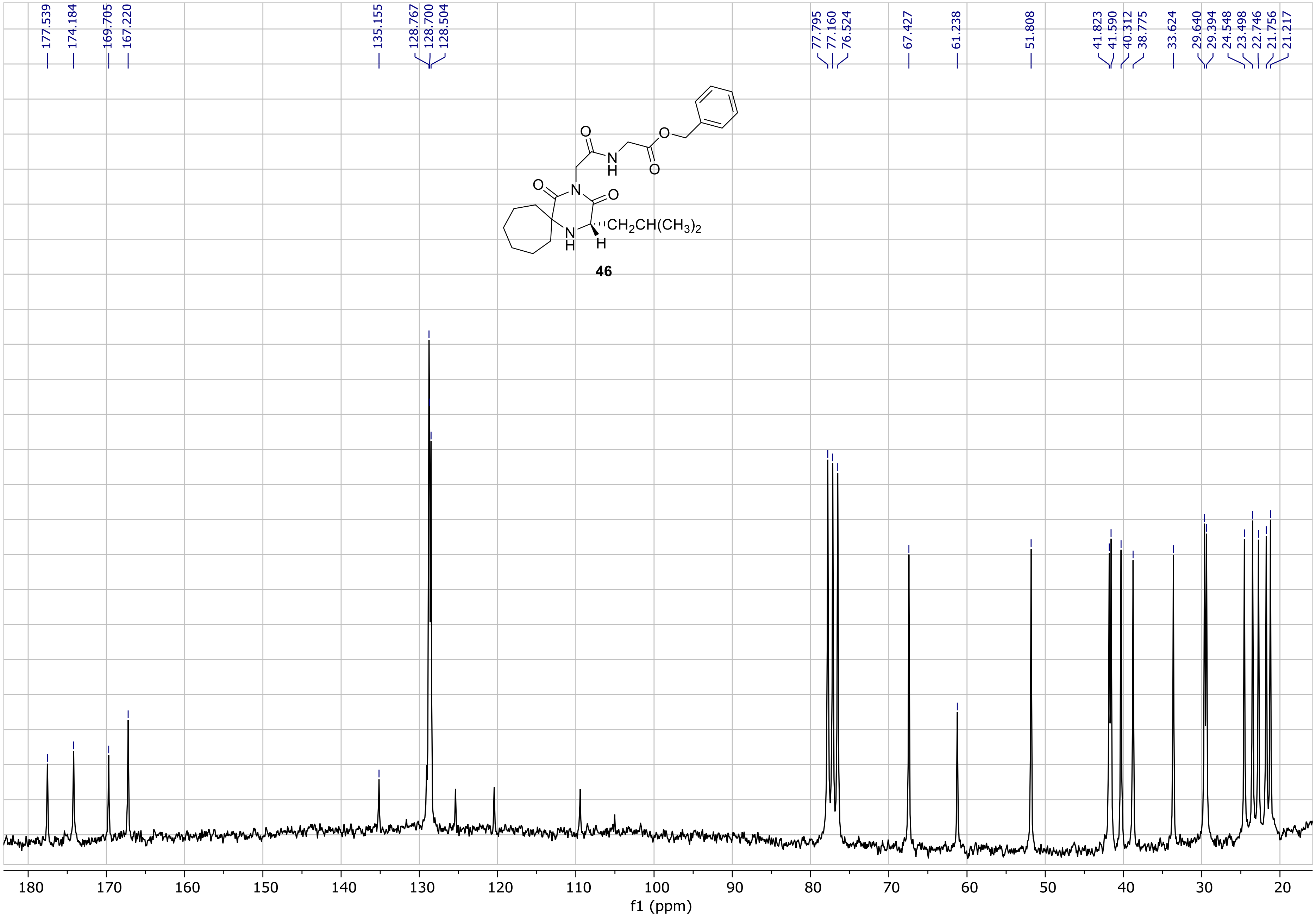

COSY NMR of **46** (400.13 MHz, DMSO-d_6)

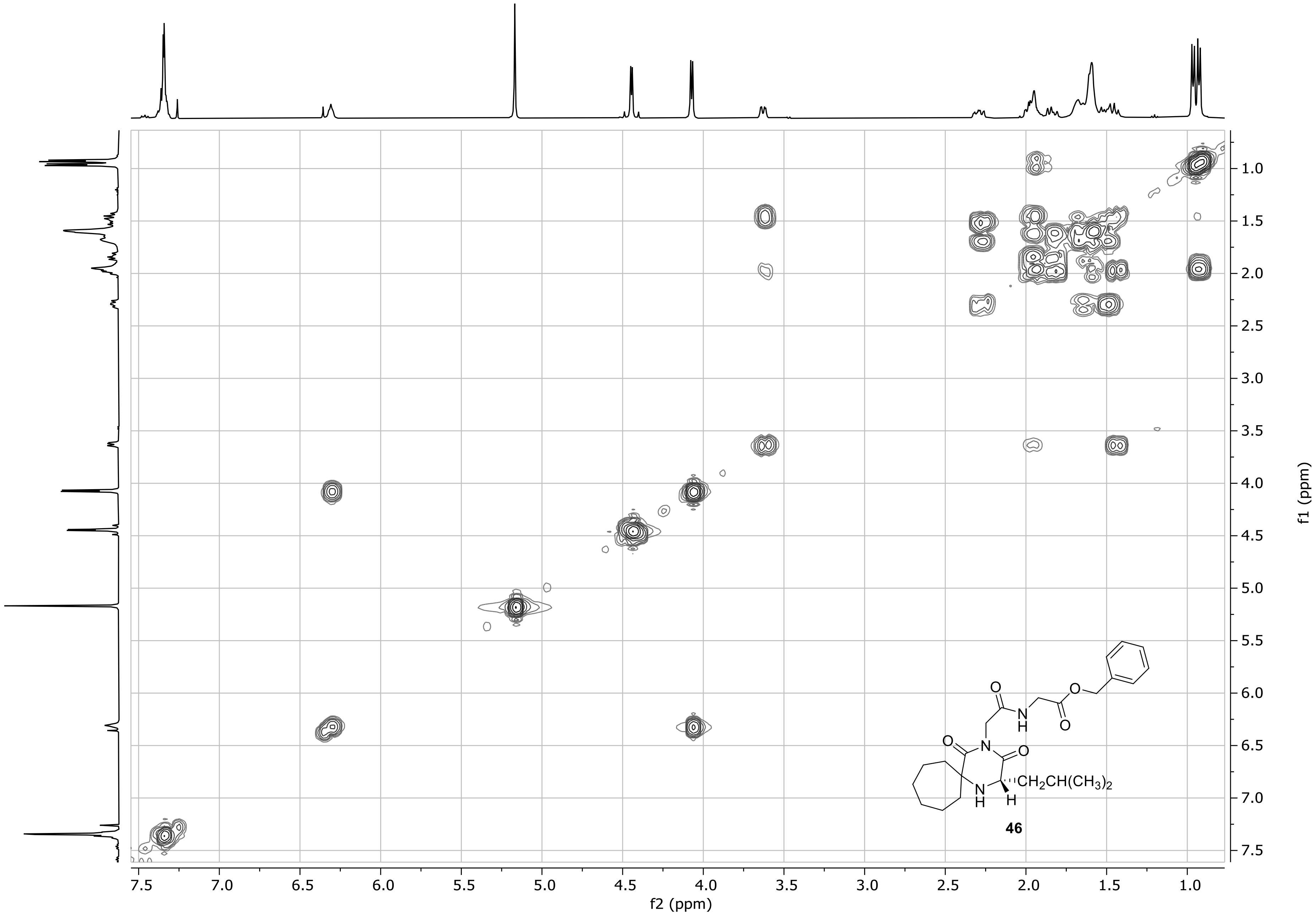

HSQC NMR of **46** (400.13 MHz, DMSO-d_6)

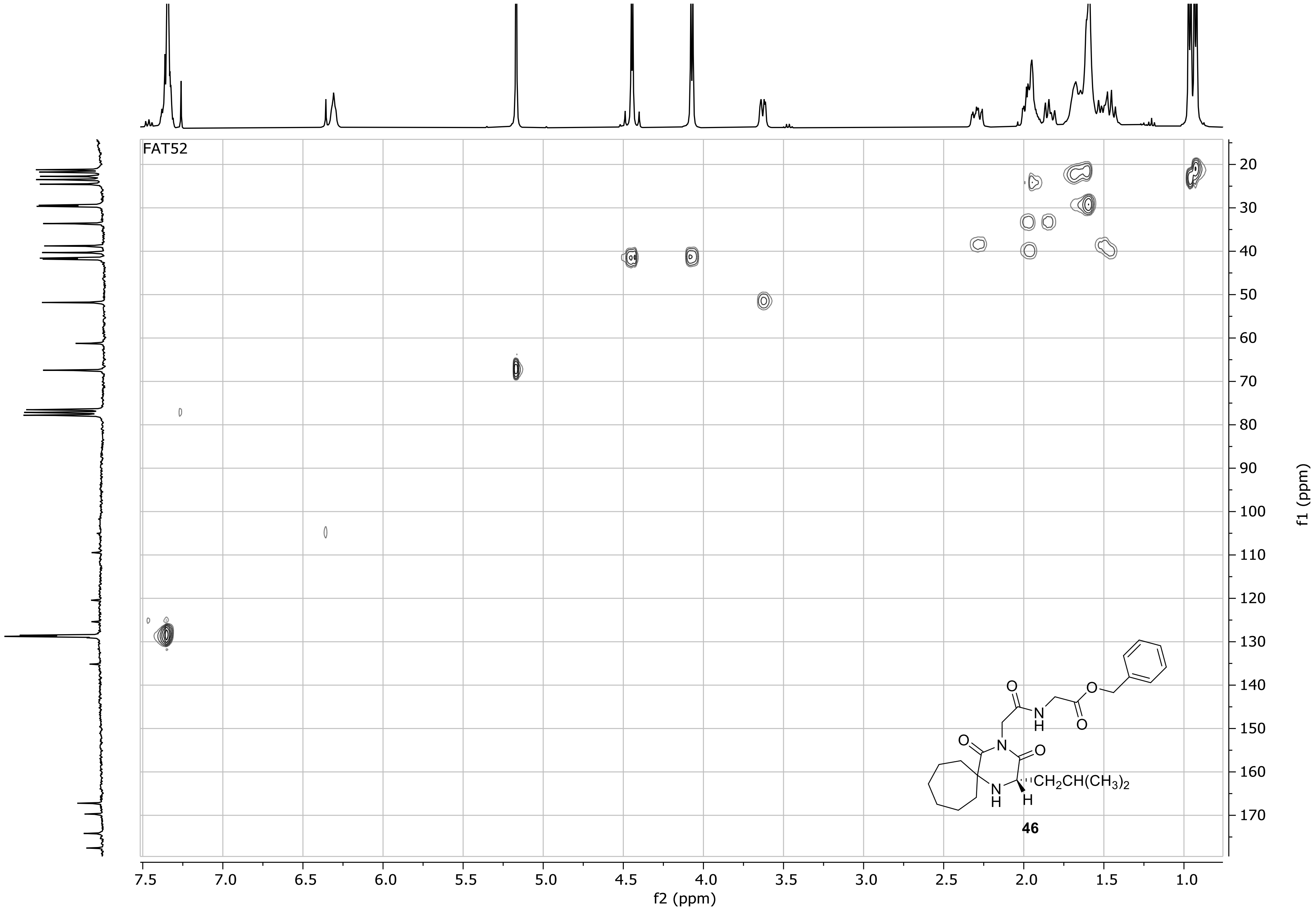

DEPT NMR of **46** (50.32 MHz, DMSO-d_6)

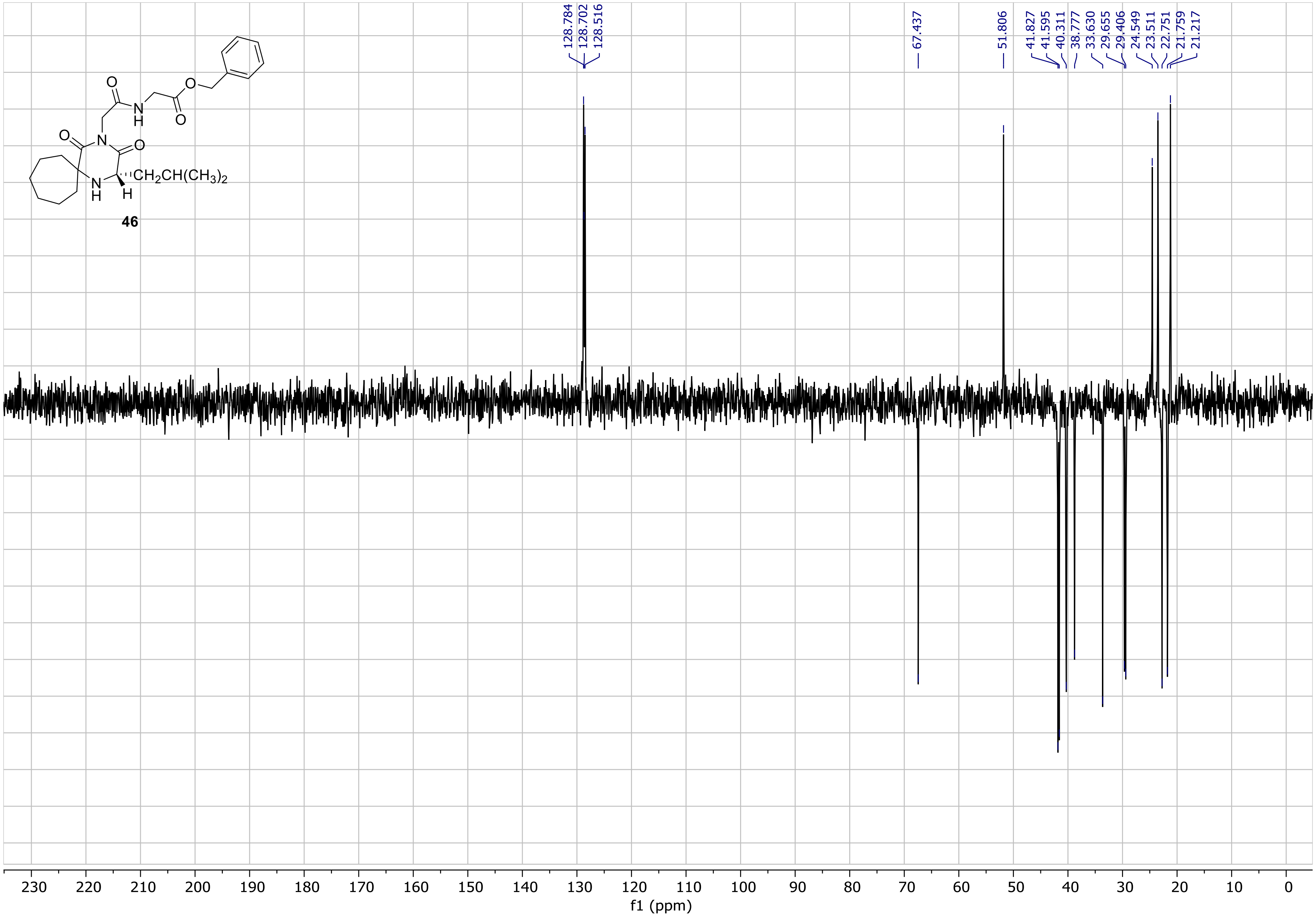

^{1}H NMR of **47** (400.13 MHz, DMSO-d_6)

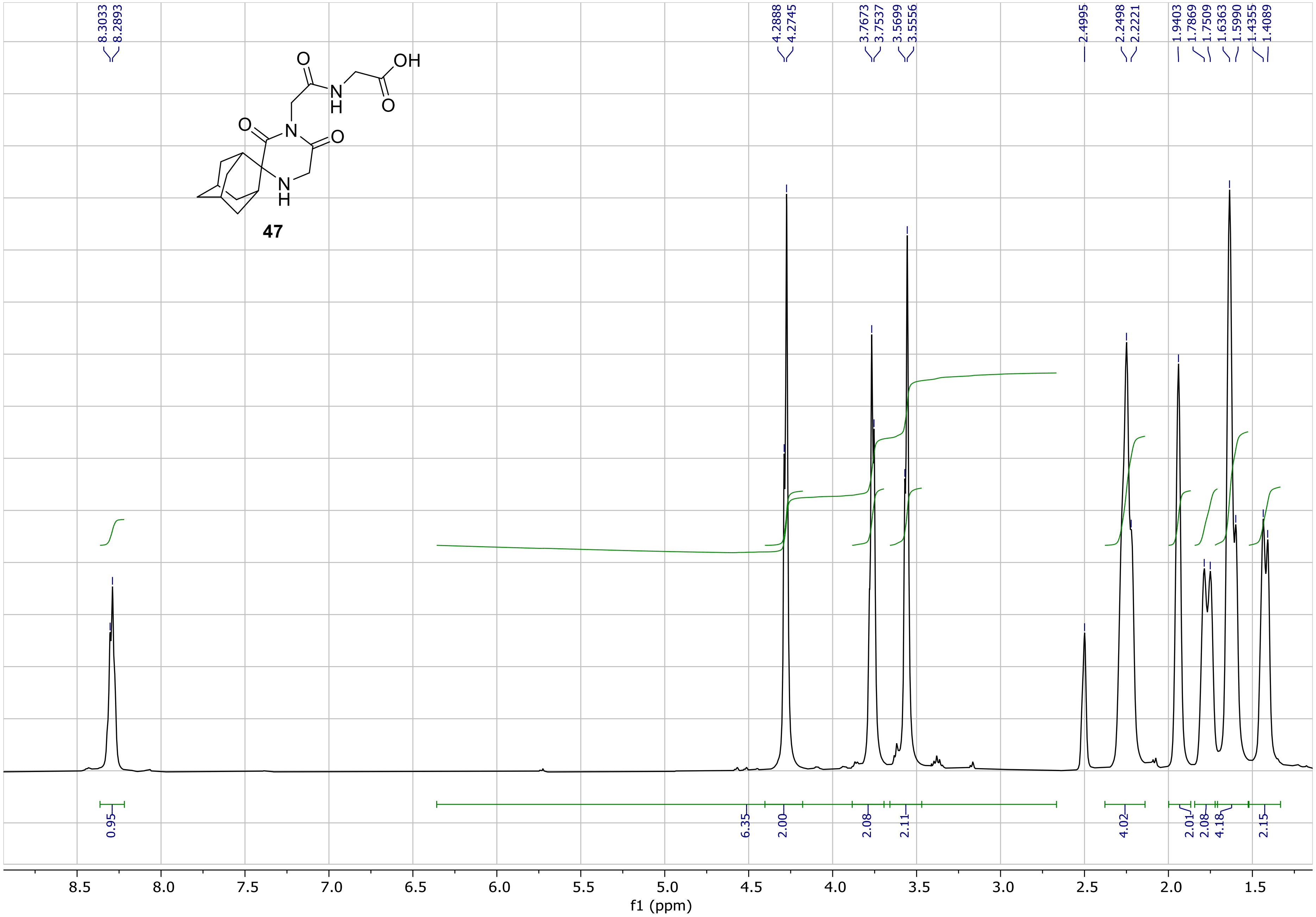

^{13}C NMR of **47** (50.32 MHz, DMSO-d_6)

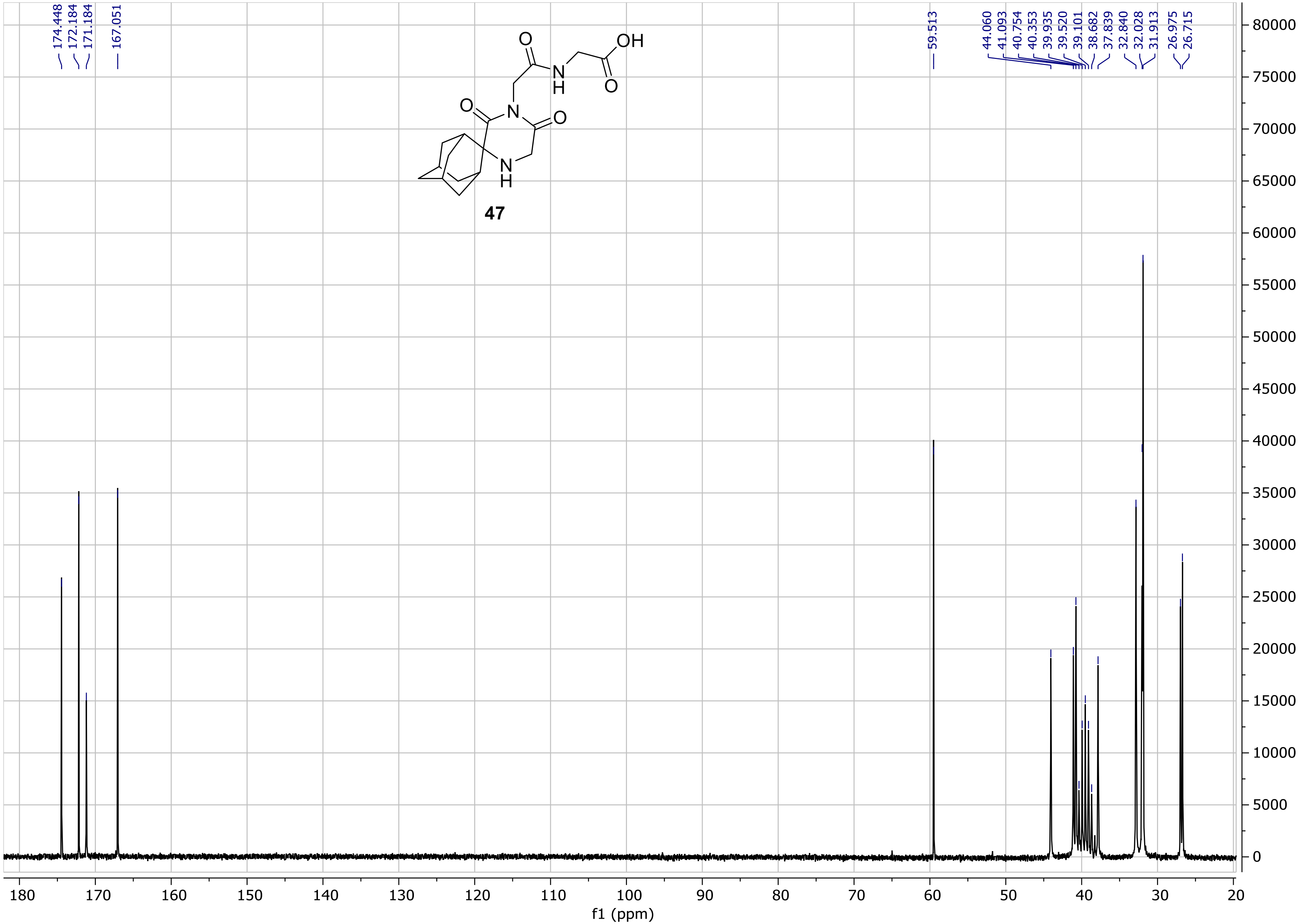

COSY NMR of **47** (400.13 MHz, DMSO-d_6)

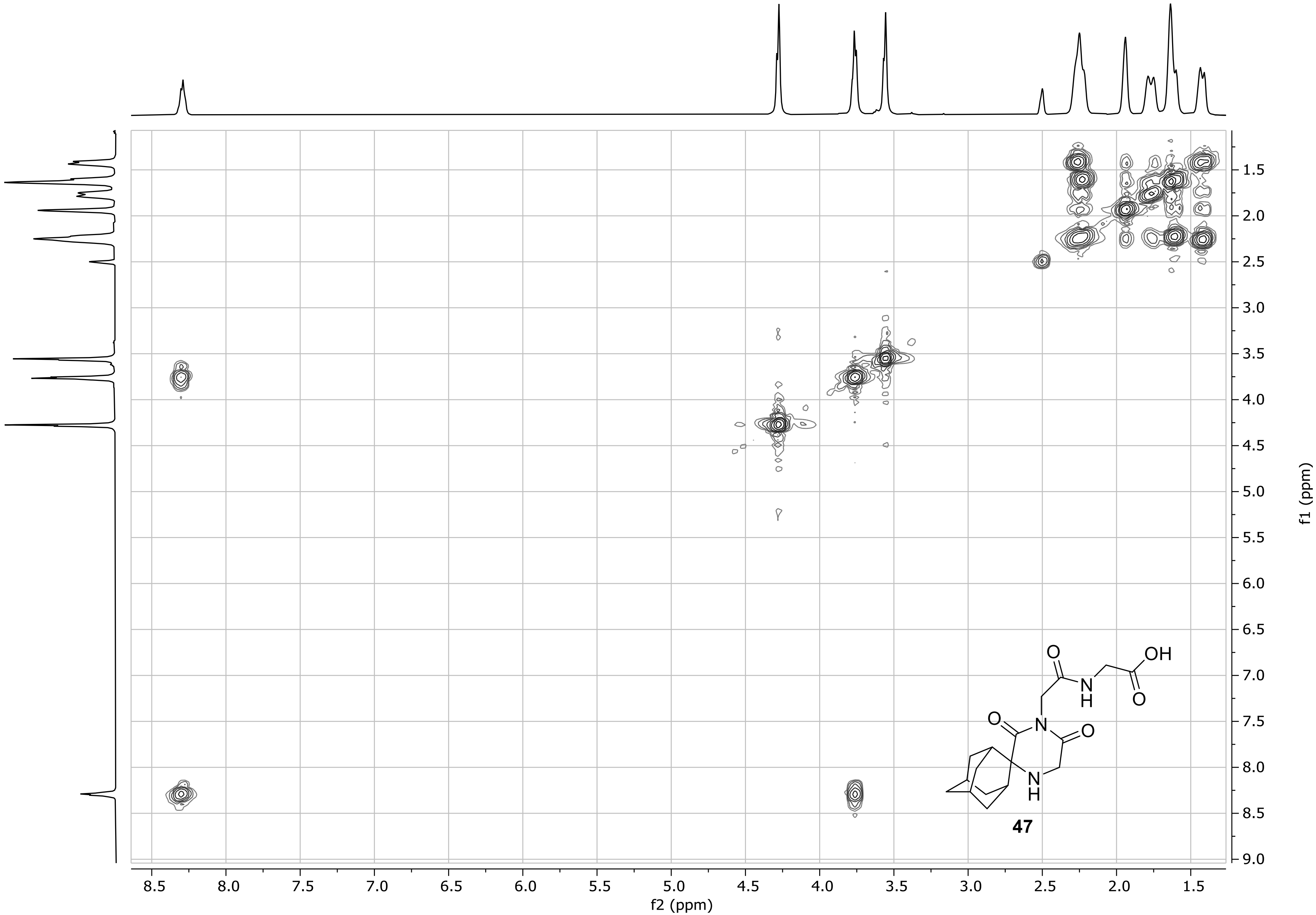

HSQC NMR of **47** (400.13 MHz, DMSO-d_6)

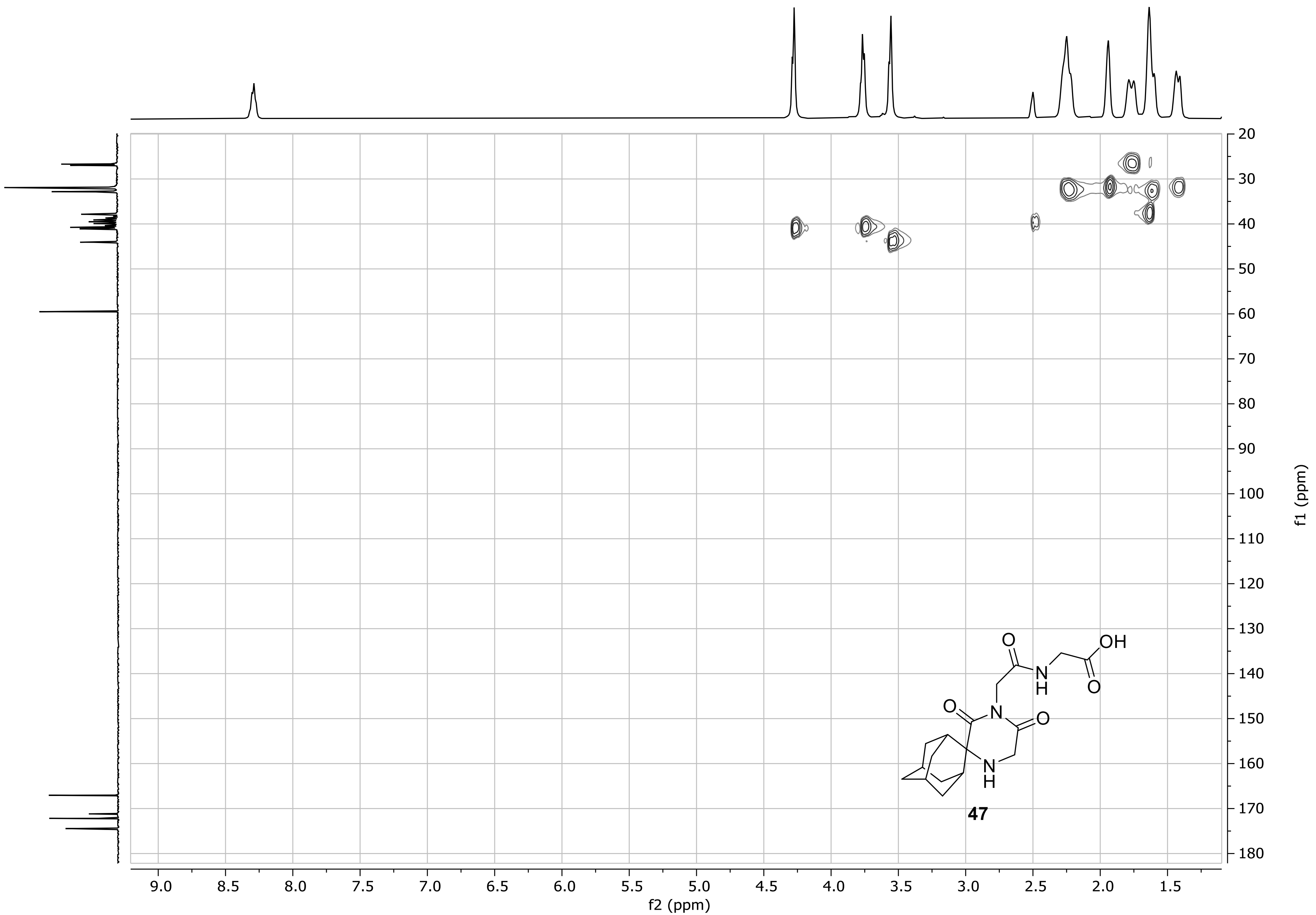

DEPT NMR of **47** (50.32 MHz, DMSO-d_6)

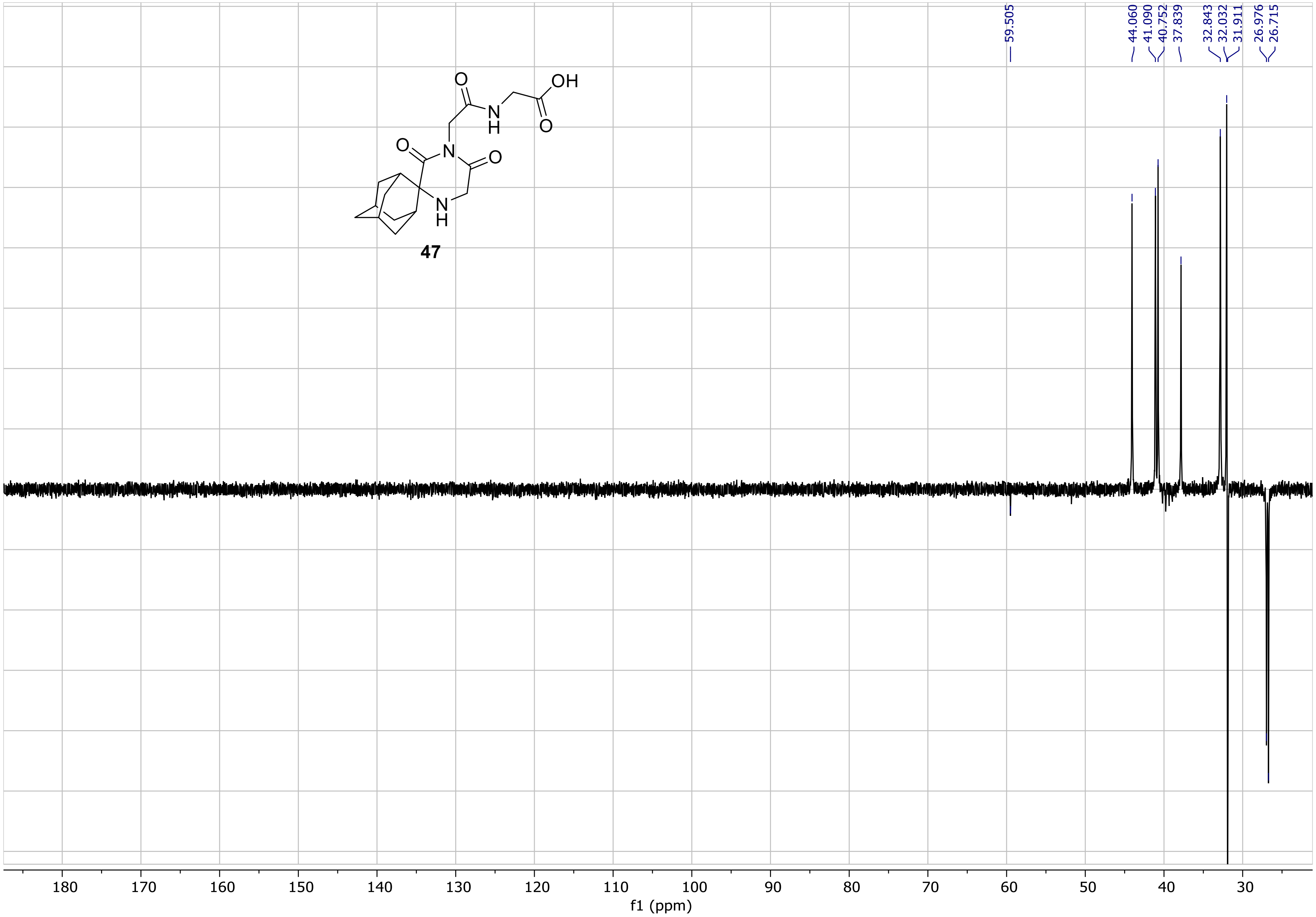

¹H NMR of **48** (600.11 MHz, $CDCl_3$)

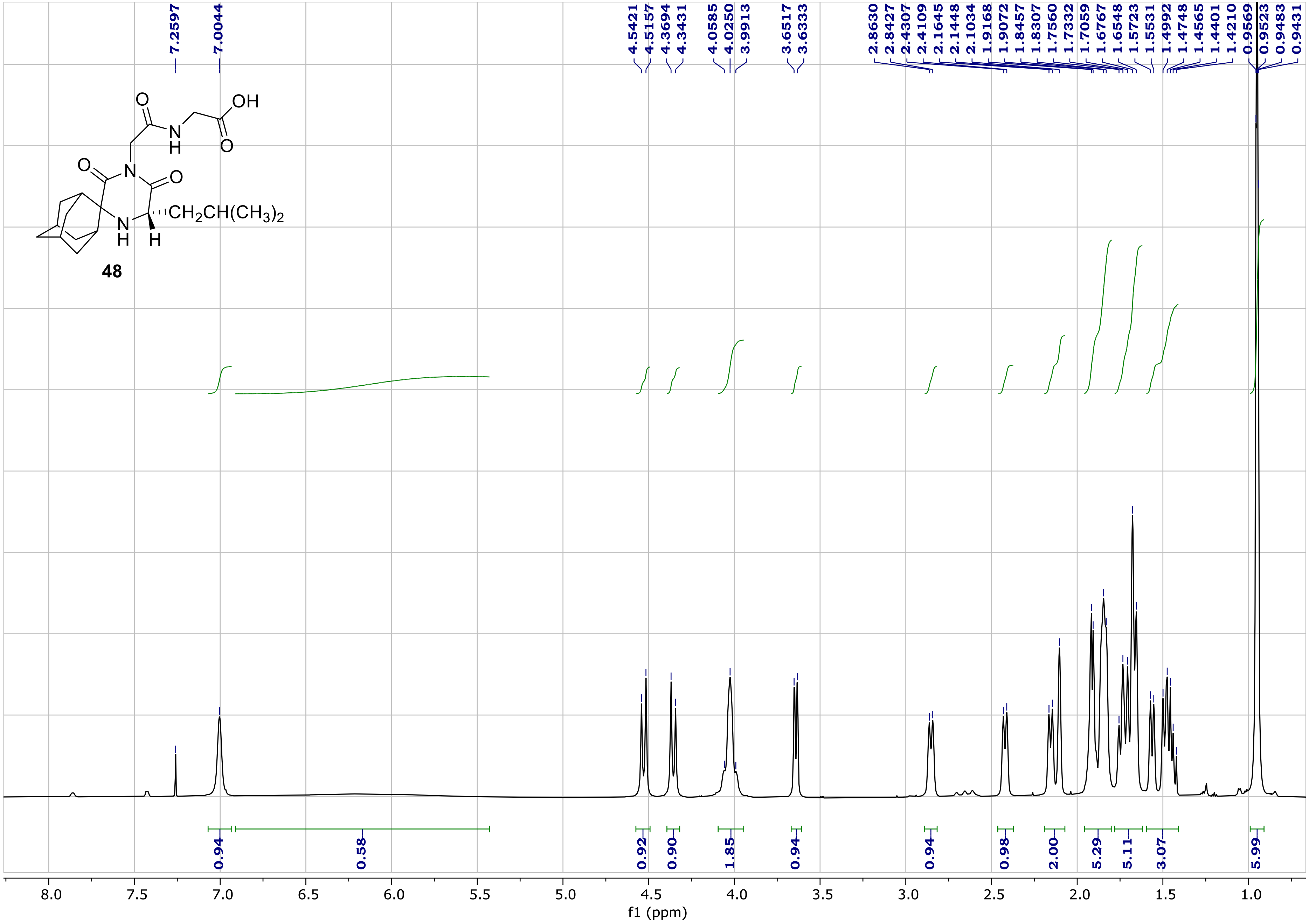

^{13}C NMR of **48** (50.32 MHz, $CDCl_3$)

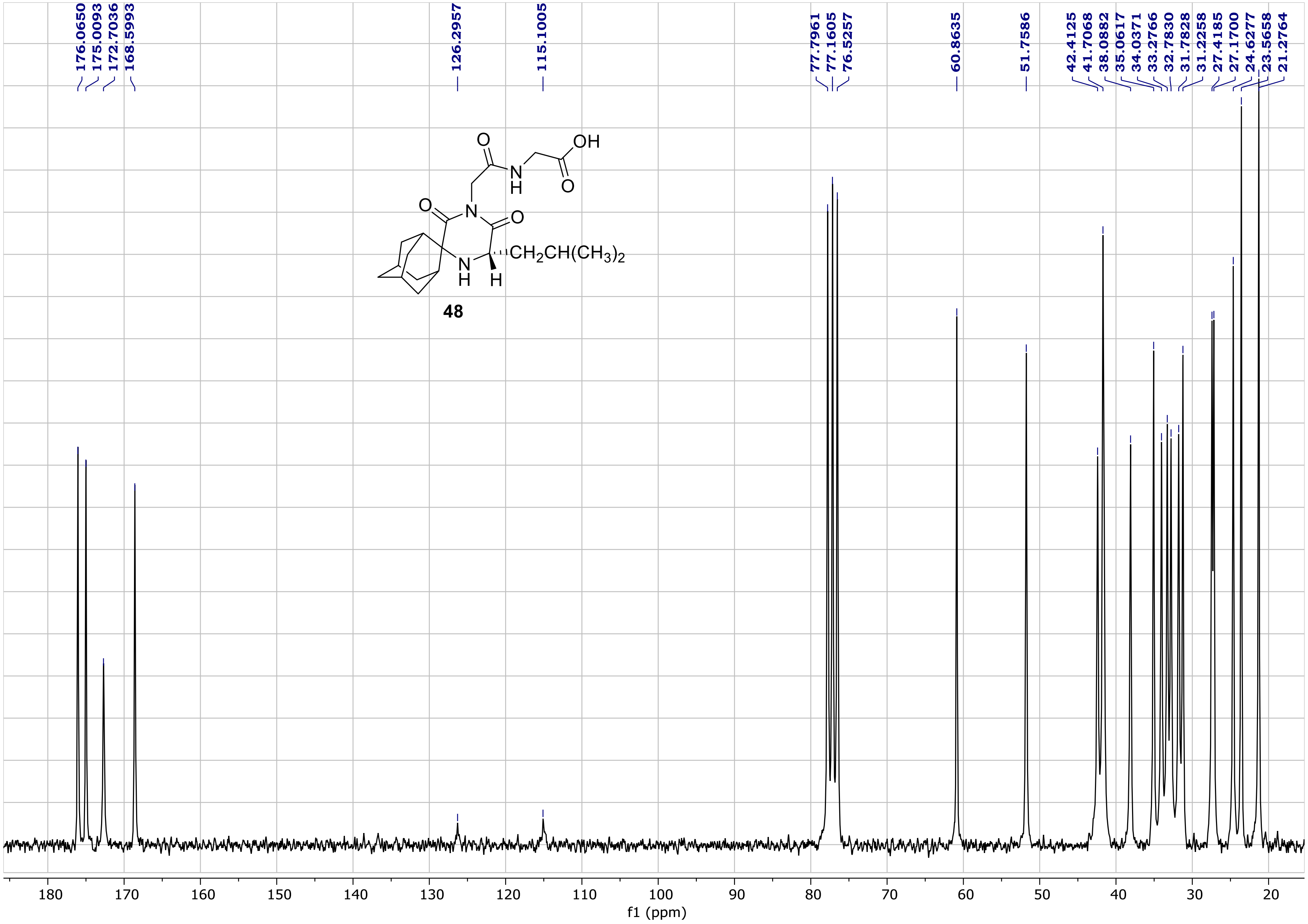

COSY NMR of **48** (600.11 MHz, $CDCl_3$)

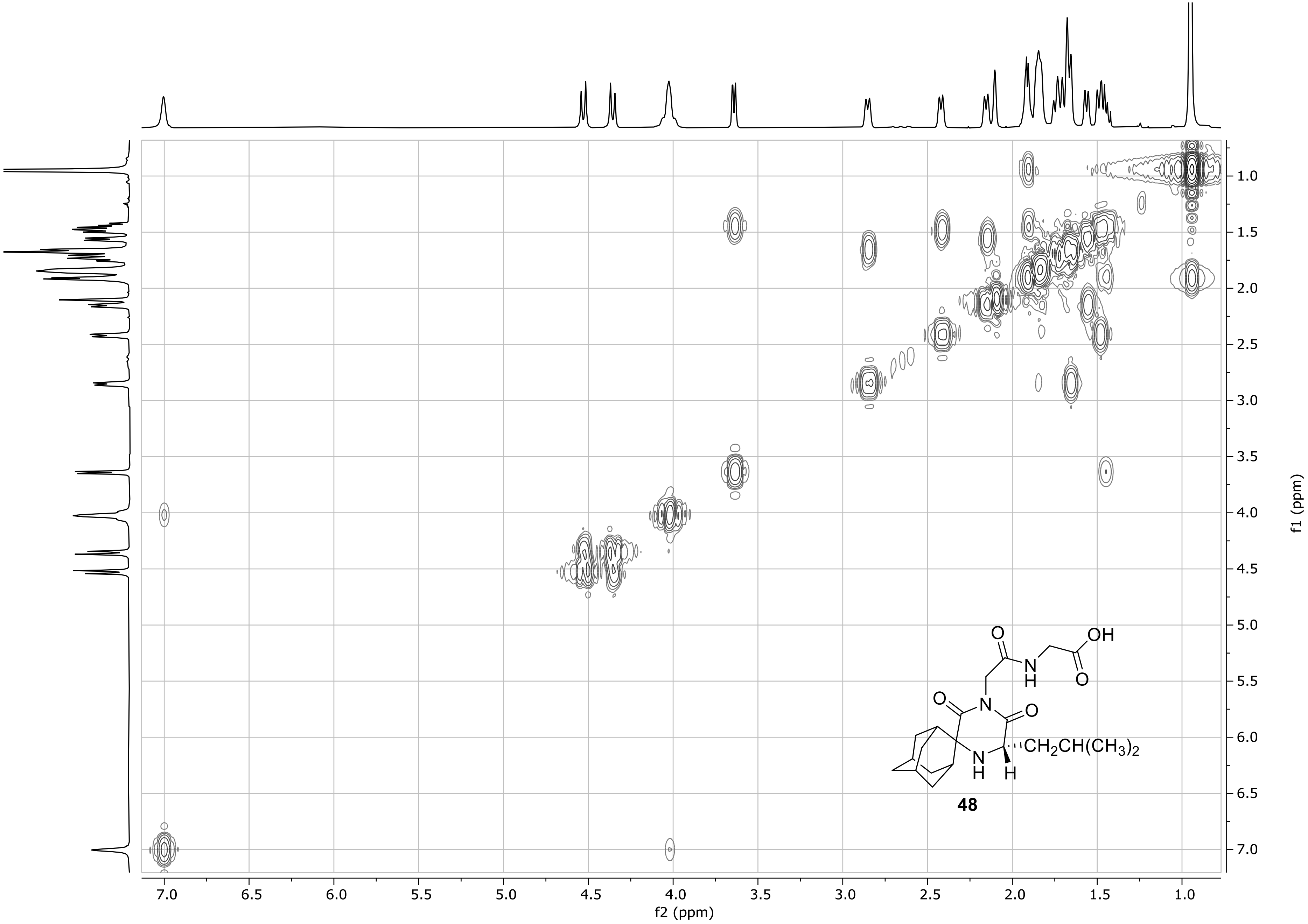

HSQC-DEPT NMR of **48** (600.11 MHz, $CDCl_3$)

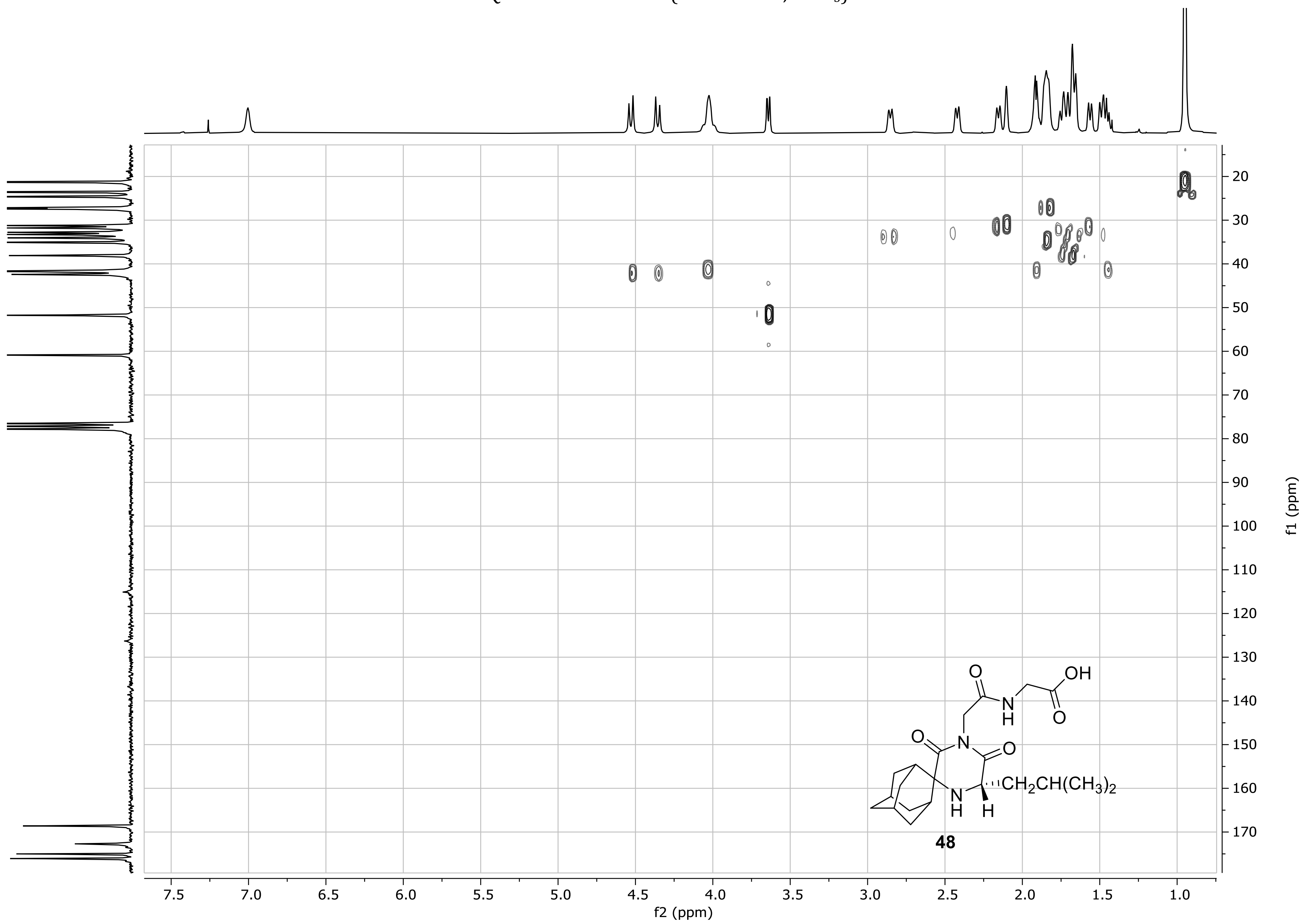

DEPT NMR of **48** (50.32 MHz, $CDCl_3$)

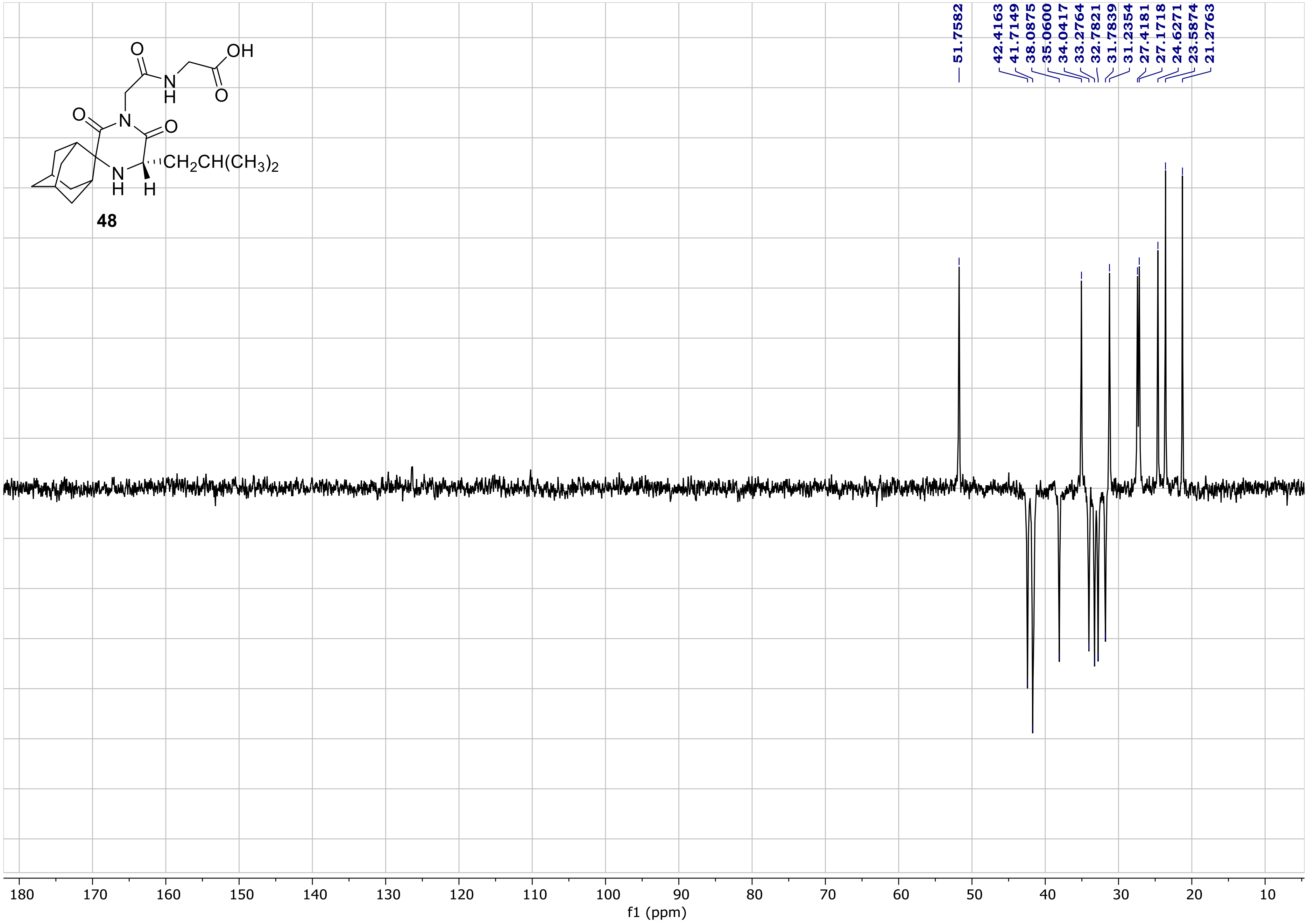

^{1}H NMR of **49** (600.11 MHz, $CDCl_3$)

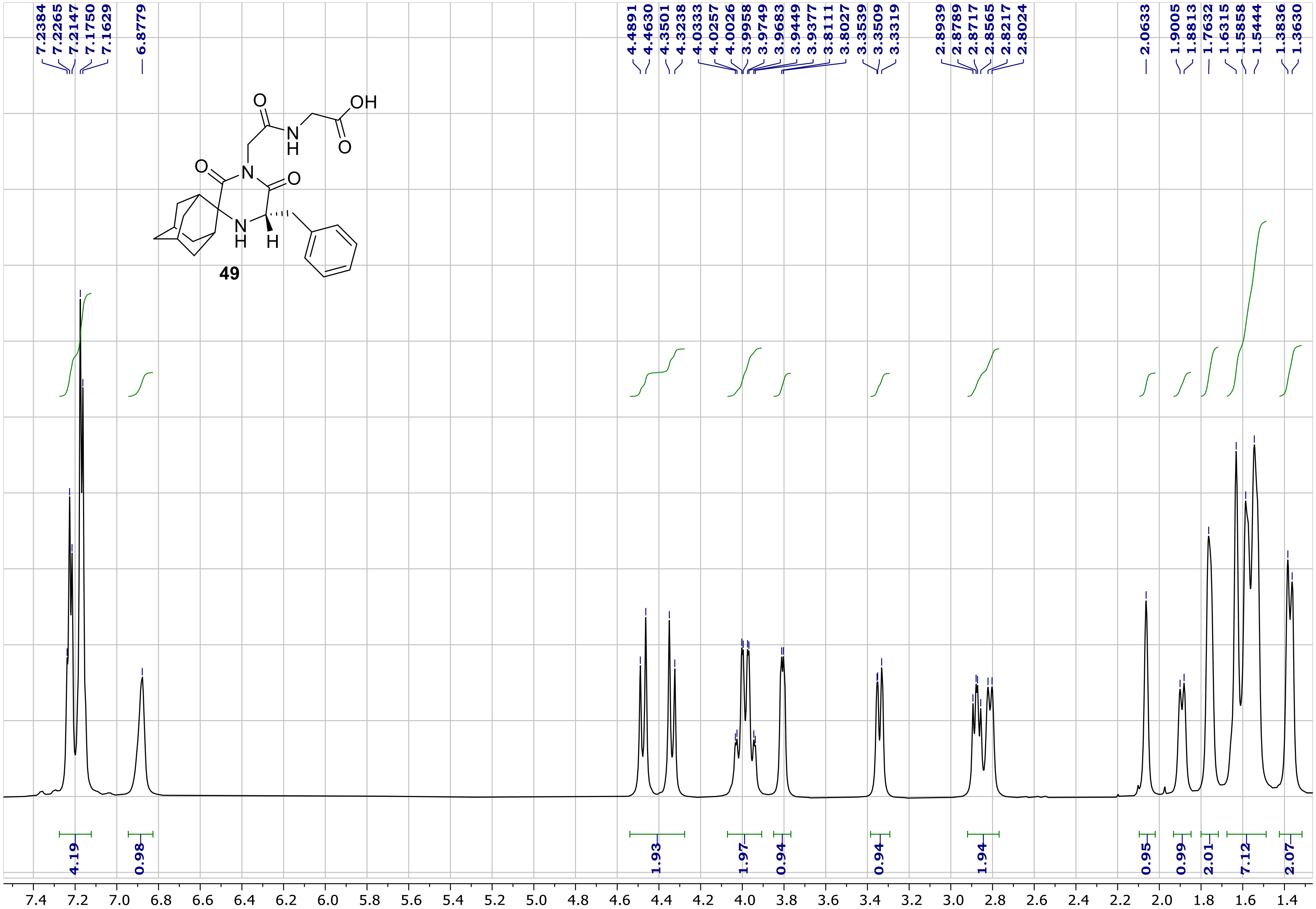

^{13}C NMR of **49** (50.32 MHz, $CDCl_3$)

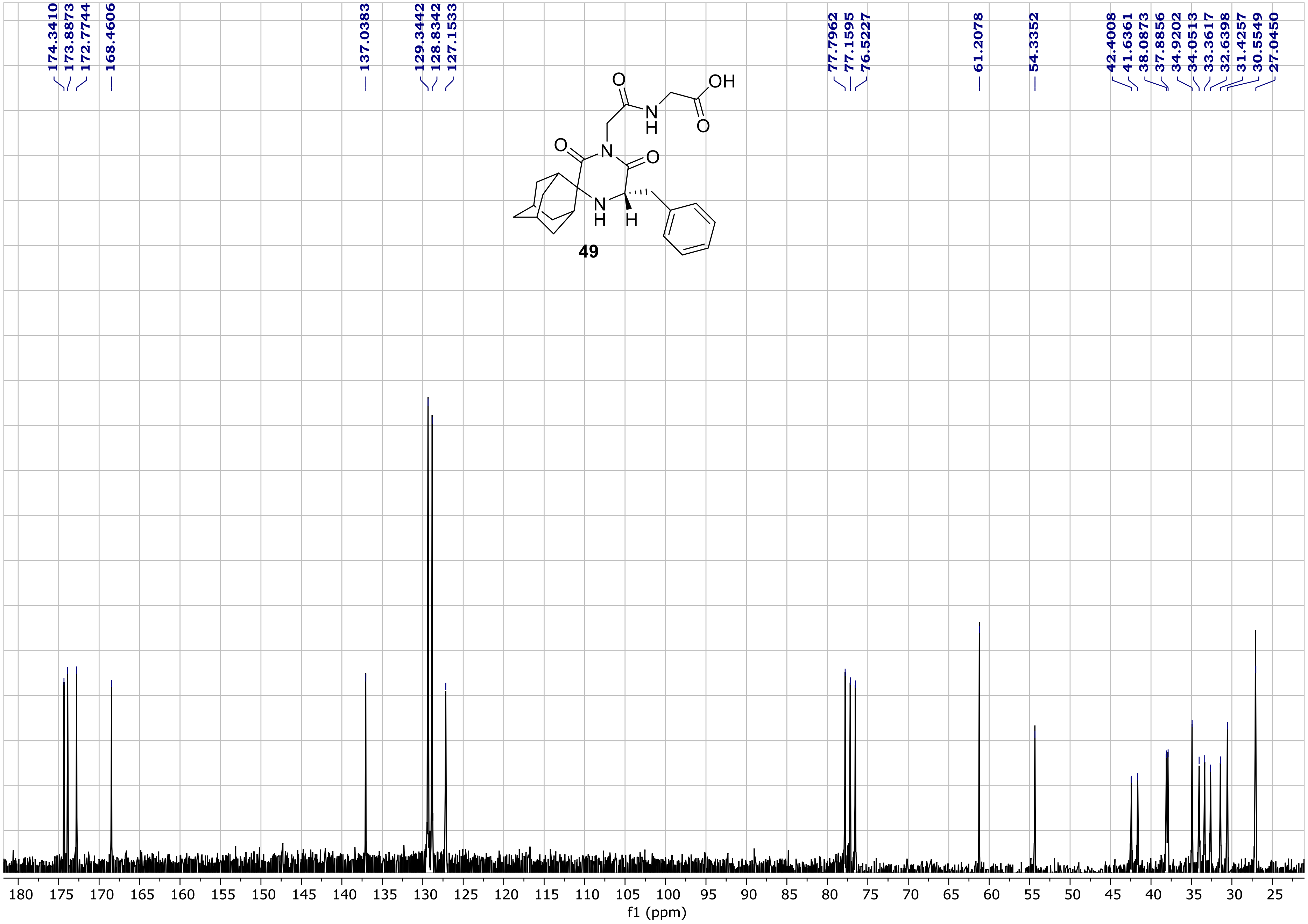

COSY NMR of **49** (600.11 MHz, $CDCl_3$)

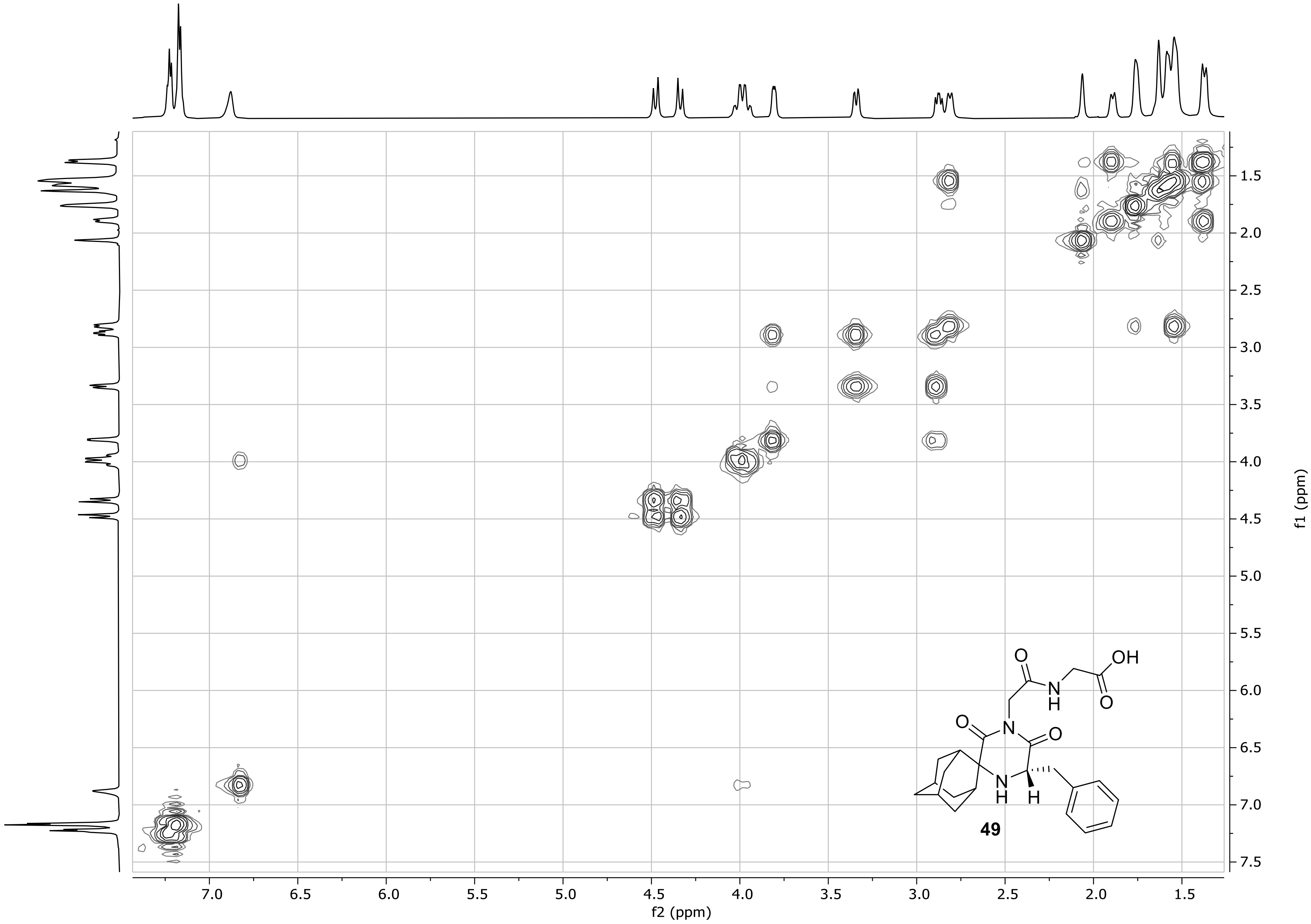

HSQC-DEPT NMR of **49** (600.11 MHz, $CDCl_3$)

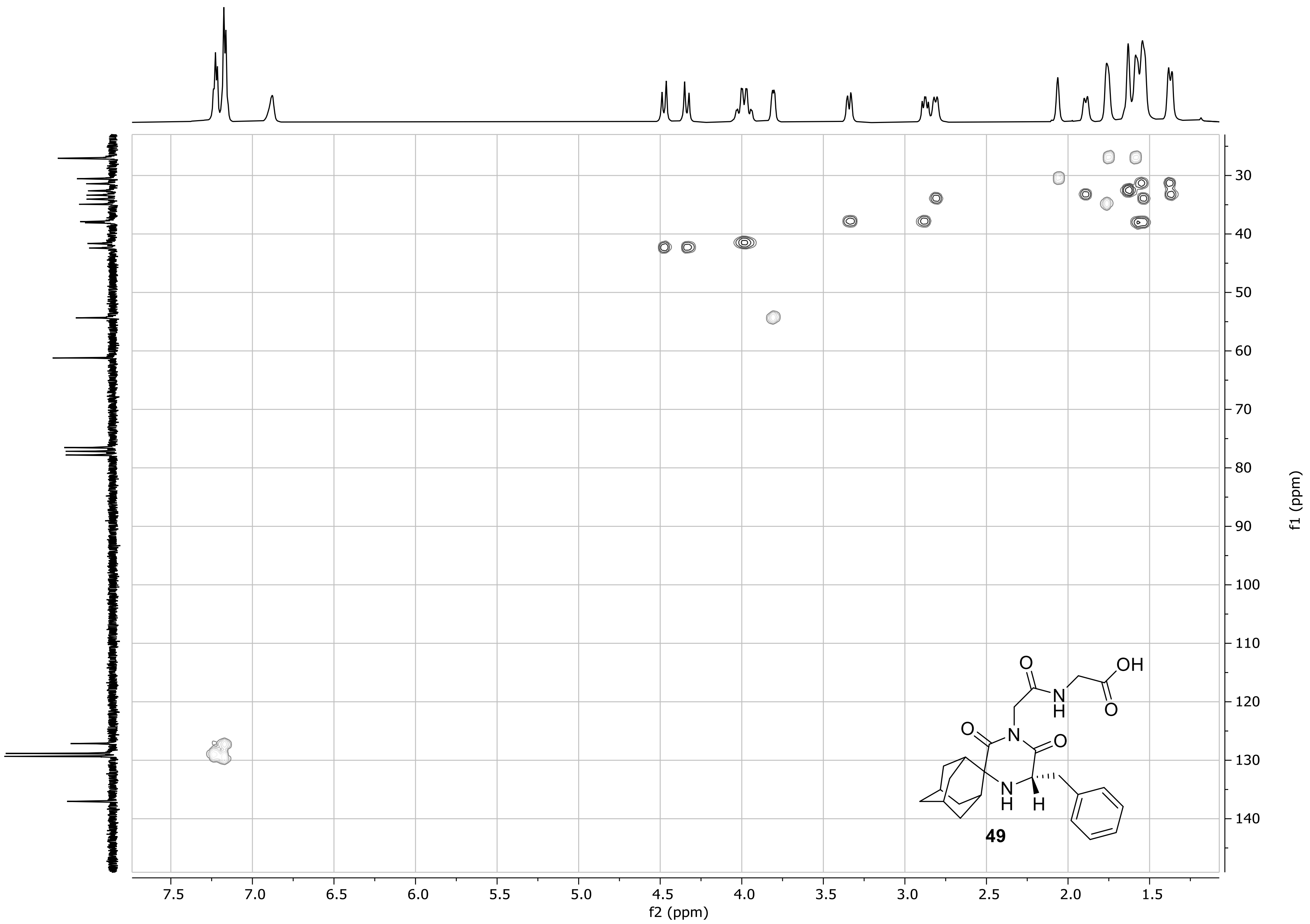

DEPT NMR of **49** (50.32 MHz, $CDCl_3$)

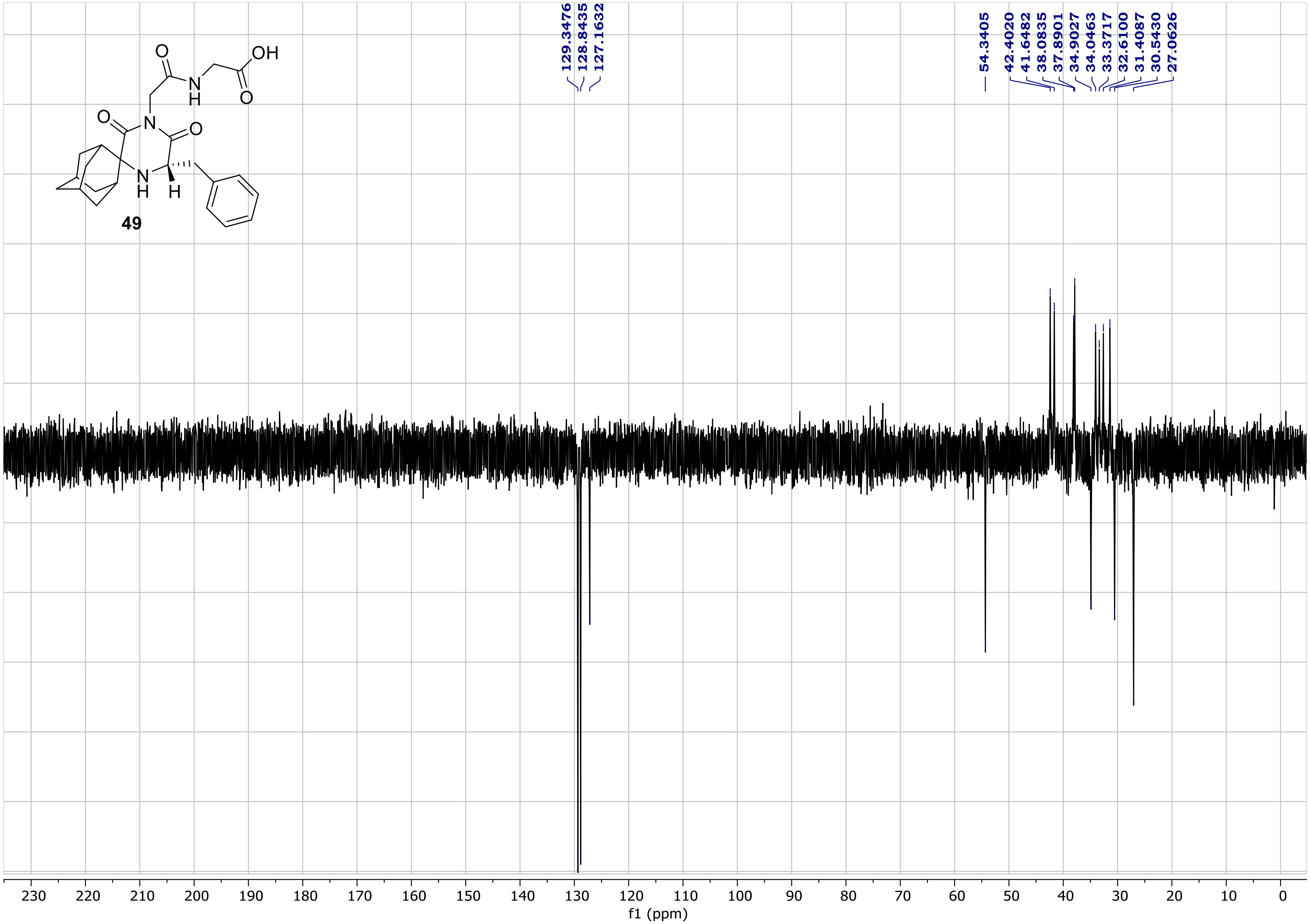

^{1}H NMR of **50** (400.11 MHz, $CDCl_3$)

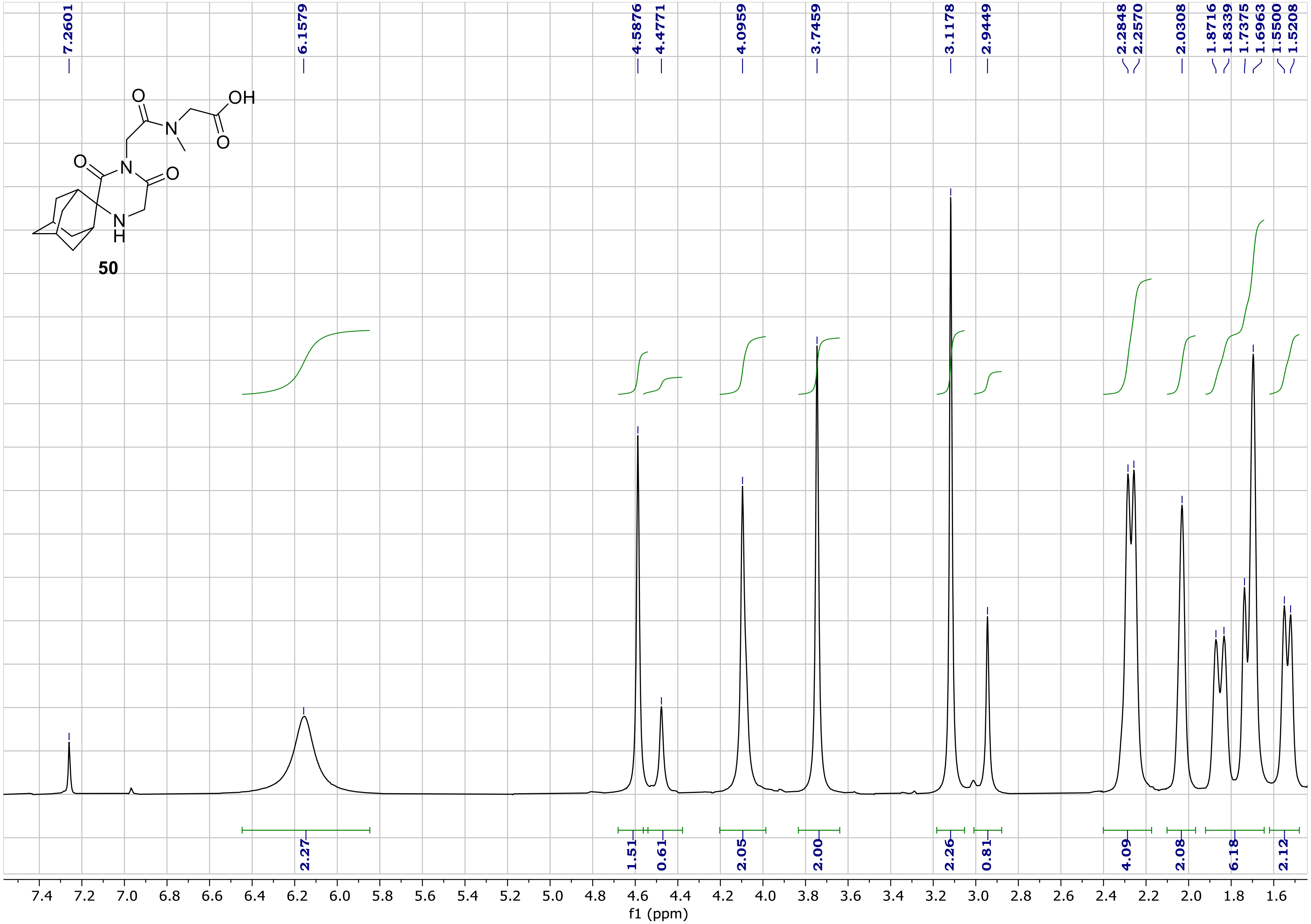

^{13}C NMR of **50** (50.32 MHz, $CDCl_3$)

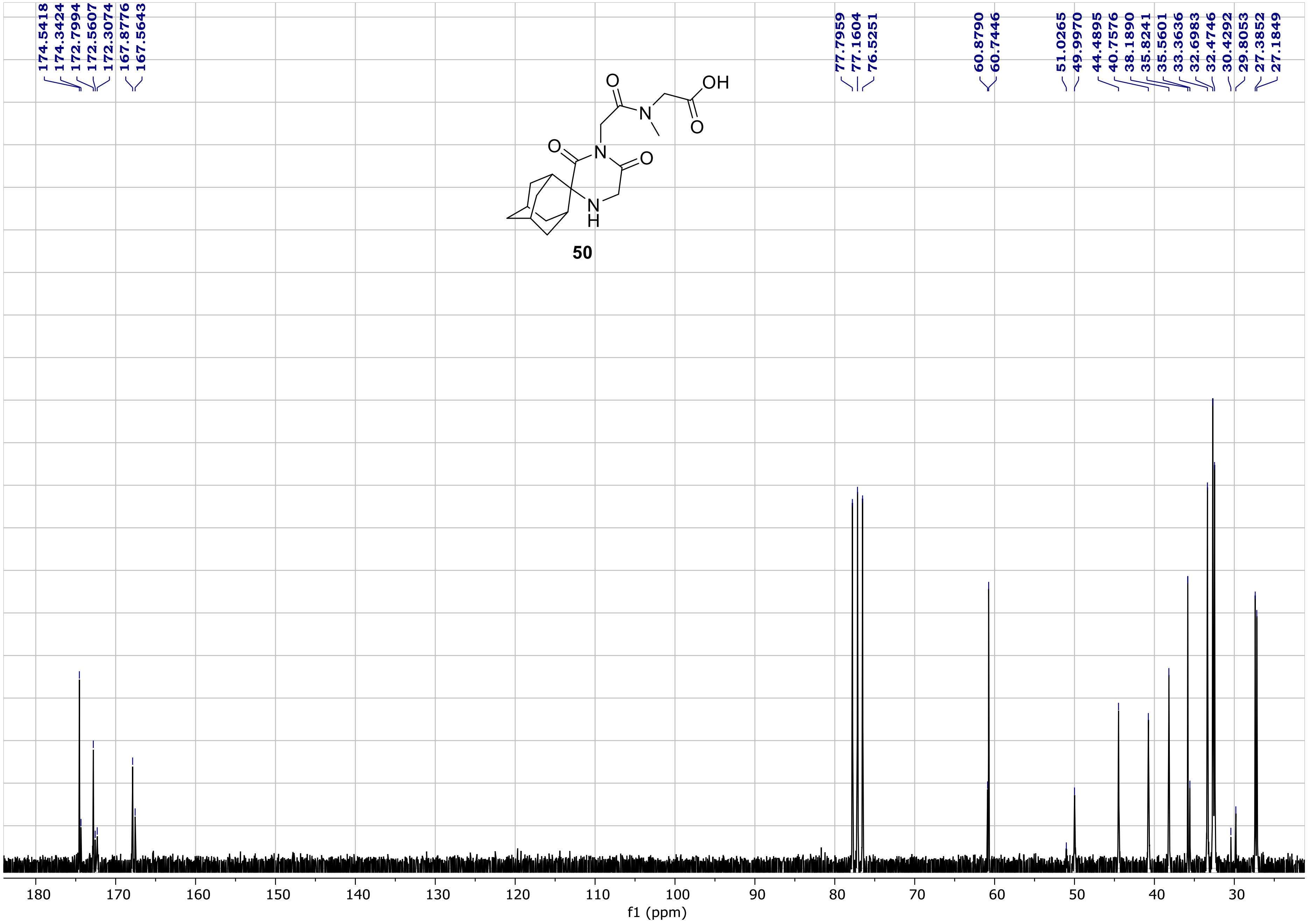

COSY NMR of **50** (400.11 MHz, $CDCl_3$)

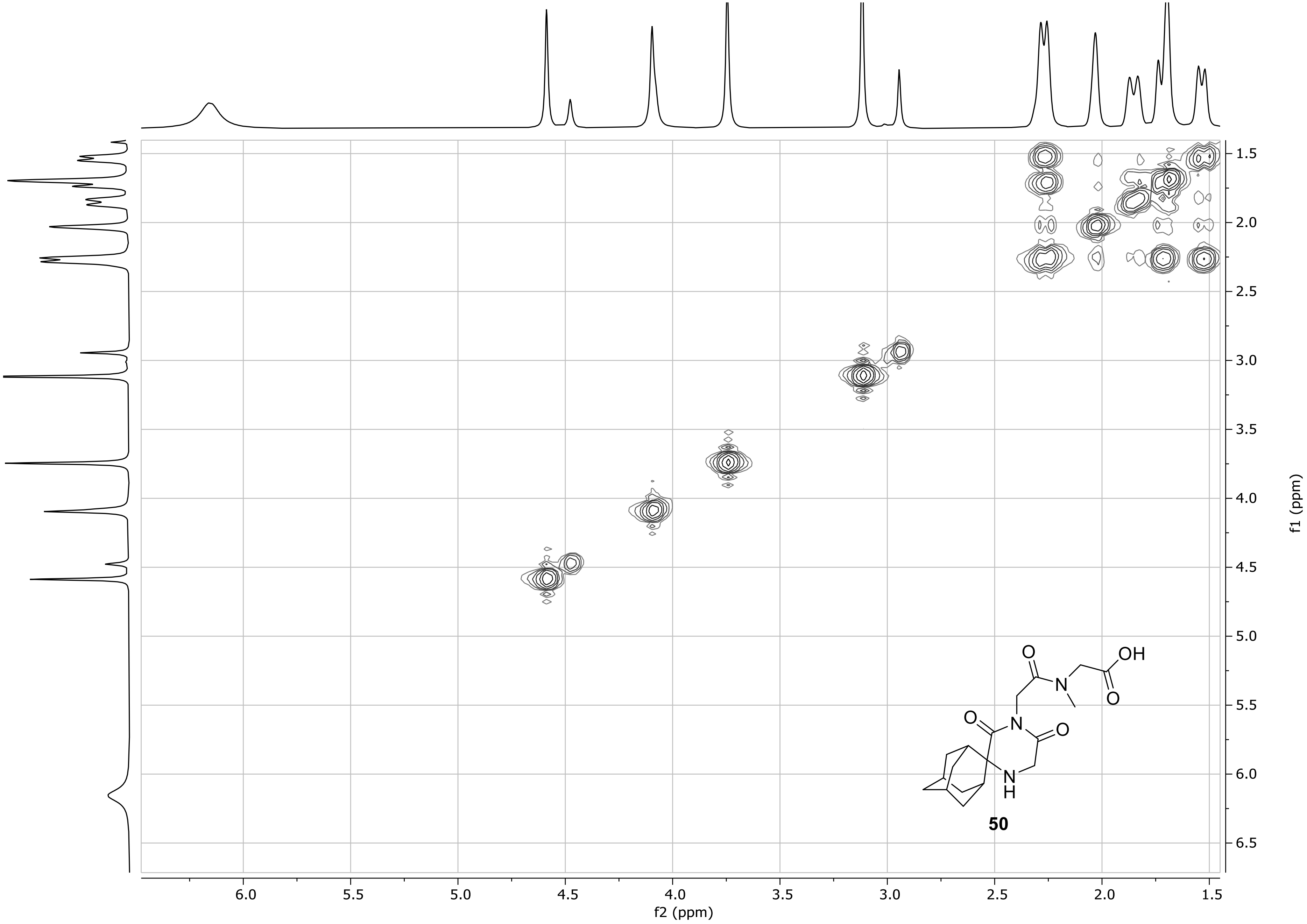

HSQC NMR of **50** (400.11 MHz, $CDCl_3$)

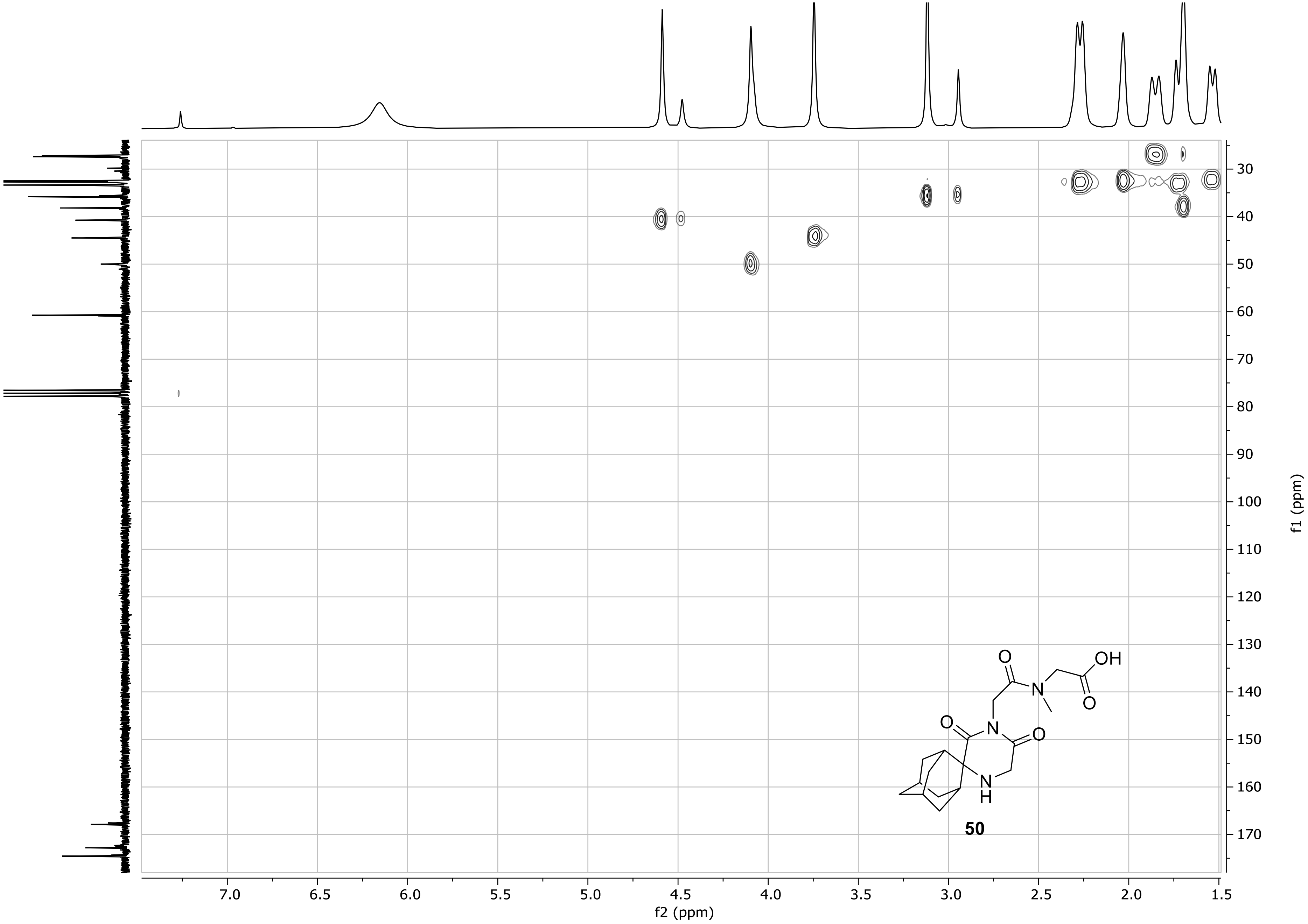

50

DEPT NMR of **50** (50.32 MHz, $CDCl_3$)

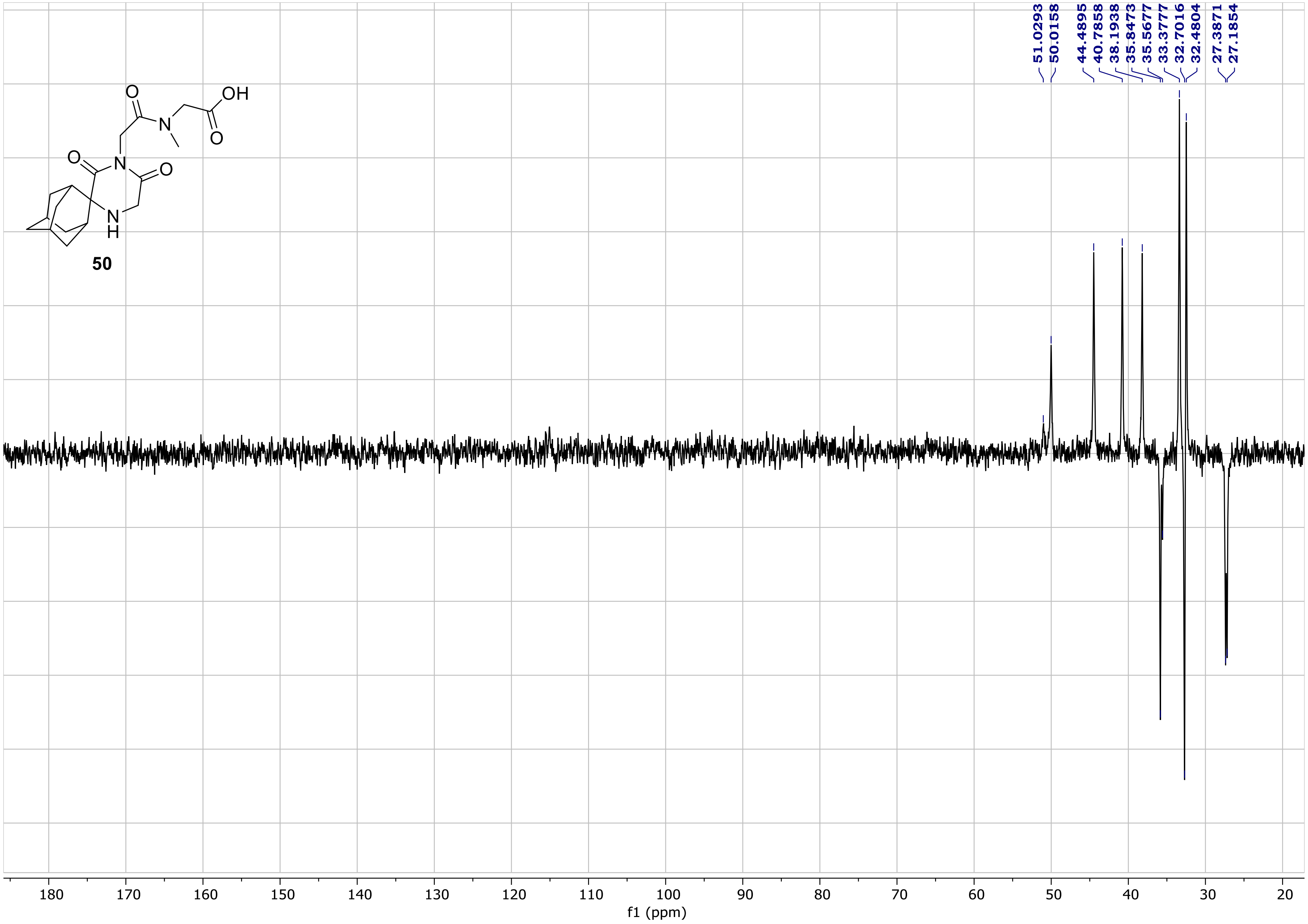

¹H NMR of **51** (600.11 MHz, $CDCl_3$)

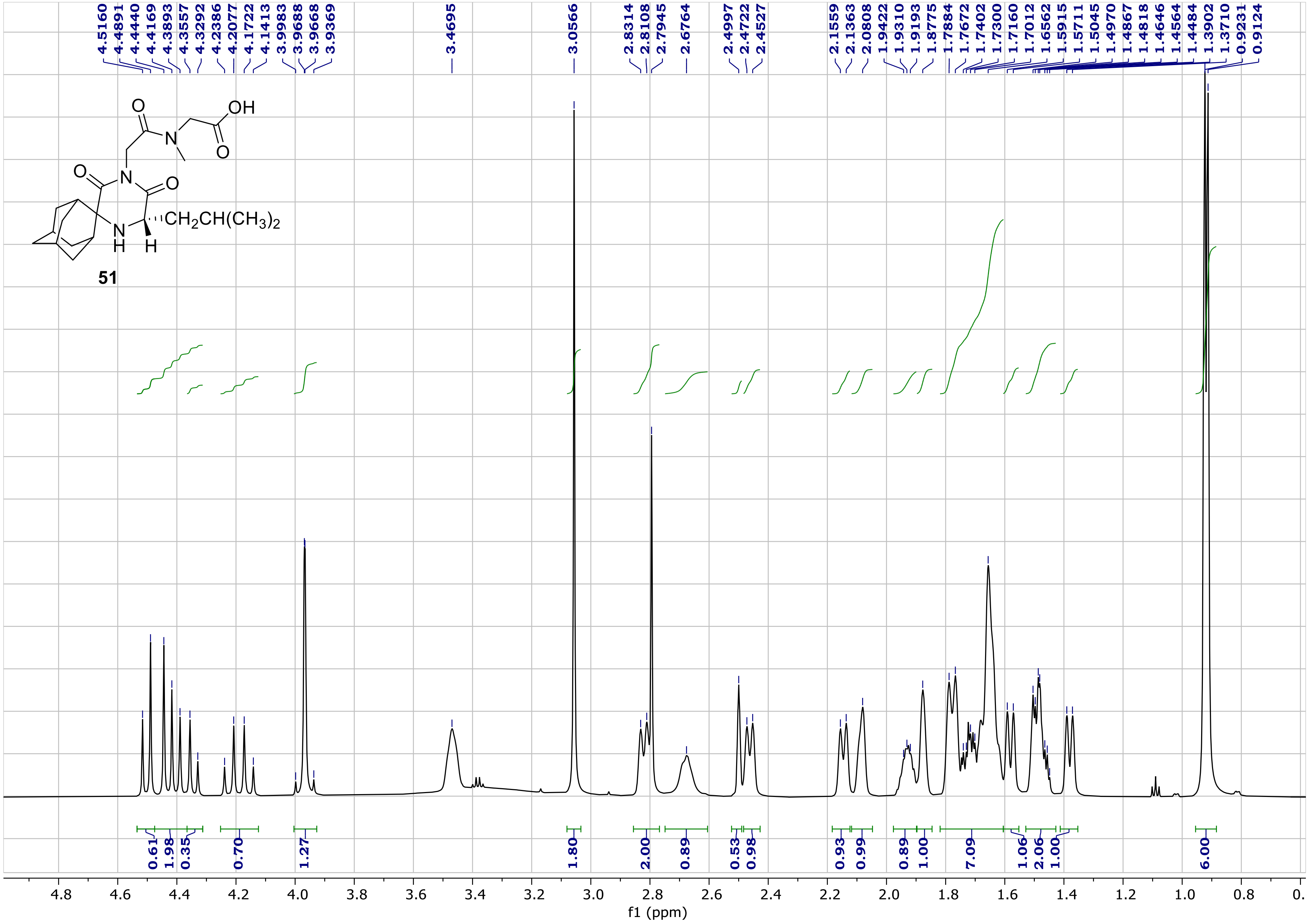

^{13}C NMR of **51** (50.32 MHz, $CDCl_3$)

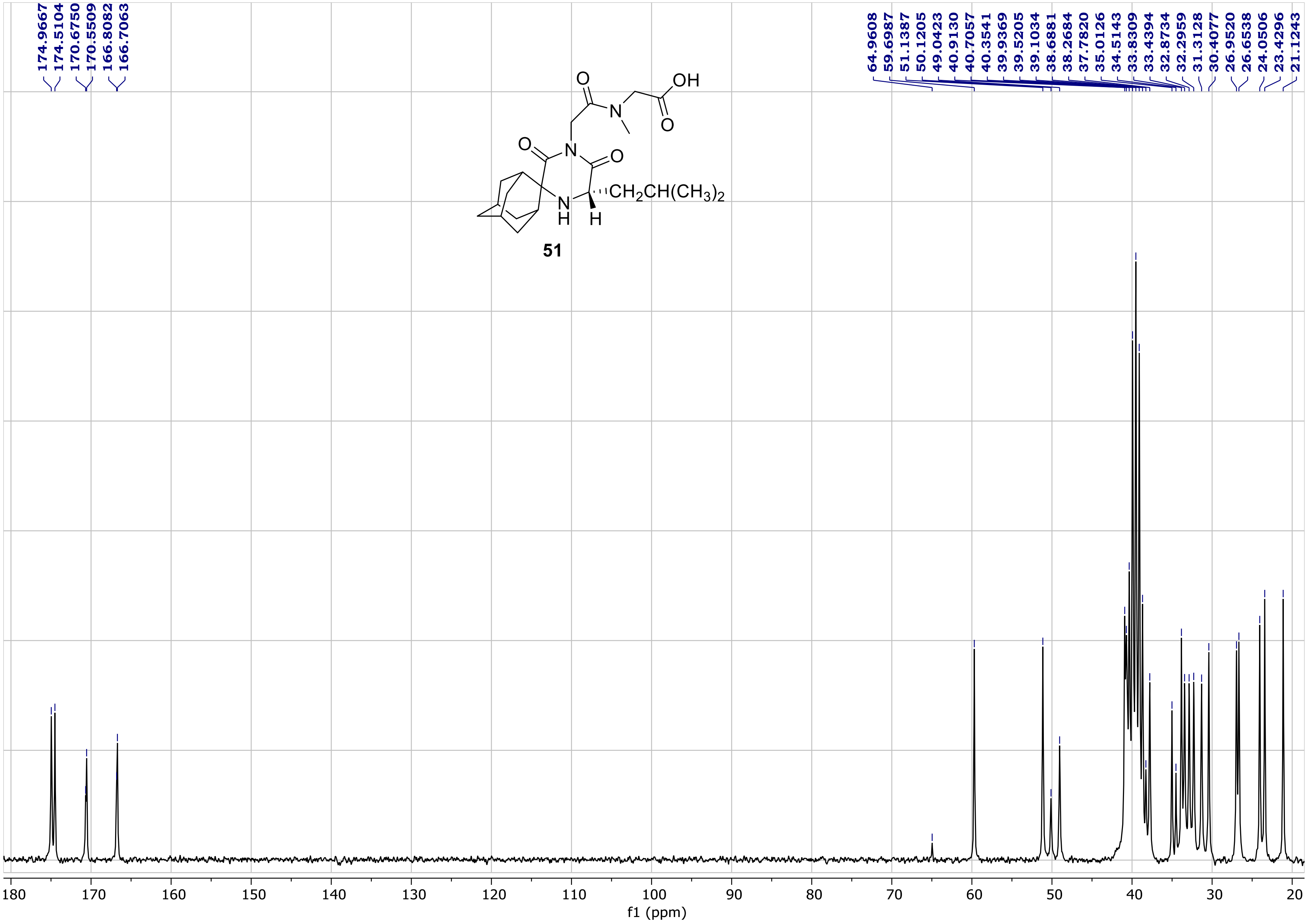

COSY NMR of **51** (600.11 MHz, $CDCl_3$)

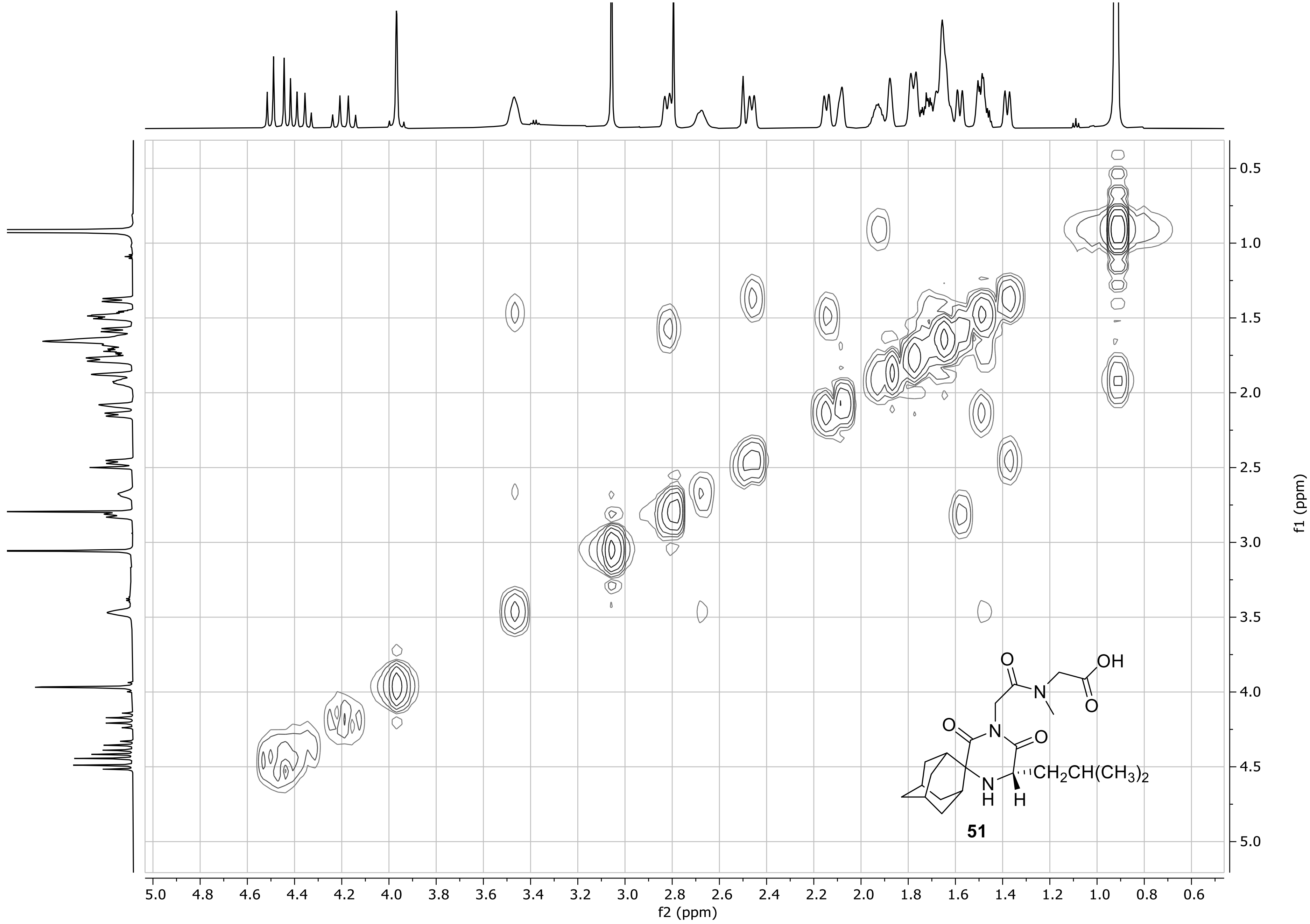

HSQC-DEPT NMR of **51** (600.11 MHz, $CDCl_3$)

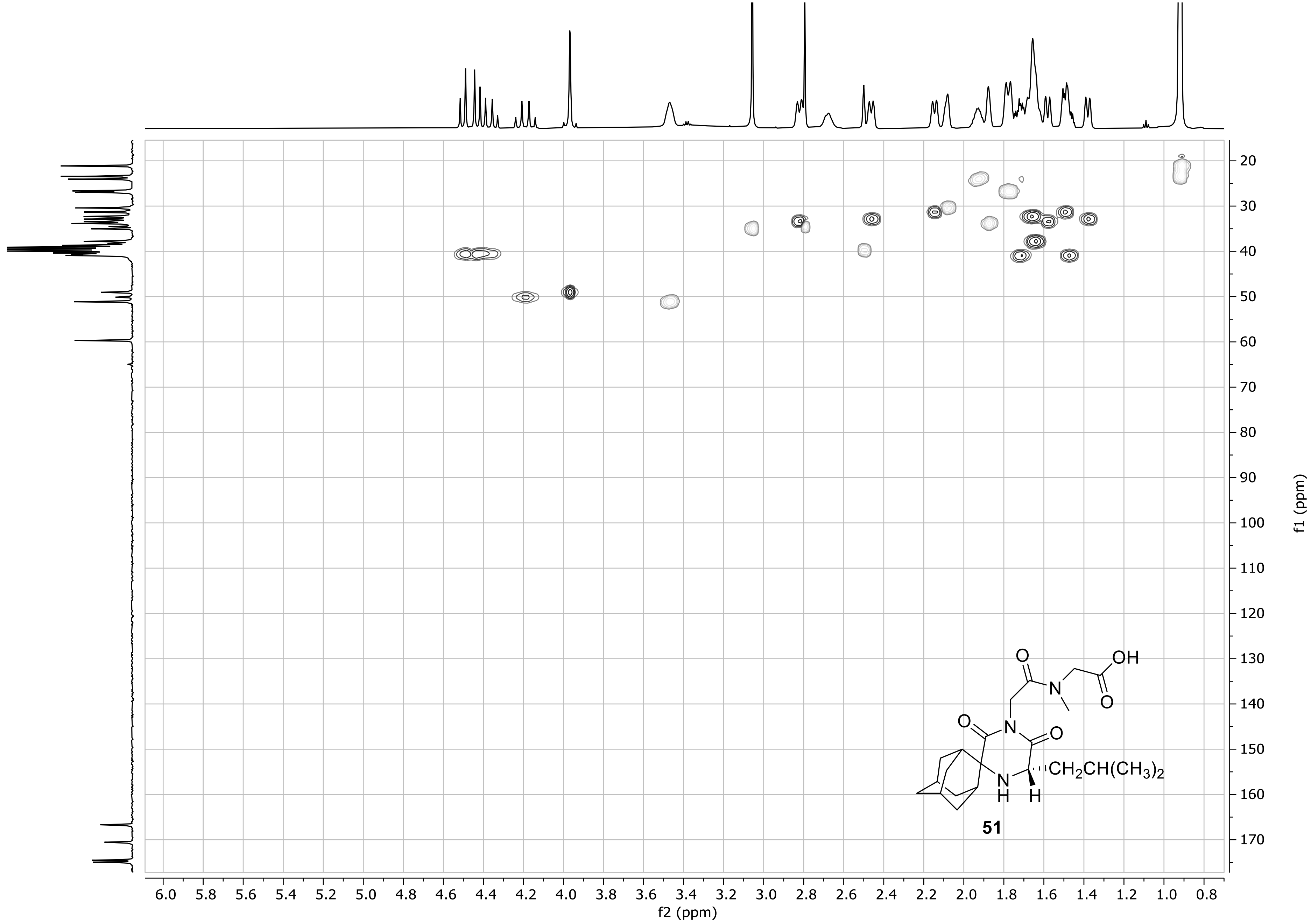

DEPT NMR of **51** (50.32 MHz, $CDCl_3$)

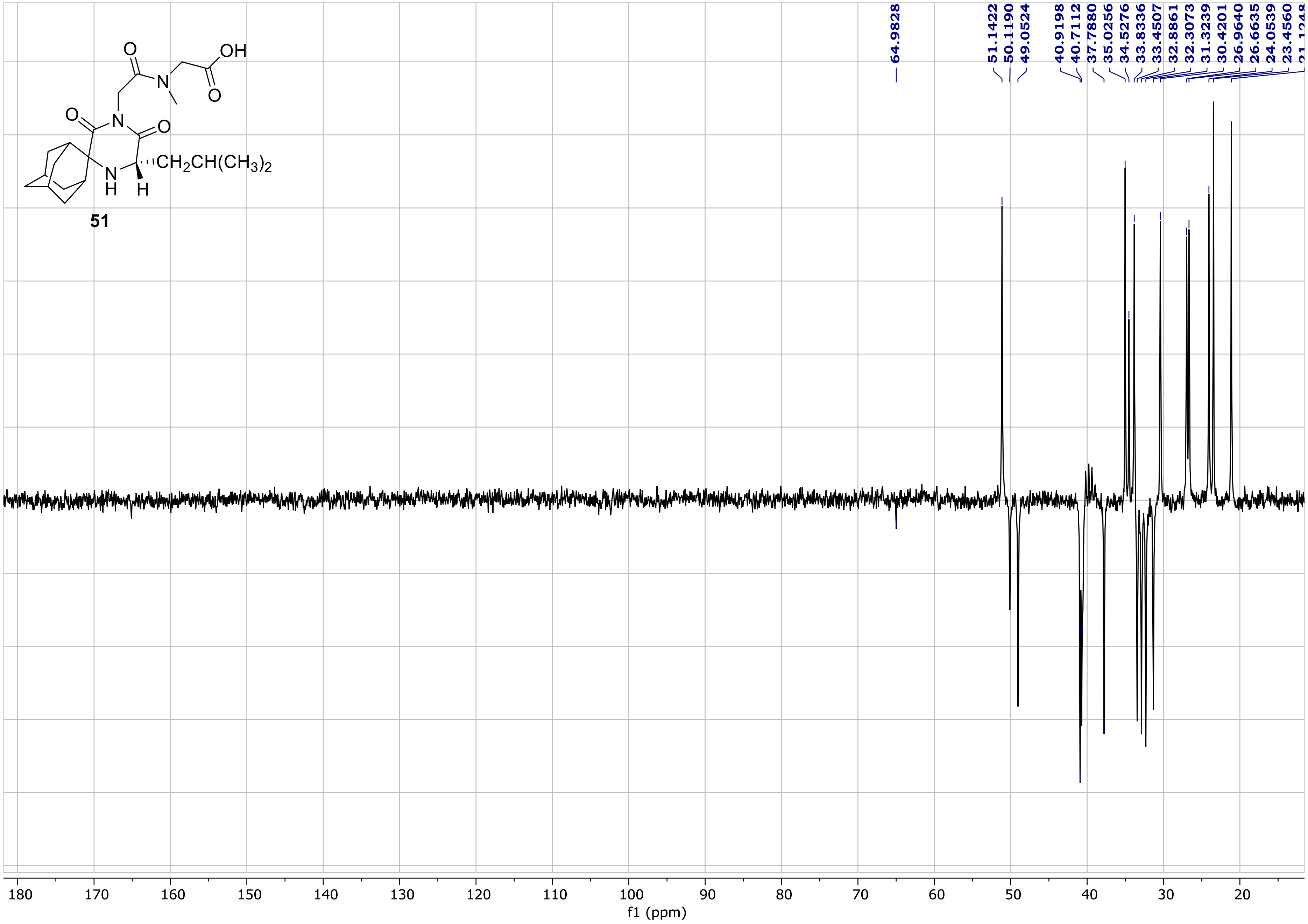

^{1}H NMR of **52** (600.11 MHz, $CDCl_3$)

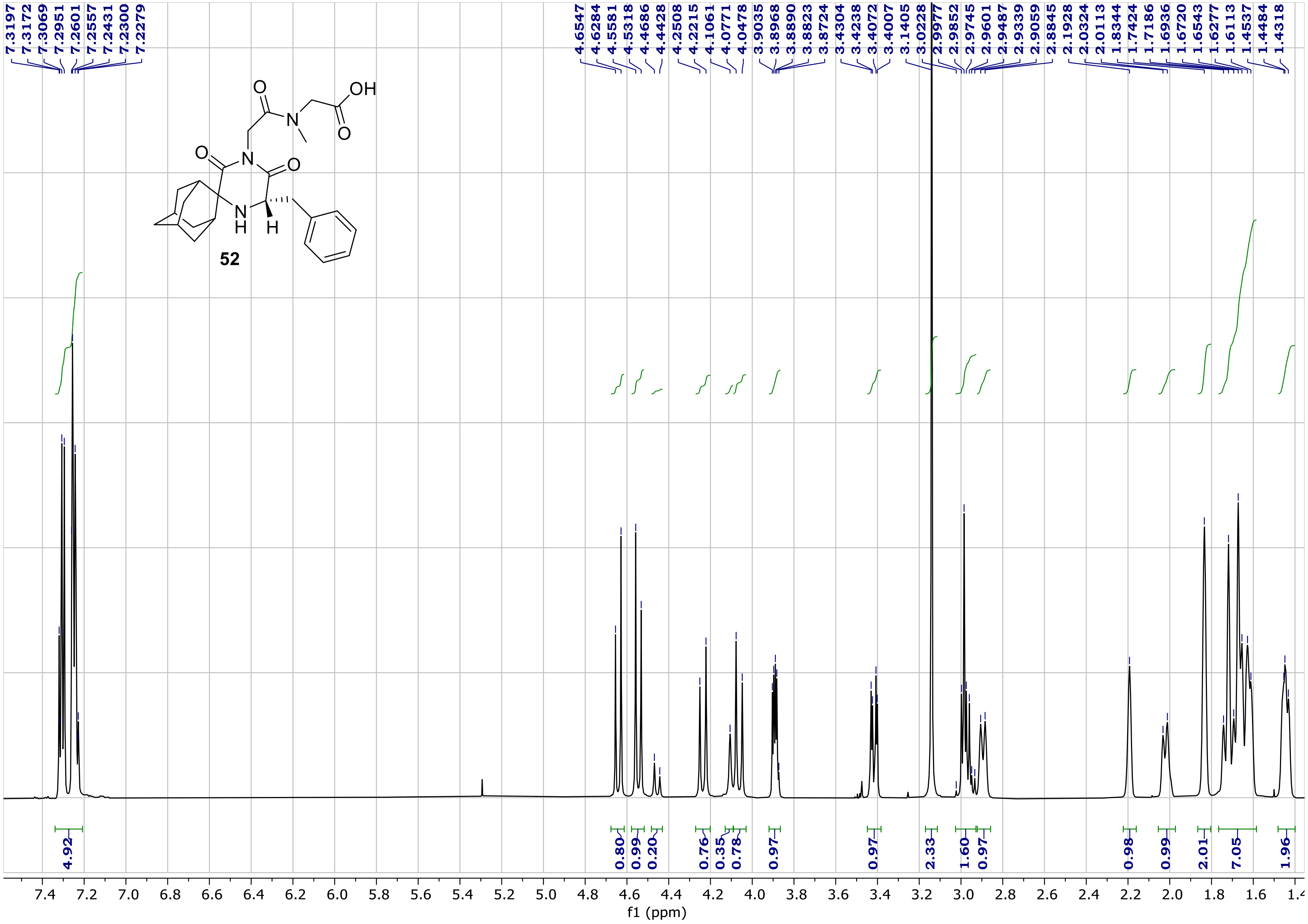

^{13}C NMR of **52** (150.9 MHz, $CDCl_3$)

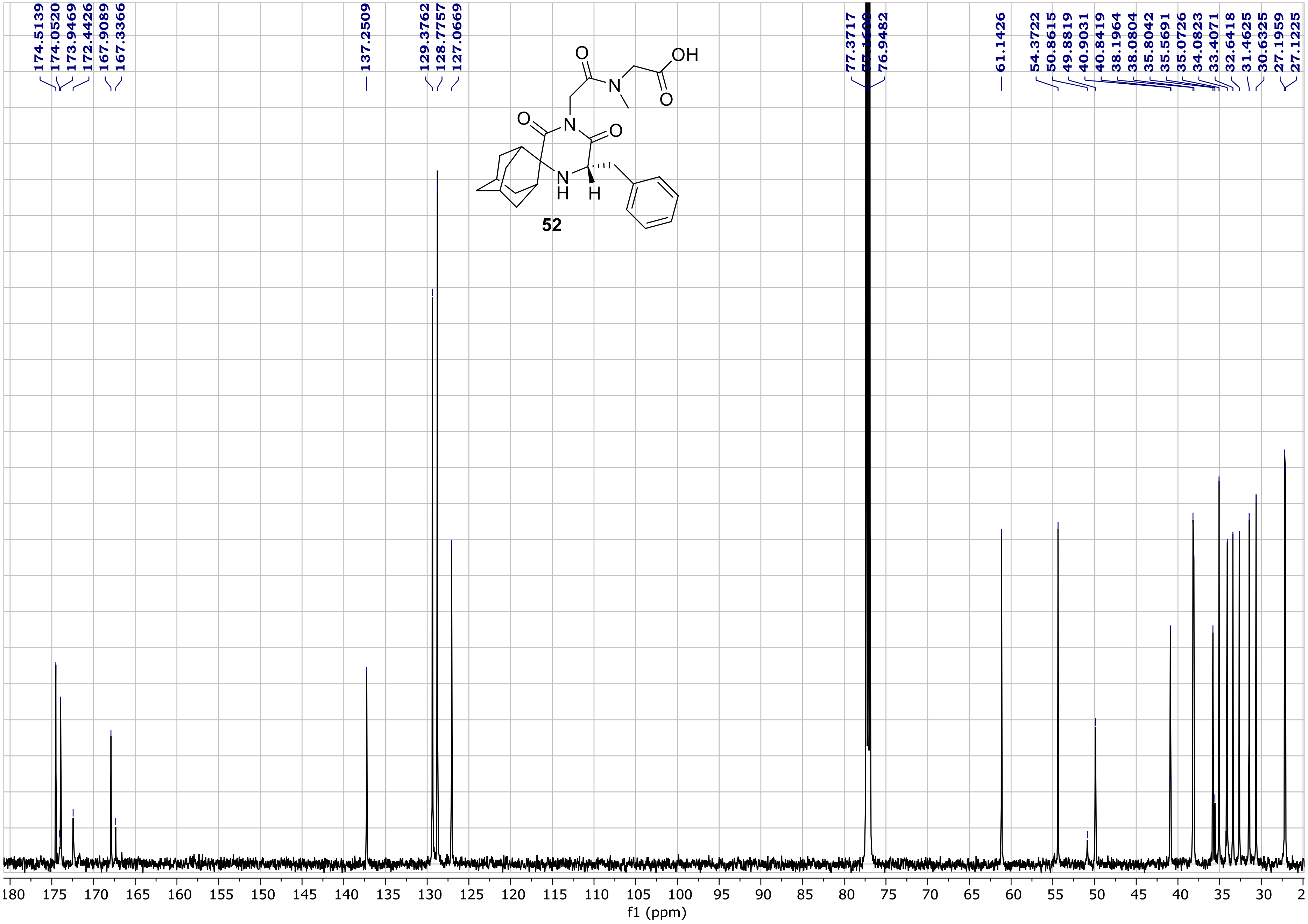

COSY NMR of **52** (600.11 MHz, $CDCl_3$)

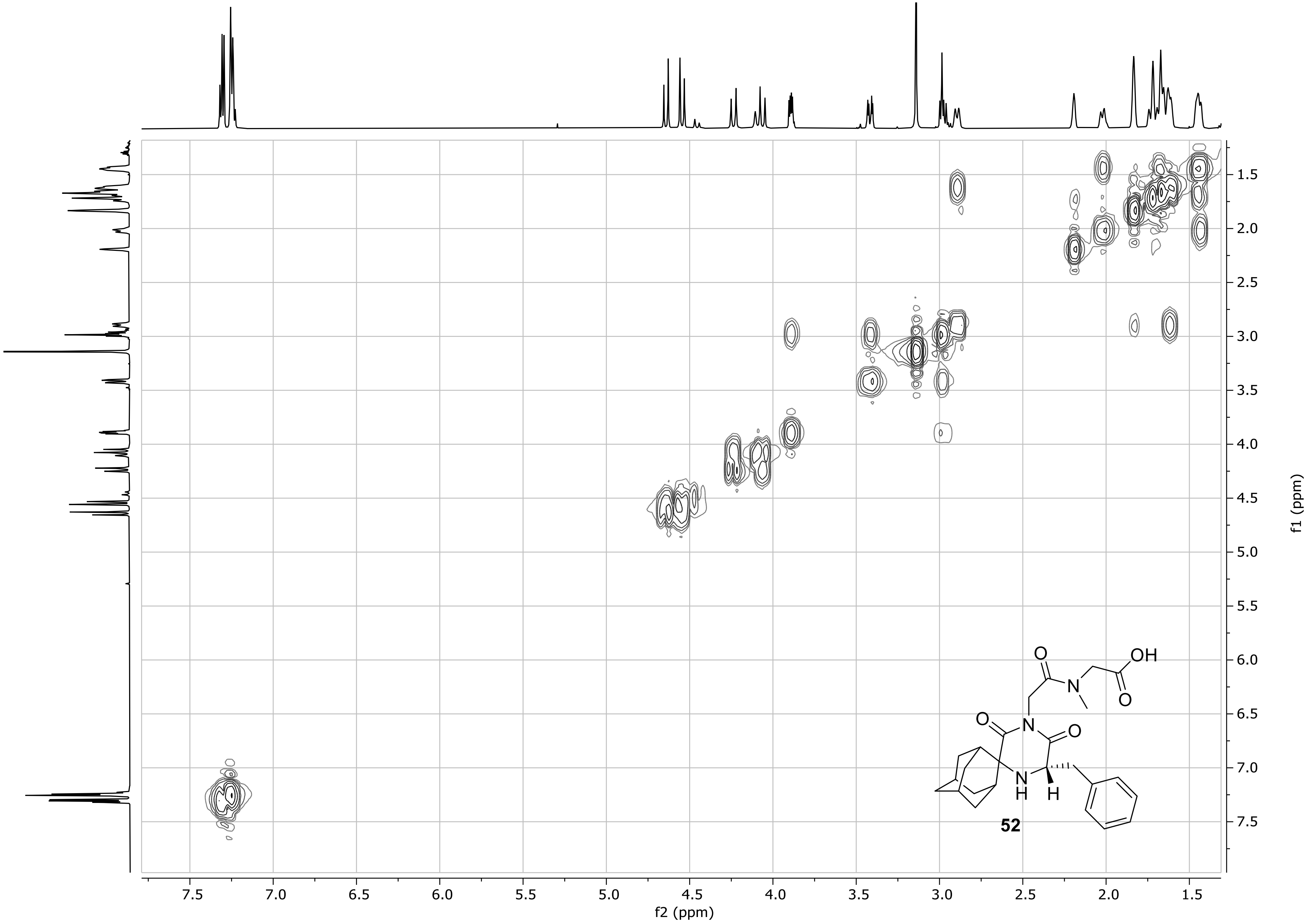

HSQC-DEPT NMR of **52** (600.11 MHz, $CDCl_3$)

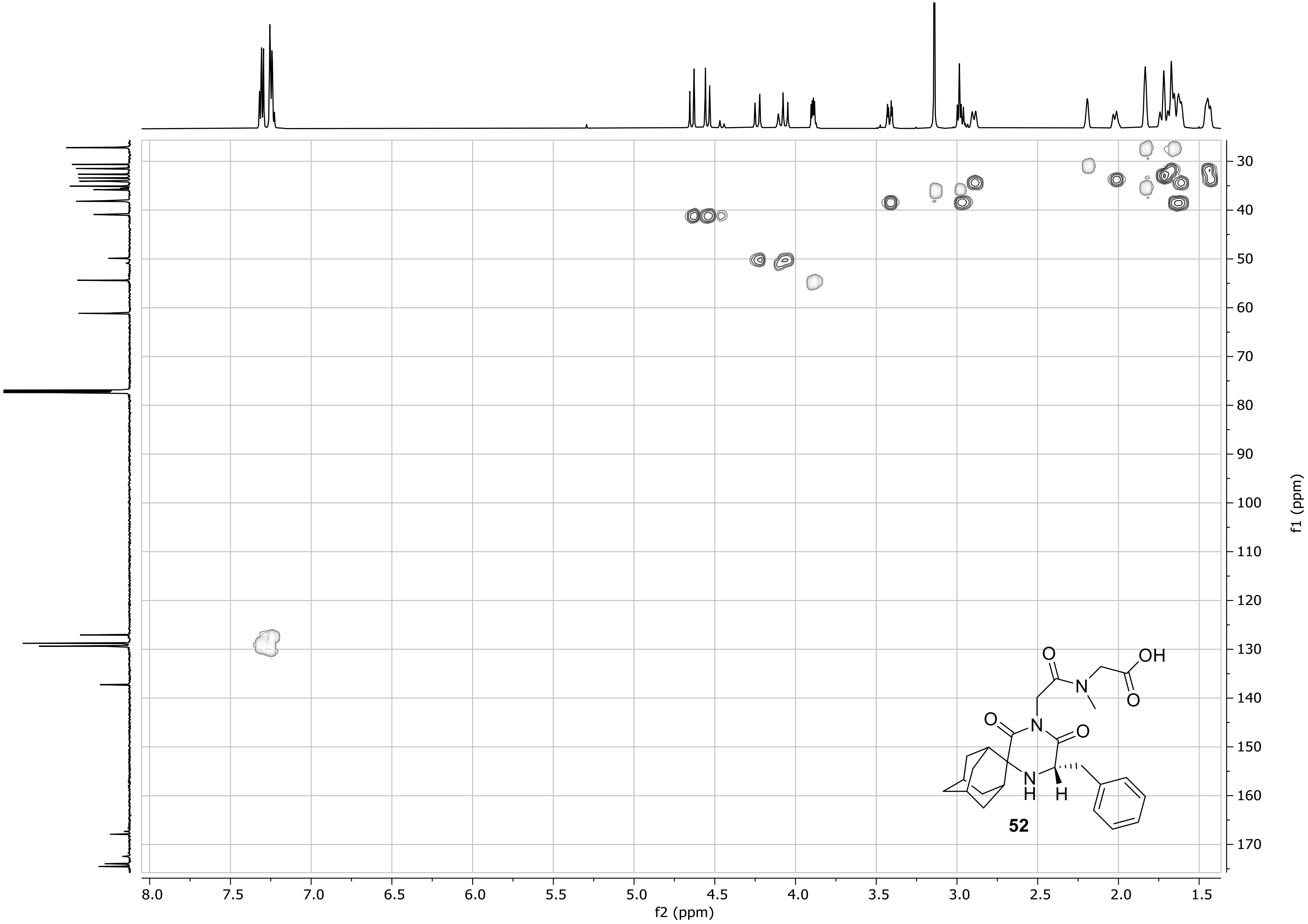

^{1}H NMR of **53** (400.11 MHz, DMSO-d_6)

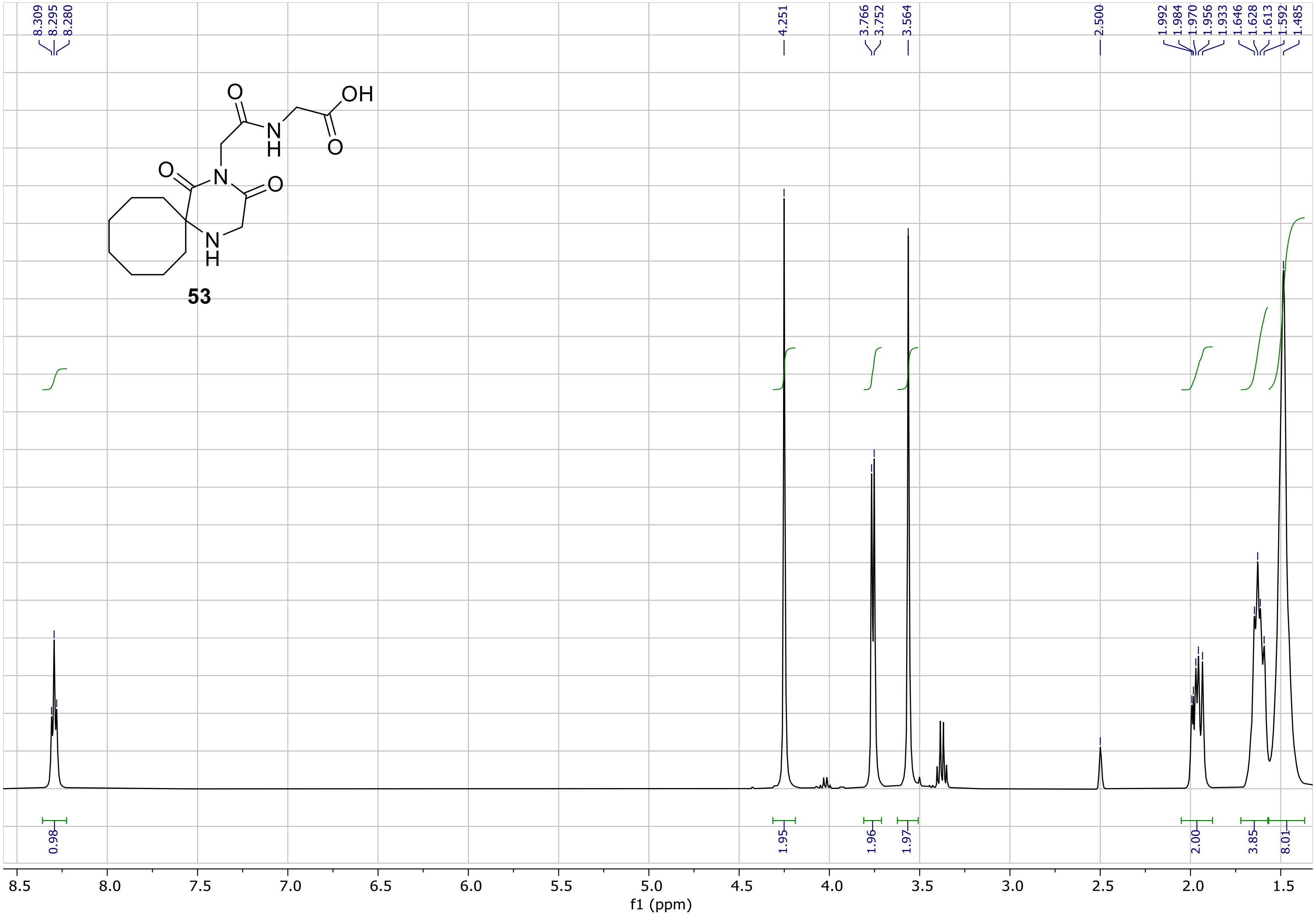

^{13}C NMR of **53** (50.32 MHz, DMSO-d_6)

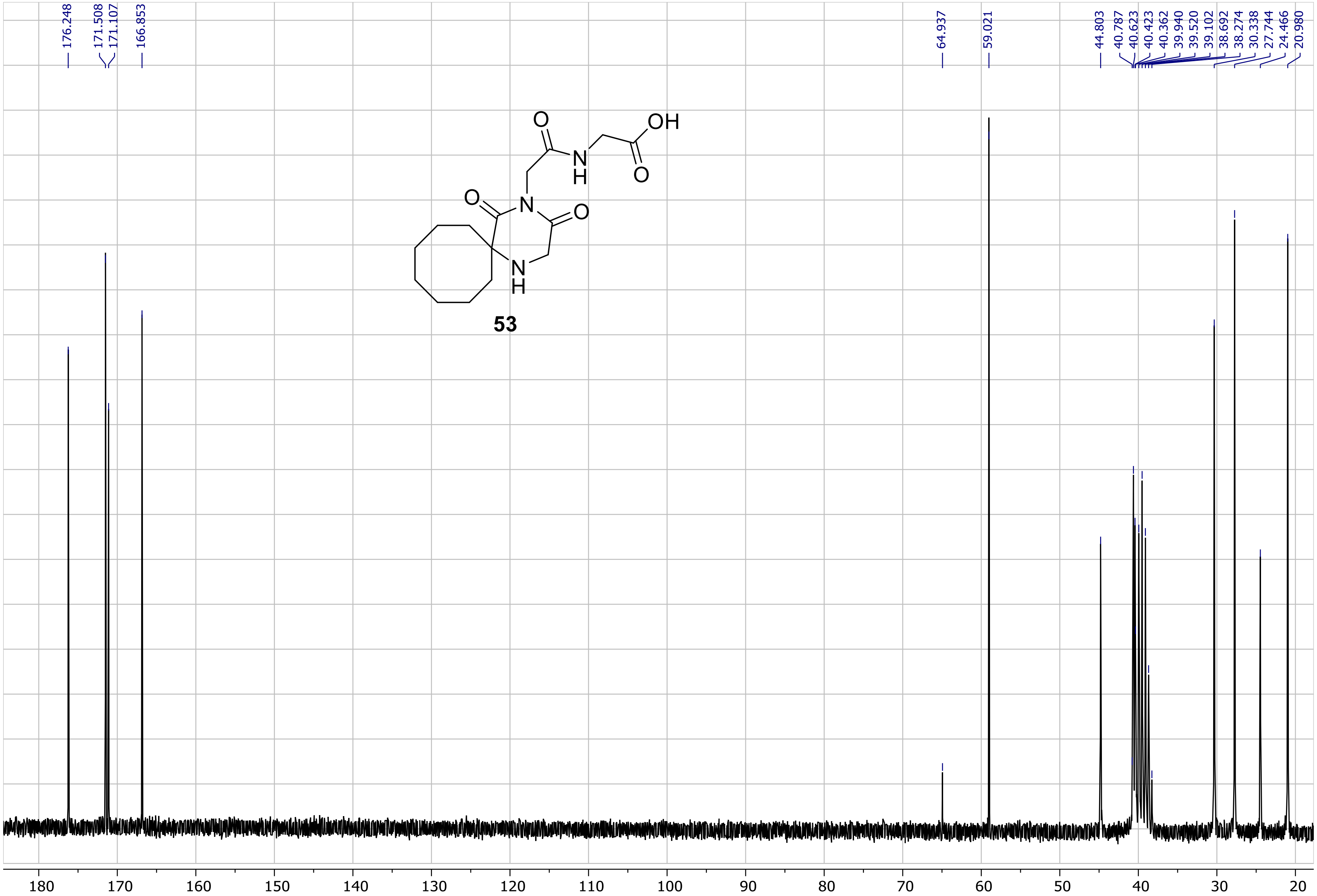

COSY NMR of **53** (400.11 MHz, DMSO-d_6)

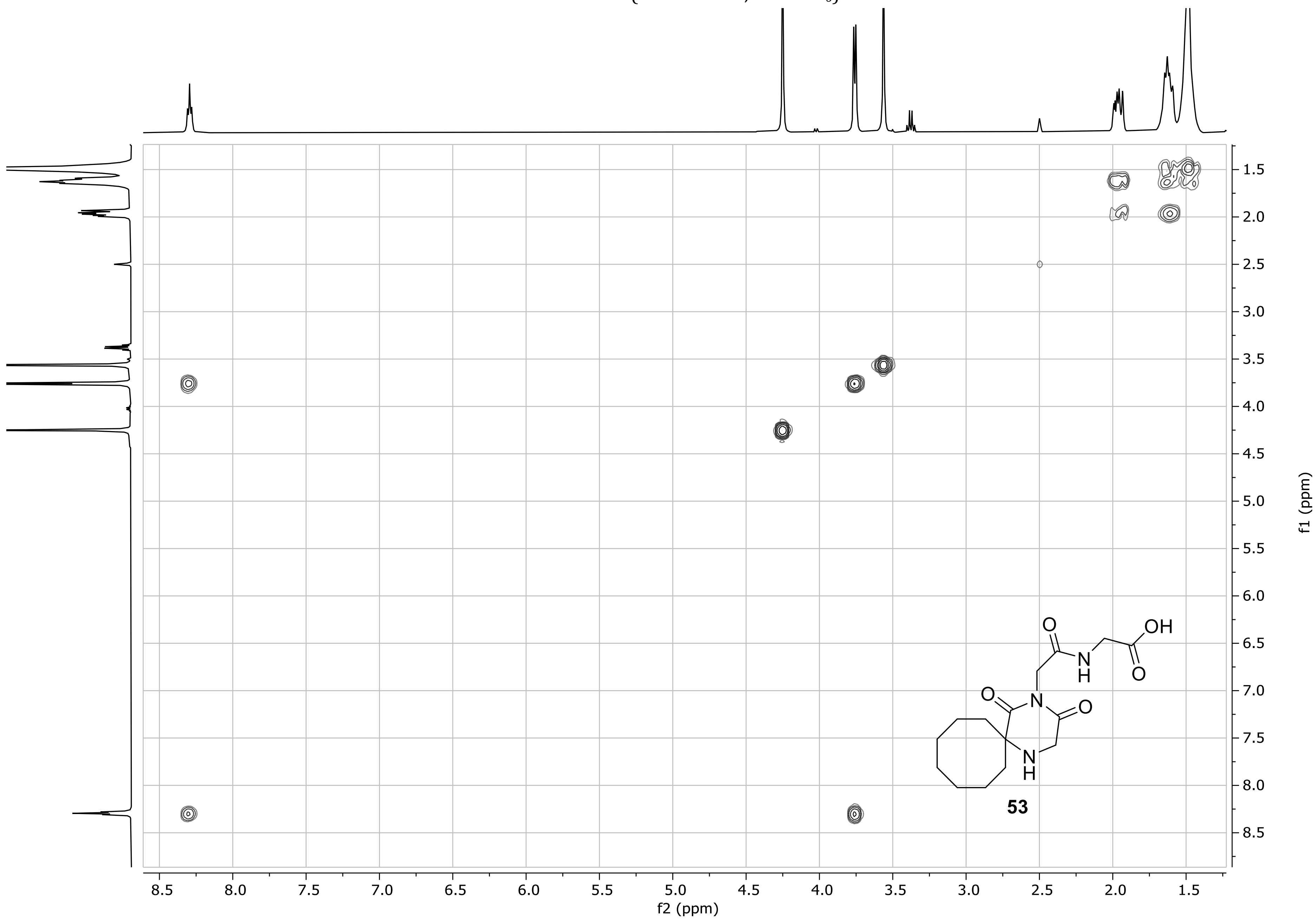

HSQC NMR of **53** (400.11 MHz, DMSO-d_6)

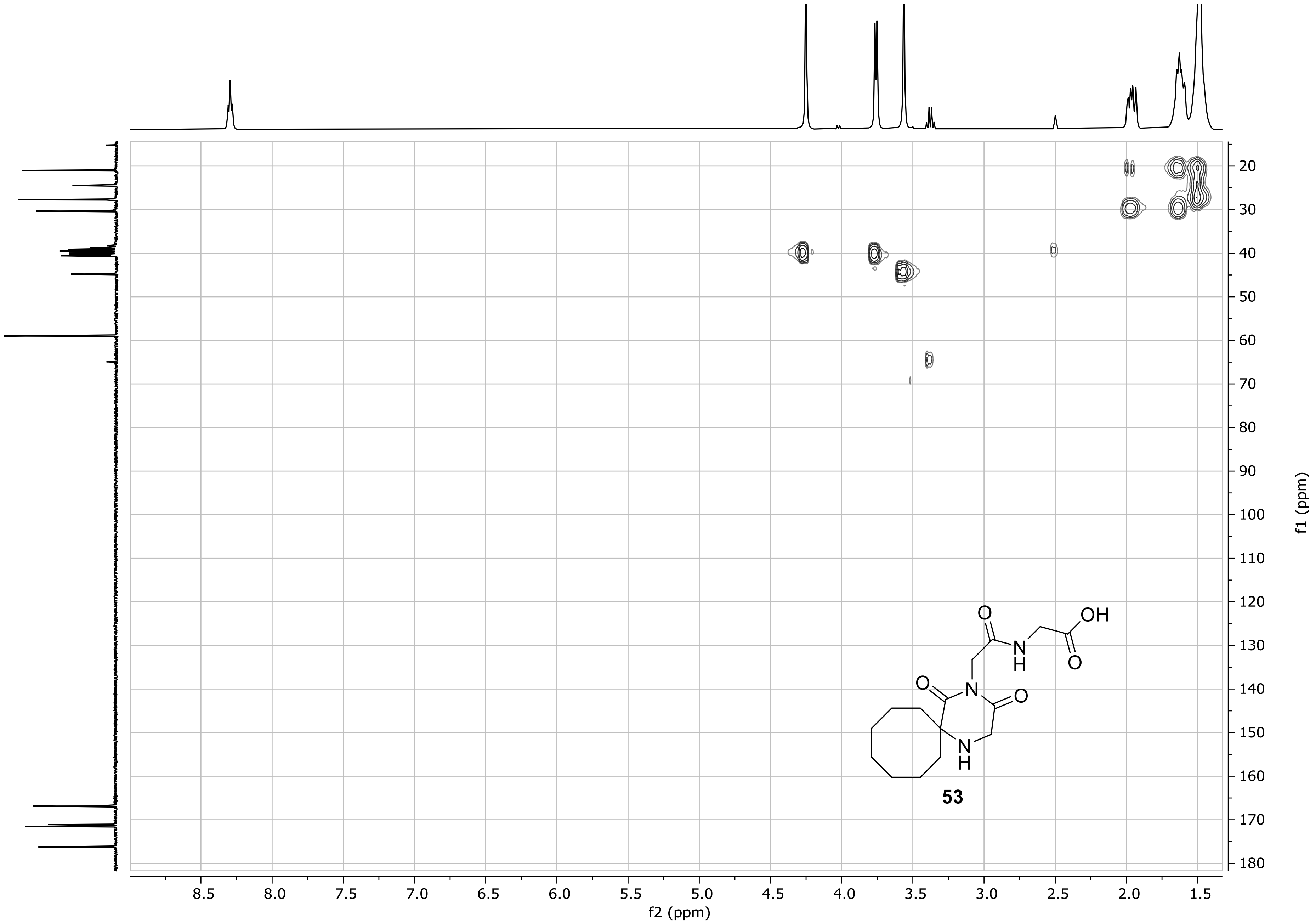

DEPT NMR of **53** (50.32 MHz, DMSO-d_6)

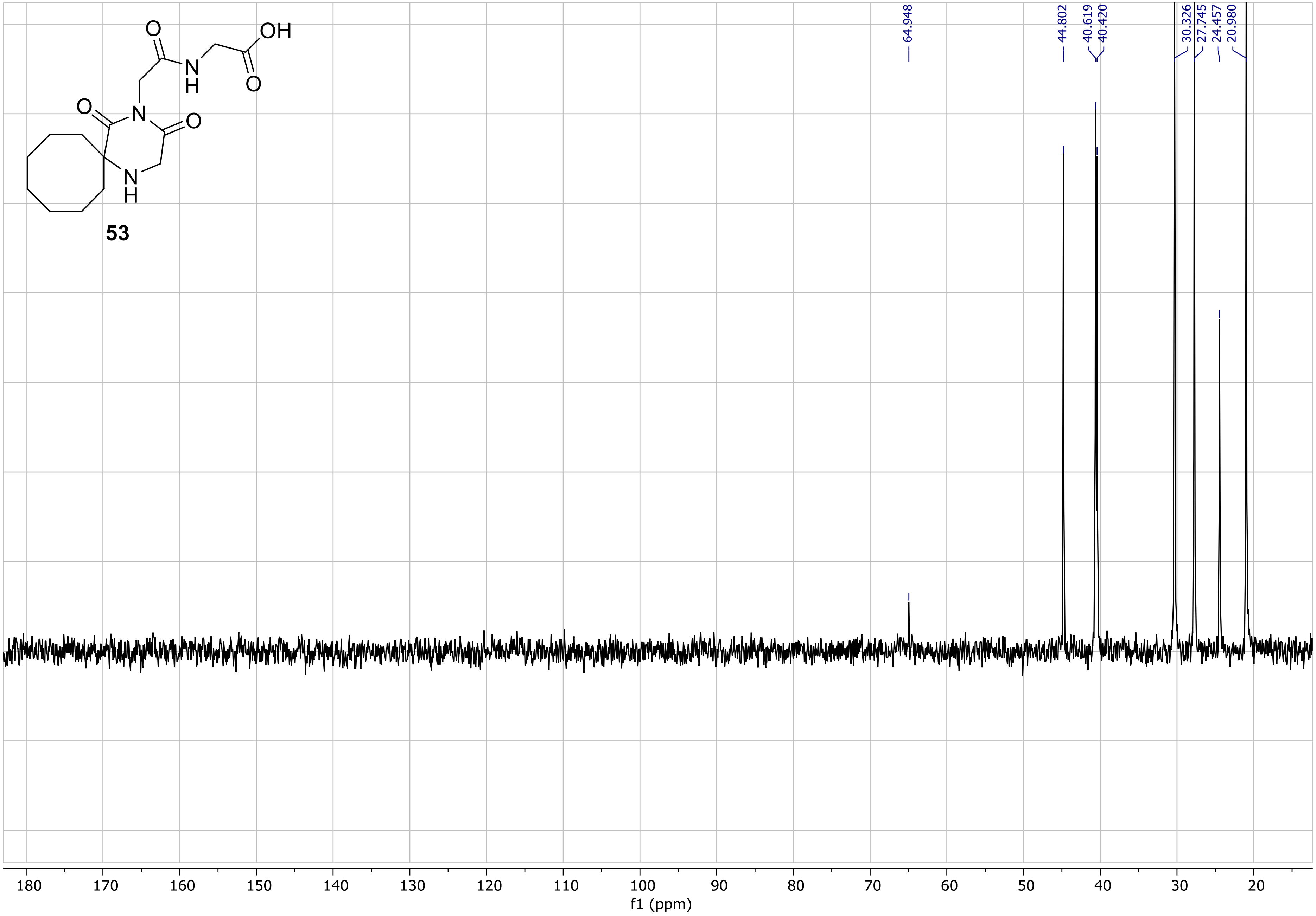

^{1}H NMR of **54** (600.11 MHz, $CDCl_3$)

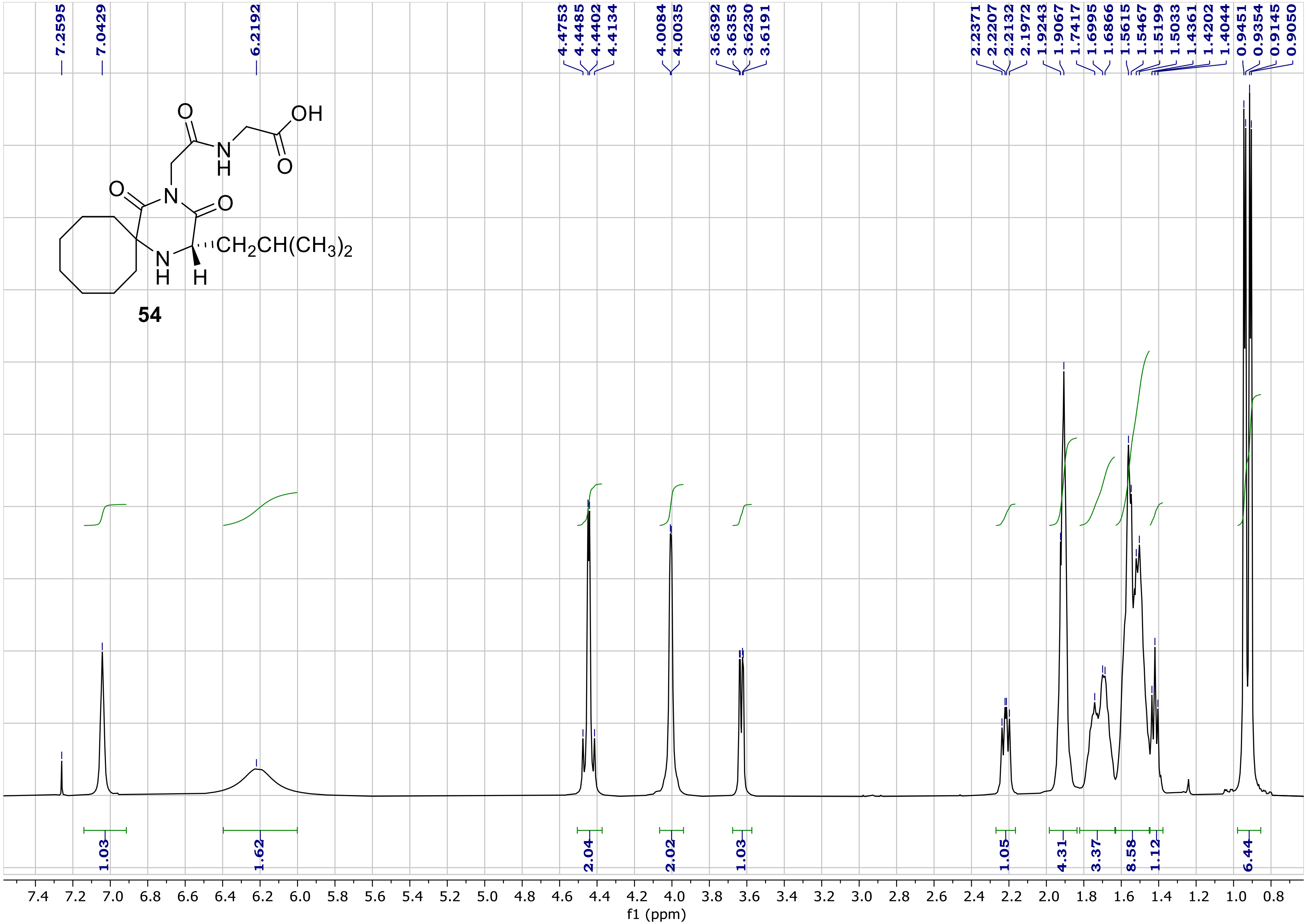

^{13}C NMR of **54** (50.32 MHz, $CDCl_3$)

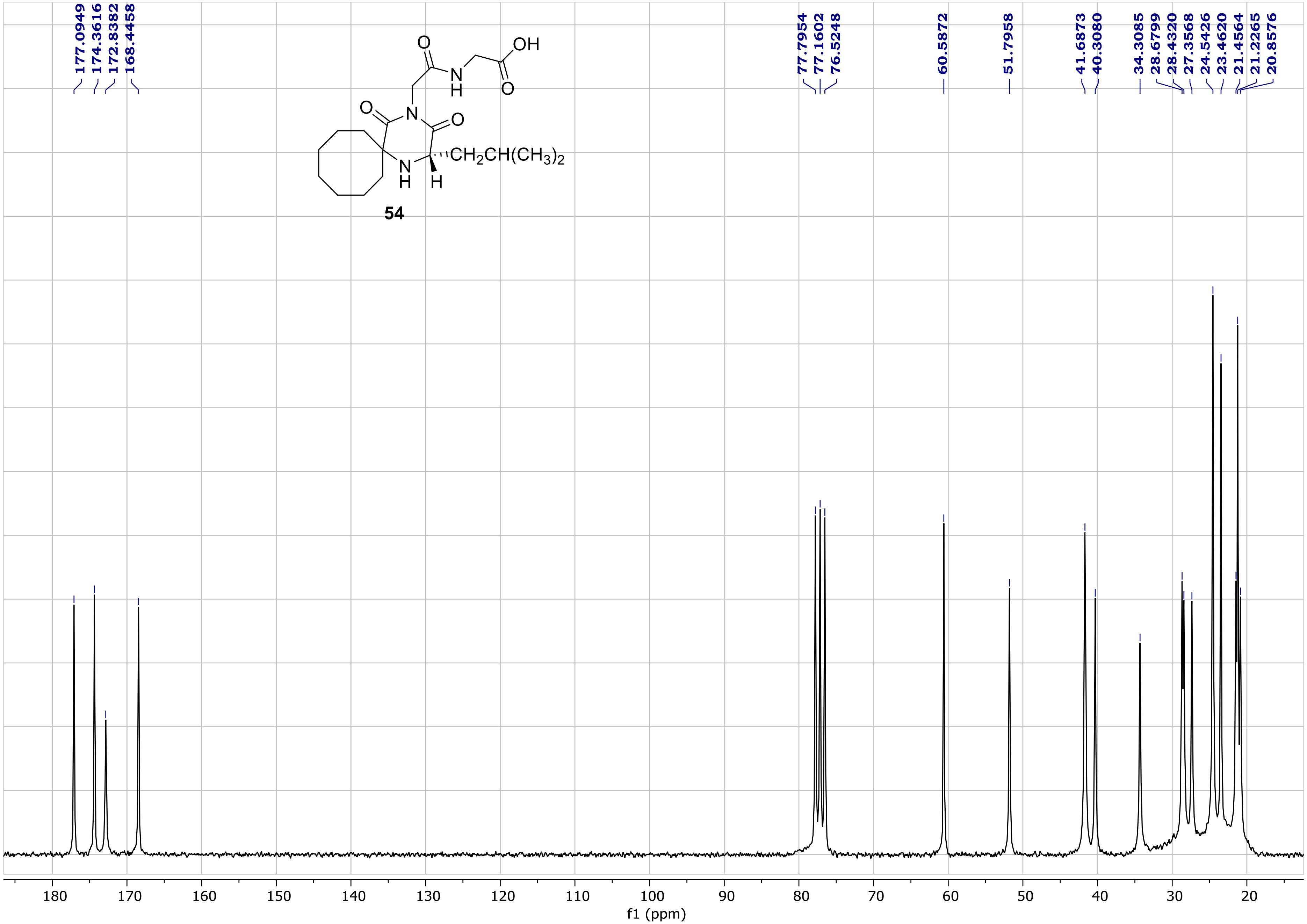

COSY NMR of **54** (600.11 MHz, $CDCl_3$)

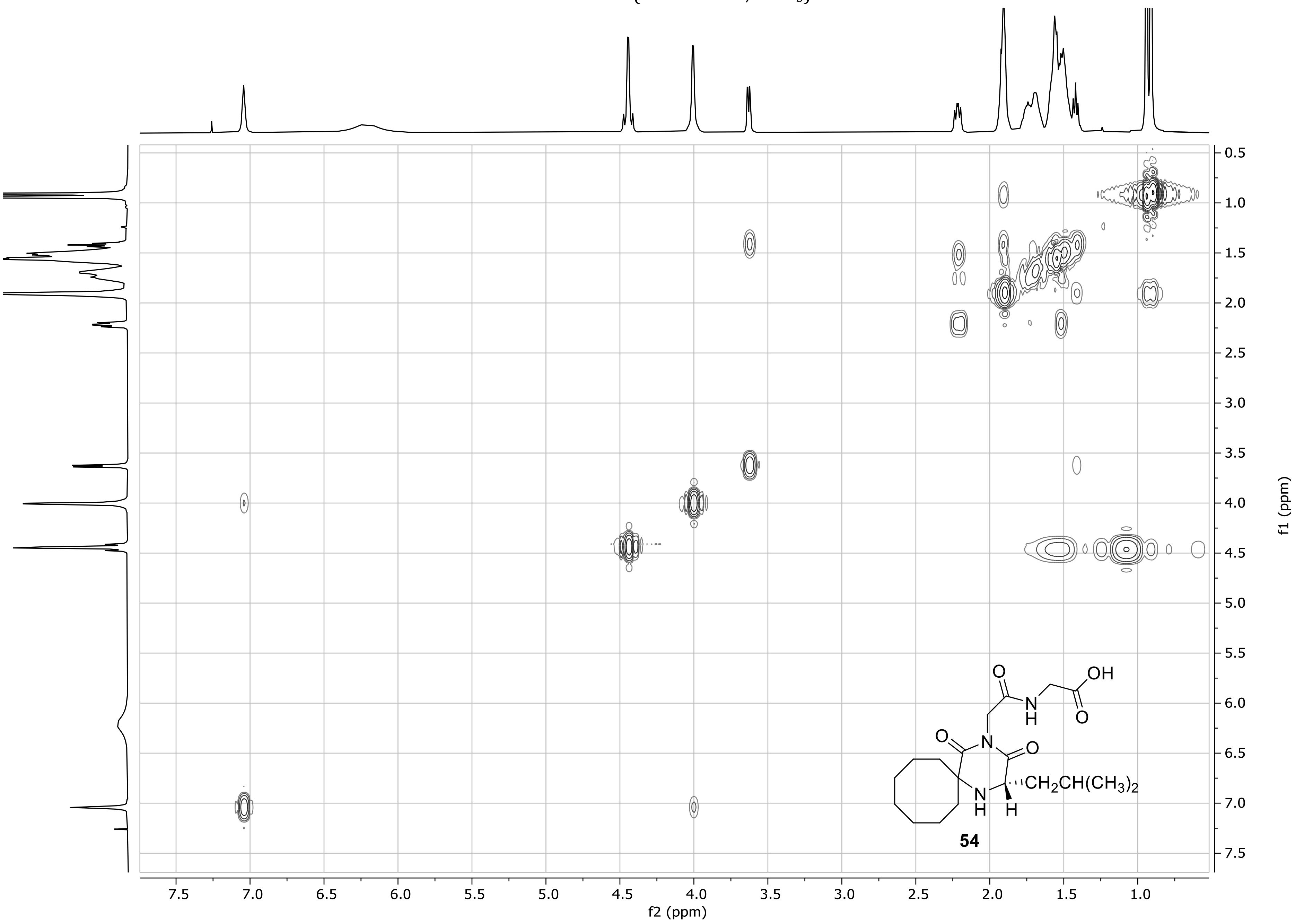

HSQC-DEPT NMR of **54** (600.11 MHz, $CDCl_3$)

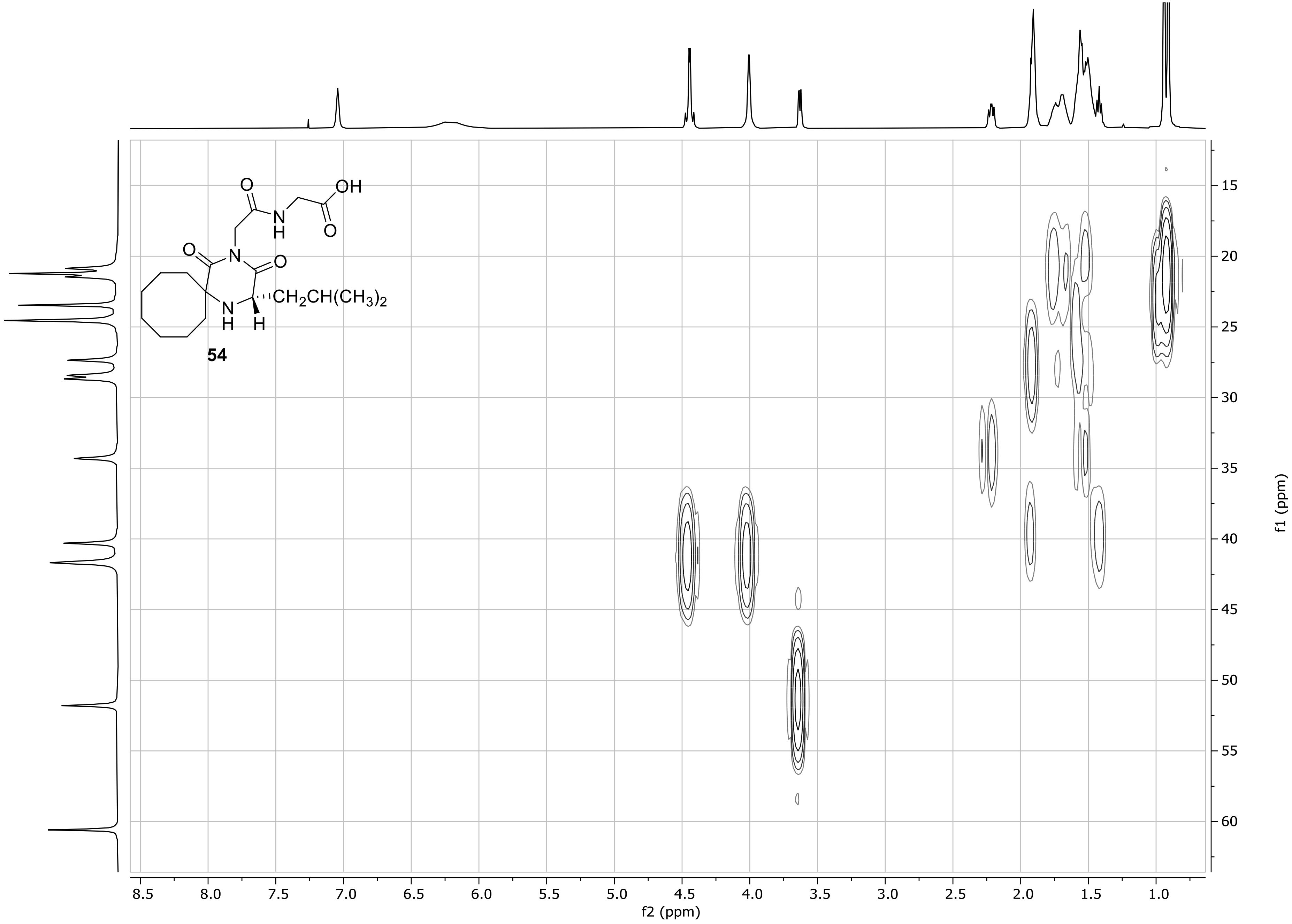

DEPT NMR of **54** (50.32 MHz, $CDCl_3$)

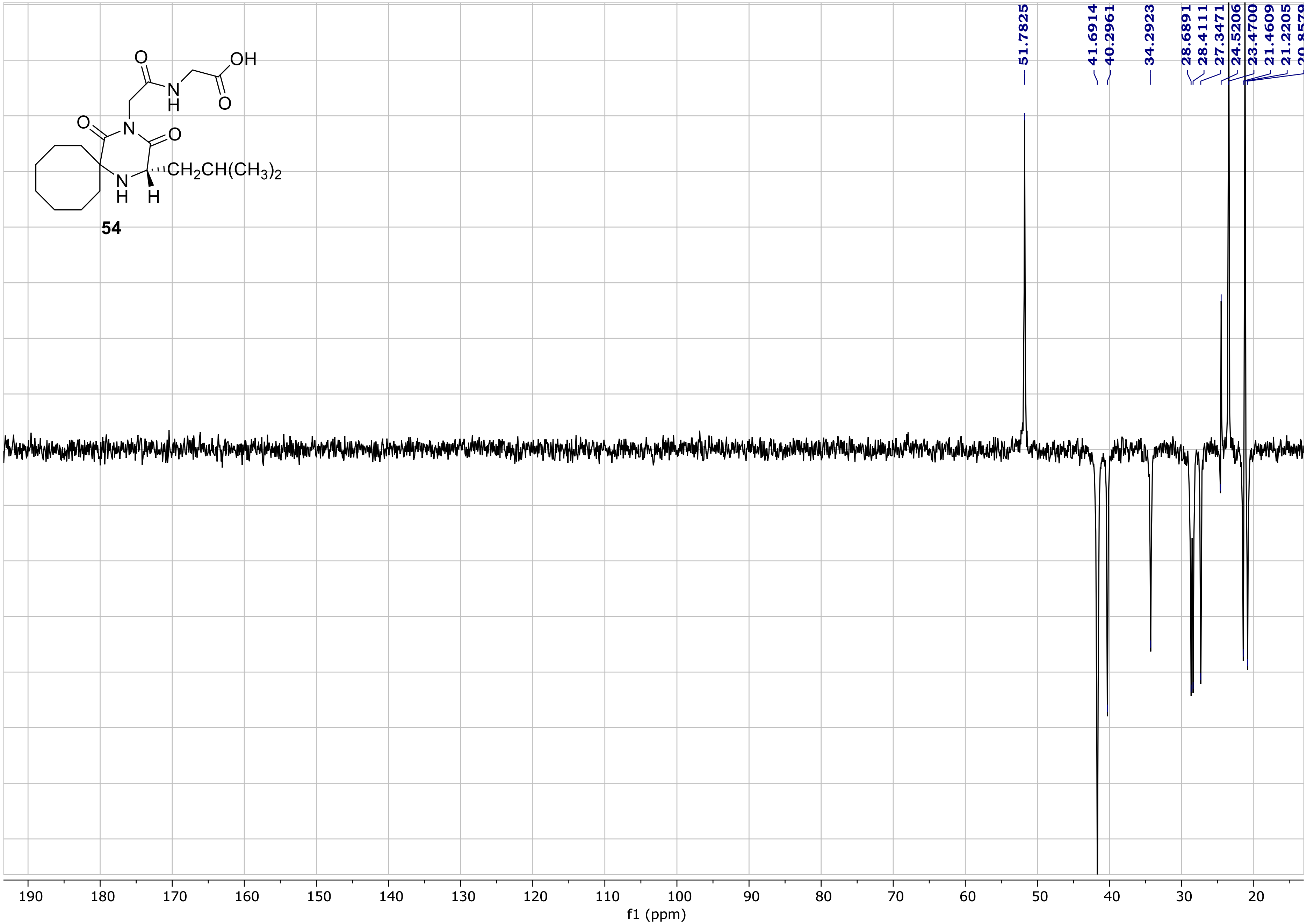

^{1}H NMR of **55** (400.11 MHz, $CDCl_3$)

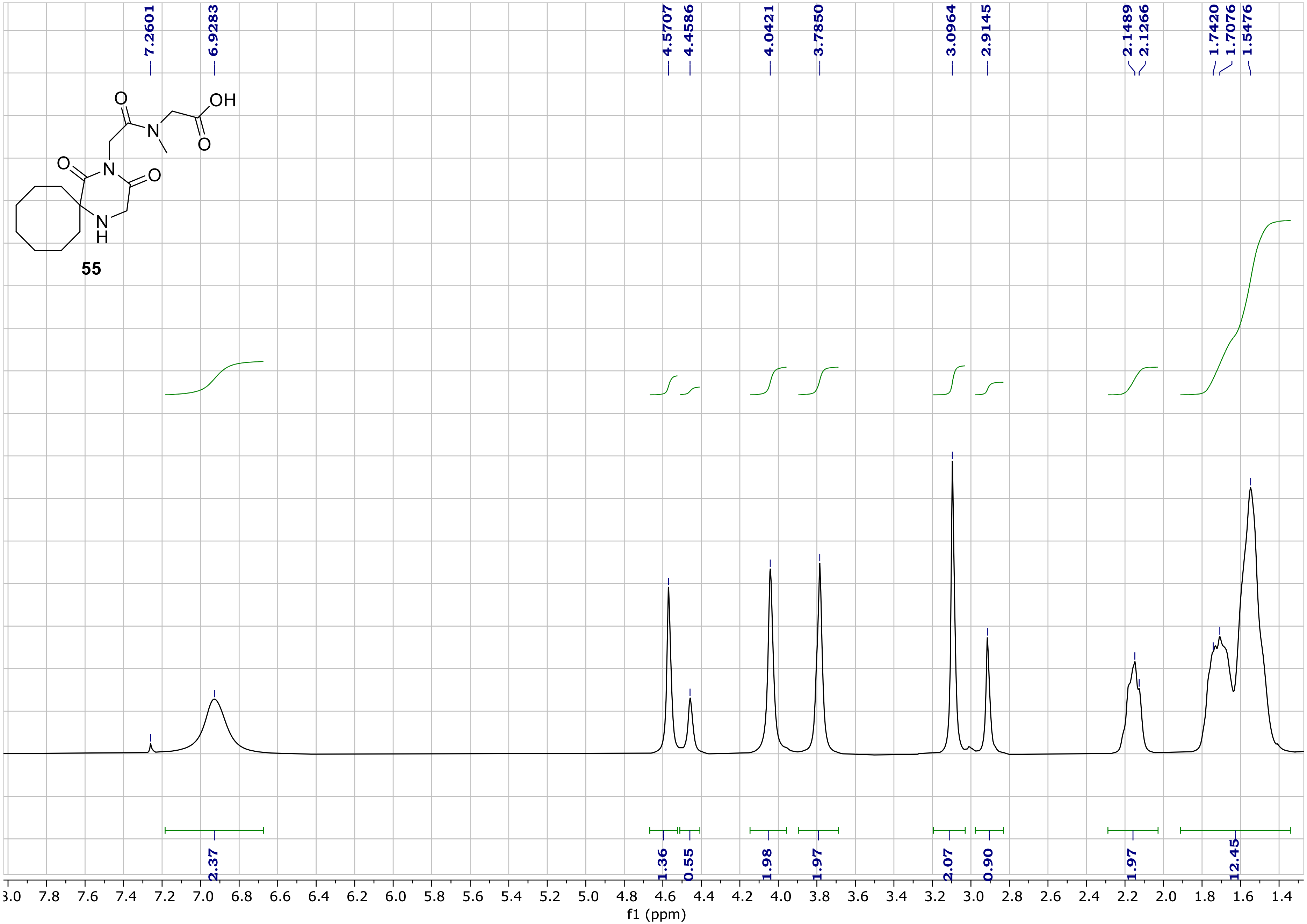

^{13}C NMR of **55** (50.32 MHz, $CDCl_3$)

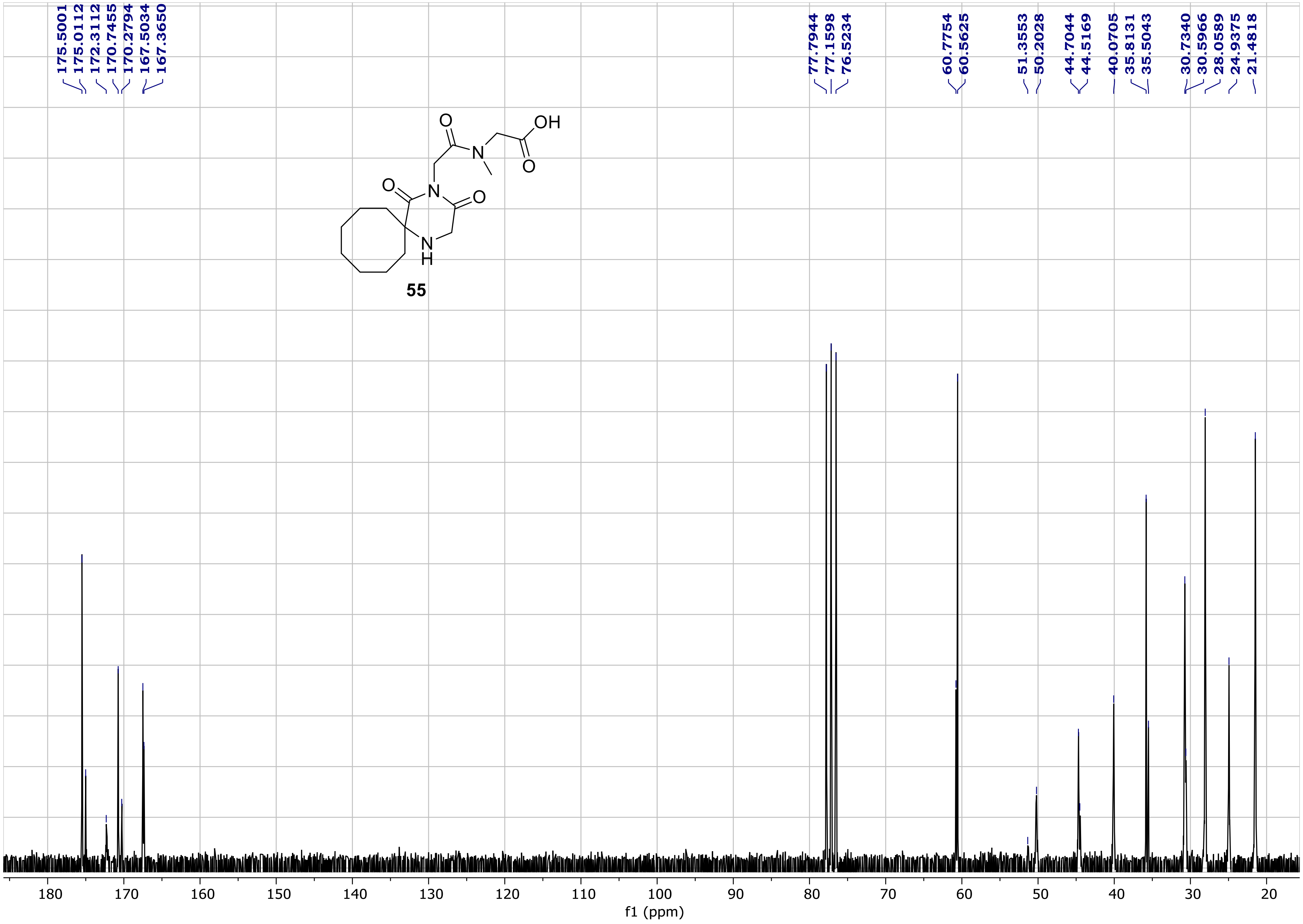

COSY NMR of **55** (400.11 MHz, $CDCl_3$)

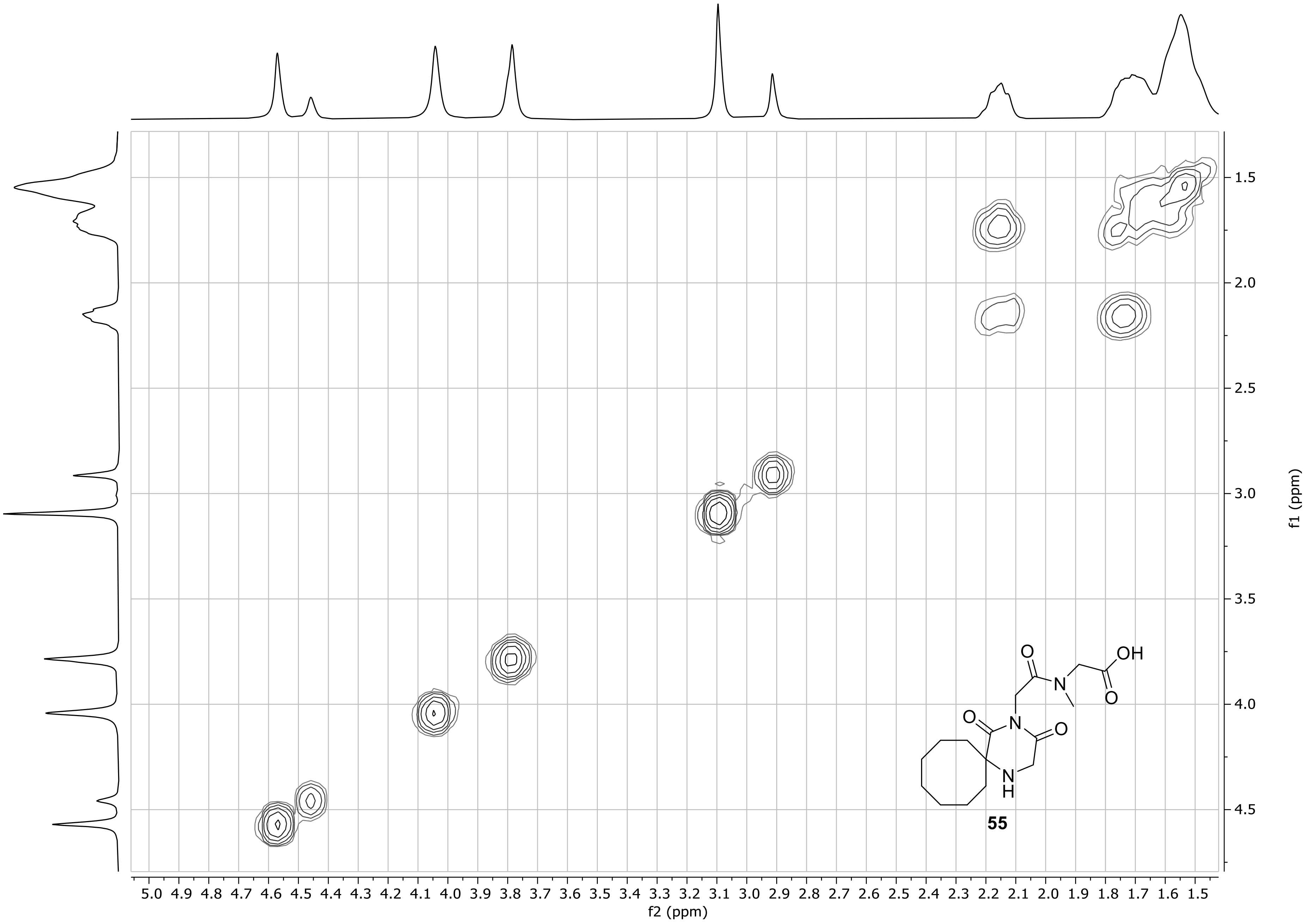

HSQC NMR of **55** (400.11 MHz, $CDCl_3$)

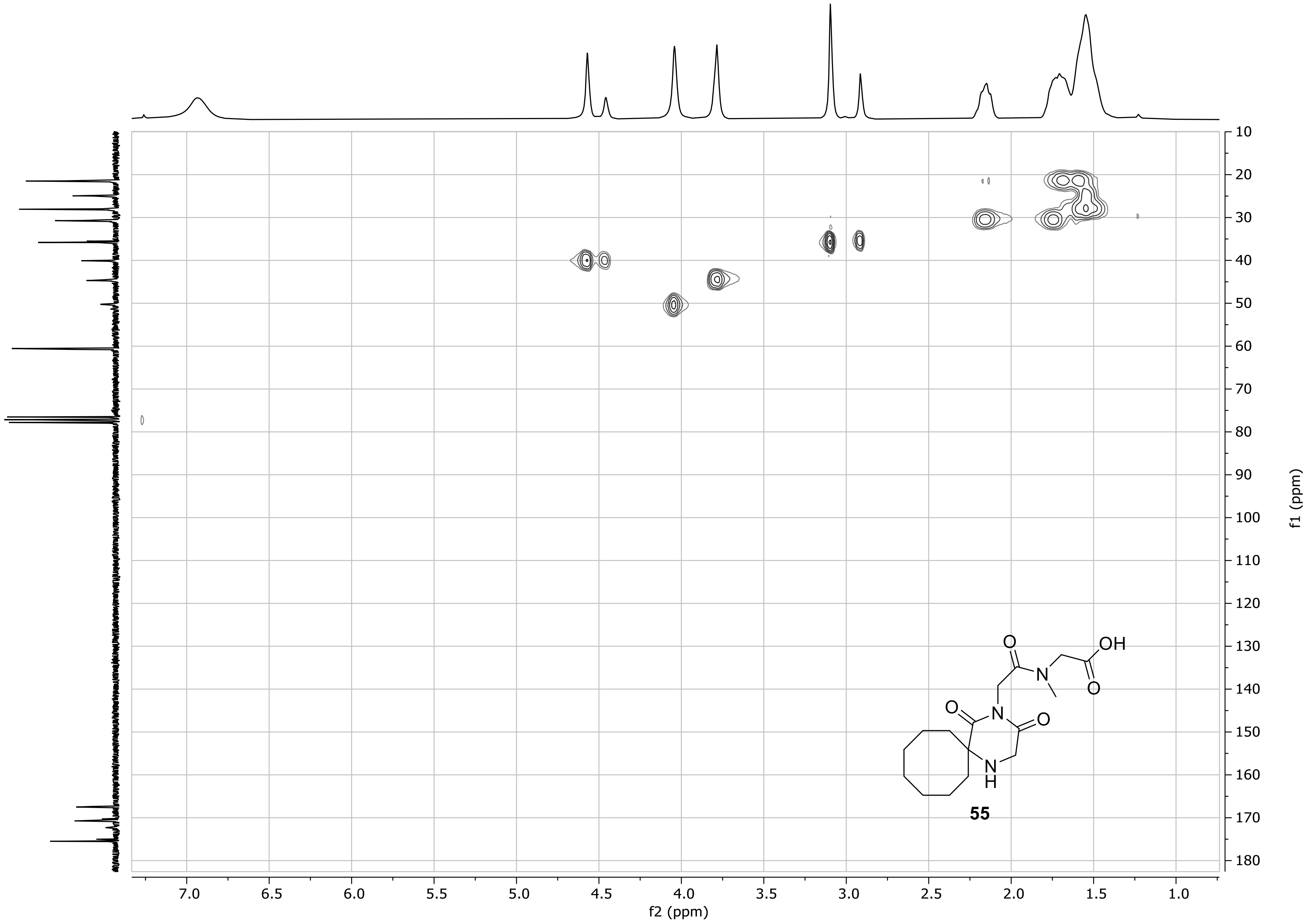

DEPT NMR of **55** (50.32 MHz, $CDCl_3$)

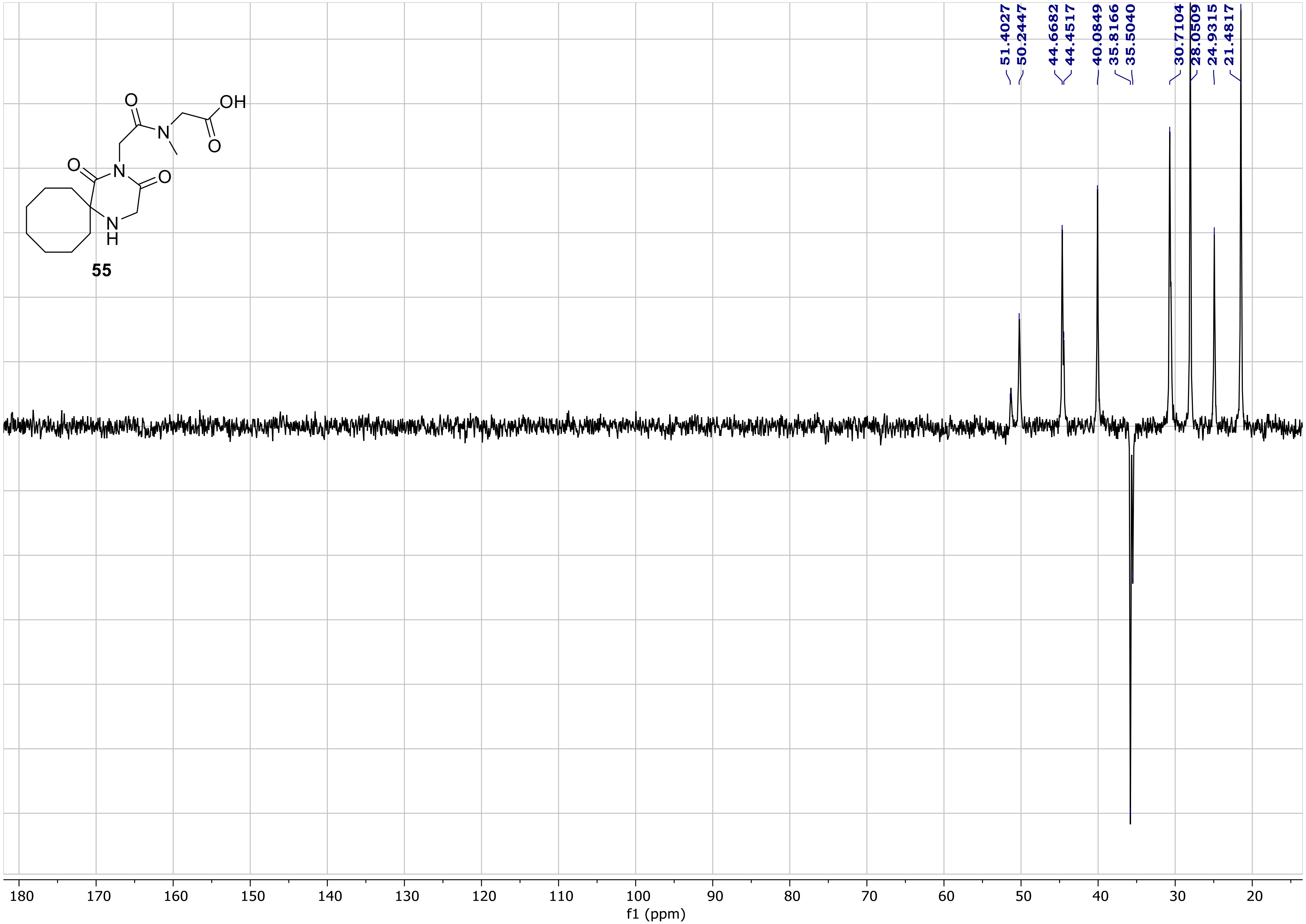

^{1}H NMR of **56** (400.11 MHz, DMSO-d_6)

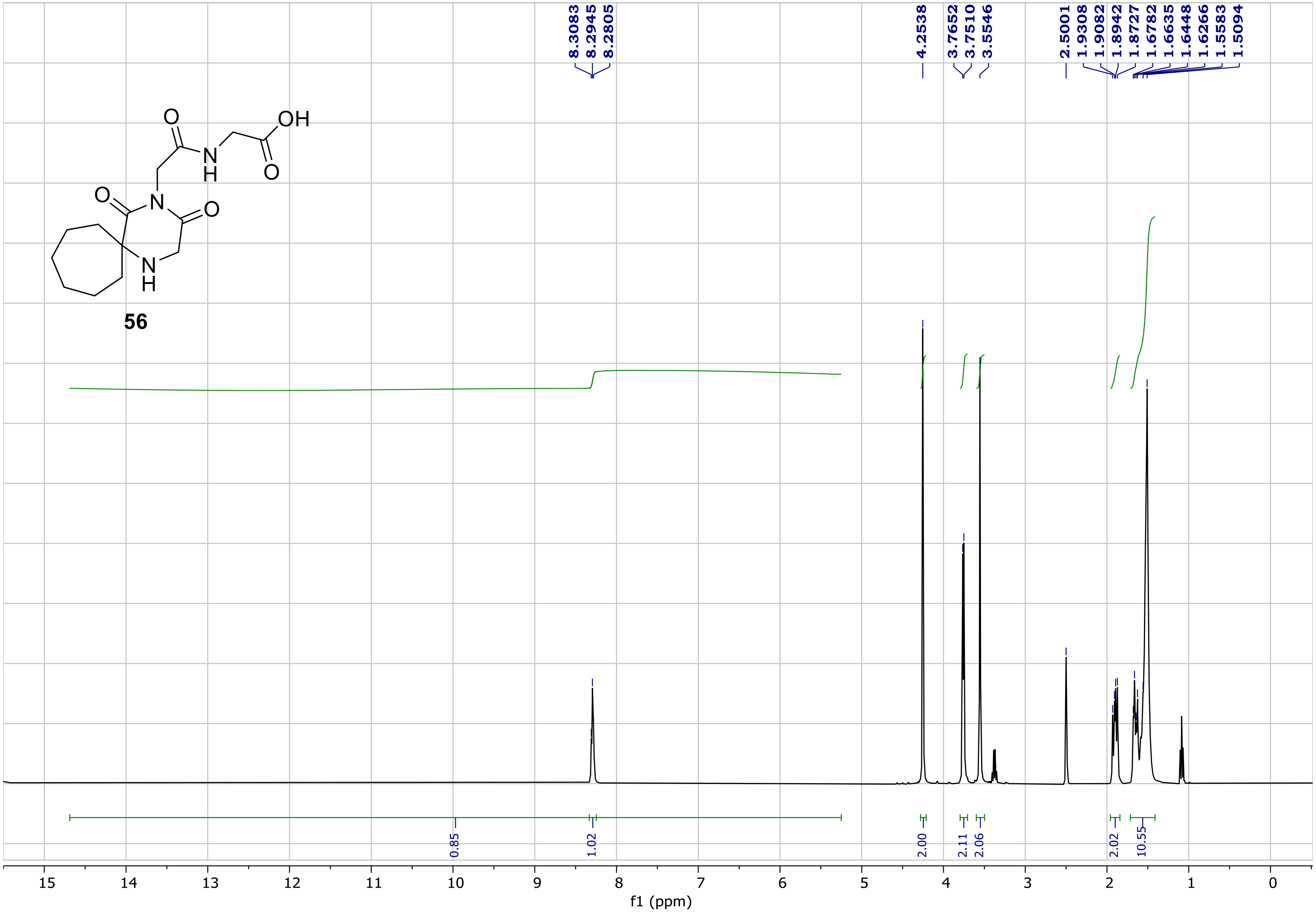

^{13}C NMR of **56** (50.32 MHz, DMSO-d_6)

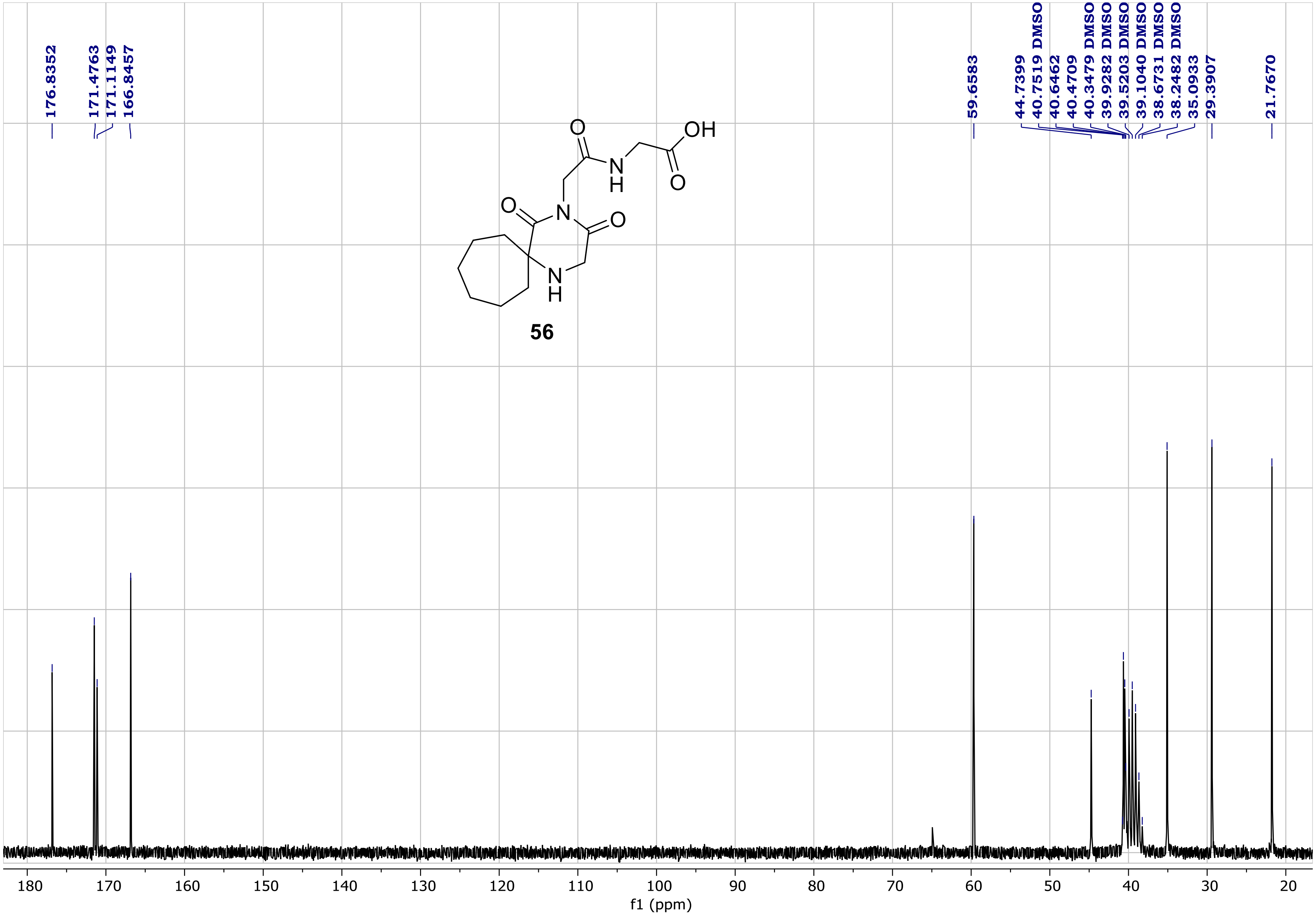

COSY NMR of **56** (400.11 MHz, DMSO-d_6)

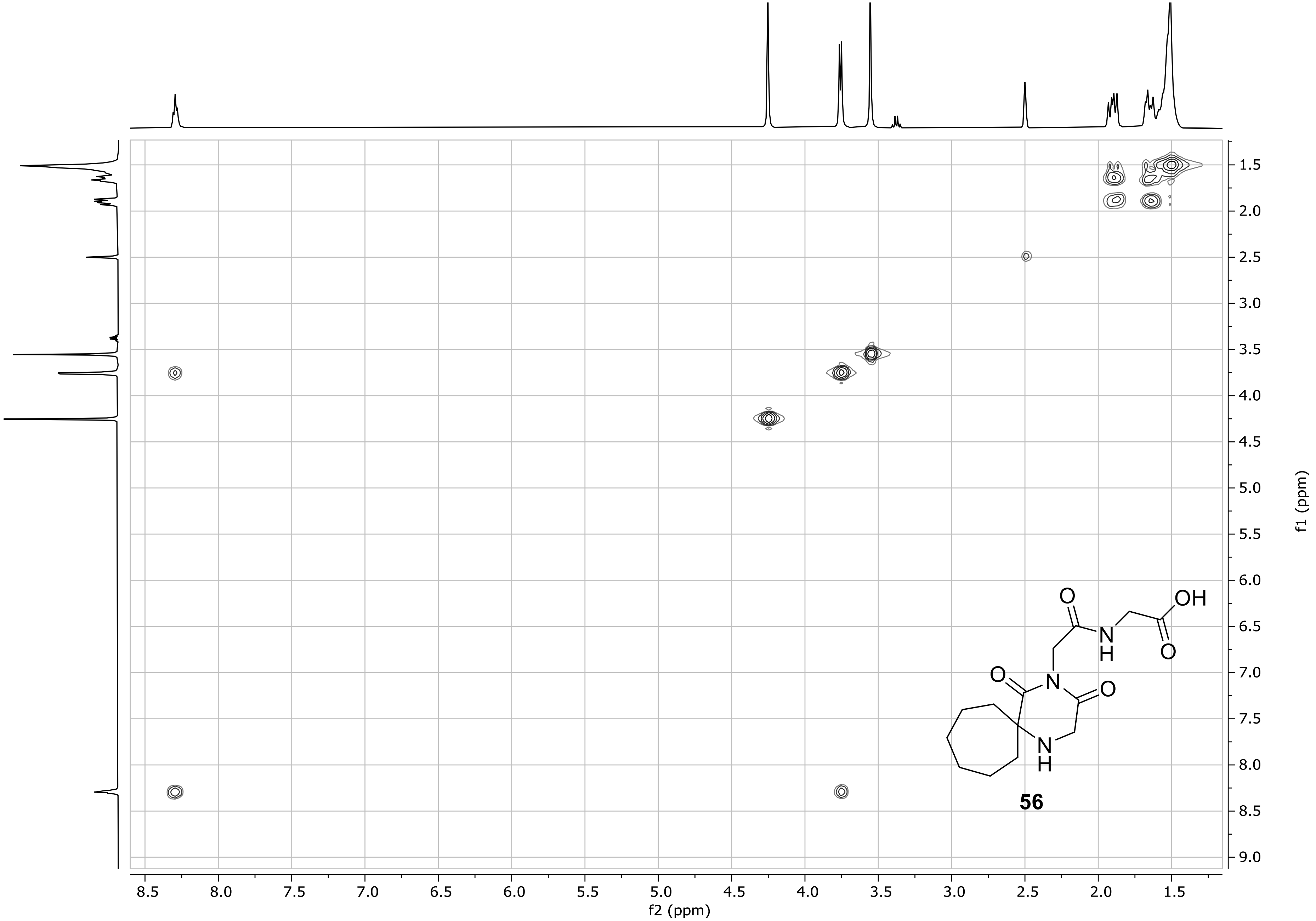

HSQC NMR of **56** (400.11 MHz, DMSO-d_6)

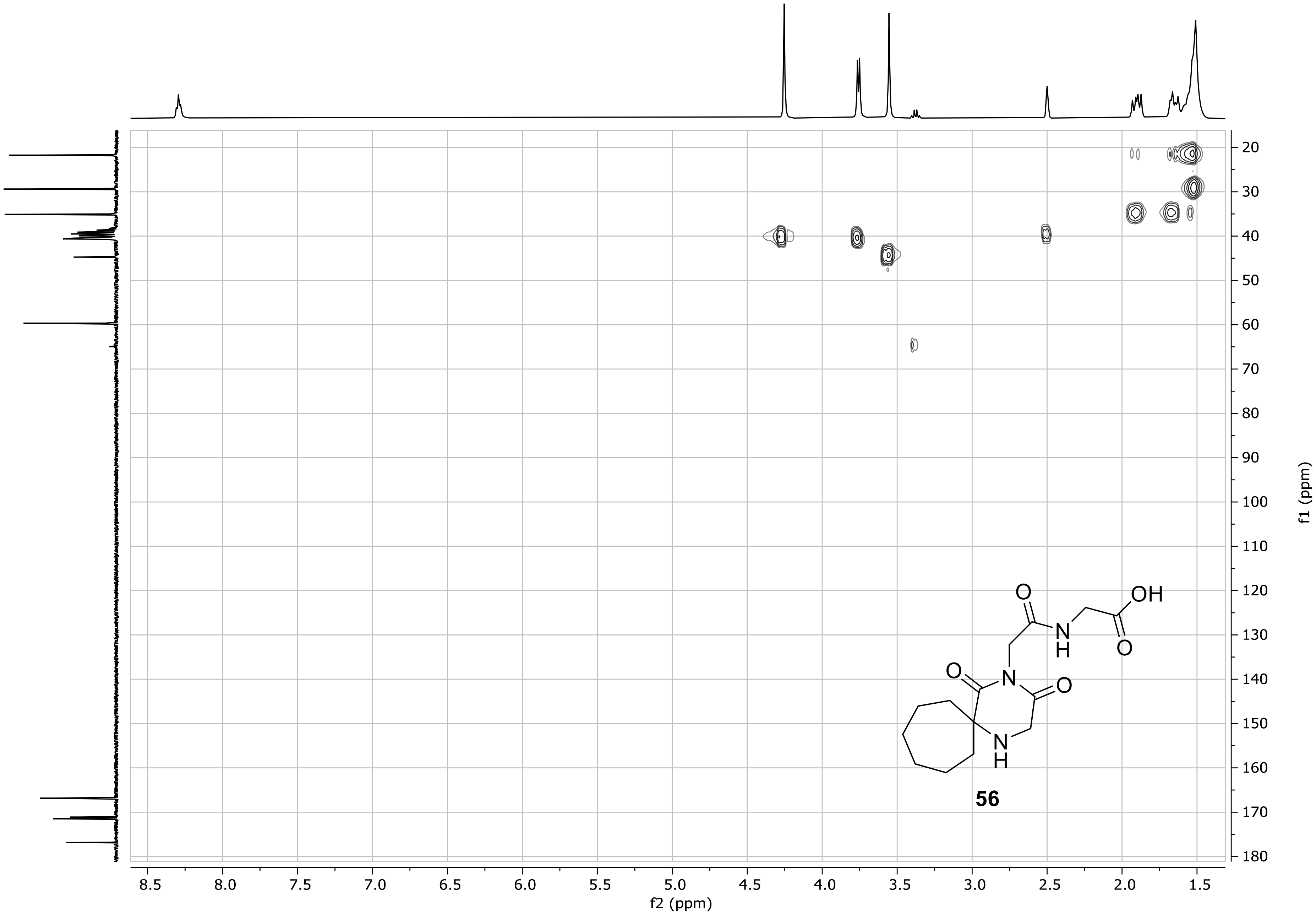

DEPT NMR of **56** (50.32 MHz, DMSO-d_6)

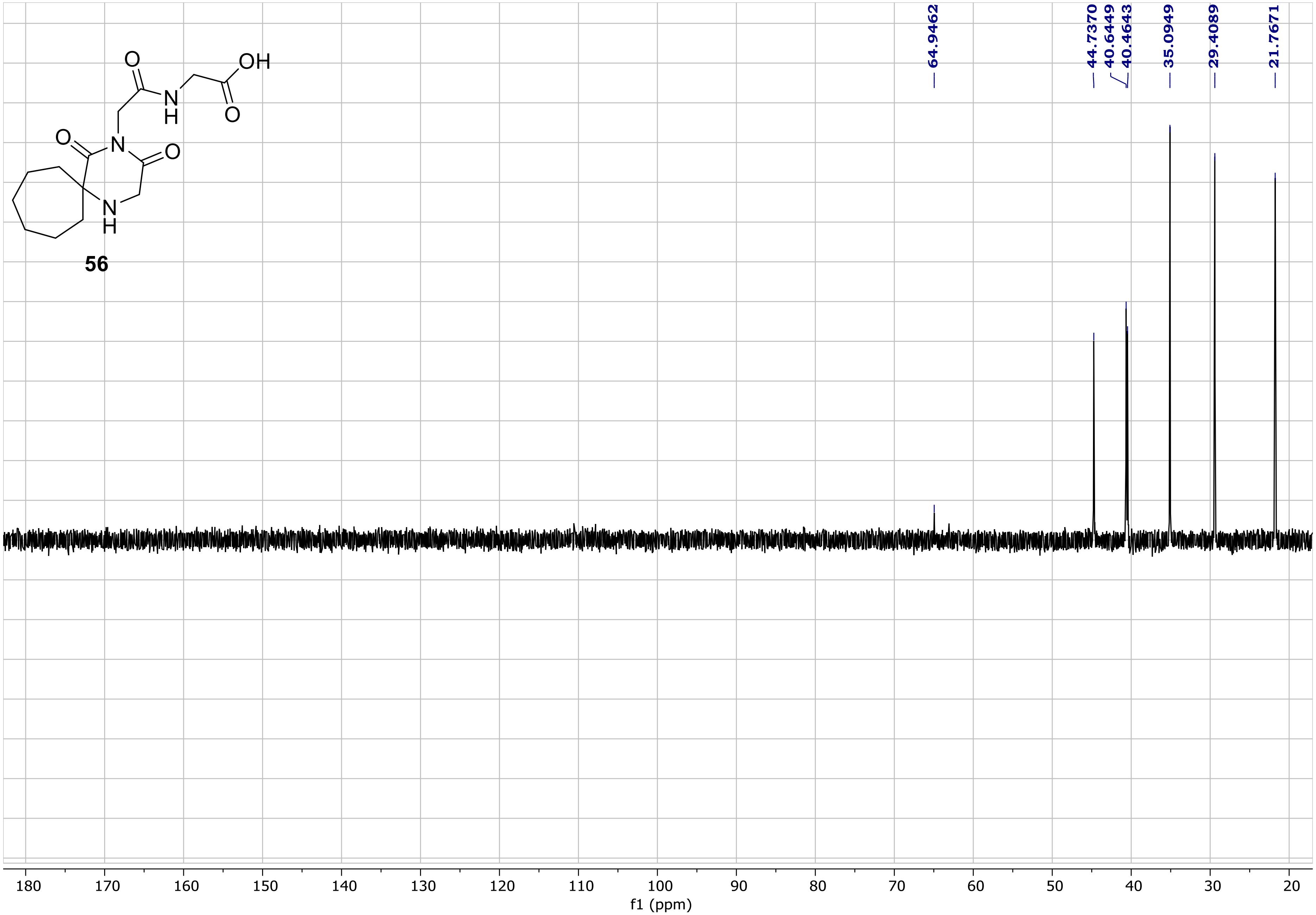

^1H NMR of **57** (400.11 MHz, $CDCl_3$)

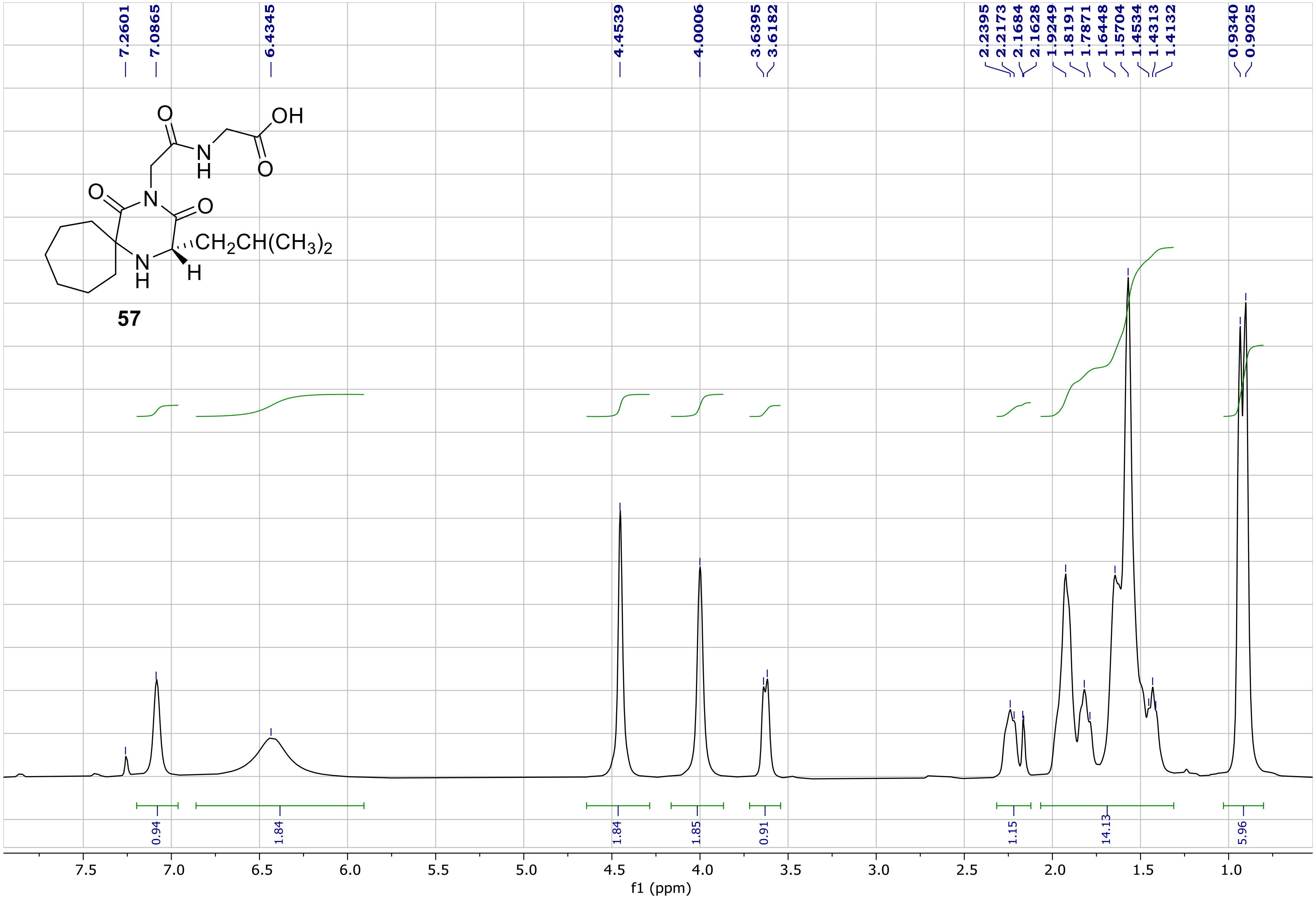

^{13}C NMR of **57** (50.32 MHz, $CDCl_3$)

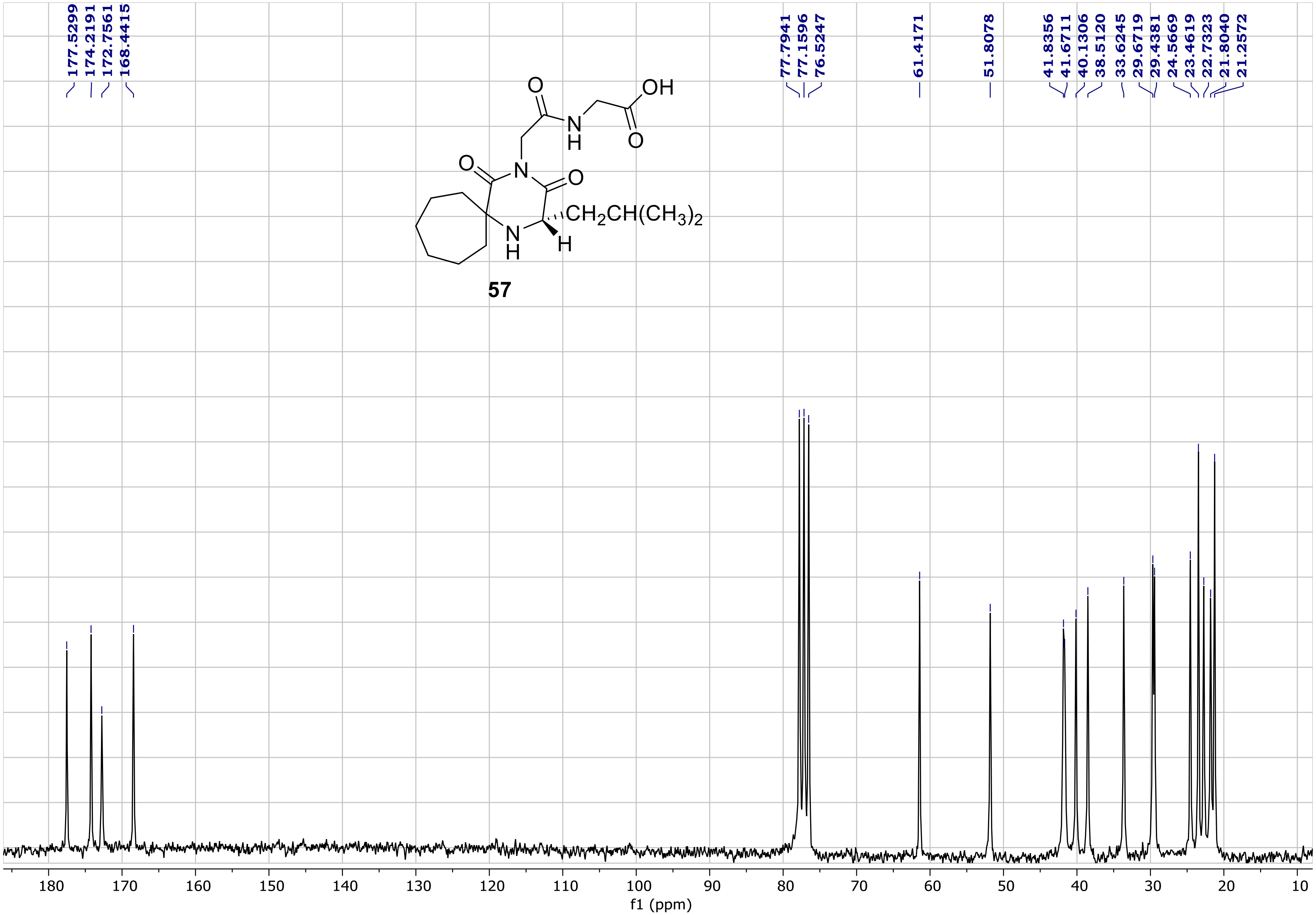

COSY NMR of **57** (400.11 MHz, $CDCl_3$)

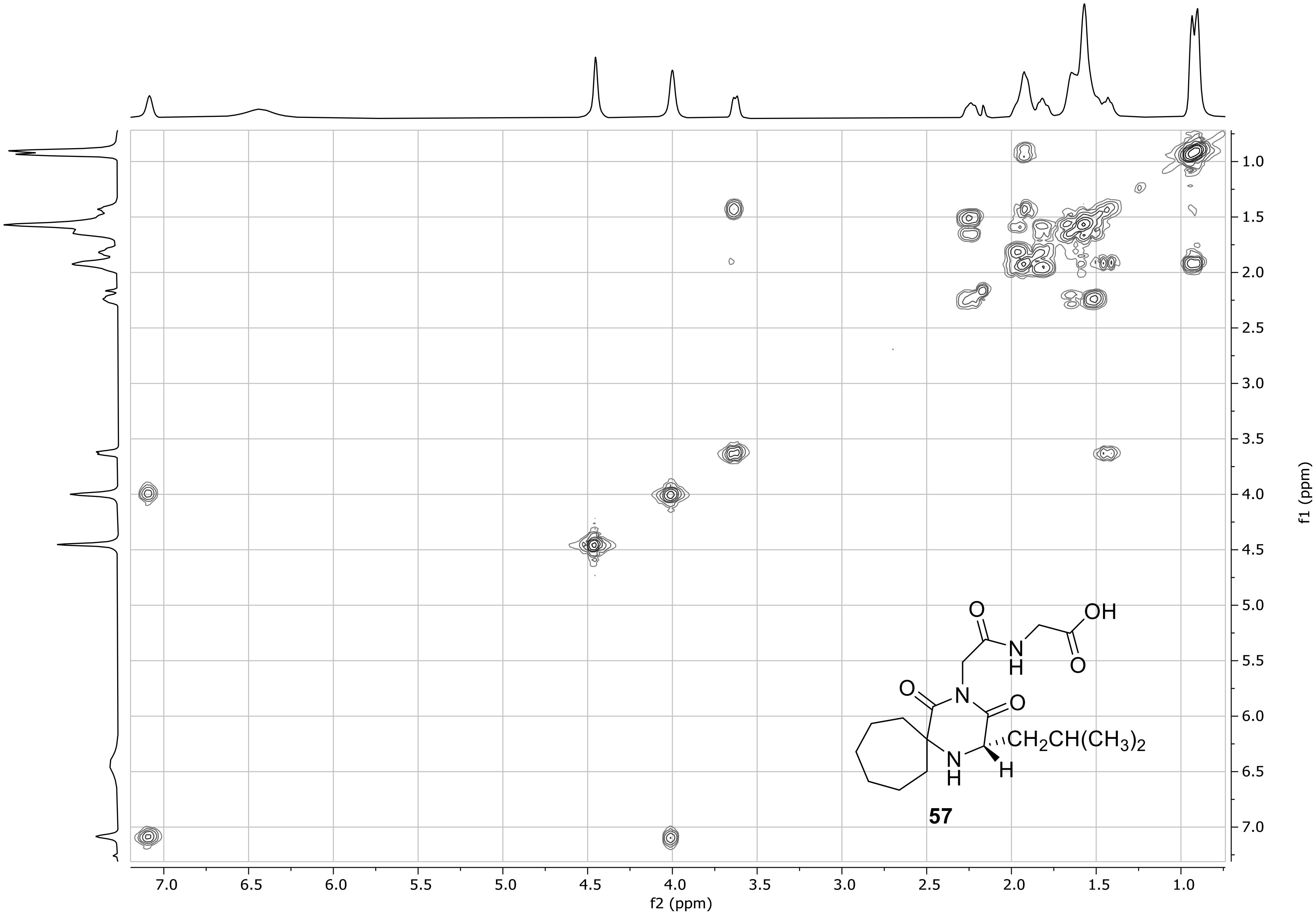

HSQC NMR of **57** (400.11 MHz, $CDCl_3$)

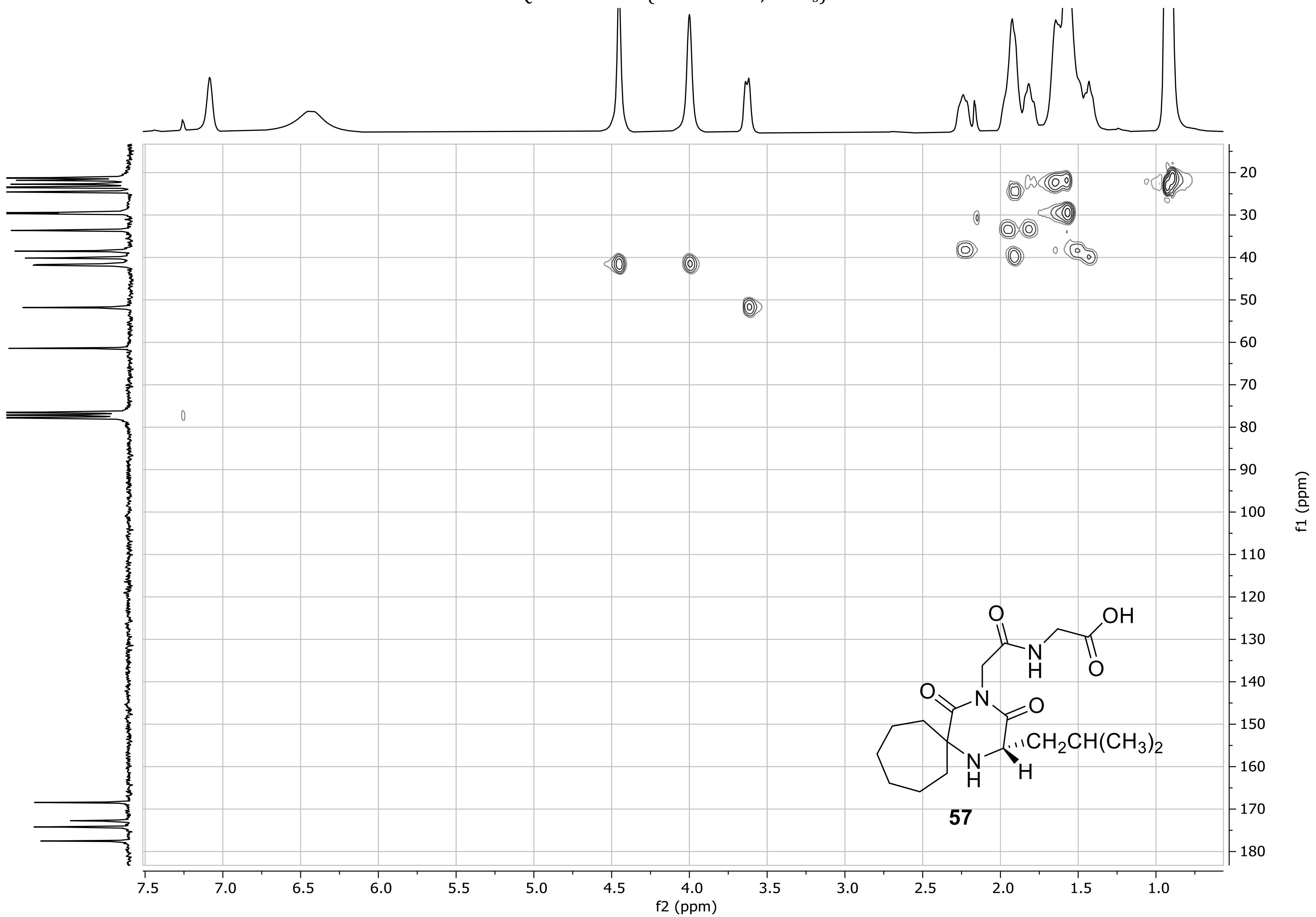

DEPT NMR of **57** (50.32 MHz, $CDCl_3$)

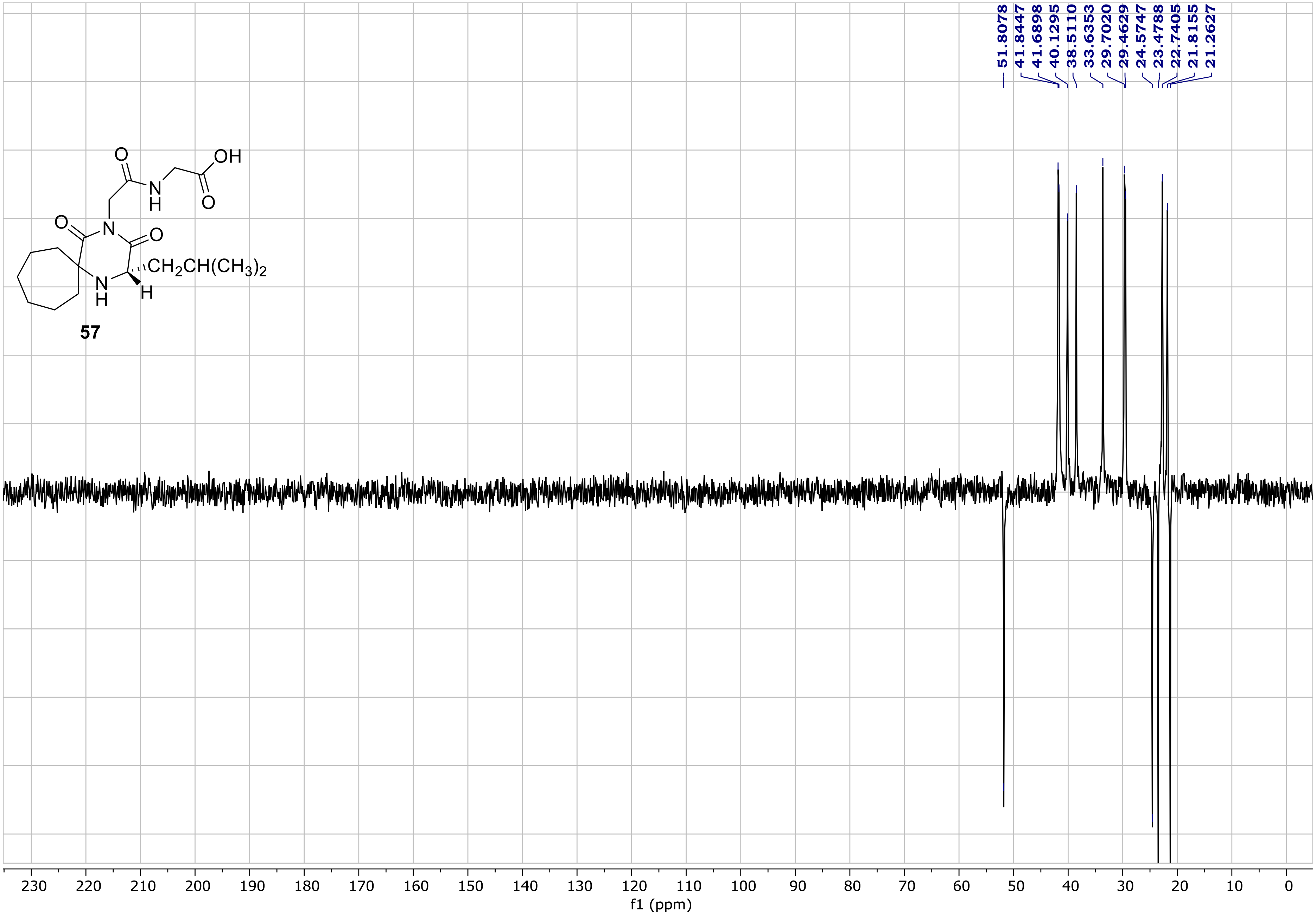

^{1}H NMR of **58** (400.11 MHz, DMSO-d_6)

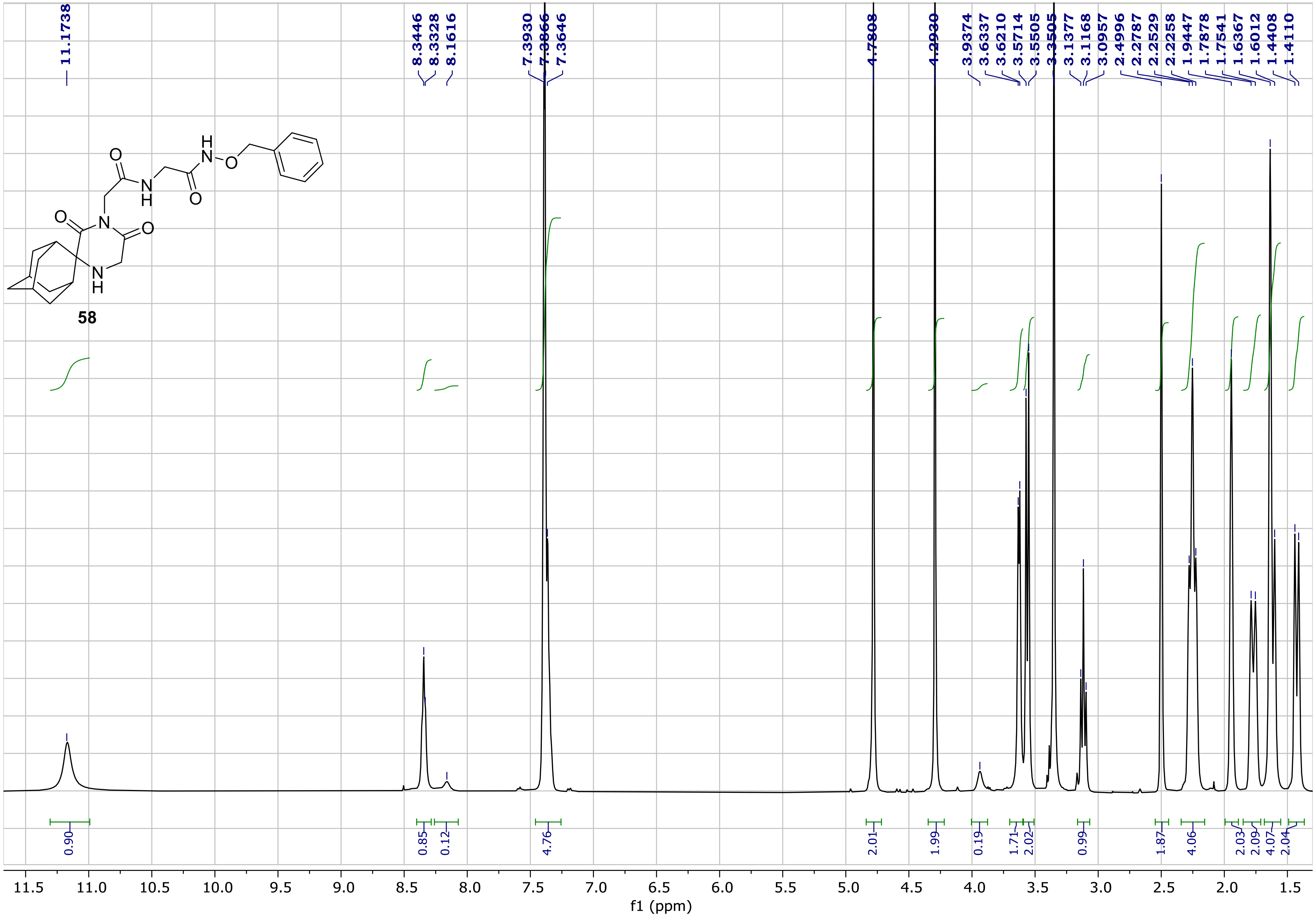

^{13}C NMR of **58** (50.32 MHz, DMSO-d_6)

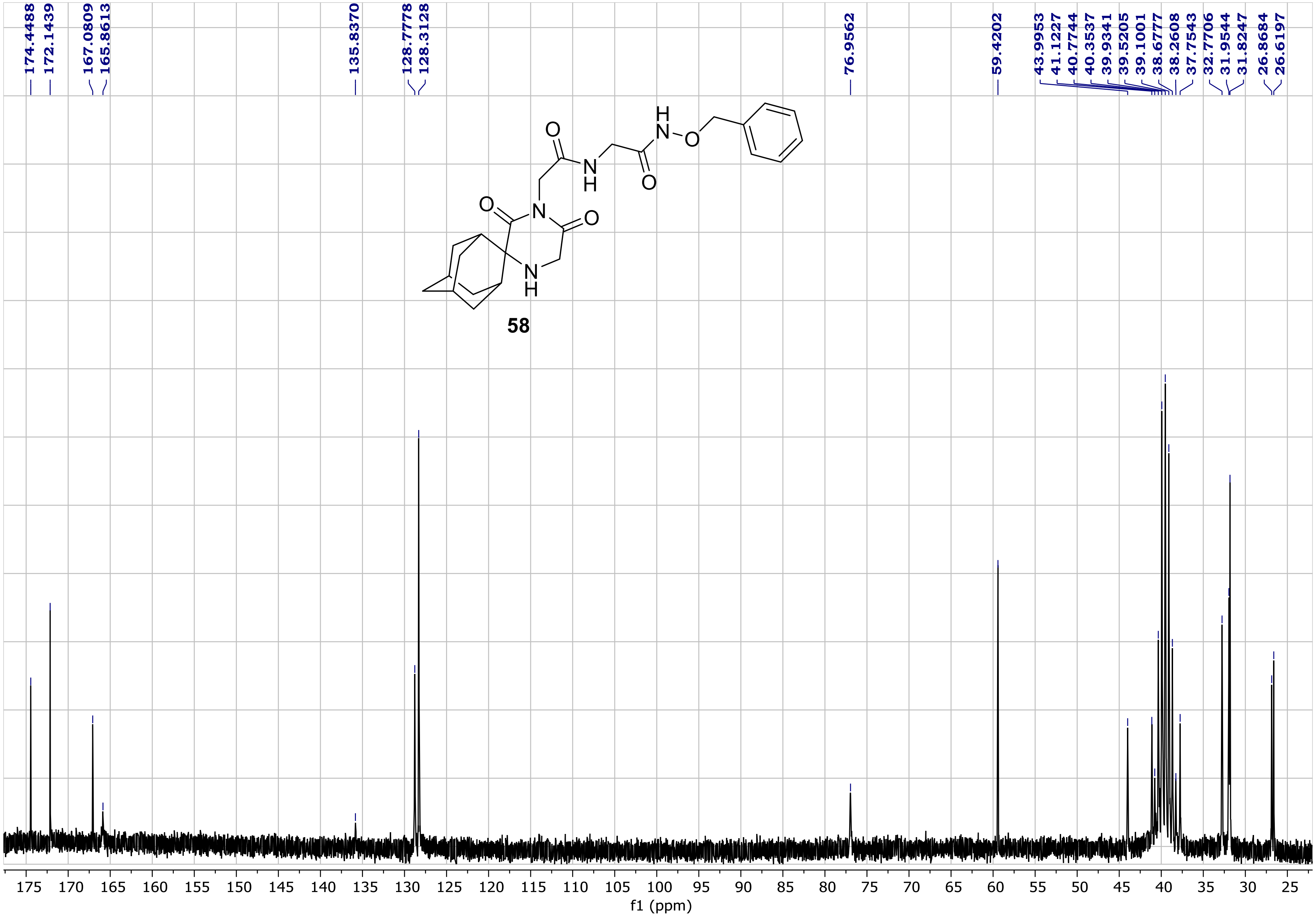

COSY NMR of **58** (400.11 MHz, DMSO-d_6)

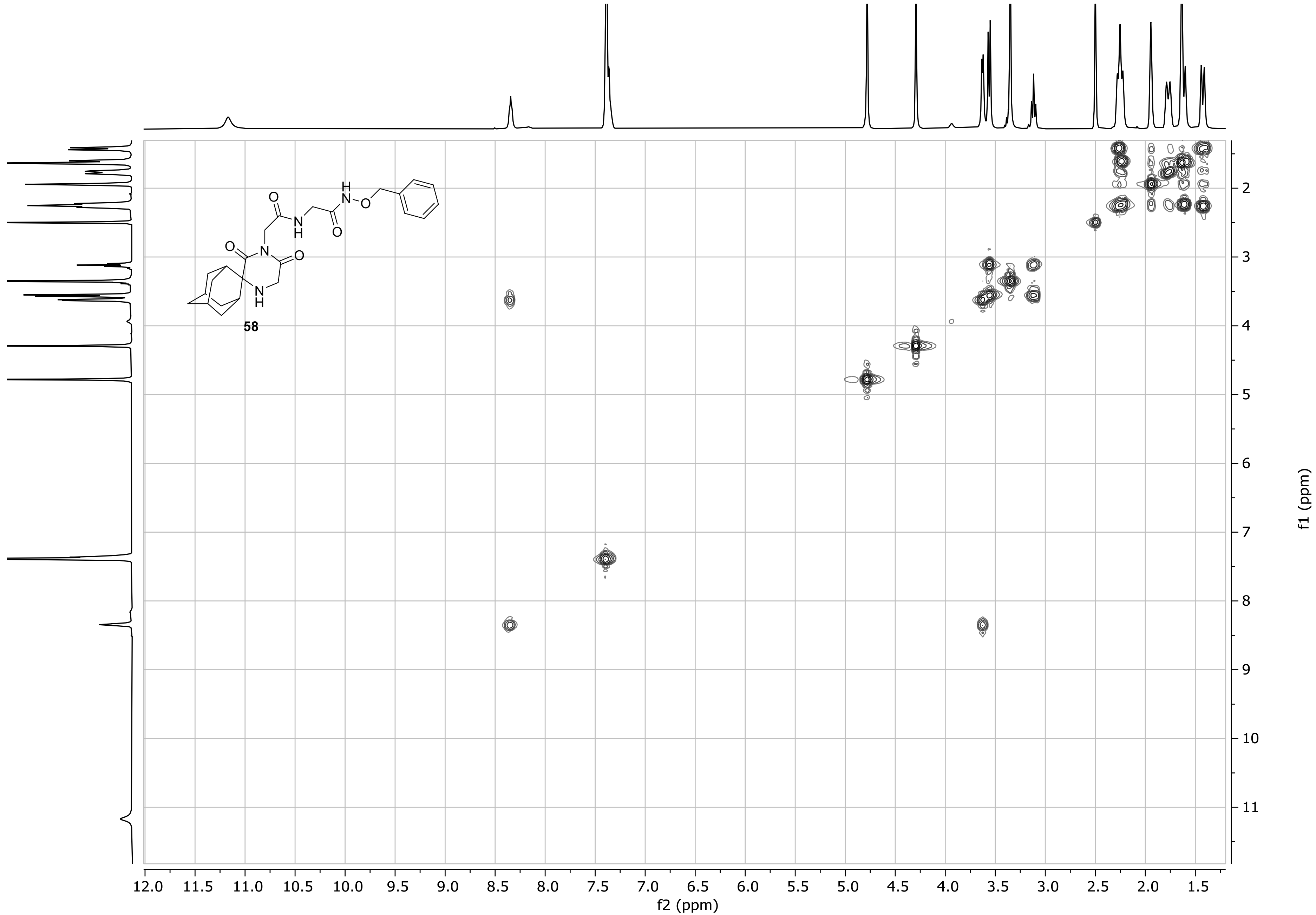

HSQC NMR of **58** (400.11 MHz, DMSO-d_6)

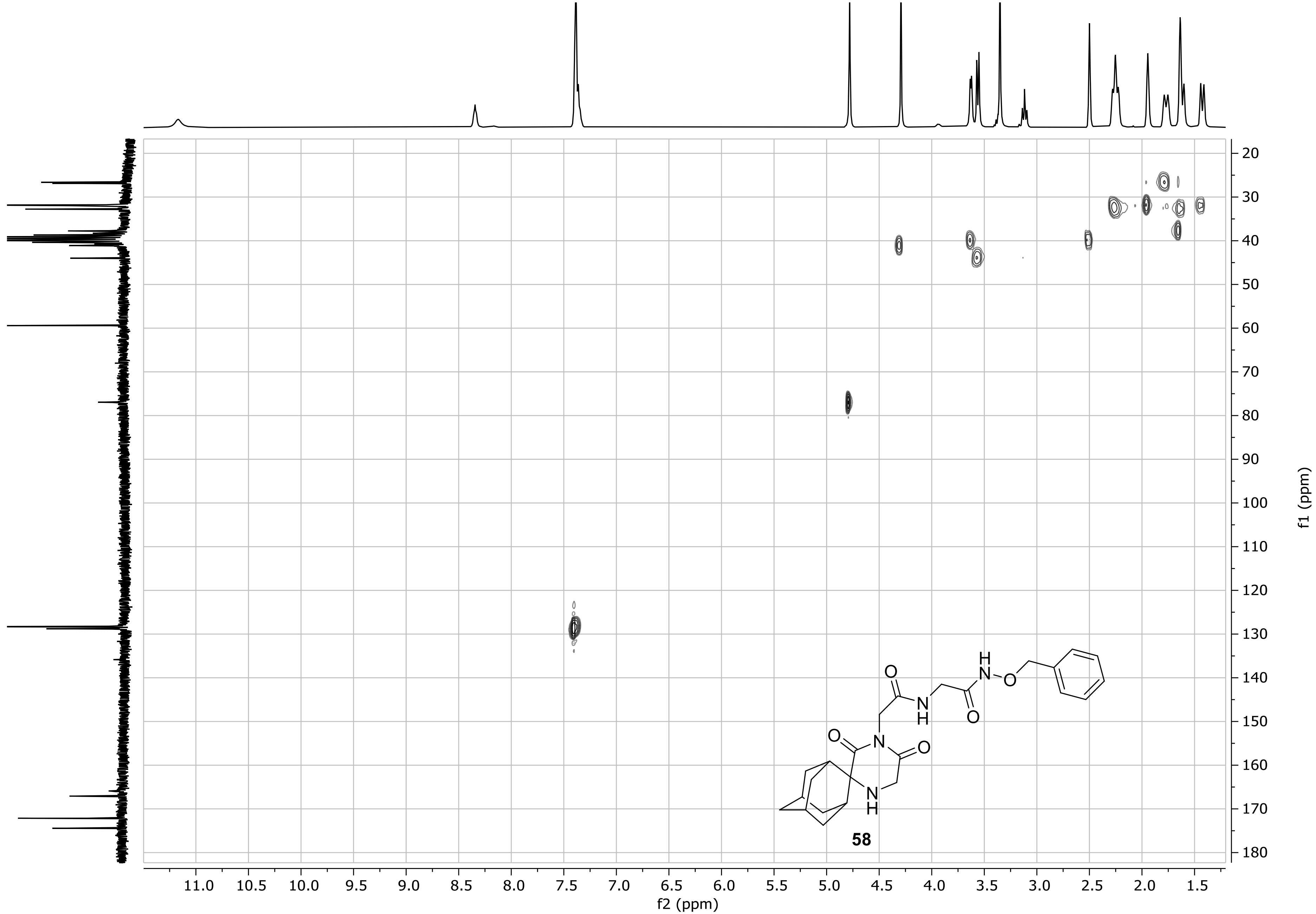

DEPT NMR of **58** (50.32 MHz, DMSO-d_6)

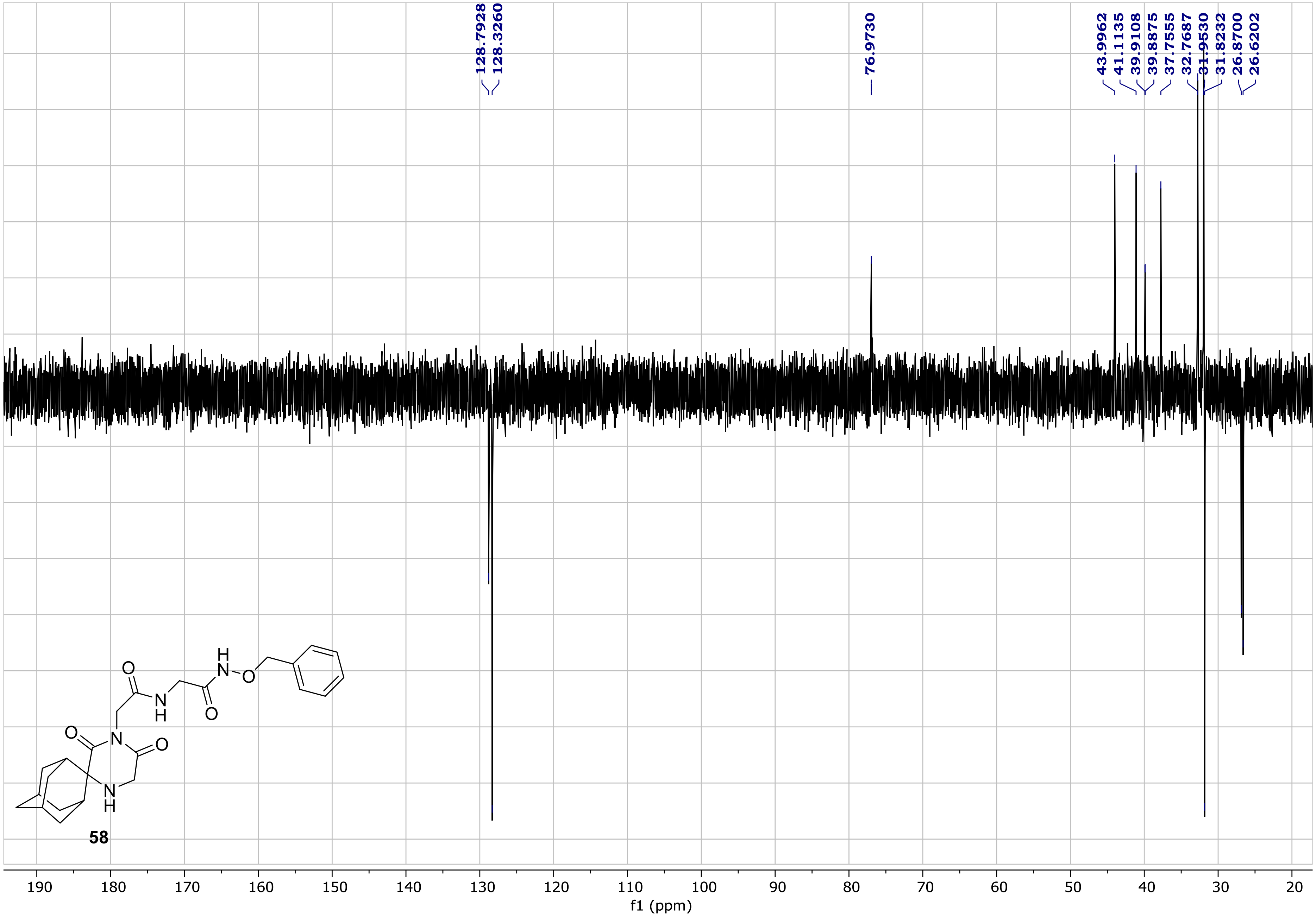

58

^{1}H NMR of **59** (600.11 MHz, $CDCl_3$)

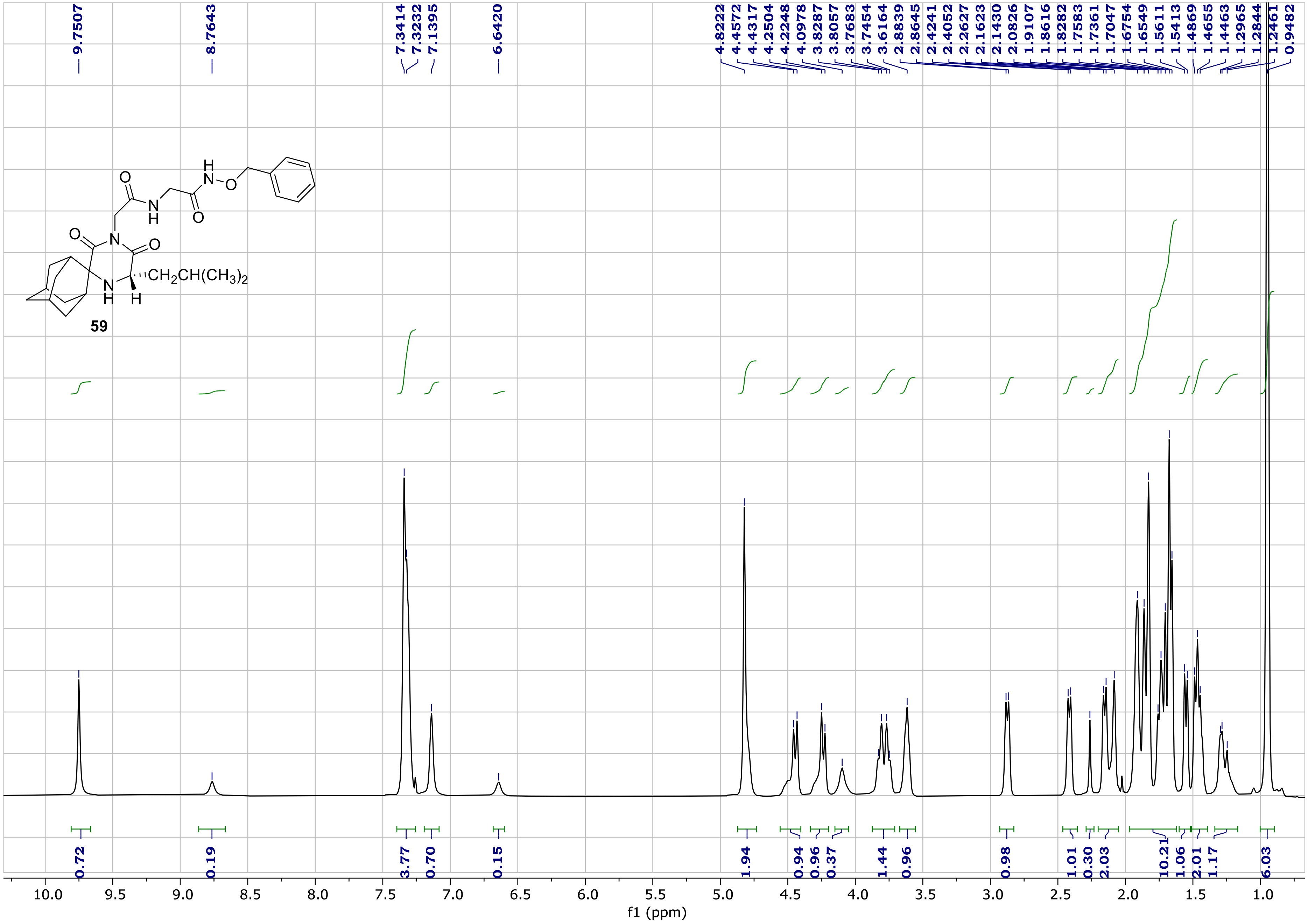

^{13}C NMR of **59** (50.32 MHz, $CDCl_3$)

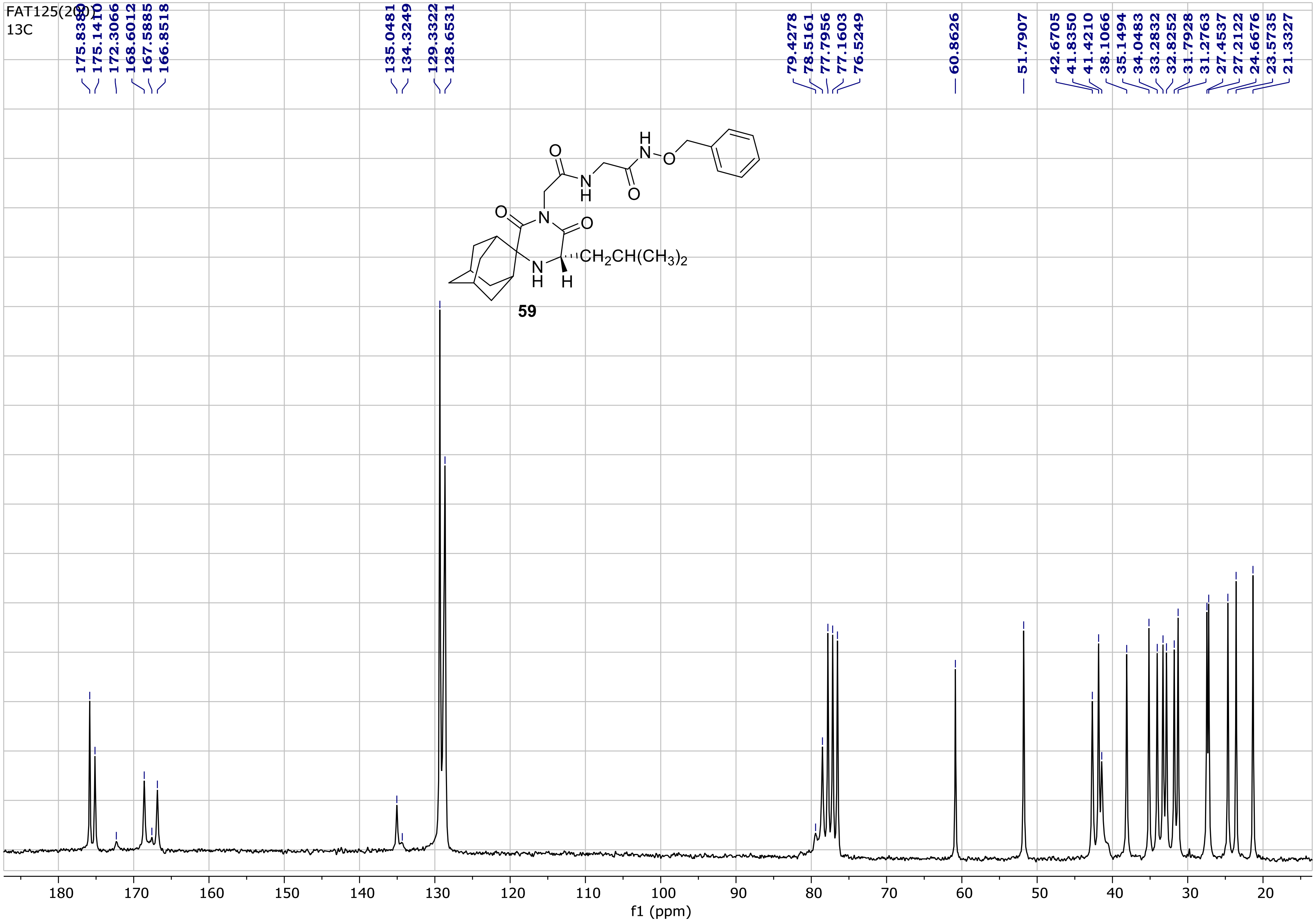

COSY NMR of **59** (600.11 MHz, $CDCl_3$)

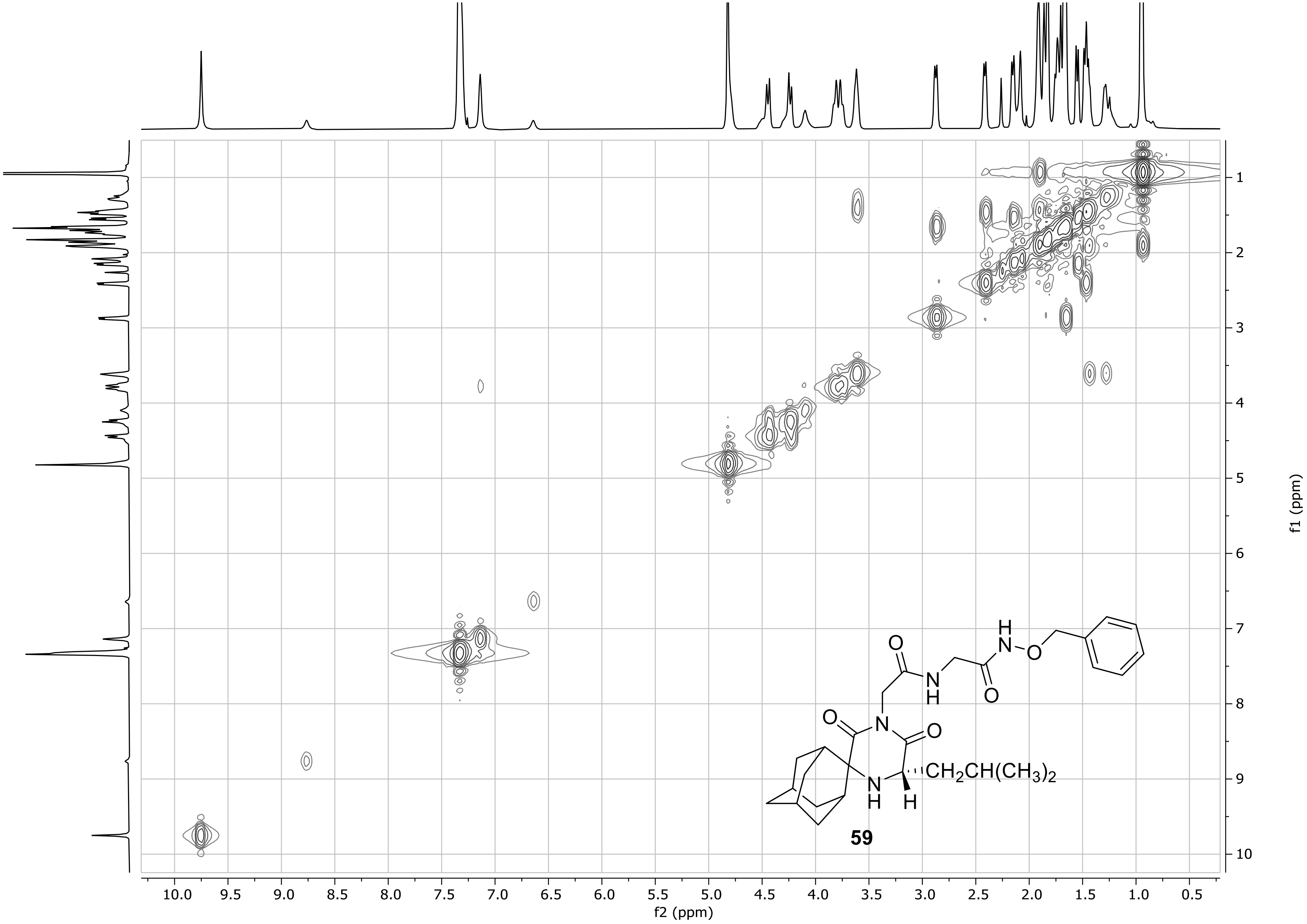

HSQC-DEPT NMR of **59** (600.11 MHz, $CDCl_3$)

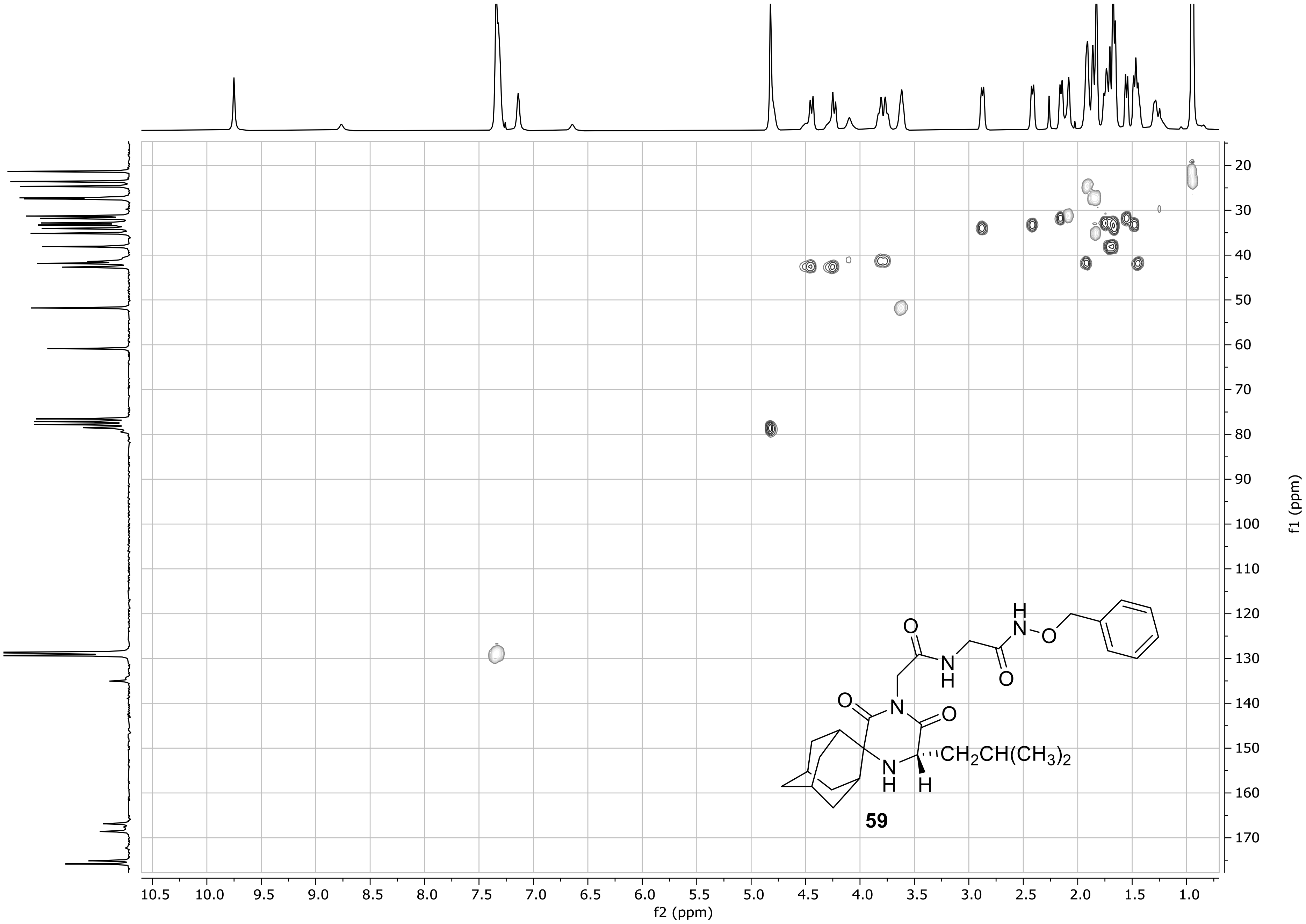

DEPT NMR of **59** (50.32 MHz, $CDCl_3$)

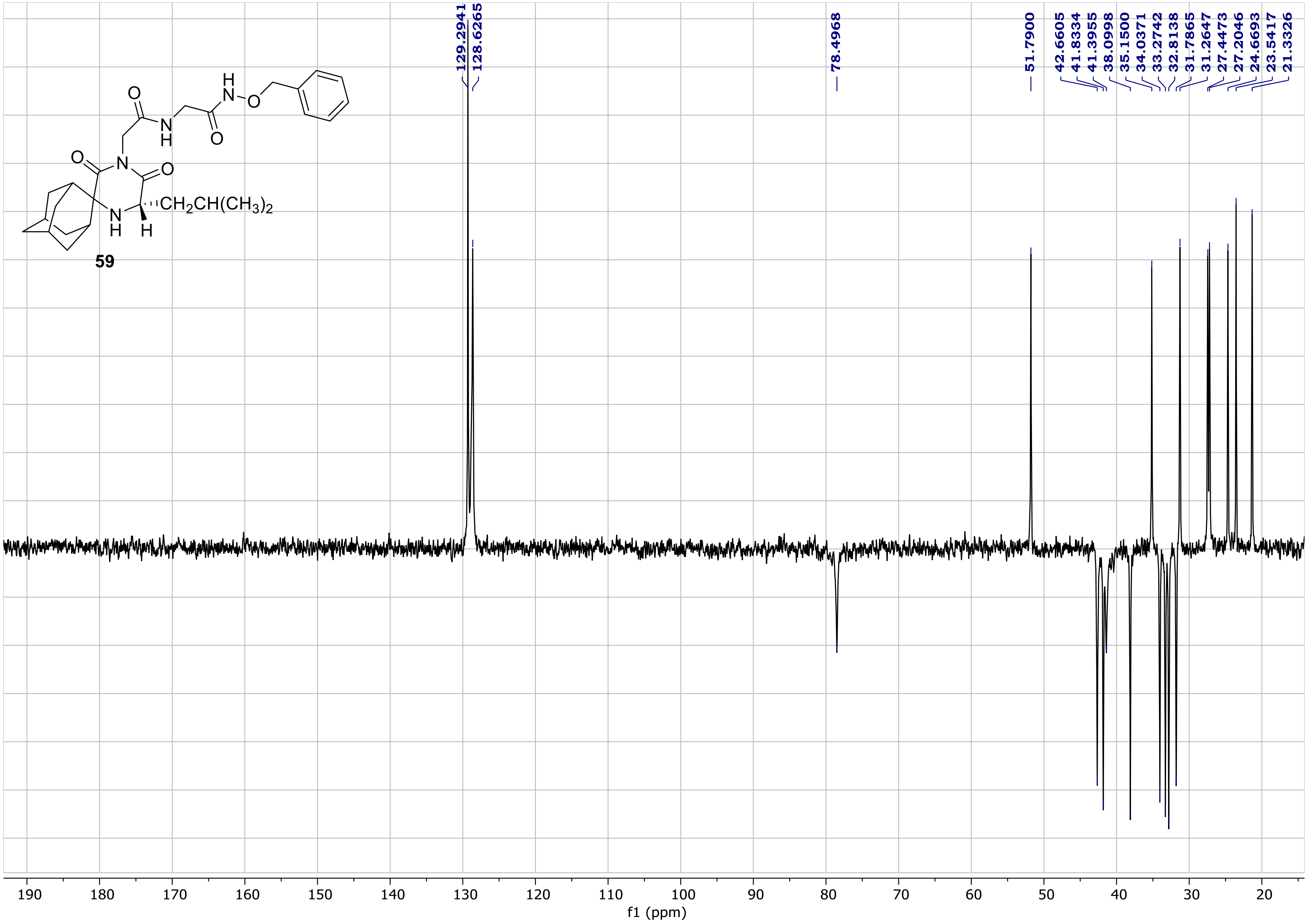

^{1}H NMR of **60** (400.11 MHz, $CDCl_3$)

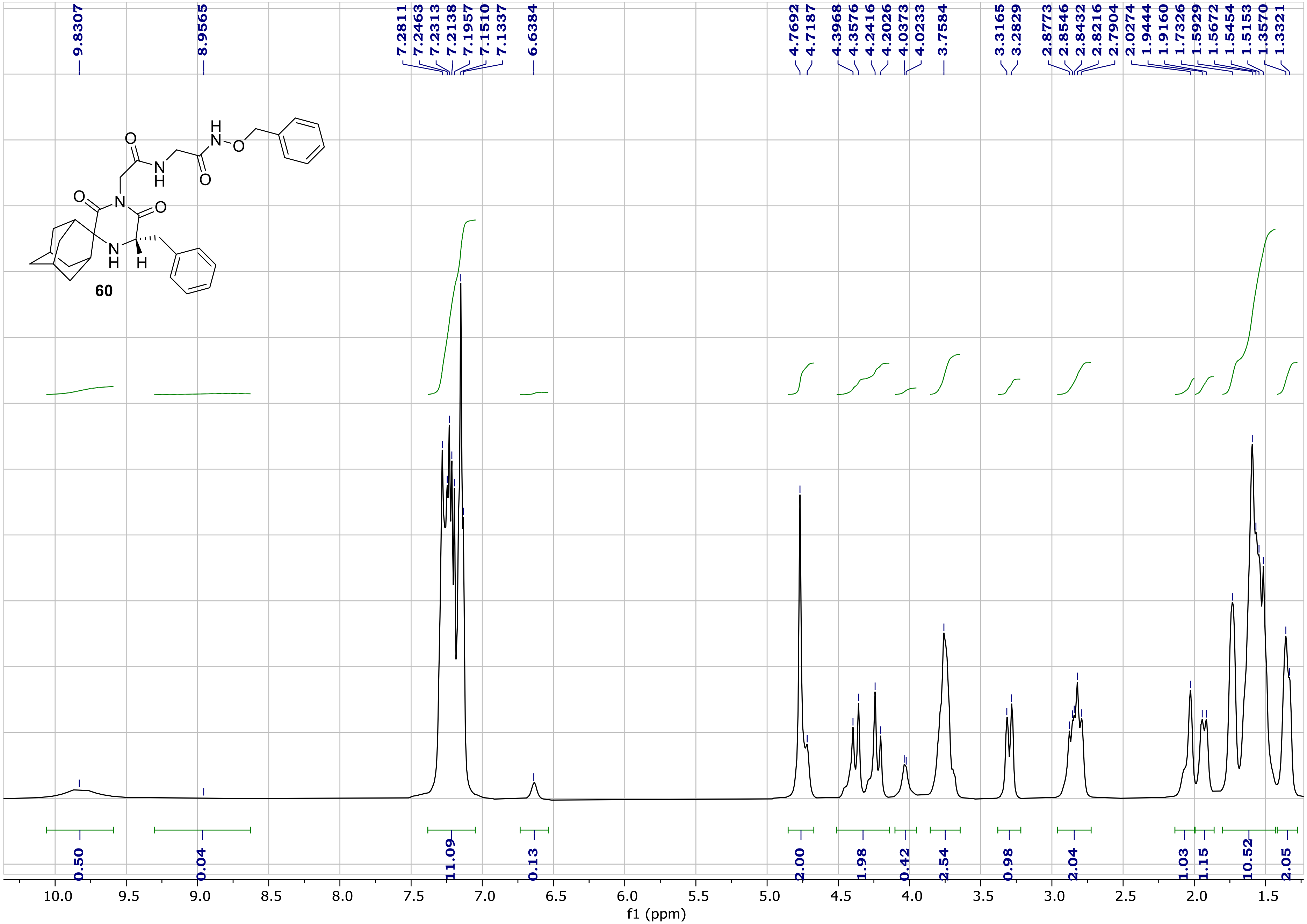

^{13}C NMR of **60** (50.32 MHz, $CDCl_3$)

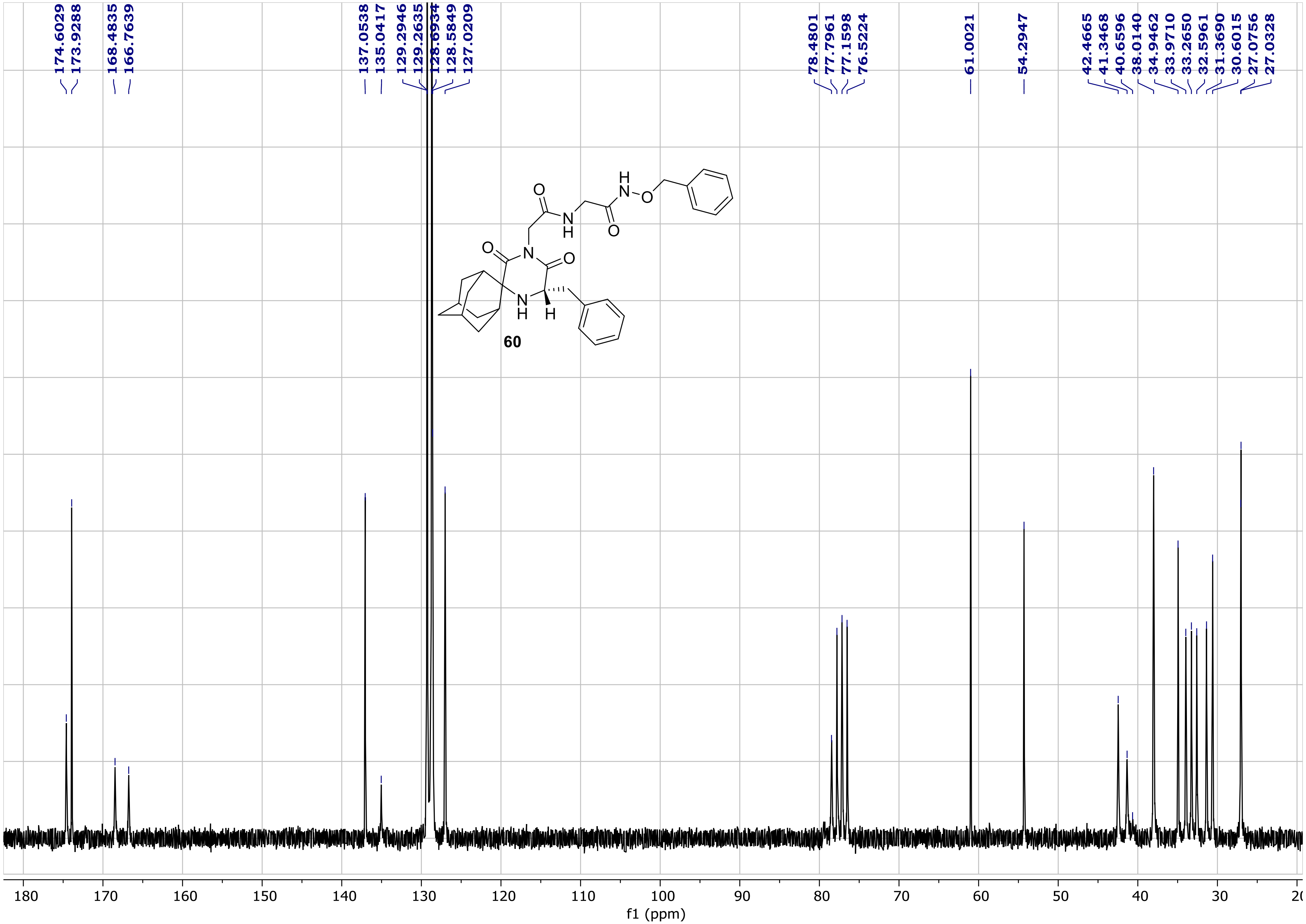

COSY NMR of **60** (400.11 MHz, $CDCl_3$)

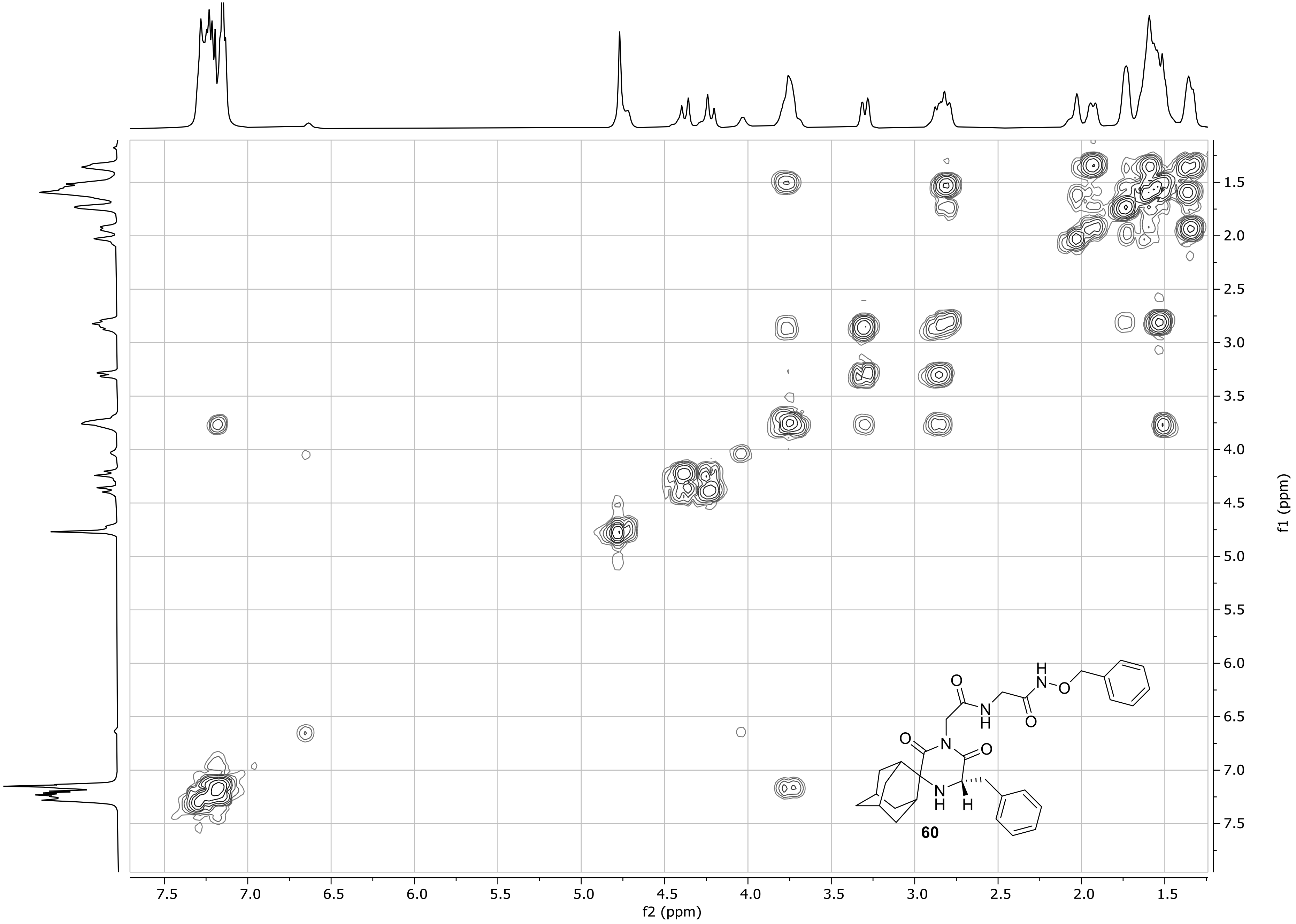

HSQC NMR of **60** (400.11 MHz, $CDCl_3$)

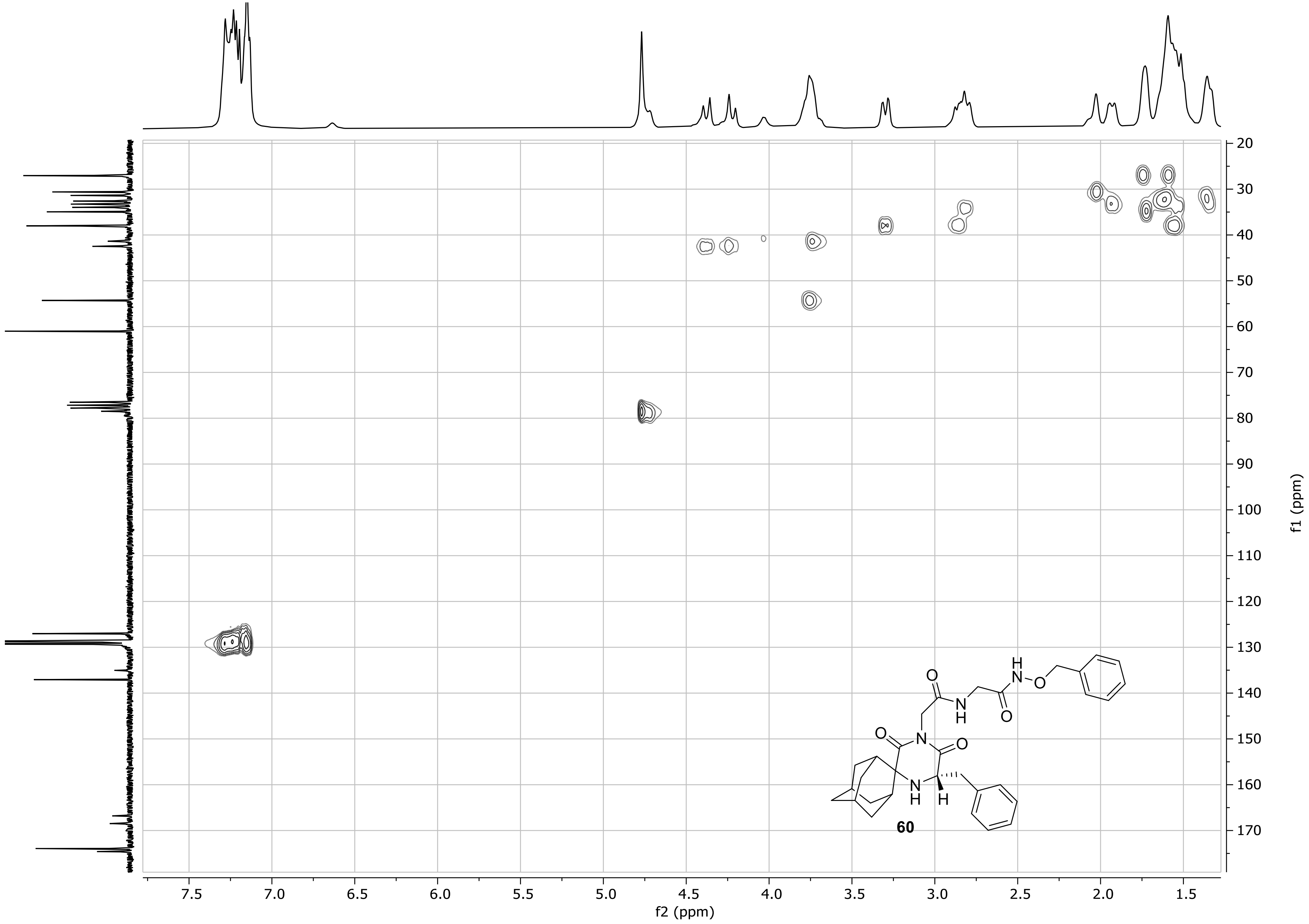

DEPT NMR of **60** (50.32 MHz, $CDCl_3$)

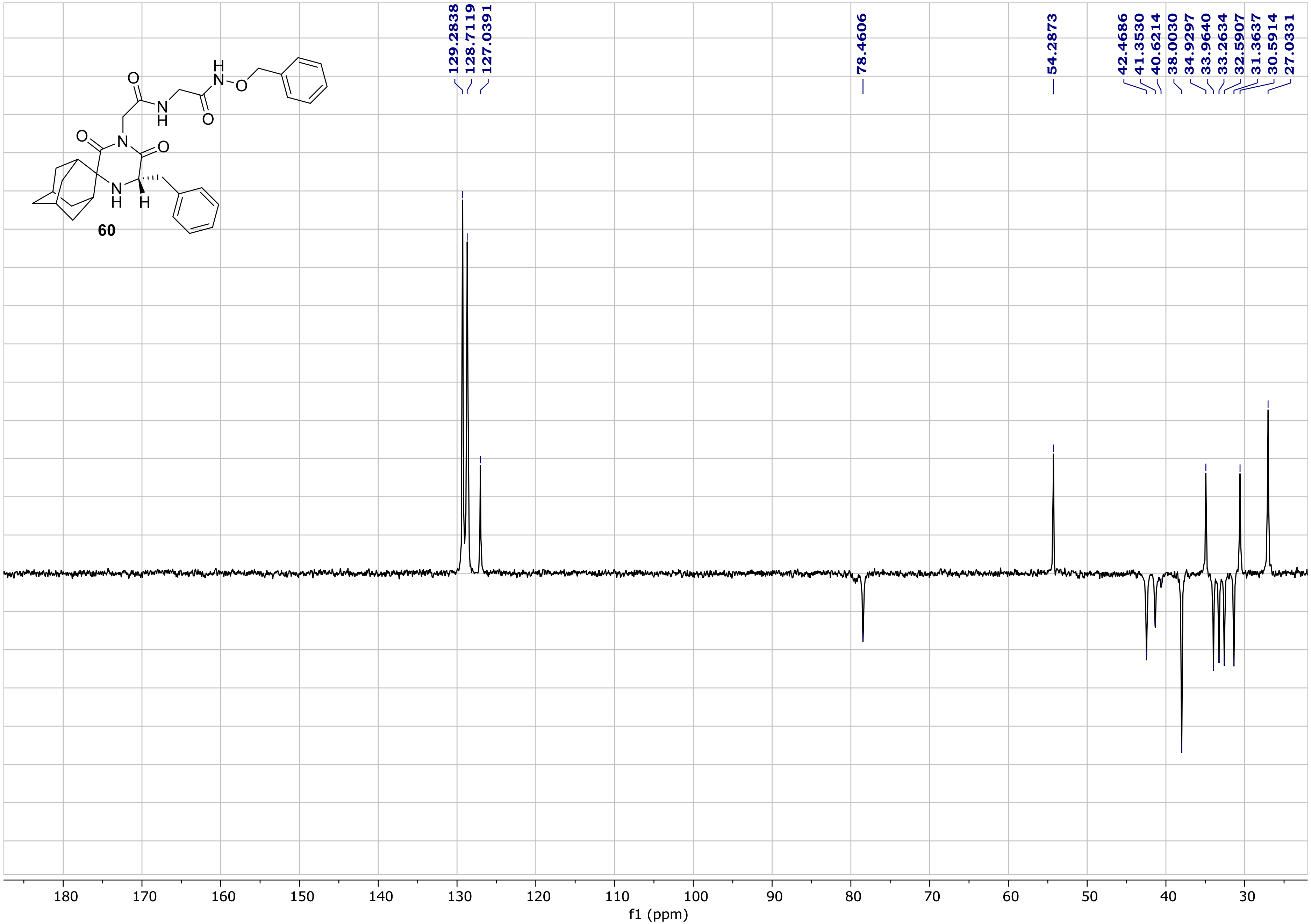

^{1}H NMR of **61** (400.11 MHz, $CDCl_3$)

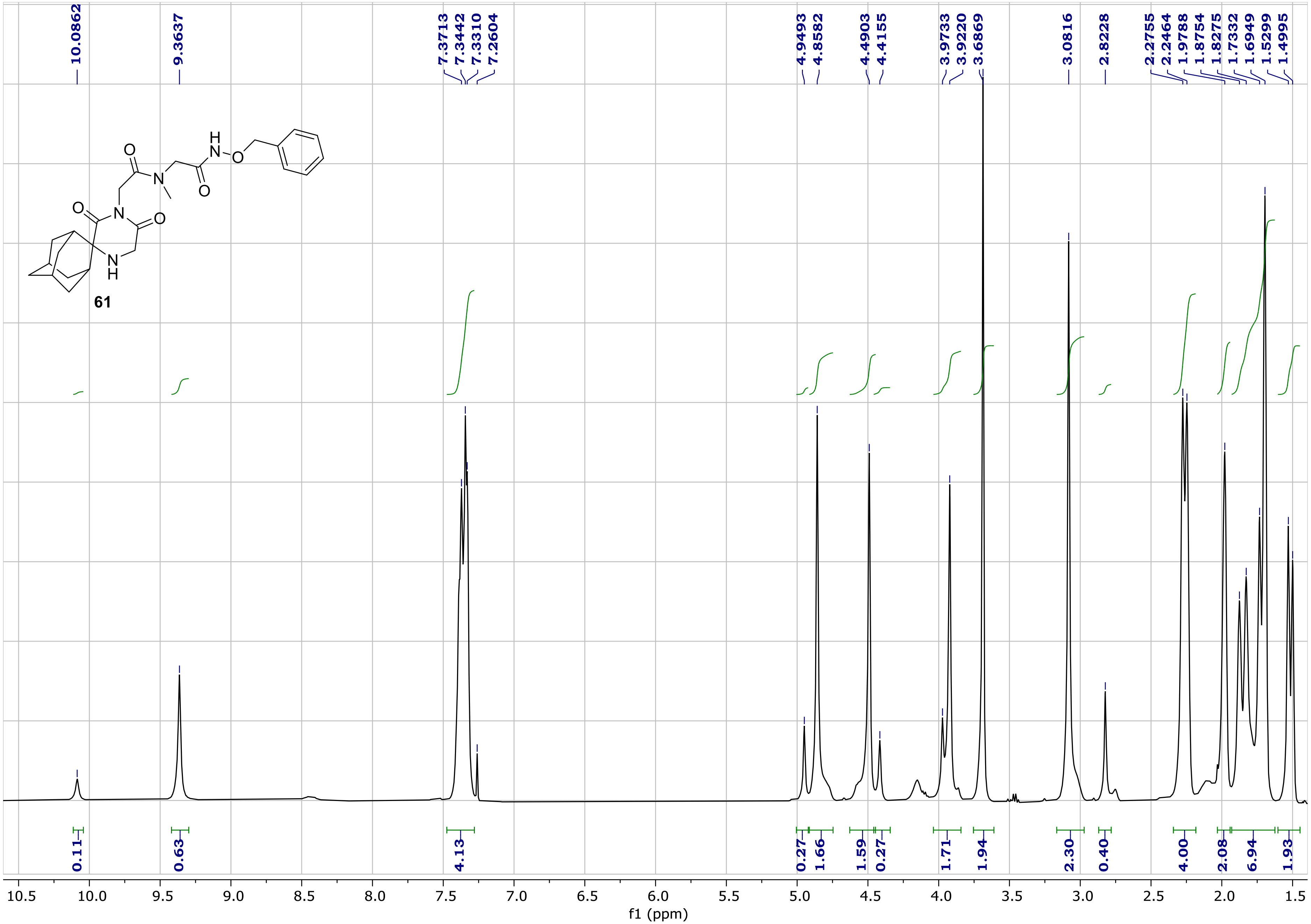

^{13}C NMR of **61** (50.32 MHz, $CDCl_3$)

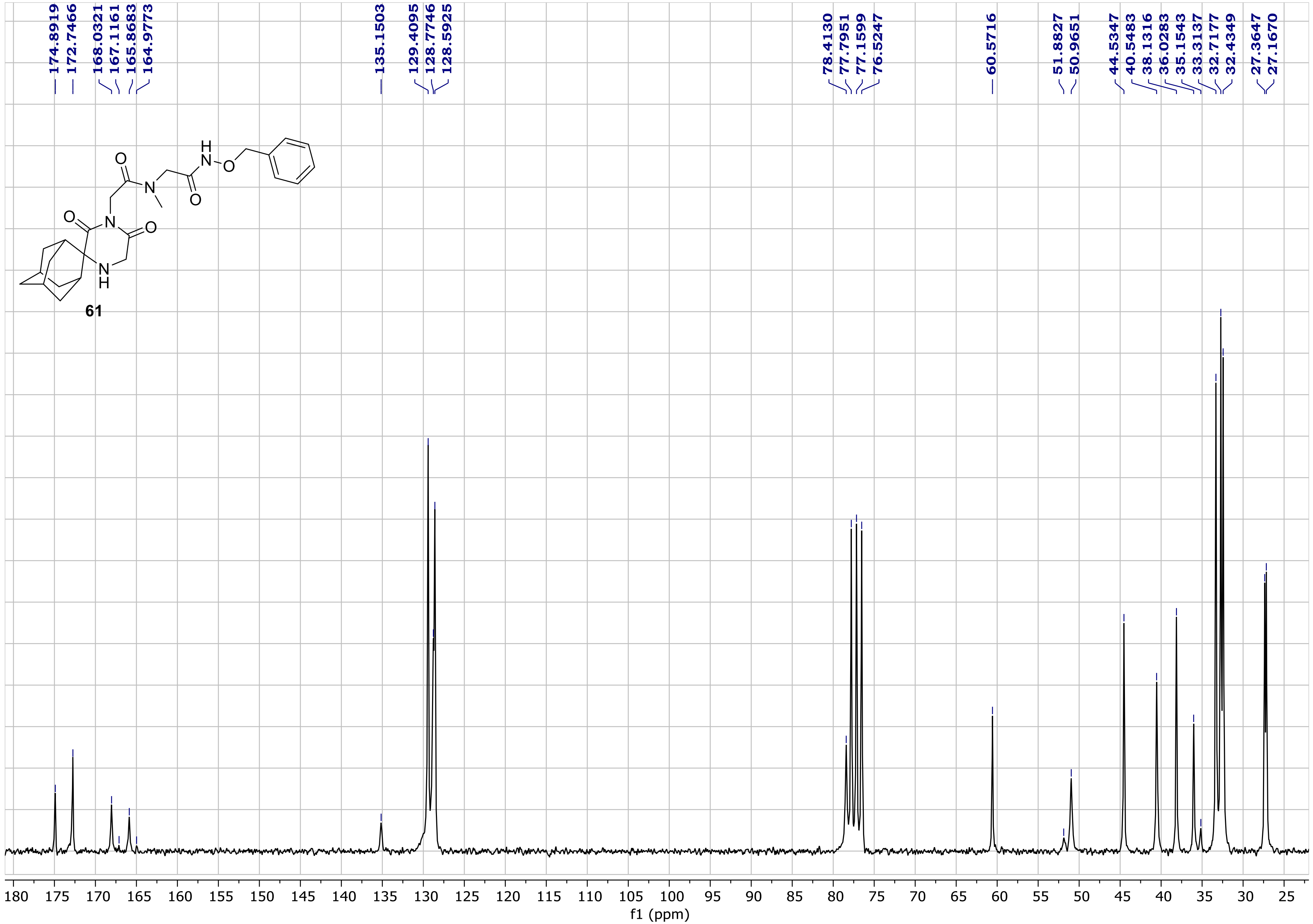

COSY NMR of **61** (400.11 MHz, $CDCl_3$)

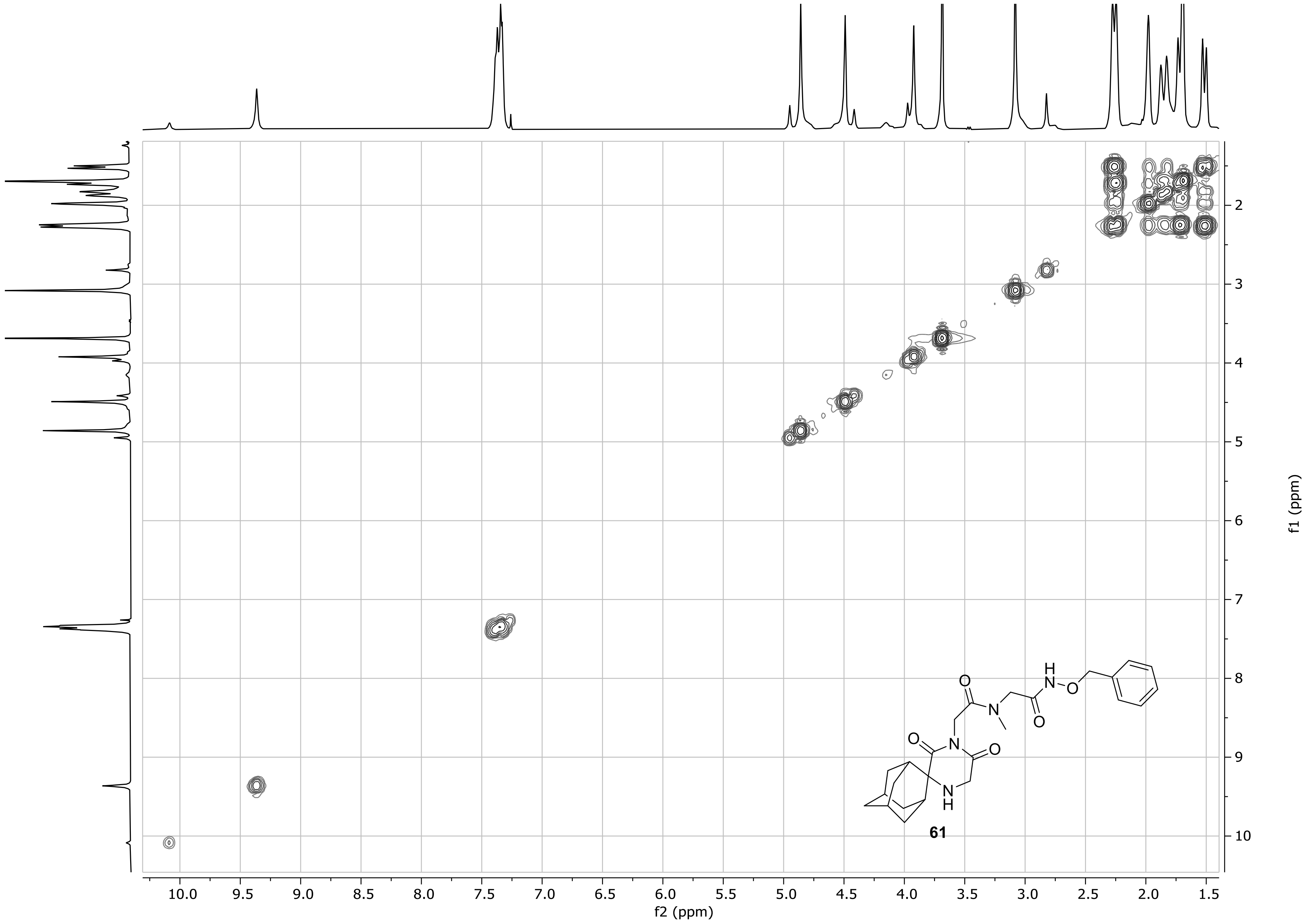

HSQC NMR of **61** (400.11 MHz, $CDCl_3$)

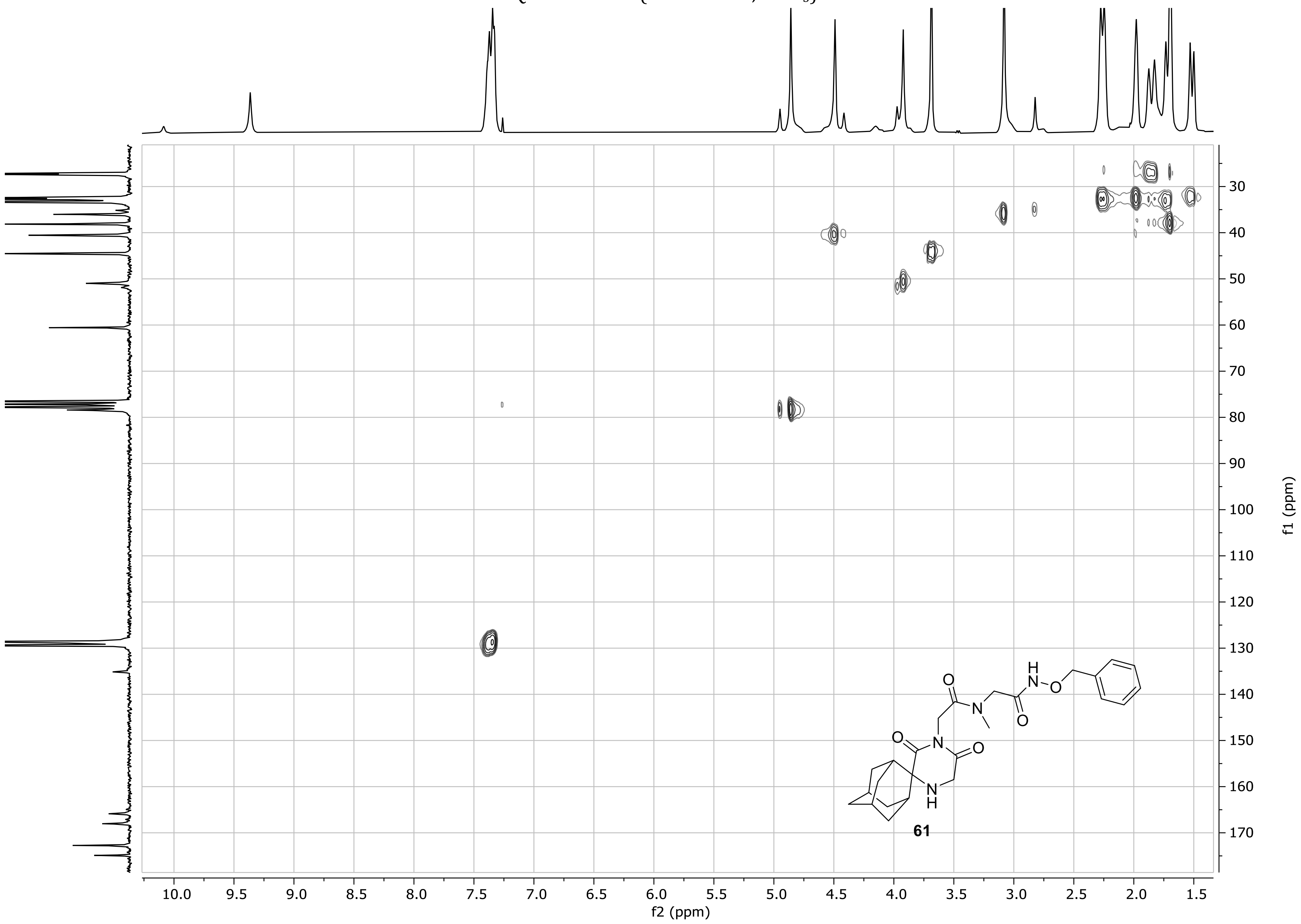

DEPT NMR of **61** (50.32 MHz, $CDCl_3$)

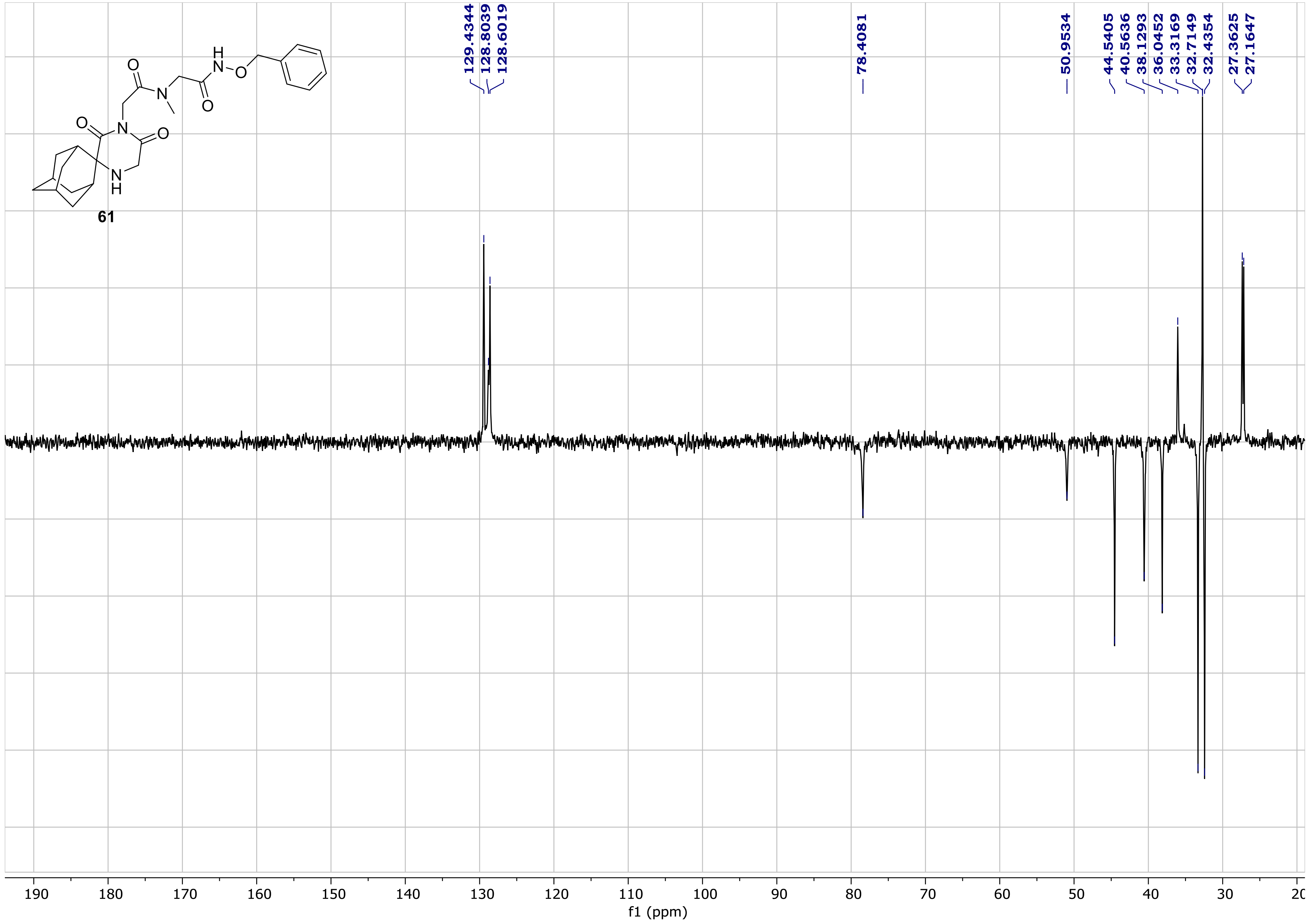

^1H NMR of **62** (600.11 MHz, $CDCl_3$)

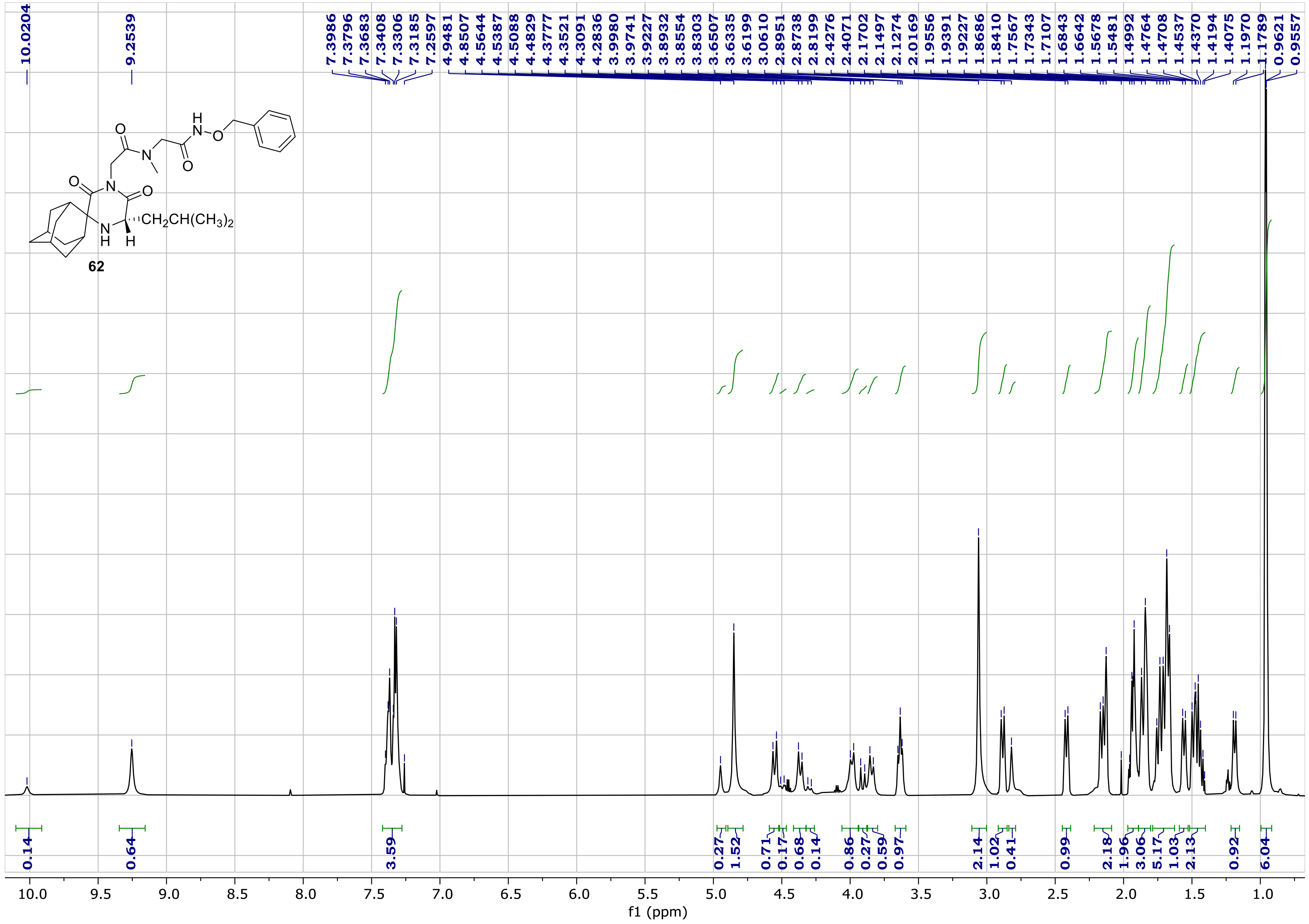

^{13}C NMR of **62** (150.9 MHz, $CDCl_3$)

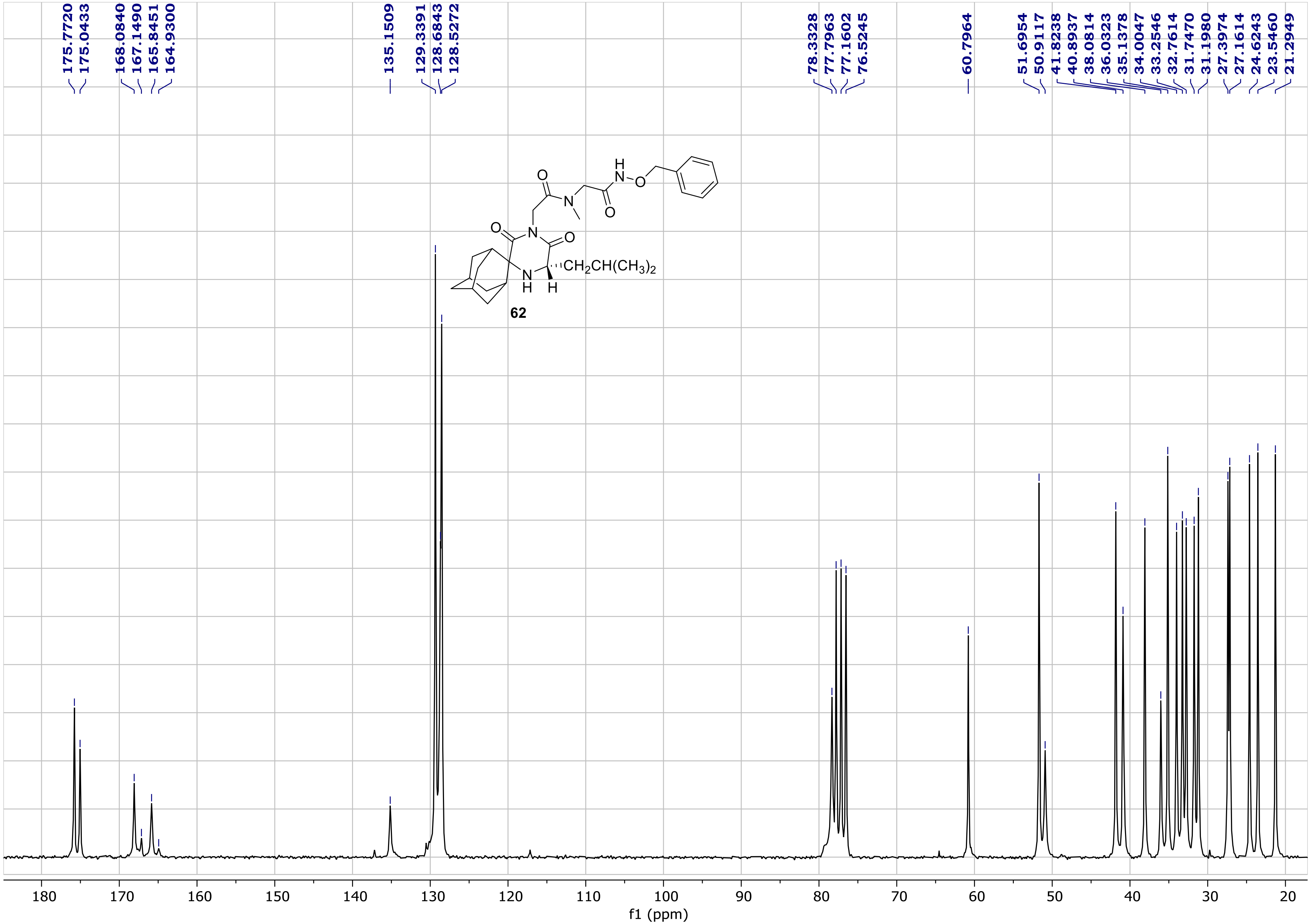

COSY NMR of **62** (600.11 MHz, $CDCl_3$)

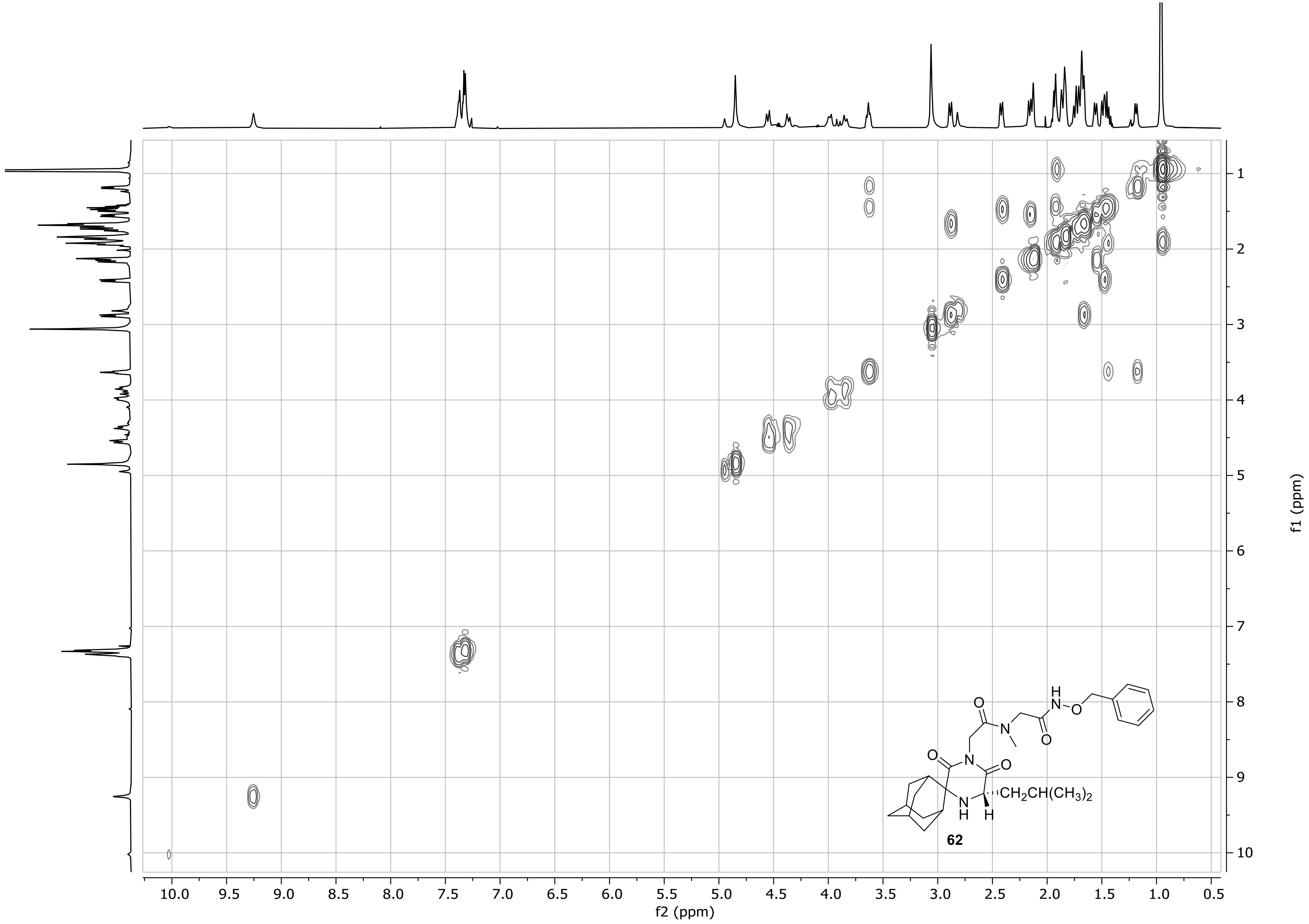

HSQC-DEPT NMR of **62** (600.11 MHz, $CDCl_3$)

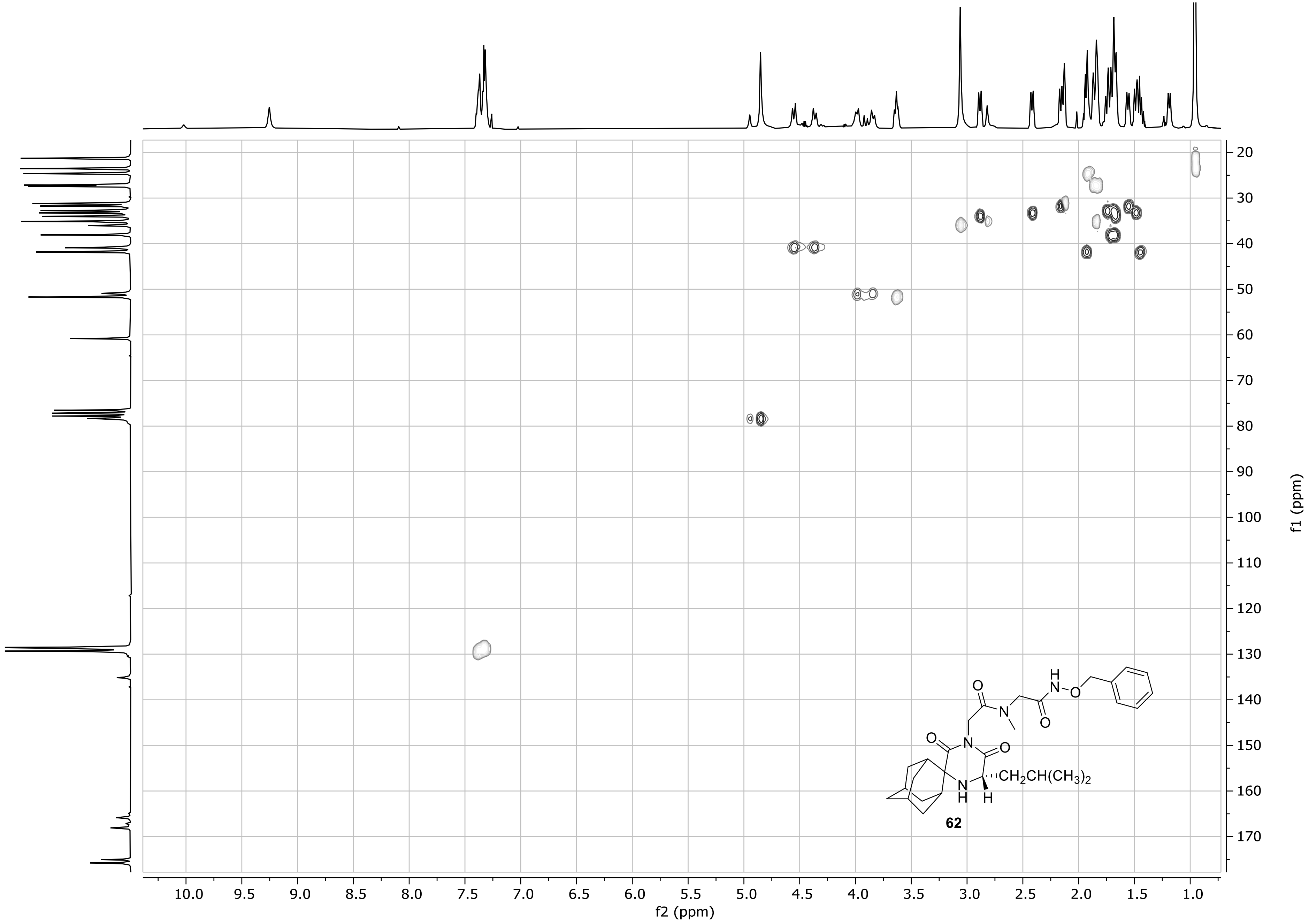

DEPT NMR of **62** (150.9 MHz, $CDCl_3$)

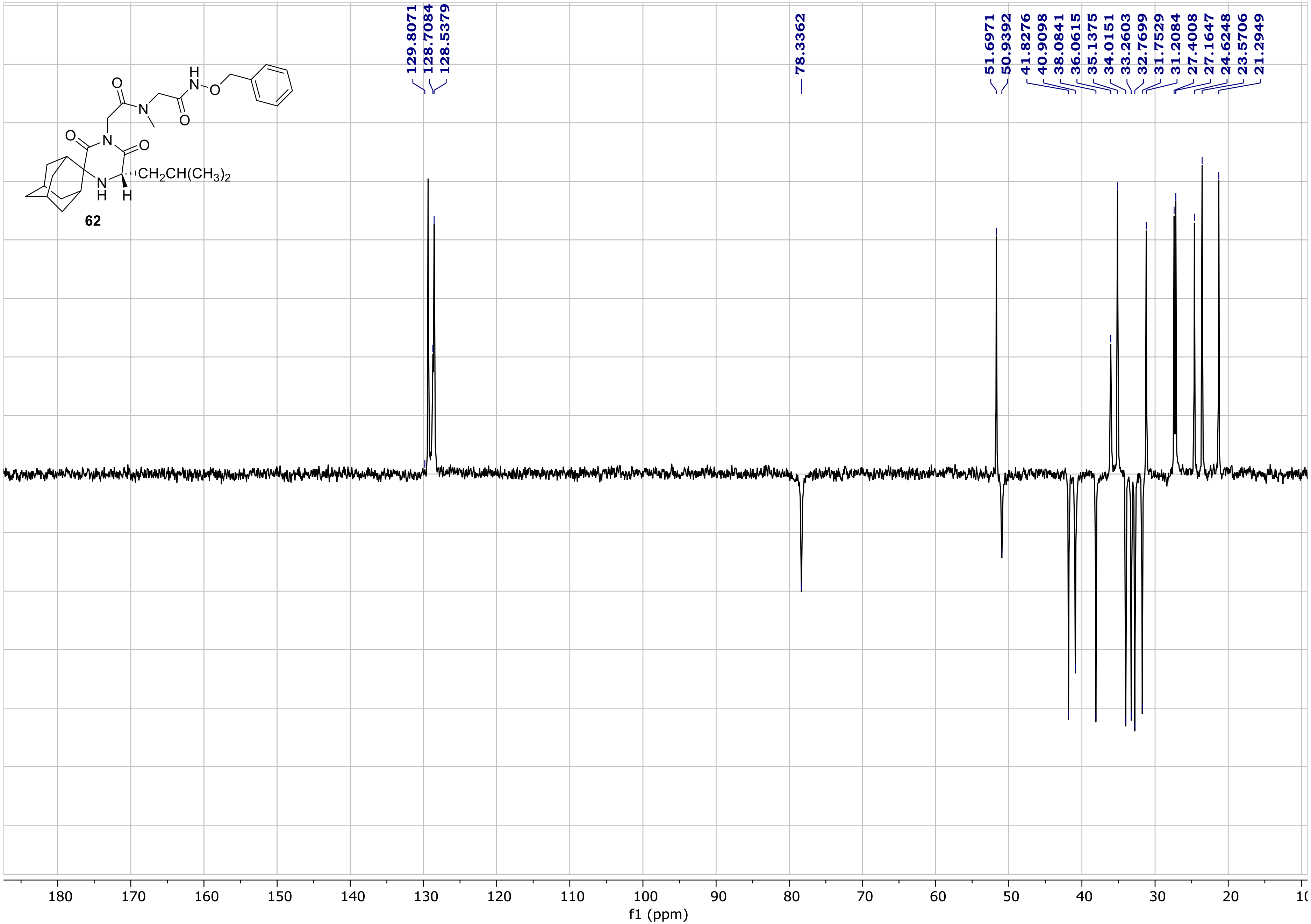

^{1}H NMR of **63** (600.11 MHz, $CDCl_3$)

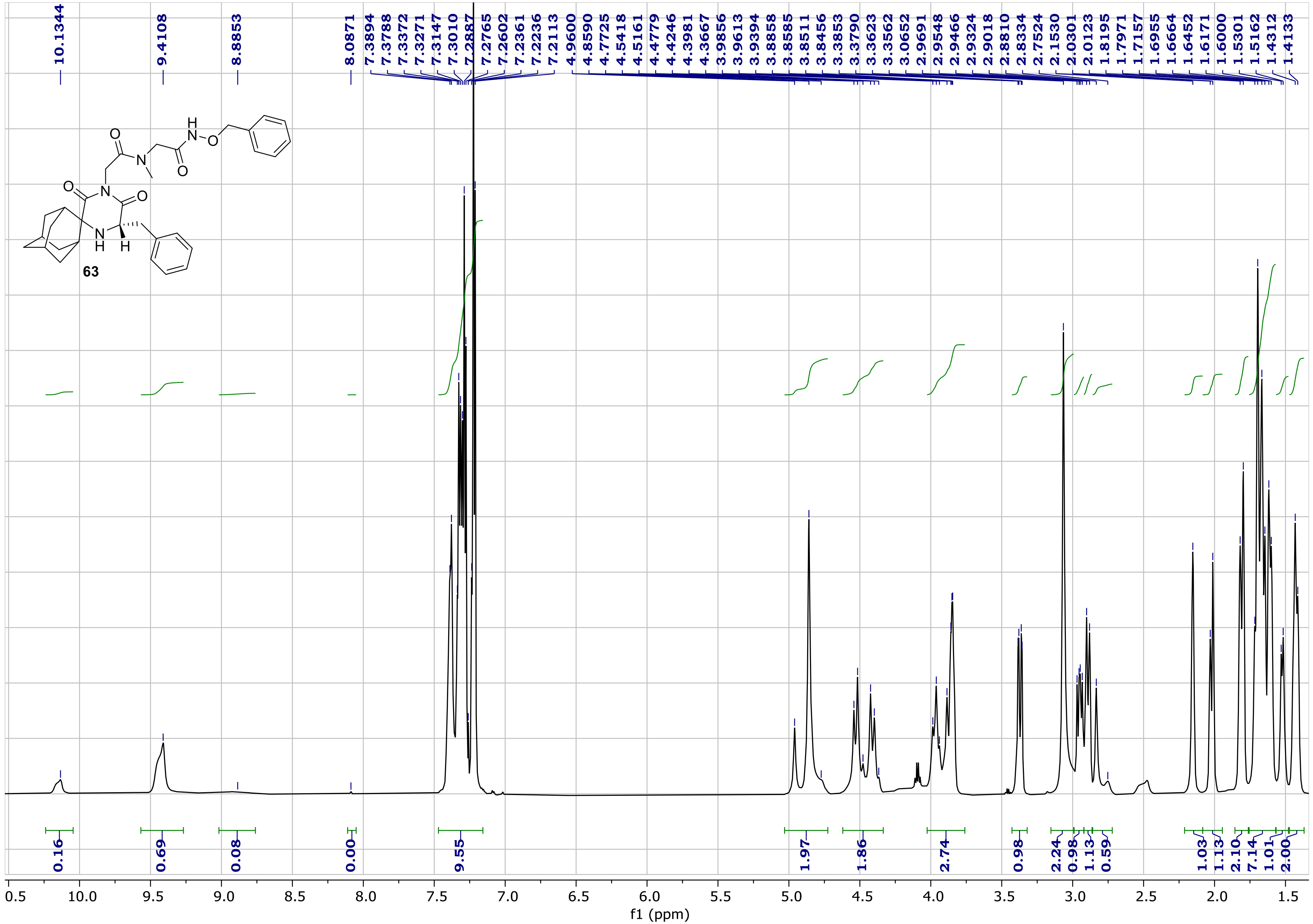

^{13}C NMR of **63** (150.9 MHz, $CDCl_3$)

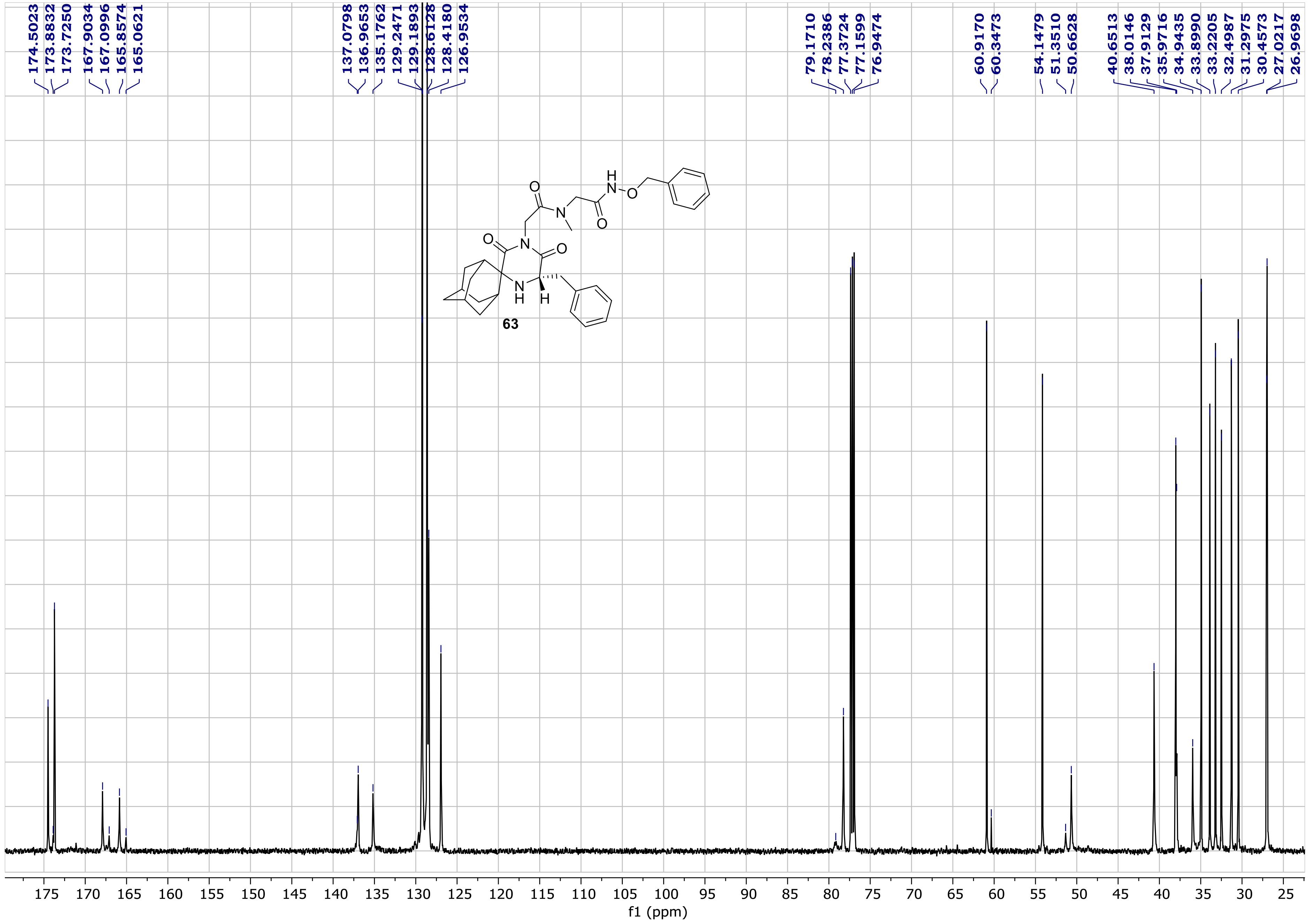

COSY NMR of **63** (600.11 MHz, $CDCl_3$)

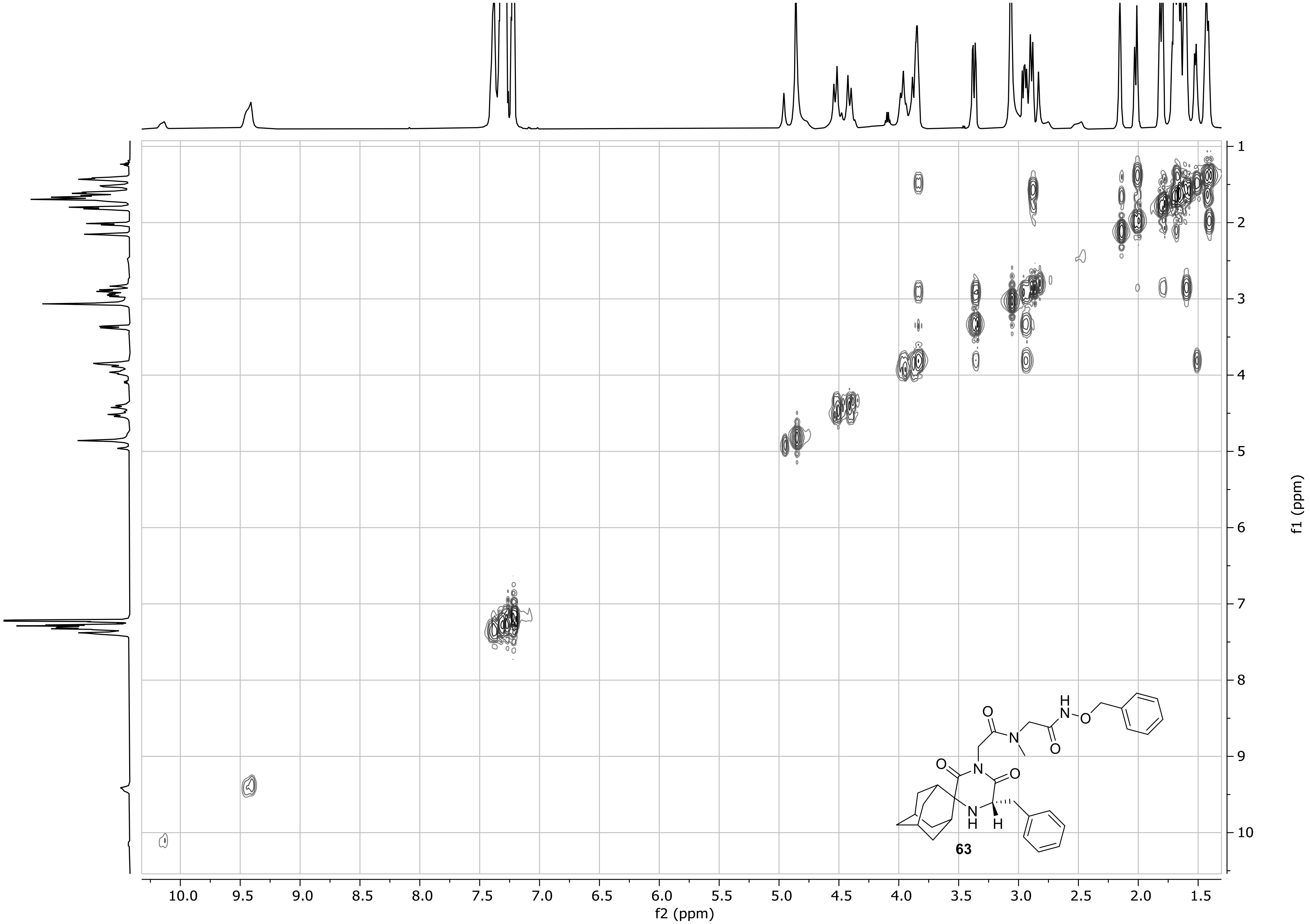

HSQC-DEPT NMR of **63** (600.11 MHz, $CDCl_3$)

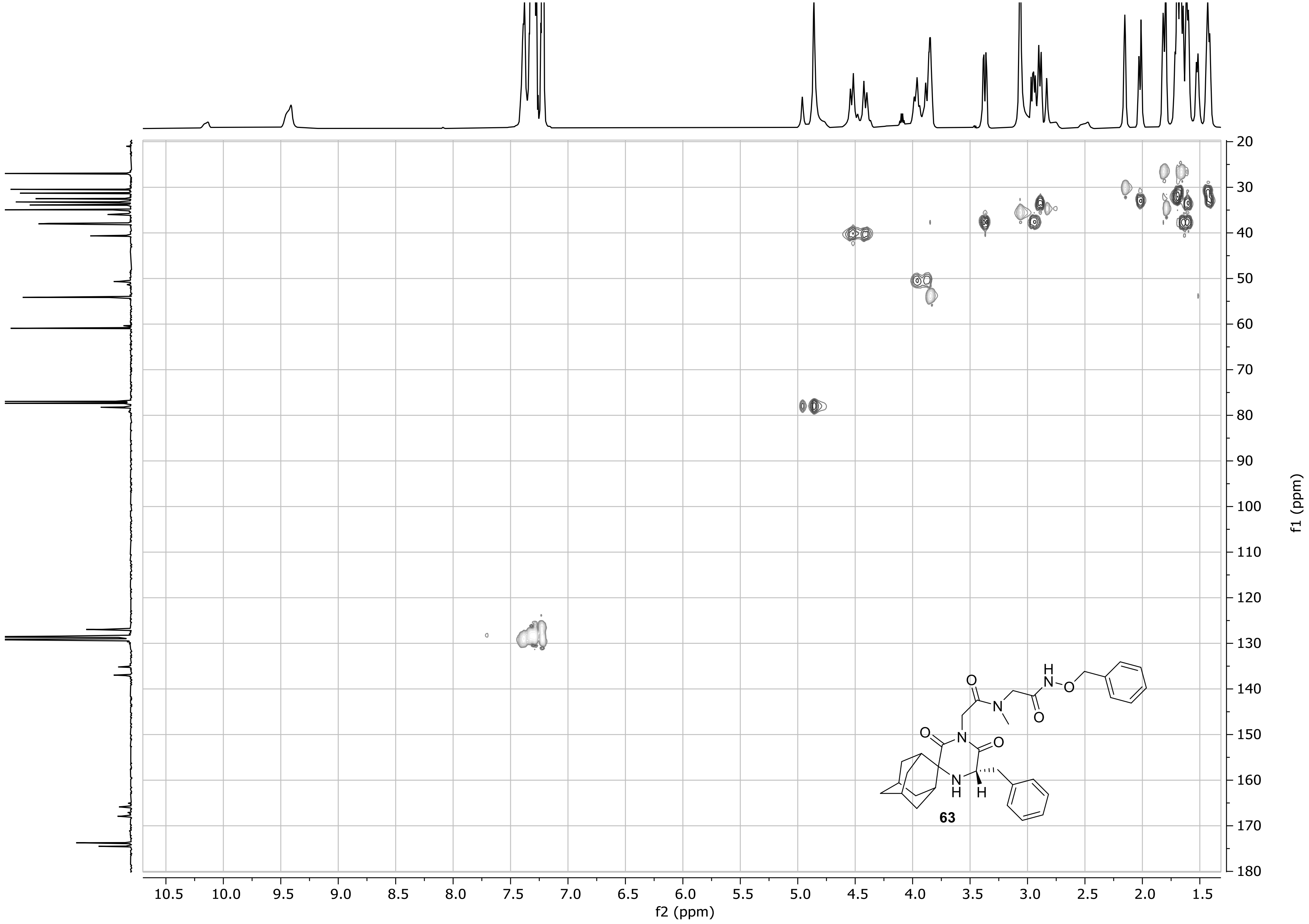

^{1}H NMR of **64** (400.11 MHz, DMSO-d_6)

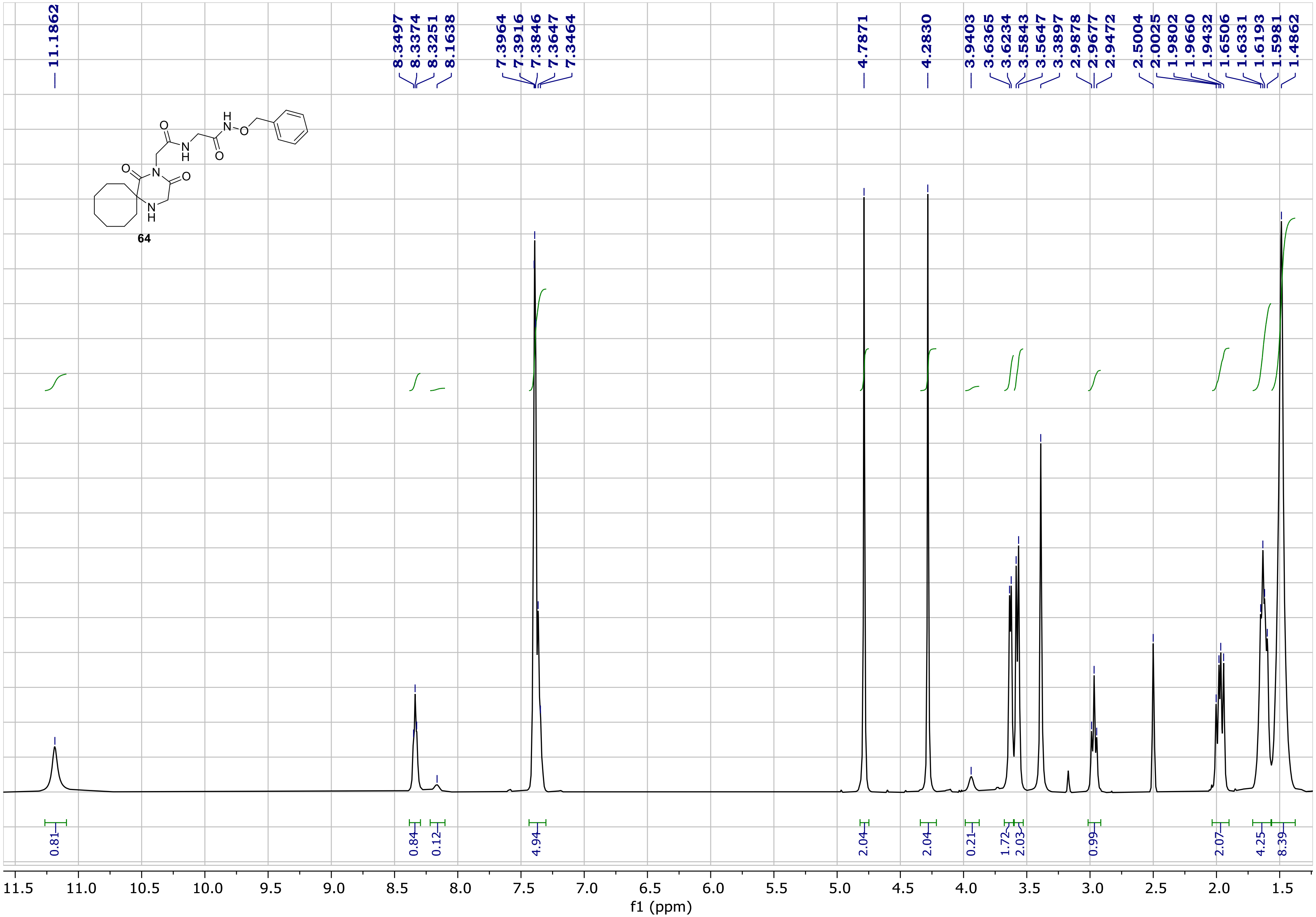

^{13}C NMR of **64** (50.32 MHz, DMSO-d_6)

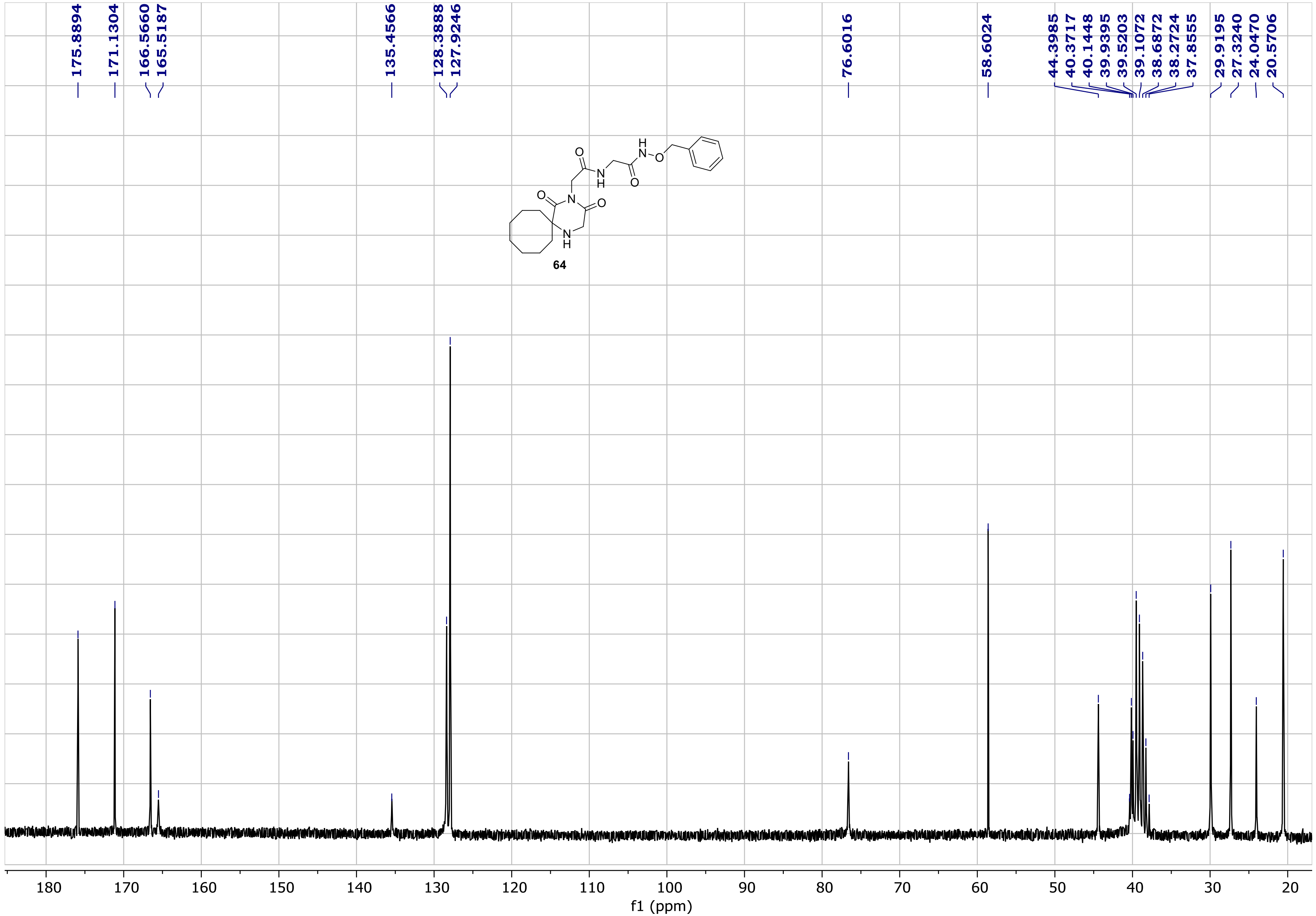

COSY NMR of **64** (400.11 MHz, DMSO-d_6)

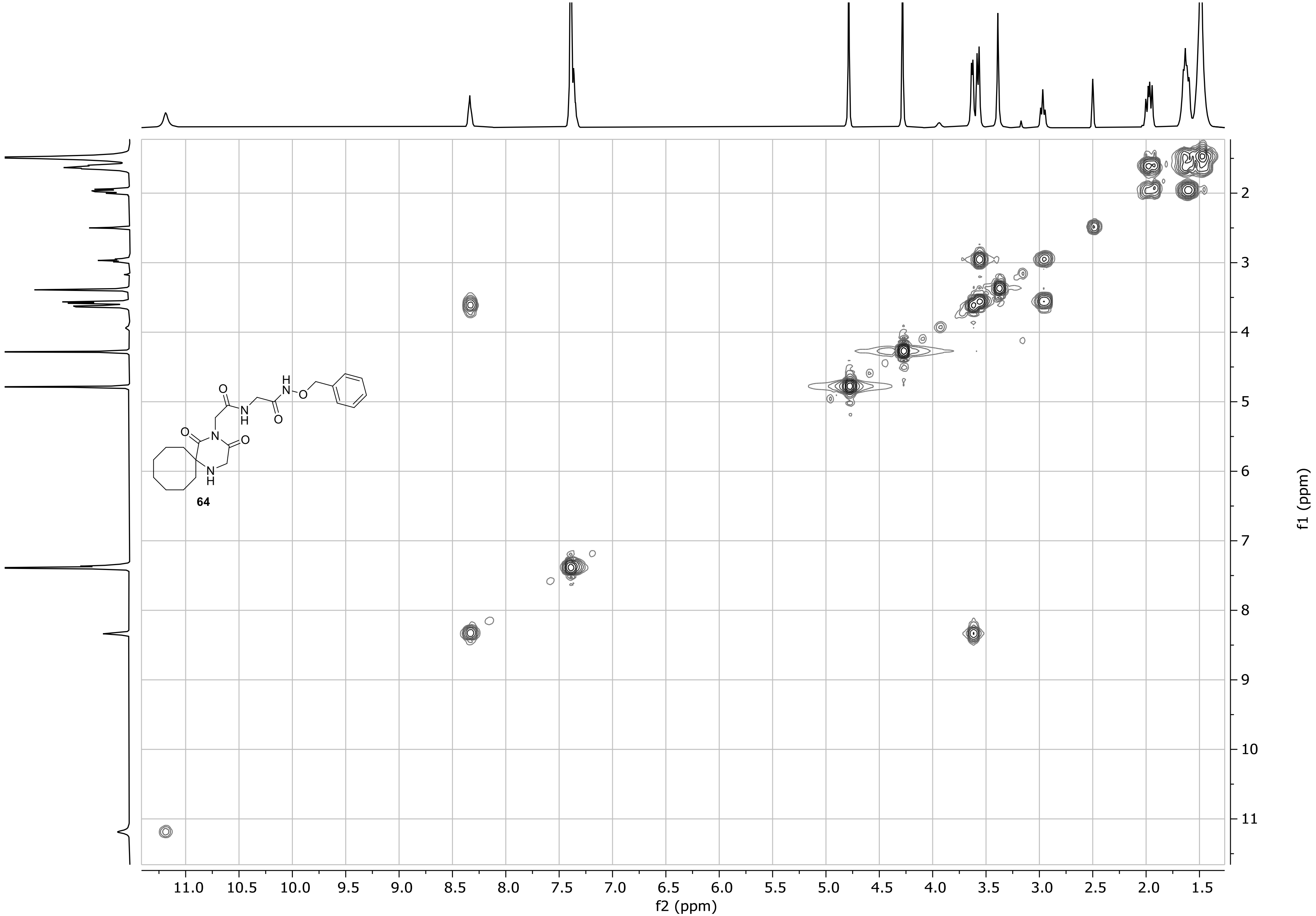

HSQC NMR of **64** (400.11 MHz, DMSO-d_6)

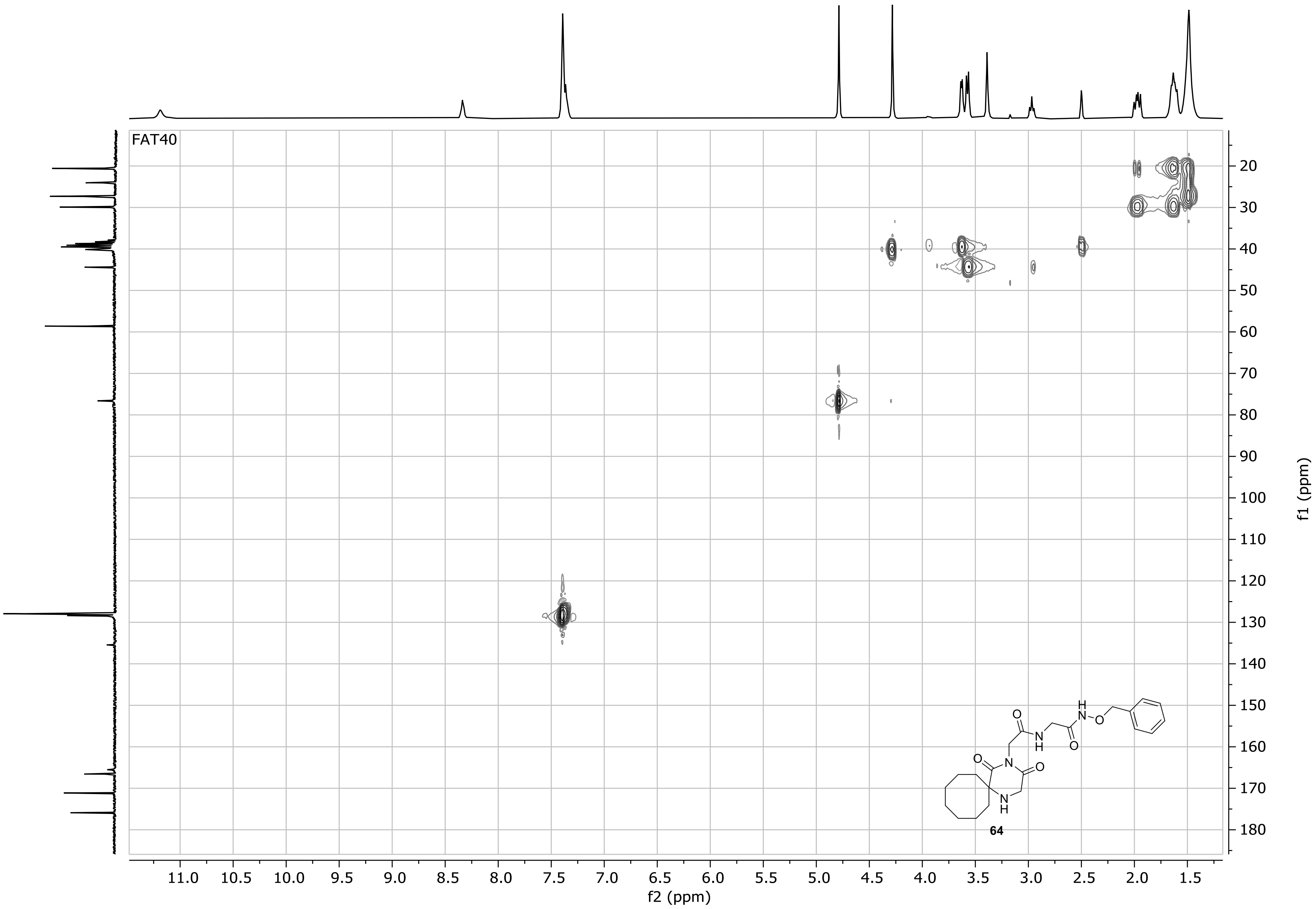

DEPT NMR of **64** (50.32 MHz, DMSO-d_6)

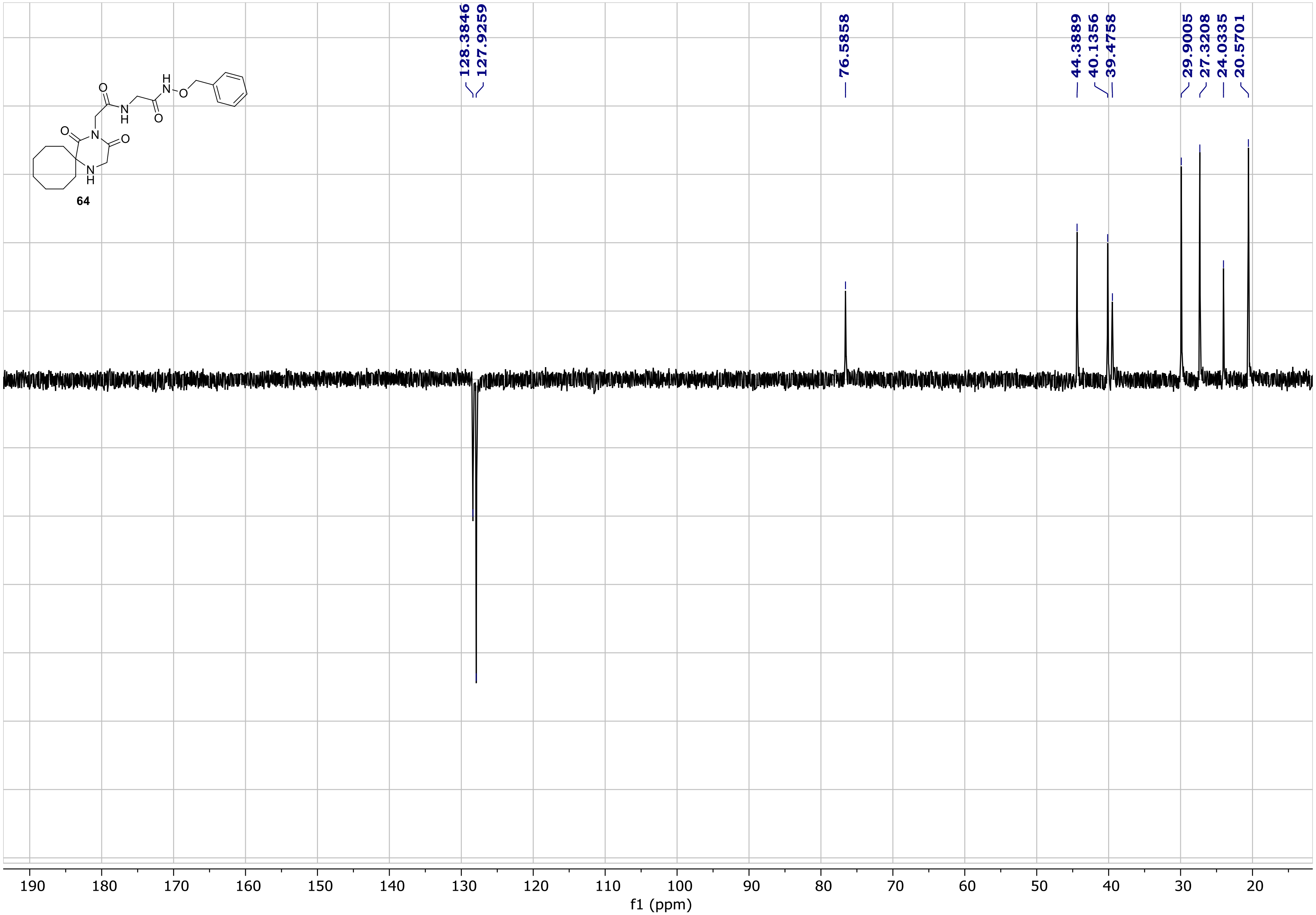

^{1}H NMR of **65** (400.11 MHz, DMSO-d_6)

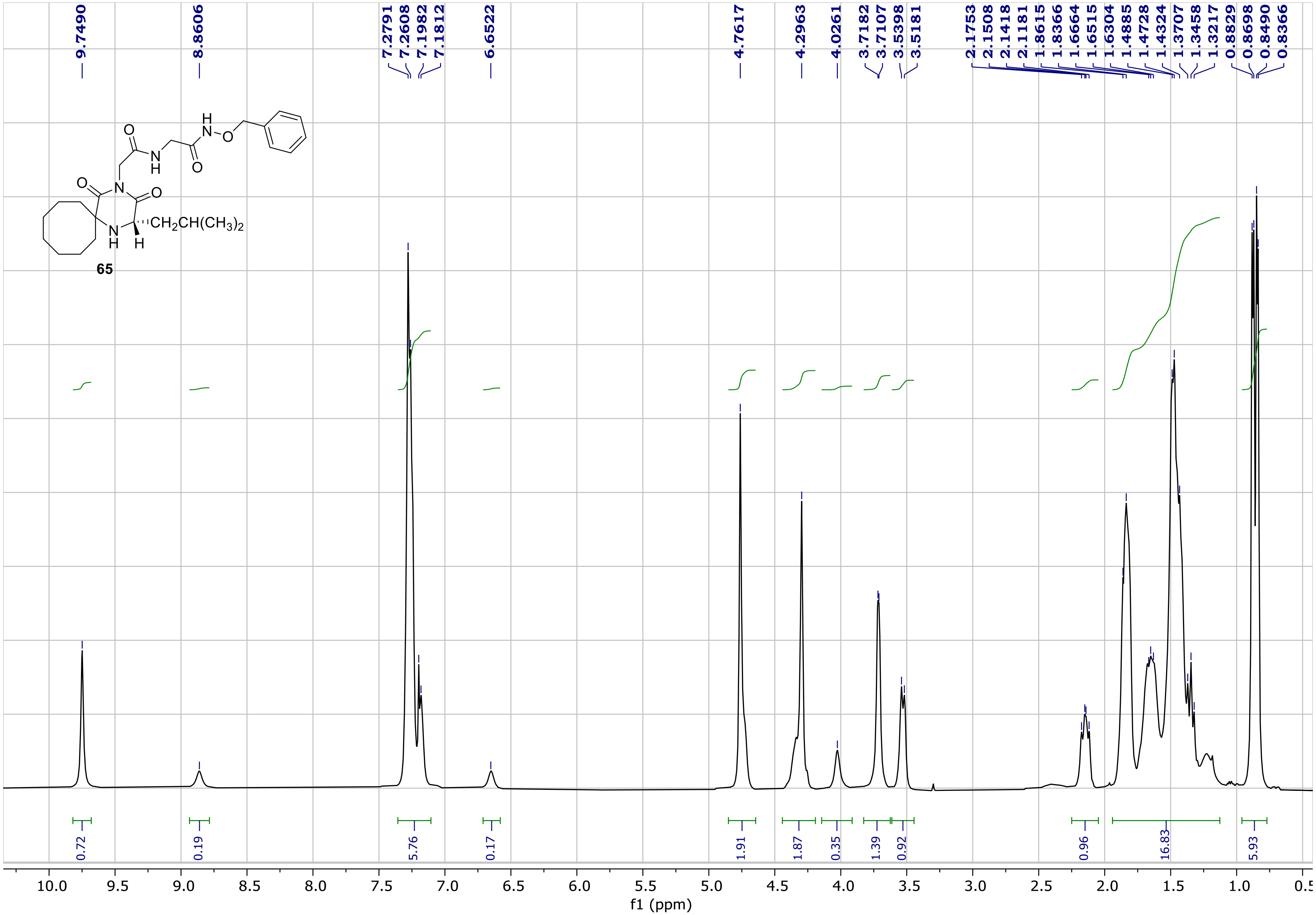

^{13}C NMR of **65** (50.32 MHz, DMSO-d_6)

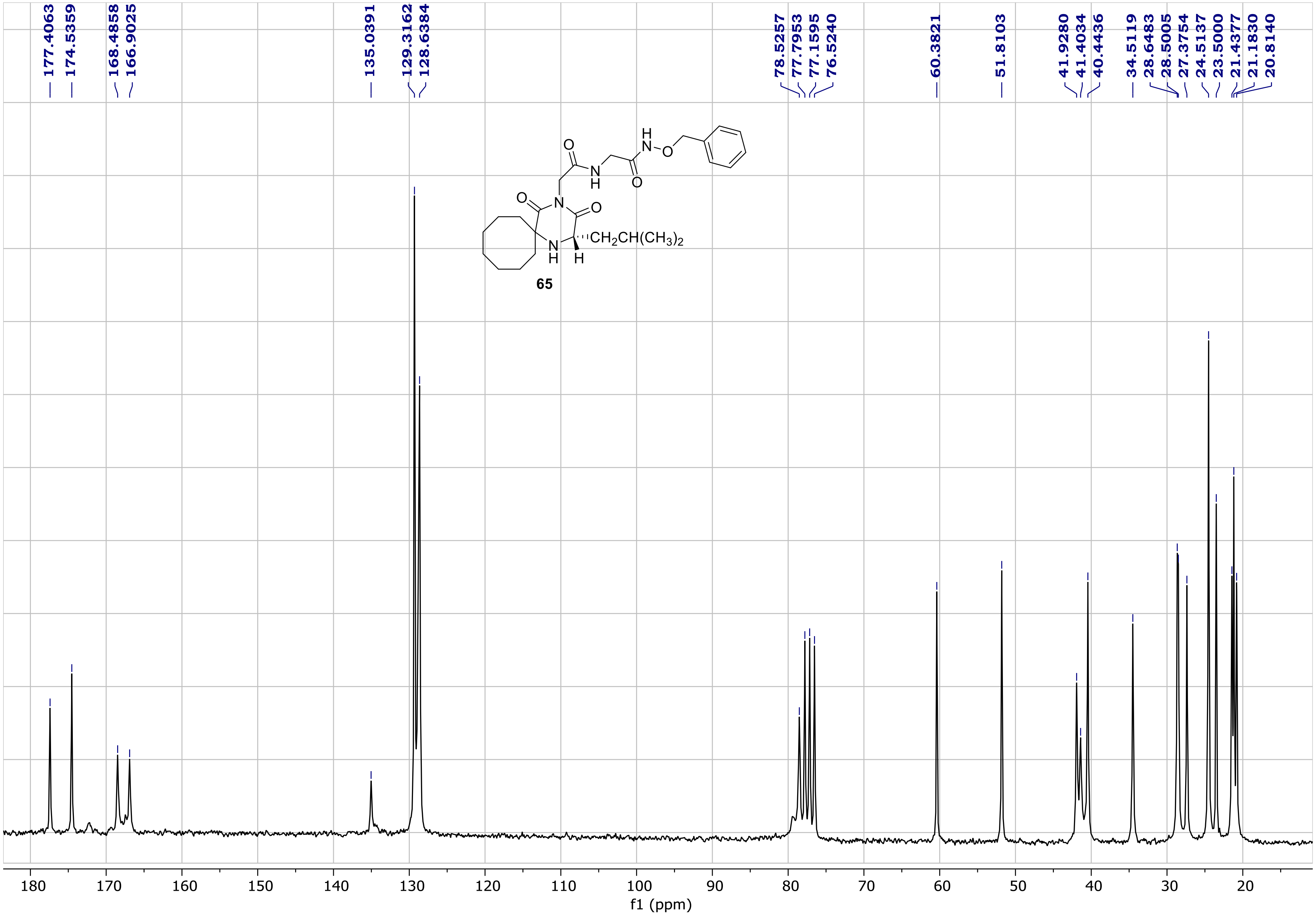

COSY NMR of **65** (400.11 MHz, DMSO-d_6)

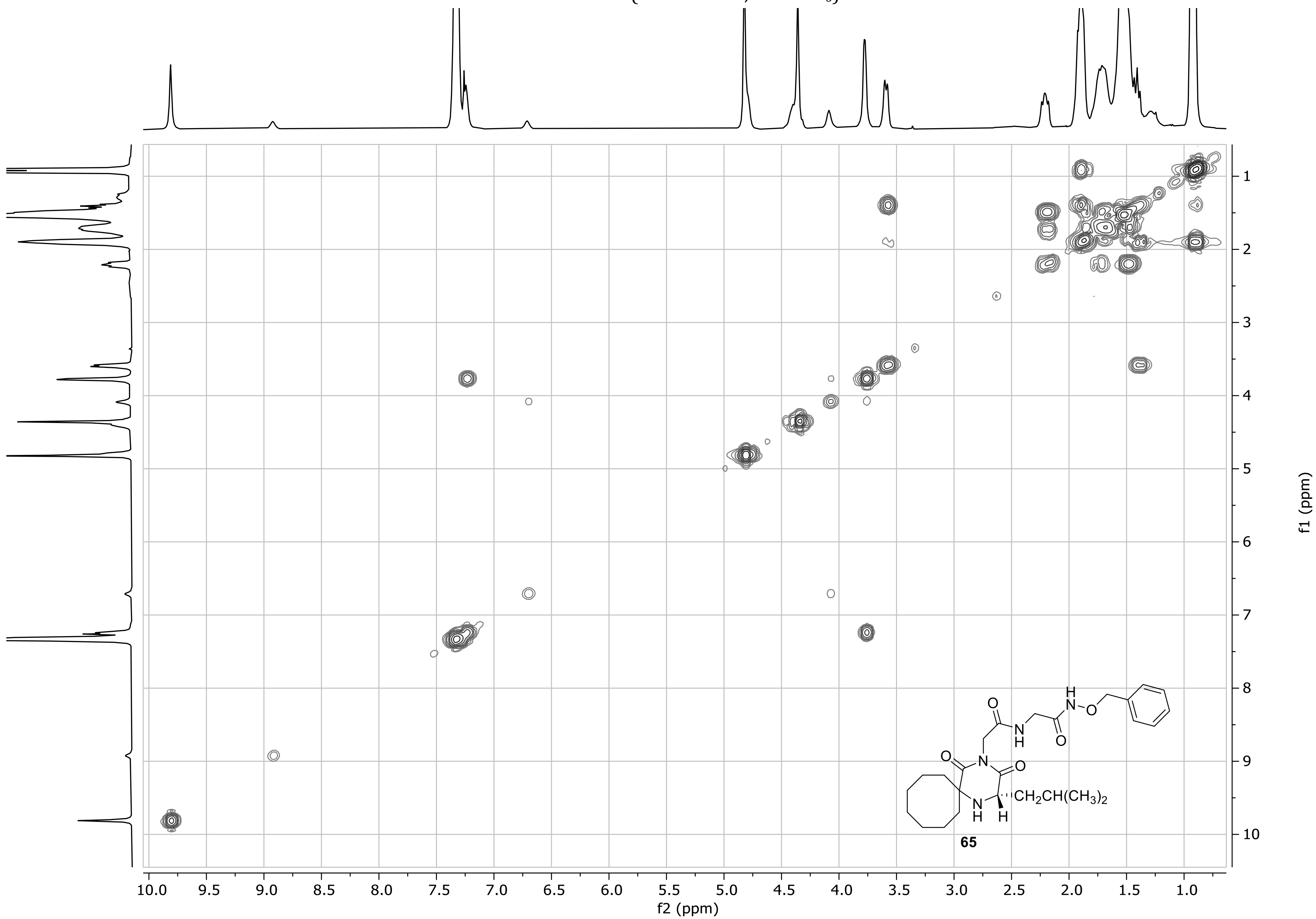

65

HSQC NMR of **65** (400.11 MHz, DMSO-d_6)

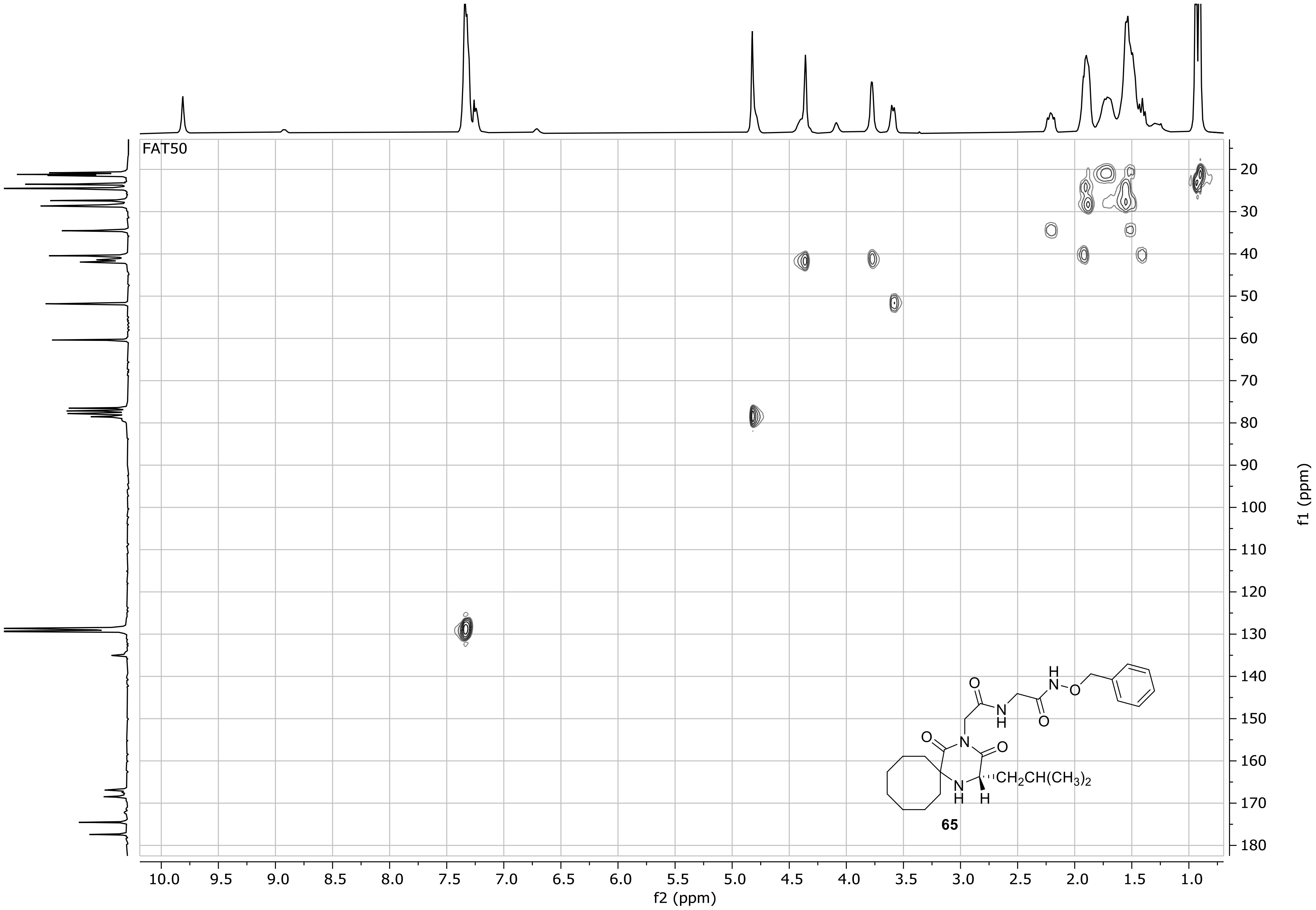

DEPT NMR of **65** (50.32 MHz, DMSO-d_6)

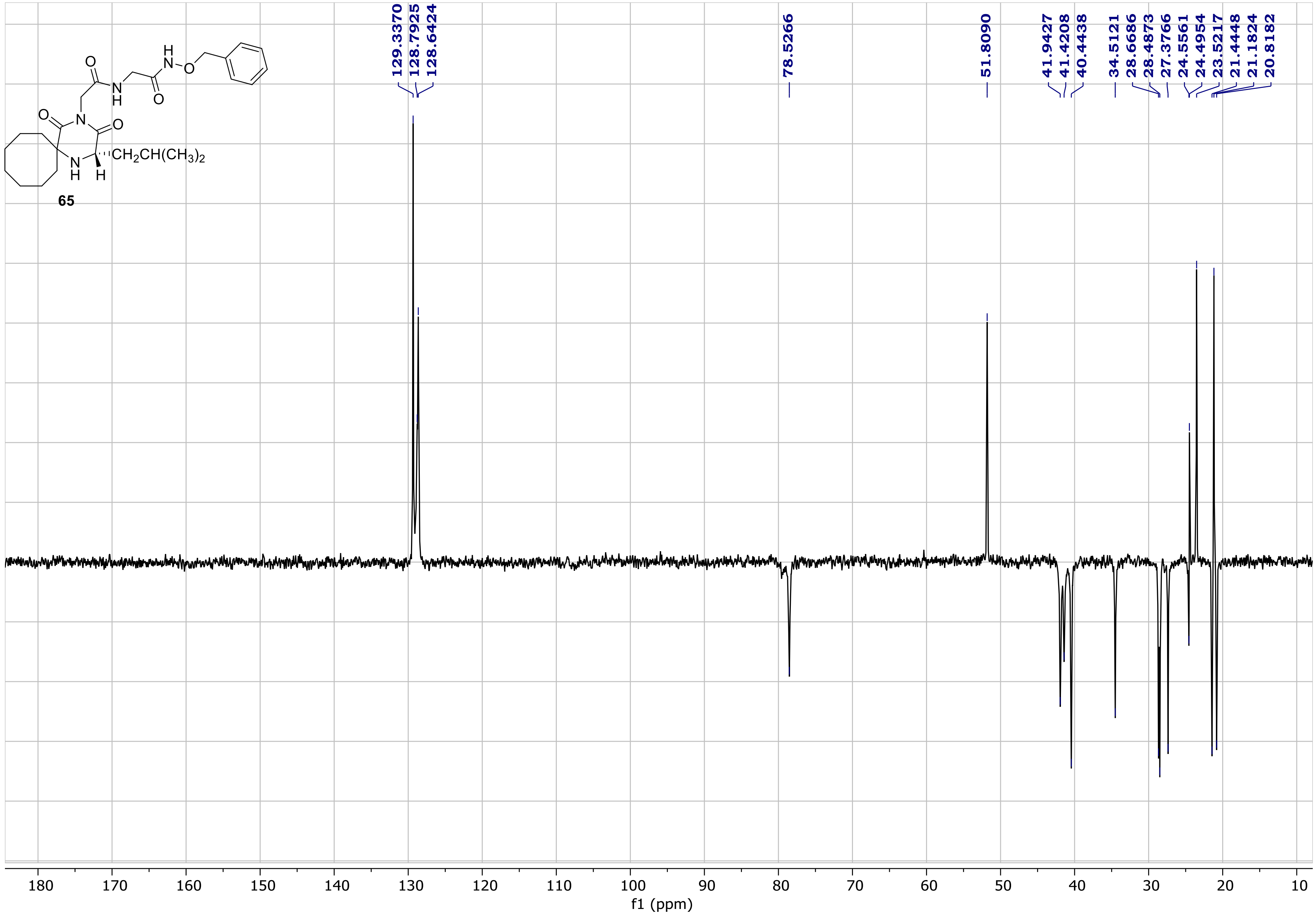

^{1}H NMR of **66** (600.11 MHz, $CDCl_3$)

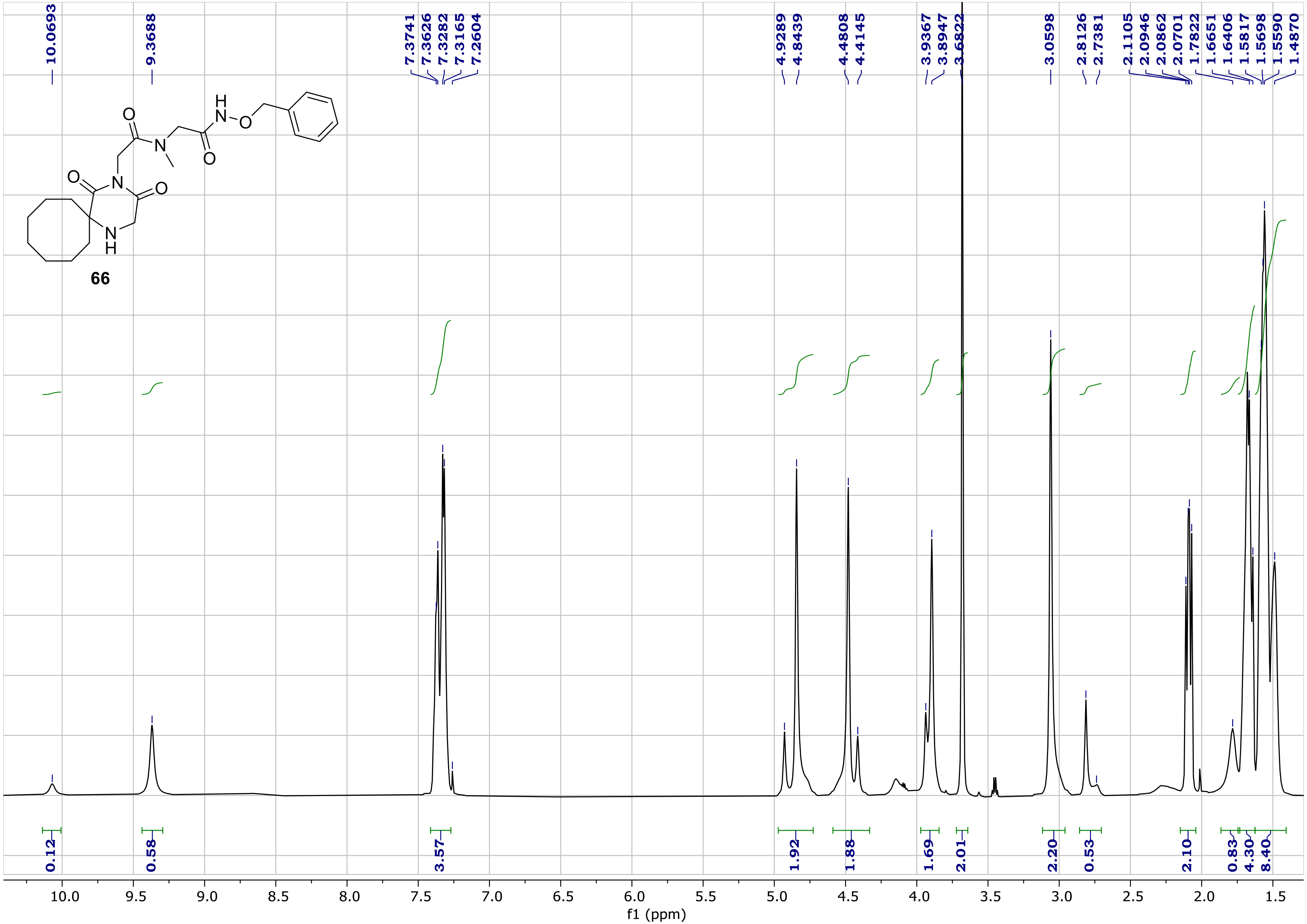

^{13}C NMR of **66** (50.32 MHz, $CDCl_3$)

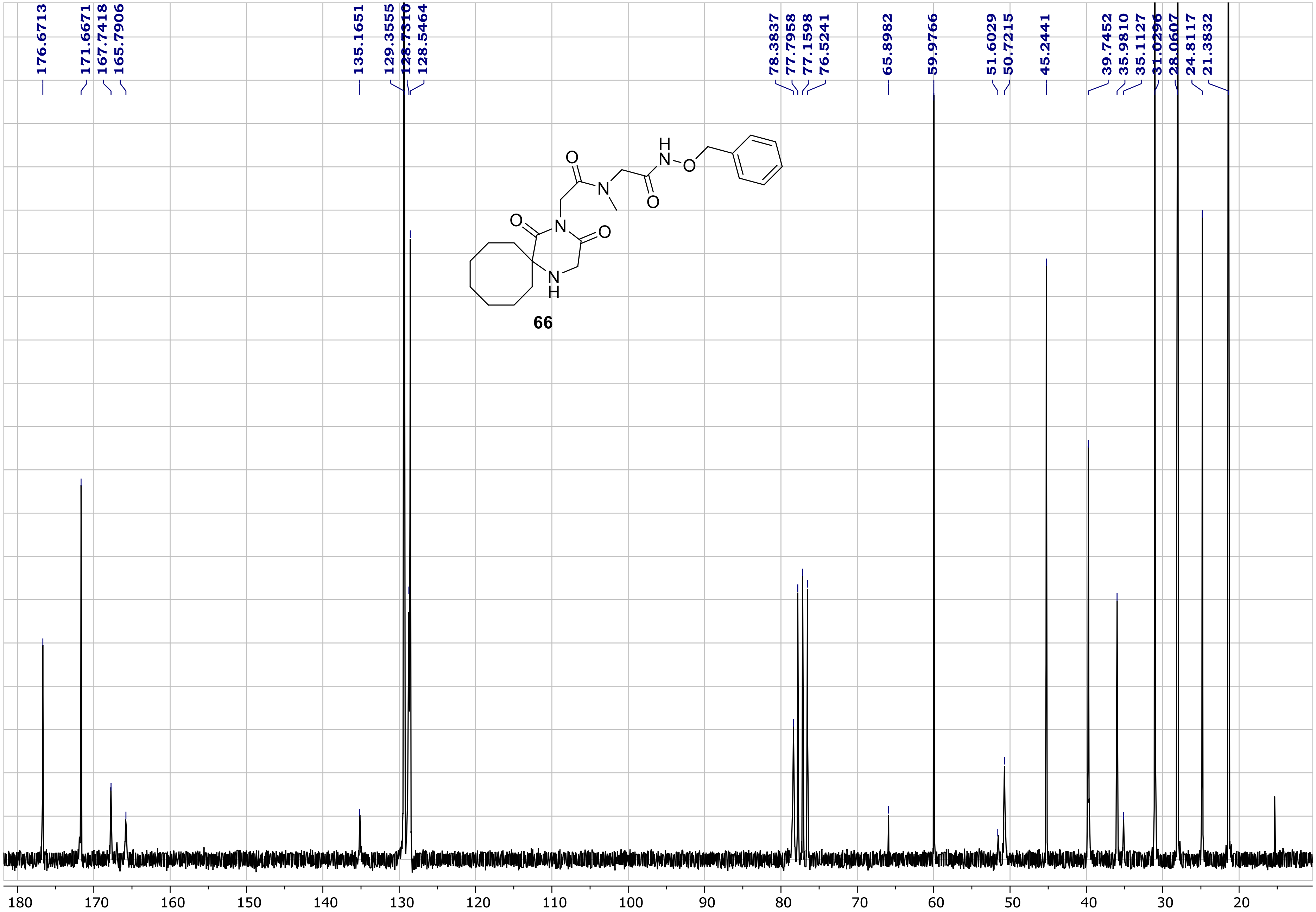

COSY NMR of **66** (600.11 MHz, $CDCl_3$)

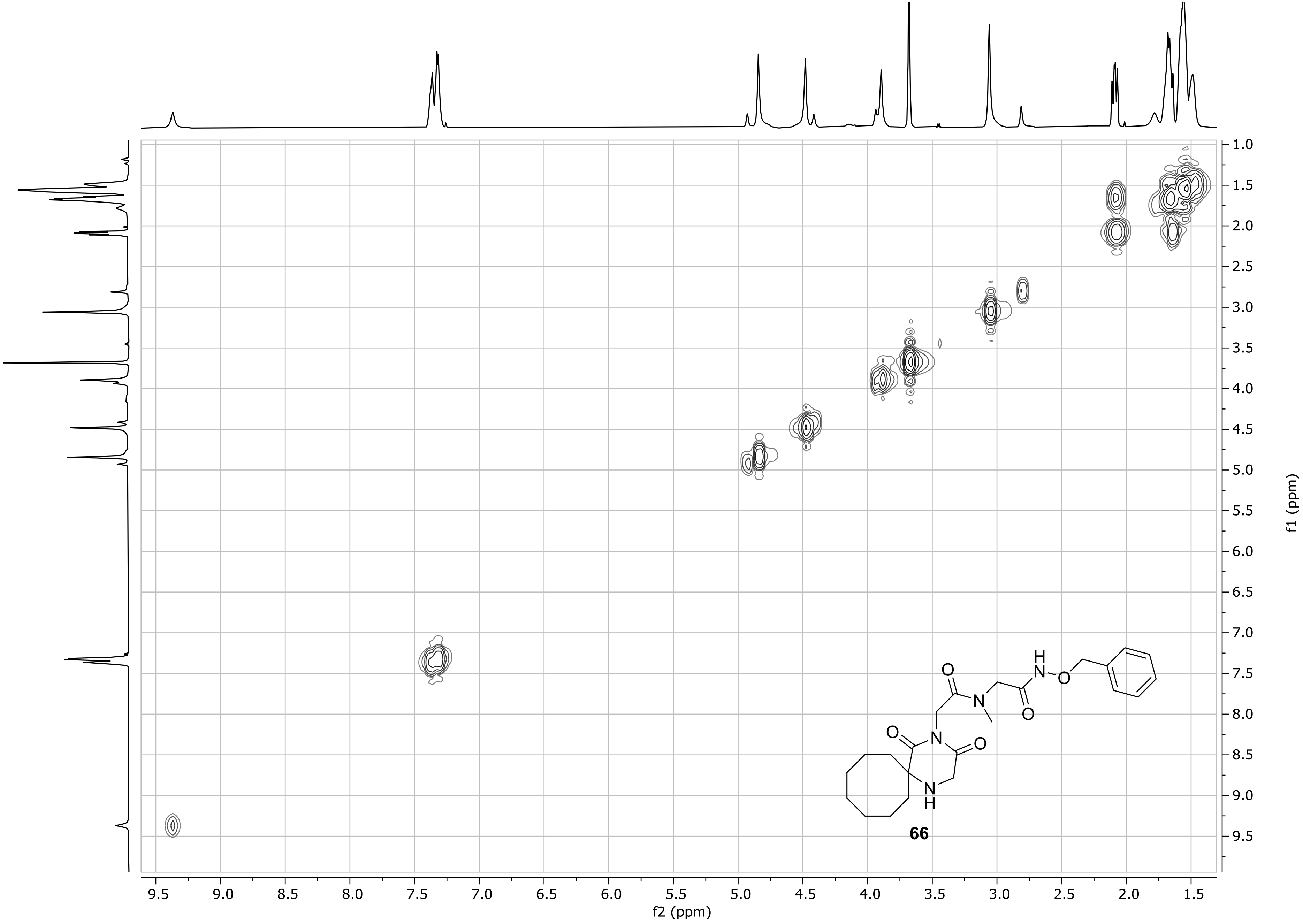

HSQC-DEPT NMR of **66** (600.11 MHz, $CDCl_3$)

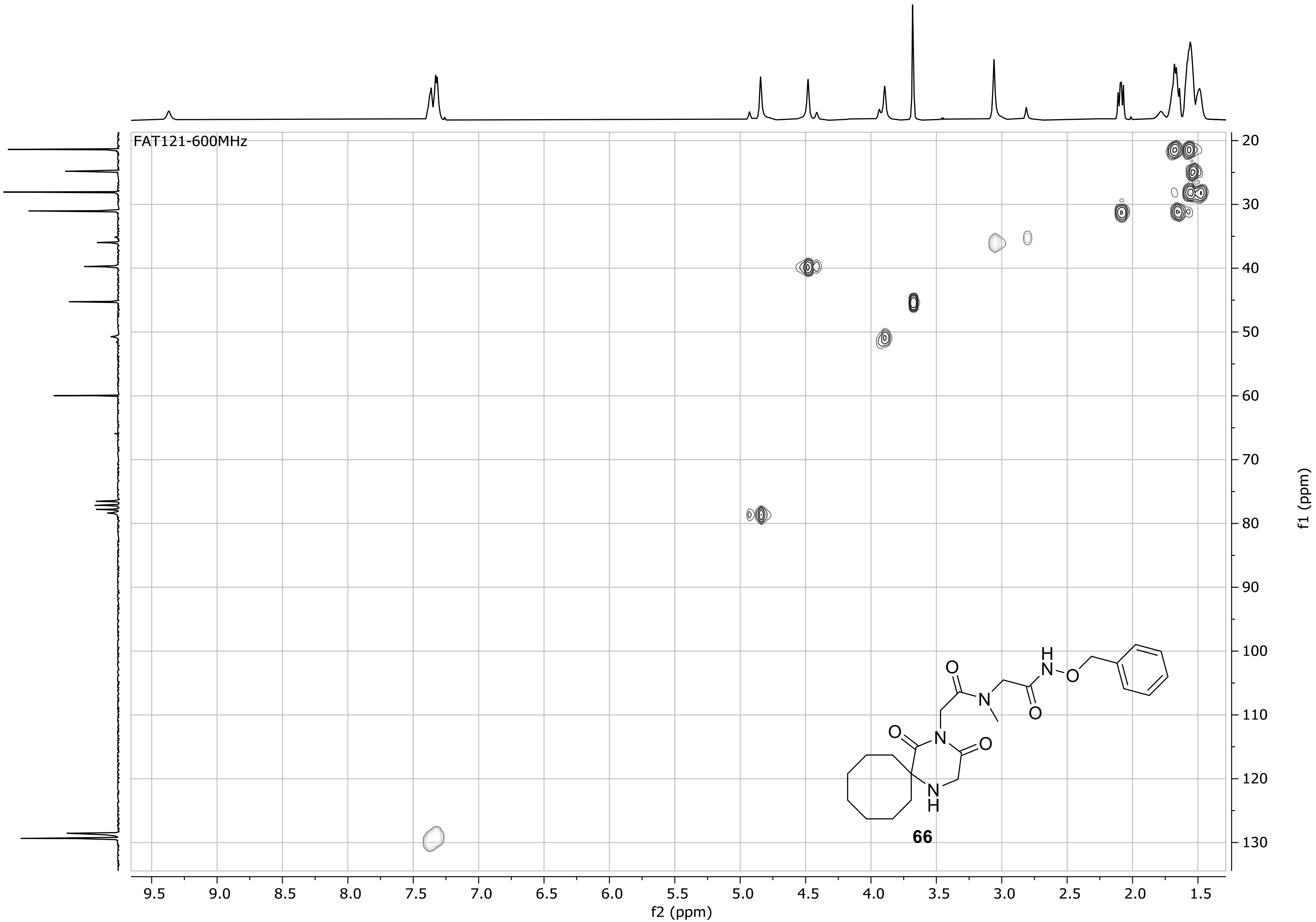

DEPT NMR of **66** (50.32 MHz, $CDCl_3$)

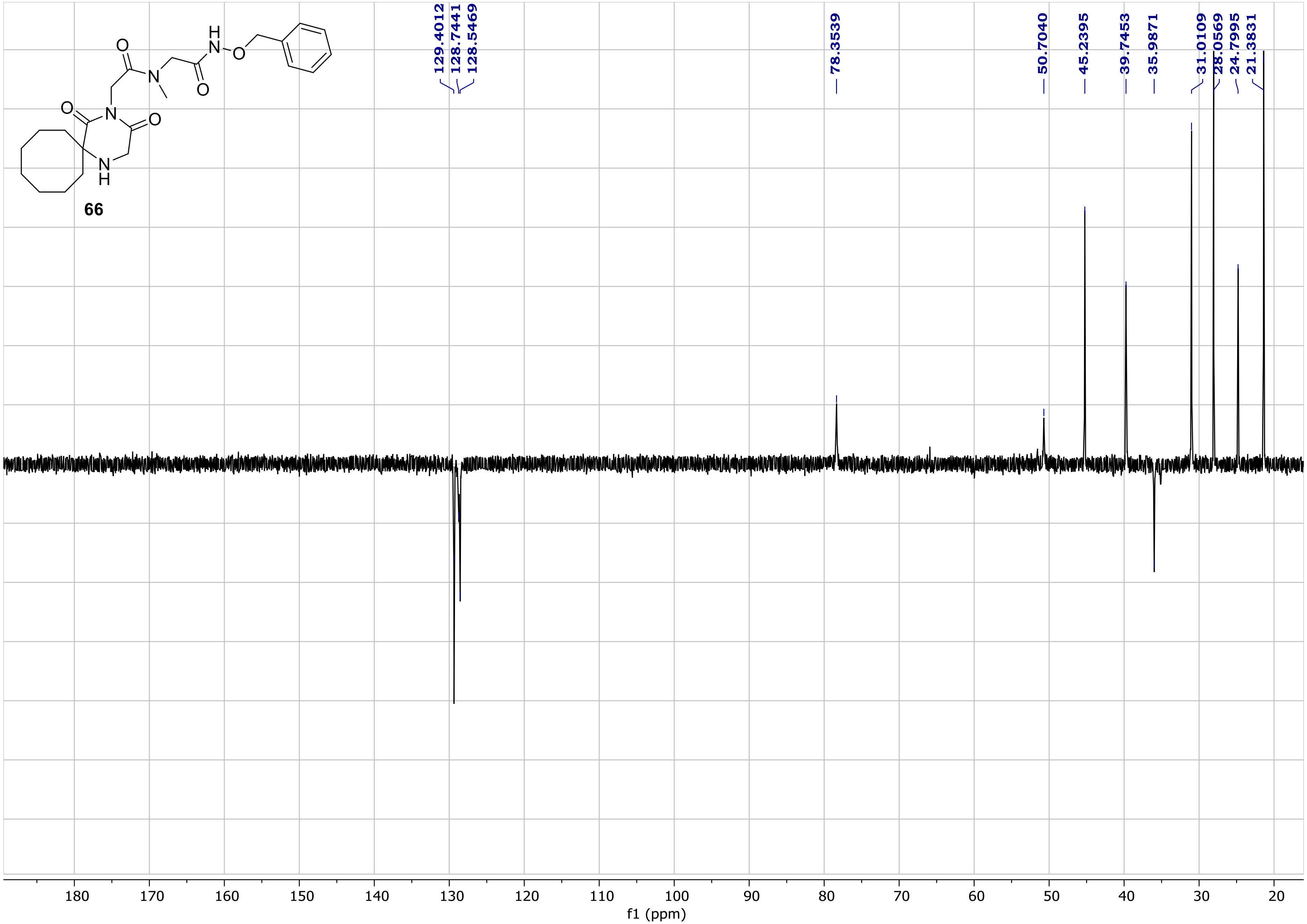

^{1}H NMR of **67** (400.11 MHz, $CDCl_3$)

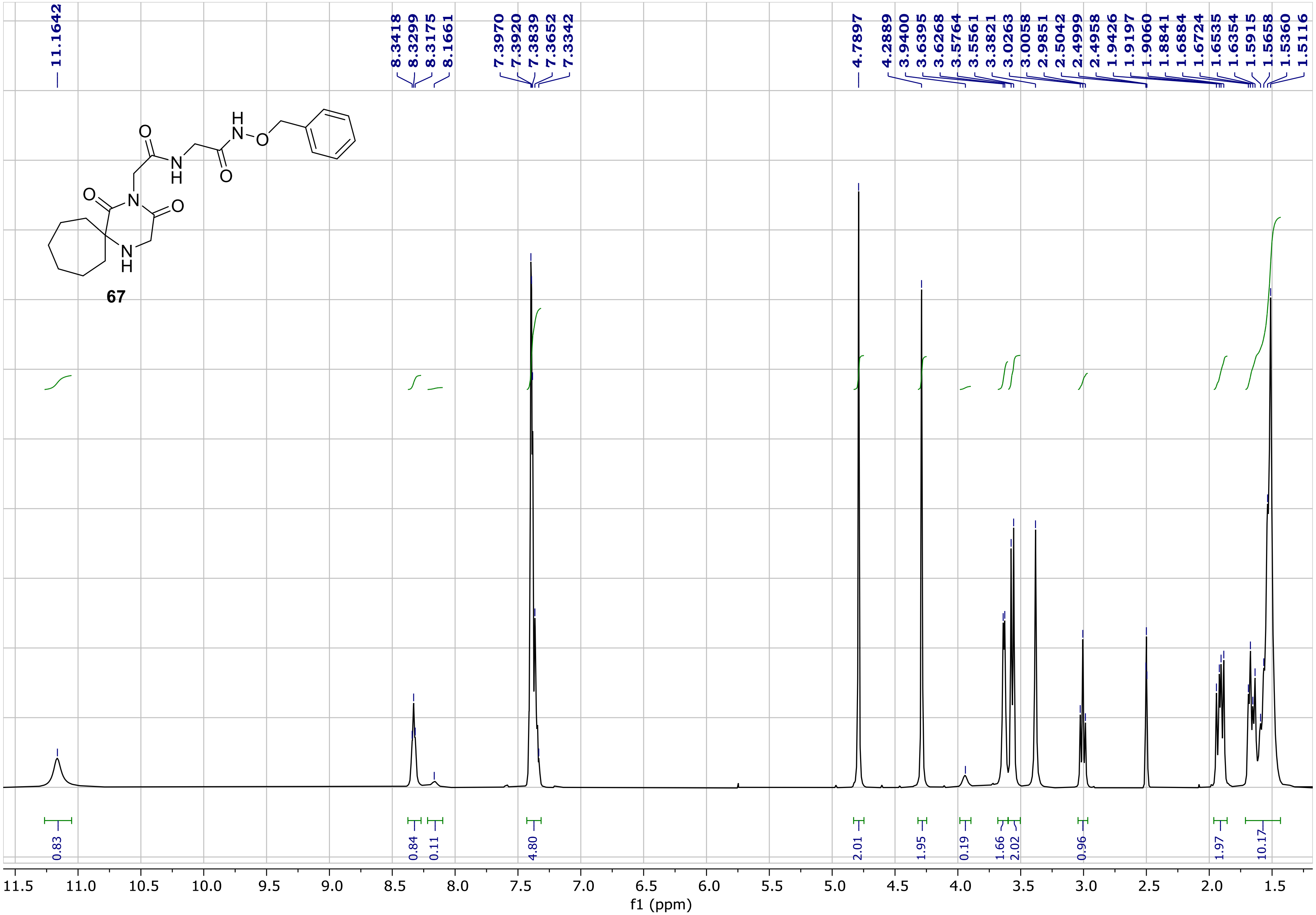

^{13}C NMR of **67** (50.32 MHz, $CDCl_3$)

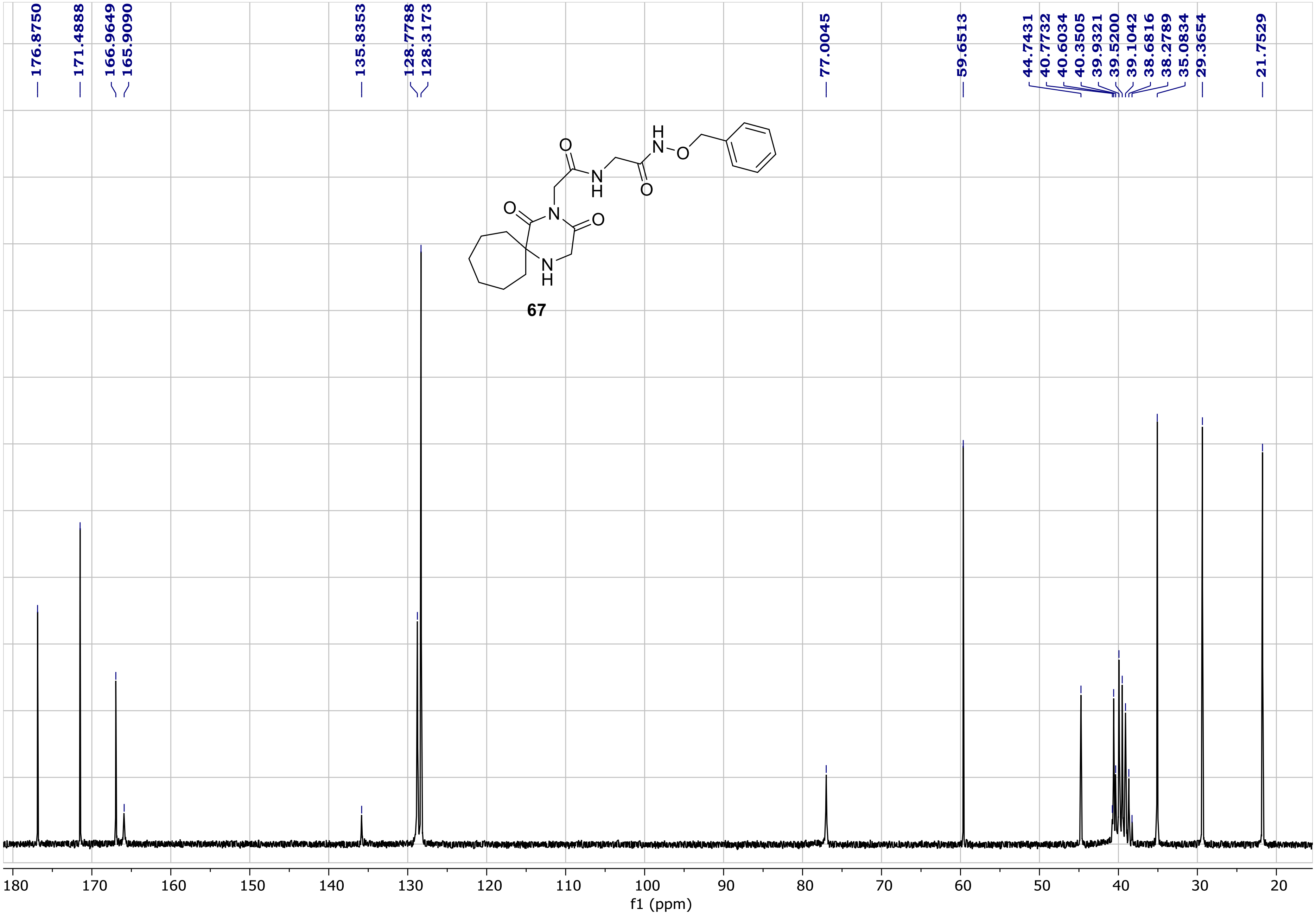

COSY NMR of **67** (400.11 MHz, $CDCl_3$)

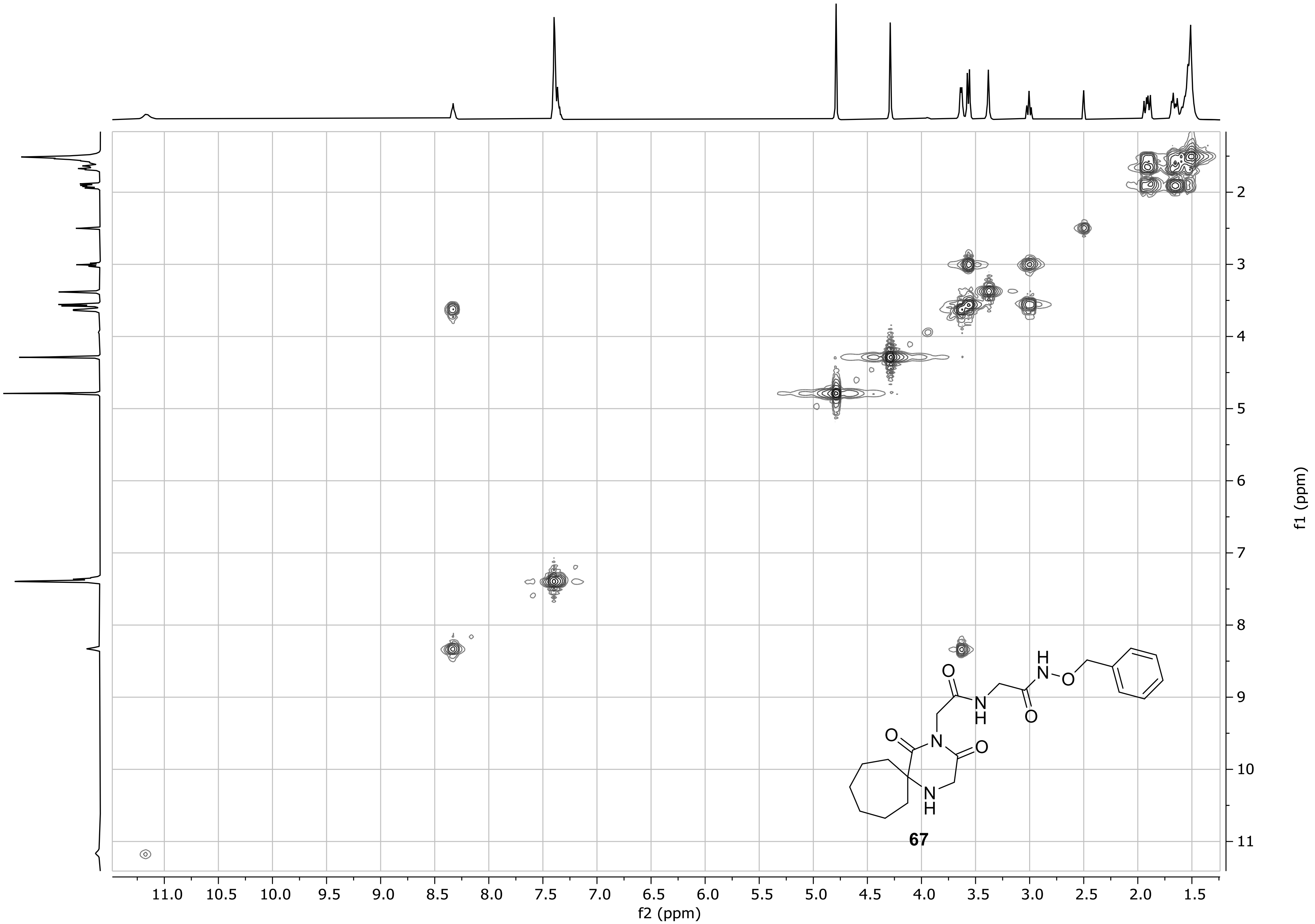

DEPT NMR of **67** (50.32 MHz, $CDCl_3$)

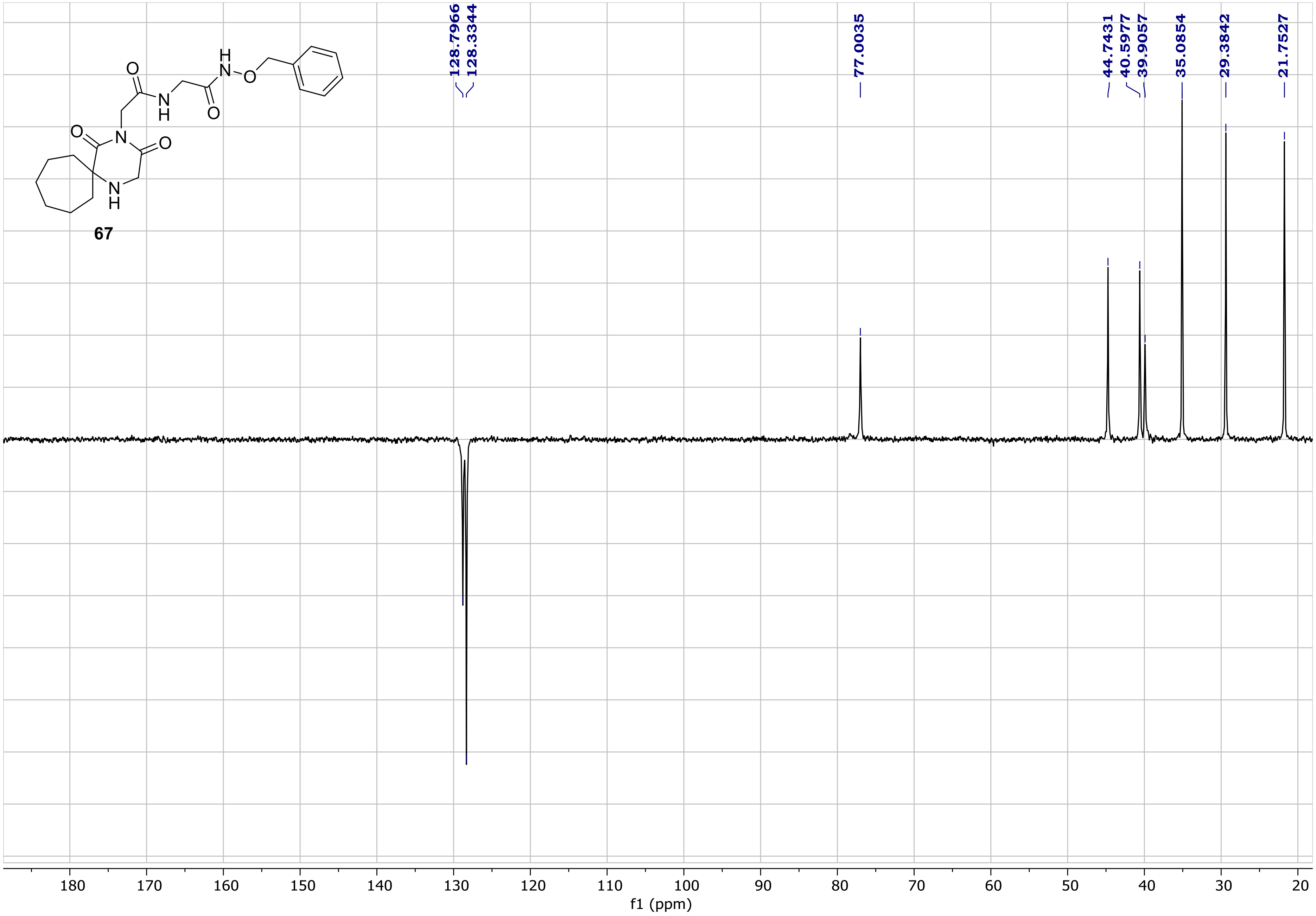

^{1}H NMR of **68** (400.11 MHz, $CDCl_3$)

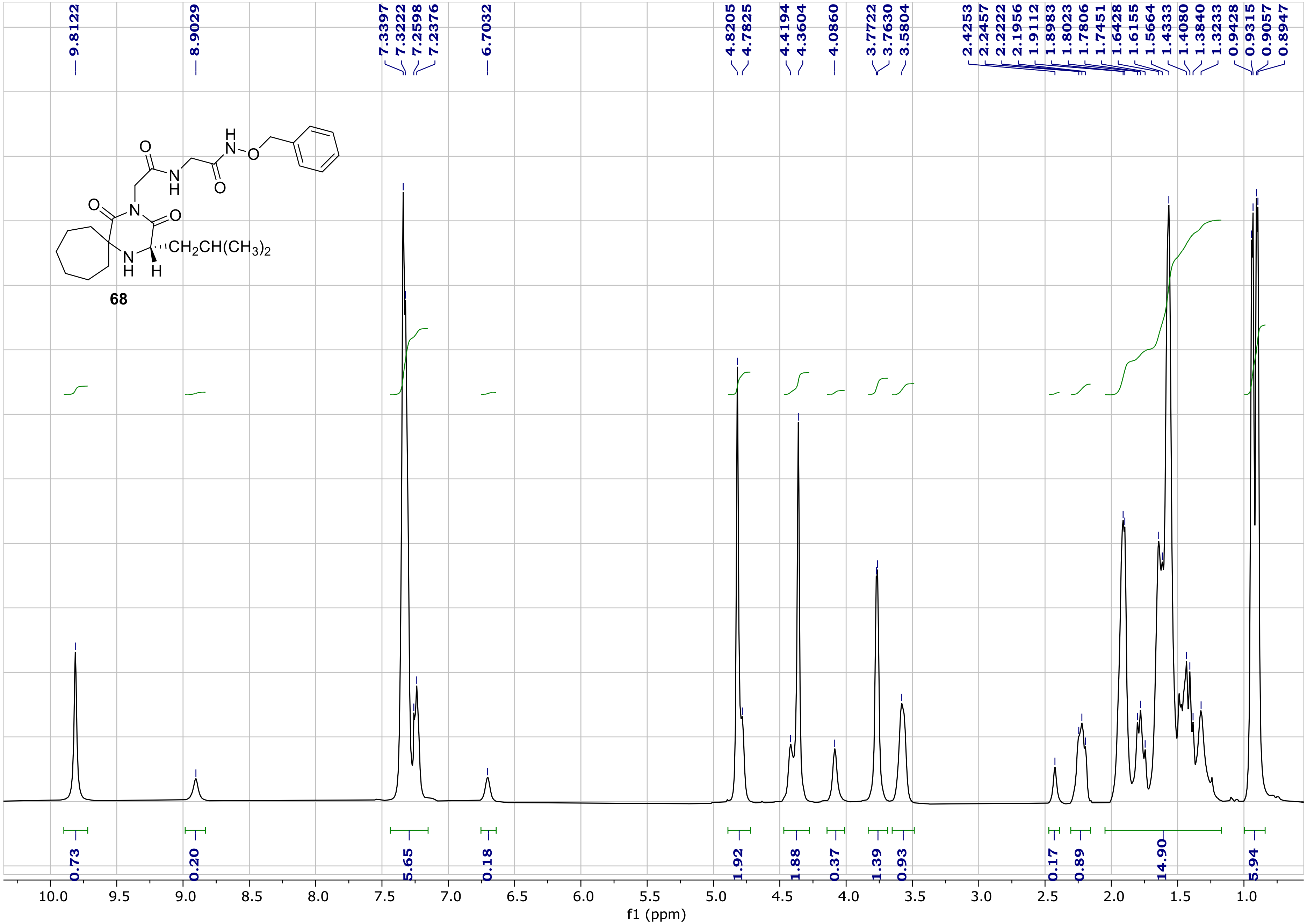

^{13}C NMR of **68** (50.32 MHz, $CDCl_3$)

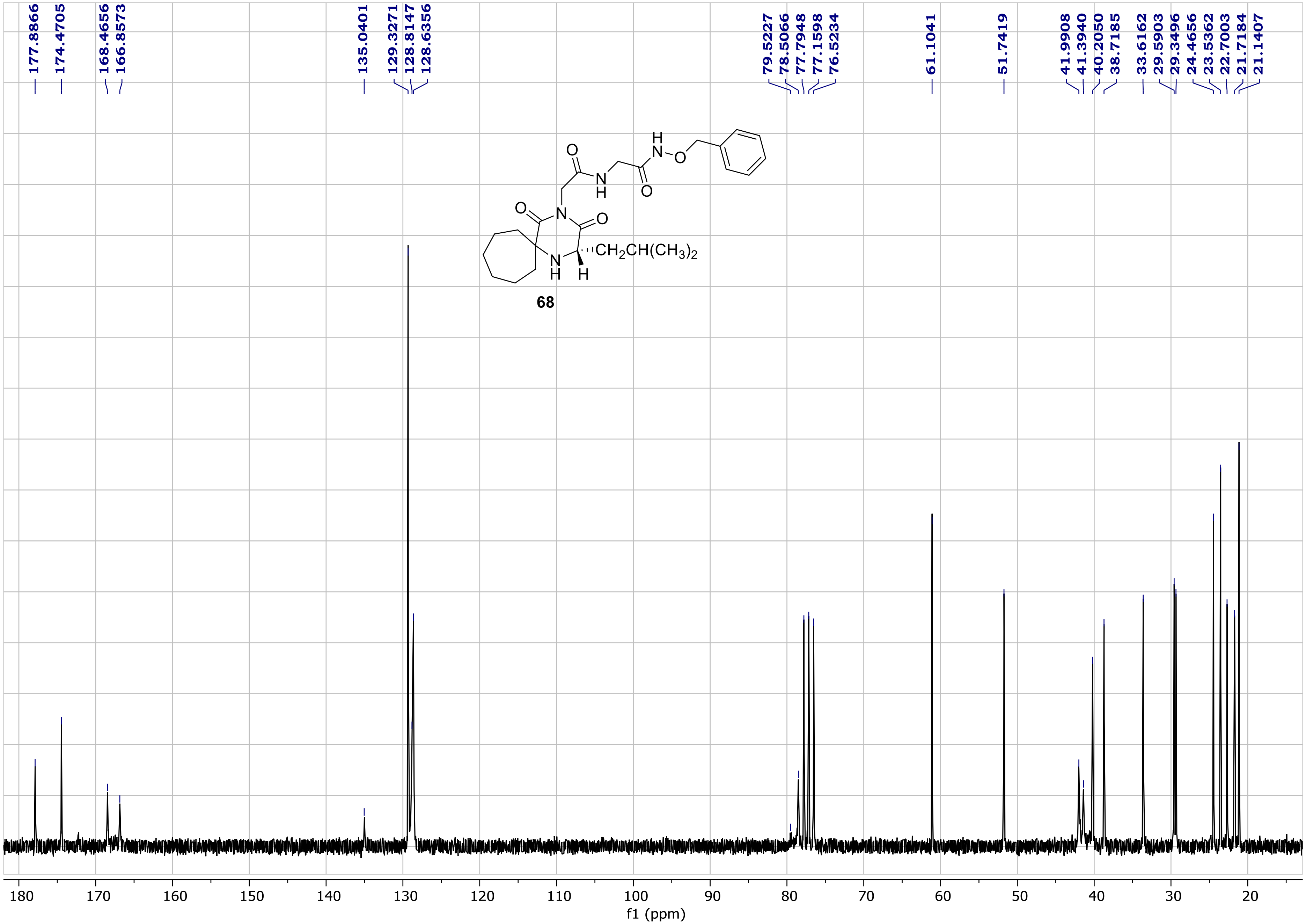

COSY NMR of **68** (400.11 MHz, $CDCl_3$)

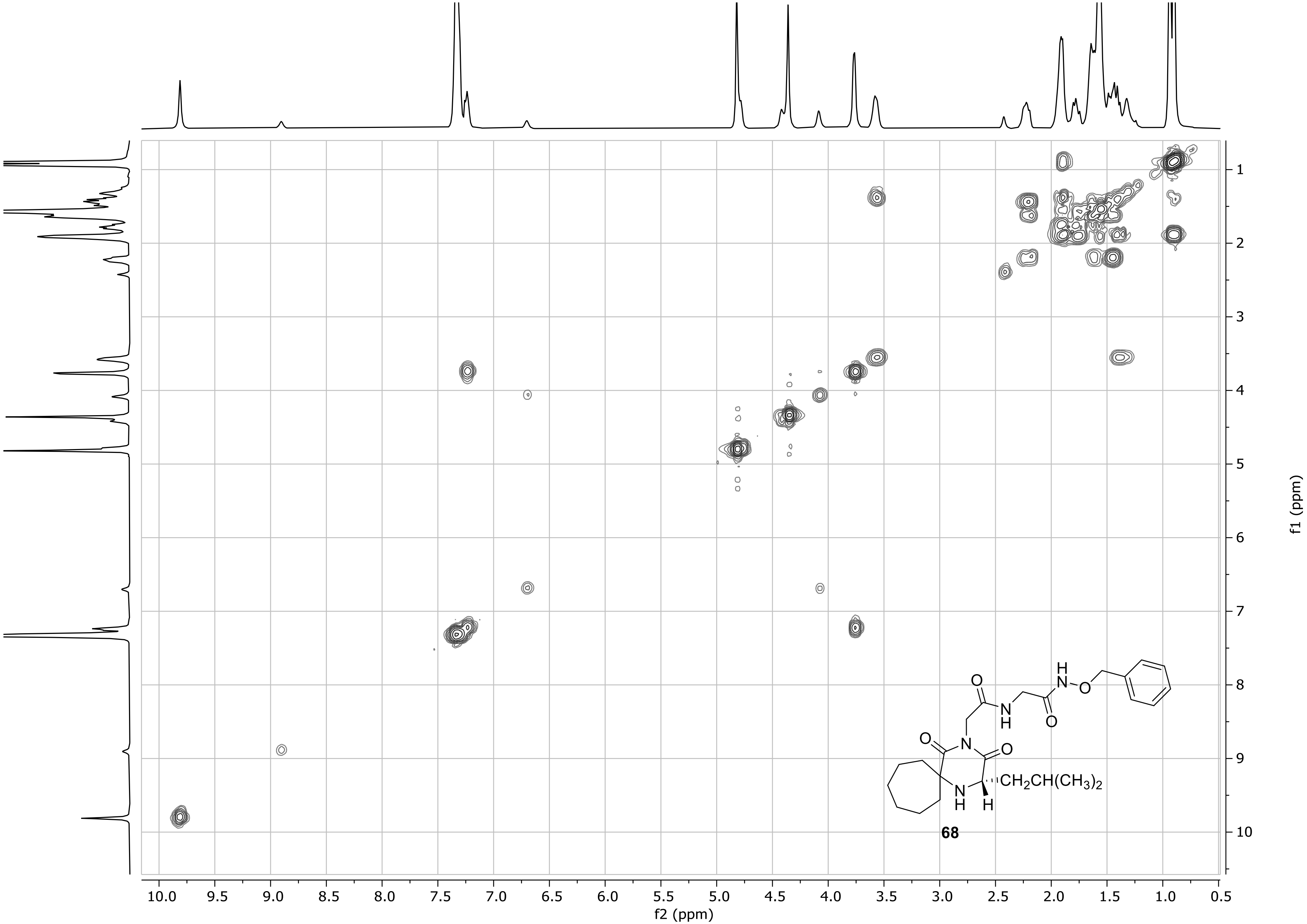

HSQC NMR of **68** (400.11 MHz, $CDCl_3$)

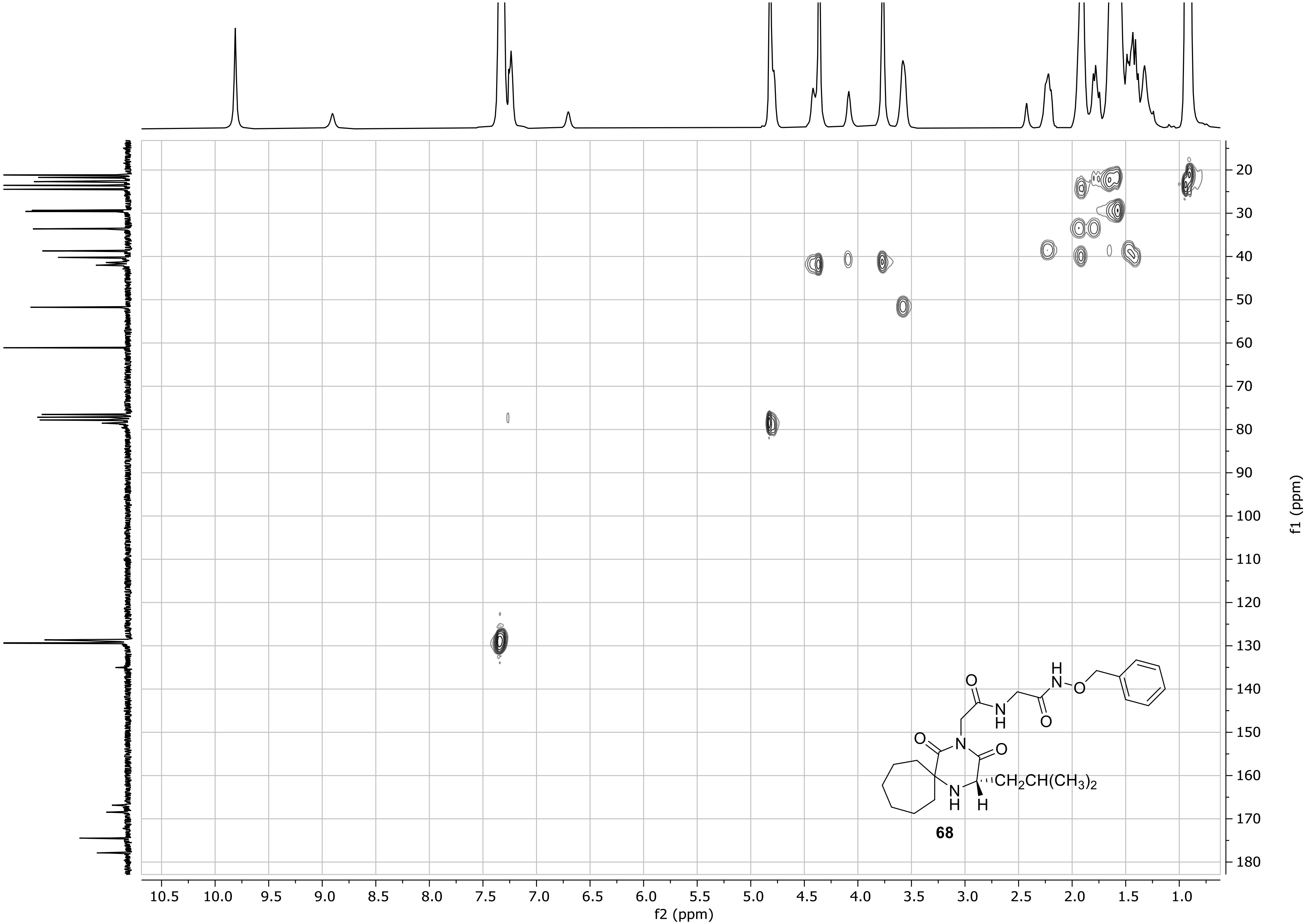

DEPT NMR of **68** (50.32 MHz, $CDCl_3$)

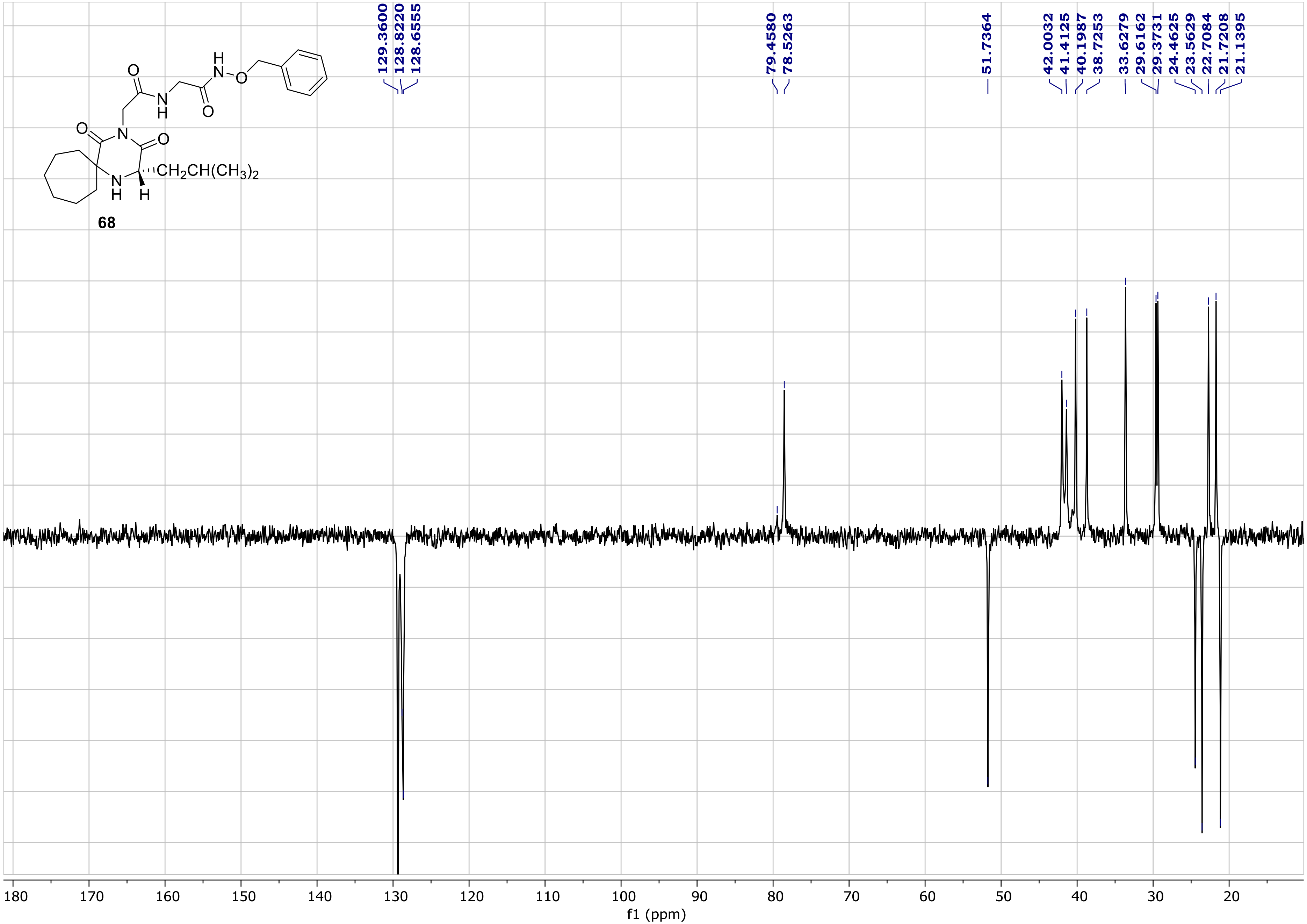

NOESY NMR of **18** (600.11 MHz, DMSO-d_6)

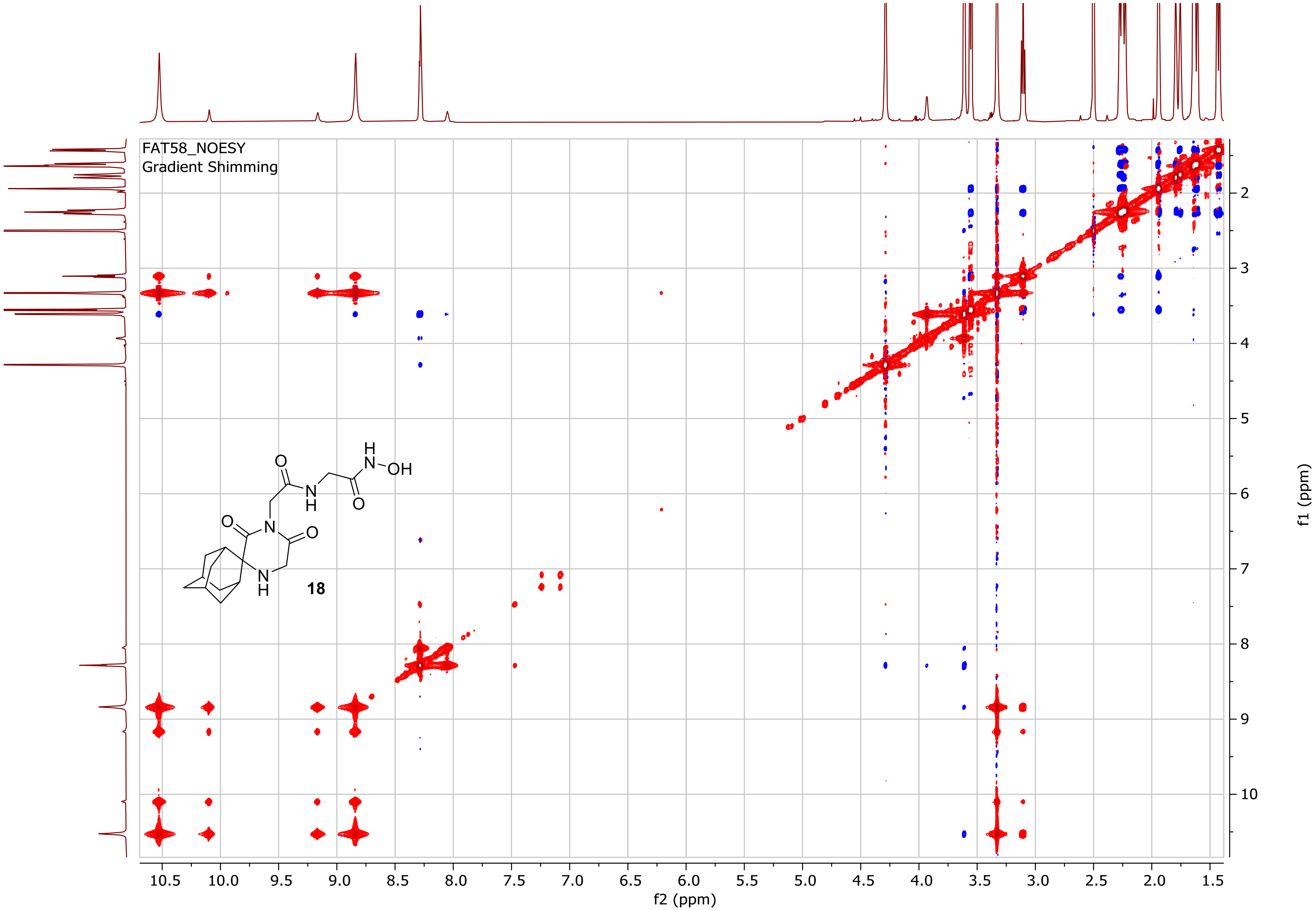

NOESY NMR of **21** (600.11 MHz, DMSO-d_6)

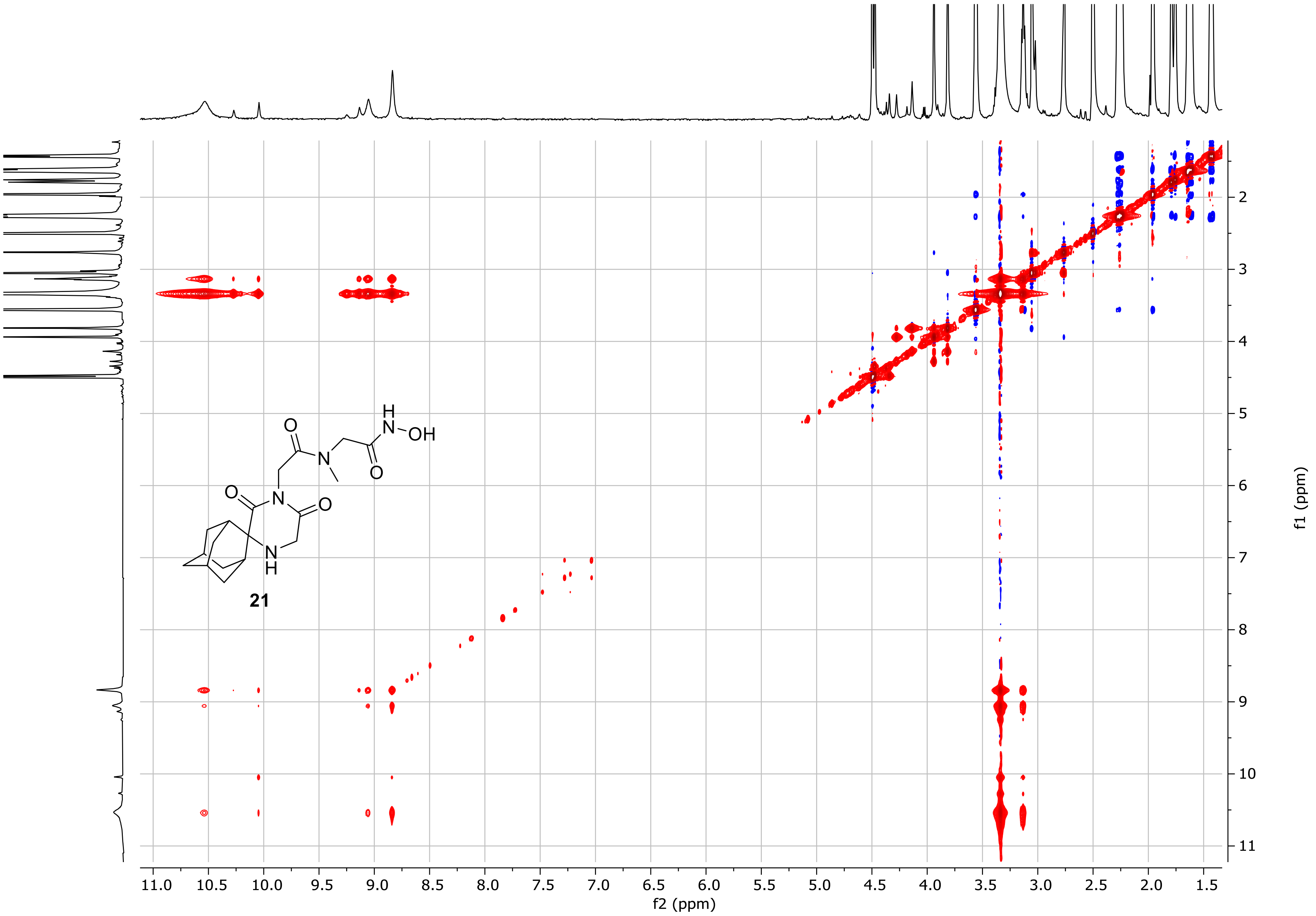

Supplement: Supplementary file 2 — ml4c00111_si_002.pdf [file ml4c00111_si_002.pdf]
